# Revealing cell cycle control by combining model-based detection of periodic expression with novel *cis*-regulatory descriptors

**DOI:** 10.1186/1752-0509-1-45

**Published:** 2007-10-16

**Authors:** Claes R Andersson, Torgeir R Hvidsten, Anders Isaksson, Mats G Gustafsson, Jan Komorowski

**Affiliations:** 1 The Linnaeus Centre for Bioinformatics, Uppsala University and Swedish University of Agricultural Sciences, Uppsala, Sweden; 2 Department of Medical Sciences, Uppsala University, Uppsala, Sweden; 3 Department of Engineering Sciences, Uppsala University, Uppsala, Sweden

## Abstract

**Background:**

We address the issue of explaining the presence or absence of phase-specific transcription in budding yeast cultures under different conditions. To this end we use a model-based detector of gene expression periodicity to divide genes into classes depending on their behavior in experiments using different synchronization methods. While computational inference of gene regulatory circuits typically relies on expression similarity (clustering) in order to find classes of potentially co-regulated genes, this method instead takes advantage of known time profile signatures related to the studied process.

**Results:**

We explain the regulatory mechanisms of the inferred periodic classes with *cis*-regulatory descriptors that combine upstream sequence motifs with experimentally determined binding of transcription factors. By systematic statistical analysis we show that periodic classes are best explained by combinations of descriptors rather than single descriptors, and that different combinations correspond to periodic expression in different classes. We also find evidence for additive regulation in that the combinations of *cis*-regulatory descriptors associated with genes periodically expressed in fewer conditions are frequently subsets of combinations associated with genes periodically expression in more conditions. Finally, we demonstrate that our approach retrieves combinations that are more specific towards known cell-cycle related regulators than the frequently used clustering approach.

**Conclusion:**

The results illustrate how a model-based approach to expression analysis may be particularly well suited to detect biologically relevant mechanisms. Our new approach makes it possible to provide more refined hypotheses about regulatory mechanisms of the cell cycle and it can easily be adjusted to reveal regulation of other, non-periodic, cellular processes.

## Background

In yeast several transcription factors and sequence motifs are known to ensure timely expression of genes whose products are required at specific phases in the cell cycle [1]. For instance, genes with the ECB motif upstream are predominantly transcribed in the M/G1 boundary of the cell cycle, while presence of the MCB motif is correlated with high expression in the G1 phase. Spellman *et al*. [2] used temperature-sensitive mutants of cdc15 and cdc28, as well as stimulation by the *α*-factor mating pheromone, to induce synchrony in budding yeast (*S. cerevisiae*). The aim was to identifying all cell cycle regulated genes in yeast by selecting genes that exhibited consistent periodic expression in cultures synchronized by these different methods. However, it has subsequently been noted that there are genes that appear to be periodically expressed only when some of the synchronization methods are used [3]. The different synchronization methods are bound to affect the system in different ways, and so it is to be expected that different dynamics follow the perturbation. Here we study this phenomenon of perturbation-dependent phase-specific expression and the corresponding *cis*-regulatory control structures in the yeast cell cycle. In doing so we devise a method in which prior information is incorporated in a model-based treatment of gene expression data, a method we expect to be useful in other analyses.

Several studies have used computational methods to uncover *cis*-regulatory control structures in yeast from high-throughput data sources. An important step was taken by Pilpel *et al*. [4] who observed that expression was more concordant for genes sharing two upstream sequence motifs than for genes sharing only one motif. This suggested that the expression program of the yeast genome in general is determined by combinations of regulatory elements and stimulated several computational studies aimed at identifying these regulatory mechanisms using sequence motifs and expression data. Segal *et al*. [5] used clusters of similarly expressed genes as initial regulatory modules, found common motifs for these modules and then iteratively refined them by reassigning genes whose promoter regions did not match the motif profile of their current module. Beer and Tavazoie [6] found a large number of sequence motifs that were overrepresented in a set of expression clusters, and inferred a statistical (probabilistic) relationship between these motifs and the clusters. Hvidsten *et al*. [7] and Wilczyński *et al*. [8] retrieved motif combinations by computing minimal sets of motifs necessary to separate genes in overlapping expression clusters. The common denominator for all these studies, as well as other related studies [9-13], is that expression similarity is characterized in terms of the presence of combinations of sequence motifs or other *cis*-regulatory information. The resulting sets of putatively co-regulated genes are then often evaluated using external data such as gene function annotations [14] or by considering additional experimental support such as binding of transcription factor to gene promoters [15,16]. A different route was taken by Segal *et al*. [17] and Pham *et al*. [18] who directly addressed the transcriptional network by using the notion that expression of genes (i.e. transcription factors) affect transcription of other genes in a direct, but not necessarily causative fashion. For instance, Segal *et al*. [17] constructed decision trees for co-regulated sets of genes (clusters) where the internal nodes in the tree were decisions based on the expression level of known transcription factors. This approach, as the above, relies on identifying groups of genes with similar expression. However, instead of explaining expression similarity with *cis*-regulatory descriptors, transcription factors were found whose joint expression pattern was concordant with that of a cluster of genes. Nevertheless, little information about putative physical interactions was provided since actual binding or occurrence of binding motifs was not included.

**Figure 1:**
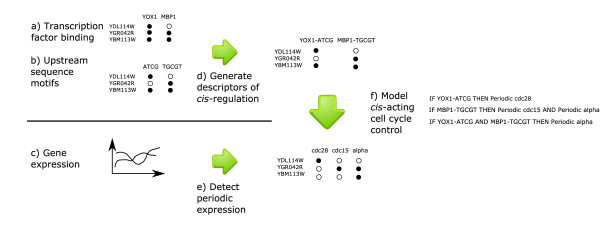
**Method overview**. We used genome-wide data including (**a**) binding of transcription factors in ChIP on chip experiments [16], (**b**) annotations of computationally inferred upstream sequence motifs [22] and (**c**) gene expression time profiles for different synchronization methods [2]. (**d**) The *cis*-regulatory information in **a **and **b **is combined by finding statistically associated combinations of transcription factors and motifs (i.e. *cis*-regulatory descriptors). (**e**) Periodic expression in each experiment is inferred from the temporal expression profiles. (**f**) Finally, machine learning is applied to model periodic expression. The model consists of rules associating *cis*-regulatory descriptors with patterns of periodic expression.

Although the previously reported clustering approaches generate interesting hypotheses on regulatory mechanisms, none of them make efficient use of any prior knowledge about the studied cellular process. This is in contrast to the model-based classification of expression profiles that we present here. Rather than using clustering, we employ a previously published Bayesian detector where a sinusoidal function and prior knowledge of cell division times is used to model periodic temporal profiles [19]. The detector is used to classify the temporal expression profiles as periodic or aperiodic for each of the three experiments using different synchronization methods published by Spellman *et al*. [2]. We argue that this class division is more suited for investigations of cell cycle regulation than a class division based on clustering (computed from, for example, Euclidean distance or correlation between expression profiles). With periodic expression as the criterion for class inclusion, the classes are directly associated with phase-specific regulation and, consequently, with the cell cycle machinery. When clustering is used, relationships between the classes and cellular processes may only be inferred from secondary data such as functional annotations. Furthermore, we show that the new approach yields understanding of regulation in terms of novel *cis*-regulatory descriptors. Each *cis*-regulatory descriptor is a binary variable that corresponds to the simultaneous presence or absence of an upstream sequence motif and observed binding of a transcription factor. Several of these interactions are supported in the literature. We then use a previously published rule-based method [7,8] to model the information available about the regulation of the periodic genes defined by the Bayesian detector as logical rules associating minimal combinations of descriptors with one or more of the periodic classes. An overview of the method is given in Figure 1.

Our results provide several fundamental observations about the regulation of the *S. cerevisiae *cell cycle. Firstly, we show that combinations of *cis*-regulatory descriptors are better at explaining the periodic classes than single descriptors. Secondly, we demonstrate that these combinations are specific to different periodic classes. Although others have argued that the variation in periodic behavior of the different synchronization studies is experimental noise [20,21], our results seem to indicate that the synchronization methods induce different perturbations that initially activate different regulatory mechanisms visible in the two first periods of the cell cycle. Thirdly, we show that combinations of regulatory descriptors specific to genes that are periodic in only one synchronization experiment are frequently subsets of combinations specific to genes periodic in two experiments, which again are subsets of combinations for genes periodic in all three experiments. This suggests that the periodic classes are regulated in an additive fashion. Finally, we show that combinations retrieved using the model-based detector are much more specific towards known phase-specific motifs and transcription factors than when employing conventional clustering. This illustrates that replacing clustering-based classification of dynamic gene expression patterns with model-based classification is advantageous for discovering the mechanisms underlying cellular control processes.

In the context of previous methods explaining expression clusters with *cis*-regulatory information, the novelty of our work chiefly consists in a model-based treatment of the dynamical gene expressions. This allows us to retrieve more relevant regulatory mechanisms than when using expression similarity (i.e. expression clustering). We show this by letting the same machine learning method first discover regulatory mechanism explaining periodic classes and then by trying the same process using expression similarity. Importantly, the use of periodic classes makes it easier for the investigator to interpret the hypotheses since they take the form of statements about how the genes expression behaves under different experimental conditions. We believe this is an improvement over methods where genes are grouped into expression clusters since these clusters only can be assigned semantics retrospectively, e.g. by looking for overrepresentation of annotations within the cluster or visual inspection of the expression pattern of the cluster. Given the systemic, genome-wide evaluation outlined above, we believe that our method provides reliable predictions for the regulatory mechanisms underlying periodic expression. In addition, the new approach introduced here is generic and may easily be adjusted to the analysis of other types of temporal gene expression profiles.

## Results

Our study includes several steps towards demonstrating how a model-based treatment of gene expression results in the retrieval of more focused and relevant regulatory mechanisms than what is obtained by expression profile clustering. First we describe how we divide the genes into different classes depending on which synchronization method induces periodicity in three synchronization experiments [2]. To this end, we use a previously published detector of periodic expression [19]. This detector only relies on the approximate period time and cyclicity of the process. Then we explain how we combine sequence motifs and transcription factor binding from ChIP-chip experiments [16] to obtain *cis*-regulatory descriptors. These are later used as the basic building blocks for constructing regulatory mechanisms. Having obtained descriptors of the genes that reflect possibilities of regulation, we perform statistical tests to show that it indeed is possible to explain the periodic classes with *cis*-regulatory descriptors. We start by testing for overrepresentation of single descriptors, and go on to pairs of descriptors. Since we see that pairs of descriptors explain the periodic classes better than single descriptors, we proceed by retrieving higher order combinations. To this end, we apply a previously published rule-based approach [7,8]. We interpret the rules and use them to explain the structure of the periodic classes. In particular, we observe that we can organize the regulatory mechanisms in a hierarchical structure that mimics the hierarchical structure of the periodic classes. We compare the regulatory mechanisms we obtain from applying our approach to periodic classes with those obtained from conventional expression clusters (i.e. the approach taken by almost all other studies in this field). This shows that our model-based approach enables the retrieval of regulatory mechanisms that are much more specific towards known cell cycle regulators. Finally, we illustrate some of the strongest regulatory mechanisms using a network-representation. We also present a Gene Ontology analysis of the periodic classes to further confirm the biological significance of our class division.

### Class generation

**Figure 2:**
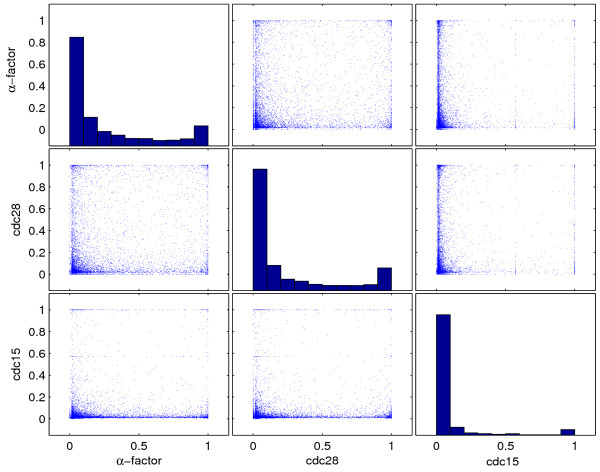
**Probability of periodic expression**. The detector used in the present paper calculates a probability of periodic expression. For each gene in the *S. cerevisiae *genome, the probability of periodic expression in the *α*-factor, *cdc28*, and *cdc15 *experiment of Spellman *et al*. [2] is plotted against the probability of periodicity in the other experiments. Also, the distribution of probabilities in each of the experiments is shown on the diagonal.

Periodic classes were computationally inferred from expression measurements. We applied a previously published detector of periodic expression [19] that took into account the approximate period time of the cell cycle in the *α*-factor, cdc15 and cdc28 experiments reported by Spellman *et al*. [2]. Basically, the detector calculates a statistic *s *which is restricted to the unit interval [0, 1] for each of the expression profiles where *s = *1 corresponds to absolute certainty in periodic expression and *s = *0 to no support for periodic expression (see Andersson *et al*. [19] and Methods for details). Figure 2 shows the score assigned in the *α*-factor, *cdc15 *and *cdc28 *experiment to each gene. It shows a strong pattern where the majority of genes is assigned a score close to either 0 or 1 in each experiment. However, the observed correlation is poor; there are many genes that show signs of periodic expression in the *cdc28 *experiment but not in the *α*-factor experiment and vice versa, and similarly for the *cdc15 *experiment.

From the expression data we select genes that have a high score for periodicity in at least one of the experiments and a very low score in the other, thus removing uncertain categorizations. Consequently we divide the genes from the microarray experiments into several classes, depending on whether they are detected as periodically expressed or not. To classify a gene as periodically expressed, one must choose a threshold for the detector statistic *s*. A trade-off must be made as to maximize the number of genes in the dataset while keeping the number of false positives at a minimum. We used the criteria *A *: *s *<*s*_*np *_= 0.1 and *B *: *s *> *s*_*p *_= 0.9 to classify each gene as periodic (if it satisfies criterion *B*) or not periodic (if it satisfies criterion *A*) in each of the experiments. The 2079 genes that fulfilled either of these criteria were then divided into eight classes labeled 000, 001,...,110, 111. Each digit in the label corresponds to *α*-factor, *cdc28 *and *cdc15 *from left to right and takes value 0 or 1 depending on whether criterion *A *or *B *is fulfilled for that gene in the corresponding experiment. In the following we shall refer to the classes of genes that are detected as periodically expressed in at least one experiment, i.e. all classes excluding 000, as the *periodic classes*.

The number of genes assigned to each class is shown in Table 1. With the strict criteria A and B, the number of genes that was included in the data set constitutes roughly 1/3 of all genes in the genome (2079 out of 6178 genes). Some classes contain very few genes, for instance, genes that are periodically expressed only in the *cdc*-based experiments (six genes). Here we note again that the two sided criterion results in robust class separability. Due to criterion *A *it is not the case that e.g. some of the genes in class 011 would have moved to class 111 if the threshold in criterion *B *was slightly lowered. Hence, the two sided criterion ensures that genes with uncertain periodicity are excluded from further analysis. The fact there are so few genes in class 111 is a testament to the high variability between the different synchronization methods. The actual number however depends on the specific thresholds chosen.

**Table 1:** Periodic classes

**Class label**	***α*-factor**	***cdc28***	***cdc15***	**Number of genes**
000	Not periodic	Not periodic	Not periodic	1490
001	Not periodic	Not periodic	Periodic	66
010	Not periodic	Periodic	Not periodic	185
011	Not periodic	Periodic	Periodic	6
100	Periodic	Not periodic	Not periodic	163
101	Periodic	Not periodic	Periodic	15
110	Periodic	Periodic	Not periodic	135
111	Periodic	Periodic	Periodic	19

Periodic classes. Class distribution inferred from expression data. All genes in the *S. cerevisiae *genome were classified into periodic or not periodic for each of the synchronization experiments of Spellman *et al*. [2].

### *Cis*-regulatory descriptors

We aim to describe the different periodic classes in terms of *cis*-regulatory descriptors. We produced such *cis*-regulatory descriptors using the compilation of upstream sequence motifs published by Hughes *et al*. [22] in combination with the genome-wide location analysis showing the binding of transcription factors to gene promoters published by Harbison *et al*. [16]. These datasets both contain noise. In particular, we expect a large number of the sequence motifs to be non-functional (i.e. false positives). Moreover, since bindings in the genome-wide location analysis are inferred from the measured signal strength using some arbitrary threshold, we would expect this data to contain both false positives and false negatives.

In order to automatically curate false positives we formed new descriptors of the form *X *- *Y *where *X *is a transcription factor and *Y *a sequence motif present at the same gene in the genome wide location analysis and upstream sequence motif data, respectively. We then filtered these transcription factor-motif pairs and retained pairs where the transcription factor was statistically associated with the motif or vice versa. To ensure that most of the motifs and transcription factors were included, we selected the three best scoring significant pairs (p-value < 0.05) for each transcription factor and each motif for further analysis (see Methods for details).

It was encouraging to see that known transcription factor-motif pairings such as *MCM1*-MCM1, *MBP1*-MCB, *SWI6*-MCB, *SWI4*-SCB, *YOX1*-ECB and *HIR2*-CCA were recovered. This strengthens our belief in the predicted novel relationships where the motif is putative or the association between the transcription factor and the motif has not been described earlier (e.g. *SWI*6-LYS14). Some illustrative examples are shown in Table 2 and 3. Note that the best scoring motif for transcription factor *ABF1 *is the ABF1 motif (Table 2).

**Table 2:** Regulatory descriptors – transcription factors

**Transcription factor**	**P-value**	**Sequence motif**
*YOX1*	2.25e-06	MCM1
	3.83e-05	ECB
	0.0034	m_organization_of_cell wall_orfnum2SD_n6
*UME6*	5.70e-114	m_meiosis_orfnum2SD_n3
	1.75e-70	Ume6 (URS1)
	1.74e-17	m_glyoxylate_cycle_orfnum2SD_n11
*ABF1*	6.5e-214	ABF1
	2.98e-09	Ume6 (URS1)
	7.8e-09	RPN4

The three most significantly co-occuring sequence motifs for the *YOX1, UME6 *and *ABF1 *transcription factors.

**Table 3:** Regulatory descriptors – sequence motifs

**Sequence motif**	**P-value**	**Transcription factor**
SCB	3.67e-08	*AZF1*
	1.02e-05	*UME6*
	3.31e-05	*SWI4*
SFF	1.14e-10	*FKH2*
	9.26e-10	*FKH1*
	1.92e-05	*HIR1*

The three most significantly co-occuring transcription factors for the SCB and SFF sequence motifs.

Inclusion of a pair in the further analysis is due either to the transcription factor being among the three best scoring factors for the motif or vice versa. For example, note in Table 3 that binding of transcription factor *UME6*, which regulates early meiotic genes, is one of the three most significant transcription factors co-located with the SCB motif. However, the SCB motif is not one of the three most significant motifs that is co-located with the binding of *UME6 *(Table 2). Furthermore, the p-value is an indicator of the strength of the association. For example, we see a pair with *UME6 *which includes the putative motif m_glyoxylate_cycle_orfnum2SD_n11. This motif was inferred from a group of genes known to be involved in the glyoxylate cycle by Hughes *et al*. [22]. However, the p-value of this pair is much higher than the p-value of the two first, both of which are known to be associated with *UME6*. Thus one may have less confidence in this predicted interaction (see Additional file 1 for all pairs and p-values).

We described each gene in terms of the transcription factor-motif pairs found upstream of that gene. Since not all the genes included in the periodic classes were covered by these pairs, the size of the periodic classes shrinked. The resulting class distribution of the 1644 covered genes is shown in Table 4. In total, 1459 transcription factor-motif pairs were kept, resulting in a list (vector) of 1459 binary elements, each indicating the presence or absence of a particular pair. It is important to note that these *cis*-regulatory descriptors were formed without using expression data, or more specifically, without any direct relation to the classes inferred from the microarray experiments.

**Table 4:** Periodic classes

**Class label**	**Number of genes**
000	1173
001	55
010	140
011	4
100	127
101	11
110	115
111	19
Sum	1644

The class distribution after all genes were represented in terms of the regulatory descriptors. Compared to Table 1, 435 genes did not match any regulatory descriptor (only 118 of these were periodic).

### Describing expression with *cis*-regulatory descriptors

If the *cis*-regulatory descriptors explain the regulation of the periodic classes, it should be possible to observe a statistical dependency between the presence of a transcription factor-motif pair upstream (i.e. the match of a *cis*-regulatory descriptor) and the class designation of each gene. To investigate this, we calculated the probability that a descriptor matches by chance at least the observed number of genes from the same class. A p-value lower than 0.05 indicates a significant overrepresentation of this descriptor in the promoters of genes from that specific class. This calculation was done for all descriptors that were associated with genes in each of the periodic classes. However, these p-values are not informative in themselves; due to the large number of descriptors one would expect to find many overrepresentations by chance. If there is no relationship between any of the descriptors and the class designation, the expected number of descriptors to have a p-value less than 0.05 is 0.05 × *n*, where *n *is the number of descriptors associated with the class. Thus, a ratio of observed significant descriptors over expected significant descriptors greater than 1.0 will indicate an overrepresentation of descriptors that are significant at the level of 0.05. This ratio can also be complemented with a p-value (see Methods). Table 5 shows the ratio between expected and observed numbers and the corresponding p-values for each periodic class.

**Table 5:** Indications of significant *single *regulatory descriptors

**Class label**	**Observed/Expected**	**P-value**	**Number of genes**
001	1.1	0.35	55
010	0.77	0.9	140
011	9.1	1.3e-11	4
100	0.85	0.84	127
101	4.42	1.55e-9	11
110	2.2	1.30e-10	115
111	3.7	4.79e-13	19

The second column contains for each class the ratios of the number of significant descriptors at the significance level of 0.05 to the expected number of significant descriptors for single descriptors. Also displayed (third column) is the p-value of the ratio. To aid the reader the number of genes in each class is included in the last column.

As the ratio is close to 1.0 for classes 001, 010 and 100, it appears that single descriptors are not informative for these classes. This is also indicated by the associated high p-values. However, all classes of genes showing periodicity in more than one experiment have more significantly overrepresented descriptors than would be expected by chance. Furthermore, in view of Pilpel *et al *[4] we do not expect it to be possible to differentiate between all classes on the basis of single descriptors. We find it more likely that transcription factors will interact with each other. To see if this appears to be the case, the same calculation was done for all *pairs *of regulatory descriptors. As is seen in Table 6, many more pairs of descriptors than single descriptors are significant compared to what would be expected by chance. The exception is class 100, which actually has fewer significant pairs of descriptors than would be expected by chance. A possible interpretation could be that class 100 is very homogeneous with respect to the regulatory descriptors found in this class, i.e. a few pairs explain a very large part of the regulatory mechanisms for these genes. Furthermore, class 010 remains close to the chance distribution. Thus, it is not possible to statistically ascertain that this class of genes is different from other genes in the genome based on the available upstream regulatory information.

**Table 6:** Indications of significant *pairs *of regulatory descriptors

**Class label**	**Observed/Expected**	**P-value**	**Number of genes**
001	7.3	5.0e-10	55
010	0.95	0.91	140
011	19.3	< 1e-20	4
100	0.36	1.0	7
101	19.1	2.5e-12	11
110	1.6	1.44e-10	115
111	17.1	< 1e-20	19

The ratio of the number of significant descriptors at a significance level of 0.05 to the expected number of significant descriptors for pairs of descriptors. To aid the reader the number of genes in each class is included in the last column.

We were also interested in whether combinations of descriptors were specific to genes only clearly periodically expressed in a subset of experiments. Thus, we performed the calculation again, this time with a background distribution of descriptor pairs formed only from genes in the periodic classes instead of all 1644 genes (i.e. class 000 was excluded from the analysis). In this way we eliminated the possibility that the overrepresentation we saw in periodic classes was the result of genes observed as periodically expressed in at least one experiment, but that the actual assignment of genes into periodic classes was random. As is seen in Table 7, the pattern of overrepresentation remains and thus provides evidence for a relationship between descriptor pairs and class membership beyond that of the genes observed as periodically expressed in at least one experiment. The fact that the ratio drops is expected. As is shown by a Gene Ontology [14] analysis of the periodic classes later in this study, the genes of class 000 participate in fundamentally different processes than the genes of the periodic classes. Consequently, one expect periodic genes and aperiodic genes to be regulated differently in a more fundamental way than different periodic classes are.

**Table 7:** Indications of over representation of pairs exclusively within the periodic classes.

**Class label**	**Observed/Expected**	**P-value**	**Number of genes**
001	0.86	0.71	55
010	0.31	1.0	140
011	18.18	1.02e-12	4
100	0.0	1.0	127
101	12.9	1.3e-14	11
110	1.81	0.0013	115
111	6.7	3.8e-14	19

The ratio of significant number of descriptors at a significance level of 0.05 to the expected number for pairs of descriptors. To aid the reader the number of genes in each class is included in the last column.

### Combinations of *cis*-regulatory descriptors

Having ascertained that there is a statistically significant relationship between the *cis*-regulatory descriptors and the periodic classes of genes, we proceeded to apply the rough set methodology of Hvidsten *et al*. [7] to recover higher-order combinations of descriptors. Importantly, these combinations are minimal in terms of the number of descriptors required to discriminate genes in different classes with a user defined success rate *α *≥ 95%. The resulting combinations can be presented in the form of rules, such as "**IF **Descriptor 1 *AND *Descriptor 2 **THEN **Periodic(101) *OR *Periodic(110)", meaning that Descriptor 1 and Descriptor 2 are found co-located at gene promoters in classes 101 and 110, and only those.

All rules induced by the method are ranked according to their p-values. Such p-values are calculated as the probability that the descriptor combination in the left hand side of the rule *by chance *matches at least the observed number of genes in the class found on the right-hand side (see Additional file 2 for all rules and p-values).

From this output we find some particularly interesting examples of regulatory mechanisms (Table 8). E.g. the rule "**IF ***MBP1*-STRE' *AND SWI6*-MCB **THEN **Periodic(110)", which states that if the upstream region of a gene is bound by *MBP1 *and *SWI6 *in the Harbison *et al*. [16] experiment and it has the STRE' and MCB motifs upstream according to Hughes *et al*. [22], it is periodically expressed when the yeast cells are synchronized using *α*-factor and *cdc28*, but not *cdc15*. This particular rule has a right-hand side support of 2 (i.e. it applies to two genes) and a p-value of 0.0049. Furthermore, this rule finds support in the literature. *SWI6 *and *MBP1 *together form the MBF complex which regulates late G1-genes and binds the MCB motif [23]. Another known interaction recovered in class 110 is the combination *SWI6*-LYS14 and *SWI4*-SCB (support = 3, p-value = 0.00033). It is known that *SWI4 *and *SWI6 *form the SBF-complex which binds the SCB motif and regulates G1-specific transcription [23].

**Table 8:** Known interactions

**Rule**	**Support**	**P-value**	**Reference**
**IF ***MBP1*-STRE' *AND SWI6*-MCB **THEN **Periodic(110)	2	0.0049	[23]
**IF ***SWI6*-LYS14 *AND SWI4*-SCB **THEN **Periodic(110)	3	0.00033	[23]

Two examples of rules describing regulatory mechanisms that are known in the litterature.

### A hierarchy of regulation

**Figure 3:**
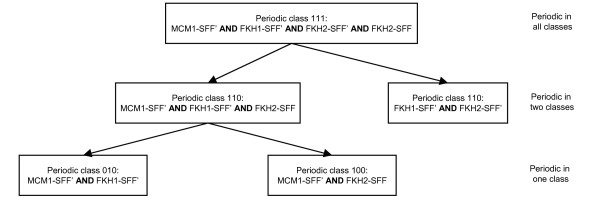
**Regulatory hierarchy**. A part of the hierarchy formed from combinations of *cis*-regulatory descriptors that are subsets of the combination *MCM1-SFF' FKH1-SFF' FKH2-SFF' FKH2-SFF*. Note that the hierarchical structure of the combinations are matched by a hierarchical structure of the corresponding class labels (i.e. the classes from which each combination was induced).

Of particular interest are rules describing higher order combinations since they cannot be studied exhaustively in a reasonable time frame (as was done for pairs of descriptors earlier in this work). The rough set methodology used here allows retrieving the most interesting higher order combinations in terms of their ability to discern the different classes. The average length and number of combinations retrieved in each periodic class is shown in Table 9. No class specific combinations were recovered for class 011 (not included in the table). This comes as no surprise since there are only four genes in this class. As is seen in Table 9, the average length of combinations in rules tends towards two for all classes. However, some higher order combinations are found. For example, in class 111 we have a combination of transcription factors *FKH1, FKH2 *and *MCM1*. It is known that *MCM1 *interacts with *FKH1 *or *FKH2 *in controlling the transcription of G2/M genes, and that *FKH1 *and *FKH2 *bind at the SFF motif [24]. It was interesting to find that this particular combination was specific to class 111, while subsets of the combination were found in other classes (shown in Figure 3). Such patterns, where different subparts of a regulatory combination are present in different classes, suggest a hierarchy of regulation. Intrigued by this observation, we calculated the fraction of combinations found in each periodic class for which subsets could be found in other classes. As is seen in Table 10, more of the combinations of class 111 have subsets in other classes, followed by 110. It is striking that even though 1398 combinations specific to 010 are retrieved, only 59% had a subset that could be found in other classes. In class 111, 76% of the 166 combinations have subsets in other classes. Class 110 is perhaps a better example since the sample size (1692) is comparable to the sample size for class 010. 68% of the combinations in this class are supersets of combinations in other classes. Consequently, the trend seen in Table 10 suggests additive regulation, that is, when genes are periodically expressed under more conditions, more of the inferred combinations consist of supersets of combinations specific to genes periodically expressed under a smaller set of conditions (see Additional file 3 for all hierarchies).

**Table 9:** Length of rules

**Class label**	**Average length**	**Number of combinations**
001	1.72	55
010	2.00	1398
100	1.82	861
101	1.25	4
110	2.06	1692
111	2.02	166

Distribution of the average length of a *cis*-regulatory descriptor combination in rules and the number of combinations retrieved from each periodic class.

**Table 10:** Regulatory hierarchy

**Class label**	**Fraction**	**Number of combinations**
001	0.48	55
010	0.59	1398
100	0.59	861
101	0.25	4
110	0.68	1692
111	0.76	166

Distribution of the fraction of combinations in rules that had a subset in another periodic class.

### Comparison with clustering

**Figure 4:**
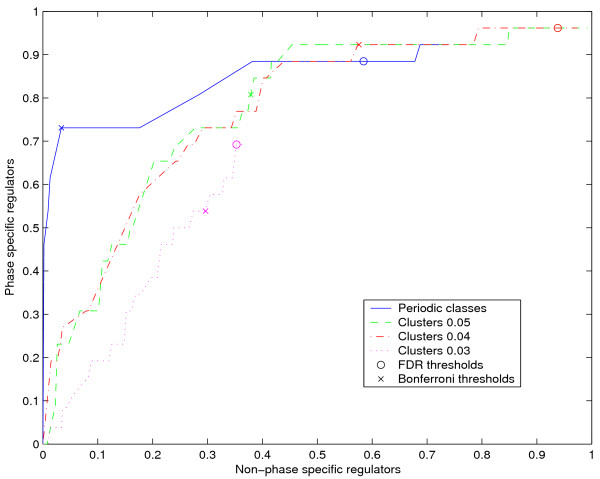
**Specificity towards cell cycle regulators**. Fraction of cell cycle regulators included in rules versus fraction of non-cell cycle regulators included in rules for different significance threshold. "Periodicity" refers to the method based on periodic classes presented here, "Cluster *d" *refers to applying the method of Hvidsten *et al*. [7] using the Euclidean distances *d *for including a gene in a cluster. Bonferroni (BF) and FDR corrected thresholds (at the 0.05 level) are shown for each method (Note that the FDR thresholds for "Clusters 0.04" and "Clusters 0.05" are located at the same point).

Previous work in this area has used clustering to obtain groups of genes expected to have similar regulation and then proceeded by using machine learning techniques to obtain combinations of sequence motifs that explain that grouping. To see how the class division used here compared to that of clustering we applied the clustering method of Hvidsten *et al*. [7] to the Spellman *et al*. [2] data. The principal difference is that combinations are recovered using classes defined by expression similarity. Specifically, genes belong to the same class if the Euclidean distance between the expression profiles is less than a predefined threshold *d*. Since good validation sets of known regulatory modules are not available, we needed a suitable performance measure for comparing the different methods. We argue that a relevant measure in this case is the degree to which already known cell cycle regulators, both transcription factors and sequence motifs, are included in the combinations retrieved by the different methods. Out of the motifs in the Hughes *et al*. [22] data set, we defined CCA, ECB, MCB, SWI5, SCB, SFF, SFF', MCM1 and MCM1' as cell cycle motifs after reviewing the literature. Furthermore, from the literature we defined the following transcription factors in the Harbison *et al*. [16] data as cell cycle regulating: *ACE2, FKH1, FKH2, GTS1, HIR1, HIR2, MBP1, MCM1, NDD1, STB1, SWI4, SWI5, SWI6, XBP1, YBR267W, YHP1 *and *YOX1*. We can calculate the fraction of all these cell cycle regulators that are present in all significant rules at a specific p-value threshold as well as the fraction of regulators with no known relationship to the cell cycle. Figure 4 shows a plot of these fractions generated by varying the p-value significance threshold from 0 to 1.

As is seen in Figure 4, the method proposed in this paper is more specific towards cell cycle-related regulators than the clustering approach. For example, for the model-based approach one may select a significance level (p < 6.77e-06) so that 46% of the known cell cycle regulators are included in the rules but virtually none of the other regulators. Clearly, a similar specificity towards cell cycle regulators is not possible using the clustering approach, even when the cluster inclusion threshold *d *is made stricter. In fact, the specificity degrades when the criterion becomes too strict (i.e. *d = *0.03). This may be explained by the fact that tighter inclusion thresholds give smaller groups from which it is more difficult to recover significant associations between different *cis*-regulatory descriptors and expression similarity. Nonetheless, the expression data under consideration mostly contain periodic signals. This is apparent from the fact that the combinations retrieved using the clustering method also are geared towards cell cycle regulators. In fact, a method that selected combinations of *cis*-regulatory descriptors randomly would produce a curve that approximately follows a straight line between (0, 0) and (1, 1). The clustering approach, however, is notably different from such a straight line. We also visualized the points on the curves that correspond to the p-value thresholds that would have been chosen with Bonferroni (BF) or FDR correction for multiple hypothesis tests (both at the 0.05 level). As can be seen in Figure 4, the model-based approach yields higher specificity than the clustering method when the BF threshold is chosen. Also, as expected, the FDR procedure is less strict than BF. However, the important thing to note is that the rules with the lowest p-values are highly enriched for phase-specific regulators.

### Examples of putative mechanisms for cell cycle control

**Figure 5:**
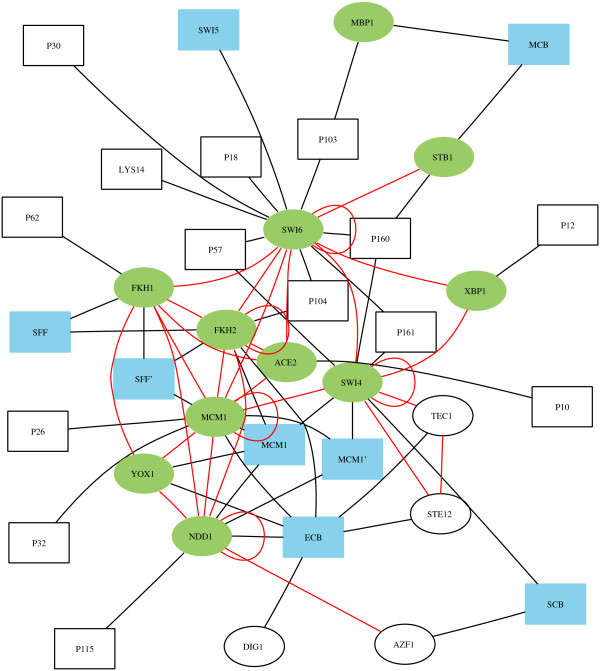
**Examples of cell cycle control**. Transcription factors (ellipses) are linked by (red) edges if they appear in the same combination at p-value threshold 0.000195. The corresponding sequence motifs (rectangles) occurring in *cis*-regulatory descriptors with these transcription factors are also shown, and black edges indicate with which factors they belong. Green indicate known phase-specific factors, while blue indicate known phase-specific motifs. Motifs named P# are putative motifs (see Additional file 4). Thus, for instance, one sees that the forkhead proteins *FKH1 *and *FKH2 *bind to genes that have the SFF and SFF' motifs and tend to bind simultaneously.

Given the system-wide evaluation presented so far, we have reason to believe that many of the combinations retrieved may offer new insight into the specific regulation of cell cycle-related genes. It might be particularly interesting to look at *cis*-regulatory descriptor combinations at a p-value threshold that we know will include mostly known phase-specific regulators. Combinations of these known phase-specific regulators as well as of known phase-specific regulators and other regulators may provide testable hypotheses explaining the selective regulation of periodic expression. The point (0.034, 0.73) on the curve representing our method in Figure 4 (accidentally the same as the Bonferroni point) is a good example. This point is associated with 145 rules with p-value lower than 0.000195. These rules include 19 of the 26 known phase specific regulators (73%) and 18 other regulators (3.4%). Furthermore, they describe 24% of the genes in the periodic classes. Figure 5 shows the co-occuring transcription factors in these rules. The figure mostly explains the regulation of genes in classes 011 and 111 since these classes are associated with the most significant combinations of *cis*-regulatory descriptors. Consequently, lower thresholds need to be chosen if one wants to study the specific regulatory mechanisms of other classes. Thresholds that balance the number of known phase-specific regulators against the number of other regulators may be obtained from the class-specific curves similar to that of Figure 4 (for motive numbers see additional file 4).

### Biologically meaningful classes

It is possible that the different synchronization methods evoke detectable periodic signals from functionally different groups. We analyzed Gene Ontology [14] "biological processes" associated with the classes to see if some annotations occurred more often within a class than would be expected by chance. One immediately notices that class 000 predominantly consists of metabolic genes. For example, among the 384 genes annotated to "protein metabolism" (GO:0019538), 319 genes belong to class 000 (an overrepresentation in class 000 at p-value 5.47*e *- 7).

Considering the seven periodic classes together, one notes a large number of "cell cycle" (GO:0007049) annotations. 57 out of 120 genes annotated to "cell cycle" are found in the periodic classes (an overrepresentation at p-value 1.2*e *- 4). Notably, the term "cell cycle" is a rather general designation, and it is therefore expected that not all genes annotated to this term are periodically expressed. On the other hand, many genes not annotated to "cell cycle" could still be periodic. For example, although 49 out of 58 genes annotated to "protein catabolism" (GO:0030163) were found in class 000, 4 out of 5 genes annotated to the more specific subterm "cyclin catabolism" (GO:0008054), a cell cycle-related process, were found in the periodic classes. This shows that although the genes of the periodic classes are annotated to a wide range of biological processes, many of these processes are cell cycle-related (see Additional file 5 for all functional signatures).

We also considered the periodic classes separately to see whether we could find terms specific to the different classes when compared to each other. For example, one would expect the genes in class 111 to be annotated to core processes of the cell cycle. Indeed, the term "regulation of cell cycle" (GO:0007088) was significantly overrepresented in class 111 at the 0.05 level. Although the functional signatures of the other periodic classes were rather specific, all periodic classes had some functions that were overrepresented only in that class. Even though the associated p-values were only significant at the 0.05 level, and were generally not sufficiently low to be significant when corrected for multiple hypotheses, we find it encouraging that unique functional signatures could be recovered for each of the periodic classes, even when the comparison was done only among the periodic classes. We interpret the existence of functional signatures as further evidence that the periodic classes inferred from expression data do not constitute a set of random selections of cell cycle genes.

## Discussion and conclusion

There has been an upsurge in publications attempting to uncover genome-wide transcriptional control structures using machine learning strategies. We believe approaches such as the one presented here have two-fold use: i) They allow researchers with interest in particular genes or regulators to find *in silico *support for their hypotheses. ii) They demonstrate genome-wide properties of the transcriptional network. We were able to find known interactions in the data, and we expect predicted interactions to be a valuable resource for experimentalists when designing experiments.

The basic idea in the present work is to identify genes whose responses over time due to treatment are similar. However, such similarity will naturally depend on the measure of similarity used. As demonstrated here, it is possible to focus on a particular process of the system, like the cell cycle in budding yeast, by choosing a measure of similarity which divides the genes into classes that are known to be related to the process of interest. The cell cycle has some special characteristics that makes defining such a measure easy. However, one may very well define such measures with natural semantics for other biological settings. For instance, in an infection study one may be interested in finding regulatory descriptors corresponding to different stages of the infection. By designing a detector that specifically identifies genes active at different stages we would expect more relevant classes to be found than by relying on clustering. Of course, designing such detectors can become quite cumbersome. An interesting direction of future research would be to use hidden Markov models for dividing the genes into different groups. Such models allow incorporation of prior knowledge about the dynamics of the process and has been successfully applied to gene expression data [25].

One notable exception to the use of clustering analysis is Tsai *et al*. [26] where transcription factors were defined as cell cycle-related if the genes they were regulating had a significantly different expression level in at least one of five phases of the cell cycle compared to one or more of the other phases. Although this is an interesting approach, we were fascinated by the large discrepancies between sets of genes detected as periodic in *S. cerevisiae *for different synchronization methods [20]. Our work lends support to the notion that periodic expression is conditioned on different stimuli. Using descriptors of possible *cis*-regulatory elements, we were able to find regulatory combinations specific to different sets of synchronization methods. It should be noted that the rationale for using different synchronizations in the experiments by Spellman *et al*. [2] was to be able to identify a signal that is assumed to be common across the experiments corresponding to the physiological pattern of expression in the cell cycle. In this study we instead acknowledged that different synchronization methods may correspond to different environments (internal and/or external) in which the cells propagate, and sought to explain why genes have different expression patterns with respect to periodicity under these conditions. The fact that it is possible to find combinations of transcription factors and sequence motifs that are significantly more common among genes within our classes supports this hypothesis. However, the expression data was only available for the two first periods of the *S. cerevisiae *cell cycle, thus we cannot claim that the differences in periodic behavior are long-lived. In fact, it is intuitively more appealing to regard these differences as temporary in the sense that the effects of different initializations of the cell cycle (i.e. synchronization methods) will die out in the long run.

The fact that we group genes according to periodicity in three different synchronization experiments, and not according to conventional expression similarity, means that we cannot expect all genes within a periodic class to be regulated by the same mechanism. Indeed, what we see is many different mechanisms describing different subsets of genes within each periodic class. In principle, we could have subdivided our periodic classes into cleaner regulatory modules based on, for example, the time of peak expression. However, with several of the periodic classes already containing very few genes, a more practical approach was to let the rule method arrive at this subdivision automatically based on the available *cis*-regulatory information and the periodic classes.

The periodic classes are inferred exclusively via computational analysis of expression data, and no biological experimental validation has been performed. The class division will depend on the specific thresholds of detection. The results presented here are based on a scheme known as "classification with rejection" where genes for which neither outcome is supported are rejected from further analysis. We also attempted a class division with a single sided criterion (using only criterion *B *as introduced earlier), classifying genes as periodic if *s *> 0.95 and non-periodic otherwise. Using this single criterion for classification we found the Gene Ontology term "regulation of cyclin-dependent protein kinase activity" (GO:0000079) to be overrepresented in class 011, the set of genes detected as periodically expressed only in the *cdc*-experiments. This was encouraging since the *cdc*-based synchronizations act by interfering with different cyclin dependent protein kinases [27]. However, as expected, such a criterion renders a class distribution that is skewed towards class 000. Thus, this reduces the chances of extracting rules describing the regulatory mechanisms of the (relatively) few representatives in the periodic classes.

We also attempted the use of only motifs or only transcription factors as descriptors. Results were similar; the class division based on detected periodicity was more specific towards cell cycle regulators than the class division based on clustering. However, class specific overrepresentation of *cis*-regulatory elements (i.e. motifs or transcription factors) was weaker, indicating an advantage of using the novel *cis*-regulatory descriptors based on both sequence motifs and actual transcription factor binding. A further improvement of these descriptors would be to include proximity and order of the sequence motifs in the promoter regions [28], however, such information was not utilized in this study.

The class-specific hierarchical structure of the discovered *cis*-regulatory descriptor combinations is an example of general system-wide properties discovered by our method. Genes in class 111 have the largest fraction of combinations where smaller subsets are associated with more restricted periodic expression. Note that due to the classification with rejection criterion discussed above, this hierarchy is not a trivial result of the class structure, e.g. genes in class 111 are neither a subset nor periodically similar to genes in class 110, in fact, genes in class 110 have a low probability for being periodic in the third experiment. Hence, the hierarchy suggests that the subsets of *cis*-regulatory descriptors are sufficient for periodic expression of the genes in fewer conditions. From an evolutionary standpoint it may be advantageous to ensure periodic expression of vital components in a wide variety of conditions by building in redundancy and to include many *cis*-regulatory elements. One alternative way to regulate periodic expression would have been to use only one phase-specific mechanism in genes that are always periodically expressed and to block periodic transcription in the appropriate conditions. However, this model has no support in the data and can be excluded.

## Methods

### Datasets

#### Expression data

We use the publicly cDNA and ratio-transformed oligonucleotide microarray data published by Spellman *et al*. [2,29]. In these experiments, yeast cell cultures were synchronized using various methods. Our detector was applicable to the three experiments for which period times were reported: *α*-factor synchronization (18 time points), *cdc15 *synchronization (24 time points) and *cdc28 *synchronization (17 time points). *α*-factor is a mating pheromone that blocks haploid *S. cerevisiae *in the G1 phase of the cell cycle. When the blocked cells are moved to an *α*-factor free media they proceed to divide synchronously. The *cdc *synchronizations use a temperature sensitive mutant yeast that will block at a specific phase when temperature is increased and proceed in synchrony as it is lowered. For more details see Wagner [27].

#### Binding site data

We used the 43 known binding sites as well as the 313 putative motifs recovered from sequence by AlignACE [22]. The putative motifs were found by applying AlignACE to the upstream regions of genes that constituted functional classes in the literature, i.e. classes having similar biochemical functions or participating in the same process. In total 5650 open reading frames (ORFs) are annotated in this manner by Hughes *et al*. [22].

#### Transcription factor binding data

The genome-wide location assays reported by Harbison *et al*. [16] were used to identify transcription factor binding. A total of 251 transcription factors were linked to the upstream regions of ORFs determined by microarrays. For each transcription factor and ORF, a p-value of binding was reported. We considered a transcription factor to bind upstream of a gene if the corresponding p-value of binding was below 0.05 (as recommended by Harbison *et al*. [16]).

#### Transcription factor – sequence motif filtering

For all pairs of transcription factor and sequence motif present in the data, the probability of obtaining as many or more occurrence of the pair by chance was calculated. E.g. for a given transcription factor that occurs *r *times among the *k *ORFs with a given sequence motif follows the hypergeometric distribution when the *k *genes with a particular sequence motif is considered a random selection. A p-value is calculated as the probability of *r *bindings or more. This is done for each transcription factor as well as each motif. The three best scoring partners (lowest p-values) below 0.05 were included for further analysis. Note that only significant partners were included, i.e., if a motif only had two partner transcriptions factors with *p *< 0.05, only those two were included.

### Significance of descriptor overrepresentation

The probability of finding *r *out of *k *ORFs with a specific transcription factor (TF)-motif pair (or a combination thereof) within a class by random sampling is calculated using the hypergeometric sampling distribution. I.e., the class is considered a random selection of genes. This yields the probability of observing the data under the null hypothesis *H*_0_: Presence of TF-motif pair and class membership are independent. When all pairs of *cis*-regulatory descriptors are considered, a large number of such p-values are obtained, one for each combination.

Since small p-values are bound to be found among many tests even if all hypotheses tested are true nulls, we need to test whether we have more small p-values than would be expected by chance. To see this, we perform a test as to whether the hypothesis *H*: "All hypotheses tested are true *H*_0_" can be rejected. Our reasoning is similar to the development of the Bonferroni correction for multiple hypotheses, that is, one assumes a null model in which all hypotheses tested are independent and true null. By definition the distribution of p-values under multiple independent true null hypotheses is uniform on [0, 1] i.e. *p *~ *U *(0, 1). In other words, the probability that a true null hypothesis yields a p-value smaller than some value *q *will be *q*. Thus, if *n *true null hypotheses are tested, the number of hypotheses *k *rejected at some level *α *will follow a binomial distribution. Consequently, for a given test level *α *we calculate the probability of observing *k *hypotheses or more with p <*α *if all *n *hypotheses were true null using the binomial probability distribution. These are the probabilities given in Table 5. A small p-value in this test suggests there are far too many p-values lower than *q *for all of them to be true nulls. We note that these p-values should be interpreted with care as they may be optimistic, i.e. there may be dependencies. In other words, the class overrepresentation of combinations is likely to be correlated for combinations sharing a *cis*-regulatory descriptor.

### Rule-based retrieval of regulatory mechanisms

We assume that all genes with an identical regulatory structure will exhibit identical expression. Members of the same partition in the partitioning of yeast genes shown in Figure 1 will then not have identical regulatory structure to any gene in any other partition. We thus seek to identify descriptors (TF-motif pairs) that are unique to each partition. However, this would be an ideal situation. In practice, the partitioning contains noise and ambiguities are bound to arise.

The rough set theory was developed to deal with ambiguities in data [30,31]. In this setting, a rough set *X *⊆ *U *is a subset of all genes *U *that cannot be uniquely discriminated from the other genes in *U *on the basis of their descriptors. One may, however, describe *X *in terms of all genes in *X *that certainly are distinguishable from other genes (in rough set terminology the lower approximation of *X*) and the set of genes that are either in *X *or indistinguishable from members of *X *(the upper approximation). Minimal combinations of TF-motif pairs that preserve the information in the data (the lower and upper approximation of the periodic classes) are called reducts. In this study, we computed reducts for each gene, and interpreted them as hypothetical regulatory mechanisms. Each reduct gives rise to an IF-THEN rule that discriminate that gene, and genes in the same perioidc class, from genes from other perioidc classes. However, in noisy data reducts tend to be too specific and will not generalize well. A solution to this problem is to employ a development in rough set theory called approximate reducts (*α*-reducts) [32]. In the present study these a-reducts are minimal combinations of TF-motif pairs that discern a gene from a user-defined fraction *α *of the genes from other periodic classes.

There are a number of efficient heuristics available for calculating reducts. In this study, reduct calculation was done using a genetic algorithm [33] implemented in the ROSETTA system [34,35] with the default setting of 0.95 for *α*. For more details, see Hvidsten *et al*. [7].

To filter out overly specific (only present in ~1 ORFs) or overly general (non-discriminatory) combinations, the degree of overrepresentation within the class was calculated for each TF-motif or each TF-motif combination. This was done by calculating the probability of obtaining as many or more ORFs having the combination by chance when the class is considered a random selection, i.e. the hypergeometric sampling probability. Note that if the right hand side of a rule includes more than one class, a p-value is still only computed for the periodic class from which the rule was induced. Typically, only this class is significant while the others are a result of inconsistencies and noise in the data.

### Detecting periodic expression

A recently proposed detector of periodic expression was used [19]. It is built on the Bayesian formalism and relies on prior knowledge about the period time and has been shown to be robust against different waveforms as well as errors in the estimation of the period length. In essence, the detector fits a times series to two different models: one time independent and one that is time dependent and periodic. The fits to the two different models are compared and the Bayesian formalism allows a probability of periodicity to be calculated, denoted *s*. Strictly speaking this probability is an approximation of the probability a fully analytical Bayesian treatment would yield. However, a fully analytical treatment is not possible and has to be approximated. See Andersson *et al*. [19] for further details. The probability *s *is used as a detector statistic and was calculated for each of the ORFs in the Spellman *et al*. [2] experiments using experiment-specific cell cyle period times. The genes were then divided into classes as described earlier. Unlike many other methods, our detector does not require training examples for estimating parameters. Moreover, our method is the sole one that manages to apply significant yet vaguely expressed information such as the period time and uses it in a potent and formal framework including a model of the dynamics. This offers a solid starting point for evaluating usefulness of prior information as examplified in the current paper.

## Competing interests 

The author(s) declares that there are no competing interests.

## Authors' contributions

CRA, AI and MGG contributed the Bayesian detector of periodic expression and software to this end. TRH and JK contributed the rule-based method of describing gene expression in terms of sequence motifs. JK provided the ROSETTA system. CRA and TRH conceived the idea of merging the methods and designed the approach presented in this paper. All authors helped improving the design, interpreting the results and writing the manuscript. CRA inferred periodic classes and did the statistical analysis. TRH induced rules and did the comparison with clustering. All authors read and approved the final manuscript.

## Supplementary Material

Additional file 1 All significant cis-regulatory descriptors (i.e. transcription factor – sequence motif pairs). For each transcription factor and each sequence motif, the three best (p < 0.05) sequence motifs/transcription factors are listed with p-values. Click here for file

Additional file 2 All rules. All rules associating combinations of cis-regulatory descriptors with periodic classes of expression. The p-value for the periodic class that the rule was induced for is given together with the parameters for the the hypergeometric distribution: (N,n,k,x), where N is the number of genes in the data set, n is the number of genes matching the rule, k is the number of genes in the periodic class and x is the number of genes matched by the rule and the periodic class. Click here for file

Additional file 3 All hierarchies of cis-regulatory descriptor combinations. All hierarchies of cis-regulatory descriptor combinations. Click here for file

Additional file 4 Names of the putative sequence motifs. See P# in Figure 5. Click here for file

Additional file 5 The functional signatures of each periodic class. Overrepresentation of function was calculated for a background consisting only of genes detected as periodically expressed in at least one of the experiments. Terms with a two-sided p-value below 0.05 in at least one class are shown. Click here for file
