# Supplementary material for: Revealing cell cycle control by combining model-based detection of periodic expression with novel cis-regulatory descriptors
Source: BMC Syst Biol. 2007 Oct 16;1:45. doi: 10.1186/1752-0509-1-45 (PMC2200664; doi:10.1186/1752-0509-1-45)
Supplement: Additional file 1 — All significant cis-regulatory descriptors (i.e. transcription factor – sequence motif pairs). For each transcription factor and each sequence motif, the three best (p < 0.05) sequence motifs/transcription factors are listed with p-values. [file 1752-0509-1-45-S1.pdf]

All significant *cis*-regulatory descriptors (i.e. transcription factor - sequence motif pairs). For each transcription factor and each sequence motif, the three best ($p < 0.05$) sequence motifs/transcription factors are listed with p-values.

PDR1
RAP1: 1.59008373555703e-33
m_RPE17: 7.68295449884346e-12
m_RPE6: 9.27548996633533e-12

MDS3
m_other_morphogenetic_activities_n7: 0.00024639072887464
m_organization_of_cell_wall_n6: 0.000600664009557197
m_utilization_of_vitamins_cofactors_and_prosthetic_groups_n6: 0.0087668378150708

UME1
m_meiosis_n3: 7.55461338431318e-28
Ume6(URS1): 1.77156914414105e-19
m_other_energy_generation_activities_n4: 0.000175102396589089

INO2
m_other_cation_transporters_n8: 2.33607925786658e-05
m_regulation_of_lipid_fatty: 2.43645032100293e-05
m_other_nutritional: 4.874536344343e-05

PDR3
m_other_morphogenetic_activities_n8: 0.0293151785239645
m_cytokinesis_n10: 0.0298960752400988
m_lipid_and_fatty: 0.046807989828118

ECM22
m_phosphate_transport_n18: 3.17145810646386e-05
m_MERE4: 3.30460941384763e-05
m_other_pheromone_response_activities_n8: 8.01933058137794e-05

INO4
m_phosphate_transport_n18: 3.10953249425969e-14
m_other_mrna: 3.68819553814208e-12
m_regulation_of_lipid_fatty: 3.76613352844683e-12

PIP2
m_other_mrna: 1.33125436782639e-06
m_metabolism_of_energy_reserves_n8: 2.99326969536294e-05
m_other_transport_facilitators_n10: 8.87027255691236e-05

HAA1
m_lipid_and_fatty: 0.0163292451139672
m_other_energy_generation_activities_n20: 0.0164023718384477
m_abc_transporters_n10: 0.0459515918789104

SMK1
m_biogenesis_of_cytoskeleton_n12: 0.000804010661341636
m_pheromone_response_generation_n7: 0.00162054527868754
m_fermentation_n18: 0.00456712900954078

SKO1
m_glyoxylate_cycle_n11: 3.30617858337978e-09
MIG1: 1.77815610482868e-07
m_purine_and_pyrimidine_transporters_n10: 1.62100444764741e-06

SOK2
m_other_nutritional: 0.00210850041901499
OAF1: 0.00259719735830953
m_cellular_import_n12: 0.00313450000635261

RFX1
OAF1: 0.0205939219061928

YML081W
HSE: 1.31562212670246e-05
m_regulation_of_amino: 0.000551057456765521
m_cytokinesis_n10: 0.00213312241980789

PPR1
ABF1: 0.0124675394008331
m_anion_transporters_n19: 0.0263015903360696
m_other_pheromone_response_activities_n14: 0.0295737032992536

GAL3
SFF: 0.00264053008762532
Ume6(URS1): 0.00744303318333125
m_meiosis_n3: 0.0331308586000423

BAS1
m_metabolism_of_energy_reserves_n30: 0.00022549370987968
m_RPE68: 0.00102222317832344
GCN4: 0.00445538143190949

IFH1
m_peroxisomal_transport_n15: 0.000494990400571306
m_pheromone_response_generation_n7: 0.00149140534552906
m_pheromone_response_generation_n10: 0.00244406975710661

YDR520C
m_organization_of_golgi_n7: 0.00673074155172024
m_RPE68: 0.0152487419990267
m_other_nutritional: 0.0212235115637455

WTM1
ndt80(MSE): 0.00395167794894418
m_deoxyribonucleotide_metabolism_n8: 0.0303697801773932

PDC2
m_peroxisomal_transport_n15: 0.00249085313475092
MCM1': 0.013812920893423
m_fermentation_n4: 0.0190068170722721

PUT3
m_phosphate_transport_n18: 1.77917333119914e-08
m_MERE4: 2.45006208787269e-06
m_proteolysis_n8: 7.7169155295361e-06

GAT1
m_glyoxylate_cycle_n11: 2.05495862338789e-15
m_regulation_of_lipid_fatty: 3.00063547271362e-15
m_lipid_and_fatty: 4.26617582714689e-15

AFT2
m_regulation_of_lipid_fatty: 0.000209248714545554
m_cellular_import_n12: 0.00544760884236616
m_phosphate_utilization_n4: 0.0382723046489345

GAT3
RAP1: 2.15371413882562e-40
m_RPE6: 5.78129238075292e-21
m_RPE72: 1.87998108904742e-16

MET28
m_peroxisomal_transport_n19: 0.00293820920953545
m_translational_control_n10: 0.00633903633995807
m_peroxisomal_transport_n15: 0.0133892874739332

RCO1
m_peroxisomal_organization_n6: 0.00105293767487864
ALPHA2: 0.00645684509069433
m_nitrogen_and_sulphur_utilization_n15: 0.0100218968206164

A1(MATA1)

PAC: 0.00333305698562118
m_biogenesis_of_cytoskeleton_n12: 0.00697003459284201
mRRPE: 0.00714001249988378

SPT10
m_pheromone_response_generation_n7: 9.83101645102062e-05
m_drug_transporters_n14: 0.0019771117895247
m_lysosomal_and_vacuolar_degradation_n8: 0.00337438790398326

LEU3
RAP1: 1.14631697185188e-06
Leu3: 1.45781653543413e-06
mRRPE: 0.000732918113942664

MTH1
m_MERE4: 3.72346367131908e-14
m_pentose: 1.02239026000171e-13
m_cell_death_n22: 1.27935280824473e-12

GZF3
m_nitrogen_and_sulphur_transport_n5: 0.000296939564718367
SFF: 0.000433115824938056
m_peroxisomal_transport_n15: 0.0149480919155595

STB2
m_organization_of_centrosome_n6: 0.000378262416761373
m_other_pheromone_response_activities_n5: 0.000397059998910323
m_allantoin_and_allantoate_transporters_n13: 0.00262054741486525

RPI1
m_lipid_transporters_n8: 0.00144829579162535
SFF: 0.00905869427291162
m_biogenesis_of_chromosome_structure_n18: 0.0194503635065962

STB4
MCB: 0.000106562176468792
m_fermentation_n18: 0.01498017462499
ALPHA1': 0.0184001501459273

STB6
m_utilization_of_vitamins_cofactors_and_prosthetic_groups_n5: 0.00166516501329859
m_peroxisomal_transport_n15: 0.00358296103672868
zap1: 0.00465283344125494

SIP3
m_other_signal: 0.00198596653781242
SFF: 0.00687499431603118
SFF': 0.0176740940654156

MSN1
m_RPE58: 4.47291829308483e-09
RAP1: 7.83859952794245e-08
m_RPE34: 2.62610195354675e-06

CRZ1
m_lipid_and_fatty: 2.41285787042819e-06
m_ion_transporters_n4: 3.78722405862196e-06
m_other_transport_facilitators_n10: 0.000104827918841026

SMP1
RAP1: 4.55299838513654e-21
m_RPE21: 2.42978078198128e-15
m_RPE17: 6.12602573766609e-11

CUP9
m_cellular_import_n12: 2.62517934874099e-05
m_metabolism_of_energy_reserves_n30: 0.000824717697494341
m_amino: 0.00162153052067963

YKR064W

m_metal_ion_transporters_n10: 0.00206314460804532
m_other_signal: 0.0088863813479696
m_pheromone_response_generation_n7: 0.00997980652169243

TYE7
PHO4: 7.57378180385721e-08
m_organization_of_cell_wall_n8: 0.000198346417108449
Gcr1: 0.00122231192432489

MET31
32: 1.86090057266741e-06
RAP1: 0.000890501056327511
m_peroxisomal_organization_n6: 0.00526977535380285

FKH1
SFF': 8.56508871819599e-19
SFF: 9.25518618197633e-10
m_pentose: 3.47877818067024e-08

ZMS1
m_other_nutritional: 0.00045018131052439
m_cytok9: 0.00366537808860935
m_other_energy_generation_activities_n4: 0.0161575405126003

PHD1
m_other_pheromone_response_activities_n12: 3.52768972977497e-07
m_organization_of_cell_wall_n8: 7.01181015391447e-07
m_amino: 9.14876078554752e-07

SUT2
m_biosynthesis_of_vitamins_cofactors_and_prosthetic_groups_n17.scn: 0.00330732919765503
Leu3: 0.00416984670259931
m_pheromone_response_generation_n7: 0.00530326613894897

MIG1
m_cellular_import_n12: 5.97118516922863e-05
SFF: 0.000270537881005311
m_regulation_of_nitrogen_and_sulphur_utilization_n10: 0.00070351685445916

USV1
m_regulation_of_lipid_fatty: 0.00122061108206236
m_regulation_of_lipid_fatty: 0.00591021796314031
m_other_cell_growth_cell_division_and_dna_synthesis_activities_n14.scn: 0.0101577508205803

MIG3
MIG1: 3.88435270498322e-08
m_cellular_import_n12: 5.17239419139785e-07
m_other_pheromone_response_activities_n12: 1.90138585884945e-05

YPR196W
RAP1: 6.92017760212335e-06
m_RPE8: 0.00201697605775886
m_cytokinesis_n10: 0.00413308839620726

RAP1
RAP1: 4.56096999608239e-127
m_RPE17: 1.95361527000086e-19
m_RPE6: 6.74204044536169e-17

HIR2
m_organization_of_chromosome_structure_n12: 0.00280018781160782
RAP1: 0.00588707947732573
m_amino: 0.00783332248907265

YBL054W
mRRPE: 0.0139295876198649
m_other_intracellular: 0.0146419281863605
ABF1: 0.0150521522704389

FZF1

m_other_morphogenetic_activities_n7: 0.00560339569255242
ALPHA2': 0.012612564168412
m_fermentation_n10: 0.0130325839872509

DAL80
m_purine_and_pyrimidine_transporters_n6: 0.00109287040557034
m_regulation_of_lipid_fatty: 0.0107897230359572
m_metal_ion_transporters_n25: 0.0131236769469468

YLR278C
m_other_proteolytic_degradation_n7: 0.00549544466675638
m_biogenesis_of_cytoskeleton_n12: 0.00690264818859406
m_other_signal: 0.00749244874242626

XBP1
m_phosphate_transport_n18: 8.47533425725024e-14
m_metabolism_of_energy_reserves_n8: 2.95961616480632e-10
m_other_mrna: 9.4258010917725e-09

DAL82
BAS1: 0.000148570456605617
m_peroxisomal_transport_n19: 0.00106842457143126
GAL: 0.0130101729484676

ZAP1
RAP1: 8.20285583301733e-06
m_RPE57: 0.000152473028155743
zap1: 0.000271332230875359

SPT23
m_other_cell_growth_cell_division_and_dna_synthesis_activities_n10.scn: 0.00161681406433061
m_other_proteolytic_degradation_n12: 0.00714163726599011
m_cytoskeleton: 0.0126245080821964

GAL80
m_lipid_transporters_n10: 0.0401401220649934

HAC1
m_metabolism_of_cyclic_and_unusual_nucleotides_n5: 0.000317388825165739
m_glycolysis_and_gluconeogenesis_n11: 0.000478214354423612
m_allantoin_and_allantoate_transporters_n17: 0.00839303944208345

RGT1
m_drug_transporters_n7: 0.000124380292935824
m_nutritional_response_pathway_n3: 0.00171397049044899
mPROTEOL18(m_proteolysis_n18): 0.00293762445888484

DIG1
STE12: 0.000544203895987083
m_pheromone_response_generation_n10: 0.00134210770607427
ECB: 0.00170708751498702

SUM1
ndt80(MSE): 3.23379876891414e-31
BAS1: 0.00131689656621134
m_abc_transporters_n2: 0.00461957336031101

MBF1
m_amino: 0.000188332904491273
m_anion_transporters_n4: 0.000950891865044874
m_anion_transporters_n20: 0.00180279677414641

OAF1
m_other_transport_facilitators_n10: 7.24493737254762e-09
m_regulation_of_lipid_fatty: 4.74011511057784e-08
m_other_mrna: 3.79436927503699e-07

SWI5
m_amino: 1.17487700934108e-08
m_lipid_and_fatty: 3.89224545096411e-07

m_intracellular_communication_n4: 1.75090609554628e-06

KRE33
GAL: 0.00246654549221527
m_other_cell_growth_cell_division_and_dna_synthesis_activities_n14.scn: 0.00348538084583143
m_organization_of_centrosome_n6: 0.00793464844972551

GCN4
GCN4: 4.67474443017011e-32
BAS1: 9.29303247024623e-06
ALPHA2': 0.000293967458117515

GCR1
Gcr1: 0.000336334966610521
m_pentose: 0.0013690150531067
m_phosphate_metabolism_n18: 0.00161116754354065

STE12
STE12: 1.3577344157494e-09
m_allantoin_and_allantoate_transporters_n7: 4.04326325866853e-06
ECB: 0.000210736464759453

HOG1
m_other_morphogenetic_activities_n7: 2.11185228451538e-06
ndt80(MSE): 2.55548092510454e-06
SFF: 0.000507237412515912

YDR266c
ndt80(MSE): 0.00497743545736885
m_other_cell_growth_cell_division_and_dna_synthesis_activities_n10.scn: 0.00687509148935883
MET31: 0.00894732072642802

BYE1
ABF1: 0.00126358110713362
m_other_signal: 0.0120552125364316
m_utilization_of_vitamins_cofactors_and_prosthetic_groups_n6: 0.0197760890478159

RME1
RAP1: 1.53381553579961e-06
m_RPE32: 1.90602257661971e-05
m_pentose: 9.63556181349295e-05

SFL1
m_other_pheromone_response_activities_n14: 0.000383826485746748
m_c: 0.00214584112572978
m_regulation_of_nitrogen_and_sulphur_utilization_n10: 0.00670596346076201

ARR1
m_other_morphogenetic_activities_n7: 6.85064764062746e-05
m_regulation_of_amino: 0.000274950908197551
SCB: 0.00718440032085879

ACA1
m_glycolysis_and_gluconeogenesis_n4: 0.00902037807206742
m_organization_of_centrosome_n6: 0.0121231706358066
m_biogenesis_of_cytoskeleton_n5: 0.0128273875417468

THI2
HAP234: 0.0242818577916333
STE12: 0.0283509789810395
m_metal_ion_transporters_n14: 0.0351028170672356

HMS1
RAP1: 5.2630942586162e-05
m_other_pheromone_response_activities_n14: 0.000884025666247098
m_RPE52: 0.00254813380139936

SRD1
OAF1: 0.00669215673971451
HSE: 0.0153429911263877

m_pyrimidine: 0.0197028993222964

RDS1
m_glycolysis_and_gluconeogenesis_n11: 0.000338288534039095
m_allantoin_and_allantoate_transporters_n7: 0.0218911372187221
m_pheromone_response_generation_n7: 0.0235571366887013

YAP1
Yap1: 4.39829259434032e-13
m_ionic_homeostasis_n6: 1.04862044059436e-08
m_RPE58: 2.31656087849301e-06

ACE2
m_cell_death_n16: 8.6896503924113e-09
m_MERE4: 8.86272843912802e-09
m_regulation_of_amino: 2.76286447573246e-08

YAP3
m_lysosomal_and_vacuolar_degradation_n3: 0.000940441706731405
m_other_energy_generation_activities_n17: 0.00095605105931018
BAS1: 0.0065344858924998

MAL13
m_MERE17: 1.17490845381093e-07
m_cell_death_n22: 1.73271686254651e-06
m_RPE49: 7.51923373437155e-06

YAP5
RAP1: 1.79166235258345e-55
m_RPE72: 9.98530498643493e-18
m_RPE6: 1.24680502512434e-15

CHA4
m_other_pheromone_response_activities_n14: 0.0451753389827518

YAP7
ABF1: 0.0145120460605745
m_nitrogen_and_sulphur_utilization_n15: 0.0174657103050161
m_other_energy_generation_activities_n20: 0.0313208123618439

ARG80
m_other_pheromone_response_activities_n14: 0.000826351218771766
SFF: 0.0269931839228931
m_other_signal: 0.042689715802913

SKN7
m_metabolism_of_energy_reserves_n8: 2.64382723174593e-15
m_other_mrna: 4.51597429896278e-15
m_amino: 1.7049440044179e-14

YER130C
ndt80(MSE): 0.00142249032213504
m_utilization_of_vitamins_cofactors_and_prosthetic_groups_n6: 0.0017150575881003
m_glycolysis_and_gluconeogenesis_n11: 0.00608171546808338

NDD1
MCM1: 1.52540772414436e-17
ECB: 1.56287331678198e-15
m_MERE4: 1.60335562945271e-09

STP1
m_other_morphogenetic_activities_n7: 0.000512103026220431
m_regulation_of_lipid_fatty: 0.00148992238553386
m_trna_processing_n6: 0.00245514892973458

SPT2
m_RPE52: 0.000442689595284812
mRRPE: 0.00110457087543934
m_amino: 0.00251354932858916

YJL206C
m_anion_transporters_n23: 0.00275697003458878
m_c: 0.00392031331224039
m_other_cell_growth_cell_division_and_dna_synthesis_activities_n14.scn: 0.00498748628658396

REB1
REB1: 6.8478832574409e-88
RPN4: 1.11515357877553e-06
m_other_signal: 0.000581777689235879

DAT1
m_biogenesis_of_cytoskeleton_n12: 0.0133425292818014
RAP1: 0.0191859027993363
ALPHA1: 0.0320127581099348

ARO80
RAP1: 0.000284650703844548
m_trna_processing_n6: 0.000899673659586509
m_regulation_of_amino: 0.00147552314440795

HAP1
m_metal_ion_transporters_n10: 4.70601792094747e-07
HAP234: 1.45890307965126e-06
m_homeostasis_of_metal_ions_n20: 1.53522602752177e-06

FHL1
RAP1: 2.70879873268246e-90
m_RPE21: 1.277388343114e-24
m_RPE6: 1.54533031457453e-20

HAP3
m_utilization_of_vitamins_cofactors_and_prosthetic_groups_n5: 0.00640713897726557
m_other_proteolytic_degradation_n11: 0.00715204028065214
m_lysosomal_and_vacuolar_degradation_n8: 0.00955199164571638

IME1
m_pheromone_response_generation_n12: 0.00168876970606473
m_utilization_of_vitamins_cofactors_and_prosthetic_groups_n5: 0.00685811253283861
m_nitrogen_and_sulphur_utilization_n4: 0.0120652757408725

YRR1
m_fermentation_n10: 0.00159541376379105
m_utilization_of_vitamins_cofactors_and_prosthetic_groups_n7: 0.00351091418003995
m_lipid_and_fatty: 0.00990320256484348

MCM1
ECB: 2.01670137062298e-30
MCM1: 2.81166173140235e-25
MCM1': 2.32780850078996e-09

HAP5
ALPHA2: 0.0213693581491901
m_lipid_and_fatty: 0.0217840912127928
m_amino: 0.0372895495282773

MET18
m_organization_of_cell_wall_n6: 0.00828934625148736
m_metal_ion_transporters_n25: 0.0359225468127212
MET31: 0.0446924560199119

YPR022C
m_metal_ion_transporters_n10: 0.0102413281410414
m_other_nucleotide: 0.0119185632662026
m_utilization_of_vitamins_cofactors_and_prosthetic_groups_n7: 0.02429470794694

YGR067C
m_phosphate_transport_n8: 0.00375566139374936
m_translational_control_n10: 0.00544157669609023
m_other_nucleotide: 0.00628269465507774

SIG1
ABF1: 0.00107617565267853
m_other_nucleotide: 0.00432893969340488
m_pheromone_response_generation_n7: 0.00658366947933661

MSS11
OAF1: 0.00790417766507024
m_other_cell_rescue_activities_n10: 0.00914812009950213
m_nitrogen_and_sulphur_transport_n5: 0.0136832776080312

CAD1
Yap1: 1.1611247314217e-22
m_c: 7.1751083205478e-05
ECB: 0.00368997371849706

OPI1
m_trna_processing_n6: 0.00418514169053383
m_peroxisomal_transport_n15: 0.00737612014698252
m_drug_transporters_n14: 0.0114672063093284

UPC2
ndt80(MSE): 0.000186919927558738
ALPHA1': 0.000653505217573012
m_PNDE6: 0.00227348842118597

RIM101
ALPHA1': 9.56776778603689e-06
SWI5: 0.000480677929056736
m_fermentation_n12: 0.000527784028830139

RPH1
m_MERE4: 0.000144323663971089
m_RPE57: 0.000398872219696742
m_other_cation_transporters_n13: 0.0006644977064523

CBF1
PHO4: 2.17564341142812e-31
m_regulation_of_amino: 3.22214010505226e-07
m_deoxyribonucleotide_metabolism_n23: 8.46320951010662e-07

UME6
m_meiosis_n3: 5.69823248189224e-114
Ume6(URS1): 1.74929739951136e-70
m_glyoxylate_cycle_n11: 1.74253734970203e-17

DOT6
m_regulation_of_lipid_fatty: 1.05778759503665e-11
m_abc_transporters_n5: 6.31386567995493e-07
m_metabolism_of_energy_reserves_n8: 1.80731564721087e-06

KSS1
m_other_cation_transporters_n14: 0.000856805965595611
ALPHA1': 0.00096692493617715
SWI5: 0.00138683334276019

ABT1
m_lipid_transporters_n8: 0.00197340266819541
m_organization_of_cell_wall_n10: 0.00884325214283461
m_other_cell_rescue_activities_n10: 0.0192746592476899

GAL4
GAL: 3.82109065386869e-06
m_regulation_of_amino: 0.00862243539309164
m_peroxisomal_transport_n15: 0.0121546716708866

YKL222C
m_other_nucleotide: 0.000926281783576027
m_tricarboxylic: 0.00210239337986837
m_organization_of_cell_wall_n14: 0.00812825512564043

YDR026c
Ume6(URS1): 1.02136434174773e-06
m_meiosis_n3: 0.0001619832042947
REB1: 0.00100080670667541

WTM2
m_lysosomal_and_vacuolar_degradation_n8: 0.00702918983445336
m_metal_ion_transporters_n25: 0.0120613284964738
m_fermentation_n14: 0.021107992414958

MBP1
MCB: 1.41939314989144e-45
mPROTEOL18(m_proteolysis_n18): 7.00294570714152e-10
m_cell_death_n16: 1.19330066662039e-07

GLN3
m_regulation_of_amino: 0.00161744616816796
m_metabolism_of_energy_reserves_n30: 0.00251174539904629
m_fermentation_n18: 0.00326227112790305

SNF1
m_other_morphogenetic_activities_n8: 0.0240476550314019
ABF1: 0.0288859980141894
m_nitrogen_and_sulphur_transport_n14: 0.0348593845820286

YFL052w
m_other_signal: 0.000833278246907098
m_metal_ion_transporters_n10: 0.00486345292484809
MCB: 0.00891363396090709

PHO2
m_organization_of_cell_wall_n14: 7.17078367003117e-05
m_lipid_transporters_n8: 8.39884392095007e-05
RAP1: 0.000157060287737239

RCS1
m_glycolysis_and_gluconeogenesis_n11: 0.00215638091075421
mRRPE: 0.00451253172237324
m_other_morphogenetic_activities_n7: 0.00553756225338228

TBS1
m_regulation_of_amino: 0.000254332699069663
m_trna_processing_n6: 0.00277968219922932
m_amino: 0.00331347506563804

STB1
m_glycolysis_and_gluconeogenesis_n27: 0.000296370742261263
MCB: 0.000585447007359913
m_cytoskeleton: 0.000707534689578075

PHO4
m_metabolism_of_energy_reserves_n27: 0.0010944192548113
m_other_energy_generation_activities_n22: 0.00123163933072617
m_cell_rescue_defense_cell_death_and_ageing_n20: 0.00310130001163144

RLM1
m_utilization_of_vitamins_cofactors_and_prosthetic_groups_n7: 8.85219078840359e-06
mRRPE: 0.000154887279141361
SFF': 0.000195098788750508

STB5
OAF1: 0.00093586982803691
m_cytokinesis_n10: 0.00353214403868293
m_assembly_of_protein_complexes_n23: 0.00782418478026869

SIP4
m_other_energy_generation_activities_n4: 0.00568974611617122
m_biogenesis_of_chromosome_structure_n18: 0.015980019736598
MCB: 0.0175260286682079

CST6
m_lipid_and_fatty: 0.00370603838082381
m_organization_of_cell_wall_n6: 0.008628068457543
m_glycolysis_and_gluconeogenesis_n11: 0.0244895687262415

MAL33
m_other_pheromone_response_activities_n12: 2.30087310410478e-07
m_regulation_of_amino: 2.78944457043044e-06
m_amino: 3.88589581392493e-06

MSN2
m_trna_processing_n6: 5.96132628912483e-06
ALPHA2: 0.000112444381352308
m_fermentation_n21: 0.000483245333902344

YHP1
ndt80(MSE): 0.0136982144221139
m_biogenesis_of_chromosome_structure_n18: 0.0185893266434279
m_peroxisomal_transport_n15: 0.0192431698719759

MAC1
m_peroxisomal_organization_n8: 0.000108691823014731
m_other_pheromone_response_activities_n8: 0.00022269595509496
m_transport_facilitation_n29: 0.000233700164746019

MSN4
m_RPE8: 1.23427323239305e-08
m_lipid_and_fatty: 6.09068591611816e-07
RAP1: 2.83627868265284e-06

YBR267W
m_phosphate_utilization_n7: 0.00609116340829892
m_fermentation_n3: 0.0240678521354218
m_utilization_of_vitamins_cofactors_and_prosthetic_groups_n5: 0.0242442745742765

FKH2
SFF': 2.41642333301566e-12
MCM1: 3.70701824504807e-11
SFF: 1.13795624744081e-10

MET32
MET31: 1.75348468775393e-11
m_organization_of_cell_wall_n6: 1.95792618305618e-05
m_metabolism_of_energy_reserves_n8: 7.37780002378989e-05

SUT1
MIG1: 5.72336714455647e-08
CSRE: 5.98320976163486e-08
m_RPE17: 3.01197185354442e-07

YBR239c
ALPHA1': 0.00392379907661829
m_biogenesis_of_chromosome_structure_n9: 0.00656780645163271
m_pyrimidine: 0.00763692331807289

FAP7
m_biosynthesis_of_vitamins_cofactors_and_prosthetic_groups_n17.scn: 0.000570742529259672
m_PNDE6: 0.000588923709317696
PHO: 0.00224154648169658

MIG2
MCB: 0.00139538860125932
m_amino: 0.020149585524007
ALPHA2': 0.0207730585581109

YER184C
m_other_nucleotide: 0.00200344536128041
m_peroxisomal_organization_n3: 0.0151113475995874
SFF: 0.0314920768635816

HSF1
HSE: 2.51319232751667e-05
m_utilization_of_vitamins_cofactors_and_prosthetic_groups_n7: 0.00256286145501766
m_chromatin_modification_n21: 0.00315916174396563

HIR1
SFF: 1.92563339762432e-05
SFF': 3.19065120448311e-05
m_other_energy_generation_activities_n22: 4.60594214241345e-05

NNF2
m_other_signal: 0.00176248853641293
m_other_nucleotide: 0.0180540786926181
m_organization_of_cell_wall_n6: 0.0238555739862382

HIR3
m_other_proteolytic_degradation_n12: 0.00304600595285983
SFF': 0.00310905144057762
m_phosphate_utilization_n9: 0.00394306482897439

RDR1
m_lipid_and_fatty: 0.0053127028817271
m_meiosis_n3: 0.0195792033328642
m_utilization_of_vitamins_cofactors_and_prosthetic_groups_n7: 0.0251370952304692

DAL81
m_utilization_of_vitamins_cofactors_and_prosthetic_groups_n7: 0.00559885489064695
m_amino: 0.012291693146224
m_amino: 0.0137871969725225

ABF1
ABF1: 6.51081155498384e-214
Ume6(URS1): 2.98446262658455e-09
RPN4: 7.80264827001799e-09

GTS1
m_allantoin_and_allantoate_transporters_n7: 0.000227411015185995
m_nutritional_response_pathway_n12: 0.000241480351982433
m_glycolysis_and_gluconeogenesis_n4: 0.00126476658655151

RLR1
m_g: 0.00574840061158142
PAC: 0.0302847485799752
m_other_cell_growth_cell_division_and_dna_synthesis_activities_n10.scn: 0.0358768130372278

RPN4
m_lipid_and_fatty: 0.000530183720942343
m_utilization_of_vitamins_cofactors_and_prosthetic_groups_n7: 0.0012757069357361
RPN4: 0.00309620972225149

SWI4
m_ion_transporters_n11: 2.95293614906722e-15
MCM1: 1.09150589848423e-11
m_cell_death_n22: 4.74634844779453e-10

ADR1
m_glycolysis_and_gluconeogenesis_n11: 0.000208699454517613
m_other_cation_transporters_n7: 0.000305625358380776
m_regulation_of_lipid_fatty: 0.000777394722287909

SWI6
MCB: 1.01476000449615e-28
m_amino: 4.61934253333022e-13
m_nucleotide_metabolism_n6: 8.77175850554833e-11

ROX1
RAP1: 3.77254436580935e-08
MIG1: 1.90528611084431e-07
m_lipid_and_fatty: 2.97635525320657e-06

TEC1
STE12: 8.55271152870755e-07
m_other_energy_generation_activities_n17: 0.000449768986169191
ECB: 0.00124466072614795

ASH1
m_glyoxylate_cycle_n7: 2.53244379984374e-09
m_phosphate_transport_n18: 3.84899286441178e-09
m_amino: 3.72509955332574e-07

GCR2
m_other_morphogenetic_activities_n7: 0.00168092604601031
m_abc_transporters_n10: 0.00366642241355184
m_cytoskeleton: 0.00713877762134701

CIN5
MIG1: 3.58805166871143e-07
m_other_transport_facilitators_n5: 6.15192593974134e-07
m_glyoxylate_cycle_n7: 4.65245466699917e-06

AZF1
SCB: 3.67342942613943e-08
m_pentose: 4.00421865889727e-08
m_amino: 0.000166425643719409

RGM1
RAP1: 2.68228459609188e-32
m_RPE6: 3.58143873793957e-22
m_RPE72: 1.23510467852229e-21

NRG1
m_regulation_of_amino: 1.0949881098447e-06
m_other_proteolytic_degradation_n5: 1.40043857094918e-05
m_cellular_import_n12: 6.49723939265604e-05

HMS2
m_organization_of_centrosome_n6: 0.010017201423994
m_lipid_and_fatty: 0.014126045224101
m_detoxificaton_n34: 0.0169356874120324

MET4
MET31: 1.66468236249962e-08
m_metabolism_of_energy_reserves_n8: 1.35560556420668e-06
m_drug_transporters_n10: 1.71180231932498e-06

SFP1
RAP1: 1.07452316731019e-16
m_RPE21: 3.12525964628172e-11
m_RPE17: 1.11480057432684e-10

WAR1
m_utilization_of_vitamins_cofactors_and_prosthetic_groups_n5: 0.0104301475789486
ABF1: 0.01105573709405
m_other_nucleotide: 0.012539572994831

YER051w
ALPHA1': 2.79631082919245e-06
m_utilization_of_vitamins_cofactors_and_prosthetic_groups_n6: 0.00165738713690428
ndt80(MSE): 0.00318138425213559

RTG1
Yap1: 1.85523073248386e-05
m_other_morphogenetic_activities_n7: 0.00226139562397103
m_cell_death_n15: 0.00331980257689467

YAP6
m_phosphate_transport_n18: 4.19355283141714e-06
mPROTEOL18(m_proteolysis_n18): 6.57028810203606e-06
MIG1: 1.52822391041789e-05

ASK10
m_fermentation_n5: 0.00582133544272515
m_other_proteolytic_degradation_n12: 0.00943705260124607
m_other_cell_growth_cell_division_and_dna_synthesis_activities_n10.scn: 0.0341204626785286

RTG3
SFF: 3.2196892133864e-05
SFF': 0.000680600922221215
m_morphogenesis_n5: 0.000753211718236402

YDR049W
ndt80(MSE): 0.0017034768258218
m_biogenesis_of_chromosome_structure_n18: 0.0210018703750317
m_utilization_of_vitamins_cofactors_and_prosthetic_groups_n6: 0.0262498189436682

ARG81
RAP1: 0.0019686258626115
m_nitrogen_and_sulphur_metabolism_n16: 0.00724848106091822
m_lipid_and_fatty: 0.0130728011965845

MOT3
m_cell_death_n15: 0.0149805623939721
m_other_intracellular: 0.0241740112779084
m_homeostasis_of_metal_ions_n20: 0.0274568390082371

IXR1
m_organization_of_cytoplasm_n72: 0.00194546686967112
mRRPE: 0.00333203683177088
STE12: 0.0132825440942315

YFL044C
m_RRSE3: 0.020888974394842
m_peroxisomal_transport_n15: 0.0322758696615498
m_organization_of_cell_wall_n6: 0.0430667089879547

MGA1
m_organization_of_cell_wall_n20: 0.00717774293838217
m_deoxyribonucleotide_metabolism_n8: 0.0271905182065753
m_other_cell_growth_cell_division_and_dna_synthesis_activities_n10.scn: 0.0278341885469536

SNT2
m_amino: 0.000684413902397364
STRE: 0.00142705010572523
m_MERE17: 0.00240069626025106

YNR063W
m_metal_ion_transporters_n10: 0.000673291372575276
m_other_signal: 0.00407097857517364
MCB: 0.0275396233997314

STP2
m_utilization_of_vitamins_cofactors_and_prosthetic_groups_n7: 0.0117158917660071
m_lipid_and_fatty: 0.0144990106648336

STP4
m_other_cation_transporters_n13: 0.00135127091746098
m_sugar_and_carbohydrate_transporters_n14: 0.00149231012072824
m_homeostasis_of_metal_ions_n17: 0.00183956258808693

HAP2
HAP234: 4.24594313736567e-06
m_allantoin_and_allantoate_transporters_n18: 0.00110909533623326
m_RPE32: 0.00213758028100859

RTS2
m_lipid_and_fatty: 2.1357813873674e-05
ABF1: 0.000467059699662903
m_utilization_of_vitamins_cofactors_and_prosthetic_groups_n7: 0.0134236907286995

HAP4

HAP234: 2.07113217328185e-29
RAP1: 0.000120427349864923
m_anion_transporters_n22: 0.000335001224590172

EDS1
m_metabolism_of_energy_reserves_n30: 0.000828701184368985
m_other_morphogenetic_activities_n8: 0.0019807085586331
m_utilization_of_vitamins_cofactors_and_prosthetic_groups_n6: 0.0079854133965862

TOS8
m_phosphate_transport_n18: 1.0429666518458e-12
m_metabolism_of_energy_reserves_n8: 3.26880571649515e-09
m_cell_death_n22: 4.1845624624256e-09

HAL9
Yap1: 0.00086473236784928
m_other_pheromone_response_activities_n14: 0.0055301105816756
m_g: 0.017041866666766

NDT80
m_translational_control_n10: 0.0055721654677111

IME4
m_regulation_of_amino: 2.4766955125997e-07
PHO: 0.000243317909290488
m_g: 0.00072933778158975 1

UGA3
m_amino: 0.0129478324646446
m_other_intracellular: 0.0173986416134105
m_metal_ion_transporters_n17: 0.0182955865562336

YOX1
MCM1: 2.25276499470062e-06
ECB: 3.83412312249637e-05
m_organization_of_cell_wall_n6: 0.00344473582600707

m_intracellular_communication_n10
SKN7: 5.00157597624844e-07
MTH1: 2.55870340379806e-05
INO4: 0.000110791464256851

m_RPE49
FHL1: 5.7466852705339e-13
RGM1: 1.48324236551788e-11
GAT3: 1.17079940040057e-10

m_cell_death_n22
RGM1: 6.87908257980417e-18
SKN7: 1.43475643558959e-13
MTH1: 1.27935280824473e-12

m_osmosensing_n6
ROX1: 4.72269953770934e-05
SWI4: 7.57292055005029e-05
INO4: 0.00037175915691825

m_transport_facilitation_n29
MAC1: 0.000233700164746019
GAT1: 0.000309898997156145
HAP4: 0.000419290300391912

m_anion_transporters_n32
INO4: 0.000436542041895266
INO2: 0.000475815624118691
NDD1: 0.00279956901657591

m_organization_of_plasma_membrane_n15
INO4: 1.89020320053668e-05
UME6: 0.000287973744855958

MTH1: 0.000340871528166595

m_aminoacid_transport_n20
m_amino: 2.50852239466818e-10
m_amino: 6.81994371121035e-08
m_amino: 7.86969149687415e-07

m_organization_of_plasma_membrane_n17
INO4: 1.87351164988364e-08
OAF1: 1.21124023957946e-06
SFP1: 4.79841222538362e-05

m_regulation_of_aminoacid_metabolism_n7
m_regulation_of_amino: 0.000208830235376889
m_regulation_of_amino: 0.000417844619433547
m_regulation_of_amino: 0.0014543415111215

m_peroxisomal_organization_n3
UME6: 0.000138593556120508
XBP1: 0.00102937300842079
TOS8: 0.00276914636404199

RAP1
RAP1: 4.56096999608239e-127
FHL1: 2.70879873268246e-90
YAP5: 1.79166235258345e-55

m_peroxisomal_organization_n6
FKH1: 0.00040856879221855
INO4: 0.000488169199422591
MAC1: 0.00103545706295345

m_peroxisomal_organization_n8
SWI4: 1.52852779127422e-07
SWI6: 1.3992569196305e-05
YAP1: 5.67132944972684e-05

m_glycolysis_and_gluconeogenesis_n4
SFP1: 2.06491485117263e-06
INO4: 8.34605742047903e-05
PDR1: 0.00039246133382031

m_RPE52
FHL1: 2.58347793962986e-06
SPT2: 0.000442689595284812
SFP1: 0.000570173261288039

m_biogenesis_of_cytoskeleton_n12
SMK1: 0.000804010661341636
NRG1: 0.00187814191498378
SFP1: 0.00285237995934799

m_allantoin_and_allantoate_transporters_n6
MAL13: 0.00322263883484239
MET4: 0.00415077436100735
PHO4: 0.0146620450573896

m_allantoin_and_allantoate_transporters_n7
STE12: 4.04326325866853e-06
SFP1: 6.59170147581283e-05
ACE2: 7.46868514269936e-05

m_RPE57
RGM1: 4.97720731690161e-14
SKN7: 2.08105537360726e-13
FHL1: 1.23789752681852e-12

m_RPE58
RGM1: 7.1889941299189e-15
RAP1: 1.0860325266673e-12

YAP5: 8.04530466324867e-12

m_other_signaltransduction_activities_n8
m_other_signal: 3.07007608699646e-05
m_other_signal: 0.000150415174056079
m_other_signal: 0.000353562089656242

m_PNDE6
SFP1: 8.70231141179584e-05
MAL13: 0.000333063240293873
FAP7: 0.000588923709317696

m_other_pheromone_response_activities_n12
MAL33: 2.30087310410478e-07
PHD1: 3.52768972977497e-07
MIG3: 1.90138585884945e-05

m_other_transport_facilitators_n10
GAT1: 6.27666384665585e-10
SKN7: 7.89948347385997e-10
SWI6: 9.7373189830025e-10

GCN4
GCN4: 4.67474443017011e-32
CBF1: 0.000582758144815477
CRZ1: 0.00058299480205747

m_other_pheromone_response_activities_n14
FHL1: 0.000205455544183559
SFL1: 0.000383826485746748
RME1: 0.000757542324150747

m_other_transport_facilitators_n15
TOS8: 1.6850997900391e-05
GAT1: 0.00021186390727829
SWI5: 0.000250679416293185

m_regulation_of_lipid_fattyacid_and_isoprenoid_biosynthesis_n22.scn
m_regulation_of_lipid_fatty: 0.000209248714545554
m_regulation_of_lipid_fatty: 0.00354856645965168
m_regulation_of_lipid_fatty: 0.00591021796314031

MCM1'
MCM1: 2.32780850078996e-09
SWI4: 7.0044174987957e-09
NDD1: 3.71947060836773e-06

m_lipid_and_fattyacid_transport_n7
m_lipid_and_fatty: 2.02461229927302e-06
m_lipid_and_fatty: 4.57815659255237e-06
m_lipid_and_fatty: 5.69113172275264e-05

m_drug_transporters_n7
SKN7: 7.29242723115066e-07
MET4: 5.16825102560616e-06
MTH1: 5.52858692788725e-06

m_drug_transporters_n9
SWI4: 2.84960091634415e-06
GAT1: 4.40403369474677e-06
CRZ1: 0.000113270409512814

m_RPE68
FHL1: 4.30518086385651e-06
RAP1: 7.34351473914545e-06
PDR1: 7.3701512763965e-06

m_lipid_transporters_n10
MAL33: 0.00157467432515771
SUT1: 0.00236809561369576

INO4: 0.00391029316499915

m_RPE69
RGM1: 1.77235104543753e-18
FHL1: 5.09578628576883e-14
YAP5: 1.06834472921292e-13

m_ccompound_carbohydrate_transport_n11
m_c: 7.1751083205478e-05
m_c: 0.00242383905188339
m_c: 0.0113168322282691

m_deoxyribonucleotide_metabolism_n10
SKN7: 1.68012216652457e-05
INO4: 1.95235976721113e-05
SWI6: 4.43996833245031e-05

m_deoxyribonucleotide_metabolism_n12
TOS8: 0.000246194351021317
ECM22: 0.00029348820544485
MAL13: 0.00102672463067033

m_metabolism_of_energy_reserves_n8
SKN7: 2.64382723174593e-15
XBP1: 2.95961616480632e-10
RGM1: 1.7572889504457e-09

m_nucleotide_transport_n9
INO4: 1.10119701349629e-07
MCM1: 1.17252677241876e-05
SWI6: 0.00108113318811021

m_ccompound_carbohydrate_transport_n18
m_c: 0.00010676348921857
m_c: 0.00121125928101839
m_c: 0.00121125928101839

MCM1
MCM1: 2.81166173140235e-25
NDD1: 1.52540772414436e-17
SWI4: 1.09150589848423e-11

MET3132
MET31: 1.75348468775393e-11
MET31: 1.66468236249962e-08
MET31: 1.86090057266741e-06

m_regulation_of_nucleotide_metabolism_n5
SWI4: 2.69003506882438e-08
RGM1: 2.90633026590576e-06
UME6: 3.48778282684367e-06

m_other_nutritionalresponse_activities_n10
m_other_nutritional: 1.54188567811236e-05
m_other_nutritional: 4.874536344343e-05
m_other_nutritional: 5.37770117827146e-05

m_other_nutritionalresponse_activities_n11
m_other_nutritional: 0.000125525183665287
m_other_nutritional: 0.000454564853356253
m_other_nutritional: 0.00210850041901499

ALPHA1
SFP1: 2.587713640042e-05
GAT3: 3.83607405749364e-05
RGM1: 0.000195775407083509

ALPHA2
MSN2: 0.000112444381352308
INO2: 0.000719842130631664

NRG1: 0.00226317762589266

m_other_morphogenetic_activities_n7
HOG1: 2.11185228451538e-06
MCM1: 5.42515411469052e-05
ARR1: 6.85064764062746e-05

m_other_morphogenetic_activities_n8
EDS1: 0.0019807085586331
SFP1: 0.0025956291409796
ADR1: 0.00316724601882163

m_other_proteolytic_degradation_n11
HAP4: 0.00149778768965418
SUM1: 0.00553394647623887
HAP3: 0.00715204028065214

m_other_proteolytic_degradation_n12
STE12: 0.00267854887214156
HIR3: 0.00304600595285983
DIG1: 0.00344869641207673

m_OCSE15
FHL1: 2.20224436476705e-07
MTH1: 2.80401050995719e-06
RGM1: 3.58721366727407e-06

m_RPE72
RGM1: 1.23510467852229e-21
FHL1: 7.92867751894825e-19
YAP5: 9.98530498643493e-18

m_LFTE17
GAT1: 2.22542370041247e-14
UME6: 1.11055812133179e-09
SKN7: 4.12331013734776e-09

SFF
FKH2: 1.13795624744081e-10
FKH1: 9.25518618197633e-10
HIR1: 1.92563339762432e-05

m_mitochondrial_biogenesis_n5
MAL13: 7.48469360429833e-05
INO4: 0.000105617934755109
GAT1: 0.000249918623689174

ndt80(MSE)
SUM1: 3.23379876891414e-31
HOG1: 2.55548092510454e-06
UPC2: 0.000186919927558738

m_regulation_of_lipid_fattyacid_and_isoprenoid_biosynthesis_n5.scn
m_regulation_of_lipid_fatty: 2.38779883424967e-05
m_regulation_of_lipid_fatty: 2.53216660094874e-05
m_regulation_of_lipid_fatty: 0.000233793346217235

m_nucleotide_metabolism_n6
SWI6: 8.77175850554833e-11
UME6: 1.48734262850552e-09
MTH1: 2.68845697186735e-08

SFF'
FKH1: 8.56508871819599e-19
FKH2: 2.41642333301566e-12
MCM1: 4.59057595388865e-06

m_metal_ion_transporters_n10
SWI6: 1.4020246724787e-07
SKN7: 3.17206992765586e-07

HAP1: 4.70601792094747e-07

BAS1
GCN4: 9.29303247024623e-06
DAL82: 0.000148570456605617
INO2: 0.000710529371225567

m_ccompound_carbohydrate_transport_n23
m_c: 0.000833678580518003
m_c: 0.00702839604135072
m_c: 0.00986069616888374

m_peroxisomal_organization_n28
INO4: 4.87973651871207e-07
SKN7: 3.33164178019728e-05
UME6: 3.67003323980845e-05

m_deoxyribonucleotide_metabolism_n23
CBF1: 8.46320951010662e-07
MTH1: 1.88747120085571e-05
SWI4: 8.18898389583554e-05

ECB
MCM1: 2.01670137062298e-30
NDD1: 1.56287331678198e-15
FKH2: 4.48562991926193e-09

m_metal_ion_transporters_n14
GAT1: 3.17175212431583e-05
KSS1: 0.00168987746013121
INO4: 0.00183992207016597

m_homeostasis_of_metal_ions_n17
SKN7: 2.43844159114714e-05
MSN4: 0.000638561966478982
STP4: 0.00183956258808693

m_deoxyribonucleotide_metabolism_n27
SWI6: 4.68756383054597e-05
SWI4: 0.000710325998646567
MTH1: 0.000755572798416616

m_metal_ion_transporters_n17
MSN4: 2.41059150218407e-05
XBP1: 7.48451354987985e-05
FKH2: 0.000161566543109314

m_organization_of_chromosome_structure_n12
INO4: 0.000159778845501874
SFP1: 0.00224597014012086
MIG3: 0.00232403948182399

MCB
MBP1: 1.41939314989144e-45
SWI6: 1.01476000449615e-28
STB4: 0.000106562176468792

m_pentosephosphate_pathway_n5
m_pentose: 8.3716505148063e-05
m_pentose: 0.000257652222397286
m_pentose: 0.000275640370125405

m_organization_of_chromosome_structure_n17
SKN7: 3.22443457653714e-07
INO4: 5.2473219860667e-07
MTH1: 4.23350609882702e-05

m_pentosephosphate_pathway_n7
m_pentose: 6.03196845718822e-06
m_pentose: 1.61978807725104e-05

m_pentose: 2.10371222200956e-05

m_other_energy_generation_activities_n4
UME6: 3.63605968512852e-17
UME1: 0.000175102396589089
ABF1: 0.000231202490509861

m_homeostasis_of_other_ions_n30
GAT1: 3.67534106493052e-08
INO4: 2.18462930804362e-06
SWI4: 2.84960091634415e-06

m_glyoxylate_cycle_n11
UME6: 1.74253734970203e-17
GAT1: 2.05495862338789e-15
RGM1: 8.37065746097868e-14

m_other_energy_generation_activities_n9
UME6: 1.17596106974136e-06
RGM1: 5.25456853756531e-05
FKH2: 0.000538445886134675

m_RRSE3
SWI4: 0.000190259736206587
SKN7: 0.000473514586164473
INO4: 0.000526830917336093

m_aminoacid_transport_n3
m_amino: 1.7049440044179e-14
m_amino: 2.21826928073504e-09
m_amino: 6.10091730883474e-09

m_proteolysis_n8
PUT3: 7.7169155295361e-06
UME6: 2.09943632867044e-05
RGM1: 0.000232326461742917

m_drug_transporters_n10
INO4: 3.01805400194665e-07
MET4: 1.71180231932498e-06
SKN7: 3.97920445743505e-05

m_lipid_and_fattyacid_transport_n11
m_lipid_and_fatty: 4.26617582714689e-15
m_lipid_and_fatty: 2.80217639897803e-11
m_lipid_and_fatty: 4.67722582006401e-09

m_glyoxylate_cycle_n19
UME6: 3.09796841525807e-07
SWI6: 2.53477691645877e-06
SWI4: 9.47377053854465e-05

m_drug_transporters_n14
SMP1: 0.00137497576088794
SPT10: 0.0019771117895247
LEU3: 0.00767705224476747

m_homeostasis_of_metal_ions_n20
ACE2: 4.44565337338333e-08
HAP1: 1.53522602752177e-06
RAP1: 6.6554119041329e-06

m_ccompound_and_carbohydrate_metabolism_n8
m_c: 8.27433403413252e-08
m_c: 3.95204491596264e-07
m_c: 1.46959334190678e-06

m_metal_ion_transporters_n25
HAP1: 5.36249986167953e-05
SFP1: 0.00724554900422805

HAP4: 0.00939514115606972

m_purine_and_pyrimidine_transporters_n10
SKO1: 1.62100444764741e-06
YAP1: 9.89196863858646e-05
CIN5: 0.000483546856048389

m_organization_of_chromosome_structure_n20
GAT1: 6.38287721461822e-08
SKN7: 2.61715579304346e-07
UME6: 9.75587214450642e-05

m_metal_ion_transporters_n26
PHO4: 0.013326366483329
RGT1: 0.0144243467492455
PUT3: 0.0146105993136483

m_phosphate_utilization_n4
HAP4: 0.00515119248951407
UME6: 0.018347815279978
USV1: 0.0209734317219399

m_purine_and_pyrimidine_transporters_n17
GAT1: 6.23797796210094e-10
INO4: 2.7937571308198e-09
RGM1: 8.47030344044395e-08

m_chromatin_modification_n21
HSF1: 0.00315916174396563
CBF1: 0.00343971800394722
IFH1: 0.0113380169445401

m_vacuolar_and_lysosomal_organization_n8
FAP7: 0.00779606072162031
RTG1: 0.0131219664212927
SMK1: 0.0218867574225299

m_gproteins_n11
m_g: 4.43363858807584e-05
m_g: 0.00105821641563613
m_g: 0.00186276488673428

m_phosphate_utilization_n7
MIG3: 4.13876277889322e-05
YAP6: 0.000575950435234716
MET32: 0.00184374315617319

m_gproteins_n12
m_g: 5.4529411310455e-06
m_g: 0.000207049531506134
m_g: 0.000571078321780533

m_gproteins_n13
m_g: 0.00574840061158142
m_g: 0.00690664782248533
m_g: 0.0142341627769339

m_phosphate_utilization_n9
INO4: 0.0011367411637762
SKO1: 0.00160864708301497
GTS1: 0.00318864233557757

ABF1
ABF1: 6.51081155498384e-214
RTS2: 0.000467059699662903
SIG1: 0.00107617565267853

m_regulation_of_ccompound_and_carbohydrate_utilization_n18
m_regulation_of_c: 4.00652811044678e-05
m_regulation_of_c: 0.000108487336555493

m_regulation_of_c: 0.000147751478007904

m_ccompound_and_carbohydrate_utilization_n9
m_c: 8.47008489949816e-06
m_c: 2.15214131343128e-05
m_c: 3.88940512456369e-05

m_nutritional_response_pathway_n12
INO4: 6.16590682329135e-05
MTH1: 0.000136041422751288
MAL13: 0.000176689985673288

m_other_transport_facilitators_n5
INO4: 4.81600030331537e-08
SWI6: 5.70560457996572e-07
CIN5: 6.15192593974134e-07

m_pentosephosphate_pathway_n14
m_pentose: 1.02239026000171e-13
m_pentose: 1.13129405348625e-08
m_pentose: 3.47877818067024e-08

m_pentosephosphate_pathway_n19
m_pentose: 5.9069681948169e-06
m_pentose: 4.99419370180417e-05
m_pentose: 8.4319790744506e-05

m_pyrimidineribonucleotide_metabolism_n5
m_pyrimidine: 0.00393908782816138
m_pyrimidine: 0.00763692331807289
m_pyrimidine: 0.0118776050703533

m_biosynthesis_of_vitamins_cofactors_and_prosthetic_groups_n17.scn
SKN7: 0.000410947317127767
FAP7: 0.000570742529259672
OAF1: 0.00204165467793433

m_detoxificaton_n34
OAF1: 0.00079107662432237
DOT6: 0.00710180619128958
PHO4: 0.0100539497831483

m_nucleotide_metabolism_n11
INO4: 0.000191923406104759
AZF1: 0.00113082497508135
MBP1: 0.00152232342694666

GAL
UME6: 3.57512258664315e-08
TOS8: 9.32452331948586e-08
XBP1: 7.5606988536589e-07

m_nitrogen_and_sulphur_metabolism_n16
SWI6: 0.00179303092568546
SKN7: 0.00282263867225071
ARG81: 0.00724848106091822

m_detoxificaton_n39
RLM1: 0.00083098092793494
INO4: 0.00102527742686137
RIM101: 0.00107211307958769

m_nitrogen_and_sulphur_metabolism_n17
SKN7: 4.25080849205177e-08
GAT1: 7.25313859448417e-08
XBP1: 1.84696071953908e-05

m_nitrogen_and_sulphur_metabolism_n18
CBF1: 6.2304993782612e-05
MBP1: 0.000124677629169913

GAT1: 0.000361030916608299

m_fermentation_n3
UME6: 0.0030341338974965
RTG1: 0.00512794061312812
GAT1: 0.00631136868071782

m_fermentation_n4
INO4: 0.00097555489780277
PDR1: 0.00110435208194648
FKH2: 0.00121960215082755

m_biogenesis_of_chromosome_structure_n9
RLM1: 0.000414894597929574
RAP1: 0.000554806443330499
REB1: 0.0036967349518322

m_fermentation_n5
ASK10: 0.00582133544272515
MSN4: 0.0165865669283477
RIM101: 0.0178931953771116

m_metabolism_of_energy_reserves_n27
SKN7: 2.31218590145438e-07
NDD1: 5.42556986621048e-05
ACE2: 5.93905370098128e-05

m_metabolism_of_energy_reserves_n29
YAP1: 4.93598744204814e-05
SKN7: 0.000472406055928615
PHD1: 0.00982777842059429

m_extracellular_transport_n10
MTH1: 0.000598946774319443
AZF1: 0.00376848347694752
MIG3: 0.00516653198528126

m_trnasynthetases_n6
m_trna: 1.13740137187651e-06
m_trna: 5.6349630958501e-06
m_trna: 2.41035414396993e-05

m_purine_and_pyrimidine_transporters_n6
DAL80: 0.00109287040557034
STP4: 0.00348464643716178
HAP1: 0.00532500656137282

m_pentosephosphate_pathway_n21
m_pentose: 7.02720259135252e-05
m_pentose: 0.000250634832366445
m_pentose: 0.000718272083030954

m_nitrogen_and_sulphur_transport_n5
GZF3: 0.000296939564718367
MET4: 0.000339539418754097
CBF1: 0.00074559561891854

m_pentosephosphate_pathway_n23
m_pentose: 4.73202568248649e-06
m_pentose: 5.98514210285121e-06
m_pentose: 9.71413191392282e-06

m_organization_of_cytoplasm_n27
FHL1: 4.91949390967384e-07
RAP1: 4.21066477187903e-06
YAP5: 3.2119375629639e-05

m_ion_transporters_n3
INO4: 9.97698465191485e-07
MTH1: 1.14978143886636e-05

DOT6: 0.000532269308453146

m_nitrogen_and_sulphur_transport_n9
SFP1: 3.99397974949822e-07
RGM1: 2.04061302054784e-06
FHL1: 2.22311576783067e-06

m_ion_transporters_n4
GAT1: 1.77446826052445e-11
INO4: 4.56114868024544e-09
RGM1: 1.91581390287064e-06

m_MERE4
MTH1: 3.72346367131908e-14
SKN7: 9.27314227251666e-13
NDD1: 1.60335562945271e-09

m_ion_transporters_n6
ACE2: 4.75253795069055e-07
SKN7: 6.20495571651428e-07
MTH1: 7.15922583844645e-07

m_ion_transporters_n7
SKN7: 4.51654480907708e-06
TOS8: 1.08083623356498e-05
XBP1: 1.79332235470082e-05

m_nutritional_response_pathway_n3
SWI6: 1.93788065521309e-07
SWI4: 6.16841715162308e-07
SKN7: 1.19494443750387e-06

m_cytok9
SWI6: 9.93412795925263e-06
SWI4: 0.000341343799793754
DOT6: 0.000562960860934005

m_organization_of_intracellular_transport_vesicles_n5
MTH1: 4.76256701660378e-11
SKN7: 1.87777651706765e-07
SWI4: 2.67687043633309e-07

m_pheromone_response_generation_n4
PUT3: 0.000307365288607338
ECM22: 0.000406253940238564
SKN7: 0.000942850878451294

m_nitrogen_and_sulphur_metabolism_n22
GAT1: 3.61167598844347e-06
RGM1: 8.13911586515626e-06
SKN7: 8.75097746402262e-06

m_metabolism_of_energy_reserves_n30
PHD1: 4.19077193824825e-05
MIG3: 0.000166767113650987
BAS1: 0.00022549370987968

m_nitrogen_and_sulphur_transport_n14
SUT1: 0.0141866513341142
INO4: 0.0179396228869314
SKO1: 0.0314902346073282

m_nutritional_response_pathway_n7
SWI6: 0.000328327590114838
SWI4: 0.00164916589850802
ACE2: 0.00234626473322043

m_nutritional_response_pathway_n8
SKN7: 4.60422395805352e-07
INO4: 6.93168549536157e-07

XBP1: 5.14663740200764e-05

m_pheromone_response_generation_n7
SPT10: 9.83101645102062e-05
IFH1: 0.00149140534552906
SMK1: 0.00162054527868754

m_tricarboxylicacid_pathway_n3
m_tricarboxylic: 5.34471198776056e-06
m_tricarboxylic: 6.2687706823134e-06
m_tricarboxylic: 7.70424666001792e-06

m_nitrogen_and_sulphur_metabolism_n29
SKN7: 1.19192270891566e-07
MTH1: 2.68034772469987e-06
GAT1: 1.6673226130461e-05

m_aminoacid_biosynthesis_n9
m_amino: 6.23928414178809e-07
m_amino: 1.74065684111727e-05
m_amino: 0.000133797410375877

m_tricarboxylicacid_pathway_n6
m_tricarboxylic: 0.00210239337986837
m_tricarboxylic: 0.00254873755329481
m_tricarboxylic: 0.00294778476605026

m_other_pheromone_response_activities_n5
CBF1: 0.00020128123255304
STB2: 0.000397059998910323
GAT1: 0.00114892294633759

m_trna_transcription_n10
FKH1: 0.00320478698023998
SWI6: 0.00654370510187466
YAP6: 0.012202364140247

m_tricarboxylicacid_pathway_n9
m_tricarboxylic: 1.25050305976799e-09
m_tricarboxylic: 1.58112856817045e-06
m_tricarboxylic: 8.5609326089385e-05

m_pheromone_response_generation_n10
DIG1: 0.00134210770607427
ADR1: 0.00221024402179837
IFH1: 0.00244406975710661

m_other_pheromone_response_activities_n8
SWI6: 1.55475488989323e-07
INO4: 2.14754750445919e-06
FKH2: 1.39797827756412e-05

m_pheromone_response_generation_n12
MET4: 3.42836577423311e-06
FKH1: 0.000168888348846813
IME1: 0.00168876970606473

m_organization_of_cell_wall_n6
MET32: 1.95792618305618e-05
MCM1: 0.000109618879616045
RLM1: 0.000462319655500605

m_organization_of_cell_wall_n8
PHD1: 7.01181015391447e-07
INO4: 2.66558831948874e-06
SWI4: 3.05511308353023e-06

m_biogenesis_of_chromosome_structure_n18
RTG3: 0.0052222338357761
HAP1: 0.0076669835301605

YBR239c: 0.00838963800121919

m_RPE6
RGM1: 3.58143873793957e-22
GAT3: 5.78129238075292e-21
FHL1: 1.54533031457453e-20

m_RPE8
RGM1: 3.63299093848644e-13
YAP5: 4.50847812326991e-11
FHL1: 5.31938569591011e-11

m_biogenesis_of_cytoskeleton_n5
AZF1: 0.000212207113468308
SKN7: 0.00135682542457717
MIG3: 0.00319875406386215

Gcr1
NDD1: 0.000187378124329151
GCR1: 0.000336334966610521
SWI6: 0.000602398143683105

m_nitrogen_and_sulphur_metabolism_n31
UME6: 4.56774023672851e-05
CIN5: 0.000195987221273364
SWI4: 0.00130589136531054

PAC
ABF1: 3.014533986825e-05
REB1: 0.00263262367317767
YDR026c: 0.00266556183080568

PHO4
CBF1: 2.17564341142812e-31
INO4: 2.74938310712983e-10
TYE7: 7.57378180385721e-08

Yap1
CAD1: 1.1611247314217e-22
YAP1: 4.39829259434032e-13
RTG1: 1.85523073248386e-05

m_lysosomal_and_vacuolar_degradation_n3
YAP3: 0.000940441706731405
RGM1: 0.00550689119687094
KSS1: 0.0127252475412599

m_lysosomal_and_vacuolar_degradation_n8
SPT10: 0.00337438790398326
INO4: 0.0052731855064239
WTM2: 0.00702918983445336

m_other_energy_generation_activities_n11
UME6: 0.000306576459724763
GAT1: 0.00423116209370769
HAP1: 0.00574982033162517

m_other_energy_generation_activities_n12
SWI6: 4.85791108609511e-07
FKH1: 1.30745304741951e-06
MTH1: 2.08374517994277e-05

m_phosphate_transport_n5
INO4: 3.15053462708228e-06
GAT1: 0.00775962093316353
SKN7: 0.0232207242787798

m_abc_transporters_n2
SKN7: 4.61574466944225e-08
GAT1: 1.65292950618531e-07

SWI4: 3.34248295349807e-06

m_glycolysis_and_gluconeogenesis_n11
MBP1: 0.000146914308053835
ADR1: 0.000208699454517613
RDS1: 0.000338288534039095

m_phosphate_transport_n8
SWI5: 0.000105220446019336
INO4: 0.000549370100001136
SWI4: 0.000581782665038547

m_other_energy_generation_activities_n16
SKN7: 0.000147074356278931
SWI4: 0.000201198913278017
INO4: 0.000362558434463593

m_abc_transporters_n5
DOT6: 6.31386567995493e-07
CAD1: 0.00534333078380501
YAP6: 0.00799028068023046

m_glycolysis_and_gluconeogenesis_n14
MTH1: 4.04621401366198e-11
RGM1: 1.36298424781656e-07
INO4: 3.71238414001119e-07

m_other_energy_generation_activities_n17
TEC1: 0.000449768986169191
RLM1: 0.000490731901927476
YAP3: 0.00095605105931018

m_other_proteolytic_degradation_n2
MTH1: 1.10932415913658e-09
RGM1: 1.18722475817644e-07
SFP1: 1.64833625795172e-05

m_other_proteolytic_degradation_n5
NRG1: 1.40043857094918e-05
CIN5: 0.00031337757130764
PDR1: 0.000626950677099624

MIG1
MIG3: 3.88435270498322e-08
SUT1: 5.72336714455647e-08
SKO1: 1.77815610482868e-07

m_other_proteolytic_degradation_n7
SWI6: 0.00170647911327217
YLR278C: 0.00549544466675638
RPH1: 0.0068302771572336

m_other_proteolytic_degradation_n8
INO2: 5.00132314929742e-05
FHL1: 0.00214188905335796
ARO80: 0.00438172791981092

m_glyoxylate_cycle_n7
ASH1: 2.53244379984374e-09
MTH1: 3.70735855357612e-06
CIN5: 4.65245466699917e-06

m_organization_of_cell_wall_n10
SKN7: 5.6262259691823e-05
MAL33: 0.000223784004958237
GAT1: 0.000670808665731142

m_lipid_and_fattyacid_binding_n12
m_lipid_and_fatty: 0.00300478600575226
m_lipid_and_fatty: 0.00370603838082381

m_lipid_and_fatty: 0.0157771949678944

m_glyoxylate_cycle_n8
MTH1: 4.88764494953061e-09
INO4: 8.79303562361219e-09
UME6: 1.90280561689332e-08

m_lipid_and_fattyacid_binding_n13
m_lipid_and_fatty: 2.1357813873674e-05
m_lipid_and_fatty: 0.00388468837699866
m_lipid_and_fatty: 0.00454195643696627

m_lipid_and_fattyacid_binding_n14
m_lipid_and_fatty: 0.000697959639511086
m_lipid_and_fatty: 0.000978584899405222
m_lipid_and_fatty: 0.00164603914395689

m_organization_of_centrosome_n5
SUM1: 0.007941660581652
ACA1: 0.0145576239232778
CRZ1: 0.0249820029608252

m_organization_of_cell_wall_n14
PHO2: 7.17078367003117e-05
FHL1: 0.00308976046013303
CBF1: 0.00620777254541898

m_lipid_and_fattyacid_binding_n15
m_lipid_and_fatty: 6.09068591611816e-07
m_lipid_and_fatty: 2.93815546086137e-05
m_lipid_and_fatty: 0.000530183720942343

m_other_cell_rescue_activities_n10
RGM1: 0.00013704212111407
SMP1: 0.000513621027438905
PHO4: 0.00734648030765783

m_organization_of_centrosome_n6
STB2: 0.000378262416761373
HIR1: 0.00374129467657081
MAC1: 0.00655747703437271

m_aminoacid_transporters_n11
m_amino: 7.90276180082197e-10
m_amino: 3.61229414589958e-08
m_amino: 1.49895438210796e-07

m_anion_transporters_n4
MTH1: 1.04967729519551e-10
INO4: 2.37855525726864e-08
OAF1: 2.29164690058362e-06

m_regulation_of_aminoacid_metabolism_n10
m_regulation_of_amino: 1.67978788894312e-07
m_regulation_of_amino: 2.62145312964964e-07
m_regulation_of_amino: 2.78944457043044e-06

m_regulation_of_aminoacid_metabolism_n11
m_regulation_of_amino: 0.000106528116570325
m_regulation_of_amino: 0.000320863516407514
m_regulation_of_amino: 0.0010259819170241

m_anion_transporters_n9
DOT6: 0.000996912955123204
SKN7: 0.00144914748254893
MTH1: 0.0016061006319549

m_regulation_of_aminoacid_metabolism_n15
m_regulation_of_amino: 2.76286447573246e-08
m_regulation_of_amino: 2.4766955125997e-07

m_regulation_of_amino: 2.75558288299835e-07

m_other_energy_generation_activities_n20
INO4: 9.02919037284545e-08
GAT1: 0.000349761393371121
FKH1: 0.00219205612241115

m_other_energy_generation_activities_n22
HIR1: 4.60594214241345e-05
INO4: 0.000404402242125797
PHO4: 0.00123163933072617

m_allantoin_and_allantoate_transporters_n11
RIM101: 0.000971492669614567
UME6: 0.00425672512593853
MAL33: 0.00529976631200403

SWI5
INO4: 2.21369575000863e-11
SKN7: 1.79823516094205e-08
SWI6: 2.30127138346271e-08

m_allantoin_and_allantoate_transporters_n12
OAF1: 6.35948687541498e-06
ACE2: 0.000515212604711114
SKO1: 0.00061200815645167

m_allantoin_and_allantoate_transporters_n13
MAL13: 0.000130439017902673
STB2: 0.00262054741486525
SWI6: 0.00369005483019613

m_regulation_of_nitrogen_and_sulphur_utilization_n10
MTH1: 1.48213700759265e-05
SKO1: 3.36719828732239e-05
ROX1: 0.000223253825149577

m_other_mrnatranscription_activities_n11
m_other_mrna: 6.05138265562396e-08
m_other_mrna: 4.23148523645151e-07
m_other_mrna: 5.29994953471029e-07

m_regulation_of_nitrogen_and_sulphur_utilization_n12
SWI6: 3.43284299803174e-05
INO4: 0.000119955632310037
SWI5: 0.000151076836158067

m_aminoacid_metabolism_n14
m_amino: 8.30727683640798e-08
m_amino: 4.93702439214477e-07
m_amino: 5.4558734924842e-06

m_glycolysis_and_gluconeogenesis_n27
SWI6: 1.22323098558889e-05
SWI4: 4.45600737844357e-05
UME6: 0.0001209188233969

m_allantoin_and_allantoate_transporters_n17
GCN4: 0.00394675047035596
HAC1: 0.00839303944208345
OAF1: 0.0172716816909211

m_regulation_of_nitrogen_and_sulphur_utilization_n13
UME6: 2.49790563307142e-07
ACE2: 8.15977895209807e-06
MTH1: 1.50361344243776e-05

m_allantoin_and_allantoate_transporters_n18
FKH2: 0.000415894780447537
YAP1: 0.000738889428726443

HAP2: 0.00110909533623326

m_organization_of_cell_wall_n20
CRZ1: 0.000842829400191828
UME6: 0.00497393825418766
MGA1: 0.00717774293838217

m_regulation_of_lipid_fattyacid_and_isoprenoid_biosynthesis_n20.scn
m_regulation_of_lipid_fatty: 1.04936812056067e-05
m_regulation_of_lipid_fatty: 3.46277519396458e-05
m_regulation_of_lipid_fatty: 4.87015197862451e-05

m_aminoacid_degradation_n7
m_amino: 7.03667310428695e-07
m_amino: 8.49681749941431e-07
m_amino: 3.41779446965204e-06

m_aminoacid_degradation_n8
m_amino: 0.000112725011832933
m_amino: 0.000137510265407786
m_amino: 0.000166026873059729

m_polynucleotide_degradation_n3
SFP1: 0.00517526265879041
MSN2: 0.013035447240273
CAD1: 0.0254590045477733

m_peroxisomal_transport_n15
IFH1: 0.000494990400571306
SKN7: 0.00223642085459001
PDC2: 0.00249085313475092

m_utilization_of_vitamins_cofactors_and_prosthetic_groups_n5
STB6: 0.00166516501329859
HAP3: 0.00640713897726557
IME1: 0.00685811253283861

HSE
YML081W: 1.31562212670246e-05
HSF1: 2.51319232751667e-05
RIM101: 0.00797411053917014

m_assembly_of_protein_complexes_n23
OAF1: 3.70799699174346e-06
PIP2: 0.00209672529382356
RGM1: 0.00222512399655743

m_utilization_of_vitamins_cofactors_and_prosthetic_groups_n6
YER051w: 0.00165738713690428
YER130C: 0.0017150575881003
GTS1: 0.00386564677697205

m_peroxisomal_transport_n19
UME6: 6.84579886535336e-05
ECM22: 0.000155656737149209
YAP5: 0.000793525935369616

m_utilization_of_vitamins_cofactors_and_prosthetic_groups_n7
RLM1: 8.85219078840359e-06
RPN4: 0.0012757069357361
HSF1: 0.00256286145501766

m_other_proteindestination_activities_n7
m_other_protein: 1.16435587830149e-05
m_other_protein: 3.19417325007974e-05
m_other_protein: 9.25619574577196e-05

m_cellular_import_n12
MIG3: 5.17239419139785e-07
CIN5: 5.17770309034836e-06

CUP9: 2.62517934874099e-05

m_biosynthesis_of_vitamins_cofactors_and_prosthetic_groups_n8
YAP3: 0.00727228587320275
MET4: 0.00745484436479942
MAC1: 0.00815924097446242

REB1
REB1: 6.8478832574409e-88
UME6: 7.90263175865878e-05
YDR026c: 0.00100080670667541

CSRE
UME6: 4.86815521819253e-12
INO4: 4.43971728044194e-09
SKN7: 4.76115595415744e-09

m_other_mrnatranscription_activities_n20
m_other_mrna: 4.51597429896278e-15
m_other_mrna: 3.68819553814208e-12
m_other_mrna: 1.34602601283619e-11

m_aminoacid_metabolism_n25
m_amino: 4.61934253333022e-13
m_amino: 1.17487700934108e-08
m_amino: 3.29376287187719e-07

m_stress_response_n17
RGM1: 3.52457452889033e-09
MTH1: 3.42027159772018e-07
GAT1: 4.62102003687901e-07

m_phosphate_transport_n13
SKN7: 1.0037254538829e-07
GAT1: 1.2048733123678e-06
RGM1: 6.46712095471149e-06

m_deoxyribonucleotide_metabolism_n4
SWI6: 4.76704828342437e-05
FKH2: 0.000488172818462367
TBS1: 0.00407070138187618

m_phosphate_transport_n18
GAT1: 1.61023409494401e-14
INO4: 3.10953249425969e-14
XBP1: 8.47533425725024e-14

m_deoxyribonucleotide_metabolism_n5
GAT1: 4.09397207459517e-07
MTH1: 1.30351162477328e-06
UME6: 1.21173688679412e-05

m_peroxisomal_transport_n22
RAP1: 4.19155076499594e-06
RGM1: 4.28856349222717e-06
TOS8: 1.04670914647941e-05

m_metal_ion_transporters_n6
REB1: 0.000627021497139404
ADR1: 0.00182014489937943
YAP1: 0.00215580514637906

m_deoxyribonucleotide_metabolism_n8
MAL13: 0.00203966763216669
HSF1: 0.00512811931388971
YAP1: 0.00633709452799318

ALPHA1'
YER051w: 2.79631082919245e-06
RIM101: 9.56776778603689e-06

SFP1: 1.2631768631272e-05

m_sugar_and_carbohydrate_transporters_n14
RAP1: 9.24914467088513e-05
SKO1: 0.000314998140439489
FKH1: 0.000335089342173223

m_aminoacid_degradation_n24
m_amino: 8.58080119807757e-06
m_amino: 8.63225121381066e-06
m_amino: 2.16850030158008e-05

m_organization_of_cytoplasm_n72
MTH1: 0.000494204430456395
YAP5: 0.00162458389322153
IXR1: 0.00194546686967112

m_aminoacid_degradation_n25
m_amino: 5.16219629559095e-06
m_amino: 0.000134630433098237
m_amino: 0.000225971469669978

PDR
UME6: 1.75453246830555e-10
INO4: 1.18440206372138e-08
SUT1: 1.00709823197326e-06

mRRPE
ABF1: 1.25510731821095e-08
HIR1: 0.000111137712585841
RLM1: 0.000154887279141361

m_aminoacid_degradation_n27
m_amino: 2.10806632484596e-06
m_amino: 0.000208537664187521
m_amino: 0.000279611494880637

m_regulation_of_lipid_fattyacid_and_isoprenoid_biosynthesis_n7.scn
m_regulation_of_lipid_fatty: 3.19345811801606e-05
m_regulation_of_lipid_fatty: 0.00101238286654632
m_regulation_of_lipid_fatty: 0.00635548079375445

m_aminoacid_degradation_n29
m_amino: 2.0707565721298e-06
m_amino: 1.18479740500189e-05
m_amino: 1.45540340953625e-05

m_organization_of_golgi_n7
MET4: 0.000370135874364089
MAL13: 0.00121573319252826
FKH1: 0.00160249779486184

m_other_nutritionalresponse_activities_n6
m_other_nutritional: 1.38761353679538e-08
m_other_nutritional: 2.88366027335551e-05
m_other_nutritional: 0.000127850561992644

AFT1
RAP1: 4.50219856592722e-06
MAC1: 0.000458607528134524
PDR1: 0.000661852894314361

m_stress_response_n24
MTH1: 2.639812920367e-08
SWI4: 6.79310433095219e-07
SKN7: 1.43680287220173e-05

m_other_transcription_activities_n5
SWI6: 2.54566955129474e-07
MBP1: 8.88143952060158e-06

HAP1: 9.065895277194e-06

m_other_transcription_activities_n8
GAT1: 5.44717788536329e-06
MAL13: 1.78338446158845e-05
MTH1: 0.00021172219702625

m_translational_control_n10
STB2: 0.00336023905902538
YGR067C: 0.00544157669609023
NDT80: 0.0055721654677111

m_phosphate_metabolism_n16
MTH1: 2.8967054618919e-05
GAT1: 3.05322455870557e-05
ECM22: 0.000842057428974347

Ume6(URS1)
UME6: 1.74929739951136e-70
UME1: 1.77156914414105e-19
ABF1: 2.98446262658455e-09

m_phosphate_metabolism_n18
MET4: 0.000219996148501796
FKH1: 0.000372097762016282
RPH1: 0.000898586345769069

zap1
GAT1: 0.000107104044433959
PUT3: 0.000247635533937347
ZAP1: 0.000271332230875359

ALPHA2'
GCN4: 0.000293967458117515
FAP7: 0.00335549299486025
PIP2: 0.00423196525677823

m_aminoacid_degradation_n32
m_amino: 0.000507412196440627
m_amino: 0.0105877917267317
m_amino: 0.0129478324646446

m_fermentation_n10
MTH1: 2.4641747515185e-05
GAT1: 3.34734703987206e-05
YRR1: 0.00159541376379105

STRE'
SWI5: 7.83130778713141e-06
MAL13: 8.69183882145445e-06
MBP1: 3.35836865268633e-05

m_fermentation_n12
RIM101: 0.000527784028830139
GTS1: 0.00342522005714535
HSF1: 0.00936821543787652

m_fermentation_n14
INO4: 0.000218149204620878
INO2: 0.00306186103080597
SUT2: 0.0148705802811703

m_fermentation_n18
INO4: 0.000603808312154073
ABF1: 0.00116005121806844
GLN3: 0.00326227112790305

m_other_signaltransduction_activities_n13
m_other_signal: 0.000581777689235879
m_other_signal: 0.000594240792265388

m_other_signal: 0.0029922238265967

m_other_nucleotidemetabolism_activities_n17
m_other_nucleotide: 0.000570293492935289
m_other_nucleotide: 0.000926281783576027
m_other_nucleotide: 0.00432893969340488

m_ionic_homeostasis_n6
YAP1: 1.04862044059436e-08
SWI6: 1.60007518856358e-06
SKN7: 1.94458022233733e-05

m_other_signaltransduction_activities_n15
m_other_signal: 0.000268932746683354
m_other_signal: 0.000833278246907098
m_other_signal: 0.00176248853641293

m_other_nucleotidemetabolism_activities_n18
m_other_nucleotide: 0.00200344536128041
m_other_nucleotide: 0.00497686490956764
m_other_nucleotide: 0.0068612286045041

m_stress_response_n36
PHD1: 0.000306777716023489
MTH1: 0.000676738877463286
MET4: 0.00109668672167371

m_regulation_of_lipid_fattyacid_and_isoprenoid_biosynthesis_n12.scn
m_regulation_of_lipid_fatty: 3.00063547271362e-15
m_regulation_of_lipid_fatty: 1.25521052646266e-14
m_regulation_of_lipid_fatty: 3.76613352844683e-12

m_ion_transporters_n10
RAP1: 7.90689800769606e-09
GAT1: 2.27954491175352e-08
MTH1: 7.21345937023183e-07

m_RPE11
RGM1: 2.9896683396203e-13
FHL1: 4.44305386776597e-11
RAP1: 2.1763778769826e-10

m_ion_transporters_n11
SWI4: 2.95293614906722e-15
SWI6: 2.00398162127663e-09
SKN7: 1.5857861676581e-08

m_regulation_of_lipid_fattyacid_and_isoprenoid_biosynthesis_n16.scn
m_regulation_of_lipid_fatty: 4.55239537025595e-09
m_regulation_of_lipid_fatty: 1.02297748321617e-05
m_regulation_of_lipid_fatty: 2.33261087027515e-05

m_ion_transporters_n14
INO4: 2.79050398848614e-05
RAP1: 0.000112639445021552
MTH1: 0.000280139368142684

m_RPE17
RGM1: 2.51548595526691e-20
FHL1: 4.33203730881754e-20
RAP1: 1.95361527000086e-19

LYS14
YAP6: 2.54818099361971e-05
SWI6: 3.79915129903173e-05
SKN7: 5.16122920241163e-05

m_transport_atpases_n17
ABF1: 6.77005861504317e-06
UME6: 0.00241337880576876

SKN7: 0.00442829117141095

STRE
TOS8: 1.87113689322576e-06
SWI6: 7.5744450878779e-06
RAP1: 1.31352177600116e-05

m_metabolism_of_cyclic_and_unusual_nucleotides_n5
HAC1: 0.000317388825165739
YAP1: 0.00121583480228555
NDD1: 0.00196807097519253

m_regulation_of_nitrogen_and_sulphur_utilization_n7
INO4: 3.28532179685709e-09
RAP1: 5.04601853448024e-05
CBF1: 0.000116283818231207

m_other_intracellulartransport_activities_n6
m_other_intracellular: 0.00441363666650857
m_other_intracellular: 0.00972118404925808
m_other_intracellular: 0.0173986416134105

RPN4
ABF1: 7.80264827001799e-09
REB1: 1.11515357877553e-06
GAT1: 7.03763669904623e-06

m_other_intracellulartransport_activities_n9
m_other_intracellular: 0.00021186390727829
m_other_intracellular: 0.000389738173877808
m_other_intracellular: 0.00148273155144248

m_fermentation_n21
SKN7: 6.66961440479065e-05
SFP1: 8.44685621207604e-05
MSN2: 0.000483245333902344

OAF1
GAT1: 0.000401063206058887
STB5: 0.00093586982803691
SFP1: 0.00128215845580065

m_ccompound_and_carbohydrate_utilization_n31
m_c: 6.59401911728258e-05
m_c: 8.32112849185756e-05
m_c: 0.000175023936232635

STE12
STE12: 1.3577344157494e-09
TEC1: 8.55271152870755e-07
DIG1: 0.000544203895987083

m_morphogenesis_n5
RTG3: 0.000753211718236402
RTG1: 0.00457493118852995
SWI6: 0.00493524467807387

m_other_cation_transporters_n13
INO4: 1.11230984805526e-05
SWI4: 2.57376477096383e-05
GAT1: 0.00021186390727829

m_other_cation_transporters_n14
RAP1: 1.27529368202155e-05
MET32: 0.000390263913049678
OAF1: 0.00054020153345149

m_RPE21
FHL1: 1.277388343114e-24
RGM1: 7.31478385200757e-16

SMP1: 2.42978078198128e-15

m_other_cation_transporters_n18
DOT6: 2.36019072214998e-05
UME6: 4.5747808265368e-05
OAF1: 0.000131051315911418

m_MERE11
INO4: 1.37294165219692e-07
SUT1: 2.47325872213721e-06
HAP1: 1.08065588544118e-05

Leu3
SKN7: 2.17432068135564e-07
LEU3: 1.45781653543413e-06
INO4: 0.000219836398966402

mPROTEOL18(m_proteolysis_n18)
MBP1: 7.00294570714152e-10
SWI6: 1.05298923069683e-09
UME6: 1.91840930917768e-07

m_MERE16
RGM1: 1.21213739083715e-09
MTH1: 4.46416260747437e-08
SKN7: 2.77194499708679e-07

m_MERE17
FHL1: 2.08665083788627e-09
MTH1: 2.32710467479716e-08
MAL13: 1.17490845381093e-07

m_stress_response_n4
SKN7: 6.86880941832911e-09
ACE2: 1.90034656570888e-05
RGM1: 3.16102470696767e-05

m_other_cation_transporters_n3
INO4: 1.69757284942102e-11
OAF1: 6.65969453370885e-06
MTH1: 4.2955271503586e-05

m_other_cation_transporters_n5
RGM1: 6.96082579771904e-07
SKN7: 0.000244866570487547
OAF1: 0.000325326547386202

m_nitrogen_and_sulphur_utilization_n15
MET32: 0.00239289500556893
MAL13: 0.00448577079647436
KRE33: 0.00815425157616572

m_anion_transporters_n10
INO4: 1.60184563149682e-10
ACE2: 4.39885708861127e-08
MTH1: 4.7590048933753e-08

m_other_cation_transporters_n7
SWI6: 0.000299868512109761
ADR1: 0.000305625358380776
FKH1: 0.000609267065596326

m_other_cation_transporters_n8
RAP1: 1.85196522876128e-06
ABF1: 1.80663596459607e-05
INO2: 2.33607925786658e-05

m_nitrogen_and_sulphur_utilization_n17
UME6: 0.0038294448281859
INO4: 0.0305716659470589

SFP1: 0.0351991241699657

m_nitrogen_and_sulphur_utilization_n19
RAP1: 6.18874619411277e-05
MTH1: 0.000154181338779664
MET4: 0.000534875450147534

m_anion_transporters_n13
MTH1: 1.62065519621864e-06
RGM1: 0.000238497497386342
SWI6: 0.000382729231173977

m_anion_transporters_n15
MTH1: 2.58409325571121e-11
INO4: 3.13719109986787e-11
GAT1: 1.68078518160146e-08

m_anion_transporters_n16
INO4: 0.000470363465985966
SWI5: 0.00196846409481722
FKH2: 0.00265188745898009

m_anion_transporters_n17
UME6: 1.2634788589592e-06
INO4: 1.34084710592593e-05
SKN7: 3.14067977762964e-05

m_anion_transporters_n19
OAF1: 1.23993229905569e-06
MTH1: 0.000262206761843676
XBP1: 0.00228604861541091

m_cytokinesis_n10
UME1: 0.000347437635619529
SFP1: 0.00121840310781593
YML081W: 0.00213312241980789

m_cytokinesis_n11
SWI6: 0.000192913864555685
SWI5: 0.00381146154428858
RME1: 0.00444885137925288

m_nitrogen_and_sulphur_utilization_n4
IME1: 0.0120652757408725
CIN5: 0.0148800514607204
MET32: 0.016355738129717

HAP234
HAP4: 2.07113217328185e-29
HAP1: 1.45890307965126e-06
HAP2: 4.24594313736567e-06

m_intracellular_communication_n4
SWI5: 1.75090609554628e-06
FKH1: 3.56896175233639e-05
ACE2: 5.93905370098128e-05

m_abc_transporters_n10
MBP1: 8.97557028461524e-05
SWI6: 0.000586010771636885
PHD1: 0.000933072252355357

m_RPE32
FHL1: 1.56067034187747e-12
RAP1: 1.27864152511165e-10
YAP5: 1.84807089711206e-09

m_RPE34
RGM1: 1.63923373417964e-17
FHL1: 4.04172409917235e-17

YAP5: 1.15427757445973e-13

m_trna_processing_n6
MSN2: 5.96132628912483e-06
RME1: 0.000588764871801161
ARO80: 0.000899673659586509

m_trna_processing_n9
SFP1: 6.90879716397756e-06
INO4: 2.85392340805942e-05
GAT1: 6.35901227207059e-05

m_cell_death_n15
RLM1: 0.0012933868358819
RTG1: 0.00331980257689467
CRZ1: 0.00383286675801842

m_meiosis_n3
UME6: 5.69823248189224e-114
UME1: 7.55461338431318e-28
ABF1: 9.29224002024608e-07

m_cell_death_n16
INO4: 5.83848452558523e-12
SKN7: 6.46432508199267e-12
GAT1: 1.15163612358779e-10

m_anion_transporters_n20
MTH1: 5.81576108862695e-09
GAT1: 1.69105845680337e-08
RAP1: 1.09989618052731e-06

CCA
MAL13: 1.68909023139435e-05
SFP1: 7.83472021775342e-05
MET4: 0.00024623505042265

m_anion_transporters_n22
SKN7: 3.88576804103373e-11
RAP1: 6.37795497469916e-09
INO4: 2.33031171368554e-07

PHO
INO4: 2.65165355183922e-07
NDD1: 3.57783456440338e-07
MET4: 1.97426614054773e-05

m_anion_transporters_n23
INO4: 1.08611552184977e-08
SFP1: 5.05916559045847e-08
RAP1: 4.41995264095408e-07

m_rRSE10
MTH1: 0.000230489479882944
GAT1: 0.000235597662330172
PDR1: 0.000246553045554827

m_regulation_of_lipid_fattyacid_and_isoprenoid_biosynthesis_n8.scn
m_regulation_of_lipid_fatty: 1.28036834520482e-11
m_regulation_of_lipid_fatty: 1.62931196334974e-07
m_regulation_of_lipid_fatty: 1.5175088344507e-06

m_other_cell_growth_cell_division_and_dna_synthesis_activities_n10.scn
SPT23: 0.00161681406433061
NDD1: 0.00525629546675331
HAP1: 0.0065056262944867

m_chromatin_modification_n9
CBF1: 0.00160373973356389
ASH1: 0.00519109673266301

MBP1: 0.0110908989846123

m_anion_transporters_n27
MAL33: 9.84664582457698e-05
MTH1: 0.000131195932049707
ACE2: 0.000491177895906596

m_aminoacid_transport_n13
m_amino: 8.45102779935954e-07
m_amino: 3.36838363780671e-05
m_amino: 0.000164101409909202

m_other_cell_growth_cell_division_and_dna_synthesis_activities_n14.scn
INO4: 0.000504526499028734
SFP1: 0.00053309643973929
RGM1: 0.000857910241179138

m_aminoacid_transport_n14
m_amino: 1.10684792655428e-09
m_amino: 1.03330120927424e-08
m_amino: 1.19676870313921e-08

m_breakdown_of_lipids_fatty_acids_and_isoprenoids_n8
MBP1: 4.8294450536232e-07
SWI6: 2.49729320519482e-06
INO4: 1.35775227679019e-05

m_sugar_and_carbohydrate_transporters_n6
MIG3: 5.22090325153602e-05
INO4: 0.0013615416586633
SKN7: 0.00163410204278186

m_aminoacid_transport_n18
m_amino: 7.28361659795334e-06
m_amino: 2.99597378199179e-05
m_amino: 0.000223686320614541

m_cytoskeletondependenttransport_n4
m_cytoskeleton: 0.000707534689578075
m_cytoskeleton: 0.0018619969397103
m_cytoskeleton: 0.00357257959305367

m_cell_death_n8
SKN7: 3.6667726477532e-12
RGM1: 9.55968603318525e-09
XBP1: 9.31766128446735e-08

ATRepeat
UME6: 0.000407137975139743
HAP1: 0.00041366922877769
GAT1: 0.000446396425761722

SCB
AZF1: 3.67342942613943e-08
UME6: 1.01777564186343e-05
SWI4: 3.30945357865405e-05

m_lipid_transporters_n8
PHO2: 8.39884392095007e-05
MET4: 0.00022240528462524
ECM22: 0.000320293216758751

m_cell_rescue_defense_cell_death_and_ageing_n20
ACE2: 9.52264350270162e-08
RAP1: 5.16376411253031e-06
SWI5: 5.5341154516556e-06
