# Supplementary material for: Revealing cell cycle control by combining model-based detection of periodic expression with novel cis-regulatory descriptors
Source: BMC Syst Biol. 2007 Oct 16;1:45. doi: 10.1186/1752-0509-1-45 (PMC2200664; doi:10.1186/1752-0509-1-45)
Supplement: Additional file 2 — All rules. All rules associating combinations of cis-regulatory descriptors with periodic classes of expression. The p-value for the periodic class that the rule was induced for is given together with the parameters for the the hypergeometric distribution: (N,n,k,x), where N is the number of genes in the data set, n is the number of genes matching the rule, k is the number of genes in the periodic class and x is the number of genes matched by the rule and the periodic class. [file 1752-0509-1-45-S2.pdf]

All rules associating combinations of *cis*-regulatory descriptors with periodic classes of expression. The p-value for the periodic class that the rule was induced for is given together with the parameters for the the hypergeometric distribution: (N,n,k,x), where N is the number of genes in the data set, n is the number of genes matching the rule, k is the number of genes in the periodic class and x is the number of genes matched by the rule and the periodic class.

IF MCM1-MCM1 THEN 000 OR 010 OR 100 OR 110 OR 111
110: 6.514436e-11 (1644,29,115,15)

IF SFF'-MCM1 AND MCM1-MCM1 THEN 000 OR 010 OR 100 OR 110 OR 111
110: 2.875084e-10 (1644,27,115,14)

IF SFF-FKH1 AND SFF-FKH2 THEN 000 OR 010 OR 100 OR 110 OR 111
110: 2.284099e-09 (1644,46,115,17)

IF ECB-MCM1 THEN 000 OR 010 OR 100 OR 110 OR 111
110: 2.992094e-09 (1644,31,115,14)

IF SFF'-MCM1 AND ECB-MCM1 THEN 000 OR 010 OR 100 OR 110 OR 111
110: 4.316222e-09 (1644,27,115,13)

IF MCM1-MCM1 AND ECB-MCM1 THEN 000 OR 010 OR 100 OR 110 OR 111
110: 1.037328e-08 (1644,24,115,12)

IF SFF'-FKH1 AND SFF'-FKH2 THEN 000 OR 010 OR 100 OR 110 OR 111
110: 2.007343e-08 (1644,52,115,17)

IF MCM1'-NDD1 AND ECB-NDD1 THEN 000 OR 010 OR 110 OR 111
110: 2.393468e-08 (1644,21,115,11)

IF MCM1-NDD1 AND MCM1'-NDD1 THEN 000 OR 010 OR 100 OR 110 OR 111
110: 2.393468e-08 (1644,21,115,11)

IF MCM1-NDD1 THEN 000 OR 010 OR 100 OR 110 OR 111
110: 3.284751e-08 (1644,26,115,12)

IF ECB-NDD1 THEN 000 OR 010 OR 110 OR 111
110: 1.413857e-07 (1644,24,115,11)

IF MCM1'-NDD1 THEN 000 OR 001 OR 010 OR 100 OR 110 OR 111
110: 1.451179e-07 (1644,52,115,16)

IF MCM1-MCM1 AND MCM1-YOX1 THEN 000 OR 010 OR 110 OR 111
110: 1.771141e-07 (1644,12,115,8)

IF MCM1-YOX1 AND MCM1'-MCM1 THEN 000 OR 010 OR 110 OR 111
110: 1.771141e-07 (1644,12,115,8)

IF MCM1'-MCM1 AND MCM1'-NDD1 THEN 000 OR 010 OR 100 OR 110 OR 111
110: 1.950730e-07 (1644,20,115,10)

IF MCM1-FKH2 AND MCM1-NDD1 THEN 000 OR 010 OR 100 OR 110 OR 111
110: 2.233389e-07 (1644,16,115,9)

IF MCM1-MCM1 AND MCM1-NDD1 THEN 000 OR 010 OR 100 OR 110 OR 111
110: 2.233389e-07 (1644,16,115,9)

IF MCM1'-NDD1 AND ECB-MCM1 THEN 000 OR 010 OR 110 OR 111
110: 4.337507e-07 (1644,13,115,8)

IF MCM1'-MCM1 AND ECB-NDD1 THEN 000 OR 010 OR 110 OR 111
110: 4.337507e-07 (1644,13,115,8)

IF MCM1-NDD1 AND ECB-NDD1 THEN 000 OR 010 OR 110 OR 111
110: 4.469216e-07 (1644,17,115,9)

IF MCM1'-MCM1 THEN 000 OR 001 OR 010 OR 100 OR 101 OR 110 OR 111

110: 6.015156e-07 (1644,57,115,16)

IF SFF'-MCM1 AND MCM1'-MCM1 THEN 000 OR 001 OR 010 OR 100 OR 101 OR 110 OR 111
110: 7.195528e-07 (1644,51,115,15)

IF SFF'-FKH1 AND MCM1-MCM1 THEN 000 OR 010 OR 100 OR 110 OR 111
110: 9.533178e-07 (1644,14,115,8)

IF MCM1'-NDD1 AND ECB-FKH2 AND ECB-NDD1 THEN 000 OR 010 OR 110 OR 111
110: 9.533178e-07 (1644,14,115,8)

IF MCM1'-NDD1 AND ECB-FKH2 THEN 000 OR 010 OR 110 OR 111
110: 9.533178e-07 (1644,14,115,8)

IF MCM1-FKH2 AND MCM1-MCM1 THEN 000 OR 010 OR 100 OR 110 OR 111
110: 9.533178e-07 (1644,14,115,8)

IF MCM1'-NDD1 AND SFF-FKH2 THEN 000 OR 001 OR 010 OR 100 OR 110 OR 111
110: 1.008355e-06 (1644,23,115,10)

IF SFF'-MCM1 AND MCM1'-NDD1 THEN 000 OR 010 OR 100 OR 110 OR 111
110: 1.506057e-06 (1644,19,115,9)

IF MCM1'-MCM1 AND ECB-FKH2 THEN 000 OR 010 OR 110 OR 111
110: 1.792974e-06 (1644,11,115,7)

IF MCM1-MCM1 AND ECB-YOX1 THEN 000 OR 010 OR 110 OR 111
110: 1.792974e-06 (1644,11,115,7)

IF ECB-MCM1 AND ECB-YOX1 THEN 000 OR 010 OR 110 OR 111
110: 1.792974e-06 (1644,11,115,7)

IF MCM1-YOX1 AND ECB-MCM1 THEN 000 OR 010 OR 110 OR 111
110: 1.792974e-06 (1644,11,115,7)

IF MCM1'-NDD1 AND ECB-FKH2 AND SFF-FKH2 THEN 000 OR 010 OR 110 OR 111
110: 1.792974e-06 (1644,11,115,7)

IF SFF'-MCM1 AND MCM1-YOX1 THEN 000 OR 010 OR 110 OR 111
110: 1.792974e-06 (1644,11,115,7)

IF MCM1'-MCM1 AND ECB-YOX1 THEN 000 OR 010 OR 110 OR 111
110: 1.792974e-06 (1644,11,115,7)

IF SFF'-MCM1 AND MCM1-NDD1 THEN 000 OR 010 OR 100 OR 110 OR 111
110: 1.924239e-06 (1644,15,115,8)

IF SFF'-FKH2 AND MCM1-NDD1 THEN 000 OR 010 OR 100 OR 110 OR 111
110: 1.924239e-06 (1644,15,115,8)

IF ECB-MCM1 AND ECB-NDD1 THEN 000 OR 010 OR 110 OR 111
110: 1.924239e-06 (1644,15,115,8)

IF MCM1-MCM1 AND MCM1'-NDD1 THEN 000 OR 010 OR 100 OR 110 OR 111
110: 1.924239e-06 (1644,15,115,8)

IF ECB-YOX1 THEN 000 OR 010 OR 110 OR 111
110: 1.924239e-06 (1644,15,115,8)

IF MCM1-FKH2 AND MCM1'-NDD1 THEN 000 OR 010 OR 100 OR 110 OR 111
110: 1.924239e-06 (1644,15,115,8)

IF MCM1'-MCM1 AND SFF-FKH2 THEN 000 OR 100 OR 110 OR 111
110: 1.924239e-06 (1644,15,115,8)

IF SWI5-SWI6 THEN 000 OR 001 OR 010 OR 011 OR 100 OR 101 OR 110 OR 111
110: 2.112642e-06 (1644,55,115,15)

IF SFF'-FKH2 AND MCM1'-NDD1 THEN 000 OR 001 OR 010 OR 100 OR 110 OR 111
110: 2.554222e-06 (1644,25,115,10)

IF SFF'-FKH2 AND MCM1'-MCM1 THEN 000 OR 010 OR 100 OR 110 OR 111
110: 3.625139e-06 (1644,16,115,8)

IF ECB-FKH2 AND ECB-NDD1 THEN 000 OR 010 OR 110 OR 111
110: 3.625139e-06 (1644,16,115,8)

IF MCM1-YOX1 THEN 000 OR 010 OR 110 OR 111
110: 3.625139e-06 (1644,16,115,8)

IF MCM1-MCM1 AND m_other_morphogenetic_activities_n7-MCM1 THEN 000 OR 010 OR 100 OR 110
110: 4.055178e-06 (1644,12,115,7)

IF MCM1-NDD1 AND MCM1'-NDD1 AND SFF-FKH2 THEN 000 OR 100 OR 110 OR 111
110: 4.055178e-06 (1644,12,115,7)

IF MCM1-NDD1 AND SFF-FKH2 THEN 000 OR 100 OR 110 OR 111
110: 4.055178e-06 (1644,12,115,7)

IF ECB-NDD1 AND SFF-FKH2 THEN 000 OR 010 OR 110 OR 111
110: 4.055178e-06 (1644,12,115,7)

IF MCM1-FKH2 AND MCM1'-NDD1 AND SFF-FKH2 THEN 000 OR 100 OR 110 OR 111
110: 4.055178e-06 (1644,12,115,7)

IF SFF'-FKH1 AND MCM1'-MCM1 THEN 000 OR 010 OR 100 OR 110 OR 111
110: 6.450221e-06 (1644,17,115,8)

IF SFF'-FKH2 AND MCM1'-NDD1 AND SFF-FKH2 THEN 000 OR 001 OR 010 OR 100 OR 110 OR 111
110: 6.772743e-06 (1644,22,115,9)

IF SFF'-FKH1 AND SFF'-MCM1 THEN 000 OR 010 OR 100 OR 110 OR 111
110: 6.772743e-06 (1644,22,115,9)

IF m_pheromone_response_generation_n12-FKH1 THEN 000 OR 010 OR 100 OR 110
110: 7.294416e-06 (1644,9,115,6)

IF SFF'-MCM1 AND MCM1-FKH2 THEN 000 OR 010 OR 100 OR 110 OR 111
110: 8.280177e-06 (1644,13,115,7)

IF ECB-FKH2 AND ECB-MCM1 THEN 000 OR 010 OR 110 OR 111
110: 8.280177e-06 (1644,13,115,7)

IF SFF'-FKH2 AND MCM1'-NDD1 AND ECB-NDD1 THEN 000 OR 010 OR 110 OR 111
110: 8.280177e-06 (1644,13,115,7)

IF SFF'-MCM1 AND ECB-NDD1 THEN 000 OR 010 OR 110 OR 111
110: 8.280177e-06 (1644,13,115,7)

IF MCM1-FKH2 AND MCM1'-MCM1 THEN 000 OR 010 OR 100 OR 110 OR 111
110: 8.280177e-06 (1644,13,115,7)

IF SFF'-FKH2 AND MCM1-MCM1 THEN 000 OR 010 OR 100 OR 110 OR 111
110: 8.280177e-06 (1644,13,115,7)

IF ECB-STE12 AND ECB-TEC1 THEN 000 OR 110
110: 8.740039e-06 (1644,6,115,5)

IF SFF'-FKH2 AND SFF'-MCM1 THEN 000 OR 010 OR 011 OR 100 OR 110 OR 111
110: 0.00001 (1644,23,115,9)

IF MCM1-FKH2 THEN 000 OR 010 OR 100 OR 110 OR 111
110: 0.00001 (1644,23,115,9)

IF SCB-SWI4 AND m_deoxyribonucleotide_metabolism_n10-SWI6 THEN 000 OR 110 OR 111
111: 0.000013 (1644,5,19,3)

IF SFF'-FKH2 AND ECB-FKH2 AND ECB-NDD1 THEN 000 OR 010 OR 110 OR 111
110: 0.000016 (1644,14,115,7)

IF SFF'-FKH2 AND ECB-NDD1 THEN 000 OR 010 OR 110 OR 111
110: 0.000016 (1644,14,115,7)

IF MCM1-NDD1 AND m_other_morphogenetic_activities_n7-MCM1 THEN 000 OR 010 OR 100 OR 110
110: 0.000017 (1644,10,115,6)

IF ECB-MCM1 AND SFF-FKH2 THEN 000 OR 110 OR 111
110: 0.000017 (1644,10,115,6)

IF SFF'-FKH1 AND ECB-MCM1 THEN 000 OR 010 OR 110 OR 111
110: 0.000017 (1644,10,115,6)

IF SFF'-MCM1 AND ECB-YOX1 THEN 000 OR 010 OR 110 OR 111
110: 0.000017 (1644,10,115,6)

IF SFF'-FKH2 AND MCM1'-NDD1 AND ECB-NDD1 AND SFF-FKH2 THEN 000 OR 010 OR 110 OR 111
110: 0.000017 (1644,10,115,6)

IF MCM1'-SWI4 AND ECB-TEC1 THEN 110
110: 0.000023 (1644,4,115,4)

IF MCM1'-SWI4 AND ECB-STE12 THEN 110
110: 0.000023 (1644,4,115,4)

IF ECB-FKH2 THEN 000 OR 010 OR 110 OR 111
110: 0.000028 (1644,20,115,8)

IF ECB-FKH2 AND SFF-FKH2 THEN 000 OR 010 OR 110 OR 111
110: 0.000028 (1644,15,115,7)

IF SFF'-FKH1 AND SFF'-MCM1 AND MCM1-YOX1 THEN 010 OR 110 OR 111
110: 0.000029 (1644,7,115,5)

IF SFF'-FKH1 AND LYS14-SWI6 THEN 000 OR 110
110: 0.000029 (1644,7,115,5)

IF SFF'-FKH1 AND MCM1-YOX1 THEN 010 OR 110 OR 111
110: 0.000029 (1644,7,115,5)

IF MCM1-NDD1 AND ECB-FKH2 AND ECB-NDD1 THEN 000 OR 010 OR 110 OR 111
110: 0.000036 (1644,11,115,6)

IF SFF'-FKH2 AND MCM1-NDD1 AND SFF-FKH2 THEN 000 OR 100 OR 110 OR 111
110: 0.000036 (1644,11,115,6)

IF MCM1-FKH2 AND MCM1-NDD1 AND ECB-FKH2 THEN 000 OR 010 OR 110 OR 111
110: 0.000036 (1644,11,115,6)

IF MCM1-MCM1 AND SFF-FKH2 THEN 000 OR 100 OR 110 OR 111
110: 0.000036 (1644,11,115,6)

IF SFF'-FKH2 AND ECB-MCM1 THEN 000 OR 010 OR 110 OR 111
110: 0.000036 (1644,11,115,6)

IF MCM1-MCM1 AND ECB-NDD1 THEN 000 OR 010 OR 110 OR 111
110: 0.000036 (1644,11,115,6)

IF MCM1-NDD1 AND ECB-MCM1 THEN 000 OR 010 OR 110 OR 111
110: 0.000036 (1644,11,115,6)

IF SFF'-MCM1 AND ECB-FKH2 THEN 000 OR 010 OR 110 OR 111
110: 0.000036 (1644,11,115,6)

IF MCM1-FKH2 AND ECB-NDD1 THEN 000 OR 010 OR 110 OR 111
110: 0.000036 (1644,11,115,6)

IF MCM1-FKH2 AND MCM1'-NDD1 AND ECB-NDD1 THEN 000 OR 010 OR 110 OR 111
110: 0.000036 (1644,11,115,6)

IF MCM1-NDD1 AND ECB-FKH2 THEN 000 OR 010 OR 110 OR 111

110: 0.000036 (1644,11,115,6)

IF LYS14-SWI6 THEN 000 OR 010 OR 100 OR 110
110: 0.000043 (1644,21,115,8)

IF MCB-MBP1 THEN 000 OR 010 OR 011 OR 100 OR 101 OR 110 OR 111
110: 0.000063 (1644,22,115,8)

IF MCM1-FKH2 AND m_other_morphogenetic_activities_n7-MCM1 THEN 000 OR 010 OR 100 OR 110
110: 0.000073 (1644,8,115,5)

IF SFF'-FKH2 AND LYS14-SWI6 THEN 000 OR 110
110: 0.000073 (1644,8,115,5)

IF MCM1-MCM1 AND ECB-MCM1 AND m_other_morphogenetic_activities_n7-MCM1 THEN 000 OR 010 OR 110
110: 0.000073 (1644,8,115,5)

IF SFF'-MCM1 AND ECB-MCM1 AND m_other_morphogenetic_activities_n7-MCM1 THEN 000 OR 010 OR 110
110: 0.000073 (1644,8,115,5)

IF MCM1-MCM1 AND MCM1-SWI4 THEN 000 OR 100 OR 110
110: 0.000073 (1644,8,115,5)

IF MCM1-NDD1 AND ECB-NDD1 AND SFF-FKH2 THEN 000 OR 110 OR 111
110: 0.000073 (1644,8,115,5)

IF MCM1'-SWI4 AND ECB-MCM1 THEN 000 OR 100 OR 110
110: 0.000073 (1644,8,115,5)

IF MCM1-SWI4 THEN 000 OR 010 OR 100 OR 110 OR 111
110: 0.000074 (1644,17,115,7)

IF SFF'-FKH2 AND ECB-FKH2 THEN 000 OR 010 OR 110 OR 111
110: 0.000074 (1644,17,115,7)

IF MCM1-FKH2 AND SFF-FKH2 THEN 000 OR 100 OR 110 OR 111
110: 0.000074 (1644,17,115,7)

IF MCM1'-SWI4 AND m_nutritional_response_pathway_n7-SWI6 THEN 000 OR 101 OR 110 OR 111
111: 0.000105 (1644,9,19,3)

IF SFF'-FKH2 AND m_nucleotide_transport_n9-MCM1 THEN 010 OR 110
110: 0.000108 (1644,5,115,4)

IF m_breakdown_of_lipids_fatty_acids_and_isoprenoids_n8-SWI6 AND SWI5-SWI6 THEN 110 OR 111
110: 0.000108 (1644,5,115,4)

IF MCM1'-NDD1 AND SFF-FKH1 THEN 000 OR 010 OR 100 OR 110 OR 111
110: 0.000118 (1644,13,115,6)

IF m_breakdown_of_lipids_fatty_acids_and_isoprenoids_n8-MBP1 THEN 000 OR 001 OR 100 OR 110 OR 111
110: 0.000118 (1644,13,115,6)

IF MCM1'-NDD1 AND m_other_morphogenetic_activities_n7-MCM1 THEN 000 OR 010 OR 100 OR 110
110: 0.000118 (1644,13,115,6)

IF MCM1-SWI4 AND MCM1'-SWI4 THEN 000 OR 010 OR 100 OR 110 OR 111
110: 0.000118 (1644,13,115,6)

IF m_nutritional_response_pathway_n7-SWI6 AND m_glycolysis_and_gluconeogenesis_n27-SWI4 THEN 111
111: 0.000127 (1644,2,19,2)

IF m_ion_transporters_n11-SWI4 AND m_phosphate_transport_n18-XBP1 THEN 111
111: 0.000127 (1644,2,19,2)

IF mPROTEOL18(m_proteolysis_n18)-SWI6 AND m_nutritional_response_pathway_n7-SWI4 THEN 111
111: 0.000127 (1644,2,19,2)

IF m_allantoin_and_allantoate_transporters_n7-ACE2 AND SFF-FKH1 AND m_deoxyribonucleotide_metabolism_n4-SWI6 THEN 111
111: 0.000127 (1644,2,19,2)

IF SFF'-FKH1 AND SWI5-SWI6 AND m_deoxyribonucleotide_metabolism_n4-SWI6 THEN 111
111: 0.000127 (1644,2,19,2)

IF m_deoxyribonucleotide_metabolism_n10-SWI6 AND MCB-STB1 THEN 111
111: 0.000127 (1644,2,19,2)

IF SFF'-MCM1 AND m_deoxyribonucleotide_metabolism_n4-FKH2 THEN 111
111: 0.000127 (1644,2,19,2)

IF m_ion_transporters_n11-SWI6 AND m_phosphate_transport_n18-XBP1 THEN 111
111: 0.000127 (1644,2,19,2)

IF m_phosphate_transport_n18-XBP1 AND SCB-SWI4 THEN 111
111: 0.000127 (1644,2,19,2)

IF SWI5-SWI6 AND m_deoxyribonucleotide_metabolism_n4-FKH2 THEN 111
111: 0.000127 (1644,2,19,2)

IF SFF'-MCM1 AND m_deoxyribonucleotide_metabolism_n4-SWI6 THEN 111
111: 0.000127 (1644,2,19,2)

IF m_nutritional_response_pathway_n7-SWI4 AND m_phosphate_transport_n18-XBP1 THEN 111
111: 0.000127 (1644,2,19,2)

IF m_metabolism_of_energy_reserves_n27-NDD1 AND SCB-AZF1 THEN 111
111: 0.000127 (1644,2,19,2)

IF MCM1'-SWI4 AND m_glycolysis_and_gluconeogenesis_n27-SWI6 THEN 111
111: 0.000127 (1644,2,19,2)

IF m_glycolysis_and_gluconeogenesis_n27-STB1 THEN 111
111: 0.000127 (1644,2,19,2)

IF SFF'-MCM1 AND m_allantoin_and_allantoate_transporters_n7-ACE2 THEN 111
111: 0.000127 (1644,2,19,2)

IF m_allantoin_and_allantoate_transporters_n7-ACE2 AND m_deoxyribonucleotide_metabolism_n4-FKH2 THEN 111
111: 0.000127 (1644,2,19,2)

IF mPROTEOL18(m_proteolysis_n18)-SWI6 AND m_nutritional_response_pathway_n7-SWI6 AND SCB-SWI4 THEN 111
111: 0.000127 (1644,2,19,2)

IF MCM1'-SWI4 AND mPROTEOL18(m_proteolysis_n18)-SWI6 AND m_nutritional_response_pathway_n7-SWI6 THEN 111
111: 0.000127 (1644,2,19,2)

IF m_ion_transporters_n11-SWI6 AND mPROTEOL18(m_proteolysis_n18)-SWI6 AND m_nutritional_response_pathway_n7-SWI6 THEN 111
111: 0.000127 (1644,2,19,2)

IF m_nutritional_response_pathway_n7-SWI6 AND m_glycolysis_and_gluconeogenesis_n27-SWI6 THEN 111
111: 0.000127 (1644,2,19,2)

IF m_nutritional_response_pathway_n7-SWI4 AND m_glycolysis_and_gluconeogenesis_n27-SWI6 THEN 111
111: 0.000127 (1644,2,19,2)

IF m_nutritional_response_pathway_n7-SWI6 AND m_phosphate_transport_n18-XBP1 THEN 111
111: 0.000127 (1644,2,19,2)

IF m_deoxyribonucleotide_metabolism_n10-SWI6 THEN 000 OR 001 OR 110 OR 111
111: 0.000139 (1644,25,19,4)

IF MCM1-YOX1 AND MCM1'-NDD1 THEN 000 OR 010 OR 110 OR 111
110: 0.000155 (1644,9,115,5)

IF ECB-DIG1 THEN 000 OR 100 OR 110
110: 0.000155 (1644,9,115,5)

IF MCM1-NDD1 AND MCM1-YOX1 THEN 000 OR 010 OR 110 OR 111
110: 0.000155 (1644,9,115,5)

IF ECB-TEC1 THEN 000 OR 010 OR 100 OR 110
110: 0.000155 (1644,9,115,5)

IF MCM1-MCM1 AND MCM1'-NDD1 AND ECB-FKH2 THEN 000 OR 010 OR 110 OR 111
110: 0.000155 (1644,9,115,5)

IF ECB-MCM1 AND m_other_morphogenetic_activities_n7-MCM1 THEN 000 OR 010 OR 110
110: 0.000155 (1644,9,115,5)

IF MCM1-MCM1 AND ECB-FKH2 THEN 000 OR 010 OR 110 OR 111
110: 0.000155 (1644,9,115,5)

IF MCM1-FKH2 AND ECB-MCM1 THEN 000 OR 010 OR 110 OR 111
110: 0.000155 (1644,9,115,5)

IF SFF'-MCM1 THEN 000 OR 001 OR 010 OR 011 OR 100 OR 101 OR 110 OR 111
110: 0.000176 (1644,86,115,16)

IF MCM1'-MCM1 AND SFF-FKH1 THEN 000 OR 010 OR 100 OR 110 OR 111
110: 0.000195 (1644,14,115,6)

IF SFF'-FKH1 AND MCM1'-NDD1 THEN 000 OR 010 OR 100 OR 110 OR 111
110: 0.000195 (1644,14,115,6)

IF m_lipid_and_fatty-acid_binding_n15-STP2 THEN 000 OR 010
010: 0.000236 (1644,5,140,4)

IF SFF'-MCM1 AND SFF-FKH2 THEN 000 OR 010 OR 011 OR 100 OR 110 OR 111
110: 0.000247 (1644,20,115,7)

IF SFF'-FKH2 AND MCM1-NDD1 AND ECB-NDD1 THEN 000 OR 010 OR 110 OR 111
110: 0.000292 (1644,10,115,5)

IF SFF'-FKH1 AND MCM1-NDD1 THEN 000 OR 010 OR 100 OR 110 OR 111
110: 0.000292 (1644,10,115,5)

IF MCM1-MCM1 AND SFF-FKH1 THEN 000 OR 010 OR 100 OR 110 OR 111
110: 0.000292 (1644,10,115,5)

IF m_nucleotide_transport_n9-SWI6 THEN 000 OR 010 OR 100 OR 101 OR 110
110: 0.000306 (1644,15,115,6)

IF ECB-NDD1 AND m_other_morphogenetic_activities_n7-MCM1 THEN 000 OR 010 OR 110
110: 0.000306 (1644,6,115,4)

IF SFF-FKH1 AND LYS14-SWI6 THEN 000 OR 110
110: 0.000306 (1644,6,115,4)

IF MCM1-NDD1 AND ECB-MCM1 AND m_other_morphogenetic_activities_n7-MCM1 THEN 000 OR 010 OR 110
110: 0.000306 (1644,6,115,4)

IF SFF'-FKH1 AND ECB-YOX1 THEN 010 OR 110 OR 111
110: 0.000306 (1644,6,115,4)

IF MCM1-FKH2 AND ECB-FKH2 THEN 000 OR 010 OR 110 OR 111
110: 0.000306 (1644,15,115,6)

IF SFF'-FKH1 AND SFF'-FKH2 AND SFF'-MCM1 THEN 000 OR 010 OR 100 OR 110 OR 111
110: 0.000306 (1644,15,115,6)

IF MCM1-YOX1 AND m_other_morphogenetic_activities_n7-MCM1 THEN 000 OR 010 OR 110
110: 0.000306 (1644,6,115,4)

IF MCM1-SWI4 AND ECB-MCM1 THEN 000 OR 100 OR 110
110: 0.000306 (1644,6,115,4)

IF SFF'-MCM1 AND MCM1-SWI4 THEN 000 OR 100 OR 110
110: 0.000306 (1644,6,115,4)

IF MCM1'-NDD1 AND ECB-MCM1 AND m_other_morphogenetic_activities_n7-MCM1 THEN 000 OR 010 OR 110

110: 0.000306 (1644,6,115,4)

IF MCM1-SWI4 AND ECB-STE12 THEN 110
110: 0.000334 (1644,3,115,3)

IF ECB-TEC1 AND SFF-FKH1 THEN 110
110: 0.000334 (1644,3,115,3)

IF MCM1'-NDD1 AND ECB-TEC1 THEN 110
110: 0.000334 (1644,3,115,3)

IF ECB-STE12 AND SFF-FKH2 THEN 110
110: 0.000334 (1644,3,115,3)

IF MCM1'-NDD1 AND ECB-STE12 THEN 110
110: 0.000334 (1644,3,115,3)

IF MCM1-SWI4 AND ECB-TEC1 THEN 110
110: 0.000334 (1644,3,115,3)

IF m_cytoskeleton-dependenttransport_n4-MBP1 AND m_cytoskeleton-dependenttransport_n4-STB1 THEN 110
110: 0.000334 (1644,3,115,3)

IF ECB-FKH2 AND ECB-TEC1 THEN 110
110: 0.000334 (1644,3,115,3)

IF SWI5-SWI6 AND m_cytoskeleton-dependenttransport_n4-STB1 THEN 110
110: 0.000334 (1644,3,115,3)

IF ECB-TEC1 AND SFF-FKH2 THEN 110
110: 0.000334 (1644,3,115,3)

IF ECB-NDD1 AND ECB-TEC1 THEN 110
110: 0.000334 (1644,3,115,3)

IF m_cytoskeleton-dependenttransport_n4-STB1 AND m_other_transport_facilitators_n10-SWI6 THEN 110
110: 0.000334 (1644,3,115,3)

IF SFF'-FKH1 AND SFF'-FKH2 AND m_osmosensing_n6-ROX1 THEN 110
110: 0.000334 (1644,3,115,3)

IF SFF'-FKH2 AND m_osmosensing_n6-ROX1 AND SFF-FKH1 THEN 110
110: 0.000334 (1644,3,115,3)

IF LYS14-SWI6 AND SCB-SWI4 THEN 110
110: 0.000334 (1644,3,115,3)

IF MCM1'-SWI4 AND ECB-DIG1 THEN 110
110: 0.000334 (1644,3,115,3)

IF SFF'-MCM1 AND SCB-UME6 THEN 110
110: 0.000334 (1644,3,115,3)

IF MCM1-SWI4 AND LYS14-SWI6 THEN 110
110: 0.000334 (1644,3,115,3)

IF m_osmosensing_n6-ROX1 AND SFF-FKH2 THEN 110
110: 0.000334 (1644,3,115,3)

IF ECB-DIG1 AND SFF-FKH1 THEN 110
110: 0.000334 (1644,3,115,3)

IF MCB-MIG2 AND MCB-YFL052w THEN 110
110: 0.000334 (1644,3,115,3)

IF MCM1'-MCM1 AND m_pheromone_response_generation_n12-FKH1 THEN 110
110: 0.000334 (1644,3,115,3)

IF SFF'-FKH2 AND m_cytoskeleton-dependenttransport_n4-STB1 THEN 110
110: 0.000334 (1644,3,115,3)

IF m_pheromone_response_generation_n12-FKH1 AND m_pentose-phosphate_pathway_n14-FKH1 THEN 110
110: 0.000334 (1644,3,115,3)

IF MCM1-MCM1 AND m_pheromone_response_generation_n12-FKH1 THEN 110
110: 0.000334 (1644,3,115,3)

IF m_phosphate_transport_n8-SWI4 AND m_allantoin_and_allantoate_transporters_n12-ACE2 THEN 110
110: 0.000334 (1644,3,115,3)

IF m_other_signal-transduction_activities_n8-INO4 AND m_glyoxylate_cycle_n8-INO4 AND m_other_mrna-transcription_activities_n20-INO4 THEN 110
110: 0.000334 (1644,3,115,3)

IF MCM1-SWI4 AND ECB-DIG1 THEN 110
110: 0.000334 (1644,3,115,3)

IF MCM1-YOX1 AND m_pheromone_response_generation_n12-FKH1 THEN 110
110: 0.000334 (1644,3,115,3)

IF SFF-FKH2 AND m_cytoskeleton-dependenttransport_n4-STB1 THEN 110
110: 0.000334 (1644,3,115,3)

IF MCB-MIG2 AND MCB-YNR063W THEN 110
110: 0.000334 (1644,3,115,3)

IF m_cytoskeleton-dependenttransport_n4-MBP1 AND m_other_transport_facilitators_n10-SWI6 THEN 110
110: 0.000334 (1644,3,115,3)

IF SFF'-MCM1 AND m_pheromone_response_generation_n12-FKH1 THEN 110
110: 0.000334 (1644,3,115,3)

IF SFF'-FKH2 AND m_nucleotide_transport_n9-SWI6 THEN 110
110: 0.000334 (1644,3,115,3)

IF m_breakdown_of_lipids_fatty_acids_and_isoprenoids_n8-MBP1 AND MCB-MBP1 THEN 110
110: 0.000334 (1644,3,115,3)

IF MCM1-MCM1 AND SCB-UME6 THEN 110
110: 0.000334 (1644,3,115,3)

IF m_nutritional_response_pathway_n7-SWI4 AND m_nutritional_response_pathway_n7-SWI6 THEN 000 OR 101 OR 110 OR 111
111: 0.000348 (1644,13,19,3)

IF MCM1'-SWI4 AND m_nutritional_response_pathway_n7-SWI4 THEN 000 OR 100 OR 101 OR 110 OR 111
111: 0.000348 (1644,13,19,3)

IF m_ion_transporters_n11-SWI6 AND mPROTEOL18(m_proteolysis_n18)-SWI6 AND SCB-SWI4 THEN 000 OR 111
111: 0.000377 (1644,3,19,2)

IF m_nutritional_response_pathway_n7-SWI4 AND m_deoxyribonucleotide_metabolism_n10-SWI6 THEN 000 OR 111
111: 0.000377 (1644,3,19,2)

IF SFF'-FKH1 AND m_allantoin_and_allantoate_transporters_n7-ACE2 AND SWI5-SWI6 THEN 000 OR 111
111: 0.000377 (1644,3,19,2)

IF m_allantoin_and_allantoate_transporters_n7-ACE2 AND SFF-FKH1 AND SWI5-SWI6 THEN 000 OR 111
111: 0.000377 (1644,3,19,2)

IF m_allantoin_and_allantoate_transporters_n7-ACE2 AND m_deoxyribonucleotide_metabolism_n4-SWI6 THEN 110 OR 111
111: 0.000377 (1644,3,19,2)

IF MCM1'-SWI4 AND m_ion_transporters_n11-SWI6 AND SCB-SWI4 THEN 000 OR 111
111: 0.000377 (1644,3,19,2)

IF MCM1'-SWI4 AND m_ion_transporters_n11-SWI6 AND m_nutritional_response_pathway_n7-SWI4 THEN 000 OR 111
111: 0.000377 (1644,3,19,2)

IF SFF'-FKH1 AND SFF'-MCM1 AND SWI5-SWI6 THEN 100 OR 111
111: 0.000377 (1644,3,19,2)

IF m_deoxyribonucleotide_metabolism_n10-SWI6 AND MCB-SWI6 THEN 110 OR 111
111: 0.000377 (1644,3,19,2)

IF mPROTEOL18(m_proteolysis_n18)-SWI6 AND m_nutritional_response_pathway_n7-SWI6 THEN 000 OR 111
111: 0.000377 (1644,3,19,2)

IF m_metabolism_of_energy_reserves_n27-SKN7 AND SCB-AZF1 THEN 000 OR 111
111: 0.000377 (1644,3,19,2)

IF SWI5-SWI6 AND m_deoxyribonucleotide_metabolism_n4-SWI6 THEN 110 OR 111
111: 0.000377 (1644,3,19,2)

IF MCM1'-SWI4 AND m_phosphate_transport_n18-XBP1 THEN 000 OR 111
111: 0.000377 (1644,3,19,2)

IF MCM1'-SWI4 AND m_nutritional_response_pathway_n7-SWI6 AND m_deoxyribonucleotide_metabolism_n10-SWI6 THEN 000 OR 111
111: 0.000377 (1644,3,19,2)

IF MCM1'-SWI4 AND m_ion_transporters_n11-SWI4 AND m_nutritional_response_pathway_n7-SWI4 THEN 000 OR 111
111: 0.000377 (1644,3,19,2)

IF SFF'-FKH2 AND m_metabolism_of_energy_reserves_n27-SKN7 THEN 000 OR 111
111: 0.000377 (1644,3,19,2)

IF mPROTEOL18(m_proteolysis_n18)-SWI6 AND m_phosphate_transport_n18-XBP1 THEN 000 OR 111
111: 0.000377 (1644,3,19,2)

IF m_phosphate_transport_n18-INO4 AND m_amino-acid_transporters_n11-SKN7 THEN 000 OR 111
111: 0.000377 (1644,3,19,2)

IF m_phosphate_transport_n18-INO4 AND m_amino-acid_transporters_n11-SWI4 THEN 010 OR 111
111: 0.000377 (1644,3,19,2)

IF ECB-STE12 THEN 000 OR 001 OR 010 OR 100 OR 110
110: 0.000505 (1644,11,115,5)

IF MCM1'-MCM1 AND SFF-FKH1 AND SFF-FKH2 THEN 000 OR 100 OR 110 OR 111
110: 0.000505 (1644,11,115,5)

IF MCM1'-SWI4 THEN 000 OR 010 OR 100 OR 101 OR 110 OR 111
110: 0.000633 (1644,52,115,11)

IF m_other_mrna-transcription_activities_n20-RAP1 AND m_pentose-phosphate_pathway_n23-RAP1 THEN 000 OR 001 OR 110
001: 0.00066 (1644,6,55,3)

IF MCM1-MCM1 AND MCM1'-SWI4 THEN 000 OR 100 OR 110
110: 0.000675 (1644,7,115,4)

IF SFF-FKH2 AND LYS14-SWI6 THEN 000 OR 110
110: 0.000675 (1644,7,115,4)

IF ECB-MCM1 AND SFF-FKH1 THEN 000 OR 010 OR 110 OR 111
110: 0.000675 (1644,7,115,4)

IF m_osmosensing_n6-ROX1 AND SFF-FKH1 THEN 000 OR 010 OR 110
110: 0.000675 (1644,7,115,4)

IF SFF'-FKH1 AND m_osmosensing_n6-ROX1 THEN 000 OR 010 OR 110
110: 0.000675 (1644,7,115,4)

IF m_glyoxylate_cycle_n19-SWI6 THEN 000 OR 110
110: 0.000675 (1644,7,115,4)

IF MCM1-SWI4 AND MCM1'-MCM1 THEN 000 OR 100 OR 110
110: 0.000675 (1644,7,115,4)

IF SWI5-SWI6 AND m_nucleotide_transport_n9-SWI6 THEN 000 OR 101 OR 110
110: 0.000675 (1644,7,115,4)

IF MCM1-SWI4 AND MCM1'-MCM1 AND MCM1'-SWI4 THEN 000 OR 100 OR 110
110: 0.000675 (1644,7,115,4)

IF m_phosphate_transport_n18-YAP6 AND m_LFTE17-SKN7 THEN 000 OR 111
111: 0.000749 (1644,4,19,2)

IF m_ion_transporters_n11-SKN7 AND m_phosphate_transport_n18-YAP6 THEN 000 OR 111
111: 0.000749 (1644,4,19,2)

IF m_phosphate_transport_n18-YAP6 AND SCB-AZF1 THEN 000 OR 011 OR 111
111: 0.000749 (1644,4,19,2)

IF MCM1'-SWI4 AND m_ion_transporters_n11-SWI6 AND mPROTEOL18(m_proteolysis_n18)-SWI6 THEN 000 OR 111
111: 0.000749 (1644,4,19,2)

IF SFF'-FKH2 AND SCB-AZF1 THEN 010 OR 110 OR 111
111: 0.000749 (1644,4,19,2)

IF m_ion_transporters_n11-SKN7 AND SCB-AZF1 THEN 000 OR 111
111: 0.000749 (1644,4,19,2)

IF m_deoxyribonucleotide_metabolism_n4-FKH2 AND m_deoxyribonucleotide_metabolism_n4-SWI6 THEN 000 OR 111
111: 0.000749 (1644,4,19,2)

IF m_ion_transporters_n11-SWI6 AND m_nutritional_response_pathway_n7-SWI6 AND SCB-SWI4 THEN 000 OR 111
111: 0.000749 (1644,4,19,2)

IF m_ion_transporters_n11-SWI4 AND m_nutritional_response_pathway_n7-SWI6 AND SCB-SWI4 THEN 000 OR 111
111: 0.000749 (1644,4,19,2)

IF m_ion_transporters_n11-SWI4 AND mPROTEOL18(m_proteolysis_n18)-SWI6 THEN 000 OR 111
111: 0.000749 (1644,4,19,2)

IF SFF-FKH2 AND m_deoxyribonucleotide_metabolism_n4-SWI6 THEN 000 OR 111
111: 0.000749 (1644,4,19,2)

IF SFF-FKH2 AND m_deoxyribonucleotide_metabolism_n4-FKH2 THEN 000 OR 111
111: 0.000749 (1644,4,19,2)

IF SFF'-FKH2 AND m_deoxyribonucleotide_metabolism_n4-SWI6 THEN 000 OR 111
111: 0.000749 (1644,4,19,2)

IF m_g-proteins_n12-SKN7 AND SWI5-SKN7 AND SCB-AZF1 THEN 010 OR 111
111: 0.000749 (1644,4,19,2)

IF SFF-FKH1 AND m_deoxyribonucleotide_metabolism_n4-FKH2 THEN 000 OR 111
111: 0.000749 (1644,4,19,2)

IF MCB-STB1 THEN 000 OR 110 OR 111
111: 0.000749 (1644,4,19,2)

IF m_glycolysis_and_gluconeogenesis_n27-SWI4 AND m_glycolysis_and_gluconeogenesis_n27-SWI6 THEN 000 OR 010 OR 111
111: 0.000749 (1644,4,19,2)

IF SFF'-FKH1 AND m_deoxyribonucleotide_metabolism_n4-FKH2 THEN 000 OR 111
111: 0.000749 (1644,4,19,2)

IF m_phosphate_transport_n18-INO4 AND SCB-SWI4 THEN 000 OR 010 OR 111
111: 0.000749 (1644,4,19,2)

IF SFF'-FKH2 AND m_deoxyribonucleotide_metabolism_n4-FKH2 THEN 000 OR 111
111: 0.000749 (1644,4,19,2)

IF SFF'-FKH1 AND SFF'-FKH2 AND MCM1'-MCM1 THEN 000 OR 010 OR 100 OR 110 OR 111
110: 0.000818 (1644,12,115,5)

IF SFF'-FKH1 AND MCM1'-NDD1 AND SFF-FKH2 THEN 000 OR 010 OR 100 OR 110 OR 111
110: 0.000818 (1644,12,115,5)

IF SFF'-FKH2 AND m_other_morphogenetic_activities_n7-MCM1 THEN 000 OR 010 OR 011 OR 100 OR 110 OR 111

110: 0.000818 (1644,12,115,5)

IF SFF'-FKH1 AND SFF'-FKH2 AND SFF'-MCM1 AND MCM1'-MCM1 THEN 000 OR 010 OR 100 OR 110 OR 111
110: 0.000818 (1644,12,115,5)

IF MCM1'-MCM1 AND MCM1'-SWI4 THEN 000 OR 100 OR 101 OR 110
110: 0.000818 (1644,12,115,5)

IF m_allantoin_and_allantoate_transporters_n13-SWI6 THEN 000 OR 011 OR 100 OR 101 OR 110 OR 111
110: 0.000953 (1644,18,115,6)

IF m_other_mrna-transcription_activities_n20-RAP1 AND m_RPE32-YAP5 THEN 001
001: 0.0011 (1644,2,55,2)

IF m_phosphate_transport_n18-GAT1 AND HAP234-HAP4 THEN 001
001: 0.0011 (1644,2,55,2)

IF m_other_mrna-transcription_activities_n20-RAP1 AND m_RPE32-FHL1 THEN 001
001: 0.0011 (1644,2,55,2)

IF m_RPE32-YAP5 AND m_amino-acid_transport_n20-RAP1 THEN 001
001: 0.0011 (1644,2,55,2)

IF m_organization_of_chromosome_structure_n17-INO4 AND m_other_mrna-transcription_activities_n20-RAP1 THEN 001
001: 0.0011 (1644,2,55,2)

IF m_other_energy_generation_activities_n4-UME6 AND SWI5-SKN7 THEN 001
001: 0.0011 (1644,2,55,2)

IF m_RPE68-RAP1 AND m_pentose-phosphate_pathway_n23-RAP1 THEN 001
001: 0.0011 (1644,2,55,2)

IF m_other_energy_generation_activities_n4-UME6 AND m_anion_transporters_n22-SKN7 THEN 001
001: 0.0011 (1644,2,55,2)

IF m_pentose-phosphate_pathway_n23-INO4 AND m_nucleotide_transport_n9-INO4 THEN 001
001: 0.0011 (1644,2,55,2)

IF m_other_energy_generation_activities_n4-UME6 AND m_anion_transporters_n20-RAP1 THEN 001
001: 0.0011 (1644,2,55,2)

IF Ume6(URS1)-UME6 AND m_anion_transporters_n22-RAP1 THEN 001
001: 0.0011 (1644,2,55,2)

IF m_other_energy_generation_activities_n4-UME6 AND m_anion_transporters_n22-RAP1 THEN 001
001: 0.0011 (1644,2,55,2)

IF Ume6(URS1)-UME6 AND SWI5-SKN7 THEN 001
001: 0.0011 (1644,2,55,2)

IF m_anion_transporters_n22-SKN7 AND m_meiosis_n3-UME6 THEN 001
001: 0.0011 (1644,2,55,2)

IF m_other_mrna-transcription_activities_n20-RAP1 AND m_RPE68-PDR1 THEN 001
001: 0.0011 (1644,2,55,2)

IF OAF1-SFP1 AND m_other_mrna-transcription_activities_n20-RAP1 THEN 001
001: 0.0011 (1644,2,55,2)

IF m_organization_of_chromosome_structure_n17-INO4 AND m_other_mrna-transcription_activities_n20-INO4 THEN 001
001: 0.0011 (1644,2,55,2)

IF m_RPE68-FHL1 AND m_pentose-phosphate_pathway_n23-RAP1 THEN 001
001: 0.0011 (1644,2,55,2)

IF m_RPE68-PDR1 AND m_pentose-phosphate_pathway_n23-RAP1 THEN 001
001: 0.0011 (1644,2,55,2)

IF m_RPE32-FHL1 AND m_amino-acid_transport_n20-RAP1 THEN 001
001: 0.0011 (1644,2,55,2)

IF Ume6(URS1)-UME6 AND m_anion_transporters_n20-RAP1 THEN 001
001: 0.0011 (1644,2,55,2)

IF Ume6(URS1)-UME6 AND m_anion_transporters_n22-SKN7 THEN 001
001: 0.0011 (1644,2,55,2)

IF PDR-INO4 AND m_nucleotide_transport_n9-INO4 THEN 000 OR 001
001: 0.001128 (1644,7,55,3)

IF m_nutritional_response_pathway_n7-SWI4 THEN 000 OR 100 OR 101 OR 110 OR 111
111: 0.00113 (1644,19,19,3)

IF m_ion_transporters_n11-SWI6 AND mPROTEOL18(m_proteolysis_n18)-SWI6 THEN 000 OR 100 OR 111
111: 0.00124 (1644,5,19,2)

IF m_ion_transporters_n11-SWI6 AND SCB-SWI4 THEN 000 OR 111
111: 0.00124 (1644,5,19,2)

IF SFF'-FKH2 AND m_metabolism_of_energy_reserves_n27-NDD1 THEN 000 OR 111
111: 0.00124 (1644,5,19,2)

IF m_ion_transporters_n11-SWI4 AND m_nutritional_response_pathway_n7-SWI4 AND SCB-SWI4 THEN 000 OR 111
111: 0.00124 (1644,5,19,2)

IF MCM1'-SWI4 AND m_amino-acid_transporters_n11-SWI4 AND SCB-SWI4 THEN 000 OR 010 OR 111
111: 0.00124 (1644,5,19,2)

IF m_allantoin_and_allantoate_transporters_n7-ACE2 AND SFF-FKH1 THEN 000 OR 110 OR 111
111: 0.00124 (1644,5,19,2)

IF MCM1'-SWI4 AND m_ion_transporters_n11-SWI4 AND SCB-SWI4 THEN 000 OR 111
111: 0.00124 (1644,5,19,2)

IF m_nutritional_response_pathway_n7-SWI4 AND m_glycolysis_and_gluconeogenesis_n27-SWI4 THEN 000 OR 111
111: 0.00124 (1644,5,19,2)

IF MCM1'-SWI4 AND m_ion_transporters_n11-SWI6 THEN 000 OR 111
111: 0.00124 (1644,5,19,2)

IF m_metabolism_of_energy_reserves_n27-NDD1 AND SWI5-SKN7 THEN 000 OR 111
111: 0.00124 (1644,5,19,2)

IF MCM1'-SWI4 AND m_glycolysis_and_gluconeogenesis_n27-SWI4 THEN 000 OR 111
111: 0.00124 (1644,5,19,2)

IF MCM1'-SWI4 AND m_phosphate_transport_n18-INO4 THEN 000 OR 010 OR 100 OR 111
111: 0.00124 (1644,5,19,2)

IF m_deoxyribonucleotide_metabolism_n4-FKH2 THEN 000 OR 111
111: 0.00124 (1644,5,19,2)

IF m_LFTE17-SKN7 AND SCB-AZF1 THEN 000 OR 111
111: 0.00124 (1644,5,19,2)

IF m_nutritional_response_pathway_n7-SWI6 AND SCB-SWI4 THEN 000 OR 111
111: 0.00124 (1644,5,19,2)

IF m_metabolism_of_energy_reserves_n27-NDD1 AND m_metabolism_of_energy_reserves_n27-SKN7 AND SWI5-SKN7 THEN 000 OR 111
111: 0.00124 (1644,5,19,2)

IF SFF'-FKH1 AND MCM1-FKH2 THEN 000 OR 010 OR 100 OR 110 OR 111
110: 0.001256 (1644,13,115,5)

IF SFF'-MCM1 AND ECB-FKH2 AND m_nucleotide_transport_n9-MCM1 THEN 010 OR 110
110: 0.001268 (1644,4,115,3)

IF SWI5-SKN7 AND SWI5-SWI6 AND m_nucleotide_transport_n9-SWI6 THEN 000 OR 110
110: 0.001268 (1644,4,115,3)

IF ECB-FKH2 AND m_other_morphogenetic_activities_n7-MCM1 THEN 010 OR 110
110: 0.001268 (1644,4,115,3)

IF LYS14-SWI6 AND SCB-UME6 THEN 010 OR 110
110: 0.001268 (1644,4,115,3)

IF SFF'-RLM1 AND ECB-MCM1 THEN 110 OR 111
110: 0.001268 (1644,4,115,3)

IF MCM1'-SWI4 AND LYS14-SWI6 THEN 000 OR 110
110: 0.001268 (1644,4,115,3)

IF MCM1-FKH2 AND m_nucleotide_transport_n9-MCM1 THEN 010 OR 110
110: 0.001268 (1644,4,115,3)

IF MCM1-NDD1 AND m_nucleotide_transport_n9-MCM1 THEN 010 OR 110
110: 0.001268 (1644,4,115,3)

IF m_lipid_and_fatty-acid_transport_n11-GAT1 AND m_OCSE15-MTH1 THEN 000 OR 110
110: 0.001268 (1644,4,115,3)

IF SWI5-SKN7 AND m_nucleotide_transport_n9-SWI6 THEN 000 OR 110
110: 0.001268 (1644,4,115,3)

IF m_allantoin_and_allantoate_transporters_n18-FKH2 AND ECB-NDD1 THEN 010 OR 110
110: 0.001268 (1644,4,115,3)

IF SFF'-RLM1 AND ECB-NDD1 THEN 110 OR 111
110: 0.001268 (1644,4,115,3)

IF SFF'-RLM1 AND ECB-FKH2 THEN 110 OR 111
110: 0.001268 (1644,4,115,3)

IF SFF'-RLM1 AND MCM1'-NDD1 AND SFF-FKH2 THEN 110 OR 111
110: 0.001268 (1644,4,115,3)

IF SFF'-FKH2 AND SFF'-RLM1 AND MCM1'-NDD1 THEN 110 OR 111
110: 0.001268 (1644,4,115,3)

IF MCM1'-MCM1 AND ECB-TEC1 THEN 000 OR 110
110: 0.001268 (1644,4,115,3)

IF ECB-MCM1 AND ECB-TEC1 THEN 000 OR 110
110: 0.001268 (1644,4,115,3)

IF SWI5-SWI6 AND m_cytoskeleton-dependenttransport_n4-MBP1 THEN 110 OR 111
110: 0.001268 (1644,4,115,3)

IF SFF'-FKH2 AND m_osmosensing_n6-ROX1 THEN 110 OR 111
110: 0.001268 (1644,4,115,3)

IF ECB-NDD1 AND ECB-STE12 THEN 000 OR 110
110: 0.001268 (1644,4,115,3)

IF ECB-FKH2 AND ECB-STE12 THEN 000 OR 110
110: 0.001268 (1644,4,115,3)

IF ECB-STE12 AND SFF-FKH1 THEN 010 OR 110
110: 0.001268 (1644,4,115,3)

IF MCM1'-SWI4 AND ECB-NDD1 AND SFF-FKH2 THEN 000 OR 110
110: 0.001268 (1644,4,115,3)

IF MCM1-YOX1 AND SFF-FKH1 THEN 110 OR 111
110: 0.001268 (1644,4,115,3)

IF MCM1'-SWI4 AND ECB-FKH2 AND SFF-FKH2 THEN 000 OR 110
110: 0.001268 (1644,4,115,3)

IF SFF'-FKH2 AND ECB-MCM1 AND m_nucleotide_transport_n9-MCM1 THEN 010 OR 110

110: 0.001268 (1644,4,115,3)

IF m_breakdown_of_lipids_fatty_acids_and_isoprenoids_n8-SWI6 AND m_allantoin_and_allantoate_transporters_n7-ACE2 THEN 110 OR 111
110: 0.001268 (1644,4,115,3)

IF m_breakdown_of_lipids_fatty_acids_and_isoprenoids_n8-MBP1 AND SWI5-SWI6 THEN 110 OR 111
110: 0.001268 (1644,4,115,3)

IF MCM1-FKH2 AND ECB-NDD1 AND m_other_morphogenetic_activities_n7-MCM1 THEN 010 OR 110
110: 0.001268 (1644,4,115,3)

IF MCM1-FKH2 AND ECB-MCM1 AND m_other_morphogenetic_activities_n7-MCM1 THEN 010 OR 110
110: 0.001268 (1644,4,115,3)

IF SFF'-FKH2 AND ECB-NDD1 AND m_other_morphogenetic_activities_n7-MCM1 THEN 010 OR 110
110: 0.001268 (1644,4,115,3)

IF SFF'-FKH2 AND ECB-MCM1 AND m_other_morphogenetic_activities_n7-MCM1 THEN 010 OR 110
110: 0.001268 (1644,4,115,3)

IF m_breakdown_of_lipids_fatty_acids_and_isoprenoids_n8-MBP1 AND m_breakdown_of_lipids_fatty_acids_and_isoprenoids_n8-SWI6 AND SWI5-SWI6 THEN 110 OR 111
110: 0.001268 (1644,4,115,3)

IF SFF'-FKH2 AND MCM1'-MCM1 AND m_nucleotide_transport_n9-MCM1 THEN 010 OR 110
110: 0.001268 (1644,4,115,3)

IF m_organization_of_golgi_n7-FKH1 AND SFF-FKH2 THEN 000 OR 110
110: 0.001268 (1644,4,115,3)

IF SFF'-FKH2 AND ECB-FKH2 AND m_nucleotide_transport_n9-MCM1 THEN 010 OR 110
110: 0.001268 (1644,4,115,3)

IF SFF'-FKH2 AND ECB-NDD1 AND m_nucleotide_transport_n9-MCM1 THEN 010 OR 110
110: 0.001268 (1644,4,115,3)

IF MCM1'-NDD1 AND m_nucleotide_transport_n9-MCM1 THEN 010 OR 110
110: 0.001268 (1644,4,115,3)

IF MCM1'-MCM1 AND ECB-NDD1 AND m_nucleotide_transport_n9-MCM1 THEN 010 OR 110
110: 0.001268 (1644,4,115,3)

IF SFF'-MCM1 AND ECB-NDD1 AND m_nucleotide_transport_n9-MCM1 THEN 010 OR 110
110: 0.001268 (1644,4,115,3)

IF MCM1'-MCM1 AND ECB-FKH2 AND m_nucleotide_transport_n9-MCM1 THEN 010 OR 110
110: 0.001268 (1644,4,115,3)

IF MCM1-NDD1 AND ECB-YOX1 THEN 000 OR 010 OR 110 OR 111
110: 0.001278 (1644,8,115,4)

IF MCM1-NDD1 AND SFF-FKH1 THEN 000 OR 100 OR 110 OR 111
110: 0.001278 (1644,8,115,4)

IF SFF-FKH1 AND SFF-FKH2 AND ALPHA1'-UPC2 THEN 000 OR 010 OR 110
110: 0.001278 (1644,8,115,4)

IF MCM1'-NDD1 AND ECB-YOX1 THEN 000 OR 010 OR 110 OR 111
110: 0.001278 (1644,8,115,4)

IF SFF'-FKH1 AND SFF'-FKH2 AND SFF-FKH1 AND ALPHA1'-UPC2 THEN 000 OR 010 OR 110
110: 0.001278 (1644,8,115,4)

IF SFF'-FKH1 AND SFF-FKH2 AND ALPHA1'-UPC2 THEN 000 OR 010 OR 110
110: 0.001278 (1644,8,115,4)

IF MCM1-YOX1 AND ECB-NDD1 THEN 000 OR 010 OR 110 OR 111
110: 0.001278 (1644,8,115,4)

IF MCM1-FKH2 AND MCM1-YOX1 THEN 000 OR 010 OR 110 OR 111

110: 0.001278 (1644,8,115,4)

IF SFF'-FKH2 AND SFF-FKH1 AND ALPHA1'-UPC2 THEN 000 OR 010 OR 110
110: 0.001278 (1644,8,115,4)

IF ECB-NDD1 AND ECB-YOX1 THEN 000 OR 010 OR 110 OR 111
110: 0.001278 (1644,8,115,4)

IF MCM1'-SWI4 AND SCB-SWI4 THEN 000 OR 010 OR 100 OR 110 OR 111
111: 0.001528 (1644,21,19,3)

IF m_other_mrna-transcription_activities_n20-RAP1 AND ALPHA1'-SFP1 THEN 000 OR 001 OR 110
001: 0.001763 (1644,8,55,3)

IF SWI5-SWI6 AND m_nutritional_response_pathway_n7-SWI6 THEN 000 OR 010 OR 101 OR 110 OR 111
101: 0.00178 (1644,10,11,2)

IF SFF-FKH1 AND m_regulation_of_lipid_fatty-acid_and_isoprenoid_biosynthesis_n8.scn-FKH1 THEN 000 OR 100 OR 110
110: 0.001845 (1644,14,115,5)

IF SFF'-MCM1 AND SWI5-SWI6 THEN 000 OR 100 OR 101 OR 110 OR 111
111: 0.001847 (1644,6,19,2)

IF MCM1'-SWI4 AND mPROTEOL18(m_proteolysis_n18)-SWI6 AND SCB-SWI4 THEN 000 OR 010 OR 110 OR 111
111: 0.001847 (1644,6,19,2)

IF mPROTEOL18(m_proteolysis_n18)-SWI6 AND SCB-SWI4 THEN 000 OR 010 OR 110 OR 111
111: 0.001847 (1644,6,19,2)

IF m_ion_transporters_n11-SWI6 AND m_nutritional_response_pathway_n7-SWI4 THEN 000 OR 111
111: 0.001847 (1644,6,19,2)

IF m_ion_transporters_n11-SWI4 AND m_nutritional_response_pathway_n7-SWI6 THEN 000 OR 111
111: 0.001847 (1644,6,19,2)

IF SFF'-FKH2 AND SWI5-SKN7 THEN 000 OR 010 OR 100 OR 110 OR 111
111: 0.001847 (1644,6,19,2)

IF m_nutritional_response_pathway_n7-SWI6 AND m_deoxyribonucleotide_metabolism_n10-SWI6 THEN 000 OR 110 OR 111
111: 0.001847 (1644,6,19,2)

IF SFF'-FKH1 AND m_deoxyribonucleotide_metabolism_n4-SWI6 THEN 000 OR 010 OR 110 OR 111
111: 0.001847 (1644,6,19,2)

IF SFF'-FKH1 AND m_allantoin_and_allantoate_transporters_n7-ACE2 THEN 000 OR 110 OR 111
111: 0.001847 (1644,6,19,2)

IF SWI5-SKN7 AND SCB-AZF1 THEN 000 OR 010 OR 111
111: 0.001847 (1644,6,19,2)

IF SFF'-FKH1 AND SCB-SWI4 THEN 000 OR 110 OR 111
111: 0.001847 (1644,6,19,2)

IF SFF-FKH1 AND m_deoxyribonucleotide_metabolism_n4-SWI6 THEN 000 OR 010 OR 110 OR 111
111: 0.001847 (1644,6,19,2)

IF m_amino-acid_transporters_n11-SKN7 AND SCB-SWI4 THEN 000 OR 010 OR 110 OR 111
111: 0.001847 (1644,6,19,2)

IF m_g-proteins_n12-INO4 AND m_anion_transporters_n17-INO4 THEN 000 OR 001 OR 100 OR 110
100: 0.001864 (1644,8,127,4)

IF m_nutritional_response_pathway_n7-SWI6 THEN 000 OR 010 OR 101 OR 110 OR 111
111: 0.002005 (1644,23,19,3)

IF SFF-FKH2 AND ALPHA1'-UPC2 THEN 000 OR 010 OR 110
110: 0.002177 (1644,9,115,4)

IF SWI5-SWI6 AND m_allantoin_and_allantoate_transporters_n13-SWI6 THEN 000 OR 011 OR 100 OR 101 OR 110 OR 111
110: 0.002177 (1644,9,115,4)

IF MCM1'-SWI4 AND SFF-FKH2 THEN 000 OR 100 OR 110
110: 0.002177 (1644,9,115,4)

IF SFF'-MCM1 AND MCM1'-NDD1 AND SFF-FKH1 THEN 000 OR 100 OR 110 OR 111
110: 0.002177 (1644,9,115,4)

IF m_other_proteolytic_degradation_n7-SWI6 THEN 000 OR 110
110: 0.002177 (1644,9,115,4)

IF SFF'-HIR3 AND RAP1-GAT3 AND ALPHA1-DAT1 THEN 000 OR 010
010: 0.00227 (1644,4,140,3)

IF RAP1-GAT3 AND RAP1-RAP1 AND ALPHA1-DAT1 THEN 000 OR 010
010: 0.00227 (1644,4,140,3)

IF RAP1-RAP1 AND RAP1-YAP5 AND ALPHA1-DAT1 THEN 000 OR 010
010: 0.00227 (1644,4,140,3)

IF RAP1-RAP1 AND ALPHA1-DAT1 AND ALPHA1-GAT3 THEN 000 OR 010
010: 0.00227 (1644,4,140,3)

IF SFF'-HIR3 AND RAP1-RAP1 AND ALPHA1-DAT1 THEN 000 OR 010
010: 0.00227 (1644,4,140,3)

IF RAP1-DAT1 AND ALPHA1-GAT3 THEN 000 OR 010
010: 0.00227 (1644,4,140,3)

IF SFF'-HIR3 AND RAP1-DAT1 THEN 000 OR 010
010: 0.00227 (1644,4,140,3)

IF RAP1-DAT1 AND RAP1-GAT3 THEN 000 OR 010
010: 0.00227 (1644,4,140,3)

IF RAP1-FHL1 AND ALPHA1-DAT1 THEN 000 OR 010
010: 0.00227 (1644,4,140,3)

IF RAP1-DAT1 AND RAP1-YAP5 THEN 000 OR 010
010: 0.00227 (1644,4,140,3)

IF RAP1-PDR1 AND ALPHA1-DAT1 THEN 000 OR 010
010: 0.00227 (1644,4,140,3)

IF RAP1-DAT1 AND RAP1-FHL1 THEN 000 OR 010
010: 0.00227 (1644,4,140,3)

IF RAP1-YAP5 AND ALPHA1-DAT1 THEN 000 OR 010
010: 0.00227 (1644,4,140,3)

IF RAP1-DAT1 AND RAP1-PDR1 THEN 000 OR 010
010: 0.00227 (1644,4,140,3)

IF RAP1-GAT3 AND ALPHA1-DAT1 THEN 000 OR 010
010: 0.00227 (1644,4,140,3)

IF RAP1-DAT1 AND RAP1-GAT3 AND RAP1-RAP1 THEN 000 OR 010
010: 0.00227 (1644,4,140,3)

IF RAP1-DAT1 AND RAP1-GAT3 AND RAP1-PDR1 THEN 000 OR 010
010: 0.00227 (1644,4,140,3)

IF RAP1-DAT1 AND RAP1-GAT3 AND RAP1-YAP5 THEN 000 OR 010
010: 0.00227 (1644,4,140,3)

IF RAP1-PDR1 AND RAP1-RAP1 AND ALPHA1-DAT1 THEN 000 OR 010
010: 0.00227 (1644,4,140,3)

IF RAP1-GAT3 AND ALPHA1-DAT1 AND ALPHA1-GAT3 THEN 000 OR 010
010: 0.00227 (1644,4,140,3)

IF SFF'-HIR3 AND RAP1-PDR1 AND ALPHA1-DAT1 THEN 000 OR 010
010: 0.00227 (1644,4,140,3)

IF RAP1-DAT1 AND RAP1-FHL1 AND RAP1-RAP1 THEN 000 OR 010
010: 0.00227 (1644,4,140,3)

IF SFF'-HIR3 AND ALPHA1-DAT1 AND ALPHA1-GAT3 THEN 000 OR 010
010: 0.00227 (1644,4,140,3)

IF SFF'-HIR3 AND RAP1-YAP5 AND ALPHA1-DAT1 THEN 000 OR 010
010: 0.00227 (1644,4,140,3)

IF SFF'-HIR3 AND RAP1-DAT1 AND ALPHA1-DAT1 THEN 000 OR 010
010: 0.00227 (1644,4,140,3)

IF RAP1-DAT1 AND RAP1-RAP1 AND ALPHA1-GAT3 THEN 000 OR 010
010: 0.00227 (1644,4,140,3)

IF RAP1-DAT1 AND RAP1-PDR1 AND RAP1-RAP1 THEN 000 OR 010
010: 0.00227 (1644,4,140,3)

IF RAP1-FHL1 AND RAP1-RAP1 AND ALPHA1-DAT1 THEN 000 OR 010
010: 0.00227 (1644,4,140,3)

IF m_other_proteolytic_degradation_n2-MTH1 AND m_regulation_of_lipid_fatty-acid_and_isoprenoid_biosynthesis_n8.scn-MTH1 THEN 000 OR 010
010: 0.00227 (1644,4,140,3)

IF RAP1-DAT1 AND RAP1-FHL1 AND ALPHA1-GAT3 THEN 000 OR 010
010: 0.00227 (1644,4,140,3)

IF RAP1-PDR1 AND ALPHA1-DAT1 AND ALPHA1-GAT3 THEN 000 OR 010
010: 0.00227 (1644,4,140,3)

IF RAP1-DAT1 AND ALPHA1-DAT1 AND ALPHA1-GAT3 THEN 000 OR 010
010: 0.00227 (1644,4,140,3)

IF m_metal_ion_transporters_n14-THI2 THEN 000 OR 010
010: 0.00227 (1644,4,140,3)

IF RAP1-DAT1 AND RAP1-RAP1 AND RAP1-YAP5 THEN 000 OR 010
010: 0.00227 (1644,4,140,3)

IF m_anion_transporters_n4-MTH1 AND m_regulation_of_lipid_fatty-acid_and_isoprenoid_biosynthesis_n8.scn-MTH1 THEN 000 OR 010
010: 0.00227 (1644,4,140,3)

IF SFF'-HIR3 AND RAP1-FHL1 AND ALPHA1-DAT1 THEN 000 OR 010
010: 0.00227 (1644,4,140,3)

IF SFF'-HIR3 AND RAP1-DAT1 AND RAP1-FHL1 THEN 000 OR 010
010: 0.00227 (1644,4,140,3)

IF SFF'-HIR3 AND RAP1-DAT1 AND ALPHA1-GAT3 THEN 000 OR 010
010: 0.00227 (1644,4,140,3)

IF SFF'-HIR3 AND RAP1-DAT1 AND RAP1-RAP1 AND ALPHA1-DAT1 THEN 000 OR 010
010: 0.00227 (1644,4,140,3)

IF MCM1'-SWI4 THEN 000 OR 010 OR 100 OR 101 OR 110 OR 111
111: 0.002428 (1644,52,19,4)

IF m_allantoin_and_allantoate_transporters_n7-ACE2 AND SWI5-SWI6 THEN 000 OR 110 OR 111
111: 0.002568 (1644,7,19,2)

IF m_glycolysis_and_gluconeogenesis_n27-SWI6 THEN 000 OR 010 OR 110 OR 111
111: 0.002568 (1644,7,19,2)

IF m_deoxyribonucleotide_metabolism_n4-SWI6 THEN 000 OR 010 OR 110 OR 111
111: 0.002568 (1644,7,19,2)

IF m_ion_transporters_n11-SWI4 AND m_nutritional_response_pathway_n7-SWI4 THEN 000 OR 111
111: 0.002568 (1644,7,19,2)

IF SFF'-FKH1 AND SFF-FKH2 AND SWI5-SWI6 THEN 000 OR 100 OR 110 OR 111
111: 0.002568 (1644,7,19,2)

IF SFF'-RLM1 AND SCB-SWI4 THEN 000 OR 110 OR 111
111: 0.002568 (1644,7,19,2)

IF SFF'-RLM1 AND m_deoxyribonucleotide_metabolism_n10-SWI6 THEN 000 OR 110 OR 111
111: 0.002568 (1644,7,19,2)

IF SFF'-FKH1 AND SFF'-FKH2 AND SWI5-SWI6 THEN 000 OR 100 OR 110 OR 111
111: 0.002568 (1644,7,19,2)

IF m_ion_transporters_n11-SWI6 AND m_nutritional_response_pathway_n7-SWI6 THEN 000 OR 010 OR 111
111: 0.002568 (1644,7,19,2)

IF MCM1'-SWI4 AND m_deoxyribonucleotide_metabolism_n10-SWI6 THEN 000 OR 110 OR 111
111: 0.002568 (1644,7,19,2)

IF SFF'-FKH2 AND SFF-FKH1 AND SWI5-SWI6 THEN 000 OR 100 OR 110 OR 111
111: 0.002568 (1644,7,19,2)

IF SFF-FKH1 AND SFF-FKH2 AND SWI5-SWI6 THEN 000 OR 100 OR 110 OR 111
111: 0.002568 (1644,7,19,2)

IF SFF'-HIR3 AND RAP1-RAP1 THEN 000 OR 010
010: 0.002692 (1644,8,140,4)

IF SFF'-HIR3 AND RAP1-PDR1 THEN 000 OR 010
010: 0.002692 (1644,8,140,4)

IF SFF'-HIR3 AND RAP1-FHL1 THEN 000 OR 010
010: 0.002692 (1644,8,140,4)

IF SFF'-HIR3 AND RAP1-FHL1 AND RAP1-PDR1 THEN 000 OR 010
010: 0.002692 (1644,8,140,4)

IF MCM1'-MCM1 AND ECB-FKH2 AND SFF-FKH1 THEN 000 OR 110 OR 111
110: 0.003007 (1644,5,115,3)

IF MCM1'-SWI4 AND ECB-FKH2 THEN 000 OR 110
110: 0.003007 (1644,5,115,3)

IF MCM1'-NDD1 AND MCM1'-SWI4 AND ECB-NDD1 THEN 000 OR 110
110: 0.003007 (1644,5,115,3)

IF SFF-FKH2 AND m_other_transport_facilitators_n10-SWI6 THEN 000 OR 110
110: 0.003007 (1644,5,115,3)

IF SFF-FKH2 AND m_cytoskeleton-dependenttransport_n4-MBP1 THEN 000 OR 110 OR 111
110: 0.003007 (1644,5,115,3)

IF SFF'-FKH2 AND m_cytoskeleton-dependenttransport_n4-MBP1 THEN 000 OR 110 OR 111
110: 0.003007 (1644,5,115,3)

IF MCM1-YOX1 AND MCM1'-NDD1 AND SFF-FKH2 THEN 000 OR 110 OR 111
110: 0.003007 (1644,5,115,3)

IF SFF'-FKH2 AND m_other_transport_facilitators_n10-SWI6 THEN 000 OR 110
110: 0.003007 (1644,5,115,3)

IF MCM1-NDD1 AND MCM1-YOX1 AND SFF-FKH2 THEN 000 OR 110 OR 111
110: 0.003007 (1644,5,115,3)

IF MCM1-MCM1 AND MCM1-YOX1 AND SFF-FKH2 THEN 000 OR 110 OR 111
110: 0.003007 (1644,5,115,3)

IF MCM1-YOX1 AND MCM1'-MCM1 AND SFF-FKH2 THEN 000 OR 110 OR 111

110: 0.003007 (1644,5,115,3)

IF ECB-YOX1 AND m_other_morphogenetic_activities_n7-MCM1 THEN 000 OR 010 OR 110
110: 0.003007 (1644,5,115,3)

IF m_nutritional_response_pathway_n7-SWI6 AND MCB-MBP1 THEN 010 OR 101 OR 110
110: 0.003007 (1644,5,115,3)

IF m_other_signal-transduction_activities_n8-INO4 AND m_other_mrna-transcription_activities_n20-INO4 THEN 000 OR 001 OR 110
110: 0.003007 (1644,5,115,3)

IF m_g-proteins_n12-SKN7 AND SWI5-SWI6 AND m_amino-acid_transporters_n11-SKN7 THEN 000 OR 010 OR 110
110: 0.003007 (1644,5,115,3)

IF m_breakdown_of_lipids_fatty_acids_and_isoprenoids_n8-MBP1 AND m_allantoin_and_allantoate_transporters_n7-ACE2 THEN 000 OR 110 OR 111
110: 0.003007 (1644,5,115,3)

IF m_g-proteins_n12-SKN7 AND SWI5-SKN7 AND SWI5-SWI6 AND m_amino-acid_transporters_n11-SKN7 THEN 000 OR 010 OR 110
110: 0.003007 (1644,5,115,3)

IF SFF'-MCM1 AND MCM1-MCM1 AND m_nucleotide_transport_n9-MCM1 THEN 010 OR 110
110: 0.003007 (1644,5,115,3)

IF SFF'-FKH1 AND m_nucleotide_transport_n9-SWI6 THEN 000 OR 010 OR 110
110: 0.003007 (1644,5,115,3)

IF SFF'-MCM1 AND ECB-MCM1 AND m_nucleotide_transport_n9-MCM1 THEN 010 OR 110
110: 0.003007 (1644,5,115,3)

IF ECB-FKH2 AND m_nucleotide_transport_n9-MCM1 THEN 000 OR 010 OR 110
110: 0.003007 (1644,5,115,3)

IF ECB-NDD1 AND m_nucleotide_transport_n9-MCM1 THEN 000 OR 010 OR 110
110: 0.003007 (1644,5,115,3)

IF SFF'-MCM1 AND m_pentose-phosphate_pathway_n14-FKH1 THEN 100 OR 110 OR 111
110: 0.003007 (1644,5,115,3)

IF MCM1'-MCM1 AND MCM1'-NDD1 AND ECB-MCM1 AND SFF-FKH1 THEN 000 OR 110 OR 111
110: 0.003007 (1644,5,115,3)

IF MCM1-MCM1 AND m_pentose-phosphate_pathway_n14-FKH1 THEN 100 OR 110 OR 111
110: 0.003007 (1644,5,115,3)

IF MCM1'-NDD1 AND MCM1'-SWI4 AND ECB-FKH2 THEN 000 OR 110
110: 0.003007 (1644,5,115,3)

IF m_allantoin_and_allantoate_transporters_n18-FKH2 AND ECB-FKH2 THEN 000 OR 010 OR 110
110: 0.003007 (1644,5,115,3)

IF ECB-MCM1 AND SFF-FKH1 AND SFF-FKH2 THEN 000 OR 110 OR 111
110: 0.003007 (1644,5,115,3)

IF MCM1'-NDD1 AND ECB-MCM1 AND SFF-FKH1 THEN 000 OR 110 OR 111
110: 0.003007 (1644,5,115,3)

IF m_cytok9-SWI6 AND MCB-MBP1 THEN 010 OR 110 OR 111
110: 0.003007 (1644,5,115,3)

IF MCM1-NDD1 AND m_allantoin_and_allantoate_transporters_n18-FKH2 THEN 010 OR 100 OR 110
110: 0.003007 (1644,5,115,3)

IF m_cytok9-SWI6 AND MCB-SWI6 THEN 010 OR 110 OR 111
110: 0.003007 (1644,5,115,3)

IF MCM1'-SWI4 AND ECB-NDD1 THEN 000 OR 110
110: 0.003007 (1644,5,115,3)

IF SWI5-SWI6 AND m_pentose-phosphate_pathway_n23-MET4 THEN 000 OR 110

110: 0.003007 (1644,5,115,3)

IF ALPHA1-SFP1 AND m_deoxyribonucleotide_metabolism_n10-INO4 THEN 000 OR 001
001: 0.003228 (1644,3,55,2)

IF m_anion_transporters_n22-SKN7 AND SCB-UME6 THEN 000 OR 001
001: 0.003228 (1644,3,55,2)

IF m_g-proteins_n12-INO4 AND m_nutritional_response_pathway_n8-INO4 AND m_other_mrna-transcription_activities_n20-RAP1 THEN 000 OR 001
001: 0.003228 (1644,3,55,2)

IF m_meiosis_n3-UME6 AND m_anion_transporters_n20-RAP1 THEN 000 OR 001
001: 0.003228 (1644,3,55,2)

IF m_RPE32-YAP5 AND m_RPE21-FHL1 THEN 000 OR 001
001: 0.003228 (1644,3,55,2)

IF m_anion_transporters_n22-RAP1 AND m_meiosis_n3-UME6 THEN 000 OR 001
001: 0.003228 (1644,3,55,2)

IF m_other_mrna-transcription_activities_n20-RAP1 AND STRE-RAP1 AND m_amino-acid_transport_n20-RAP1 THEN 000 OR 001
001: 0.003228 (1644,3,55,2)

IF m_RPE32-RAP1 AND m_amino-acid_transport_n20-RAP1 THEN 000 OR 001
001: 0.003228 (1644,3,55,2)

IF m_RPE32-RAP1 AND m_RPE21-RGM1 THEN 000 OR 001
001: 0.003228 (1644,3,55,2)

IF SFF'-HIR3 AND m_glycolysis_and_gluconeogenesis_n11-CST6 THEN 000 OR 001
001: 0.003228 (1644,3,55,2)

IF m_anion_transporters_n22-RAP1 AND SCB-UME6 THEN 000 OR 001
001: 0.003228 (1644,3,55,2)

IF SCB-UME6 AND m_anion_transporters_n20-RAP1 THEN 000 OR 001
001: 0.003228 (1644,3,55,2)

IF m_other_mrna-transcription_activities_n20-RAP1 AND m_pentose-phosphate_pathway_n23-RAP1 AND m_amino-acid_transport_n20-RAP1 THEN 000 OR 001
001: 0.003228 (1644,3,55,2)

IF SWI5-SKN7 AND m_anion_transporters_n20-RAP1 THEN 000 OR 001
001: 0.003228 (1644,3,55,2)

IF m_other_energy_generation_activities_n16-INO4 AND m_nucleotide_transport_n9-INO4 THEN 001 OR 110
001: 0.003228 (1644,3,55,2)

IF m_nutritional_response_pathway_n8-INO4 AND ALPHA1'-SFP1 THEN 000 OR 001
001: 0.003228 (1644,3,55,2)

IF m_other_mrna-transcription_activities_n20-RAP1 AND m_RPE32-RAP1 THEN 000 OR 001
001: 0.003228 (1644,3,55,2)

IF m_other_mrna-transcription_activities_n20-RAP1 AND m_pentose-phosphate_pathway_n23-RAP1 AND m_amino-acid_transport_n20-MTH1 THEN 000 OR 001
001: 0.003228 (1644,3,55,2)

IF m_other_mrna-transcription_activities_n20-RAP1 AND m_RPE68-FHL1 THEN 000 OR 001
001: 0.003228 (1644,3,55,2)

IF SWI5-INO4 AND m_other_mrna-transcription_activities_n20-RAP1 THEN 000 OR 001
001: 0.003228 (1644,3,55,2)

IF m_RPE68-FHL1 AND ALPHA1'-SFP1 THEN 000 OR 001
001: 0.003228 (1644,3,55,2)

IF m_anion_transporters_n22-RAP1 AND SWI5-SKN7 THEN 000 OR 001
001: 0.003228 (1644,3,55,2)

IF m_RPE32-YAP5 AND m_RPE21-RGM1 THEN 000 OR 001
001: 0.003228 (1644,3,55,2)

IF STRE-RAP1 AND m_amino-acid_transport_n20-MTH1 THEN 000 OR 001
001: 0.003228 (1644,3,55,2)

IF m_RPE68-RAP1 AND ALPHA1'-SFP1 THEN 000 OR 001
001: 0.003228 (1644,3,55,2)

IF PDR-INO4 AND m_pentose-phosphate_pathway_n23-INO4 THEN 000 OR 001
001: 0.003228 (1644,3,55,2)

IF m_other_mrna-transcription_activities_n20-RAP1 AND STRE-RAP1 AND m_pentose-phosphate_pathway_n23-RAP1 THEN 000 OR 001
001: 0.003228 (1644,3,55,2)

IF m_pentose-phosphate_pathway_n23-RAP1 AND m_amino-acid_transport_n20-MTH1 THEN 000 OR 001
001: 0.003228 (1644,3,55,2)

IF m_RPE68-PDR1 AND ALPHA1'-SFP1 THEN 000 OR 001
001: 0.003228 (1644,3,55,2)

IF m_RPE32-RAP1 AND m_RPE21-FHL1 THEN 000 OR 001
001: 0.003228 (1644,3,55,2)

IF SWI5-INO4 AND m_other_mrna-transcription_activities_n20-INO4 AND ALPHA1'-SFP1 THEN 000 OR 001
001: 0.003228 (1644,3,55,2)

IF SFF'-HIR3 AND m_glycolysis_and_gluconeogenesis_n11-YER130C THEN 000 OR 001
001: 0.003228 (1644,3,55,2)

IF m_organization_of_chromosome_structure_n17-INO4 AND ALPHA1'-SFP1 THEN 000 OR 001
001: 0.003228 (1644,3,55,2)

IF m_other_mrna-transcription_activities_n20-RAP1 AND STRE-RAP1 AND m_amino-acid_transport_n20-MTH1 THEN 000 OR 001
001: 0.003228 (1644,3,55,2)

IF m_RPE32-FHL1 AND m_RPE21-FHL1 THEN 000 OR 001
001: 0.003228 (1644,3,55,2)

IF m_RPE32-FHL1 AND m_RPE21-RGM1 THEN 000 OR 001
001: 0.003228 (1644,3,55,2)

IF SWI5-SKN7 AND m_meiosis_n3-UME6 THEN 001 OR 100
001: 0.003228 (1644,3,55,2)

IF m_allantoin_and_allantoate_transporters_n7-ACE2 AND SFF-FKH2 THEN 000 OR 100 OR 110 OR 111
111: 0.003401 (1644,8,19,2)

IF m_ion_transporters_n11-SWI4 AND SCB-SWI4 THEN 000 OR 111
111: 0.003401 (1644,8,19,2)

IF SFF'-FKH2 AND m_g-proteins_n12-SKN7 THEN 000 OR 010 OR 111
111: 0.003401 (1644,8,19,2)

IF m_ion_transporters_n11-SKN7 AND m_LFTE17-SKN7 THEN 000 OR 100 OR 111
111: 0.003401 (1644,8,19,2)

IF SFF'-FKH2 AND m_allantoin_and_allantoate_transporters_n7-ACE2 THEN 000 OR 100 OR 110 OR 111
111: 0.003401 (1644,8,19,2)

IF m_glycolysis_and_gluconeogenesis_n27-SWI4 THEN 000 OR 010 OR 111
111: 0.003401 (1644,8,19,2)

IF MCM1'-SWI4 AND m_amino-acid_transporters_n11-SKN7 THEN 000 OR 010 OR 111
111: 0.003401 (1644,8,19,2)

IF SFF'-FKH2 AND m_allantoin_and_allantoate_transporters_n18-FKH2 THEN 000 OR 010 OR 100 OR 110
110: 0.003434 (1644,10,115,4)

IF SWI5-SWI6 AND mPROTEOL18(m_proteolysis_n18)-MBP1 AND mPROTEOL18(m_proteolysis_n18)-SWI6 THEN 000 OR 010 OR 110
110: 0.003434 (1644,10,115,4)

IF SFF'-MCM1 AND MCM1'-SWI4 THEN 000 OR 100 OR 101 OR 110
110: 0.003434 (1644,10,115,4)

IF SFF-FKH2 AND m_other_morphogenetic_activities_n7-MCM1 THEN 000 OR 011 OR 100 OR 110 OR 111
110: 0.003434 (1644,10,115,4)

IF SFF'-FKH1 AND MCM1'-MCM1 AND SFF-FKH2 THEN 000 OR 100 OR 110 OR 111
110: 0.003434 (1644,10,115,4)

IF SWI5-SWI6 AND mPROTEOL18(m_proteolysis_n18)-MBP1 THEN 000 OR 010 OR 110
110: 0.003434 (1644,10,115,4)

IF SFF'-FKH2 AND MCM1'-MCM1 AND SFF-FKH1 THEN 000 OR 100 OR 110 OR 111
110: 0.003434 (1644,10,115,4)

IF m_breakdown_of_lipids_fatty_acids_and_isoprenoids_n8-SWI6 THEN 000 OR 100 OR 110 OR 111
110: 0.003434 (1644,10,115,4)

IF SFF'-FKH1 AND SFF'-FKH2 AND ALPHA1'-UPC2 THEN 000 OR 010 OR 110
110: 0.003434 (1644,10,115,4)

IF m_other_mrna-transcription_activities_n20-RAP1 THEN 000 OR 001 OR 100 OR 110
001: 0.003523 (1644,32,55,5)

IF m_organization_of_plasma_membrane_n17-INO4 THEN 000 OR 100 OR 101 OR 110
101: 0.003547 (1644,14,11,2)

IF m_pentose-phosphate_pathway_n5-SWI4 THEN 000 OR 100 OR 101 OR 110 OR 111
101: 0.003547 (1644,14,11,2)

IF m_phosphate_transport_n8-SWI4 THEN 000 OR 010 OR 100 OR 110
110: 0.003593 (1644,16,115,5)

IF m_ion_transporters_n11-SWI4 AND m_ion_transporters_n11-SWI6 THEN 000 OR 111
111: 0.004343 (1644,9,19,2)

IF SFF'-FKH1 AND m_g-proteins_n12-SKN7 THEN 000 OR 010 OR 100 OR 111
111: 0.004343 (1644,9,19,2)

IF MCM1'-SWI4 AND m_ion_transporters_n11-SWI4 THEN 000 OR 110 OR 111
111: 0.004343 (1644,9,19,2)

IF m_metabolism_of_energy_reserves_n27-NDD1 THEN 000 OR 111
111: 0.004343 (1644,9,19,2)

IF m_nutritional_response_pathway_n7-SWI4 AND SCB-SWI4 THEN 000 OR 111
111: 0.004343 (1644,9,19,2)

IF m_g-proteins_n12-SKN7 AND SCB-AZF1 THEN 000 OR 010 OR 100 OR 111
111: 0.004343 (1644,9,19,2)

IF SWI5-SWI6 AND SCB-SWI4 THEN 000 OR 010 OR 100 OR 110 OR 111
111: 0.004343 (1644,9,19,2)

IF SFF-FKH2 AND SWI5-SWI6 THEN 000 OR 001 OR 100 OR 110 OR 111
110: 0.00481 (1644,17,115,5)

IF SFF'-MCM1 AND SFF-FKH1 THEN 000 OR 010 OR 100 OR 110 OR 111
110: 0.00481 (1644,17,115,5)

IF mPROTEOL18(m_proteolysis_n18)-MBP1 AND mPROTEOL18(m_proteolysis_n18)-SWI6 THEN 000 OR 010 OR 100 OR 110 OR 111
110: 0.00481 (1644,17,115,5)

IF SFF'-FKH2 AND SFF-FKH2 AND SWI5-SWI6 THEN 000 OR 001 OR 100 OR 110 OR 111
110: 0.00481 (1644,17,115,5)

IF m_g-proteins_n12-INO4 AND m_nucleotide_transport_n9-INO4 THEN 000 OR 001 OR 110

001: 0.004835 (1644,11,55,3)

IF m_fermentation_n4-FKH2 AND m_other_energy_generation_activities_n9-FKH2 THEN 110
110: 0.004854 (1644,2,115,2)

IF MCM1-NDD1 AND ECB-TEC1 THEN 110
110: 0.004854 (1644,2,115,2)

IF MCM1-NDD1 AND ECB-STE12 THEN 110
110: 0.004854 (1644,2,115,2)

IF MCM1'-NDD1 AND m_cell_death_n15-RLM1 THEN 110
110: 0.004854 (1644,2,115,2)

IF MCM1-FKH2 AND ECB-STE12 THEN 110
110: 0.004854 (1644,2,115,2)

IF ECB-FKH2 AND m_cell_death_n15-RLM1 THEN 110
110: 0.004854 (1644,2,115,2)

IF ECB-MCM1 AND m_cell_death_n15-RLM1 THEN 110
110: 0.004854 (1644,2,115,2)

IF MCM1-FKH2 AND ECB-TEC1 THEN 110
110: 0.004854 (1644,2,115,2)

IF ECB-NDD1 AND m_cell_death_n15-RLM1 THEN 110
110: 0.004854 (1644,2,115,2)

IF MCM1'-MCM1 AND m_cell_death_n15-RLM1 THEN 110
110: 0.004854 (1644,2,115,2)

IF SFF-FKH1 AND m_lipid_and_fatty-acid_transport_n11-GAT1 AND SWI5-SWI6 THEN 110
110: 0.004854 (1644,2,115,2)

IF SFF'-FKH1 AND m_lipid_and_fatty-acid_transport_n11-GAT1 AND SWI5-SWI6 THEN 110
110: 0.004854 (1644,2,115,2)

IF SFF-FKH1 AND m_regulation_of_lipid_fatty-acid_and_isoprenoid_biosynthesis_n8.scn-FKH1 AND m_glyoxylate_cycle_n11-GAT1 THEN 110
110: 0.004854 (1644,2,115,2)

IF LYS14-SWI6 AND m_pentose-phosphate_pathway_n23-MET4 THEN 110
110: 0.004854 (1644,2,115,2)

IF ECB-MCM1 AND ALPHA1'-UPC2 THEN 110
110: 0.004854 (1644,2,115,2)

IF MCM1-NDD1 AND ECB-DIG1 THEN 110
110: 0.004854 (1644,2,115,2)

IF m_osmosensing_n6-INO4 AND ECB-NDD1 THEN 110
110: 0.004854 (1644,2,115,2)

IF MCM1-YOX1 AND ECB-DIG1 THEN 110
110: 0.004854 (1644,2,115,2)

IF m_osmosensing_n6-INO4 AND ECB-FKH2 THEN 110
110: 0.004854 (1644,2,115,2)

IF m_organization_of_chromosome_structure_n17-SKN7 AND m_sugar_and_carbohydrate_transporters_n6-SKN7 THEN 110
110: 0.004854 (1644,2,115,2)

IF m_nitrogen_and_sulphur_metabolism_n17-SKN7 AND m_amino-acid_metabolism_n25-SWI6 THEN 110
110: 0.004854 (1644,2,115,2)

IF m_cell_rescue_defense_cell_death_and_ageing_n20-PHO4 AND m_cytoskeleton-dependenttransport_n4-ACE2 THEN 110
110: 0.004854 (1644,2,115,2)

IF m_trna_transcription_n10-SWI6 AND m_phosphate_transport_n8-SWI4 THEN 110
110: 0.004854 (1644,2,115,2)

IF m_breakdown_of_lipids_fatty_acids_and_isoprenoids_n8-SWI6 AND m_other_transport_facilitators_n10-PIP2 THEN 110
110: 0.004854 (1644,2,115,2)

IF m_cytoskeleton-dependenttransport_n4-MBP1 AND m_other_transport_facilitators_n10-PIP2 THEN 110
110: 0.004854 (1644,2,115,2)

IF SFF-FKH2 AND m_allantoin_and_allantoate_transporters_n6-PHO4 THEN 110
110: 0.004854 (1644,2,115,2)

IF m_metabolism_of_energy_reserves_n8-PIP2 AND SFF-FKH2 THEN 110
110: 0.004854 (1644,2,115,2)

IF m_biogenesis_of_cytoskeleton_n12-A1(MATA1) THEN 110
110: 0.004854 (1644,2,115,2)

IF m_phosphate_transport_n8-SWI4 AND m_cytoskeleton-dependenttransport_n4-MBP1 THEN 110
110: 0.004854 (1644,2,115,2)

IF m_metabolism_of_energy_reserves_n8-PIP2 AND m_cytoskeleton-dependenttransport_n4-MBP1 THEN 110
110: 0.004854 (1644,2,115,2)

IF m_trna_transcription_n10-SWI6 AND SFF-FKH2 THEN 110
110: 0.004854 (1644,2,115,2)

IF m_breakdown_of_lipids_fatty_acids_and_isoprenoids_n8-SWI6 AND m_metabolism_of_energy_reserves_n8-PIP2 THEN 110
110: 0.004854 (1644,2,115,2)

IF m_breakdown_of_lipids_fatty_acids_and_isoprenoids_n8-MBP1 AND m_trna_transcription_n10-SWI6 THEN 110
110: 0.004854 (1644,2,115,2)

IF m_breakdown_of_lipids_fatty_acids_and_isoprenoids_n8-SWI6 AND m_cell_rescue_defense_cell_death_and_ageing_n20-PHO4 THEN 110
110: 0.004854 (1644,2,115,2)

IF m_phosphate_transport_n8-SWI4 AND m_other_transport_facilitators_n10-PIP2 THEN 110
110: 0.004854 (1644,2,115,2)

IF m_metabolism_of_energy_reserves_n8-PIP2 AND m_allantoin_and_allantoate_transporters_n6-PHO4 THEN 110
110: 0.004854 (1644,2,115,2)

IF m_trna_transcription_n10-SWI6 AND m_other_transport_facilitators_n10-PIP2 THEN 110
110: 0.004854 (1644,2,115,2)

IF ECB-DIG1 AND MCB-SWI6 THEN 110
110: 0.004854 (1644,2,115,2)

IF m_pentose-phosphate_pathway_n14-FKH1 AND ALPHA1'-STB4 THEN 110
110: 0.004854 (1644,2,115,2)

IF m_pheromone_response_generation_n12-FKH1 AND ALPHA1'-STB4 THEN 110
110: 0.004854 (1644,2,115,2)

IF m_other_cell_growth_cell_division_and_dna_synthesis_activities_n10.scn-SPT23 AND MCB-YFL052w THEN 110
110: 0.004854 (1644,2,115,2)

IF m_other_intracellular-transport_activities_n6-UGA3 AND MCB-MIG2 THEN 110
110: 0.004854 (1644,2,115,2)

IF ECB-MCM1 AND MCB-YFL052w THEN 110
110: 0.004854 (1644,2,115,2)

IF m_other_intracellular-transport_activities_n6-UGA3 AND MCM1-MCM1 THEN 110
110: 0.004854 (1644,2,115,2)

IF ECB-MCM1 AND MCB-MIG2 THEN 110
110: 0.004854 (1644,2,115,2)

IF SFF'-MCM1 AND MCB-MIG2 THEN 110
110: 0.004854 (1644,2,115,2)

IF m_other_intracellular-transport_activities_n6-UGA3 AND m_pheromone_response_generation_n12-FKH1 THEN 110
110: 0.004854 (1644,2,115,2)

IF MCM1'-MCM1 AND MCB-YNR063W THEN 110
110: 0.004854 (1644,2,115,2)

IF m_other_cell_growth_cell_division_and_dna_synthesis_activities_n10.scn-SPT23 AND MCB-STB4 THEN 110
110: 0.004854 (1644,2,115,2)

IF m_pheromone_response_generation_n12-FKH1 AND m_other_cell_growth_cell_division_and_dna_synthesis_activities_n10.scn-SPT23 THEN 110
110: 0.004854 (1644,2,115,2)

IF m_other_intracellular-transport_activities_n6-UGA3 AND ECB-YOX1 THEN 110
110: 0.004854 (1644,2,115,2)

IF MCM1-MCM1 AND m_amino-acid_degradation_n32-UGA3 THEN 110
110: 0.004854 (1644,2,115,2)

IF MCM1-YOX1 AND MCB-YNR063W THEN 110
110: 0.004854 (1644,2,115,2)

IF m_pentose-phosphate_pathway_n14-FKH1 AND MCB-YFL052w THEN 110
110: 0.004854 (1644,2,115,2)

IF m_pheromone_response_generation_n12-FKH1 AND MCB-YFL052w THEN 110
110: 0.004854 (1644,2,115,2)

IF m_other_cell_growth_cell_division_and_dna_synthesis_activities_n10.scn-SPT23 AND MCB-MIG2 THEN 110
110: 0.004854 (1644,2,115,2)

IF m_amino-acid_degradation_n32-UGA3 AND m_other_cell_growth_cell_division_and_dna_synthesis_activities_n10.scn-SPT23 THEN 110
110: 0.004854 (1644,2,115,2)

IF ECB-YOX1 AND MCB-MIG2 THEN 110
110: 0.004854 (1644,2,115,2)

IF MCM1-YOX1 AND MCB-STB4 THEN 110
110: 0.004854 (1644,2,115,2)

IF MCM1'-MCM1 AND m_amino-acid_degradation_n32-UGA3 THEN 110
110: 0.004854 (1644,2,115,2)

IF MCM1'-MCM1 AND m_other_cell_growth_cell_division_and_dna_synthesis_activities_n10.scn-SPT23 THEN 110
110: 0.004854 (1644,2,115,2)

IF ECB-MCM1 AND MCB-STB4 THEN 110
110: 0.004854 (1644,2,115,2)

IF MCM1'-MCM1 AND MCB-STB4 THEN 110
110: 0.004854 (1644,2,115,2)

IF MCM1'-MCM1 AND MCB-YFL052w THEN 110
110: 0.004854 (1644,2,115,2)

IF SFF'-FKH1 AND MCB-YFL052w THEN 110
110: 0.004854 (1644,2,115,2)

IF SFF'-MCM1 AND MCB-STB4 THEN 110
110: 0.004854 (1644,2,115,2)

IF MCM1'-MCM1 AND MCB-MIG2 THEN 110
110: 0.004854 (1644,2,115,2)

IF SFF'-MCM1 AND MCB-YFL052w THEN 110
110: 0.004854 (1644,2,115,2)

IF MCM1'-MCM1 AND ALPHA1'-STB4 THEN 110
110: 0.004854 (1644,2,115,2)

IF m_pentose-phosphate_pathway_n14-FKH1 AND m_other_cell_growth_cell_division_and_dna_synthesis_activities_n10.scn-SPT23 THEN 110
110: 0.004854 (1644,2,115,2)

IF m_pentose-phosphate_pathway_n14-FKH1 AND MCB-MIG2 THEN 110
110: 0.004854 (1644,2,115,2)

IF MCM1-YOX1 AND MCB-YFL052w THEN 110
110: 0.004854 (1644,2,115,2)

IF MCM1'-NDD1 AND ALPHA1'-UPC2 THEN 110
110: 0.004854 (1644,2,115,2)

IF MCM1'-NDD1 AND ECB-DIG1 THEN 110
110: 0.004854 (1644,2,115,2)

IF m_g-proteins_n12-INO4 AND ECB-DIG1 THEN 110
110: 0.004854 (1644,2,115,2)

IF m_osmosensing_n6-ROX1 AND MCM1'-NDD1 THEN 110
110: 0.004854 (1644,2,115,2)

IF ECB-DIG1 AND ECB-YOX1 THEN 110
110: 0.004854 (1644,2,115,2)

IF m_osmosensing_n6-ROX1 AND ECB-MCM1 THEN 110
110: 0.004854 (1644,2,115,2)

IF m_osmosensing_n6-ROX1 AND ECB-NDD1 THEN 110
110: 0.004854 (1644,2,115,2)

IF ECB-NDD1 AND ALPHA1'-UPC2 THEN 110
110: 0.004854 (1644,2,115,2)

IF m_osmosensing_n6-ROX1 AND ECB-FKH2 THEN 110
110: 0.004854 (1644,2,115,2)

IF m_pentose-phosphate_pathway_n14-FKH1 AND MCB-YNR063W THEN 110
110: 0.004854 (1644,2,115,2)

IF SFF'-MCM1 AND MCB-YNR063W THEN 110
110: 0.004854 (1644,2,115,2)

IF SFF'-MCM1 AND m_other_cell_growth_cell_division_and_dna_synthesis_activities_n10.scn-SPT23 THEN 110
110: 0.004854 (1644,2,115,2)

IF m_osmosensing_n6-INO4 AND ALPHA1'-UPC2 THEN 110
110: 0.004854 (1644,2,115,2)

IF m_osmosensing_n6-INO4 AND ECB-DIG1 THEN 110
110: 0.004854 (1644,2,115,2)

IF m_pheromone_response_generation_n12-FKH1 AND MCB-STB4 THEN 110
110: 0.004854 (1644,2,115,2)

IF ECB-YOX1 AND MCB-YNR063W THEN 110
110: 0.004854 (1644,2,115,2)

IF m_amino-acid_degradation_n32-UGA3 AND m_pentose-phosphate_pathway_n14-FKH1 THEN 110
110: 0.004854 (1644,2,115,2)

IF SFF'-FKH1 AND ECB-DIG1 THEN 110
110: 0.004854 (1644,2,115,2)

IF m_other_intracellular-transport_activities_n6-UGA3 AND m_pentose-phosphate_pathway_n14-FKH1 THEN 110
110: 0.004854 (1644,2,115,2)

IF MCM1-YOX1 AND m_amino-acid_degradation_n32-UGA3 THEN 110
110: 0.004854 (1644,2,115,2)

IF SFF'-FKH1 AND MCB-MIG2 THEN 110

110: 0.004854 (1644,2,115,2)

IF m_osmosensing_n6-INO4 AND ECB-MCM1 THEN 110
110: 0.004854 (1644,2,115,2)

IF m_other_intracellular-transport_activities_n6-UGA3 AND MCM1-YOX1 THEN 110
110: 0.004854 (1644,2,115,2)

IF ECB-MCM1 AND MCB-YNR063W THEN 110
110: 0.004854 (1644,2,115,2)

IF ECB-YOX1 AND m_other_cell_growth_cell_division_and_dna_synthesis_activities_n10.scn-SPT23 THEN 110
110: 0.004854 (1644,2,115,2)

IF m_other_intracellular-transport_activities_n6-UGA3 AND ALPHA1'-STB4 THEN 110
110: 0.004854 (1644,2,115,2)

IF ECB-MCM1 AND m_amino-acid_degradation_n32-UGA3 THEN 110
110: 0.004854 (1644,2,115,2)

IF m_other_intracellular-transport_activities_n6-UGA3 AND m_other_cell_growth_cell_division_and_dna_synthesis_activities_n10.scn-SPT23 THEN 110
110: 0.004854 (1644,2,115,2)

IF m_other_intracellular-transport_activities_n6-UGA3 AND MCB-YNR063W THEN 110
110: 0.004854 (1644,2,115,2)

IF SFF'-FKH1 AND m_other_intracellular-transport_activities_n6-UGA3 THEN 110
110: 0.004854 (1644,2,115,2)

IF m_other_intracellular-transport_activities_n6-UGA3 AND MCB-STB4 THEN 110
110: 0.004854 (1644,2,115,2)

IF ECB-MCM1 AND ALPHA1'-STB4 THEN 110
110: 0.004854 (1644,2,115,2)

IF m_amino-acid_degradation_n32-UGA3 AND ALPHA1'-STB4 THEN 110
110: 0.004854 (1644,2,115,2)

IF ECB-YOX1 AND m_amino-acid_degradation_n32-UGA3 THEN 110
110: 0.004854 (1644,2,115,2)

IF m_other_intracellular-transport_activities_n6-UGA3 AND MCB-YFL052w THEN 110
110: 0.004854 (1644,2,115,2)

IF ECB-YOX1 AND MCB-YFL052w THEN 110
110: 0.004854 (1644,2,115,2)

IF m_pheromone_response_generation_n12-FKH1 AND ECB-MCM1 THEN 110
110: 0.004854 (1644,2,115,2)

IF m_amino-acid_degradation_n32-UGA3 AND MCB-MIG2 THEN 110
110: 0.004854 (1644,2,115,2)

IF ECB-YOX1 AND MCB-STB4 THEN 110
110: 0.004854 (1644,2,115,2)

IF m_amino-acid_degradation_n32-UGA3 AND MCB-YFL052w THEN 110
110: 0.004854 (1644,2,115,2)

IF MCM1-MCM1 AND MCB-YFL052w THEN 110
110: 0.004854 (1644,2,115,2)

IF ALPHA1'-STB4 AND MCB-MIG2 THEN 110
110: 0.004854 (1644,2,115,2)

IF SFF'-MCM1 AND m_amino-acid_degradation_n32-UGA3 THEN 110
110: 0.004854 (1644,2,115,2)

IF MCM1-MCM1 AND m_other_cell_growth_cell_division_and_dna_synthesis_activities_n10.scn-SPT23 THEN 110

110: 0.004854 (1644,2,115,2)

IF ECB-MCM1 AND m_other_cell_growth_cell_division_and_dna_synthesis_activities_n10.scn-SPT23 THEN 110
110: 0.004854 (1644,2,115,2)

IF MCM1-MCM1 AND MCB-YNR063W THEN 110
110: 0.004854 (1644,2,115,2)

IF m_other_cell_growth_cell_division_and_dna_synthesis_activities_n10.scn-SPT23 AND MCB-YNR063W THEN 110
110: 0.004854 (1644,2,115,2)

IF m_pheromone_response_generation_n12-FKH1 AND m_amino-acid_degradation_n32-UGA3 THEN 110
110: 0.004854 (1644,2,115,2)

IF m_amino-acid_degradation_n32-UGA3 AND MCB-YNR063W THEN 110
110: 0.004854 (1644,2,115,2)

IF SFF'-FKH1 AND MCB-STB4 THEN 110
110: 0.004854 (1644,2,115,2)

IF MCM1-YOX1 AND MCB-MIG2 THEN 110
110: 0.004854 (1644,2,115,2)

IF m_pentose-phosphate_pathway_n14-FKH1 AND MCB-STB4 THEN 110
110: 0.004854 (1644,2,115,2)

IF MCM1-YOX1 AND m_other_cell_growth_cell_division_and_dna_synthesis_activities_n10.scn-SPT23 THEN 110
110: 0.004854 (1644,2,115,2)

IF MCM1-MCM1 AND MCB-MIG2 THEN 110
110: 0.004854 (1644,2,115,2)

IF m_other_intracellular-transport_activities_n6-UGA3 AND m_amino-acid_degradation_n32-UGA3 THEN 110
110: 0.004854 (1644,2,115,2)

IF MCM1-MCM1 AND MCB-STB4 THEN 110
110: 0.004854 (1644,2,115,2)

IF SFF'-FKH1 AND MCB-YNR063W THEN 110
110: 0.004854 (1644,2,115,2)

IF m_pheromone_response_generation_n12-FKH1 AND ECB-YOX1 THEN 110
110: 0.004854 (1644,2,115,2)

IF m_amino-acid_degradation_n32-UGA3 AND MCB-STB4 THEN 110
110: 0.004854 (1644,2,115,2)

IF m_pheromone_response_generation_n12-FKH1 AND MCB-YNR063W THEN 110
110: 0.004854 (1644,2,115,2)

IF MCM1-MCM1 AND ALPHA1'-STB4 THEN 110
110: 0.004854 (1644,2,115,2)

IF m_pheromone_response_generation_n12-FKH1 AND MCB-MIG2 THEN 110
110: 0.004854 (1644,2,115,2)

IF ECB-DIG1 AND MCB-MBP1 THEN 110
110: 0.004854 (1644,2,115,2)

IF m_cell_rescue_defense_cell_death_and_ageing_n20-PHO4 AND m_other_transport_facilitators_n10-OAF1 THEN 110
110: 0.004854 (1644,2,115,2)

IF m_phosphate_transport_n8-SWI4 AND m_cytoskeleton-dependenttransport_n4-STB1 THEN 110
110: 0.004854 (1644,2,115,2)

IF m_metabolism_of_energy_reserves_n8-PIP2 AND m_phosphate_transport_n8-SWI4 THEN 110
110: 0.004854 (1644,2,115,2)

IF m_trna_transcription_n10-SWI6 AND m_cytoskeleton-dependenttransport_n4-ACE2 THEN 110
110: 0.004854 (1644,2,115,2)

IF m_allantoin_and_allantoate_transporters_n6-PHO4 AND m_other_transport_facilitators_n10-PIP2 THEN 110
110: 0.004854 (1644,2,115,2)

IF m_cytoskeleton-dependenttransport_n4-STB1 AND m_other_transport_facilitators_n10-OAF1 THEN 110
110: 0.004854 (1644,2,115,2)

IF m_cell_rescue_defense_cell_death_and_ageing_n20-ACE2 AND m_cytoskeleton-dependenttransport_n4-ACE2 THEN 110
110: 0.004854 (1644,2,115,2)

IF m_breakdown_of_lipids_fatty_acids_and_isoprenoids_n8-MBP1 AND m_other_transport_facilitators_n10-PIP2 THEN 110
110: 0.004854 (1644,2,115,2)

IF m_trna_transcription_n10-SWI6 AND m_cytoskeleton-dependenttransport_n4-STB1 THEN 110
110: 0.004854 (1644,2,115,2)

IF MCM1-SWI4 AND MCM1'-SWI4 AND m_drug_transporters_n9-SWI4 THEN 110
110: 0.004854 (1644,2,115,2)

IF m_trna_transcription_n10-SWI6 AND m_allantoin_and_allantoate_transporters_n6-PHO4 THEN 110
110: 0.004854 (1644,2,115,2)

IF SFF-FKH2 AND m_allantoin_and_allantoate_transporters_n12-OAF1 THEN 110
110: 0.004854 (1644,2,115,2)

IF m_cytoskeleton-dependenttransport_n4-ACE2 AND m_other_transport_facilitators_n10-PIP2 THEN 110
110: 0.004854 (1644,2,115,2)

IF m_allantoin_and_allantoate_transporters_n13-SWI6 AND mPROTEOL18(m_proteolysis_n18)-MBP1 THEN 110
110: 0.004854 (1644,2,115,2)

IF MCM1-MCM1 AND LYS14-SWI6 THEN 110
110: 0.004854 (1644,2,115,2)

IF SFF'-MCM1 AND LYS14-SWI6 THEN 110
110: 0.004854 (1644,2,115,2)

IF m_glycolysis_and_gluconeogenesis_n11-MBP1 AND m_allantoin_and_allantoate_transporters_n13-SWI6 THEN 110
110: 0.004854 (1644,2,115,2)

IF m_allantoin_and_allantoate_transporters_n13-SWI6 AND mPROTEOL18(m_proteolysis_n18)-SWI6 THEN 110
110: 0.004854 (1644,2,115,2)

IF m_cell_rescue_defense_cell_death_and_ageing_n20-ACE2 AND m_cytoskeleton-dependenttransport_n4-MBP1 THEN 110
110: 0.004854 (1644,2,115,2)

IF SWI5-SWI6 AND m_cell_rescue_defense_cell_death_and_ageing_n20-ACE2 THEN 110
110: 0.004854 (1644,2,115,2)

IF m_allantoin_and_allantoate_transporters_n7-ACE2 AND m_allantoin_and_allantoate_transporters_n12-OAF1 THEN 110
110: 0.004854 (1644,2,115,2)

IF m_allantoin_and_allantoate_transporters_n6-PHO4 AND m_other_transport_facilitators_n10-SWI6 THEN 110
110: 0.004854 (1644,2,115,2)

IF m_cytoskeleton-dependenttransport_n4-ACE2 AND m_cytoskeleton-dependenttransport_n4-STB1 THEN 110
110: 0.004854 (1644,2,115,2)

IF m_trna_transcription_n10-SWI6 AND m_cell_rescue_defense_cell_death_and_ageing_n20-ACE2 THEN 110
110: 0.004854 (1644,2,115,2)

IF m_trna_transcription_n10-SWI6 AND m_allantoin_and_allantoate_transporters_n12-OAF1 THEN 110
110: 0.004854 (1644,2,115,2)

IF m_allantoin_and_allantoate_transporters_n7-ACE2 AND m_other_transport_facilitators_n10-PIP2 THEN 110
110: 0.004854 (1644,2,115,2)

IF m_cell_rescue_defense_cell_death_and_ageing_n20-ACE2 AND m_allantoin_and_allantoate_transporters_n6-PHO4 THEN 110
110: 0.004854 (1644,2,115,2)

IF SFF'-FKH2 AND m_allantoin_and_allantoate_transporters_n12-OAF1 THEN 110
110: 0.004854 (1644,2,115,2)

IF m_cytoskeleton-dependenttransport_n4-STB1 AND m_allantoin_and_allantoate_transporters_n6-PHO4 THEN 110
110: 0.004854 (1644,2,115,2)

IF m_breakdown_of_lipids_fatty_acids_and_isoprenoids_n8-SWI6 AND m_other_transport_facilitators_n10-OAF1 THEN 110
110: 0.004854 (1644,2,115,2)

IF SFF'-FKH2 AND m_allantoin_and_allantoate_transporters_n6-PHO4 THEN 110
110: 0.004854 (1644,2,115,2)

IF m_trna_transcription_n10-SWI6 AND m_allantoin_and_allantoate_transporters_n7-ACE2 THEN 110
110: 0.004854 (1644,2,115,2)

IF m_allantoin_and_allantoate_transporters_n12-ACE2 AND m_allantoin_and_allantoate_transporters_n12-OAF1 THEN 110
110: 0.004854 (1644,2,115,2)

IF m_phosphate_transport_n8-SWI4 AND m_allantoin_and_allantoate_transporters_n6-PHO4 THEN 110
110: 0.004854 (1644,2,115,2)

IF m_breakdown_of_lipids_fatty_acids_and_isoprenoids_n8-MBP1 AND m_cell_rescue_defense_cell_death_and_ageing_n20-PHO4 THEN 110
110: 0.004854 (1644,2,115,2)

IF m_metabolism_of_energy_reserves_n8-PIP2 AND m_cell_rescue_defense_cell_death_and_ageing_n20-ACE2 THEN 110
110: 0.004854 (1644,2,115,2)

IF m_phosphate_transport_n8-SWI4 AND m_allantoin_and_allantoate_transporters_n12-OAF1 THEN 110
110: 0.004854 (1644,2,115,2)

IF m_cell_rescue_defense_cell_death_and_ageing_n20-ACE2 AND m_allantoin_and_allantoate_transporters_n12-ACE2 THEN 110
110: 0.004854 (1644,2,115,2)

IF SWI5-SWI6 AND m_allantoin_and_allantoate_transporters_n12-OAF1 THEN 110
110: 0.004854 (1644,2,115,2)

IF m_breakdown_of_lipids_fatty_acids_and_isoprenoids_n8-MBP1 AND m_cell_rescue_defense_cell_death_and_ageing_n20-ACE2 THEN 110
110: 0.004854 (1644,2,115,2)

IF m_breakdown_of_lipids_fatty_acids_and_isoprenoids_n8-SWI6 AND m_cell_rescue_defense_cell_death_and_ageing_n20-ACE2 THEN 110
110: 0.004854 (1644,2,115,2)

IF m_breakdown_of_lipids_fatty_acids_and_isoprenoids_n8-MBP1 AND m_cytoskeleton-dependenttransport_n4-STB1 THEN 110
110: 0.004854 (1644,2,115,2)

IF m_metabolism_of_energy_reserves_n8-PIP2 AND m_cytoskeleton-dependenttransport_n4-STB1 THEN 110
110: 0.004854 (1644,2,115,2)

IF MCM1'-MCM1 AND SCB-UME6 THEN 110
110: 0.004854 (1644,2,115,2)

IF ECB-MCM1 AND SCB-UME6 THEN 110
110: 0.004854 (1644,2,115,2)

IF MCM1-NDD1 AND LYS14-SWI6 THEN 110
110: 0.004854 (1644,2,115,2)

IF SFF'-FKH2 AND m_other_proteolytic_degradation_n7-SWI6 THEN 110
110: 0.004854 (1644,2,115,2)

IF MCM1-FKH2 AND LYS14-SWI6 THEN 110
110: 0.004854 (1644,2,115,2)

IF m_other_proteolytic_degradation_n7-SWI6 AND SCB-SWI4 THEN 110
110: 0.004854 (1644,2,115,2)

IF m_other_proteolytic_degradation_n7-SWI6 AND m_nucleotide_transport_n9-MCM1 THEN 110
110: 0.004854 (1644,2,115,2)

IF MCM1-NDD1 AND SCB-UME6 THEN 110

110: 0.004854 (1644,2,115,2)

IF SFF'-MCM1 AND m_other_proteolytic_degradation_n7-SWI6 THEN 110
110: 0.004854 (1644,2,115,2)

IF m_breakdown_of_lipids_fatty_acids_and_isoprenoids_n8-SWI6 AND m_trna_transcription_n10-SWI6 THEN 110
110: 0.004854 (1644,2,115,2)

IF m_cell_rescue_defense_cell_death_and_ageing_n20-ACE2 AND m_allantoin_and_allantoate_transporters_n12-OAF1 THEN 110
110: 0.004854 (1644,2,115,2)

IF SFF'-FKH2 AND m_cell_rescue_defense_cell_death_and_ageing_n20-ACE2 THEN 110
110: 0.004854 (1644,2,115,2)

IF m_allantoin_and_allantoate_transporters_n7-ACE2 AND m_cell_rescue_defense_cell_death_and_ageing_n20-ACE2 THEN 110
110: 0.004854 (1644,2,115,2)

IF m_phosphate_transport_n8-SWI4 AND m_cell_rescue_defense_cell_death_and_ageing_n20-PHO4 THEN 110
110: 0.004854 (1644,2,115,2)

IF m_phosphate_transport_n8-SWI4 AND m_cell_rescue_defense_cell_death_and_ageing_n20-ACE2 THEN 110
110: 0.004854 (1644,2,115,2)

IF m_cell_rescue_defense_cell_death_and_ageing_n20-PHO4 AND m_other_transport_facilitators_n10-SWI6 THEN 110
110: 0.004854 (1644,2,115,2)

IF m_cell_rescue_defense_cell_death_and_ageing_n20-ACE2 AND m_other_transport_facilitators_n10-SWI6 THEN 110
110: 0.004854 (1644,2,115,2)

IF m_cytoskeleton-dependenttransport_n4-STB1 AND m_allantoin_and_allantoate_transporters_n12-OAF1 THEN 110
110: 0.004854 (1644,2,115,2)

IF m_cell_rescue_defense_cell_death_and_ageing_n20-ACE2 AND m_cytoskeleton-dependenttransport_n4-STB1 THEN 110
110: 0.004854 (1644,2,115,2)

IF m_metabolism_of_energy_reserves_n8-PIP2 AND m_allantoin_and_allantoate_transporters_n12-ACE2 THEN 110
110: 0.004854 (1644,2,115,2)

IF m_cell_rescue_defense_cell_death_and_ageing_n20-PHO4 AND m_other_transport_facilitators_n10-PIP2 THEN 110
110: 0.004854 (1644,2,115,2)

IF m_cell_rescue_defense_cell_death_and_ageing_n20-PHO4 AND m_allantoin_and_allantoate_transporters_n12-ACE2 THEN 110
110: 0.004854 (1644,2,115,2)

IF m_cell_rescue_defense_cell_death_and_ageing_n20-PHO4 AND m_allantoin_and_allantoate_transporters_n6-PHO4 THEN 110
110: 0.004854 (1644,2,115,2)

IF SFF-FKH2 AND m_cell_rescue_defense_cell_death_and_ageing_n20-ACE2 THEN 110
110: 0.004854 (1644,2,115,2)

IF m_breakdown_of_lipids_fatty_acids_and_isoprenoids_n8-MBP1 AND m_allantoin_and_allantoate_transporters_n12-OAF1 THEN 110
110: 0.004854 (1644,2,115,2)

IF m_breakdown_of_lipids_fatty_acids_and_isoprenoids_n8-SWI6 AND m_allantoin_and_allantoate_transporters_n12-OAF1 THEN 110
110: 0.004854 (1644,2,115,2)

IF m_phosphate_transport_n8-SWI4 AND m_other_transport_facilitators_n10-OAF1 THEN 110
110: 0.004854 (1644,2,115,2)

IF m_allantoin_and_allantoate_transporters_n6-PHO4 AND m_allantoin_and_allantoate_transporters_n12-OAF1 THEN 110
110: 0.004854 (1644,2,115,2)

IF m_trna_transcription_n10-SWI6 AND m_allantoin_and_allantoate_transporters_n12-ACE2 THEN 110
110: 0.004854 (1644,2,115,2)

IF m_cytoskeleton-dependenttransport_n4-MBP1 AND m_allantoin_and_allantoate_transporters_n12-OAF1 THEN 110
110: 0.004854 (1644,2,115,2)

IF m_cytoskeleton-dependenttransport_n4-MBP1 AND m_other_transport_facilitators_n10-OAF1 THEN 110
110: 0.004854 (1644,2,115,2)

IF m_breakdown_of_lipids_fatty_acids_and_isoprenoids_n8-SWI6 AND m_allantoin_and_allantoate_transporters_n12-ACE2 THEN 110
110: 0.004854 (1644,2,115,2)

IF m_allantoin_and_allantoate_transporters_n7-ACE2 AND m_cytoskeleton-dependenttransport_n4-STB1 THEN 110
110: 0.004854 (1644,2,115,2)

IF m_cell_rescue_defense_cell_death_and_ageing_n20-PHO4 AND m_allantoin_and_allantoate_transporters_n12-OAF1 THEN 110
110: 0.004854 (1644,2,115,2)

IF SFF-FKH2 AND m_other_transport_facilitators_n10-OAF1 THEN 110
110: 0.004854 (1644,2,115,2)

IF m_cell_rescue_defense_cell_death_and_ageing_n20-ACE2 AND m_other_transport_facilitators_n10-OAF1 THEN 110
110: 0.004854 (1644,2,115,2)

IF SFF'-FKH2 AND m_trna_transcription_n10-SWI6 THEN 110
110: 0.004854 (1644,2,115,2)

IF SWI5-SWI6 AND m_cell_rescue_defense_cell_death_and_ageing_n20-PHO4 THEN 110
110: 0.004854 (1644,2,115,2)

IF m_allantoin_and_allantoate_transporters_n7-ACE2 AND m_cell_rescue_defense_cell_death_and_ageing_n20-PHO4 THEN 110
110: 0.004854 (1644,2,115,2)

IF m_breakdown_of_lipids_fatty_acids_and_isoprenoids_n8-SWI6 AND m_cytoskeleton-dependenttransport_n4-STB1 THEN 110
110: 0.004854 (1644,2,115,2)

IF m_cytoskeleton-dependenttransport_n4-ACE2 AND m_allantoin_and_allantoate_transporters_n12-ACE2 THEN 110
110: 0.004854 (1644,2,115,2)

IF m_trna_transcription_n10-SWI6 AND m_cell_rescue_defense_cell_death_and_ageing_n20-PHO4 THEN 110
110: 0.004854 (1644,2,115,2)

IF m_breakdown_of_lipids_fatty_acids_and_isoprenoids_n8-MBP1 AND m_allantoin_and_allantoate_transporters_n12-ACE2 THEN 110
110: 0.004854 (1644,2,115,2)

IF m_cytoskeleton-dependenttransport_n4-STB1 AND m_allantoin_and_allantoate_transporters_n12-ACE2 THEN 110
110: 0.004854 (1644,2,115,2)

IF m_cytoskeleton-dependenttransport_n4-ACE2 AND m_allantoin_and_allantoate_transporters_n12-OAF1 THEN 110
110: 0.004854 (1644,2,115,2)

IF m_allantoin_and_allantoate_transporters_n12-ACE2 AND m_other_transport_facilitators_n10-OAF1 THEN 110
110: 0.004854 (1644,2,115,2)

IF m_cell_rescue_defense_cell_death_and_ageing_n20-PHO4 AND m_cytoskeleton-dependenttransport_n4-STB1 THEN 110
110: 0.004854 (1644,2,115,2)

IF m_breakdown_of_lipids_fatty_acids_and_isoprenoids_n8-SWI6 AND m_allantoin_and_allantoate_transporters_n6-PHO4 THEN 110
110: 0.004854 (1644,2,115,2)

IF SWI5-SWI6 AND m_allantoin_and_allantoate_transporters_n6-PHO4 THEN 110
110: 0.004854 (1644,2,115,2)

IF m_allantoin_and_allantoate_transporters_n6-PHO4 AND m_allantoin_and_allantoate_transporters_n12-ACE2 THEN 110
110: 0.004854 (1644,2,115,2)

IF m_cytoskeleton-dependenttransport_n4-MBP1 AND m_allantoin_and_allantoate_transporters_n12-ACE2 THEN 110
110: 0.004854 (1644,2,115,2)

IF m_metabolism_of_energy_reserves_n8-PIP2 AND m_allantoin_and_allantoate_transporters_n7-ACE2 THEN 110
110: 0.004854 (1644,2,115,2)

IF m_trna_transcription_n10-SWI6 AND m_metabolism_of_energy_reserves_n8-PIP2 THEN 110
110: 0.004854 (1644,2,115,2)

IF m_cytoskeleton-dependenttransport_n4-ACE2 AND m_other_transport_facilitators_n10-OAF1 THEN 110
110: 0.004854 (1644,2,115,2)

IF m_trna_transcription_n10-SWI6 AND m_other_transport_facilitators_n10-OAF1 THEN 110
110: 0.004854 (1644,2,115,2)

IF SFF-FKH2 AND m_other_transport_facilitators_n10-PIP2 THEN 110
110: 0.004854 (1644,2,115,2)

IF m_allantoin_and_allantoate_transporters_n7-ACE2 AND m_other_transport_facilitators_n10-OAF1 THEN 110
110: 0.004854 (1644,2,115,2)

IF m_cell_rescue_defense_cell_death_and_ageing_n20-PHO4 AND m_cytoskeleton-dependenttransport_n4-MBP1 THEN 110
110: 0.004854 (1644,2,115,2)

IF m_cytoskeleton-dependenttransport_n4-ACE2 AND m_other_transport_facilitators_n10-SWI6 THEN 110
110: 0.004854 (1644,2,115,2)

IF m_cell_rescue_defense_cell_death_and_ageing_n20-ACE2 AND m_other_transport_facilitators_n10-PIP2 THEN 110
110: 0.004854 (1644,2,115,2)

IF m_metabolism_of_energy_reserves_n8-PIP2 AND m_cytoskeleton-dependenttransport_n4-ACE2 THEN 110
110: 0.004854 (1644,2,115,2)

IF m_trna_transcription_n10-SWI6 AND m_cytoskeleton-dependenttransport_n4-MBP1 THEN 110
110: 0.004854 (1644,2,115,2)

IF m_metabolism_of_energy_reserves_n8-PIP2 AND m_cell_rescue_defense_cell_death_and_ageing_n20-PHO4 THEN 110
110: 0.004854 (1644,2,115,2)

IF m_breakdown_of_lipids_fatty_acids_and_isoprenoids_n8-MBP1 AND m_metabolism_of_energy_reserves_n8-PIP2 THEN 110
110: 0.004854 (1644,2,115,2)

IF SFF'-FKH2 AND m_metabolism_of_energy_reserves_n8-PIP2 THEN 110
110: 0.004854 (1644,2,115,2)

IF STRE'-MBP1 AND MCB-SWI6 THEN 110
110: 0.004854 (1644,2,115,2)

IF m_chromatin_modification_n9-MBP1 AND MCB-MBP1 THEN 110
110: 0.004854 (1644,2,115,2)

IF STRE'-MBP1 AND MCB-MBP1 THEN 110
110: 0.004854 (1644,2,115,2)

IF m_breakdown_of_lipids_fatty_acids_and_isoprenoids_n8-MBP1 AND m_other_transport_facilitators_n10-OAF1 THEN 110
110: 0.004854 (1644,2,115,2)

IF m_cytoskeleton-dependenttransport_n4-STB1 AND m_other_transport_facilitators_n10-PIP2 THEN 110
110: 0.004854 (1644,2,115,2)

IF SWI5-SWI6 AND m_other_transport_facilitators_n10-OAF1 THEN 110
110: 0.004854 (1644,2,115,2)

IF m_metabolism_of_energy_reserves_n8-PIP2 AND SWI5-SWI6 THEN 110
110: 0.004854 (1644,2,115,2)

IF m_allantoin_and_allantoate_transporters_n12-ACE2 AND m_other_transport_facilitators_n10-PIP2 THEN 110
110: 0.004854 (1644,2,115,2)

IF m_pentose-phosphate_pathway_n21-MAL33 AND m_pentose-phosphate_pathway_n14-MTH1 THEN 110
110: 0.004854 (1644,2,115,2)

IF m_phosphate_transport_n13-GAT1 AND m_pentose-phosphate_pathway_n14-FKH1 THEN 110
110: 0.004854 (1644,2,115,2)

IF m_pentose-phosphate_pathway_n21-GAT1 AND m_MERE4-MTH1 THEN 110
110: 0.004854 (1644,2,115,2)

IF m_pentose-phosphate_pathway_n21-MAL33 AND m_MERE4-MTH1 THEN 110
110: 0.004854 (1644,2,115,2)

IF m_pentose-phosphate_pathway_n21-GAT1 AND m_pentose-phosphate_pathway_n14-MTH1 THEN 110

110: 0.004854 (1644,2,115,2)

IF m_ionic_homeostasis_n6-SWI6 AND m_biosynthesis_of_vitamins_cofactors_and_prosthetic_groups_n8-MET4 THEN 110
110: 0.004854 (1644,2,115,2)

IF ECB-TEC1 AND mRRPE-IXR1 THEN 110
110: 0.004854 (1644,2,115,2)

IF m_g-proteins_n12-SKN7 AND m_lipid_transporters_n8-MET4 THEN 110
110: 0.004854 (1644,2,115,2)

IF MCM1'-NDD1 AND m_biosynthesis_of_vitamins_cofactors_and_prosthetic_groups_n8-MET4 THEN 110
110: 0.004854 (1644,2,115,2)

IF SFF'-FKH1 AND mRRPE-IXR1 THEN 110
110: 0.004854 (1644,2,115,2)

IF m_drug_transporters_n10-INO4 AND mRRPE-RLM1 THEN 110
110: 0.004854 (1644,2,115,2)

IF MCM1'-NDD1 AND m_drug_transporters_n10-INO4 THEN 110
110: 0.004854 (1644,2,115,2)

IF SFF-FKH1 AND m_drug_transporters_n10-INO4 THEN 110
110: 0.004854 (1644,2,115,2)

IF SFF'-FKH2 AND m_drug_transporters_n10-INO4 THEN 110
110: 0.004854 (1644,2,115,2)

IF SFF'-FKH2 AND ECB-STE12 THEN 110
110: 0.004854 (1644,2,115,2)

IF ECB-TEC1 AND mRRPE-RLM1 THEN 110
110: 0.004854 (1644,2,115,2)

IF SFF'-FKH2 AND ECB-TEC1 THEN 110
110: 0.004854 (1644,2,115,2)

IF m_osmosensing_n6-SWI4 AND mRRPE-RLM1 THEN 110
110: 0.004854 (1644,2,115,2)

IF SFF'-RLM1 AND m_drug_transporters_n10-SKN7 THEN 110
110: 0.004854 (1644,2,115,2)

IF SFF-FKH2 AND m_drug_transporters_n10-INO4 THEN 110
110: 0.004854 (1644,2,115,2)

IF ECB-FKH2 AND mRRPE-RLM1 THEN 110
110: 0.004854 (1644,2,115,2)

IF SFF'-RLM1 AND m_drug_transporters_n10-INO4 THEN 110
110: 0.004854 (1644,2,115,2)

IF m_drug_transporters_n10-SKN7 AND mRRPE-RLM1 THEN 110
110: 0.004854 (1644,2,115,2)

IF m_osmosensing_n6-SWI4 AND ECB-TEC1 THEN 110
110: 0.004854 (1644,2,115,2)

IF ECB-NDD1 AND mRRPE-RLM1 THEN 110
110: 0.004854 (1644,2,115,2)

IF m_osmosensing_n6-SWI4 AND ECB-STE12 THEN 110
110: 0.004854 (1644,2,115,2)

IF m_osmosensing_n6-INO4 AND ECB-STE12 THEN 110
110: 0.004854 (1644,2,115,2)

IF m_osmosensing_n6-INO4 AND ECB-TEC1 THEN 110
110: 0.004854 (1644,2,115,2)

IF m_osmosensing_n6-INO4 AND mRRPE-RLM1 THEN 110
110: 0.004854 (1644,2,115,2)

IF ECB-MCM1 AND mRRPE-IXR1 THEN 110
110: 0.004854 (1644,2,115,2)

IF m_osmosensing_n6-SWI4 AND m_drug_transporters_n10-SKN7 THEN 110
110: 0.004854 (1644,2,115,2)

IF ECB-TEC1 AND STE12-TEC1 THEN 110
110: 0.004854 (1644,2,115,2)

IF m_g-proteins_n12-SKN7 AND m_sugar_and_carbohydrate_transporters_n6-SKN7 AND m_other_protein-destination_activities_n7-MET4 THEN 110
110: 0.004854 (1644,2,115,2)

IF m_organization_of_intracellular_transport_vesicles_n5-SWI4 AND ECB-TEC1 THEN 110
110: 0.004854 (1644,2,115,2)

IF MCM1'-SWI4 AND m_phosphate_transport_n8-SWI5 THEN 110
110: 0.004854 (1644,2,115,2)

IF SFF'-MCM1 AND mRRPE-IXR1 THEN 110
110: 0.004854 (1644,2,115,2)

IF m_osmosensing_n6-INO4 AND m_stress_response_n24-SWI4 THEN 110
110: 0.004854 (1644,2,115,2)

IF m_organization_of_intracellular_transport_vesicles_n5-SWI4 AND ECB-STE12 THEN 110
110: 0.004854 (1644,2,115,2)

IF ECB-TEC1 AND STE12-DIG1 THEN 110
110: 0.004854 (1644,2,115,2)

IF SFF'-FKH1 AND ECB-TEC1 THEN 110
110: 0.004854 (1644,2,115,2)

IF m_g-proteins_n12-INO4 AND m_stress_response_n24-SWI4 THEN 110
110: 0.004854 (1644,2,115,2)

IF SFF'-FKH2 AND m_abc_transporters_n10-MBP1 THEN 110
110: 0.004854 (1644,2,115,2)

IF m_abc_transporters_n10-MBP1 AND SFF-FKH2 THEN 110
110: 0.004854 (1644,2,115,2)

IF m_allantoin_and_allantoate_transporters_n18-FKH2 AND LYS14-SWI6 THEN 110
110: 0.004854 (1644,2,115,2)

IF ECB-STE12 AND STE12-TEC1 THEN 110
110: 0.004854 (1644,2,115,2)

IF ECB-TEC1 AND STE12-STE12 THEN 110
110: 0.004854 (1644,2,115,2)

IF ECB-STE12 AND mRRPE-IXR1 THEN 110
110: 0.004854 (1644,2,115,2)

IF ECB-NDD1 AND SFF-FKH2 AND m_other_morphogenetic_activities_n7-MCM1 THEN 110
110: 0.004854 (1644,2,115,2)

IF SFF-FKH1 AND mRRPE-IXR1 THEN 110
110: 0.004854 (1644,2,115,2)

IF m_allantoin_and_allantoate_transporters_n18-FKH2 AND ECB-NDD1 AND SFF-FKH2 THEN 110
110: 0.004854 (1644,2,115,2)

IF m_allantoin_and_allantoate_transporters_n18-FKH2 AND ECB-FKH2 AND SFF-FKH2 THEN 110
110: 0.004854 (1644,2,115,2)

IF ECB-FKH2 AND SFF-FKH2 AND m_other_morphogenetic_activities_n7-MCM1 THEN 110
110: 0.004854 (1644,2,115,2)

IF SFF-FKH2 AND m_nucleotide_transport_n9-MCM1 THEN 110
110: 0.004854 (1644,2,115,2)

IF ECB-MCM1 AND SFF-FKH2 AND m_other_morphogenetic_activities_n7-MCM1 THEN 110
110: 0.004854 (1644,2,115,2)

IF m_pentose-phosphate_pathway_n7-GAT1 AND m_pentose-phosphate_pathway_n7-MTH1 THEN 110
110: 0.004854 (1644,2,115,2)

IF SFF-FKH1 AND m_anion_transporters_n20-GAT1 THEN 110
110: 0.004854 (1644,2,115,2)

IF SFF'-FKH2 AND MCM1'-SWI4 AND LYS14-SWI6 THEN 110
110: 0.004854 (1644,2,115,2)

IF SFF'-FKH2 AND MCM1'-SWI4 AND m_ionic_homeostasis_n6-SWI6 THEN 110
110: 0.004854 (1644,2,115,2)

IF SFF'-FKH2 AND MCM1'-SWI4 AND ALPHA1'-UPC2 THEN 110
110: 0.004854 (1644,2,115,2)

IF SFF'-FKH1 AND MCM1'-SWI4 AND ALPHA1'-UPC2 THEN 110
110: 0.004854 (1644,2,115,2)

IF SFF-FKH2 AND LYS14-SWI6 AND m_ionic_homeostasis_n6-SWI6 THEN 110
110: 0.004854 (1644,2,115,2)

IF m_other_energy_generation_activities_n17-TEC1 AND SFF-FKH2 THEN 110
110: 0.004854 (1644,2,115,2)

IF m_other_energy_generation_activities_n17-TEC1 AND SFF-FKH1 THEN 110
110: 0.004854 (1644,2,115,2)

IF SFF'-FKH2 AND m_regulation_of_amino-acid_metabolism_n11-SWI6 THEN 110
110: 0.004854 (1644,2,115,2)

IF m_anion_transporters_n15-MTH1 AND m_nucleotide_transport_n9-SWI6 THEN 110
110: 0.004854 (1644,2,115,2)

IF SFF'-FKH1 AND m_OCSE15-MTH1 THEN 110
110: 0.004854 (1644,2,115,2)

IF MCM1'-MCM1 AND mRRPE-IXR1 THEN 110
110: 0.004854 (1644,2,115,2)

IF m_osmosensing_n6-ROX1 AND m_organization_of_golgi_n7-FKH1 THEN 110
110: 0.004854 (1644,2,115,2)

IF SWI5-SWI6 AND m_pentose-phosphate_pathway_n7-GAT1 THEN 110
110: 0.004854 (1644,2,115,2)

IF SFF'-FKH1 AND m_anion_transporters_n20-GAT1 THEN 110
110: 0.004854 (1644,2,115,2)

IF SFF-FKH2 AND m_regulation_of_amino-acid_metabolism_n11-SWI6 THEN 110
110: 0.004854 (1644,2,115,2)

IF m_abc_transporters_n10-MBP1 AND m_allantoin_and_allantoate_transporters_n13-SWI6 THEN 110
110: 0.004854 (1644,2,115,2)

IF m_abc_transporters_n10-MBP1 AND m_glyoxylate_cycle_n19-SWI6 THEN 110
110: 0.004854 (1644,2,115,2)

IF m_metal_ion_transporters_n14-GAT1 AND m_pentose-phosphate_pathway_n7-MTH1 THEN 110
110: 0.004854 (1644,2,115,2)

IF m_LFTE17-GAT1 AND m_pentose-phosphate_pathway_n7-MTH1 THEN 110
110: 0.004854 (1644,2,115,2)

IF SFF-FKH2 AND m_nucleotide_transport_n9-SWI6 THEN 110
110: 0.004854 (1644,2,115,2)

IF MCM1'-SWI4 AND SFF-FKH2 AND m_ionic_homeostasis_n6-SWI6 THEN 110
110: 0.004854 (1644,2,115,2)

IF MCM1'-SWI4 AND SFF-FKH1 AND ALPHA1'-UPC2 THEN 110
110: 0.004854 (1644,2,115,2)

IF MCM1'-SWI4 AND SFF-FKH2 AND LYS14-SWI6 THEN 110
110: 0.004854 (1644,2,115,2)

IF MCM1'-SWI4 AND SFF-FKH2 AND ALPHA1'-UPC2 THEN 110
110: 0.004854 (1644,2,115,2)

IF SFF'-FKH2 AND LYS14-SWI6 AND m_ionic_homeostasis_n6-SWI6 THEN 110
110: 0.004854 (1644,2,115,2)

IF m_metal_ion_transporters_n14-GAT1 AND m_pentose-phosphate_pathway_n7-GAT1 THEN 110
110: 0.004854 (1644,2,115,2)

IF m_regulation_of_nitrogen_and_sulphur_utilization_n13-MTH1 AND m_pentose-phosphate_pathway_n7-GAT1 THEN 110
110: 0.004854 (1644,2,115,2)

IF SFF-FKH1 AND m_OCSE15-MTH1 THEN 110
110: 0.004854 (1644,2,115,2)

IF m_regulation_of_nitrogen_and_sulphur_utilization_n13-MTH1 AND m_metal_ion_transporters_n14-GAT1 THEN 110
110: 0.004854 (1644,2,115,2)

IF SWI5-SWI6 AND m_pentose-phosphate_pathway_n7-MTH1 THEN 110
110: 0.004854 (1644,2,115,2)

IF m_OCSE15-MTH1 AND m_anion_transporters_n20-GAT1 THEN 110
110: 0.004854 (1644,2,115,2)

IF m_LFTE17-GAT1 AND m_OCSE15-MTH1 THEN 110
110: 0.004854 (1644,2,115,2)

IF m_OCSE15-MTH1 AND m_anion_transporters_n20-MTH1 THEN 110
110: 0.004854 (1644,2,115,2)

IF ECB-DIG1 AND STE12-TEC1 THEN 110
110: 0.004854 (1644,2,115,2)

IF SFF-HIR1 AND SCB-SWI4 THEN 110
110: 0.004854 (1644,2,115,2)

IF m_abc_transporters_n10-SWI6 AND m_glyoxylate_cycle_n19-SWI6 THEN 110
110: 0.004854 (1644,2,115,2)

IF SFF-RPI1 AND MCB-YNR063W THEN 110
110: 0.004854 (1644,2,115,2)

IF m_nutritional_response_pathway_n7-ACE2 AND MCB-MBP1 THEN 110
110: 0.004854 (1644,2,115,2)

IF m_abc_transporters_n10-SWI6 AND m_allantoin_and_allantoate_transporters_n13-SWI6 THEN 110
110: 0.004854 (1644,2,115,2)

IF m_allantoin_and_allantoate_transporters_n7-ACE2 AND m_biogenesis_of_chromosome_structure_n18-YDR049W THEN 110
110: 0.004854 (1644,2,115,2)

IF SFF-RPI1 AND MCB-YFL052w THEN 110
110: 0.004854 (1644,2,115,2)

IF m_nutritional_response_pathway_n7-ACE2 AND MCB-SWI6 THEN 110

110: 0.004854 (1644,2,115,2)

IF SFF'-HIR1 AND SCB-SWI4 THEN 110
110: 0.004854 (1644,2,115,2)

IF m_breakdown_of_lipids_fatty_acids_and_isoprenoids_n8-INO4 AND m_LFTE17-GAT1 THEN 110
110: 0.004854 (1644,2,115,2)

IF m_g-proteins_n11-INO4 AND m_LFTE17-GAT1 THEN 110
110: 0.004854 (1644,2,115,2)

IF m_other_morphogenetic_activities_n7-ARR1 AND m_other_morphogenetic_activities_n7-HOG1 AND m_other_morphogenetic_activities_n7-RTG1 THEN 110
110: 0.004854 (1644,2,115,2)

IF m_lipid_and_fatty-acid_transport_n11-SWI5 AND ALPHA1'-RIM101 THEN 110
110: 0.004854 (1644,2,115,2)

IF SWI5-SWI6 AND m_amino-acid_transporters_n11-SKN7 AND m_nucleotide_transport_n9-SWI6 THEN 110
110: 0.004854 (1644,2,115,2)

IF SWI5-SWI6 AND LYS14-SWI6 AND m_nitrogen_and_sulphur_metabolism_n16-SWI6 THEN 110
110: 0.004854 (1644,2,115,2)

IF m_osmosensing_n6-SWI4 AND m_stress_response_n24-SWI4 THEN 110
110: 0.004854 (1644,2,115,2)

IF m_osmosensing_n6-INO4 AND m_amino-acid_transporters_n11-SKN7 THEN 110
110: 0.004854 (1644,2,115,2)

IF SFF'-FKH1 AND m_g-proteins_n12-INO4 AND MCM1'-NDD1 THEN 110
110: 0.004854 (1644,2,115,2)

IF m_g-proteins_n12-INO4 AND MCM1'-NDD1 AND SFF-FKH2 THEN 110
110: 0.004854 (1644,2,115,2)

IF m_g-proteins_n12-INO4 AND MCM1'-NDD1 AND SFF-FKH1 THEN 110
110: 0.004854 (1644,2,115,2)

IF m_osmosensing_n6-INO4 AND m_lipid_and_fatty-acid_transport_n11-MTH1 THEN 110
110: 0.004854 (1644,2,115,2)

IF m_nitrogen_and_sulphur_metabolism_n17-SKN7 AND m_g-proteins_n11-INO4 THEN 110
110: 0.004854 (1644,2,115,2)

IF m_nitrogen_and_sulphur_metabolism_n17-SKN7 AND m_other_mrna-transcription_activities_n20-INO4 THEN 110
110: 0.004854 (1644,2,115,2)

IF m_other_mrna-transcription_activities_n20-INO4 AND m_pentose-phosphate_pathway_n14-MTH1 THEN 110
110: 0.004854 (1644,2,115,2)

IF m_other_transport_facilitators_n5-CIN5 AND m_other_transport_facilitators_n5-SWI6 THEN 110
110: 0.004854 (1644,2,115,2)

IF m_lipid_and_fatty-acid_transport_n11-MTH1 AND m_nucleotide_transport_n9-SWI6 THEN 110
110: 0.004854 (1644,2,115,2)

IF m_glyoxylate_cycle_n8-INO4 AND m_other_mrna-transcription_activities_n20-XBP1 THEN 110
110: 0.004854 (1644,2,115,2)

IF m_other_signal-transduction_activities_n8-INO4 AND m_other_mrna-transcription_activities_n20-XBP1 THEN 110
110: 0.004854 (1644,2,115,2)

IF m_g-proteins_n11-INO4 AND m_other_transport_facilitators_n5-CIN5 THEN 110
110: 0.004854 (1644,2,115,2)

IF LYS14-SWI6 AND m_pentose-phosphate_pathway_n14-FKH1 AND SCB-UME6 THEN 110
110: 0.004854 (1644,2,115,2)

IF m_other_transport_facilitators_n5-SWI6 AND m_pentose-phosphate_pathway_n14-FKH1 THEN 110

110: 0.004854 (1644,2,115,2)

IF SWI5-SWI6 AND m_other_transport_facilitators_n5-CIN5 THEN 110
110: 0.004854 (1644,2,115,2)

IF m_other_signal-transduction_activities_n8-INO4 AND MCB-SWI6 THEN 110
110: 0.004854 (1644,2,115,2)

IF SFF'-FKH1 AND LYS14-SWI6 AND SCB-UME6 THEN 110
110: 0.004854 (1644,2,115,2)

IF SFF'-FKH2 AND LYS14-SWI6 AND SCB-UME6 THEN 110
110: 0.004854 (1644,2,115,2)

IF m_nitrogen_and_sulphur_metabolism_n17-SKN7 AND m_osmosensing_n6-INO4 THEN 110
110: 0.004854 (1644,2,115,2)

IF m_deoxyribonucleotide_metabolism_n23-MTH1 AND m_organization_of_plasma_membrane_n17-INO4 THEN 110
110: 0.004854 (1644,2,115,2)

IF m_nitrogen_and_sulphur_metabolism_n17-SKN7 AND m_lipid_and_fatty-acid_transport_n11-SKN7 THEN 110
110: 0.004854 (1644,2,115,2)

IF m_nitrogen_and_sulphur_metabolism_n17-XBP1 AND m_amino-acid_metabolism_n25-SWI6 THEN 110
110: 0.004854 (1644,2,115,2)

IF m_lipid_and_fatty-acid_transport_n11-MTH1 AND m_allantoin_and_allantoate_transporters_n13-SWI6 THEN 110
110: 0.004854 (1644,2,115,2)

IF m_other_mrna-transcription_activities_n20-INO4 AND m_other_transport_facilitators_n5-CIN5 THEN 110
110: 0.004854 (1644,2,115,2)

IF m_nitrogen_and_sulphur_metabolism_n17-SKN7 AND m_lipid_and_fatty-acid_transport_n11-MTH1 THEN 110
110: 0.004854 (1644,2,115,2)

IF m_lipid_and_fatty-acid_transport_n11-MTH1 AND m_other_mrna-transcription_activities_n20-INO4 THEN 110
110: 0.004854 (1644,2,115,2)

IF m_g-proteins_n12-SKN7 AND m_amino-acid_metabolism_n25-SWI6 THEN 110
110: 0.004854 (1644,2,115,2)

IF m_g-proteins_n11-INO4 AND m_lipid_and_fatty-acid_transport_n11-MTH1 THEN 110
110: 0.004854 (1644,2,115,2)

IF m_other_signal-transduction_activities_n8-INO4 AND m_glyoxylate_cycle_n8-INO4 AND m_homeostasis_of_other_ions_n30-INO4 THEN 110
110: 0.004854 (1644,2,115,2)

IF m_other_signal-transduction_activities_n8-INO4 AND m_g-proteins_n11-INO4 AND m_glyoxylate_cycle_n8-INO4 THEN 110
110: 0.004854 (1644,2,115,2)

IF m_other_signal-transduction_activities_n8-INO4 AND m_phosphate_transport_n18-INO4 AND m_homeostasis_of_other_ions_n30-INO4 THEN 110
110: 0.004854 (1644,2,115,2)

IF m_other_signal-transduction_activities_n8-INO4 AND m_glyoxylate_cycle_n8-INO4 AND m_phosphate_transport_n18-INO4 THEN 110
110: 0.004854 (1644,2,115,2)

IF m_g-proteins_n12-INO4 AND m_g-proteins_n11-INO4 AND m_other_mrna-transcription_activities_n20-XBP1 THEN 110
110: 0.004854 (1644,2,115,2)

IF SFF'-FKH2 AND ALPHA1'-UPC2 THEN 000 OR 010 OR 110
110: 0.005107 (1644,11,115,4)

IF MCM1-FKH2 AND SFF-FKH1 THEN 000 OR 100 OR 110 OR 111
110: 0.005107 (1644,11,115,4)

IF SCB-SWI4 THEN 000 OR 010 OR 100 OR 110 OR 111
110: 0.005195 (1644,40,115,8)

IF m_peroxisomal_transport_n22-RAP1 AND RAP1-FHL1 THEN 000 OR 010

010: 0.005322 (1644,5,140,3)

IF RAP1-RAP1 AND ALPHA1-DAT1 THEN 000 OR 010
010: 0.005322 (1644,5,140,3)

IF m_other_mrna-transcription_activities_n11-SWI6 AND m_other_pheromone_response_activities_n8-SWI6 THEN 000 OR 010 OR 110
010: 0.005322 (1644,5,140,3)

IF m_deoxyribonucleotide_metabolism_n23-MTH1 AND m_regulation_of_lipid_fatty-acid_and_isoprenoid_biosynthesis_n8.scn-MTH1 THEN 000 OR 010
010: 0.005322 (1644,5,140,3)

IF m_utilization_of_vitamins_cofactors_and_prosthetic_groups_n7-HSF1 THEN 000 OR 010 OR 110
010: 0.005322 (1644,5,140,3)

IF SFF-FKH1 AND SWI5-SWI6 THEN 000 OR 100 OR 110 OR 111
111: 0.005391 (1644,10,19,2)

IF m_breakdown_of_lipids_fatty_acids_and_isoprenoids_n8-SWI6 THEN 000 OR 100 OR 110 OR 111
111: 0.005391 (1644,10,19,2)

IF SFF'-FKH1 AND SWI5-SWI6 THEN 000 OR 100 OR 110 OR 111
111: 0.005391 (1644,10,19,2)

IF m_amino-acid_transporters_n11-SWI4 AND SCB-SWI4 THEN 000 OR 010 OR 110 OR 111
111: 0.005391 (1644,10,19,2)

IF SFF-MIG1 THEN 000 OR 001 OR 010 OR 100 OR 110
100: 0.0056 (1644,61,127,11)

IF LYS14-SWI6 AND mPROTEOL18(m_proteolysis_n18)-MBP1 THEN 000 OR 010 OR 110
110: 0.005709 (1644,6,115,3)

IF LYS14-SWI6 AND mPROTEOL18(m_proteolysis_n18)-MBP1 AND mPROTEOL18(m_proteolysis_n18)-SWI6 THEN 000 OR 010 OR 110
110: 0.005709 (1644,6,115,3)

IF m_nutritional_response_pathway_n7-SWI6 AND MCB-SWI6 THEN 010 OR 101 OR 110 OR 111
110: 0.005709 (1644,6,115,3)

IF MCM1'-MCM1 AND ECB-STE12 THEN 000 OR 010 OR 100 OR 110
110: 0.005709 (1644,6,115,3)

IF ECB-NDD1 AND SFF-FKH1 THEN 000 OR 010 OR 110 OR 111
110: 0.005709 (1644,6,115,3)

IF ECB-FKH2 AND ECB-YOX1 THEN 000 OR 010 OR 110 OR 111
110: 0.005709 (1644,6,115,3)

IF MCM1'-NDD1 AND MCM1'-SWI4 AND SFF-FKH2 THEN 000 OR 100 OR 110
110: 0.005709 (1644,6,115,3)

IF MCB-MIG2 THEN 000 OR 110
110: 0.005709 (1644,6,115,3)

IF m_amino-acid_degradation_n32-UGA3 THEN 000 OR 010 OR 110
110: 0.005709 (1644,6,115,3)

IF MCM1'-MCM1 AND ECB-MCM1 AND m_nucleotide_transport_n9-MCM1 THEN 010 OR 100 OR 110
110: 0.005709 (1644,6,115,3)

IF MCM1-YOX1 AND SFF-FKH2 THEN 000 OR 110 OR 111
110: 0.005709 (1644,6,115,3)

IF MCM1-FKH2 AND MCM1-NDD1 AND MCM1-SWI4 THEN 000 OR 100 OR 110
110: 0.005709 (1644,6,115,3)

IF SFF'-FKH1 AND MCM1-FKH2 AND m_other_morphogenetic_activities_n7-MCM1 THEN 000 OR 010 OR 100 OR 110
110: 0.005709 (1644,6,115,3)

IF m_osmosensing_n6-INO4 AND SFF-FKH1 THEN 000 OR 010 OR 100 OR 110

110: 0.005709 (1644,6,115,3)

IF SFF'-FKH1 AND SCB-SWI4 THEN 000 OR 110 OR 111
110: 0.005709 (1644,6,115,3)

IF MCM1'-NDD1 AND ECB-FKH2 AND SFF-FKH1 THEN 000 OR 010 OR 110 OR 111
110: 0.005709 (1644,6,115,3)

IF MCM1-FKH2 AND m_allantoin_and_allantoate_transporters_n18-FKH2 THEN 000 OR 010 OR 100 OR 110
110: 0.005709 (1644,6,115,3)

IF m_osmosensing_n6-INO4 AND m_osmosensing_n6-ROX1 THEN 000 OR 100 OR 110
110: 0.005709 (1644,6,115,3)

IF MCM1-YOX1 AND ECB-FKH2 THEN 000 OR 010 OR 110 OR 111
110: 0.005709 (1644,6,115,3)

IF MCM1'-NDD1 AND m_allantoin_and_allantoate_transporters_n18-FKH2 THEN 010 OR 100 OR 110
110: 0.005709 (1644,6,115,3)

IF SFF'-FKH2 AND SCB-SWI4 THEN 000 OR 010 OR 110 OR 111
110: 0.005709 (1644,6,115,3)

IF SFF'-FKH1 AND SFF'-MCM1 AND ECB-FKH2 THEN 000 OR 010 OR 110 OR 111
110: 0.005709 (1644,6,115,3)

IF SFF'-FKH1 AND MCM1-MCM1 AND m_other_morphogenetic_activities_n7-MCM1 THEN 000 OR 010 OR 100 OR 110
110: 0.005709 (1644,6,115,3)

IF m_lipid_and_fatty-acid_transport_n11-MTH1 AND m_OCSE15-MTH1 THEN 000 OR 110
110: 0.005709 (1644,6,115,3)

IF SFF'-FKH1 AND MCM1-NDD1 AND m_other_morphogenetic_activities_n7-MCM1 THEN 000 OR 010 OR 100 OR 110
110: 0.005709 (1644,6,115,3)

IF SFF'-FKH1 AND MCM1'-MCM1 AND ECB-FKH2 THEN 000 OR 010 OR 110 OR 111
110: 0.005709 (1644,6,115,3)

IF MCM1-FKH2 AND ECB-YOX1 THEN 000 OR 010 OR 110 OR 111
110: 0.005709 (1644,6,115,3)

IF m_trna_transcription_n10-SWI6 THEN 000 OR 010 OR 110
110: 0.005709 (1644,6,115,3)

IF SFF'-FKH2 AND m_organization_of_golgi_n7-FKH1 THEN 000 OR 110
110: 0.005709 (1644,6,115,3)

IF MCM1-MCM1 AND m_nucleotide_transport_n9-MCM1 THEN 010 OR 100 OR 110
110: 0.005709 (1644,6,115,3)

IF LYS14-SWI6 AND mPROTEOL18(m_proteolysis_n18)-SWI6 THEN 000 OR 010 OR 110
110: 0.005709 (1644,6,115,3)

IF m_osmosensing_n6-SWI4 AND m_anion_transporters_n15-GAT1 THEN 100
100: 0.005924 (1644,2,127,2)

IF m_osmosensing_n6-SWI4 AND m_amino-acid_degradation_n27-GAT1 THEN 100
100: 0.005924 (1644,2,127,2)

IF m_amino-acid_degradation_n27-GAT1 AND m_anion_transporters_n15-INO4 THEN 100
100: 0.005924 (1644,2,127,2)

IF m_amino-acid_degradation_n27-INO4 AND m_anion_transporters_n15-GAT1 THEN 100
100: 0.005924 (1644,2,127,2)

IF SFF'-FKH1 AND m_amino-acid_transport_n18-MET4 THEN 100
100: 0.005924 (1644,2,127,2)

IF m_amino-acid_degradation_n27-GAT1 AND m_anion_transporters_n15-GAT1 THEN 100
100: 0.005924 (1644,2,127,2)

IF m_osmosensing_n6-INO4 AND m_amino-acid_degradation_n27-GAT1 THEN 100
100: 0.005924 (1644,2,127,2)

IF m_osmosensing_n6-SWI4 AND m_amino-acid_degradation_n27-INO4 THEN 100
100: 0.005924 (1644,2,127,2)

IF STRE-SNT2 AND m_other_nucleotide-metabolism_activities_n17-YKL222C THEN 100
100: 0.005924 (1644,2,127,2)

IF STRE-SNT2 AND m_other_nucleotide-metabolism_activities_n17-YGR067C THEN 100
100: 0.005924 (1644,2,127,2)

IF STRE-SNT2 AND m_other_nucleotide-metabolism_activities_n17-YPR022C THEN 100
100: 0.005924 (1644,2,127,2)

IF STRE-SNT2 AND m_other_nucleotide-metabolism_activities_n17-NNF2 THEN 100
100: 0.005924 (1644,2,127,2)

IF m_other_cell_growth_cell_division_and_dna_synthesis_activities_n14.scn-INO4 AND m_pentose-phosphate_pathway_n5-SWI4 THEN 100
100: 0.005924 (1644,2,127,2)

IF MCM1'-SWI4 AND m_other_cell_growth_cell_division_and_dna_synthesis_activities_n14.scn-INO4 THEN 100
100: 0.005924 (1644,2,127,2)

IF m_other_pheromone_response_activities_n5-CBF1 AND m_phosphate_transport_n18-ECM22 THEN 100
100: 0.005924 (1644,2,127,2)

IF m_other_pheromone_response_activities_n5-CBF1 AND m_pheromone_response_generation_n4-ECM22 THEN 100
100: 0.005924 (1644,2,127,2)

IF m_phosphate_transport_n18-PUT3 AND PHO4-CBF1 THEN 100
100: 0.005924 (1644,2,127,2)

IF m_regulation_of_lipid_fatty-acid_and_isoprenoid_biosynthesis_n8.scn-FKH1 AND m_amino-acid_transport_n18-MET4 THEN 100
100: 0.005924 (1644,2,127,2)

IF m_regulation_of_lipid_fatty-acid_and_isoprenoid_biosynthesis_n16.scn-SKN7 AND SFF-FKH1 THEN 100
100: 0.005924 (1644,2,127,2)

IF SFF-FKH1 AND m_amino-acid_transport_n18-MET4 THEN 100
100: 0.005924 (1644,2,127,2)

IF SFF-HIR1 AND SFF-YER184C THEN 100
100: 0.005924 (1644,2,127,2)

IF SFF-HIR1 AND m_anion_transporters_n15-INO4 THEN 100
100: 0.005924 (1644,2,127,2)

IF SFF'-HIR1 AND m_anion_transporters_n15-INO4 THEN 100
100: 0.005924 (1644,2,127,2)

IF SFF'-HIR1 AND SFF-YER184C THEN 100
100: 0.005924 (1644,2,127,2)

IF m_tricarboxylic-acid_pathway_n6-HAP4 AND m_other_morphogenetic_activities_n7-MCM1 THEN 100
100: 0.005924 (1644,2,127,2)

IF SFF'-MCM1 AND m_anion_transporters_n22-HAP4 THEN 100
100: 0.005924 (1644,2,127,2)

IF m_anion_transporters_n22-HAP4 AND m_other_morphogenetic_activities_n7-MCM1 THEN 100
100: 0.005924 (1644,2,127,2)

IF SFF-GZF3 AND ABF1-PPR1 THEN 100
100: 0.005924 (1644,2,127,2)

IF m_PNDE6-SFP1 AND m_biogenesis_of_cytoskeleton_n12-SFP1 THEN 100
100: 0.005924 (1644,2,127,2)

IF m_biogenesis_of_cytoskeleton_n12-SFP1 AND ALPHA1'-SFP1 THEN 100
100: 0.005924 (1644,2,127,2)

IF m_pheromone_response_generation_n7-SMK1 THEN 100
100: 0.005924 (1644,2,127,2)

IF PDR-UME6 AND m_pheromone_response_generation_n4-PUT3 THEN 100
100: 0.005924 (1644,2,127,2)

IF m_amino-acid_degradation_n27-HIR2 AND m_pheromone_response_generation_n4-PUT3 THEN 100
100: 0.005924 (1644,2,127,2)

IF m_pheromone_response_generation_n4-PUT3 AND m_glyoxylate_cycle_n11-UME6 THEN 100
100: 0.005924 (1644,2,127,2)

IF m_g-proteins_n12-INO4 AND m_regulation_of_amino-acid_metabolism_n15-INO4 AND m_cell_death_n16-INO4 THEN 100
100: 0.005924 (1644,2,127,2)

IF m_purine_and_pyrimidine_transporters_n10-SKO1 AND mRRPE-IXR1 THEN 100
100: 0.005924 (1644,2,127,2)

IF m_lipid_and_fatty-acid_transport_n11-SKN7 AND m_meiosis_n3-UME6 THEN 100
100: 0.005924 (1644,2,127,2)

IF m_allantoin_and_allantoate_transporters_n13-MAL13 AND m_deoxyribonucleotide_metabolism_n8-MAL13 THEN 100
100: 0.005924 (1644,2,127,2)

IF m_lipid_and_fatty-acid_transport_n11-SKN7 AND m_LFTE17-UME6 THEN 100
100: 0.005924 (1644,2,127,2)

IF m_lipid_and_fatty-acid_transport_n11-GAT1 AND m_allantoin_and_allantoate_transporters_n13-MAL13 THEN 100
100: 0.005924 (1644,2,127,2)

IF m_metabolism_of_cyclic_and_unusual_nucleotides_n5-YAP1 AND MCM1'-SWI4 THEN 100
100: 0.005924 (1644,2,127,2)

IF MCM1-FKH2 AND m_anion_transporters_n15-INO4 THEN 100
100: 0.005924 (1644,2,127,2)

IF SFF'-FKH2 AND m_nitrogen_and_sulphur_metabolism_n17-XBP1 THEN 100
100: 0.005924 (1644,2,127,2)

IF SFF'-MCM1 AND m_tricarboxylic-acid_pathway_n6-HAP4 THEN 100
100: 0.005924 (1644,2,127,2)

IF m_other_morphogenetic_activities_n7-RTG1 AND ABF1-SIG1 THEN 100
100: 0.005924 (1644,2,127,2)

IF m_allantoin_and_allantoate_transporters_n11-RIM101 AND SFF-RTG3 THEN 100
100: 0.005924 (1644,2,127,2)

IF CCA-MET4 AND m_organization_of_chromosome_structure_n12-HIR2 THEN 100
100: 0.005924 (1644,2,127,2)

IF m_amino-acid_degradation_n27-HIR2 AND m_phosphate_transport_n18-PUT3 THEN 100
100: 0.005924 (1644,2,127,2)

IF SFF'-FKH2 AND m_g-proteins_n13-ACE2 THEN 100
100: 0.005924 (1644,2,127,2)

IF m_allantoin_and_allantoate_transporters_n18-FKH2 AND m_ion_transporters_n7-XBP1 THEN 100
100: 0.005924 (1644,2,127,2)

IF SFF'-FKH2 AND m_phosphate_transport_n5-GAT1 THEN 100
100: 0.005924 (1644,2,127,2)

IF SFF'-FKH2 AND m_pentose-phosphate_pathway_n5-SWI4 THEN 100
100: 0.005924 (1644,2,127,2)

IF SFF-FKH1 AND m_pentose-phosphate_pathway_n5-SWI4 THEN 100

100: 0.005924 (1644,2,127,2)

IF SFF'-FKH1 AND m_g-proteins_n13-ACE2 THEN 100
100: 0.005924 (1644,2,127,2)

IF m_allantoin_and_allantoate_transporters_n18-FKH2 AND m_ion_transporters_n7-TOS8 THEN 100
100: 0.005924 (1644,2,127,2)

IF m_phosphate_transport_n5-INO4 AND PHO-INO4 THEN 100
100: 0.005924 (1644,2,127,2)

IF SFF-FKH2 AND m_pentose-phosphate_pathway_n5-SWI4 THEN 100
100: 0.005924 (1644,2,127,2)

IF m_utilization_of_vitamins_cofactors_and_prosthetic_groups_n7-DAL81 AND m_g-proteins_n11-UME6 THEN 100
100: 0.005924 (1644,2,127,2)

IF MCM1'-NDD1 AND m_other_cell_growth_cell_division_and_dna_synthesis_activities_n14.scn-INO4 THEN 100
100: 0.005924 (1644,2,127,2)

IF SFF-FKH2 AND m_g-proteins_n13-ACE2 THEN 100
100: 0.005924 (1644,2,127,2)

IF m_other_cell_growth_cell_division_and_dna_synthesis_activities_n14.scn-INO4 AND SWI5-SWI6 THEN 100
100: 0.005924 (1644,2,127,2)

IF SFF'-RTG3 AND m_allantoin_and_allantoate_transporters_n11-RIM101 THEN 100
100: 0.005924 (1644,2,127,2)

IF m_utilization_of_vitamins_cofactors_and_prosthetic_groups_n7-DAL81 AND SWI5-SWI6 THEN 100
100: 0.005924 (1644,2,127,2)

IF MCM1'-MCM1 AND m_g-proteins_n11-INO4 THEN 100
100: 0.005924 (1644,2,127,2)

IF MCM1'-NDD1 AND m_g-proteins_n11-INO4 THEN 100
100: 0.005924 (1644,2,127,2)

IF m_anion_transporters_n22-RAP1 AND MIG1-YAP6 THEN 100
100: 0.005924 (1644,2,127,2)

IF m_amino-acid_degradation_n27-GAT1 AND m_amino-acid_degradation_n27-INO4 THEN 100
100: 0.005924 (1644,2,127,2)

IF m_osmosensing_n6-INO4 AND m_amino-acid_degradation_n27-INO4 THEN 100
100: 0.005924 (1644,2,127,2)

IF SFF'-MCM1 AND m_g-proteins_n11-INO4 THEN 100
100: 0.005924 (1644,2,127,2)

IF MCM1-MCM1 AND m_g-proteins_n11-INO4 THEN 100
100: 0.005924 (1644,2,127,2)

IF SFF-FKH1 AND m_g-proteins_n13-ACE2 THEN 100
100: 0.005924 (1644,2,127,2)

IF m_other_transcription_activities_n5-SWI6 AND STRE-SWI6 THEN 100
100: 0.005924 (1644,2,127,2)

IF m_g-proteins_n11-INO4 AND m_amino-acid_degradation_n27-GAT1 THEN 100
100: 0.005924 (1644,2,127,2)

IF m_osmosensing_n6-SWI4 AND m_g-proteins_n11-INO4 THEN 100
100: 0.005924 (1644,2,127,2)

IF m_c-compound_carbohydrate_transport_n11-RIM101 AND SWI5-RIM101 THEN 100
100: 0.005924 (1644,2,127,2)

IF m_c-compound_and_carbohydrate_metabolism_n8-FKH2 AND m_metabolism_of_cyclic_and_unusual_nucleotides_n5-YAP1 THEN 100
100: 0.005924 (1644,2,127,2)

IF m_other_morphogenetic_activities_n7-HOG1 THEN 000 OR 001 OR 010 OR 100 OR 110
110: 0.006061 (1644,25,115,6)

IF mPROTEOL18(m_proteolysis_n18)-YAP6 THEN 000 OR 010 OR 100 OR 110 OR 111
010: 0.006217 (1644,15,140,5)

IF m_regulation_of_nitrogen_and_sulphur_utilization_n13-MTH1 AND m_other_nutritional-response_activities_n10-MTH1 AND m_cell_death_n22-MTH1 THEN 000 OR 001 OR 100
001: 0.006317 (1644,4,55,2)

IF STRE-RAP1 AND m_pentose-phosphate_pathway_n23-RAP1 THEN 000 OR 001
001: 0.006317 (1644,4,55,2)

IF m_other_mrna-transcription_activities_n20-RAP1 AND STRE-RAP1 THEN 000 OR 001
001: 0.006317 (1644,4,55,2)

IF m_pentose-phosphate_pathway_n23-RAP1 AND m_amino-acid_transport_n20-RAP1 THEN 000 OR 001
001: 0.006317 (1644,4,55,2)

IF m_anion_transporters_n17-INO4 AND m_g-proteins_n11-INO4 THEN 000 OR 001 OR 100
001: 0.006317 (1644,4,55,2)

IF m_g-proteins_n12-INO4 AND m_other_mrna-transcription_activities_n20-RAP1 THEN 000 OR 001
001: 0.006317 (1644,4,55,2)

IF SWI5-SKN7 AND SCB-UME6 THEN 000 OR 001 OR 010
001: 0.006317 (1644,4,55,2)

IF m_nutritional_response_pathway_n8-INO4 AND m_organization_of_chromosome_structure_n17-INO4 THEN 000 OR 001
001: 0.006317 (1644,4,55,2)

IF m_anion_transporters_n22-SKN7 AND m_anion_transporters_n20-RAP1 THEN 000 OR 001 OR 110
001: 0.006317 (1644,4,55,2)

IF m_other_mrna-transcription_activities_n20-RAP1 AND m_RPE21-RGM1 THEN 000 OR 001
001: 0.006317 (1644,4,55,2)

IF m_g-proteins_n12-INO4 AND m_nutritional_response_pathway_n8-INO4 AND m_other_mrna-transcription_activities_n20-INO4 THEN 000 OR 001 OR 010
001: 0.006317 (1644,4,55,2)

IF m_other_energy_generation_activities_n4-UME6 AND SCB-UME6 THEN 000 OR 001 OR 010
001: 0.006317 (1644,4,55,2)

IF m_amino-acid_degradation_n7-GAT1 AND m_homeostasis_of_other_ions_n30-GAT1 THEN 000 OR 001 OR 111
001: 0.006317 (1644,4,55,2)

IF MCM1'-PDC2 AND RAP1-ZAP1 THEN 010
010: 0.007204 (1644,2,140,2)

IF RAP1-DAT1 AND m_nitrogen_and_sulphur_transport_n9-FHL1 THEN 010
010: 0.007204 (1644,2,140,2)

IF m_other_morphogenetic_activities_n7-ARR1 AND RAP1-RAP1 THEN 010
010: 0.007204 (1644,2,140,2)

IF RAP1-HAP4 AND ALPHA1-DAT1 THEN 010
010: 0.007204 (1644,2,140,2)

IF SFF-MIG1 AND m_glyoxylate_cycle_n11-RGM1 THEN 010
010: 0.007204 (1644,2,140,2)

IF m_utilization_of_vitamins_cofactors_and_prosthetic_groups_n7-STP2 AND RAP1-ZAP1 THEN 010
010: 0.007204 (1644,2,140,2)

IF m_lipid_and_fatty-acid_binding_n13-ARG81 AND m_lipid_and_fatty-acid_binding_n13-PDR1 THEN 010
010: 0.007204 (1644,2,140,2)

IF m_other_morphogenetic_activities_n7-ARR1 AND m_lipid_and_fatty-acid_binding_n13-UME1 THEN 010

010: 0.007204 (1644,2,140,2)

IF SFF-HIR1 AND m_lipid_and_fatty-acid_binding_n15-FHL1 THEN 010
010: 0.007204 (1644,2,140,2)

IF m_utilization_of_vitamins_cofactors_and_prosthetic_groups_n7-STP2 AND m_other_morphogenetic_activities_n7-ARR1 THEN 010
010: 0.007204 (1644,2,140,2)

IF m_anion_transporters_n22-RAP1 AND m_utilization_of_vitamins_cofactors_and_prosthetic_groups_n7-STP2 THEN 010
010: 0.007204 (1644,2,140,2)

IF RAP1-HIR2 AND m_lipid_and_fatty-acid_binding_n13-UME1 THEN 010
010: 0.007204 (1644,2,140,2)

IF STE12-IXR1 AND RAP1-YAP5 THEN 010
010: 0.007204 (1644,2,140,2)

IF m_utilization_of_vitamins_cofactors_and_prosthetic_groups_n7-STP2 AND RAP1-YAP5 THEN 010
010: 0.007204 (1644,2,140,2)

IF SFF'-HIR3 AND m_utilization_of_vitamins_cofactors_and_prosthetic_groups_n7-STP2 THEN 010
010: 0.007204 (1644,2,140,2)

IF m_regulation_of_lipid_fatty-acid_and_isoprenoid_biosynthesis_n22.scn-DAL80 THEN 010
010: 0.007204 (1644,2,140,2)

IF m_amino-acid_transport_n13-MTH1 AND m_regulation_of_lipid_fatty-acid_and_isoprenoid_biosynthesis_n8.scn-MTH1 THEN 010
010: 0.007204 (1644,2,140,2)

IF m_utilization_of_vitamins_cofactors_and_prosthetic_groups_n7-STP2 AND RAP1-HIR2 THEN 010
010: 0.007204 (1644,2,140,2)

IF SFF-HIR1 AND ALPHA1-DAT1 THEN 010
010: 0.007204 (1644,2,140,2)

IF m_anion_transporters_n22-HAP4 AND RAP1-RAP1 THEN 010
010: 0.007204 (1644,2,140,2)

IF m_nitrogen_and_sulphur_transport_n9-FHL1 AND m_lipid_and_fatty-acid_binding_n13-ARG81 THEN 010
010: 0.007204 (1644,2,140,2)

IF RAP1-GAT3 AND m_lipid_and_fatty-acid_binding_n13-UME1 THEN 010
010: 0.007204 (1644,2,140,2)

IF RAP1-YAP5 AND m_lipid_and_fatty-acid_binding_n13-PDR1 THEN 010
010: 0.007204 (1644,2,140,2)

IF m_utilization_of_vitamins_cofactors_and_prosthetic_groups_n7-STP2 AND RAP1-GAT3 THEN 010
010: 0.007204 (1644,2,140,2)

IF m_lipid_and_fatty-acid_binding_n15-FHL1 AND RAP1-ARG81 THEN 010
010: 0.007204 (1644,2,140,2)

IF SFF'-HIR1 AND m_lipid_and_fatty-acid_binding_n13-ARG81 THEN 010
010: 0.007204 (1644,2,140,2)

IF STE12-IXR1 AND m_lipid_and_fatty-acid_binding_n15-STP2 THEN 010
010: 0.007204 (1644,2,140,2)

IF MCM1'-PDC2 AND m_lipid_and_fatty-acid_binding_n13-UME1 THEN 010
010: 0.007204 (1644,2,140,2)

IF m_anion_transporters_n22-RAP1 AND m_lipid_and_fatty-acid_binding_n15-STP2 THEN 010
010: 0.007204 (1644,2,140,2)

IF SFF'-HIR1 AND m_nitrogen_and_sulphur_transport_n9-FHL1 THEN 010
010: 0.007204 (1644,2,140,2)

IF m_lipid_and_fatty-acid_binding_n15-FHL1 AND RAP1-HIR2 THEN 010
010: 0.007204 (1644,2,140,2)

IF MCM1'-PDC2 AND m_utilization_of_vitamins_cofactors_and_prosthetic_groups_n7-STP2 THEN 010
010: 0.007204 (1644,2,140,2)

IF m_utilization_of_vitamins_cofactors_and_prosthetic_groups_n7-STP2 AND ALPHA1-GAT3 THEN 010
010: 0.007204 (1644,2,140,2)

IF RAP1-RAP1 AND m_lipid_and_fatty-acid_binding_n13-PDR1 THEN 010
010: 0.007204 (1644,2,140,2)

IF m_utilization_of_vitamins_cofactors_and_prosthetic_groups_n7-DAL81 AND RAP1-RAP1 THEN 010
010: 0.007204 (1644,2,140,2)

IF m_utilization_of_vitamins_cofactors_and_prosthetic_groups_n7-DAL81 AND m_lipid_and_fatty-acid_binding_n15-FHL1 THEN 010
010: 0.007204 (1644,2,140,2)

IF RAP1-ARG81 AND m_lipid_and_fatty-acid_binding_n13-UME1 THEN 010
010: 0.007204 (1644,2,140,2)

IF m_utilization_of_vitamins_cofactors_and_prosthetic_groups_n7-STP2 AND m_lipid_and_fatty-acid_binding_n15-STP2 THEN 010
010: 0.007204 (1644,2,140,2)

IF m_anion_transporters_n22-RAP1 AND m_utilization_of_vitamins_cofactors_and_prosthetic_groups_n7-DAL81 THEN 010
010: 0.007204 (1644,2,140,2)

IF RAP1-PDR1 AND m_lipid_and_fatty-acid_binding_n13-UME1 THEN 010
010: 0.007204 (1644,2,140,2)

IF MCM1'-PDC2 AND m_utilization_of_vitamins_cofactors_and_prosthetic_groups_n7-DAL81 THEN 010
010: 0.007204 (1644,2,140,2)

IF m_lipid_and_fatty-acid_binding_n15-FHL1 AND m_lipid_and_fatty-acid_binding_n13-ARG81 THEN 010
010: 0.007204 (1644,2,140,2)

IF m_lipid_and_fatty-acid_binding_n15-FHL1 AND RAP1-ARO80 THEN 010
010: 0.007204 (1644,2,140,2)

IF m_utilization_of_vitamins_cofactors_and_prosthetic_groups_n7-DAL81 AND m_lipid_and_fatty-acid_binding_n13-ARG81 THEN 010
010: 0.007204 (1644,2,140,2)

IF m_utilization_of_vitamins_cofactors_and_prosthetic_groups_n7-STP2 AND m_lipid_and_fatty-acid_binding_n13-UME1 THEN 010
010: 0.007204 (1644,2,140,2)

IF RAP1-ARG81 AND m_lipid_and_fatty-acid_binding_n13-PDR1 THEN 010
010: 0.007204 (1644,2,140,2)

IF MCM1'-PDC2 AND m_lipid_and_fatty-acid_binding_n13-PDR1 THEN 010
010: 0.007204 (1644,2,140,2)

IF MCM1'-PDC2 AND SFF-HIR1 THEN 010
010: 0.007204 (1644,2,140,2)

IF m_anion_transporters_n22-RAP1 AND RAP1-ARG81 THEN 010
010: 0.007204 (1644,2,140,2)

IF m_utilization_of_vitamins_cofactors_and_prosthetic_groups_n7-DAL81 AND RAP1-DAT1 THEN 010
010: 0.007204 (1644,2,140,2)

IF m_utilization_of_vitamins_cofactors_and_prosthetic_groups_n7-DAL81 AND RAP1-ARG81 THEN 010
010: 0.007204 (1644,2,140,2)

IF m_lipid_and_fatty-acid_binding_n15-STP2 AND RAP1-DAT1 THEN 010
010: 0.007204 (1644,2,140,2)

IF m_lipid_and_fatty-acid_binding_n15-STP2 AND m_lipid_and_fatty-acid_binding_n13-PDR1 THEN 010
010: 0.007204 (1644,2,140,2)

IF m_utilization_of_vitamins_cofactors_and_prosthetic_groups_n7-DAL81 AND ALPHA1-GAT3 THEN 010
010: 0.007204 (1644,2,140,2)

IF RAP1-GAT3 AND m_lipid_and_fatty-acid_binding_n13-PDR1 THEN 010
010: 0.007204 (1644,2,140,2)

IF m_utilization_of_vitamins_cofactors_and_prosthetic_groups_n7-STP2 AND RAP1-ARO80 THEN 010
010: 0.007204 (1644,2,140,2)

IF m_lipid_and_fatty-acid_binding_n15-FHL1 AND m_lipid_and_fatty-acid_binding_n13-PDR1 THEN 010
010: 0.007204 (1644,2,140,2)

IF SFF'-HIR3 AND m_lipid_and_fatty-acid_binding_n15-STP2 THEN 010
010: 0.007204 (1644,2,140,2)

IF m_anion_transporters_n22-RAP1 AND RAP1-PDR1 THEN 010
010: 0.007204 (1644,2,140,2)

IF MCM1'-PDC2 AND RAP1-RAP1 THEN 010
010: 0.007204 (1644,2,140,2)

IF m_other_morphogenetic_activities_n7-ARR1 AND RAP1-GAT3 THEN 010
010: 0.007204 (1644,2,140,2)

IF m_anion_transporters_n22-HAP4 AND RAP1-PDR1 THEN 010
010: 0.007204 (1644,2,140,2)

IF SFF'-HIR3 AND m_lipid_and_fatty-acid_binding_n13-ARG81 THEN 010
010: 0.007204 (1644,2,140,2)

IF m_anion_transporters_n22-HAP4 AND m_lipid_and_fatty-acid_binding_n15-FHL1 THEN 010
010: 0.007204 (1644,2,140,2)

IF m_lipid_and_fatty-acid_binding_n15-FHL1 AND RAP1-HAP4 THEN 010
010: 0.007204 (1644,2,140,2)

IF m_other_morphogenetic_activities_n7-ARR1 AND RAP1-YAP5 THEN 010
010: 0.007204 (1644,2,140,2)

IF STE12-IXR1 AND ALPHA1-DAT1 THEN 010
010: 0.007204 (1644,2,140,2)

IF SFF'-HIR1 AND m_utilization_of_vitamins_cofactors_and_prosthetic_groups_n7-DAL81 THEN 010
010: 0.007204 (1644,2,140,2)

IF RAP1-YAP5 AND m_lipid_and_fatty-acid_binding_n13-ARG81 THEN 010
010: 0.007204 (1644,2,140,2)

IF m_lipid_and_fatty-acid_binding_n15-STP2 AND RAP1-HAP4 THEN 010
010: 0.007204 (1644,2,140,2)

IF ALPHA1-DAT1 AND m_lipid_and_fatty-acid_binding_n13-PDR1 THEN 010
010: 0.007204 (1644,2,140,2)

IF m_other_proteolytic_degradation_n2-MTH1 AND m_rRSE10-MTH1 AND m_regulation_of_lipid_fatty-acid_and_isoprenoid_biosynthesis_n8.scn-MTH1 THEN 010
010: 0.007204 (1644,2,140,2)

IF mPROTEOL18(m_proteolysis_n18)-UME6 AND m_amino-acid_transporters_n11-CUP9 THEN 010
010: 0.007204 (1644,2,140,2)

IF m_other_morphogenetic_activities_n7-ARR1 AND RAP1-HIR2 THEN 010
010: 0.007204 (1644,2,140,2)

IF m_MERE11-SUT1 AND m_amino-acid_transporters_n11-UME6 THEN 010
010: 0.007204 (1644,2,140,2)

IF m_anion_transporters_n22-RAP1 AND RAP1-ZAP1 THEN 010
010: 0.007204 (1644,2,140,2)

IF m_utilization_of_vitamins_cofactors_and_prosthetic_groups_n7-DAL81 AND STE12-IXR1 THEN 010
010: 0.007204 (1644,2,140,2)

IF m_utilization_of_vitamins_cofactors_and_prosthetic_groups_n7-STP2 AND RAP1-HAP4 THEN 010
010: 0.007204 (1644,2,140,2)

IF SFF'-HIR3 AND m_anion_transporters_n22-HAP4 THEN 010
010: 0.007204 (1644,2,140,2)

IF m_anion_transporters_n22-HAP4 AND m_lipid_and_fatty-acid_binding_n13-PDR1 THEN 010
010: 0.007204 (1644,2,140,2)

IF SFF'-HIR3 AND m_utilization_of_vitamins_cofactors_and_prosthetic_groups_n7-DAL81 THEN 010
010: 0.007204 (1644,2,140,2)

IF MCM1'-PDC2 AND STE12-IXR1 THEN 010
010: 0.007204 (1644,2,140,2)

IF ALPHA1-DAT1 AND m_lipid_and_fatty-acid_binding_n13-UME1 THEN 010
010: 0.007204 (1644,2,140,2)

IF m_anion_transporters_n22-HAP4 AND ALPHA1-DAT1 THEN 010
010: 0.007204 (1644,2,140,2)

IF RAP1-DAT1 AND RAP1-HIR2 THEN 010
010: 0.007204 (1644,2,140,2)

IF RAP1-DAT1 AND m_lipid_and_fatty-acid_binding_n13-UME1 THEN 010
010: 0.007204 (1644,2,140,2)

IF m_utilization_of_vitamins_cofactors_and_prosthetic_groups_n7-STP2 AND STE12-IXR1 THEN 010
010: 0.007204 (1644,2,140,2)

IF m_other_morphogenetic_activities_n7-ARR1 AND RAP1-FHL1 THEN 010
010: 0.007204 (1644,2,140,2)

IF mPROTEOL18(m_proteolysis_n18)-MBP1 AND m_amino-acid_transporters_n11-CUP9 AND m_amino-acid_transporters_n11-UME6 THEN 010
010: 0.007204 (1644,2,140,2)

IF m_other_morphogenetic_activities_n7-ARR1 AND RAP1-ARG81 THEN 010
010: 0.007204 (1644,2,140,2)

IF m_other_morphogenetic_activities_n7-ARR1 AND m_nitrogen_and_sulphur_transport_n9-FHL1 THEN 010
010: 0.007204 (1644,2,140,2)

IF RAP1-ARO80 AND m_lipid_and_fatty-acid_binding_n13-UME1 THEN 010
010: 0.007204 (1644,2,140,2)

IF RAP1-ARO80 AND ALPHA1-DAT1 THEN 010
010: 0.007204 (1644,2,140,2)

IF RAP1-ARO80 AND m_lipid_and_fatty-acid_binding_n13-PDR1 THEN 010
010: 0.007204 (1644,2,140,2)

IF RAP1-HAP4 AND m_lipid_and_fatty-acid_binding_n13-PDR1 THEN 010
010: 0.007204 (1644,2,140,2)

IF m_anion_transporters_n22-RAP1 AND RAP1-HAP4 THEN 010
010: 0.007204 (1644,2,140,2)

IF MCM1'-PDC2 AND RAP1-ARO80 THEN 010
010: 0.007204 (1644,2,140,2)

IF m_lipid_and_fatty-acid_binding_n15-STP2 AND RAP1-ARO80 THEN 010
010: 0.007204 (1644,2,140,2)

IF SFF'-HIR1 AND RAP1-DAT1 THEN 010
010: 0.007204 (1644,2,140,2)

IF RAP1-ARO80 AND RAP1-DAT1 THEN 010
010: 0.007204 (1644,2,140,2)

IF m_utilization_of_vitamins_cofactors_and_prosthetic_groups_n7-STP2 AND m_lipid_and_fatty-acid_binding_n13-PDR1 THEN 010
010: 0.007204 (1644,2,140,2)

IF m_utilization_of_vitamins_cofactors_and_prosthetic_groups_n7-STP2 AND RAP1-PDR1 THEN 010
010: 0.007204 (1644,2,140,2)

IF MCM1'-PDC2 AND RAP1-YAP5 THEN 010
010: 0.007204 (1644,2,140,2)

IF MCM1'-PDC2 AND m_lipid_and_fatty-acid_binding_n15-STP2 THEN 010
010: 0.007204 (1644,2,140,2)

IF RAP1-YAP5 AND m_lipid_and_fatty-acid_binding_n13-UME1 THEN 010
010: 0.007204 (1644,2,140,2)

IF SFF'-HIR3 AND m_lipid_and_fatty-acid_binding_n13-UME1 THEN 010
010: 0.007204 (1644,2,140,2)

IF MCM1'-PDC2 AND m_lipid_and_fatty-acid_binding_n13-ARG81 THEN 010
010: 0.007204 (1644,2,140,2)

IF MCM1'-PDC2 AND m_other_morphogenetic_activities_n7-STP1 THEN 010
010: 0.007204 (1644,2,140,2)

IF SFF-MIG1 AND m_pentose-phosphate_pathway_n23-RAP1 THEN 010
010: 0.007204 (1644,2,140,2)

IF m_anion_transporters_n22-RAP1 AND RAP1-DAT1 THEN 010
010: 0.007204 (1644,2,140,2)

IF MCM1'-PDC2 AND RAP1-ARG81 THEN 010
010: 0.007204 (1644,2,140,2)

IF RAP1-ARG81 AND RAP1-GAT3 THEN 010
010: 0.007204 (1644,2,140,2)

IF m_lipid_and_fatty-acid_binding_n15-STP2 AND RAP1-HIR2 THEN 010
010: 0.007204 (1644,2,140,2)

IF SFF'-HIR3 AND m_lipid_and_fatty-acid_binding_n15-FHL1 THEN 010
010: 0.007204 (1644,2,140,2)

IF m_utilization_of_vitamins_cofactors_and_prosthetic_groups_n7-STP2 AND m_lipid_and_fatty-acid_binding_n15-FHL1 THEN 010
010: 0.007204 (1644,2,140,2)

IF m_anion_transporters_n22-RAP1 AND m_other_morphogenetic_activities_n7-ARR1 THEN 010
010: 0.007204 (1644,2,140,2)

IF RAP1-ARG81 AND m_nitrogen_and_sulphur_transport_n9-FHL1 THEN 010
010: 0.007204 (1644,2,140,2)

IF RAP1-GAT3 AND m_lipid_and_fatty-acid_binding_n13-ARG81 THEN 010
010: 0.007204 (1644,2,140,2)

IF m_other_morphogenetic_activities_n7-ARR1 AND ALPHA1-DAT1 THEN 010
010: 0.007204 (1644,2,140,2)

IF m_lipid_and_fatty-acid_binding_n15-FHL1 AND m_lipid_and_fatty-acid_binding_n15-STP2 THEN 010
010: 0.007204 (1644,2,140,2)

IF m_utilization_of_vitamins_cofactors_and_prosthetic_groups_n7-DAL81 AND m_other_morphogenetic_activities_n7-ARR1 THEN 010
010: 0.007204 (1644,2,140,2)

IF m_other_morphogenetic_activities_n7-ARR1 AND RAP1-HAP4 THEN 010
010: 0.007204 (1644,2,140,2)

IF m_utilization_of_vitamins_cofactors_and_prosthetic_groups_n7-STP2 AND ALPHA1-DAT1 THEN 010
010: 0.007204 (1644,2,140,2)

IF m_lipid_and_fatty-acid_binding_n15-STP2 AND RAP1-ZAP1 THEN 010

010: 0.007204 (1644,2,140,2)

IF m_lipid_and_fatty-acid_binding_n15-STP2 AND RAP1-PDR1 THEN 010
010: 0.007204 (1644,2,140,2)

IF RAP1-RAP1 AND m_lipid_and_fatty-acid_binding_n13-ARG81 THEN 010
010: 0.007204 (1644,2,140,2)

IF m_lipid_and_fatty-acid_binding_n15-STP2 AND m_lipid_and_fatty-acid_binding_n13-UME1 THEN 010
010: 0.007204 (1644,2,140,2)

IF m_other_morphogenetic_activities_n7-ARR1 AND m_lipid_and_fatty-acid_binding_n15-STP2 THEN 010
010: 0.007204 (1644,2,140,2)

IF m_lipid_and_fatty-acid_binding_n15-STP2 AND RAP1-ARG81 THEN 010
010: 0.007204 (1644,2,140,2)

IF m_lipid_and_fatty-acid_binding_n15-STP2 AND RAP1-YAP5 THEN 010
010: 0.007204 (1644,2,140,2)

IF SFF-ARG80 AND m_other_morphogenetic_activities_n7-GCR2 AND ALPHA1'-KSS1 THEN 010
010: 0.007204 (1644,2,140,2)

IF SFF-ARG80 AND ALPHA1'-KSS1 AND ALPHA1'-STB4 THEN 010
010: 0.007204 (1644,2,140,2)

IF m_lipid_and_fatty-acid_binding_n15-FHL1 AND ALPHA1-DAT1 THEN 010
010: 0.007204 (1644,2,140,2)

IF m_lipid_and_fatty-acid_binding_n15-STP2 AND m_lipid_and_fatty-acid_binding_n13-ARG81 THEN 010
010: 0.007204 (1644,2,140,2)

IF ALPHA1-DAT1 AND m_lipid_and_fatty-acid_binding_n13-ARG81 THEN 010
010: 0.007204 (1644,2,140,2)

IF MCM1'-SWI4 AND SWI5-SWI6 AND SCB-UME6 THEN 010
010: 0.007204 (1644,2,140,2)

IF MCM1'-SWI4 AND m_amino-acid_transporters_n11-UME6 AND SCB-UME6 THEN 010
010: 0.007204 (1644,2,140,2)

IF m_lipid_and_fatty-acid_binding_n15-STP2 AND ALPHA1-DAT1 THEN 010
010: 0.007204 (1644,2,140,2)

IF STE12-IXR1 AND RAP1-DAT1 THEN 010
010: 0.007204 (1644,2,140,2)

IF m_other_morphogenetic_activities_n7-ARR1 AND RAP1-PDR1 THEN 010
010: 0.007204 (1644,2,140,2)

IF m_lipid_and_fatty-acid_binding_n15-STP2 AND RAP1-GAT3 THEN 010
010: 0.007204 (1644,2,140,2)

IF RAP1-DAT1 AND m_lipid_and_fatty-acid_binding_n13-PDR1 THEN 010
010: 0.007204 (1644,2,140,2)

IF MCM1'-PDC2 AND m_lipid_and_fatty-acid_binding_n15-FHL1 THEN 010
010: 0.007204 (1644,2,140,2)

IF RAP1-ARG81 AND ALPHA1-GAT3 THEN 010
010: 0.007204 (1644,2,140,2)

IF SFF-HIR1 AND m_lipid_and_fatty-acid_binding_n13-UME1 THEN 010
010: 0.007204 (1644,2,140,2)

IF RAP1-DAT1 AND RAP1-HAP4 THEN 010
010: 0.007204 (1644,2,140,2)

IF m_anion_transporters_n22-HAP4 AND RAP1-DAT1 THEN 010
010: 0.007204 (1644,2,140,2)

IF SFF-ARG80 AND RAP1-PDR1 THEN 010
010: 0.007204 (1644,2,140,2)

IF SFF-ARG80 AND RAP1-HMS1 THEN 010
010: 0.007204 (1644,2,140,2)

IF m_peroxisomal_transport_n22-RAP1 AND RAP1-PDR1 THEN 010
010: 0.007204 (1644,2,140,2)

IF SFF-ARG80 AND RAP1-FHL1 THEN 010
010: 0.007204 (1644,2,140,2)

IF m_amino-acid_transport_n18-SKN7 AND SCB-AZF1 THEN 010
010: 0.007204 (1644,2,140,2)

IF SFF'-FKH1 AND m_other_transcription_activities_n5-SWI6 THEN 010
010: 0.007204 (1644,2,140,2)

IF SFF-FKH1 AND m_other_transcription_activities_n5-SWI6 THEN 010
010: 0.007204 (1644,2,140,2)

IF m_peroxisomal_transport_n22-RAP1 AND RAP1-ROX1 THEN 010
010: 0.007204 (1644,2,140,2)

IF m_amino-acid_transporters_n11-UME6 AND SCB-SWI4 THEN 010
010: 0.007204 (1644,2,140,2)

IF m_amino-acid_transporters_n11-SWI4 AND SCB-UME6 THEN 010
010: 0.007204 (1644,2,140,2)

IF SWI5-SWI6 AND m_amino-acid_transporters_n11-UME6 THEN 010
010: 0.007204 (1644,2,140,2)

IF mPROTEOL18(m_proteolysis_n18)-SWI6 AND m_other_mrna-transcription_activities_n20-INO4 THEN 010
010: 0.007204 (1644,2,140,2)

IF SCB-SWI4 AND m_glyoxylate_cycle_n11-SKO1 THEN 010
010: 0.007204 (1644,2,140,2)

IF mPROTEOL18(m_proteolysis_n18)-YAP6 AND m_other_mrna-transcription_activities_n20-INO4 THEN 010
010: 0.007204 (1644,2,140,2)

IF m_tricarboxylic-acid_pathway_n9-SKN7 AND m_glyoxylate_cycle_n11-SKO1 THEN 010
010: 0.007204 (1644,2,140,2)

IF m_tricarboxylic-acid_pathway_n9-SKN7 AND SCB-SWI4 THEN 010
010: 0.007204 (1644,2,140,2)

IF m_anion_transporters_n22-HAP4 AND m_utilization_of_vitamins_cofactors_and_prosthetic_groups_n7-STP2 THEN 010
010: 0.007204 (1644,2,140,2)

IF m_utilization_of_vitamins_cofactors_and_prosthetic_groups_n7-STP2 AND RAP1-FHL1 THEN 010
010: 0.007204 (1644,2,140,2)

IF RAP1-ZAP1 AND m_lipid_and_fatty-acid_binding_n13-ARG81 THEN 010
010: 0.007204 (1644,2,140,2)

IF STE12-IXR1 AND ALPHA1-GAT3 THEN 010
010: 0.007204 (1644,2,140,2)

IF m_utilization_of_vitamins_cofactors_and_prosthetic_groups_n7-STP2 AND RAP1-ARG81 THEN 010
010: 0.007204 (1644,2,140,2)

IF ALPHA1-GAT3 AND m_lipid_and_fatty-acid_binding_n13-ARG81 THEN 010
010: 0.007204 (1644,2,140,2)

IF SFF-HIR1 AND m_lipid_and_fatty-acid_binding_n13-PDR1 THEN 010
010: 0.007204 (1644,2,140,2)

IF m_utilization_of_vitamins_cofactors_and_prosthetic_groups_n7-DAL81 AND SFF-HIR1 THEN 010
010: 0.007204 (1644,2,140,2)

IF STE12-IXR1 AND RAP1-ARG81 THEN 010
010: 0.007204 (1644,2,140,2)

IF SFF-HIR1 AND RAP1-DAT1 THEN 010
010: 0.007204 (1644,2,140,2)

IF RAP1-PDR1 AND m_lipid_and_fatty-acid_binding_n13-ARG81 THEN 010
010: 0.007204 (1644,2,140,2)

IF RAP1-ARG81 AND m_lipid_and_fatty-acid_binding_n13-ARG81 THEN 010
010: 0.007204 (1644,2,140,2)

IF m_anion_transporters_n22-RAP1 AND MCM1'-PDC2 THEN 010
010: 0.007204 (1644,2,140,2)

IF RAP1-ZAP1 AND m_nitrogen_and_sulphur_transport_n9-FHL1 THEN 010
010: 0.007204 (1644,2,140,2)

IF RAP1-GAT3 AND RAP1-HIR2 THEN 010
010: 0.007204 (1644,2,140,2)

IF m_utilization_of_vitamins_cofactors_and_prosthetic_groups_n7-DAL81 AND m_lipid_and_fatty-acid_binding_n13-UME1 THEN 010
010: 0.007204 (1644,2,140,2)

IF m_anion_transporters_n22-RAP1 AND ALPHA1-DAT1 THEN 010
010: 0.007204 (1644,2,140,2)

IF m_lipid_and_fatty-acid_binding_n13-ARG81 AND m_lipid_and_fatty-acid_binding_n13-UME1 THEN 010
010: 0.007204 (1644,2,140,2)

IF m_utilization_of_vitamins_cofactors_and_prosthetic_groups_n7-DAL81 AND RAP1-HAP4 THEN 010
010: 0.007204 (1644,2,140,2)

IF m_utilization_of_vitamins_cofactors_and_prosthetic_groups_n7-HSF1 AND m_other_morphogenetic_activities_n7-STP1 THEN 010
010: 0.007204 (1644,2,140,2)

IF RAP1-HAP4 AND m_lipid_and_fatty-acid_binding_n13-ARG81 THEN 010
010: 0.007204 (1644,2,140,2)

IF STE12-IXR1 AND RAP1-HAP4 THEN 010
010: 0.007204 (1644,2,140,2)

IF m_lipid_and_fatty-acid_binding_n15-FHL1 AND RAP1-DAT1 THEN 010
010: 0.007204 (1644,2,140,2)

IF RAP1-DAT1 AND m_lipid_and_fatty-acid_binding_n13-ARG81 THEN 010
010: 0.007204 (1644,2,140,2)

IF m_anion_transporters_n22-HAP4 AND ALPHA1-GAT3 THEN 010
010: 0.007204 (1644,2,140,2)

IF MCM1'-PDC2 AND RAP1-HAP4 THEN 010
010: 0.007204 (1644,2,140,2)

IF m_anion_transporters_n22-RAP1 AND m_lipid_and_fatty-acid_binding_n13-ARG81 THEN 010
010: 0.007204 (1644,2,140,2)

IF m_anion_transporters_n22-HAP4 AND STE12-IXR1 THEN 010
010: 0.007204 (1644,2,140,2)

IF m_anion_transporters_n22-RAP1 AND m_lipid_and_fatty-acid_binding_n13-PDR1 THEN 010
010: 0.007204 (1644,2,140,2)

IF STE12-IXR1 AND RAP1-RAP1 THEN 010
010: 0.007204 (1644,2,140,2)

IF STE12-IXR1 AND m_lipid_and_fatty-acid_binding_n13-ARG81 THEN 010

010: 0.007204 (1644,2,140,2)

IF m_anion_transporters_n22-HAP4 AND RAP1-GAT3 THEN 010
010: 0.007204 (1644,2,140,2)

IF m_anion_transporters_n22-HAP4 AND RAP1-YAP5 THEN 010
010: 0.007204 (1644,2,140,2)

IF m_anion_transporters_n22-HAP4 AND m_lipid_and_fatty-acid_binding_n13-ARG81 THEN 010
010: 0.007204 (1644,2,140,2)

IF m_utilization_of_vitamins_cofactors_and_prosthetic_groups_n7-DAL81 AND RAP1-GAT3 THEN 010
010: 0.007204 (1644,2,140,2)

IF SFF'-HIR3 AND m_anion_transporters_n22-RAP1 THEN 010
010: 0.007204 (1644,2,140,2)

IF SFF'-HIR1 AND m_lipid_and_fatty-acid_binding_n15-FHL1 THEN 010
010: 0.007204 (1644,2,140,2)

IF m_anion_transporters_n22-RAP1 AND STE12-IXR1 THEN 010
010: 0.007204 (1644,2,140,2)

IF m_other_morphogenetic_activities_n7-ARR1 AND m_lipid_and_fatty-acid_binding_n13-ARG81 THEN 010
010: 0.007204 (1644,2,140,2)

IF MCM1'-PDC2 AND RAP1-FHL1 THEN 010
010: 0.007204 (1644,2,140,2)

IF SFF'-HIR3 AND m_lipid_and_fatty-acid_binding_n13-PDR1 THEN 010
010: 0.007204 (1644,2,140,2)

IF m_metabolism_of_cyclic_and_unusual_nucleotides_n5-YAP1 AND m_other_cation_transporters_n14-MET32 THEN 010
010: 0.007204 (1644,2,140,2)

IF MET31-32-MET32 AND m_other_cation_transporters_n14-MET32 THEN 010
010: 0.007204 (1644,2,140,2)

IF m_other_morphogenetic_activities_n7-ARR1 AND RAP1-ZAP1 THEN 010
010: 0.007204 (1644,2,140,2)

IF m_anion_transporters_n22-HAP4 AND RAP1-HAP4 THEN 010
010: 0.007204 (1644,2,140,2)

IF m_utilization_of_vitamins_cofactors_and_prosthetic_groups_n7-DAL81 AND RAP1-ZAP1 THEN 010
010: 0.007204 (1644,2,140,2)

IF RAP1-HAP4 AND m_lipid_and_fatty-acid_binding_n13-UME1 THEN 010
010: 0.007204 (1644,2,140,2)

IF RAP1-HIR2 AND ALPHA1-GAT3 THEN 010
010: 0.007204 (1644,2,140,2)

IF STE12-IXR1 AND m_nitrogen_and_sulphur_transport_n9-FHL1 THEN 010
010: 0.007204 (1644,2,140,2)

IF m_other_morphogenetic_activities_n7-ARR1 AND RAP1-DAT1 THEN 010
010: 0.007204 (1644,2,140,2)

IF m_utilization_of_vitamins_cofactors_and_prosthetic_groups_n7-DAL81 AND RAP1-HIR2 THEN 010
010: 0.007204 (1644,2,140,2)

IF m_other_morphogenetic_activities_n7-ARR1 AND m_lipid_and_fatty-acid_binding_n13-PDR1 THEN 010
010: 0.007204 (1644,2,140,2)

IF m_g-proteins_n12-NRG1 AND m_amino-acid_transporters_n11-PHD1 THEN 010
010: 0.007204 (1644,2,140,2)

IF SFF'-HIR3 AND STE12-IXR1 THEN 010
010: 0.007204 (1644,2,140,2)

IF MCM1'-PDC2 AND RAP1-DAT1 THEN 010
010: 0.007204 (1644,2,140,2)

IF m_anion_transporters_n22-RAP1 AND RAP1-ARO80 THEN 010
010: 0.007204 (1644,2,140,2)

IF SFF'-HIR1 AND m_utilization_of_vitamins_cofactors_and_prosthetic_groups_n7-STP2 THEN 010
010: 0.007204 (1644,2,140,2)

IF ALPHA1-GAT3 AND m_lipid_and_fatty-acid_binding_n13-UME1 THEN 010
010: 0.007204 (1644,2,140,2)

IF m_anion_transporters_n22-RAP1 AND m_lipid_and_fatty-acid_binding_n15-FHL1 THEN 010
010: 0.007204 (1644,2,140,2)

IF m_anion_transporters_n22-HAP4 AND RAP1-ZAP1 THEN 010
010: 0.007204 (1644,2,140,2)

IF STE12-IXR1 AND m_lipid_and_fatty-acid_binding_n15-FHL1 THEN 010
010: 0.007204 (1644,2,140,2)

IF RAP1-ARO80 AND m_nitrogen_and_sulphur_transport_n9-FHL1 THEN 010
010: 0.007204 (1644,2,140,2)

IF RAP1-ZAP1 AND m_lipid_and_fatty-acid_binding_n13-UME1 THEN 010
010: 0.007204 (1644,2,140,2)

IF SFF-HIR1 AND m_lipid_and_fatty-acid_binding_n13-ARG81 THEN 010
010: 0.007204 (1644,2,140,2)

IF m_anion_transporters_n22-HAP4 AND m_other_morphogenetic_activities_n7-ARR1 THEN 010
010: 0.007204 (1644,2,140,2)

IF STE12-IXR1 AND RAP1-HIR2 THEN 010
010: 0.007204 (1644,2,140,2)

IF m_anion_transporters_n22-HAP4 AND RAP1-ARO80 THEN 010
010: 0.007204 (1644,2,140,2)

IF SFF'-HIR1 AND ALPHA1-DAT1 THEN 010
010: 0.007204 (1644,2,140,2)

IF m_anion_transporters_n22-HAP4 AND m_lipid_and_fatty-acid_binding_n15-STP2 THEN 010
010: 0.007204 (1644,2,140,2)

IF m_other_morphogenetic_activities_n7-ARR1 AND RAP1-ARO80 THEN 010
010: 0.007204 (1644,2,140,2)

IF m_anion_transporters_n22-RAP1 AND RAP1-HIR2 THEN 010
010: 0.007204 (1644,2,140,2)

IF m_g-proteins_n12-INO4 AND m_g-proteins_n11-INO4 AND m_other_energy_generation_activities_n22-INO4 THEN 010
010: 0.007204 (1644,2,140,2)

IF m_g-proteins_n12-INO4 AND m_g-proteins_n11-INO4 AND m_phosphate_transport_n5-INO4 THEN 010
010: 0.007204 (1644,2,140,2)

IF m_g-proteins_n11-INO4 AND m_phosphate_transport_n5-INO4 AND m_other_energy_generation_activities_n22-INO4 THEN 010
010: 0.007204 (1644,2,140,2)

IF m_g-proteins_n12-INO4 AND m_nutritional_response_pathway_n7-SWI6 AND m_anion_transporters_n10-INO4 THEN 010
010: 0.007204 (1644,2,140,2)

IF SCB-UME6 AND m_other_energy_generation_activities_n22-INO4 THEN 010
010: 0.007204 (1644,2,140,2)

IF m_g-proteins_n11-HAL9 AND ALPHA1'-YBR239c THEN 010
010: 0.007204 (1644,2,140,2)

IF m_mitochondrial_biogenesis_n5-MAL13 AND m_other_proteolytic_degradation_n2-SFP1 THEN 010
010: 0.007204 (1644,2,140,2)

IF m_regulation_of_amino-acid_metabolism_n7-RAP1 AND PHO4-CBF1 THEN 010
010: 0.007204 (1644,2,140,2)

IF m_RPE11-RAP1 AND PHO4-CBF1 THEN 010
010: 0.007204 (1644,2,140,2)

IF m_other_cation_transporters_n14-RAP1 AND m_regulation_of_amino-acid_metabolism_n7-CBF1 THEN 010
010: 0.007204 (1644,2,140,2)

IF m_regulation_of_amino-acid_metabolism_n7-CBF1 AND m_regulation_of_amino-acid_metabolism_n7-RAP1 THEN 010
010: 0.007204 (1644,2,140,2)

IF m_other_cation_transporters_n14-RAP1 AND m_regulation_of_amino-acid_metabolism_n7-RAP1 THEN 010
010: 0.007204 (1644,2,140,2)

IF m_RPE11-RAP1 AND m_regulation_of_amino-acid_metabolism_n7-CBF1 THEN 010
010: 0.007204 (1644,2,140,2)

IF m_utilization_of_vitamins_cofactors_and_prosthetic_groups_n7-DAL81 AND RAP1-FHL1 THEN 010
010: 0.007204 (1644,2,140,2)

IF m_lipid_and_fatty-acid_binding_n15-STP2 AND RAP1-RAP1 THEN 010
010: 0.007204 (1644,2,140,2)

IF RAP1-HAP4 AND m_nitrogen_and_sulphur_transport_n9-FHL1 THEN 010
010: 0.007204 (1644,2,140,2)

IF MCM1'-PDC2 AND ALPHA1-DAT1 THEN 010
010: 0.007204 (1644,2,140,2)

IF RAP1-HIR2 AND m_lipid_and_fatty-acid_binding_n13-PDR1 THEN 010
010: 0.007204 (1644,2,140,2)

IF ALPHA1-GAT3 AND m_lipid_and_fatty-acid_binding_n13-PDR1 THEN 010
010: 0.007204 (1644,2,140,2)

IF m_utilization_of_vitamins_cofactors_and_prosthetic_groups_n7-DAL81 AND m_nitrogen_and_sulphur_transport_n9-FHL1 THEN 010
010: 0.007204 (1644,2,140,2)

IF RAP1-HAP4 AND ALPHA1-GAT3 THEN 010
010: 0.007204 (1644,2,140,2)

IF m_other_morphogenetic_activities_n7-HOG1 AND m_metabolism_of_energy_reserves_n30-MIG3 THEN 010
010: 0.007204 (1644,2,140,2)

IF m_other_morphogenetic_activities_n7-GCR2 AND m_metabolism_of_energy_reserves_n30-MIG3 THEN 010
010: 0.007204 (1644,2,140,2)

IF SFF-HOG1 AND m_metabolism_of_energy_reserves_n30-MIG3 THEN 010
010: 0.007204 (1644,2,140,2)

IF m_other_morphogenetic_activities_n7-GCR2 AND m_metabolism_of_energy_reserves_n30-EDS1 THEN 010
010: 0.007204 (1644,2,140,2)

IF SFF'-HIR3 AND m_metabolism_of_energy_reserves_n30-MIG3 THEN 010
010: 0.007204 (1644,2,140,2)

IF m_other_morphogenetic_activities_n7-HOG1 AND m_metabolism_of_energy_reserves_n30-EDS1 THEN 010
010: 0.007204 (1644,2,140,2)

IF SFF'-HIR3 AND m_metabolism_of_energy_reserves_n30-EDS1 THEN 010
010: 0.007204 (1644,2,140,2)

IF SFF-HOG1 AND m_metabolism_of_energy_reserves_n30-EDS1 THEN 010
010: 0.007204 (1644,2,140,2)

IF SFF-ARG80 AND m_metabolism_of_energy_reserves_n30-EDS1 THEN 010

010: 0.007204 (1644,2,140,2)

IF m_metabolism_of_energy_reserves_n30-EDS1 AND m_metabolism_of_energy_reserves_n30-MIG3 THEN 010
010: 0.007204 (1644,2,140,2)

IF SFF-ARG80 AND m_metabolism_of_energy_reserves_n30-MIG3 THEN 010
010: 0.007204 (1644,2,140,2)

IF m_lipid_and_fatty-acid_binding_n15-STP2 AND m_nitrogen_and_sulphur_transport_n9-FHL1 THEN 010
010: 0.007204 (1644,2,140,2)

IF RAP1-RAP1 AND m_lipid_and_fatty-acid_binding_n13-UME1 THEN 010
010: 0.007204 (1644,2,140,2)

IF ALPHA1'-RIM101 AND m_chromatin_modification_n21-HSF1 THEN 010
010: 0.007204 (1644,2,140,2)

IF RAP1-FHL1 AND m_lipid_and_fatty-acid_binding_n13-UME1 THEN 010
010: 0.007204 (1644,2,140,2)

IF SFF'-HIR1 AND m_lipid_and_fatty-acid_binding_n13-UME1 THEN 010
010: 0.007204 (1644,2,140,2)

IF m_anion_transporters_n22-HAP4 AND m_lipid_and_fatty-acid_binding_n13-UME1 THEN 010
010: 0.007204 (1644,2,140,2)

IF m_utilization_of_vitamins_cofactors_and_prosthetic_groups_n7-DAL81 AND ALPHA1-DAT1 THEN 010
010: 0.007204 (1644,2,140,2)

IF m_anion_transporters_n22-HAP4 AND RAP1-FHL1 THEN 010
010: 0.007204 (1644,2,140,2)

IF SFF-HIR1 AND m_nitrogen_and_sulphur_transport_n9-FHL1 THEN 010
010: 0.007204 (1644,2,140,2)

IF m_utilization_of_vitamins_cofactors_and_prosthetic_groups_n7-DAL81 AND m_lipid_and_fatty-acid_binding_n15-STP2 THEN 010
010: 0.007204 (1644,2,140,2)

IF SFF'-HIR1 AND MCM1'-PDC2 THEN 010
010: 0.007204 (1644,2,140,2)

IF m_utilization_of_vitamins_cofactors_and_prosthetic_groups_n7-DAL81 AND RAP1-YAP5 THEN 010
010: 0.007204 (1644,2,140,2)

IF MCM1'-PDC2 AND RAP1-HIR2 THEN 010
010: 0.007204 (1644,2,140,2)

IF m_lipid_and_fatty-acid_binding_n15-STP2 AND RAP1-FHL1 THEN 010
010: 0.007204 (1644,2,140,2)

IF m_lipid_and_fatty-acid_binding_n15-STP2 AND ALPHA1-GAT3 THEN 010
010: 0.007204 (1644,2,140,2)

IF m_anion_transporters_n22-HAP4 AND m_nitrogen_and_sulphur_transport_n9-FHL1 THEN 010
010: 0.007204 (1644,2,140,2)

IF m_other_transcription_activities_n5-SWI6 AND SCB-AZF1 THEN 010
010: 0.007204 (1644,2,140,2)

IF m_anion_transporters_n22-RAP1 AND m_lipid_and_fatty-acid_binding_n13-UME1 THEN 010
010: 0.007204 (1644,2,140,2)

IF SFF'-HIR3 AND m_nitrogen_and_sulphur_transport_n9-FHL1 THEN 010
010: 0.007204 (1644,2,140,2)

IF RAP1-HIR2 AND m_lipid_and_fatty-acid_binding_n13-ARG81 THEN 010
010: 0.007204 (1644,2,140,2)

IF m_anion_transporters_n22-HAP4 AND RAP1-HIR2 THEN 010
010: 0.007204 (1644,2,140,2)

IF m_utilization_of_vitamins_cofactors_and_prosthetic_groups_n7-DAL81 AND RAP1-ARO80 THEN 010
010: 0.007204 (1644,2,140,2)

IF MCM1'-PDC2 AND RAP1-PDR1 THEN 010
010: 0.007204 (1644,2,140,2)

IF STE12-IXR1 AND RAP1-GAT3 THEN 010
010: 0.007204 (1644,2,140,2)

IF m_utilization_of_vitamins_cofactors_and_prosthetic_groups_n7-STP2 AND m_nitrogen_and_sulphur_transport_n9-FHL1 THEN 010
010: 0.007204 (1644,2,140,2)

IF SFF'-MCM1 AND SFF-FKH1 AND ALPHA1'-RIM101 THEN 010
010: 0.007204 (1644,2,140,2)

IF m_utilization_of_vitamins_cofactors_and_prosthetic_groups_n7-DAL81 AND RAP1-PDR1 THEN 010
010: 0.007204 (1644,2,140,2)

IF m_g-proteins_n12-IME4 AND m_glyoxylate_cycle_n11-RGM1 THEN 010
010: 0.007204 (1644,2,140,2)

IF m_anion_transporters_n22-HAP4 AND RAP1-ARG81 THEN 010
010: 0.007204 (1644,2,140,2)

IF m_nitrogen_and_sulphur_transport_n9-FHL1 AND ALPHA1-DAT1 THEN 010
010: 0.007204 (1644,2,140,2)

IF MCM1'-PDC2 AND ALPHA1-GAT3 THEN 010
010: 0.007204 (1644,2,140,2)

IF MCM1'-PDC2 AND m_nitrogen_and_sulphur_transport_n9-FHL1 THEN 010
010: 0.007204 (1644,2,140,2)

IF m_utilization_of_vitamins_cofactors_and_prosthetic_groups_n7-DAL81 AND m_lipid_and_fatty-acid_binding_n13-PDR1 THEN 010
010: 0.007204 (1644,2,140,2)

IF STE12-IXR1 AND RAP1-ARO80 THEN 010
010: 0.007204 (1644,2,140,2)

IF SFF'-HIR1 AND m_lipid_and_fatty-acid_binding_n13-PDR1 THEN 010
010: 0.007204 (1644,2,140,2)

IF m_nitrogen_and_sulphur_transport_n9-FHL1 AND m_lipid_and_fatty-acid_binding_n13-PDR1 THEN 010
010: 0.007204 (1644,2,140,2)

IF m_anion_transporters_n22-HAP4 AND m_utilization_of_vitamins_cofactors_and_prosthetic_groups_n7-DAL81 THEN 010
010: 0.007204 (1644,2,140,2)

IF STE12-IXR1 AND m_lipid_and_fatty-acid_binding_n13-UME1 THEN 010
010: 0.007204 (1644,2,140,2)

IF m_nitrogen_and_sulphur_transport_n9-FHL1 AND m_lipid_and_fatty-acid_binding_n13-UME1 THEN 010
010: 0.007204 (1644,2,140,2)

IF RAP1-FHL1 AND m_lipid_and_fatty-acid_binding_n13-ARG81 THEN 010
010: 0.007204 (1644,2,140,2)

IF m_utilization_of_vitamins_cofactors_and_prosthetic_groups_n7-STP2 AND RAP1-DAT1 THEN 010
010: 0.007204 (1644,2,140,2)

IF m_utilization_of_vitamins_cofactors_and_prosthetic_groups_n7-STP2 AND m_lipid_and_fatty-acid_binding_n13-ARG81 THEN 010
010: 0.007204 (1644,2,140,2)

IF m_utilization_of_vitamins_cofactors_and_prosthetic_groups_n7-STP2 AND RAP1-RAP1 THEN 010
010: 0.007204 (1644,2,140,2)

IF m_utilization_of_vitamins_cofactors_and_prosthetic_groups_n7-STP2 AND SFF-HIR1 THEN 010
010: 0.007204 (1644,2,140,2)

IF RAP1-ARO80 AND m_lipid_and_fatty-acid_binding_n13-ARG81 THEN 010
010: 0.007204 (1644,2,140,2)

IF m_other_morphogenetic_activities_n7-ARR1 AND m_lipid_and_fatty-acid_binding_n15-FHL1 THEN 010
010: 0.007204 (1644,2,140,2)

IF m_lipid_and_fatty-acid_binding_n15-FHL1 AND m_lipid_and_fatty-acid_binding_n13-UME1 THEN 010
010: 0.007204 (1644,2,140,2)

IF RAP1-HIR2 AND ALPHA1-DAT1 THEN 010
010: 0.007204 (1644,2,140,2)

IF RAP1-HIR2 AND m_nitrogen_and_sulphur_transport_n9-FHL1 THEN 010
010: 0.007204 (1644,2,140,2)

IF MCM1'-PDC2 AND RAP1-GAT3 THEN 010
010: 0.007204 (1644,2,140,2)

IF m_organization_of_golgi_n7-FKH1 THEN 000 OR 010 OR 100 OR 110
110: 0.007252 (1644,12,115,4)

IF SFF'-FKH2 AND SFF'-MCM1 AND SFF-FKH1 THEN 000 OR 010 OR 100 OR 110 OR 111
110: 0.007252 (1644,12,115,4)

IF SFF'-FKH1 AND SFF'-MCM1 AND SFF-FKH2 THEN 000 OR 010 OR 100 OR 110 OR 111
110: 0.007252 (1644,12,115,4)

IF m_g-proteins_n11-INO4 AND m_other_mrna-transcription_activities_n20-INO4 THEN 000 OR 001 OR 010 OR 100 OR 110
110: 0.007252 (1644,12,115,4)

IF SFF'-MCM1 AND SFF-FKH1 AND SFF-FKH2 THEN 000 OR 010 OR 100 OR 110 OR 111
110: 0.007252 (1644,12,115,4)

IF m_other_transcription_activities_n5-SWI6 THEN 000 OR 010 OR 100 OR 101 OR 110
100: 0.007315 (1644,11,127,4)

IF m_anion_transporters_n17-INO4 THEN 000 OR 001 OR 100 OR 110
100: 0.007382 (1644,17,127,5)

IF m_osmosensing_n6-INO4 THEN 000 OR 010 OR 100 OR 101 OR 110
110: 0.007437 (1644,26,115,6)

IF PAC-YDR026c THEN 000 OR 100
100: 0.007576 (1644,6,127,3)

IF m_purine_and_pyrimidine_transporters_n10-YAP1 THEN 000 OR 100
100: 0.007576 (1644,6,127,3)

IF SWI5-INO4 AND m_organization_of_chromosome_structure_n12-INO4 THEN 000 OR 001 OR 100
100: 0.007576 (1644,6,127,3)

IF SFF'-FKH2 AND SFF'-MCM1 AND SFF-FKH1 THEN 000 OR 010 OR 100 OR 110 OR 111
111: 0.007798 (1644,12,19,2)

IF SFF'-FKH1 AND SFF'-MCM1 AND SFF-FKH2 THEN 000 OR 010 OR 100 OR 110 OR 111
111: 0.007798 (1644,12,19,2)

IF SFF'-FKH1 AND SFF'-FKH2 AND SFF'-MCM1 AND SFF-FKH2 THEN 000 OR 010 OR 100 OR 110 OR 111
111: 0.007798 (1644,12,19,2)

IF MCM1'-SWI4 AND mPROTEOL18(m_proteolysis_n18)-SWI6 THEN 000 OR 010 OR 100 OR 110 OR 111
111: 0.007798 (1644,12,19,2)

IF MCM1'-SWI4 AND m_amino-acid_transporters_n11-SWI4 THEN 000 OR 010 OR 111
111: 0.007798 (1644,12,19,2)

IF SFF'-FKH2 AND SWI5-SWI6 THEN 000 OR 001 OR 010 OR 100 OR 110 OR 111
110: 0.008071 (1644,19,115,5)

IF m_ionic_homeostasis_n6-SWI6 THEN 000 OR 010 OR 100 OR 101 OR 110

110: 0.008071 (1644,19,115,5)

IF m_amino-acid_transport_n20-RAP1 THEN 000 OR 001 OR 010 OR 100 OR 110
001: 0.008409 (1644,25,55,4)

IF m_regulation_of_lipid_fatty-acid_and_isoprenoid_biosynthesis_n8.scn-MTH1 THEN 000 OR 010 OR 100 OR 110
010: 0.008433 (1644,16,140,5)

IF m_pentose-phosphate_pathway_n14-FKH1 THEN 000 OR 010 OR 100 OR 110 OR 111
110: 0.009026 (1644,27,115,6)

IF m_metabolism_of_energy_reserves_n27-SKN7 AND SWI5-SKN7 THEN 000 OR 111
111: 0.009153 (1644,13,19,2)

IF m_ion_transporters_n11-SKN7 THEN 000 OR 100 OR 111
111: 0.009153 (1644,13,19,2)

IF SFF'-FKH2 AND SCB-UME6 THEN 000 OR 010 OR 110
110: 0.009484 (1644,7,115,3)

IF ECB-MCM1 AND ECB-STE12 THEN 000 OR 010 OR 100 OR 110
110: 0.009484 (1644,7,115,3)

IF MCM1'-SWI4 AND m_ionic_homeostasis_n6-SWI6 THEN 000 OR 101 OR 110
110: 0.009484 (1644,7,115,3)

IF m_abc_transporters_n10-SWI6 THEN 000 OR 100 OR 110 OR 111
110: 0.009484 (1644,7,115,3)

IF SFF'-FKH1 AND MCM1'-NDD1 AND m_other_morphogenetic_activities_n7-MCM1 THEN 000 OR 010 OR 100 OR 110
110: 0.009484 (1644,7,115,3)

IF m_allantoin_and_allantoate_transporters_n18-FKH2 AND SFF-FKH2 THEN 000 OR 100 OR 110
110: 0.009484 (1644,7,115,3)

IF m_cytoskeleton-dependenttransport_n4-STB1 THEN 000 OR 100 OR 110
110: 0.009484 (1644,7,115,3)

IF SWI5-SWI6 AND m_ionic_homeostasis_n6-SWI6 THEN 000 OR 010 OR 101 OR 110
110: 0.009484 (1644,7,115,3)

IF ECB-MCM1 AND m_nucleotide_transport_n9-MCM1 THEN 000 OR 010 OR 100 OR 110
110: 0.009484 (1644,7,115,3)

IF SFF'-FKH1 AND MCM1'-NDD1 AND ECB-FKH2 THEN 000 OR 010 OR 110 OR 111
110: 0.009484 (1644,7,115,3)

IF SFF'-FKH1 AND ECB-NDD1 THEN 000 OR 010 OR 110 OR 111
110: 0.009484 (1644,7,115,3)

IF MCM1-FKH2 AND MCM1-SWI4 THEN 000 OR 010 OR 100 OR 110
110: 0.009484 (1644,7,115,3)

IF SFF'-FKH2 AND MCM1-YOX1 THEN 000 OR 010 OR 110 OR 111
110: 0.009484 (1644,7,115,3)

IF SFF'-FKH1 AND m_osmosensing_n6-INO4 THEN 000 OR 010 OR 100 OR 110
110: 0.009484 (1644,7,115,3)

IF SFF'-RLM1 AND MCM1'-NDD1 THEN 000 OR 110 OR 111
110: 0.009484 (1644,7,115,3)

IF m_allantoin_and_allantoate_transporters_n7-ACE2 AND SWI5-SWI6 THEN 000 OR 110 OR 111
110: 0.009484 (1644,7,115,3)

IF m_nutritional_response_pathway_n7-SWI6 THEN 000 OR 010 OR 101 OR 110 OR 111
101: 0.009541 (1644,23,11,2)

IF SWI5-SWI6 AND mPROTEOL18(m_proteolysis_n18)-SWI6 THEN 000 OR 010 OR 100 OR 110 OR 111
110: 0.009917 (1644,13,115,4)

IF m_other_mrna-transcription_activities_n20-RAP1 AND m_amino-acid_transport_n20-RAP1 THEN 000 OR 001 OR 110
001: 0.009931 (1644,14,55,3)

IF SFF'-HIR3 AND RAP1-PDR1 AND ALPHA1-GAT3 THEN 000 OR 010
010: 0.009983 (1644,6,140,3)

IF SFF'-RTG3 AND ECB-YOX1 THEN 000 OR 010 OR 110
010: 0.009983 (1644,6,140,3)

IF m_peroxisomal_transport_n22-RAP1 AND RAP1-RAP1 THEN 000 OR 010
010: 0.009983 (1644,6,140,3)

IF SFF'-HIR3 AND RAP1-FHL1 AND RAP1-GAT3 THEN 000 OR 010
010: 0.009983 (1644,6,140,3)

IF AFT1-PDR1 THEN 000 OR 010
010: 0.009983 (1644,6,140,3)

IF ALPHA1-DAT1 AND ALPHA1-GAT3 THEN 000 OR 010 OR 110
010: 0.009983 (1644,6,140,3)

IF SFF'-HIR3 AND RAP1-FHL1 AND RAP1-RAP1 AND ALPHA1-GAT3 THEN 000 OR 010
010: 0.009983 (1644,6,140,3)

IF SFF'-HIR3 AND MCM1'-PDC2 THEN 000 OR 001 OR 010 OR 100
010: 0.009983 (1644,6,140,3)

IF SFF'-HIR3 AND RAP1-GAT3 THEN 000 OR 010
010: 0.009983 (1644,6,140,3)

IF SFF'-HIR3 AND RAP1-FHL1 AND RAP1-YAP5 AND ALPHA1-GAT3 THEN 000 OR 010
010: 0.009983 (1644,6,140,3)

IF SFF'-HIR3 AND RAP1-GAT3 AND RAP1-PDR1 AND RAP1-RAP1 THEN 000 OR 010
010: 0.009983 (1644,6,140,3)

IF SFF'-HIR3 AND RAP1-GAT3 AND RAP1-YAP5 THEN 000 OR 010
010: 0.009983 (1644,6,140,3)

IF SFF'-HIR3 AND RAP1-GAT3 AND RAP1-RAP1 THEN 000 OR 010
010: 0.009983 (1644,6,140,3)

IF m_anion_transporters_n4-MTH1 AND m_deoxyribonucleotide_metabolism_n23-MTH1 THEN 000 OR 010
010: 0.009983 (1644,6,140,3)

IF m_other_proteolytic_degradation_n2-MTH1 AND m_deoxyribonucleotide_metabolism_n23-MTH1 THEN 000 OR 010
010: 0.009983 (1644,6,140,3)

IF SFF'-HIR3 AND RAP1-GAT3 AND RAP1-PDR1 AND ALPHA1-GAT3 THEN 000 OR 010
010: 0.009983 (1644,6,140,3)

IF SFF'-HIR3 AND RAP1-GAT3 AND RAP1-PDR1 THEN 000 OR 010
010: 0.009983 (1644,6,140,3)

IF SFF'-HIR3 AND RAP1-FHL1 AND ALPHA1-GAT3 THEN 000 OR 010
010: 0.009983 (1644,6,140,3)

IF SFF'-HIR3 AND RAP1-RAP1 AND ALPHA1-GAT3 THEN 000 OR 010
010: 0.009983 (1644,6,140,3)

IF SFF'-HIR3 AND RAP1-PDR1 AND RAP1-RAP1 AND RAP1-YAP5 AND ALPHA1-GAT3 THEN 000 OR 010
010: 0.009983 (1644,6,140,3)

IF ABF1-ABF1 AND ALPHA1'-RIM101 THEN 000 OR 010
010: 0.009983 (1644,6,140,3)

IF SFF'-HIR3 AND RAP1-YAP5 AND ALPHA1-GAT3 THEN 000 OR 010
010: 0.009983 (1644,6,140,3)

IF m_regulation_of_lipid_fatty-acid_and_isoprenoid_biosynthesis_n8.scn-FKH1 THEN 000 OR 010 OR 100 OR 110 OR 111
110: 0.01017 (1644,20,115,5)

IF m_osmosensing_n6-SWI4 THEN 000 OR 010 OR 100 OR 101 OR 110 OR 111
110: 0.01017 (1644,20,115,5)

IF m_lysosomal_and_vacuolar_degradation_n8-HAP3 THEN 000 OR 001
001: 0.010302 (1644,5,55,2)

IF m_regulation_of_nitrogen_and_sulphur_utilization_n13-MTH1 AND m_other_nutritional-response_activities_n10-MTH1 THEN 000 OR 001 OR 100
001: 0.010302 (1644,5,55,2)

IF m_glyoxylate_cycle_n8-MTH1 AND m_regulation_of_nitrogen_and_sulphur_utilization_n13-MTH1 THEN 000 OR 001
001: 0.010302 (1644,5,55,2)

IF m_RPE21-RGM1 AND m_amino-acid_transport_n20-RAP1 THEN 000 OR 001
001: 0.010302 (1644,5,55,2)

IF m_RPE21-FHL1 AND m_amino-acid_transport_n20-RAP1 THEN 000 OR 001
001: 0.010302 (1644,5,55,2)

IF SWI5-INO4 AND ALPHA1'-SFP1 THEN 000 OR 001 OR 010
001: 0.010302 (1644,5,55,2)

IF m_nutritional_response_pathway_n8-INO4 AND m_other_mrna-transcription_activities_n20-RAP1 THEN 000 OR 001
001: 0.010302 (1644,5,55,2)

IF m_other_mrna-transcription_activities_n20-INO4 AND ALPHA1'-SFP1 THEN 000 OR 001 OR 110
001: 0.010302 (1644,5,55,2)

IF OAF1-SFP1 THEN 000 OR 001
001: 0.010302 (1644,5,55,2)

IF m_g-proteins_n12-INO4 AND SWI5-INO4 AND m_nucleotide_transport_n9-INO4 THEN 000 OR 001 OR 110
001: 0.010302 (1644,5,55,2)

IF m_other_mrna-transcription_activities_n20-RAP1 AND m_RPE68-RAP1 THEN 000 OR 001 OR 100
001: 0.010302 (1644,5,55,2)

IF m_utilization_of_vitamins_cofactors_and_prosthetic_groups_n7-RPN4 THEN 000 OR 010 OR 100
010: 0.010356 (1644,11,140,4)

IF SFF'-RLM1 AND SCB-AZF1 THEN 000 OR 010 OR 011 OR 111
111: 0.010605 (1644,14,19,2)

IF m_pentose-phosphate_pathway_n5-SWI4 THEN 000 OR 100 OR 101 OR 110 OR 111
111: 0.010605 (1644,14,19,2)

IF m_amino-acid_transporters_n11-SKN7 AND m_amino-acid_transporters_n11-SWI4 THEN 000 OR 010 OR 110 OR 111
111: 0.010605 (1644,14,19,2)

IF m_other_nucleotide-metabolism_activities_n18-PHO2 THEN 000 OR 010 OR 100
010: 0.011143 (1644,17,140,5)

IF SFF-MIG1 THEN 000 OR 001 OR 010 OR 100 OR 110
010: 0.011634 (1644,61,140,11)

IF PDR-INO4 AND m_g-proteins_n12-INO4 THEN 000 OR 001 OR 100
001: 0.012123 (1644,15,55,3)

IF m_ion_transporters_n11-SWI6 THEN 000 OR 010 OR 100 OR 110 OR 111
111: 0.012153 (1644,15,19,2)

IF m_amino-acid_degradation_n27-INO4 THEN 000 OR 100
100: 0.012515 (1644,7,127,3)

IF SFF'-MCM1 AND m_nucleotide_transport_n9-MCM1 THEN 000 OR 010 OR 100 OR 101 OR 110
110: 0.013145 (1644,14,115,4)

IF m_allantoin_and_allantoate_transporters_n18-FKH2 THEN 000 OR 010 OR 100 OR 110
110: 0.013145 (1644,14,115,4)

IF m_allantoin_and_allantoate_transporters_n12-ACE2 THEN 000 OR 001 OR 110
110: 0.013145 (1644,14,115,4)

IF m_other_pheromone_response_activities_n8-SWI6 THEN 000 OR 001 OR 010 OR 110 OR 111
110: 0.013145 (1644,14,115,4)

IF m_ion_transporters_n11-SWI4 THEN 000 OR 100 OR 110 OR 111
111: 0.013793 (1644,16,19,2)

IF m_organization_of_intracellular_transport_vesicles_n5-SWI4 AND MCM1'-SWI4 AND m_cell_death_n22-TOS8 THEN 000 OR 110
110: 0.013893 (1644,3,115,2)

IF MCM1-FKH2 AND SCB-UME6 THEN 010 OR 110
110: 0.013893 (1644,3,115,2)

IF MCM1-FKH2 AND MCM1'-SWI4 AND ECB-FKH2 AND SFF-FKH2 THEN 000 OR 110
110: 0.013893 (1644,3,115,2)

IF MCM1-SWI4 AND MCM1'-SWI4 AND ECB-FKH2 AND SFF-FKH2 THEN 000 OR 110
110: 0.013893 (1644,3,115,2)

IF Gcr1-SWI6 AND SWI5-SWI6 AND mPROTEOL18(m_proteolysis_n18)-MBP1 AND mPROTEOL18(m_proteolysis_n18)-SWI6 THEN 000 OR 110
110: 0.013893 (1644,3,115,2)

IF m_breakdown_of_lipids_fatty_acids_and_isoprenoids_n8-MBP1 AND m_other_transport_facilitators_n10-SWI6 THEN 000 OR 110
110: 0.013893 (1644,3,115,2)

IF m_breakdown_of_lipids_fatty_acids_and_isoprenoids_n8-SWI6 AND m_other_transport_facilitators_n10-SWI6 THEN 000 OR 110
110: 0.013893 (1644,3,115,2)

IF m_nitrogen_and_sulphur_metabolism_n17-SKN7 AND SFF-FKH1 THEN 000 OR 110
110: 0.013893 (1644,3,115,2)

IF m_g-proteins_n12-SKN7 AND m_sugar_and_carbohydrate_transporters_n6-SKN7 THEN 000 OR 110
110: 0.013893 (1644,3,115,2)

IF SFF-FKH1 AND m_organization_of_chromosome_structure_n17-SKN7 THEN 100 OR 110
110: 0.013893 (1644,3,115,2)

IF m_MERE4-SKN7 AND m_organization_of_chromosome_structure_n17-SKN7 THEN 000 OR 110
110: 0.013893 (1644,3,115,2)

IF m_sugar_and_carbohydrate_transporters_n6-SKN7 AND m_other_protein-destination_activities_n7-MET4 THEN 000 OR 110
110: 0.013893 (1644,3,115,2)

IF SFF'-FKH2 AND ndt80(MSE)-YDR049W THEN 000 OR 110
110: 0.013893 (1644,3,115,2)

IF SFF-FKH1 AND ndt80(MSE)-YER130C THEN 000 OR 110
110: 0.013893 (1644,3,115,2)

IF SFF-FKH1 AND ndt80(MSE)-YDR049W THEN 000 OR 110
110: 0.013893 (1644,3,115,2)

IF SFF'-FKH2 AND ndt80(MSE)-YER130C THEN 000 OR 110
110: 0.013893 (1644,3,115,2)

IF m_phosphate_transport_n8-SWI4 AND m_other_transport_facilitators_n10-SWI6 THEN 010 OR 110
110: 0.013893 (1644,3,115,2)

IF m_osmosensing_n6-INO4 AND MCM1'-MCM1 THEN 100 OR 110
110: 0.013893 (1644,3,115,2)

IF ECB-DIG1 AND ECB-NDD1 THEN 000 OR 110
110: 0.013893 (1644,3,115,2)

IF SFF'-MCM1 AND m_osmosensing_n6-INO4 THEN 100 OR 110
110: 0.013893 (1644,3,115,2)

IF SFF-FKH2 AND ndt80(MSE)-YDR049W THEN 000 OR 110
110: 0.013893 (1644,3,115,2)

IF m_osmosensing_n6-INO4 AND MCM1'-NDD1 THEN 100 OR 110
110: 0.013893 (1644,3,115,2)

IF SWI5-SWI6 AND m_other_transport_facilitators_n10-PIP2 THEN 000 OR 110
110: 0.013893 (1644,3,115,2)

IF Gcr1-SWI6 AND SWI5-SWI6 AND LYS14-SWI6 AND mPROTEOL18(m_proteolysis_n18)-MBP1 THEN 000 OR 110
110: 0.013893 (1644,3,115,2)

IF m_osmosensing_n6-INO4 AND m_drug_transporters_n10-SKN7 THEN 000 OR 110
110: 0.013893 (1644,3,115,2)

IF MCM1-FKH2 AND ECB-DIG1 THEN 000 OR 110
110: 0.013893 (1644,3,115,2)

IF SFF-FKH2 AND ndt80(MSE)-YER130C THEN 000 OR 110
110: 0.013893 (1644,3,115,2)

IF ECB-DIG1 AND SFF-FKH2 THEN 000 OR 110
110: 0.013893 (1644,3,115,2)

IF m_osmosensing_n6-ROX1 AND MCM1'-MCM1 THEN 000 OR 110
110: 0.013893 (1644,3,115,2)

IF m_regulation_of_lipid_fatty-acid_and_isoprenoid_biosynthesis_n8.scn-FKH1 AND m_glyoxylate_cycle_n11-GAT1 THEN 010 OR 110
110: 0.013893 (1644,3,115,2)

IF SFF'-FKH1 AND m_regulation_of_lipid_fatty-acid_and_isoprenoid_biosynthesis_n8.scn-GAT1 AND m_glyoxylate_cycle_n11-GAT1 THEN 010 OR 110
110: 0.013893 (1644,3,115,2)

IF m_g-proteins_n11-INO4 AND m_other_transport_facilitators_n5-SWI6 THEN 010 OR 110
110: 0.013893 (1644,3,115,2)

IF Gcr1-SWI6 AND SWI5-SWI6 AND mPROTEOL18(m_proteolysis_n18)-SWI6 THEN 000 OR 110
110: 0.013893 (1644,3,115,2)

IF m_allantoin_and_allantoate_transporters_n7-ACE2 AND SWI5-SWI6 AND m_allantoin_and_allantoate_transporters_n12-ACE2 THEN 000 OR 110
110: 0.013893 (1644,3,115,2)

IF SFF'-FKH1 AND ECB-STE12 THEN 010 OR 110
110: 0.013893 (1644,3,115,2)

IF SWI5-SWI6 AND LYS14-SWI6 AND mPROTEOL18(m_proteolysis_n18)-SWI6 THEN 000 OR 110
110: 0.013893 (1644,3,115,2)

IF Gcr1-SWI6 AND SWI5-SWI6 AND mPROTEOL18(m_proteolysis_n18)-MBP1 THEN 000 OR 110
110: 0.013893 (1644,3,115,2)

IF MCM1'-NDD1 AND m_other_cell_growth_cell_division_and_dna_synthesis_activities_n10.scn-NDD1 THEN 010 OR 110
110: 0.013893 (1644,3,115,2)

IF SFF'-FKH1 AND m_drug_transporters_n10-INO4 THEN 000 OR 110
110: 0.013893 (1644,3,115,2)

IF PHO-NDD1 AND m_other_cell_growth_cell_division_and_dna_synthesis_activities_n10.scn-NDD1 THEN 010 OR 110
110: 0.013893 (1644,3,115,2)

IF ECB-YOX1 AND SFF-FKH1 THEN 110 OR 111
110: 0.013893 (1644,3,115,2)

IF MCM1-FKH2 AND m_other_cell_growth_cell_division_and_dna_synthesis_activities_n10.scn-NDD1 THEN 010 OR 110
110: 0.013893 (1644,3,115,2)

IF ECB-NDD1 AND m_other_cell_growth_cell_division_and_dna_synthesis_activities_n10.scn-NDD1 THEN 010 OR 110
110: 0.013893 (1644,3,115,2)

IF m_other_energy_generation_activities_n12-SWI6 AND SFF-FKH1 THEN 000 OR 110
110: 0.013893 (1644,3,115,2)

IF SFF'-RLM1 AND ECB-STE12 THEN 000 OR 110
110: 0.013893 (1644,3,115,2)

IF SFF'-FKH2 AND MCM1'-NDD1 AND SWI5-INO4 THEN 100 OR 110
110: 0.013893 (1644,3,115,2)

IF m_other_signal-transduction_activities_n8-INO4 AND m_homeostasis_of_other_ions_n30-INO4 THEN 001 OR 110
110: 0.013893 (1644,3,115,2)

IF ECB-STE12 AND mRRPE-RLM1 THEN 000 OR 110
110: 0.013893 (1644,3,115,2)

IF ECB-DIG1 AND ECB-FKH2 AND ECB-MCM1 THEN 000 OR 110
110: 0.013893 (1644,3,115,2)

IF SFF'-FKH1 AND SFF'-FKH2 AND ndt80(MSE)-YER130C THEN 000 OR 110
110: 0.013893 (1644,3,115,2)

IF MCM1-FKH2 AND ECB-DIG1 AND ECB-FKH2 THEN 000 OR 110
110: 0.013893 (1644,3,115,2)

IF SFF'-FKH1 AND MCM1'-NDD1 AND SWI5-INO4 THEN 100 OR 110
110: 0.013893 (1644,3,115,2)

IF SFF'-FKH1 AND m_other_energy_generation_activities_n12-SWI6 THEN 000 OR 110
110: 0.013893 (1644,3,115,2)

IF SFF'-FKH1 AND SFF'-MCM1 AND ALPHA1'-UPC2 THEN 010 OR 110
110: 0.013893 (1644,3,115,2)

IF m_other_mrna-transcription_activities_n20-INO4 AND m_phosphate_transport_n18-INO4 AND m_homeostasis_of_other_ions_n30-INO4 THEN 000 OR 110
110: 0.013893 (1644,3,115,2)

IF ECB-YOX1 AND ALPHA1'-STB4 THEN 010 OR 110
110: 0.013893 (1644,3,115,2)

IF MCM1'-NDD1 AND SFF-FKH1 AND SWI5-INO4 THEN 100 OR 110
110: 0.013893 (1644,3,115,2)

IF m_osmosensing_n6-ROX1 AND MCM1'-MCM1 AND ALPHA1'-UPC2 THEN 000 OR 110
110: 0.013893 (1644,3,115,2)

IF SFF'-FKH2 AND m_g-proteins_n12-INO4 AND MCM1'-NDD1 THEN 000 OR 110
110: 0.013893 (1644,3,115,2)

IF MCM1-YOX1 AND ALPHA1'-STB4 THEN 010 OR 110
110: 0.013893 (1644,3,115,2)

IF m_other_signal-transduction_activities_n8-INO4 AND m_phosphate_transport_n8-INO4 THEN 000 OR 110
110: 0.013893 (1644,3,115,2)

IF m_anion_transporters_n32-INO4 AND m_anion_transporters_n20-RAP1 THEN 000 OR 110
110: 0.013893 (1644,3,115,2)

IF SFF'-RLM1 AND MCM1'-MCM1 AND MCM1'-NDD1 THEN 110 OR 111
110: 0.013893 (1644,3,115,2)

IF SFF-FKH2 AND mRRPE-RLM1 THEN 000 OR 110
110: 0.013893 (1644,3,115,2)

IF m_deoxyribonucleotide_metabolism_n12-TOS8 AND m_cell_death_n16-MBP1 THEN 000 OR 110
110: 0.013893 (1644,3,115,2)

IF SFF'-FKH2 AND mRRPE-RLM1 THEN 000 OR 110
110: 0.013893 (1644,3,115,2)

IF SFF'-MCM1 AND ECB-TEC1 THEN 000 OR 110
110: 0.013893 (1644,3,115,2)

IF m_pheromone_response_generation_n12-FKH1 AND m_other_pheromone_response_activities_n8-SWI6 THEN 010 OR 110
110: 0.013893 (1644,3,115,2)

IF m_osmosensing_n6-SWI4 AND m_drug_transporters_n10-INO4 THEN 100 OR 110
110: 0.013893 (1644,3,115,2)

IF m_other_mrna-transcription_activities_n20-INO4 AND m_anion_transporters_n32-INO4 THEN 000 OR 110
110: 0.013893 (1644,3,115,2)

IF SFF'-RLM1 AND m_osmosensing_n6-INO4 THEN 000 OR 110
110: 0.013893 (1644,3,115,2)

IF SFF'-RLM1 AND ECB-TEC1 THEN 010 OR 110
110: 0.013893 (1644,3,115,2)

IF m_pheromone_response_generation_n12-FKH1 AND m_nutritional_response_pathway_n7-SWI6 THEN 010 OR 110
110: 0.013893 (1644,3,115,2)

IF MCM1'-SWI4 AND ALPHA1'-UPC2 THEN 000 OR 110
110: 0.013893 (1644,3,115,2)

IF SFF'-MCM1 AND SFF'-RLM1 AND MCM1'-SWI4 THEN 000 OR 110
110: 0.013893 (1644,3,115,2)

IF m_other_mrna-transcription_activities_n20-RAP1 AND m_anion_transporters_n32-INO4 THEN 000 OR 110
110: 0.013893 (1644,3,115,2)

IF m_cell_death_n22-TOS8 AND m_cell_death_n16-MBP1 THEN 000 OR 110
110: 0.013893 (1644,3,115,2)

IF SWI5-SWI6 AND LYS14-SWI6 AND mPROTEOL18(m_proteolysis_n18)-MBP1 THEN 000 OR 110
110: 0.013893 (1644,3,115,2)

IF SFF'-MCM1 AND SFF-FKH1 AND ALPHA1'-UPC2 THEN 010 OR 110
110: 0.013893 (1644,3,115,2)

IF SFF'-RLM1 AND MCM1'-MCM1 AND MCM1'-SWI4 THEN 000 OR 110
110: 0.013893 (1644,3,115,2)

IF m_sugar_and_carbohydrate_transporters_n6-SKN7 AND m_other_protein-destination_activities_n7-SKN7 THEN 000 OR 110
110: 0.013893 (1644,3,115,2)

IF m_organization_of_intracellular_transport_vesicles_n5-SWI4 AND m_cell_death_n22-TOS8 THEN 000 OR 110
110: 0.013893 (1644,3,115,2)

IF m_cytok9-SWI6 AND m_nutritional_response_pathway_n7-SWI6 AND MCB-SWI6 THEN 010 OR 110
110: 0.013893 (1644,3,115,2)

IF m_g-proteins_n12-INO4 AND MCB-SWI6 THEN 000 OR 110
110: 0.013893 (1644,3,115,2)

IF m_osmosensing_n6-INO4 AND MCB-SWI6 THEN 000 OR 110
110: 0.013893 (1644,3,115,2)

IF m_glyoxylate_cycle_n8-INO4 AND m_other_mrna-transcription_activities_n20-INO4 AND m_phosphate_transport_n18-INO4 THEN 000 OR 110
110: 0.013893 (1644,3,115,2)

IF MCM1-YOX1 AND MCM1'-SWI4 THEN 000 OR 110
110: 0.013893 (1644,3,115,2)

IF MCM1'-SWI4 AND ECB-YOX1 THEN 000 OR 110
110: 0.013893 (1644,3,115,2)

IF mPROTEOL18(m_proteolysis_n18)-MBP1 AND m_deoxyribonucleotide_metabolism_n27-SWI6 THEN 000 OR 110
110: 0.013893 (1644,3,115,2)

IF m_deoxyribonucleotide_metabolism_n27-SWI6 AND STRE-SWI6 THEN 000 OR 110
110: 0.013893 (1644,3,115,2)

IF MCM1-SWI4 AND mPROTEOL18(m_proteolysis_n18)-SWI6 THEN 010 OR 110
110: 0.013893 (1644,3,115,2)

IF SFF'-RLM1 AND LYS14-SWI6 THEN 000 OR 110
110: 0.013893 (1644,3,115,2)

IF MCM1-MCM1 AND SCB-SWI4 THEN 100 OR 110
110: 0.013893 (1644,3,115,2)

IF MCM1-SWI4 AND SCB-UME6 THEN 010 OR 110
110: 0.013893 (1644,3,115,2)

IF m_allantoin_and_allantoate_transporters_n13-SWI6 AND m_deoxyribonucleotide_metabolism_n10-SWI6 THEN 000 OR 110
110: 0.013893 (1644,3,115,2)

IF m_allantoin_and_allantoate_transporters_n13-SWI6 AND SCB-SWI4 THEN 000 OR 110
110: 0.013893 (1644,3,115,2)

IF MCM1-SWI4 AND mPROTEOL18(m_proteolysis_n18)-MBP1 THEN 010 OR 110
110: 0.013893 (1644,3,115,2)

IF m_organization_of_intracellular_transport_vesicles_n5-SWI4 AND m_phosphate_transport_n8-SWI4 THEN 000 OR 110
110: 0.013893 (1644,3,115,2)

IF MCM1'-MCM1 AND m_other_cell_growth_cell_division_and_dna_synthesis_activities_n10.scn-NDD1 THEN 010 OR 110
110: 0.013893 (1644,3,115,2)

IF SFF'-FKH1 AND MCM1-YOX1 AND SFF-FKH2 THEN 110 OR 111
110: 0.013893 (1644,3,115,2)

IF MCM1-MCM1 AND m_other_cell_growth_cell_division_and_dna_synthesis_activities_n10.scn-NDD1 THEN 010 OR 110
110: 0.013893 (1644,3,115,2)

IF SFF'-MCM1 AND SCB-SWI4 THEN 110 OR 111
110: 0.013893 (1644,3,115,2)

IF ECB-FKH2 AND m_other_cell_growth_cell_division_and_dna_synthesis_activities_n10.scn-NDD1 THEN 010 OR 110
110: 0.013893 (1644,3,115,2)

IF ECB-MCM1 AND m_other_cell_growth_cell_division_and_dna_synthesis_activities_n10.scn-NDD1 THEN 010 OR 110
110: 0.013893 (1644,3,115,2)

IF SWI5-SWI6 AND m_biosynthesis_of_vitamins_cofactors_and_prosthetic_groups_n8-MET4 THEN 010 OR 110
110: 0.013893 (1644,3,115,2)

IF SFF'-FKH2 AND m_osmosensing_n6-SWI4 AND SCB-SWI4 THEN 110 OR 111
110: 0.013893 (1644,3,115,2)

IF MCM1'-NDD1 AND m_ionic_homeostasis_n6-SWI6 THEN 000 OR 110
110: 0.013893 (1644,3,115,2)

IF MCM1-SWI4 AND ECB-FKH2 AND SFF-FKH2 THEN 000 OR 110
110: 0.013893 (1644,3,115,2)

IF SWI5-INO4 AND m_amino-acid_transporters_n11-SKN7 THEN 001 OR 110
110: 0.013893 (1644,3,115,2)

IF m_osmosensing_n6-SWI4 AND MCM1-SWI4 THEN 100 OR 110
110: 0.013893 (1644,3,115,2)

IF m_osmosensing_n6-ROX1 AND SFF-FKH1 AND ALPHA1'-UPC2 THEN 010 OR 110
110: 0.013893 (1644,3,115,2)

IF MCM1-SWI4 AND ECB-NDD1 AND SFF-FKH2 THEN 000 OR 110
110: 0.013893 (1644,3,115,2)

IF SFF'-MCM1 AND SFF'-RLM1 AND MCM1'-NDD1 THEN 110 OR 111
110: 0.013893 (1644,3,115,2)

IF m_other_transport_facilitators_n5-INO4 AND m_other_transport_facilitators_n5-SWI6 THEN 010 OR 110
110: 0.013893 (1644,3,115,2)

IF ECB-DIG1 AND STE12-STE12 THEN 100 OR 110
110: 0.013893 (1644,3,115,2)

IF m_pentose-phosphate_pathway_n14-FKH1 AND SCB-UME6 THEN 000 OR 110
110: 0.013893 (1644,3,115,2)

IF m_lipid_transporters_n8-MET4 AND SWI5-SKN7 THEN 000 OR 110
110: 0.013893 (1644,3,115,2)

IF MCM1'-MCM1 AND MCM1'-SWI4 AND ECB-FKH2 THEN 000 OR 110
110: 0.013893 (1644,3,115,2)

IF MCM1'-MCM1 AND MCM1'-SWI4 AND SFF-FKH1 THEN 100 OR 110
110: 0.013893 (1644,3,115,2)

IF ECB-DIG1 AND STE12-DIG1 THEN 100 OR 110
110: 0.013893 (1644,3,115,2)

IF LYS14-SWI6 AND m_nucleotide_transport_n9-SWI6 THEN 000 OR 110
110: 0.013893 (1644,3,115,2)

IF m_other_proteolytic_degradation_n7-SWI6 AND m_nucleotide_transport_n9-SWI6 THEN 000 OR 110
110: 0.013893 (1644,3,115,2)

IF m_lipid_transporters_n8-MET4 AND m_organization_of_chromosome_structure_n17-SKN7 THEN 000 OR 110
110: 0.013893 (1644,3,115,2)

IF STE12-STE12 AND STE12-TEC1 THEN 000 OR 110
110: 0.013893 (1644,3,115,2)

IF ECB-STE12 AND STE12-STE12 THEN 100 OR 110
110: 0.013893 (1644,3,115,2)

IF ECB-FKH2 AND m_other_morphogenetic_activities_n7-MCM1 AND PHO-NDD1 THEN 010 OR 110
110: 0.013893 (1644,3,115,2)

IF SFF'-FKH1 AND MCM1-NDD1 AND m_nucleotide_transport_n9-MCM1 THEN 010 OR 110
110: 0.013893 (1644,3,115,2)

IF SFF'-FKH1 AND MCM1-MCM1 AND m_nucleotide_transport_n9-MCM1 THEN 010 OR 110
110: 0.013893 (1644,3,115,2)

IF SFF'-FKH2 AND m_other_transport_facilitators_n10-PIP2 THEN 110 OR 111
110: 0.013893 (1644,3,115,2)

IF SFF'-FKH1 AND MCM1-FKH2 AND m_nucleotide_transport_n9-MCM1 THEN 010 OR 110
110: 0.013893 (1644,3,115,2)

IF SFF'-FKH2 AND m_other_morphogenetic_activities_n7-MCM1 AND PHO-NDD1 THEN 010 OR 110
110: 0.013893 (1644,3,115,2)

IF m_allantoin_and_allantoate_transporters_n18-FKH2 AND m_nucleotide_transport_n9-MCM1 THEN 010 OR 110
110: 0.013893 (1644,3,115,2)

IF m_allantoin_and_allantoate_transporters_n18-FKH2 AND ECB-MCM1 THEN 010 OR 110
110: 0.013893 (1644,3,115,2)

IF MCM1-FKH2 AND ECB-MCM1 AND m_nucleotide_transport_n9-MCM1 THEN 010 OR 110
110: 0.013893 (1644,3,115,2)

IF MCM1-FKH2 AND m_other_morphogenetic_activities_n7-MCM1 AND m_nucleotide_transport_n9-MCM1 THEN 010 OR 110

110: 0.013893 (1644,3,115,2)

IF MCM1-NDD1 AND ECB-MCM1 AND m_nucleotide_transport_n9-MCM1 THEN 010 OR 110
110: 0.013893 (1644,3,115,2)

IF MCM1-MCM1 AND ECB-FKH2 AND m_nucleotide_transport_n9-MCM1 THEN 010 OR 110
110: 0.013893 (1644,3,115,2)

IF MCM1-NDD1 AND ECB-FKH2 AND m_nucleotide_transport_n9-MCM1 THEN 010 OR 110
110: 0.013893 (1644,3,115,2)

IF ECB-FKH2 AND m_other_morphogenetic_activities_n7-MCM1 AND m_nucleotide_transport_n9-MCM1 THEN 010 OR 110
110: 0.013893 (1644,3,115,2)

IF m_phosphate_transport_n8-SWI4 AND m_cytoskeleton-dependenttransport_n4-ACE2 THEN 000 OR 110
110: 0.013893 (1644,3,115,2)

IF m_allantoin_and_allantoate_transporters_n7-ACE2 AND m_phosphate_transport_n8-SWI4 THEN 000 OR 110
110: 0.013893 (1644,3,115,2)

IF MCM1-MCM1 AND ECB-NDD1 AND m_nucleotide_transport_n9-MCM1 THEN 010 OR 110
110: 0.013893 (1644,3,115,2)

IF m_MERE4-MTH1 AND m_pentose-phosphate_pathway_n14-FKH1 THEN 000 OR 110
110: 0.013893 (1644,3,115,2)

IF MCM1-NDD1 AND MCM1'-MCM1 AND m_nucleotide_transport_n9-MCM1 THEN 010 OR 110
110: 0.013893 (1644,3,115,2)

IF MCM1-SWI4 AND ECB-YOX1 THEN 000 OR 110
110: 0.013893 (1644,3,115,2)

IF MCM1-SWI4 AND MCM1-YOX1 THEN 000 OR 110
110: 0.013893 (1644,3,115,2)

IF MCM1'-MCM1 AND MCM1'-SWI4 AND ECB-NDD1 THEN 000 OR 110
110: 0.013893 (1644,3,115,2)

IF ECB-STE12 AND STE12-DIG1 THEN 100 OR 110
110: 0.013893 (1644,3,115,2)

IF ECB-NDD1 AND m_other_morphogenetic_activities_n7-MCM1 AND m_nucleotide_transport_n9-MCM1 THEN 010 OR 110
110: 0.013893 (1644,3,115,2)

IF m_other_pheromone_response_activities_n8-SWI6 AND MCB-SWI6 THEN 010 OR 110
110: 0.013893 (1644,3,115,2)

IF MCM1-FKH2 AND ECB-NDD1 AND m_nucleotide_transport_n9-MCM1 THEN 010 OR 110
110: 0.013893 (1644,3,115,2)

IF m_other_pheromone_response_activities_n8-SWI6 AND MCB-MBP1 THEN 010 OR 110
110: 0.013893 (1644,3,115,2)

IF STE12-DIG1 AND STE12-TEC1 THEN 000 OR 110
110: 0.013893 (1644,3,115,2)

IF MCM1-FKH2 AND MCM1'-MCM1 AND m_nucleotide_transport_n9-MCM1 THEN 010 OR 110
110: 0.013893 (1644,3,115,2)

IF m_metal_ion_transporters_n10-SWI6 AND STRE'-MBP1 THEN 000 OR 110
110: 0.013893 (1644,3,115,2)

IF MCM1-NDD1 AND ECB-NDD1 AND m_nucleotide_transport_n9-MCM1 THEN 010 OR 110
110: 0.013893 (1644,3,115,2)

IF SFF'-FKH2 AND m_other_morphogenetic_activities_n7-MCM1 AND m_nucleotide_transport_n9-MCM1 THEN 010 OR 110
110: 0.013893 (1644,3,115,2)

IF MCM1-NDD1 AND m_other_morphogenetic_activities_n7-MCM1 AND m_nucleotide_transport_n9-MCM1 THEN 010 OR 110
110: 0.013893 (1644,3,115,2)

IF MCM1-FKH2 AND ECB-FKH2 AND m_nucleotide_transport_n9-MCM1 THEN 010 OR 110
110: 0.013893 (1644,3,115,2)

IF MCM1-FKH2 AND m_other_morphogenetic_activities_n7-MCM1 AND PHO-NDD1 THEN 010 OR 110
110: 0.013893 (1644,3,115,2)

IF SFF'-FKH2 AND SFF'-MCM1 AND m_other_cell_growth_cell_division_and_dna_synthesis_activities_n10.scn-NDD1 THEN 010 OR 110
110: 0.013893 (1644,3,115,2)

IF SFF'-FKH1 AND SFF'-FKH2 AND m_nucleotide_transport_n9-MCM1 THEN 010 OR 110
110: 0.013893 (1644,3,115,2)

IF m_lipid_and_fatty-acid_transport_n11-MTH1 AND m_LFTE17-GAT1 AND m_anion_transporters_n20-GAT1 THEN 110 OR 111
110: 0.013893 (1644,3,115,2)

IF m_LFTE17-GAT1 AND m_anion_transporters_n20-GAT1 AND m_anion_transporters_n20-MTH1 THEN 110 OR 111
110: 0.013893 (1644,3,115,2)

IF m_organization_of_golgi_n7-FKH1 AND m_g-proteins_n11-INO4 THEN 000 OR 110
110: 0.013893 (1644,3,115,2)

IF m_g-proteins_n11-INO4 AND SWI5-INO4 AND m_pentose-phosphate_pathway_n14-MTH1 THEN 000 OR 110
110: 0.013893 (1644,3,115,2)

IF m_metal_ion_transporters_n10-SWI6 AND MCB-MBP1 THEN 100 OR 110
110: 0.013893 (1644,3,115,2)

IF m_metal_ion_transporters_n10-SWI6 AND MCB-SWI6 THEN 100 OR 110
110: 0.013893 (1644,3,115,2)

IF m_g-proteins_n11-INO4 AND SWI5-SWI6 AND m_other_transport_facilitators_n5-SWI6 THEN 010 OR 110
110: 0.013893 (1644,3,115,2)

IF SWI5-SWI6 AND m_metal_ion_transporters_n14-GAT1 THEN 000 OR 110
110: 0.013893 (1644,3,115,2)

IF SWI5-INO4 AND m_deoxyribonucleotide_metabolism_n5-MTH1 THEN 000 OR 110
110: 0.013893 (1644,3,115,2)

IF m_breakdown_of_lipids_fatty_acids_and_isoprenoids_n8-INO4 AND m_other_mrna-transcription_activities_n20-INO4 THEN 000 OR 110
110: 0.013893 (1644,3,115,2)

IF SFF-FKH2 AND SWI5-SWI6 AND mPROTEOL18(m_proteolysis_n18)-SWI6 THEN 000 OR 110
110: 0.013893 (1644,3,115,2)

IF m_abc_transporters_n10-SWI6 AND SFF-FKH2 THEN 000 OR 110
110: 0.013893 (1644,3,115,2)

IF m_allantoin_and_allantoate_transporters_n13-SWI6 AND m_glyoxylate_cycle_n19-SWI6 THEN 000 OR 110
110: 0.013893 (1644,3,115,2)

IF m_allantoin_and_allantoate_transporters_n13-SWI6 AND m_regulation_of_amino-acid_metabolism_n11-SWI6 THEN 000 OR 110
110: 0.013893 (1644,3,115,2)

IF SFF'-FKH2 AND m_abc_transporters_n10-SWI6 THEN 000 OR 110
110: 0.013893 (1644,3,115,2)

IF m_allantoin_and_allantoate_transporters_n12-ACE2 AND m_other_transport_facilitators_n10-SWI6 THEN 000 OR 110
110: 0.013893 (1644,3,115,2)

IF SWI5-SWI6 AND mPROTEOL18(m_proteolysis_n18)-MBP1 AND m_nucleotide_transport_n9-SWI6 THEN 000 OR 110
110: 0.013893 (1644,3,115,2)

IF SFF'-FKH1 AND m_nutritional_response_pathway_n7-SWI6 THEN 010 OR 110
110: 0.013893 (1644,3,115,2)

IF SWI5-SWI6 AND m_allantoin_and_allantoate_transporters_n13-SWI6 AND m_nucleotide_transport_n9-SWI6 THEN 101 OR 110
110: 0.013893 (1644,3,115,2)

IF SFF-FKH1 AND m_nutritional_response_pathway_n7-SWI6 THEN 010 OR 110
110: 0.013893 (1644,3,115,2)

IF SFF'-FKH2 AND SFF-FKH2 AND SWI5-SWI6 AND mPROTEOL18(m_proteolysis_n18)-MBP1 AND mPROTEOL18(m_proteolysis_n18)-SWI6 THEN 000 OR 110
110: 0.013893 (1644,3,115,2)

IF m_other_cation_transporters_n7-SWI6 AND m_nucleotide_transport_n9-SWI6 THEN 010 OR 110
110: 0.013893 (1644,3,115,2)

IF m_allantoin_and_allantoate_transporters_n7-ACE2 AND SWI5-SWI6 AND m_other_transport_facilitators_n10-SWI6 THEN 000 OR 110
110: 0.013893 (1644,3,115,2)

IF m_LFTE17-GAT1 AND m_anion_transporters_n20-MTH1 THEN 110 OR 111
110: 0.013893 (1644,3,115,2)

IF m_MERE4-MTH1 AND m_pentose-phosphate_pathway_n14-FKH1 AND m_pentose-phosphate_pathway_n14-MTH1 THEN 000 OR 110
110: 0.013893 (1644,3,115,2)

IF m_cytoskeleton-dependenttransport_n4-ACE2 AND m_allantoin_and_allantoate_transporters_n6-PHO4 THEN 000 OR 110
110: 0.013893 (1644,3,115,2)

IF SWI5-SWI6 AND m_other_mrna-transcription_activities_n20-INO4 AND m_other_transport_facilitators_n5-SWI6 THEN 010 OR 110
110: 0.013893 (1644,3,115,2)

IF SWI5-INO4 AND SWI5-SWI6 AND m_other_transport_facilitators_n5-SWI6 THEN 010 OR 110
110: 0.013893 (1644,3,115,2)

IF m_cytoskeleton-dependenttransport_n4-MBP1 AND m_allantoin_and_allantoate_transporters_n6-PHO4 THEN 000 OR 110
110: 0.013893 (1644,3,115,2)

IF SFF-FKH2 AND m_ionic_homeostasis_n6-SWI6 THEN 000 OR 110
110: 0.013893 (1644,3,115,2)

IF SFF'-FKH2 AND m_ionic_homeostasis_n6-SWI6 THEN 000 OR 110
110: 0.013893 (1644,3,115,2)

IF m_allantoin_and_allantoate_transporters_n6-PHO4 AND m_other_transport_facilitators_n10-OAF1 THEN 000 OR 110
110: 0.013893 (1644,3,115,2)

IF SFF-FKH1 AND m_anion_transporters_n20-MTH1 THEN 000 OR 110
110: 0.013893 (1644,3,115,2)

IF SFF'-FKH2 AND SFF-FKH2 AND SWI5-SWI6 AND mPROTEOL18(m_proteolysis_n18)-MBP1 THEN 000 OR 110
110: 0.013893 (1644,3,115,2)

IF m_breakdown_of_lipids_fatty_acids_and_isoprenoids_n8-MBP1 AND m_allantoin_and_allantoate_transporters_n6-PHO4 THEN 000 OR 110
110: 0.013893 (1644,3,115,2)

IF SFF'-FKH1 AND m_anion_transporters_n20-MTH1 THEN 000 OR 110
110: 0.013893 (1644,3,115,2)

IF m_breakdown_of_lipids_fatty_acids_and_isoprenoids_n8-SWI6 AND m_phosphate_transport_n8-SWI4 THEN 000 OR 110
110: 0.013893 (1644,3,115,2)

IF SFF'-FKH2 AND m_other_transport_facilitators_n10-OAF1 THEN 110 OR 111
110: 0.013893 (1644,3,115,2)

IF SFF'-FKH2 AND m_cytoskeleton-dependenttransport_n4-ACE2 THEN 110 OR 111
110: 0.013893 (1644,3,115,2)

IF SFF'-FKH2 AND SFF-FKH2 AND SWI5-SWI6 AND mPROTEOL18(m_proteolysis_n18)-SWI6 THEN 000 OR 110
110: 0.013893 (1644,3,115,2)

IF m_breakdown_of_lipids_fatty_acids_and_isoprenoids_n8-MBP1 AND m_phosphate_transport_n8-SWI4 THEN 000 OR 110
110: 0.013893 (1644,3,115,2)

IF SFF-FKH2 AND SWI5-SWI6 AND mPROTEOL18(m_proteolysis_n18)-MBP1 THEN 000 OR 110
110: 0.013893 (1644,3,115,2)

IF SFF-FKH2 AND m_cytoskeleton-dependenttransport_n4-ACE2 THEN 110 OR 111
110: 0.013893 (1644,3,115,2)

IF SFF'-FKH2 AND m_cell_rescue_defense_cell_death_and_ageing_n20-PHO4 THEN 000 OR 110
110: 0.013893 (1644,3,115,2)

IF SFF-FKH2 AND m_cell_rescue_defense_cell_death_and_ageing_n20-PHO4 THEN 000 OR 110
110: 0.013893 (1644,3,115,2)

IF m_allantoin_and_allantoate_transporters_n7-ACE2 AND m_other_transport_facilitators_n10-SWI6 THEN 000 OR 110
110: 0.013893 (1644,3,115,2)

IF m_allantoin_and_allantoate_transporters_n7-ACE2 AND m_allantoin_and_allantoate_transporters_n6-PHO4 THEN 000 OR 110
110: 0.013893 (1644,3,115,2)

IF m_breakdown_of_lipids_fatty_acids_and_isoprenoids_n8-SWI6 AND m_cytoskeleton-dependenttransport_n4-ACE2 THEN 110 OR 111
110: 0.013893 (1644,3,115,2)

IF m_breakdown_of_lipids_fatty_acids_and_isoprenoids_n8-SWI6 AND m_cytoskeleton-dependenttransport_n4-MBP1 THEN 110 OR 111
110: 0.013893 (1644,3,115,2)

IF SFF-FKH1 AND SWI5-SWI6 AND m_allantoin_and_allantoate_transporters_n13-SWI6 THEN 110 OR 111
110: 0.013893 (1644,3,115,2)

IF SFF'-FKH1 AND SWI5-SWI6 AND m_allantoin_and_allantoate_transporters_n13-SWI6 THEN 110 OR 111
110: 0.013893 (1644,3,115,2)

IF m_g-proteins_n12-SKN7 AND SWI5-SKN7 AND m_amino-acid_transporters_n11-SWI4 THEN 010 OR 110
110: 0.013893 (1644,3,115,2)

IF LYS14-SWI6 AND m_nitrogen_and_sulphur_metabolism_n16-SWI6 THEN 000 OR 110
110: 0.013893 (1644,3,115,2)

IF MCM1-SWI4 AND MCB-MBP1 THEN 000 OR 110
110: 0.013893 (1644,3,115,2)

IF MCM1-SWI4 AND MCB-SWI6 THEN 000 OR 110
110: 0.013893 (1644,3,115,2)

IF MCM1'-SWI4 AND MCB-MBP1 THEN 000 OR 110
110: 0.013893 (1644,3,115,2)

IF m_nitrogen_and_sulphur_metabolism_n16-SWI6 AND mPROTEOL18(m_proteolysis_n18)-MBP1 THEN 000 OR 110
110: 0.013893 (1644,3,115,2)

IF m_allantoin_and_allantoate_transporters_n18-HAP2 AND m_metal_ion_transporters_n25-HAP4 THEN 000 OR 110
110: 0.013893 (1644,3,115,2)

IF m_allantoin_and_allantoate_transporters_n18-HAP2 AND HAP234-HAP4 THEN 000 OR 110
110: 0.013893 (1644,3,115,2)

IF m_g-proteins_n12-SKN7 AND SWI5-SWI6 AND m_nucleotide_transport_n9-SWI6 THEN 000 OR 110
110: 0.013893 (1644,3,115,2)

IF m_g-proteins_n12-SKN7 AND SWI5-SWI6 AND m_amino-acid_transporters_n11-SWI4 THEN 010 OR 110
110: 0.013893 (1644,3,115,2)

IF m_metal_ion_transporters_n14-GAT1 AND m_LFTE17-GAT1 THEN 000 OR 110
110: 0.013893 (1644,3,115,2)

IF SWI5-SWI6 AND m_other_transport_facilitators_n5-INO4 THEN 010 OR 110
110: 0.013893 (1644,3,115,2)

IF SFF'-MCM1 AND ECB-MCM1 AND m_pentose-phosphate_pathway_n5-MCM1 THEN 110 OR 111
110: 0.013893 (1644,3,115,2)

IF MCM1-MCM1 AND ECB-MCM1 AND m_pentose-phosphate_pathway_n5-MCM1 THEN 110 OR 111
110: 0.013893 (1644,3,115,2)

IF m_amino-acid_transporters_n11-SKN7 AND m_nucleotide_transport_n9-SWI6 THEN 000 OR 110

110: 0.013893 (1644,3,115,2)

IF m_g-proteins_n11-INO4 AND m_pentose-phosphate_pathway_n14-MTH1 THEN 000 OR 110
110: 0.013893 (1644,3,115,2)

IF m_lipid_and_fatty-acid_transport_n11-MTH1 AND m_other_mrna-transcription_activities_n20-SKN7 THEN 000 OR 110
110: 0.013893 (1644,3,115,2)

IF m_allantoin_and_allantoate_transporters_n13-SWI6 AND m_anion_transporters_n15-MTH1 THEN 000 OR 110
110: 0.013893 (1644,3,115,2)

IF m_g-proteins_n12-INO4 AND m_other_mrna-transcription_activities_n20-XBP1 THEN 001 OR 110
110: 0.013893 (1644,3,115,2)

IF SWI5-INO4 AND m_other_transport_facilitators_n5-SWI6 THEN 010 OR 110
110: 0.013893 (1644,3,115,2)

IF m_other_mrna-transcription_activities_n20-INO4 AND m_other_transport_facilitators_n5-SWI6 THEN 010 OR 110
110: 0.013893 (1644,3,115,2)

IF m_other_transport_facilitators_n5-CIN5 AND m_pentose-phosphate_pathway_n14-MTH1 THEN 000 OR 110
110: 0.013893 (1644,3,115,2)

IF SWI5-SWI6 AND m_OCSE15-MTH1 THEN 000 OR 110
110: 0.013893 (1644,3,115,2)

IF m_g-proteins_n11-INO4 AND m_other_mrna-transcription_activities_n20-XBP1 THEN 100 OR 110
110: 0.013893 (1644,3,115,2)

IF m_phosphate_transport_n13-SKN7 AND m_pentose-phosphate_pathway_n14-MTH1 THEN 010 OR 110
110: 0.013893 (1644,3,115,2)

IF m_other_transport_facilitators_n5-SWI6 AND m_pentose-phosphate_pathway_n14-MTH1 THEN 100 OR 110
110: 0.013893 (1644,3,115,2)

IF SWI5-SWI6 AND LYS14-SWI6 AND mPROTEOL18(m_proteolysis_n18)-MBP1 AND mPROTEOL18(m_proteolysis_n18)-SWI6 THEN 000 OR 110
110: 0.013893 (1644,3,115,2)

IF SFF'-RLM1 AND MCM1'-MCM1 THEN 000 OR 100 OR 110 OR 111
110: 0.014408 (1644,8,115,3)

IF m_stress_response_n24-SWI4 THEN 000 OR 010 OR 110
110: 0.014408 (1644,8,115,3)

IF SWI5-SWI6 AND m_amino-acid_transporters_n11-SKN7 THEN 000 OR 010 OR 110
110: 0.014408 (1644,8,115,3)

IF SFF'-FKH2 AND m_allantoin_and_allantoate_transporters_n7-ACE2 THEN 000 OR 100 OR 110 OR 111
110: 0.014408 (1644,8,115,3)

IF m_allantoin_and_allantoate_transporters_n7-ACE2 AND SFF-FKH2 THEN 000 OR 100 OR 110 OR 111
110: 0.014408 (1644,8,115,3)

IF m_pentose-phosphate_pathway_n14-GAT1 AND m_pentose-phosphate_pathway_n14-MTH1 THEN 000 OR 010 OR 100 OR 110
110: 0.014408 (1644,8,115,3)

IF m_glyoxylate_cycle_n8-INO4 AND m_other_mrna-transcription_activities_n20-INO4 THEN 000 OR 010 OR 110
110: 0.014408 (1644,8,115,3)

IF m_metal_ion_transporters_n14-GAT1 THEN 000 OR 110
110: 0.014408 (1644,8,115,3)

IF SFF'-FKH1 AND m_lipid_and_fatty-acid_transport_n11-GAT1 THEN 000 OR 010 OR 110
110: 0.014408 (1644,8,115,3)

IF ECB-FKH2 AND SFF-FKH1 THEN 000 OR 010 OR 110 OR 111
110: 0.014408 (1644,8,115,3)

IF SFF-FKH1 AND m_lipid_and_fatty-acid_transport_n11-GAT1 THEN 000 OR 010 OR 110

110: 0.014408 (1644,8,115,3)

IF SFF'-FKH2 AND m_osmosensing_n6-SWI4 THEN 000 OR 100 OR 110 OR 111
110: 0.014408 (1644,8,115,3)

IF SFF'-FKH1 AND SFF-FKH1 AND m_lipid_and_fatty-acid_transport_n11-GAT1 THEN 000 OR 010 OR 110
110: 0.014408 (1644,8,115,3)

IF SFF'-FKH1 AND SFF'-FKH2 AND m_other_morphogenetic_activities_n7-MCM1 THEN 000 OR 010 OR 100 OR 110 OR 111
110: 0.014408 (1644,8,115,3)

IF m_abc_transporters_n10-MBP1 THEN 000 OR 100 OR 110 OR 111
110: 0.014408 (1644,8,115,3)

IF MCM1'-SWI4 AND SFF-FKH1 THEN 000 OR 100 OR 110
110: 0.014408 (1644,8,115,3)

IF SFF'-FKH2 AND MCM1'-SWI4 AND SFF-FKH2 THEN 000 OR 100 OR 110
110: 0.014408 (1644,8,115,3)

IF MCM1'-SWI4 AND m_phosphate_transport_n8-SWI4 THEN 000 OR 110
110: 0.014408 (1644,8,115,3)

IF SFF'-HIR3 AND ALPHA1-DAT1 THEN 000 OR 010 OR 110
010: 0.014523 (1644,12,140,4)

IF ALPHA2-INO2 THEN 000 OR 010 OR 100
010: 0.014523 (1644,12,140,4)

IF m_RPE21-FHL1 THEN 000 OR 001 OR 010 OR 100
001: 0.014571 (1644,16,55,3)

IF m_pentose-phosphate_pathway_n23-RAP1 AND ALPHA1'-SFP1 THEN 000 OR 001
001: 0.015123 (1644,6,55,2)

IF m_RPE68-FHL1 AND m_RPE68-PDR1 THEN 000 OR 001 OR 010 OR 100
001: 0.015123 (1644,6,55,2)

IF m_RPE68-PDR1 AND m_RPE68-RAP1 THEN 000 OR 001 OR 010 OR 100
001: 0.015123 (1644,6,55,2)

IF m_RPE32-YAP5 THEN 000 OR 001
001: 0.015123 (1644,6,55,2)

IF m_other_mrna-transcription_activities_n20-RAP1 AND m_RPE21-FHL1 THEN 000 OR 001
001: 0.015123 (1644,6,55,2)

IF m_anion_transporters_n22-RAP1 AND m_anion_transporters_n22-SKN7 THEN 000 OR 001 OR 110
001: 0.015123 (1644,6,55,2)

IF m_glycolysis_and_gluconeogenesis_n11-CST6 THEN 000 OR 001
001: 0.015123 (1644,6,55,2)

IF STRE-RAP1 AND m_amino-acid_transport_n20-RAP1 THEN 000 OR 001 OR 010 OR 100
001: 0.015123 (1644,6,55,2)

IF m_g-proteins_n12-INO4 AND ALPHA1'-SFP1 THEN 000 OR 001
001: 0.015123 (1644,6,55,2)

IF m_cell_death_n16-INO4 THEN 000 OR 001 OR 010 OR 100 OR 110 OR 111
100: 0.015324 (1644,20,127,5)

IF PHO-INO4 THEN 000 OR 001 OR 010 OR 100 OR 101
100: 0.015324 (1644,20,127,5)

IF SFF'-FKH1 AND m_pentose-phosphate_pathway_n14-FKH1 THEN 000 OR 010 OR 100 OR 110 OR 111
110: 0.015435 (1644,22,115,5)

IF SFF-FKH2 AND SWI5-SWI6 THEN 000 OR 001 OR 100 OR 110 OR 111
111: 0.015525 (1644,17,19,2)

IF SFF'-MCM1 AND SFF-FKH1 THEN 000 OR 010 OR 100 OR 110 OR 111
111: 0.015525 (1644,17,19,2)

IF m_metabolism_of_energy_reserves_n27-SKN7 THEN 000 OR 111
111: 0.015525 (1644,17,19,2)

IF m_phosphate_transport_n18-XBP1 THEN 000 OR 001 OR 010 OR 100 OR 110 OR 111
111: 0.016299 (1644,48,19,3)

IF SFF-FKH1 AND ALPHA1'-RIM101 THEN 000 OR 010 OR 100 OR 110
010: 0.016389 (1644,7,140,3)

IF SFF'-HIR3 AND RAP1-PDR1 AND RAP1-YAP5 THEN 000 OR 010
010: 0.016389 (1644,7,140,3)

IF SFF'-HIR3 AND RAP1-YAP5 THEN 000 OR 010
010: 0.016389 (1644,7,140,3)

IF ABF1-YAP7 THEN 000 OR 001 OR 010
010: 0.016389 (1644,7,140,3)

IF m_anion_transporters_n22-HAP4 THEN 000 OR 001 OR 010 OR 100
010: 0.016389 (1644,7,140,3)

IF m_anion_transporters_n4-MTH1 AND m_other_proteolytic_degradation_n2-MTH1 THEN 000 OR 010
010: 0.016389 (1644,7,140,3)

IF m_osmosensing_n6-INO4 AND m_glyoxylate_cycle_n8-INO4 THEN 000 OR 010 OR 110
010: 0.016389 (1644,7,140,3)

IF RAP1-DAT1 THEN 000 OR 010 OR 100
010: 0.016389 (1644,7,140,3)

IF SFF'-RTG3 AND m_other_morphogenetic_activities_n7-GCR2 THEN 000 OR 010 OR 110
010: 0.016389 (1644,7,140,3)

IF SFF'-HIR3 AND RAP1-FHL1 AND RAP1-YAP5 THEN 000 OR 010
010: 0.016389 (1644,7,140,3)

IF m_phosphate_transport_n18-TOS8 THEN 000 OR 001 OR 010 OR 100 OR 110 OR 111
001: 0.016787 (1644,46,55,5)

IF SFF'-MCM1 AND SWI5-INO4 THEN 100 OR 110
100: 0.016871 (1644,3,127,2)

IF m_anion_transporters_n17-INO4 AND PHO-MET4 THEN 000 OR 100
100: 0.016871 (1644,3,127,2)

IF MCM1-MCM1 AND SWI5-INO4 THEN 100 OR 110
100: 0.016871 (1644,3,127,2)

IF m_pheromone_response_generation_n4-ECM22 THEN 010 OR 100
100: 0.016871 (1644,3,127,2)

IF SFF-MIG1 AND ATRepeat-UME6 THEN 010 OR 100
100: 0.016871 (1644,3,127,2)

IF m_amino-acid_transport_n18-MET4 AND m_amino-acid_transport_n18-TBS1 THEN 010 OR 100
100: 0.016871 (1644,3,127,2)

IF m_other_nutritional-response_activities_n6-PUT3 THEN 010 OR 100
100: 0.016871 (1644,3,127,2)

IF SFF'-RLM1 AND SFF-YER184C THEN 000 OR 100
100: 0.016871 (1644,3,127,2)

IF m_MERE11-SUT1 AND CSRE-UME6 THEN 010 OR 100
100: 0.016871 (1644,3,127,2)

IF PHO-MET4 AND m_pentose-phosphate_pathway_n23-INO4 THEN 000 OR 100
100: 0.016871 (1644,3,127,2)

IF SWI5-SKN7 AND m_pentose-phosphate_pathway_n23-INO4 THEN 000 OR 100
100: 0.016871 (1644,3,127,2)

IF PHO-INO4 AND m_purine_and_pyrimidine_transporters_n17-INO4 THEN 000 OR 100
100: 0.016871 (1644,3,127,2)

IF m_phosphate_transport_n18-INO4 AND m_purine_and_pyrimidine_transporters_n17-GAT1 THEN 000 OR 100
100: 0.016871 (1644,3,127,2)

IF m_ion_transporters_n4-GAT1 AND m_ion_transporters_n4-INO4 AND m_phosphate_transport_n18-GAT1 THEN 000 OR 100
100: 0.016871 (1644,3,127,2)

IF m_pentose-phosphate_pathway_n23-MET4 AND m_amino-acid_transport_n18-MET4 THEN 000 OR 100
100: 0.016871 (1644,3,127,2)

IF SFF'-HIR3 AND m_other_signal-transduction_activities_n8-RAP1 THEN 000 OR 100
100: 0.016871 (1644,3,127,2)

IF m_ion_transporters_n4-GAT1 AND PHO4-CBF1 THEN 000 OR 100
100: 0.016871 (1644,3,127,2)

IF m_ion_transporters_n4-INO4 AND m_purine_and_pyrimidine_transporters_n17-GAT1 THEN 000 OR 100
100: 0.016871 (1644,3,127,2)

IF m_ion_transporters_n4-GAT1 AND PHO4-INO4 THEN 000 OR 100
100: 0.016871 (1644,3,127,2)

IF m_phosphate_transport_n18-INO4 AND PHO4-CBF1 THEN 000 OR 100
100: 0.016871 (1644,3,127,2)

IF m_phosphate_transport_n18-TOS8 AND PHO4-INO4 THEN 000 OR 100
100: 0.016871 (1644,3,127,2)

IF m_phosphate_transport_n18-INO4 AND PHO4-INO4 THEN 000 OR 100
100: 0.016871 (1644,3,127,2)

IF m_phosphate_transport_n18-TOS8 AND PHO4-CBF1 THEN 000 OR 100
100: 0.016871 (1644,3,127,2)

IF m_biogenesis_of_chromosome_structure_n9-RAP1 AND RPN4-REB1 THEN 000 OR 100
100: 0.016871 (1644,3,127,2)

IF SFF'-FKH1 AND SFF-FKH2 AND m_anion_transporters_n15-INO4 THEN 000 OR 100
100: 0.016871 (1644,3,127,2)

IF SFF'-FKH2 AND SWI5-INO4 AND m_anion_transporters_n15-INO4 THEN 000 OR 100
100: 0.016871 (1644,3,127,2)

IF SFF-FKH2 AND SWI5-INO4 AND m_anion_transporters_n15-INO4 THEN 000 OR 100
100: 0.016871 (1644,3,127,2)

IF SFF-FKH1 AND SFF-FKH2 AND m_anion_transporters_n15-INO4 THEN 000 OR 100
100: 0.016871 (1644,3,127,2)

IF SFF'-FKH2 AND SFF-FKH1 AND m_anion_transporters_n15-INO4 THEN 000 OR 100
100: 0.016871 (1644,3,127,2)

IF SFF'-FKH1 AND SFF'-FKH2 AND m_anion_transporters_n15-INO4 THEN 000 OR 100
100: 0.016871 (1644,3,127,2)

IF m_ion_transporters_n4-GAT1 AND m_phosphate_transport_n18-TOS8 THEN 000 OR 100
100: 0.016871 (1644,3,127,2)

IF m_nitrogen_and_sulphur_metabolism_n17-XBP1 AND SFF-FKH2 THEN 100 OR 110
100: 0.016871 (1644,3,127,2)

IF SWI5-INO4 AND PHO-INO4 AND m_deoxyribonucleotide_metabolism_n10-INO4 THEN 000 OR 100

100: 0.016871 (1644,3,127,2)

IF m_biogenesis_of_chromosome_structure_n9-RAP1 AND RPN4-RPN4 THEN 000 OR 100
100: 0.016871 (1644,3,127,2)

IF m_MERE11-SUT1 AND m_glyoxylate_cycle_n11-UME6 THEN 010 OR 100
100: 0.016871 (1644,3,127,2)

IF m_organization_of_plasma_membrane_n17-INO4 AND m_purine_and_pyrimidine_transporters_n17-INO4 THEN 000 OR 100
100: 0.016871 (1644,3,127,2)

IF m_anion_transporters_n16-INO4 AND PHO-INO4 THEN 000 OR 100
100: 0.016871 (1644,3,127,2)

IF PHO-INO4 AND m_pentose-phosphate_pathway_n23-MET4 THEN 000 OR 100
100: 0.016871 (1644,3,127,2)

IF m_anion_transporters_n16-INO4 AND PHO4-INO4 THEN 000 OR 100
100: 0.016871 (1644,3,127,2)

IF m_RPE17-PDR1 AND m_RPE17-SFP1 THEN 000 OR 100
100: 0.016871 (1644,3,127,2)

IF m_peroxisomal_organization_n8-YAP1 AND m_ionic_homeostasis_n6-YAP1 THEN 000 OR 100
100: 0.016871 (1644,3,127,2)

IF mRRPE-ABF1 AND m_cell_death_n16-INO4 THEN 000 OR 100
100: 0.016871 (1644,3,127,2)

IF MCM1'-SWI4 AND m_RRSE3-SWI4 AND SCB-SWI4 THEN 010 OR 100
100: 0.016871 (1644,3,127,2)

IF SFF-FKH2 AND m_pentose-phosphate_pathway_n5-MCM1 THEN 100 OR 111
100: 0.016871 (1644,3,127,2)

IF m_phosphate_transport_n18-XBP1 AND PHO4-INO4 THEN 000 OR 100
100: 0.016871 (1644,3,127,2)

IF SFF'-FKH2 AND m_pentose-phosphate_pathway_n5-MCM1 THEN 100 OR 111
100: 0.016871 (1644,3,127,2)

IF m_ion_transporters_n4-GAT1 AND m_phosphate_transport_n18-XBP1 THEN 000 OR 100
100: 0.016871 (1644,3,127,2)

IF m_phosphate_transport_n18-XBP1 AND m_purine_and_pyrimidine_transporters_n17-GAT1 THEN 000 OR 100
100: 0.016871 (1644,3,127,2)

IF m_anion_transporters_n17-INO4 AND PHO-INO4 THEN 000 OR 100
100: 0.016871 (1644,3,127,2)

IF SWI5-INO4 AND PHO-MET4 THEN 000 OR 100
100: 0.016871 (1644,3,127,2)

IF m_ion_transporters_n4-GAT1 AND m_phosphate_transport_n18-INO4 THEN 000 OR 100
100: 0.016871 (1644,3,127,2)

IF CSRE-INO4 AND RPN4-ABF1 THEN 000 OR 100
100: 0.016871 (1644,3,127,2)

IF m_drug_transporters_n10-MET4 AND m_deoxyribonucleotide_metabolism_n10-INO4 THEN 000 OR 100
100: 0.016871 (1644,3,127,2)

IF m_pheromone_response_generation_n7-SIG1 THEN 000 OR 100
100: 0.016871 (1644,3,127,2)

IF SFF'-FKH2 AND m_metal_ion_transporters_n10-SKN7 THEN 100 OR 110
100: 0.016871 (1644,3,127,2)

IF SFF-FKH2 AND m_metal_ion_transporters_n10-SKN7 THEN 100 OR 110
100: 0.016871 (1644,3,127,2)

IF m_phosphate_transport_n18-XBP1 AND PHO4-CBF1 THEN 000 OR 100
100: 0.016871 (1644,3,127,2)

IF m_phosphate_transport_n18-TOS8 AND m_purine_and_pyrimidine_transporters_n17-GAT1 THEN 000 OR 100
100: 0.016871 (1644,3,127,2)

IF m_phosphate_transport_n18-GAT1 AND m_anion_transporters_n10-INO4 THEN 000 OR 100
100: 0.016871 (1644,3,127,2)

IF m_biogenesis_of_chromosome_structure_n9-REB1 AND RPN4-REB1 THEN 000 OR 100
100: 0.016871 (1644,3,127,2)

IF m_biogenesis_of_chromosome_structure_n9-REB1 AND RPN4-RPN4 THEN 000 OR 100
100: 0.016871 (1644,3,127,2)

IF m_allantoin_and_allantoate_transporters_n18-FKH2 AND SFF-FKH1 THEN 100 OR 110
100: 0.016871 (1644,3,127,2)

IF SFF'-FKH1 AND m_allantoin_and_allantoate_transporters_n18-FKH2 AND SFF-FKH2 THEN 100 OR 110
100: 0.016871 (1644,3,127,2)

IF m_other_transcription_activities_n5-SWI6 AND m_amino-acid_transport_n14-SWI6 THEN 000 OR 100
100: 0.016871 (1644,3,127,2)

IF SFF'-MCM1 AND SFF-FKH2 AND m_pentose-phosphate_pathway_n5-MCM1 THEN 100 OR 111
100: 0.016871 (1644,3,127,2)

IF m_LFTE17-SKN7 AND m_meiosis_n3-UME6 THEN 000 OR 100
100: 0.016871 (1644,3,127,2)

IF m_phosphate_transport_n18-GAT1 AND PHO4-CBF1 THEN 000 OR 100
100: 0.016871 (1644,3,127,2)

IF SFF'-FKH2 AND SFF'-MCM1 AND m_pentose-phosphate_pathway_n5-MCM1 THEN 100 OR 111
100: 0.016871 (1644,3,127,2)

IF SWI5-RIM101 AND m_fermentation_n18-GLN3 THEN 000 OR 100
100: 0.016871 (1644,3,127,2)

IF SFF'-FKH1 AND m_pentose-phosphate_pathway_n5-SWI4 THEN 100 OR 111
100: 0.016871 (1644,3,127,2)

IF mRRPE-LEU3 AND m_RPE52-SPT2 THEN 000 OR 100
100: 0.016871 (1644,3,127,2)

IF m_ion_transporters_n4-GAT1 AND m_purine_and_pyrimidine_transporters_n17-INO4 THEN 000 OR 100
100: 0.016871 (1644,3,127,2)

IF ALPHA1'-RIM101 AND m_fermentation_n18-GLN3 THEN 000 OR 100
100: 0.016871 (1644,3,127,2)

IF ATRepeat-HAP1 AND ATRepeat-UME6 THEN 000 OR 100
100: 0.016871 (1644,3,127,2)

IF m_g-proteins_n11-INO4 AND m_pentose-phosphate_pathway_n5-SWI4 THEN 000 OR 100
100: 0.016871 (1644,3,127,2)

IF m_g-proteins_n12-SKN7 AND m_lipid_and_fatty-acid_transport_n11-SKN7 AND m_deoxyribonucleotide_metabolism_n10-SKN7 THEN 000 OR 100
100: 0.016871 (1644,3,127,2)

IF m_g-proteins_n11-INO4 AND SWI5-INO4 AND m_organization_of_chromosome_structure_n12-INO4 THEN 001 OR 100
100: 0.016871 (1644,3,127,2)

IF m_regulation_of_lipid_fatty-acid_and_isoprenoid_biosynthesis_n8.scn-FKH1 AND m_pentose-phosphate_pathway_n23-MET4 THEN 100 OR 110
100: 0.016871 (1644,3,127,2)

IF ATRepeat-UME6 AND mPROTEOL18(m_proteolysis_n18)-UME6 THEN 010 OR 100

100: 0.016871 (1644,3,127,2)

IF m_purine_and_pyrimidine_transporters_n17-GAT1 AND m_anion_transporters_n10-INO4 THEN 000 OR 100
100: 0.016871 (1644,3,127,2)

IF m_anion_transporters_n17-INO4 AND PHO-INO4 AND m_pentose-phosphate_pathway_n23-INO4 THEN 000 OR 100
100: 0.016871 (1644,3,127,2)

IF m_anion_transporters_n17-INO4 AND SWI5-INO4 AND PHO-INO4 THEN 000 OR 100
100: 0.016871 (1644,3,127,2)

IF m_allantoin_and_allantoate_transporters_n18-FKH2 AND SFF-FKH2 AND m_other_morphogenetic_activities_n7-MCM1 THEN 100 OR 110
100: 0.016871 (1644,3,127,2)

IF SFF-HIR1 AND CCA-MET4 THEN 000 OR 100
100: 0.016871 (1644,3,127,2)

IF SFF'-HIR1 AND CCA-MET4 THEN 000 OR 100
100: 0.016871 (1644,3,127,2)

IF m_g-proteins_n11-UME6 AND SWI5-SWI6 THEN 010 OR 100
100: 0.016871 (1644,3,127,2)

IF MCM1'-MCM1 AND m_allantoin_and_allantoate_transporters_n18-FKH2 AND SFF-FKH2 THEN 100 OR 110
100: 0.016871 (1644,3,127,2)

IF mRRPE-SPT2 AND m_RPE52-SPT2 THEN 000 OR 100
100: 0.016871 (1644,3,127,2)

IF m_metabolism_of_cyclic_and_unusual_nucleotides_n5-YAP1 AND SFF-FKH2 THEN 000 OR 100
100: 0.016871 (1644,3,127,2)

IF SFF'-HIR3 AND CCA-MET4 THEN 000 OR 100
100: 0.016871 (1644,3,127,2)

IF m_ion_transporters_n4-INO4 AND m_phosphate_transport_n18-PUT3 THEN 000 OR 100
100: 0.016871 (1644,3,127,2)

IF SFF'-HIR3 AND m_organization_of_chromosome_structure_n12-HIR2 THEN 100 OR 101
100: 0.016871 (1644,3,127,2)

IF SFF'-MCM1 AND m_allantoin_and_allantoate_transporters_n18-FKH2 AND SFF-FKH2 THEN 100 OR 110
100: 0.016871 (1644,3,127,2)

IF m_anion_transporters_n22-RAP1 AND MIG1-ROX1 THEN 100 OR 110
100: 0.016871 (1644,3,127,2)

IF PHO-INO4 AND m_pentose-phosphate_pathway_n23-INO4 AND m_deoxyribonucleotide_metabolism_n10-INO4 THEN 000 OR 100
100: 0.016871 (1644,3,127,2)

IF m_g-proteins_n11-INO4 AND m_anion_transporters_n15-GAT1 THEN 000 OR 100
100: 0.016871 (1644,3,127,2)

IF m_g-proteins_n11-INO4 AND m_amino-acid_degradation_n27-INO4 THEN 000 OR 100
100: 0.016871 (1644,3,127,2)

IF m_anion_transporters_n17-INO4 AND m_amino-acid_degradation_n27-INO4 THEN 000 OR 100
100: 0.016871 (1644,3,127,2)

IF m_osmosensing_n6-SWI4 AND m_anion_transporters_n15-INO4 THEN 000 OR 100
100: 0.016871 (1644,3,127,2)

IF m_MERE17-MTH1 AND m_MERE16-MTH1 THEN 000 OR 100
100: 0.016871 (1644,3,127,2)

IF PHO-INO4 AND PHO-MET4 THEN 000 OR 100
100: 0.016871 (1644,3,127,2)

IF MCM1'-MCM1 AND SWI5-INO4 THEN 100 OR 110
100: 0.016871 (1644,3,127,2)

IF m_phosphate_transport_n18-GAT1 AND m_purine_and_pyrimidine_transporters_n17-INO4 THEN 000 OR 100
100: 0.016871 (1644,3,127,2)

IF PHO-MET4 AND m_deoxyribonucleotide_metabolism_n10-INO4 THEN 000 OR 100
100: 0.016871 (1644,3,127,2)

IF m_phosphate_transport_n18-GAT1 AND PHO4-INO4 THEN 000 OR 100
100: 0.016871 (1644,3,127,2)

IF m_ion_transporters_n4-INO4 AND m_phosphate_transport_n18-GAT1 THEN 000 OR 100
100: 0.016871 (1644,3,127,2)

IF m_g-proteins_n12-SKN7 AND m_lipid_and_fatty-acid_transport_n11-SKN7 THEN 000 OR 010 OR 100 OR 110 OR 111
111: 0.017346 (1644,18,19,2)

IF m_purine_and_pyrimidine_transporters_n17-INO4 THEN 000 OR 100
100: 0.018494 (1644,14,127,4)

IF m_nutritional_response_pathway_n7-SWI6 THEN 000 OR 010 OR 101 OR 110 OR 111
110: 0.018645 (1644,23,115,5)

IF mPROTEOL18(m_proteolysis_n18)-MBP1 THEN 000 OR 001 OR 010 OR 100 OR 110 OR 111
110: 0.018645 (1644,23,115,5)

IF ALPHA2-HAP5 THEN 000 OR 010 OR 100
100: 0.018906 (1644,8,127,3)

IF m_anion_transporters_n17-INO4 AND SWI5-INO4 THEN 000 OR 001 OR 100 OR 110
100: 0.018906 (1644,8,127,3)

IF m_pheromone_response_generation_n4-PUT3 THEN 000 OR 010 OR 100 OR 110
100: 0.018906 (1644,8,127,3)

IF m_allantoin_and_allantoate_transporters_n7-ACE2 THEN 000 OR 010 OR 100 OR 110 OR 111
111: 0.019254 (1644,19,19,2)

IF SFF'-FKH2 AND SWI5-SWI6 THEN 000 OR 001 OR 010 OR 100 OR 110 OR 111
111: 0.019254 (1644,19,19,2)

IF SFF'-HIR3 AND m_other_morphogenetic_activities_n7-RCS1 AND RAP1-YAP5 THEN 000 OR 010
010: 0.020402 (1644,3,140,2)

IF SFF'-HIR1 AND RAP1-FHL1 AND RAP1-GAT3 THEN 000 OR 010
010: 0.020402 (1644,3,140,2)

IF SFF'-HIR3 AND m_other_morphogenetic_activities_n7-RCS1 AND ALPHA1-DAT1 THEN 000 OR 010
010: 0.020402 (1644,3,140,2)

IF SFF'-HIR3 AND m_other_morphogenetic_activities_n7-RCS1 AND RAP1-RGM1 THEN 000 OR 010
010: 0.020402 (1644,3,140,2)

IF m_g-proteins_n12-INO4 AND m_tricarboxylic-acid_pathway_n9-SKN7 THEN 000 OR 010
010: 0.020402 (1644,3,140,2)

IF SFF'-HIR3 AND RAP1-FHL1 AND RAP1-HAP4 THEN 000 OR 010
010: 0.020402 (1644,3,140,2)

IF m_glycolysis_and_gluconeogenesis_n4-INO4 AND m_tricarboxylic-acid_pathway_n9-SKN7 THEN 000 OR 010
010: 0.020402 (1644,3,140,2)

IF SFF-FKH1 AND m_chromatin_modification_n21-HSF1 THEN 010 OR 100
010: 0.020402 (1644,3,140,2)

IF SFF'-FKH1 AND m_chromatin_modification_n21-HSF1 THEN 010 OR 100
010: 0.020402 (1644,3,140,2)

IF m_anion_transporters_n15-INO4 AND m_tricarboxylic-acid_pathway_n9-SKN7 THEN 000 OR 010
010: 0.020402 (1644,3,140,2)

IF SWI5-INO4 AND m_tricarboxylic-acid_pathway_n9-SKN7 THEN 000 OR 010
010: 0.020402 (1644,3,140,2)

IF SFF'-HIR3 AND m_other_morphogenetic_activities_n7-RCS1 AND RAP1-RAP1 THEN 000 OR 010
010: 0.020402 (1644,3,140,2)

IF SFF'-HIR3 AND RAP1-HMS1 THEN 000 OR 010
010: 0.020402 (1644,3,140,2)

IF m_other_morphogenetic_activities_n7-RCS1 AND RAP1-GAT3 THEN 000 OR 010
010: 0.020402 (1644,3,140,2)

IF m_other_morphogenetic_activities_n7-RCS1 AND RAP1-YAP5 THEN 000 OR 010
010: 0.020402 (1644,3,140,2)

IF RAP1-RGM1 AND ALPHA1-DAT1 THEN 000 OR 010
010: 0.020402 (1644,3,140,2)

IF m_other_morphogenetic_activities_n7-RCS1 AND ALPHA1-DAT1 THEN 000 OR 010
010: 0.020402 (1644,3,140,2)

IF m_other_morphogenetic_activities_n7-RCS1 AND RAP1-RGM1 THEN 000 OR 010
010: 0.020402 (1644,3,140,2)

IF m_other_morphogenetic_activities_n7-RCS1 AND RAP1-PDR1 THEN 000 OR 010
010: 0.020402 (1644,3,140,2)

IF m_g-proteins_n12-NRG1 AND SWI5-SKN7 AND SCB-SWI4 THEN 000 OR 010
010: 0.020402 (1644,3,140,2)

IF m_g-proteins_n12-NRG1 AND SWI5-SWI6 AND SCB-SWI4 THEN 000 OR 010
010: 0.020402 (1644,3,140,2)

IF m_other_morphogenetic_activities_n7-RCS1 AND ALPHA1-RGM1 THEN 000 OR 010
010: 0.020402 (1644,3,140,2)

IF m_other_morphogenetic_activities_n7-RCS1 AND ALPHA1-GAT3 THEN 000 OR 010
010: 0.020402 (1644,3,140,2)

IF RAP1-ARO80 AND RAP1-HAP4 AND RAP1-PDR1 THEN 000 OR 010
010: 0.020402 (1644,3,140,2)

IF m_g-proteins_n12-IME4 AND SFF-MIG1 THEN 000 OR 010
010: 0.020402 (1644,3,140,2)

IF SFF-ARG80 AND m_other_morphogenetic_activities_n7-ARR1 THEN 000 OR 010
010: 0.020402 (1644,3,140,2)

IF ABF1-ABF1 AND m_other_nutritional-response_activities_n10-INO2 THEN 000 OR 010
010: 0.020402 (1644,3,140,2)

IF ABF1-ABF1 AND m_other_nutritional-response_activities_n10-INO4 THEN 000 OR 010
010: 0.020402 (1644,3,140,2)

IF m_lipid_and_fatty-acid_transport_n11-GAT1 AND m_lipid_and_fatty-acid_binding_n14-GAT1 THEN 000 OR 010
010: 0.020402 (1644,3,140,2)

IF m_amino-acid_transport_n13-MTH1 AND m_stress_response_n17-MTH1 THEN 000 OR 010
010: 0.020402 (1644,3,140,2)

IF m_other_nutritional-response_activities_n10-INO4 AND m_ion_transporters_n14-INO4 THEN 000 OR 010
010: 0.020402 (1644,3,140,2)

IF RAP1-FHL1 AND ALPHA1-DAT1 AND ALPHA1-RGM1 THEN 000 OR 010
010: 0.020402 (1644,3,140,2)

IF m_other_morphogenetic_activities_n7-RCS1 AND RAP1-FHL1 AND RAP1-RAP1 AND RAP1-RGM1 THEN 000 OR 010
010: 0.020402 (1644,3,140,2)

IF SFF'-HIR3 AND m_other_morphogenetic_activities_n7-RCS1 AND RAP1-FHL1 THEN 000 OR 010

010: 0.020402 (1644,3,140,2)

IF m_other_morphogenetic_activities_n7-RCS1 AND RAP1-GAT3 AND ALPHA1-GAT3 THEN 000 OR 010
010: 0.020402 (1644,3,140,2)

IF SFF'-HIR1 AND SFF'-HIR3 AND RAP1-ARO80 THEN 000 OR 010
010: 0.020402 (1644,3,140,2)

IF SFF'-HIR3 AND RAP1-HAP4 AND RAP1-PDR1 THEN 000 OR 010
010: 0.020402 (1644,3,140,2)

IF SFF-HIR1 AND RAP1-ARO80 AND RAP1-RAP1 THEN 000 OR 010
010: 0.020402 (1644,3,140,2)

IF SFF'-HIR1 AND RAP1-ARO80 AND RAP1-FHL1 THEN 000 OR 010
010: 0.020402 (1644,3,140,2)

IF SFF'-HIR1 AND RAP1-FHL1 AND ALPHA1-GAT3 THEN 000 OR 010
010: 0.020402 (1644,3,140,2)

IF m_osmosensing_n6-INO4 AND m_RPE57-SKN7 THEN 000 OR 010
010: 0.020402 (1644,3,140,2)

IF m_other_morphogenetic_activities_n7-RCS1 AND RAP1-DAT1 AND ALPHA1-DAT1 THEN 000 OR 010
010: 0.020402 (1644,3,140,2)

IF m_glyoxylate_cycle_n8-MTH1 AND m_regulation_of_amino-acid_metabolism_n10-SKN7 THEN 000 OR 010
010: 0.020402 (1644,3,140,2)

IF RAP1-DAT1 AND RAP1-FHL1 AND RAP1-RAP1 AND RAP1-RGM1 THEN 000 OR 010
010: 0.020402 (1644,3,140,2)

IF AFT1-PDR1 AND m_regulation_of_nitrogen_and_sulphur_utilization_n7-RAP1 THEN 000 OR 010
010: 0.020402 (1644,3,140,2)

IF SFF'-HIR1 AND SFF'-HIR3 AND RAP1-RAP1 THEN 000 OR 010
010: 0.020402 (1644,3,140,2)

IF SFF'-HIR3 AND SFF-HIR1 AND RAP1-ARO80 THEN 000 OR 010
010: 0.020402 (1644,3,140,2)

IF STE12-IXR1 AND RAP1-PDR1 THEN 000 OR 010
010: 0.020402 (1644,3,140,2)

IF AFT1-PDR1 AND RAP1-YAP5 THEN 000 OR 010
010: 0.020402 (1644,3,140,2)

IF AFT1-PDR1 AND RAP1-RGM1 THEN 000 OR 010
010: 0.020402 (1644,3,140,2)

IF m_other_morphogenetic_activities_n7-RCS1 AND RAP1-FHL1 AND RAP1-PDR1 THEN 000 OR 010
010: 0.020402 (1644,3,140,2)

IF SFF-GZF3 AND ABF1-WAR1 THEN 010 OR 100
010: 0.020402 (1644,3,140,2)

IF RAP1-ARG81 AND RAP1-FHL1 AND RAP1-RAP1 THEN 000 OR 010
010: 0.020402 (1644,3,140,2)

IF SFF'-HIR1 AND RAP1-HAP4 AND RAP1-YAP5 THEN 000 OR 010
010: 0.020402 (1644,3,140,2)

IF m_anion_transporters_n22-RAP1 AND RAP1-RAP1 AND m_nitrogen_and_sulphur_transport_n9-FHL1 THEN 000 OR 010
010: 0.020402 (1644,3,140,2)

IF SFF'-HIR3 AND RAP1-ARG81 AND RAP1-ZAP1 THEN 000 OR 010
010: 0.020402 (1644,3,140,2)

IF RAP1-DAT1 AND RAP1-RAP1 AND RAP1-RGM1 THEN 000 OR 010
010: 0.020402 (1644,3,140,2)

IF SFF'-HIR1 AND RAP1-ARO80 AND RAP1-YAP5 THEN 000 OR 010
010: 0.020402 (1644,3,140,2)

IF RAP1-ARG81 AND RAP1-DAT1 AND RAP1-RAP1 THEN 000 OR 010
010: 0.020402 (1644,3,140,2)

IF RAP1-PDR1 AND ALPHA1-DAT1 AND ALPHA1-RGM1 THEN 000 OR 010
010: 0.020402 (1644,3,140,2)

IF SFF-HIR1 AND RAP1-FHL1 AND RAP1-HAP4 THEN 000 OR 010
010: 0.020402 (1644,3,140,2)

IF m_other_morphogenetic_activities_n7-RCS1 AND RAP1-PDR1 AND RAP1-RGM1 THEN 000 OR 010
010: 0.020402 (1644,3,140,2)

IF RAP1-DAT1 AND ALPHA1-DAT1 AND ALPHA1-RGM1 THEN 000 OR 010
010: 0.020402 (1644,3,140,2)

IF RAP1-ARO80 AND RAP1-FHL1 AND RAP1-HAP4 THEN 000 OR 010
010: 0.020402 (1644,3,140,2)

IF RAP1-DAT1 AND RAP1-RAP1 AND ALPHA1-RGM1 THEN 000 OR 010
010: 0.020402 (1644,3,140,2)

IF SFF'-HIR1 AND SFF'-HIR3 AND RAP1-PDR1 THEN 000 OR 010
010: 0.020402 (1644,3,140,2)

IF SFF'-HIR1 AND RAP1-PDR1 AND RAP1-YAP5 THEN 000 OR 010
010: 0.020402 (1644,3,140,2)

IF SFF'-HIR1 AND RAP1-FHL1 AND RAP1-PDR1 THEN 000 OR 010
010: 0.020402 (1644,3,140,2)

IF SFF-HIR1 AND RAP1-FHL1 AND RAP1-PDR1 THEN 000 OR 010
010: 0.020402 (1644,3,140,2)

IF SFF-HIR1 AND RAP1-PDR1 AND RAP1-RAP1 THEN 000 OR 010
010: 0.020402 (1644,3,140,2)

IF m_anion_transporters_n22-RAP1 AND RAP1-FHL1 AND m_nitrogen_and_sulphur_transport_n9-FHL1 THEN 000 OR 010
010: 0.020402 (1644,3,140,2)

IF RAP1-RGM1 AND ALPHA1-DAT1 AND ALPHA1-RGM1 THEN 000 OR 010
010: 0.020402 (1644,3,140,2)

IF SFF'-HIR1 AND RAP1-RAP1 AND ALPHA1-GAT3 THEN 000 OR 010
010: 0.020402 (1644,3,140,2)

IF SFF'-HIR1 AND RAP1-YAP5 AND ALPHA1-GAT3 THEN 000 OR 010
010: 0.020402 (1644,3,140,2)

IF m_other_morphogenetic_activities_n7-RCS1 AND RAP1-FHL1 AND ALPHA1-GAT3 THEN 000 OR 010
010: 0.020402 (1644,3,140,2)

IF SFF-HIR1 AND RAP1-PDR1 AND RAP1-YAP5 THEN 000 OR 010
010: 0.020402 (1644,3,140,2)

IF SFF'-HIR1 AND m_other_morphogenetic_activities_n7-ARR1 AND STE12-IXR1 THEN 000 OR 010
010: 0.020402 (1644,3,140,2)

IF RAP1-ARO80 AND RAP1-HAP4 AND RAP1-RAP1 THEN 000 OR 010
010: 0.020402 (1644,3,140,2)

IF RAP1-HAP4 AND RAP1-YAP5 AND RAP1-ZAP1 THEN 000 OR 010
010: 0.020402 (1644,3,140,2)

IF RAP1-FHL1 AND RAP1-HAP4 AND RAP1-ZAP1 THEN 000 OR 010
010: 0.020402 (1644,3,140,2)

IF SFF'-HIR3 AND ALPHA1-DAT1 AND ALPHA1-RGM1 THEN 000 OR 010
010: 0.020402 (1644,3,140,2)

IF m_utilization_of_vitamins_cofactors_and_prosthetic_groups_n7-STP2 THEN 000 OR 010
010: 0.020402 (1644,3,140,2)

IF RAP1-FHL1 AND RAP1-GAT3 AND RAP1-HAP4 THEN 000 OR 010
010: 0.020402 (1644,3,140,2)

IF SFF-HIR1 AND RAP1-HAP4 AND RAP1-RAP1 THEN 000 OR 010
010: 0.020402 (1644,3,140,2)

IF RAP1-ARG81 AND RAP1-HAP4 AND RAP1-RAP1 THEN 000 OR 010
010: 0.020402 (1644,3,140,2)

IF RAP1-DAT1 AND RAP1-RAP1 AND RAP1-ZAP1 THEN 000 OR 010
010: 0.020402 (1644,3,140,2)

IF SFF-HIR1 AND RAP1-ARO80 AND RAP1-YAP5 THEN 000 OR 010
010: 0.020402 (1644,3,140,2)

IF SFF-HIR1 AND RAP1-RAP1 AND ALPHA1-GAT3 THEN 000 OR 010
010: 0.020402 (1644,3,140,2)

IF m_lipid_and_fatty-acid_binding_n15-FHL1 AND RAP1-YAP5 THEN 000 OR 010
010: 0.020402 (1644,3,140,2)

IF m_lipid_and_fatty-acid_binding_n15-FHL1 AND RAP1-FHL1 THEN 000 OR 010
010: 0.020402 (1644,3,140,2)

IF m_glyoxylate_cycle_n8-MTH1 AND m_RPE57-SKN7 THEN 000 OR 010
010: 0.020402 (1644,3,140,2)

IF m_anion_transporters_n22-RAP1 AND SFF-HIR1 THEN 010 OR 100
010: 0.020402 (1644,3,140,2)

IF RAP1-RAP1 AND m_other_proteolytic_degradation_n5-PDR1 THEN 000 OR 010
010: 0.020402 (1644,3,140,2)

IF RAP1-SMP1 AND m_other_proteolytic_degradation_n5-PDR1 THEN 000 OR 010
010: 0.020402 (1644,3,140,2)

IF RAP1-FHL1 AND m_other_proteolytic_degradation_n5-PDR1 THEN 000 OR 010
010: 0.020402 (1644,3,140,2)

IF SFF'-HIR3 AND m_other_proteolytic_degradation_n5-PDR1 THEN 000 OR 010
010: 0.020402 (1644,3,140,2)

IF RAP1-PDR1 AND m_other_proteolytic_degradation_n5-PDR1 THEN 000 OR 010
010: 0.020402 (1644,3,140,2)

IF SFF-FKH1 AND m_amino-acid_transport_n18-SKN7 THEN 010 OR 100
010: 0.020402 (1644,3,140,2)

IF SFF'-RTG3 AND MCM1-YOX1 THEN 010 OR 110
010: 0.020402 (1644,3,140,2)

IF SFF'-HIR3 AND SFF-HIR1 AND RAP1-RAP1 THEN 000 OR 010
010: 0.020402 (1644,3,140,2)

IF SFF'-HIR3 AND SFF-HIR1 AND RAP1-FHL1 THEN 000 OR 010
010: 0.020402 (1644,3,140,2)

IF SFF-FKH1 AND SCB-AZF1 THEN 010 OR 111
010: 0.020402 (1644,3,140,2)

IF AFT1-RAP1 AND RAP1-PDR1 THEN 000 OR 010
010: 0.020402 (1644,3,140,2)

IF ECB-YOX1 AND m_other_morphogenetic_activities_n7-GCR2 THEN 010 OR 110

010: 0.020402 (1644,3,140,2)

IF MCM1-YOX1 AND m_other_morphogenetic_activities_n7-GCR2 THEN 010 OR 110
010: 0.020402 (1644,3,140,2)

IF RAP1-ARG81 AND RAP1-PDR1 THEN 000 OR 010
010: 0.020402 (1644,3,140,2)

IF RAP1-HAP4 AND RAP1-ZAP1 THEN 000 OR 010
010: 0.020402 (1644,3,140,2)

IF m_lipid_and_fatty-acid_binding_n15-FHL1 AND RAP1-ZAP1 THEN 000 OR 010
010: 0.020402 (1644,3,140,2)

IF STE12-IXR1 AND m_lipid_and_fatty-acid_binding_n13-PDR1 THEN 000 OR 010
010: 0.020402 (1644,3,140,2)

IF RAP1-ZAP1 AND m_lipid_and_fatty-acid_binding_n13-PDR1 THEN 000 OR 010
010: 0.020402 (1644,3,140,2)

IF m_rRSE10-PDR1 AND ALPHA1-RGM1 THEN 000 OR 010
010: 0.020402 (1644,3,140,2)

IF m_other_morphogenetic_activities_n7-ARR1 AND ALPHA1-GAT3 THEN 000 OR 010
010: 0.020402 (1644,3,140,2)

IF m_lipid_and_fatty-acid_binding_n15-FHL1 AND m_nitrogen_and_sulphur_transport_n9-FHL1 THEN 000 OR 010
010: 0.020402 (1644,3,140,2)

IF SFF'-HIR3 AND SFF-HIR1 AND RAP1-YAP5 THEN 000 OR 010
010: 0.020402 (1644,3,140,2)

IF mRRPE-ABF1 AND ABF1-ABF1 AND ALPHA1'-RIM101 THEN 000 OR 010
010: 0.020402 (1644,3,140,2)

IF ALPHA1'-RIM101 AND ALPHA1'-STB4 AND ALPHA1'-YER051w THEN 000 OR 010
010: 0.020402 (1644,3,140,2)

IF SFF'-HIR1 AND RAP1-PDR1 THEN 000 OR 010
010: 0.020402 (1644,3,140,2)

IF m_lipid_and_fatty-acid_binding_n15-FHL1 AND RAP1-RAP1 THEN 000 OR 010
010: 0.020402 (1644,3,140,2)

IF SFF'-HIR1 AND RAP1-ARO80 THEN 000 OR 010
010: 0.020402 (1644,3,140,2)

IF m_regulation_of_amino-acid_metabolism_n10-SKN7 AND m_RPE57-SKN7 THEN 000 OR 010
010: 0.020402 (1644,3,140,2)

IF SFF-HIR1 AND STE12-IXR1 THEN 000 OR 010
010: 0.020402 (1644,3,140,2)

IF SFF'-RTG3 AND m_g-proteins_n11-HAL9 AND SFF-ARG80 THEN 010 OR 110
010: 0.020402 (1644,3,140,2)

IF SFF'-HIR1 AND RAP1-HAP4 THEN 000 OR 010
010: 0.020402 (1644,3,140,2)

IF AFT1-RAP1 AND RAP1-GAT3 AND RAP1-RGM1 THEN 000 OR 010
010: 0.020402 (1644,3,140,2)

IF ECB-YOX1 AND SFF-ARG80 THEN 010 OR 110
010: 0.020402 (1644,3,140,2)

IF m_g-proteins_n11-INO4 AND m_phosphate_transport_n5-INO4 THEN 010 OR 110
010: 0.020402 (1644,3,140,2)

IF m_phosphate_transport_n5-INO4 AND m_other_energy_generation_activities_n22-INO4 THEN 010 OR 110
010: 0.020402 (1644,3,140,2)

IF mPROTEOL18(m_proteolysis_n18)-MBP1 AND m_other_transport_facilitators_n5-SWI6 THEN 000 OR 010
010: 0.020402 (1644,3,140,2)

IF m_other_transport_facilitators_n5-SWI6 AND m_cell_death_n22-SWI4 THEN 000 OR 010
010: 0.020402 (1644,3,140,2)

IF RAP1-ARG81 AND RAP1-HAP4 THEN 000 OR 010
010: 0.020402 (1644,3,140,2)

IF MCM1'-SWI4 AND m_amino-acid_transporters_n11-UME6 THEN 000 OR 010
010: 0.020402 (1644,3,140,2)

IF m_amino-acid_transporters_n11-SWI4 AND m_amino-acid_transporters_n11-UME6 THEN 000 OR 010
010: 0.020402 (1644,3,140,2)

IF RAP1-FHL1 AND m_lipid_and_fatty-acid_binding_n13-PDR1 THEN 000 OR 010
010: 0.020402 (1644,3,140,2)

IF m_lipid_and_fatty-acid_binding_n15-FHL1 AND RAP1-GAT3 THEN 000 OR 010
010: 0.020402 (1644,3,140,2)

IF SFF-HIR1 AND RAP1-ARO80 THEN 000 OR 010
010: 0.020402 (1644,3,140,2)

IF SFF'-HIR1 AND RAP1-ZAP1 THEN 000 OR 010
010: 0.020402 (1644,3,140,2)

IF SFF'-HIR1 AND RAP1-ARG81 THEN 000 OR 010
010: 0.020402 (1644,3,140,2)

IF ECB-YOX1 AND SFF-RTG3 THEN 010 OR 110
010: 0.020402 (1644,3,140,2)

IF AFT1-RAP1 AND RAP1-RGM1 AND ALPHA1-RGM1 THEN 000 OR 010
010: 0.020402 (1644,3,140,2)

IF SFF-HIR1 AND RAP1-ZAP1 THEN 000 OR 010
010: 0.020402 (1644,3,140,2)

IF m_rRSE10-PDR1 AND ALPHA1-GAT3 THEN 000 OR 010
010: 0.020402 (1644,3,140,2)

IF SFF'-HIR1 AND SFF'-HIR3 AND RAP1-FHL1 THEN 000 OR 010
010: 0.020402 (1644,3,140,2)

IF m_g-proteins_n11-HAL9 AND SFF-RTG3 THEN 010 OR 110
010: 0.020402 (1644,3,140,2)

IF AFT1-RAP1 AND m_rRSE10-PDR1 THEN 000 OR 010
010: 0.020402 (1644,3,140,2)

IF SFF'-HIR1 AND m_anion_transporters_n22-RAP1 THEN 010 OR 100
010: 0.020402 (1644,3,140,2)

IF m_osmosensing_n6-INO4 AND m_lipid_and_fatty-acid_transport_n7-SKN7 THEN 000 OR 010
010: 0.020402 (1644,3,140,2)

IF RAP1-PDR1 AND m_lipid_and_fatty-acid_binding_n13-PDR1 THEN 000 OR 010
010: 0.020402 (1644,3,140,2)

IF m_lipid_and_fatty-acid_binding_n15-FHL1 AND RAP1-PDR1 THEN 000 OR 010
010: 0.020402 (1644,3,140,2)

IF STE12-IXR1 AND RAP1-FHL1 THEN 000 OR 010
010: 0.020402 (1644,3,140,2)

IF m_g-proteins_n11-INO4 AND SCB-SWI4 THEN 000 OR 010
010: 0.020402 (1644,3,140,2)

IF m_g-proteins_n11-INO4 AND mPROTEOL18(m_proteolysis_n18)-SWI6 THEN 010 OR 100
010: 0.020402 (1644,3,140,2)

IF m_g-proteins_n11-INO4 AND mPROTEOL18(m_proteolysis_n18)-YAP6 THEN 010 OR 100
010: 0.020402 (1644,3,140,2)

IF SFF-MIG1 AND m_drug_transporters_n10-MET4 THEN 010 OR 100
010: 0.020402 (1644,3,140,2)

IF m_lipid_and_fatty-acid_transport_n11-GAT1 AND m_other_mrna-transcription_activities_n20-INO4 AND m_glyoxylate_cycle_n11-GAT1 THEN 000 OR 010
010: 0.020402 (1644,3,140,2)

IF m_other_mrna-transcription_activities_n20-SKN7 AND m_tricarboxylic-acid_pathway_n9-SKN7 THEN 000 OR 010
010: 0.020402 (1644,3,140,2)

IF m_anion_transporters_n4-MTH1 AND m_rRSE10-MTH1 AND m_deoxyribonucleotide_metabolism_n23-MTH1 THEN 000 OR 010
010: 0.020402 (1644,3,140,2)

IF m_other_mrna-transcription_activities_n20-SKN7 AND m_tricarboxylic-acid_pathway_n9-GAT1 THEN 000 OR 010
010: 0.020402 (1644,3,140,2)

IF SFF'-RTG3 AND m_other_morphogenetic_activities_n7-HOG1 THEN 000 OR 010
010: 0.020402 (1644,3,140,2)

IF m_other_mrna-transcription_activities_n20-INO4 AND m_tricarboxylic-acid_pathway_n9-GAT1 THEN 000 OR 010
010: 0.020402 (1644,3,140,2)

IF m_other_mrna-transcription_activities_n20-INO4 AND m_tricarboxylic-acid_pathway_n9-SKN7 THEN 000 OR 010
010: 0.020402 (1644,3,140,2)

IF SFF-RTG3 AND m_other_morphogenetic_activities_n7-HOG1 THEN 000 OR 010
010: 0.020402 (1644,3,140,2)

IF m_g-proteins_n11-HAL9 AND ALPHA1'-YER051w THEN 000 OR 010
010: 0.020402 (1644,3,140,2)

IF mPROTEOL18(m_proteolysis_n18)-UME6 AND m_MERE11-SUT1 THEN 010 OR 100
010: 0.020402 (1644,3,140,2)

IF mPROTEOL18(m_proteolysis_n18)-MBP1 AND m_amino-acid_transporters_n11-CUP9 THEN 010 OR 111
010: 0.020402 (1644,3,140,2)

IF m_other_proteolytic_degradation_n2-MTH1 AND m_rRSE10-MTH1 THEN 000 OR 010
010: 0.020402 (1644,3,140,2)

IF m_g-proteins_n12-NRG1 AND m_amino-acid_transporters_n11-UME6 THEN 000 OR 010
010: 0.020402 (1644,3,140,2)

IF SFF-HIR1 AND RAP1-PDR1 THEN 000 OR 010
010: 0.020402 (1644,3,140,2)

IF mPROTEOL18(m_proteolysis_n18)-UME6 AND m_biosynthesis_of_vitamins_cofactors_and_prosthetic_groups_n8-MET4 THEN 010 OR 100
010: 0.020402 (1644,3,140,2)

IF MCM1'-SWI4 AND m_other_mrna-transcription_activities_n20-INO4 THEN 010 OR 100
010: 0.020402 (1644,3,140,2)

IF RAP1-RAP1 AND RAP1-ZAP1 AND ALPHA1-DAT1 THEN 000 OR 010
010: 0.020402 (1644,3,140,2)

IF ALPHA2-INO2 AND m_other_proteolytic_degradation_n8-INO2 THEN 000 OR 010
010: 0.020402 (1644,3,140,2)

IF mPROTEOL18(m_proteolysis_n18)-YAP6 AND SCB-SWI4 THEN 010 OR 111
010: 0.020402 (1644,3,140,2)

IF SFF'-HIR1 AND m_utilization_of_vitamins_cofactors_and_prosthetic_groups_n7-RPN4 THEN 000 OR 010
010: 0.020402 (1644,3,140,2)

IF m_utilization_of_vitamins_cofactors_and_prosthetic_groups_n7-RPN4 AND SFF-HIR1 THEN 000 OR 010
010: 0.020402 (1644,3,140,2)

IF SFF-MIG1 AND m_glyoxylate_cycle_n11-UME6 THEN 010 OR 100
010: 0.020402 (1644,3,140,2)

IF SFF-ARG80 AND ALPHA1'-KSS1 THEN 000 OR 010
010: 0.020402 (1644,3,140,2)

IF SFF'-HIR1 AND RAP1-GAT3 AND RAP1-YAP5 THEN 000 OR 010
010: 0.020402 (1644,3,140,2)

IF RAP1-ARG81 AND RAP1-ARO80 THEN 000 OR 010
010: 0.020402 (1644,3,140,2)

IF SFF-HIR1 AND RAP1-ARG81 THEN 000 OR 010
010: 0.020402 (1644,3,140,2)

IF RAP1-GAT3 AND RAP1-HAP4 THEN 000 OR 010
010: 0.020402 (1644,3,140,2)

IF AFT1-RAP1 AND ALPHA1-RGM1 THEN 000 OR 010
010: 0.020402 (1644,3,140,2)

IF m_rRSE10-PDR1 AND RAP1-RGM1 THEN 000 OR 010
010: 0.020402 (1644,3,140,2)

IF SFF'-HIR3 AND RAP1-ARG81 THEN 000 OR 010
010: 0.020402 (1644,3,140,2)

IF STE12-IXR1 AND RAP1-ZAP1 THEN 000 OR 010
010: 0.020402 (1644,3,140,2)

IF SFF'-HIR1 AND RAP1-GAT3 AND RAP1-RAP1 THEN 000 OR 010
010: 0.020402 (1644,3,140,2)

IF SFF'-HIR1 AND SFF-HIR1 AND RAP1-GAT3 THEN 000 OR 010
010: 0.020402 (1644,3,140,2)

IF m_lipid_and_fatty-acid_transport_n11-GAT1 AND m_other_mrna-transcription_activities_n20-SKN7 AND m_glyoxylate_cycle_n11-GAT1 THEN 000 OR 010
010: 0.020402 (1644,3,140,2)

IF AFT1-RAP1 AND RAP1-RGM1 THEN 000 OR 010
010: 0.020402 (1644,3,140,2)

IF m_deoxyribonucleotide_metabolism_n27-MTH1 AND m_regulation_of_lipid_fatty-acid_and_isoprenoid_biosynthesis_n8.scn-MTH1 THEN 000 OR 010
010: 0.020402 (1644,3,140,2)

IF m_other_morphogenetic_activities_n7-RCS1 AND RAP1-RAP1 AND RAP1-YAP5 THEN 000 OR 010
010: 0.020402 (1644,3,140,2)

IF SFF-HIR1 AND RAP1-HAP4 AND RAP1-PDR1 THEN 000 OR 010
010: 0.020402 (1644,3,140,2)

IF SFF'-HIR3 AND RAP1-RGM1 AND ALPHA1-DAT1 THEN 000 OR 010
010: 0.020402 (1644,3,140,2)

IF SFF-HIR1 AND RAP1-HAP4 THEN 000 OR 010
010: 0.020402 (1644,3,140,2)

IF SFF'-HIR3 AND RAP1-HAP4 AND RAP1-YAP5 THEN 000 OR 010
010: 0.020402 (1644,3,140,2)

IF SFF'-HIR1 AND STE12-IXR1 THEN 000 OR 010
010: 0.020402 (1644,3,140,2)

IF m_other_morphogenetic_activities_n7-ARR1 AND STE12-IXR1 THEN 000 OR 010
010: 0.020402 (1644,3,140,2)

IF m_other_morphogenetic_activities_n7-RCS1 AND RAP1-PDR1 AND ALPHA1-GAT3 THEN 000 OR 010
010: 0.020402 (1644,3,140,2)

IF RAP1-ZAP1 AND ALPHA1-DAT1 THEN 000 OR 010
010: 0.020402 (1644,3,140,2)

IF m_other_proteolytic_degradation_n2-MTH1 AND m_anion_transporters_n19-MTH1 THEN 000 OR 010
010: 0.020402 (1644,3,140,2)

IF m_other_morphogenetic_activities_n7-RCS1 AND RAP1-PDR1 AND ALPHA1-RGM1 THEN 000 OR 010
010: 0.020402 (1644,3,140,2)

IF m_other_morphogenetic_activities_n7-RCS1 AND RAP1-PDR1 AND RAP1-RAP1 THEN 000 OR 010
010: 0.020402 (1644,3,140,2)

IF RAP1-DAT1 AND RAP1-ZAP1 THEN 000 OR 010
010: 0.020402 (1644,3,140,2)

IF RAP1-ARG81 AND ALPHA1-DAT1 THEN 000 OR 010
010: 0.020402 (1644,3,140,2)

IF m_other_morphogenetic_activities_n7-RCS1 AND RAP1-RAP1 AND ALPHA1-DAT1 THEN 000 OR 010
010: 0.020402 (1644,3,140,2)

IF SFF'-HIR1 AND RAP1-FHL1 AND RAP1-ZAP1 THEN 000 OR 010
010: 0.020402 (1644,3,140,2)

IF m_other_morphogenetic_activities_n7-RCS1 AND RAP1-FHL1 AND RAP1-RGM1 THEN 000 OR 010
010: 0.020402 (1644,3,140,2)

IF RAP1-ARG81 AND RAP1-DAT1 THEN 000 OR 010
010: 0.020402 (1644,3,140,2)

IF m_metal_ion_transporters_n14-KSS1 THEN 000 OR 010
010: 0.020402 (1644,3,140,2)

IF SFF'-HIR3 AND RAP1-FHL1 AND RAP1-HMS1 THEN 000 OR 010
010: 0.020402 (1644,3,140,2)

IF m_other_morphogenetic_activities_n7-RCS1 AND RAP1-RGM1 AND ALPHA1-RGM1 THEN 000 OR 010
010: 0.020402 (1644,3,140,2)

IF SFF-HIR1 AND RAP1-RAP1 AND RAP1-ZAP1 THEN 000 OR 010
010: 0.020402 (1644,3,140,2)

IF SFF-HIR1 AND RAP1-GAT3 AND RAP1-RAP1 THEN 000 OR 010
010: 0.020402 (1644,3,140,2)

IF m_other_morphogenetic_activities_n7-RCS1 AND RAP1-RAP1 AND RAP1-RGM1 THEN 000 OR 010
010: 0.020402 (1644,3,140,2)

IF SFF-HIR1 AND RAP1-GAT3 AND RAP1-YAP5 THEN 000 OR 010
010: 0.020402 (1644,3,140,2)

IF RAP1-ARG81 AND RAP1-ARO80 AND RAP1-RAP1 THEN 000 OR 010
010: 0.020402 (1644,3,140,2)

IF m_other_morphogenetic_activities_n7-RCS1 AND RAP1-YAP5 AND ALPHA1-GAT3 THEN 000 OR 010
010: 0.020402 (1644,3,140,2)

IF SFF-HIR1 AND RAP1-YAP5 AND RAP1-ZAP1 THEN 000 OR 010
010: 0.020402 (1644,3,140,2)

IF m_other_morphogenetic_activities_n7-RCS1 AND RAP1-GAT3 AND RAP1-PDR1 THEN 000 OR 010
010: 0.020402 (1644,3,140,2)

IF SFF'-HIR1 AND SFF'-HIR3 AND RAP1-ARG81 THEN 000 OR 010
010: 0.020402 (1644,3,140,2)

IF SFF-HIR1 AND RAP1-GAT3 AND ALPHA1-GAT3 THEN 000 OR 010
010: 0.020402 (1644,3,140,2)

IF SFF'-HIR1 AND RAP1-GAT3 AND RAP1-RAP1 AND RAP1-YAP5 THEN 000 OR 010
010: 0.020402 (1644,3,140,2)

IF RAP1-HAP4 AND RAP1-PDR1 AND RAP1-ZAP1 THEN 000 OR 010
010: 0.020402 (1644,3,140,2)

IF SFF-HIR1 AND RAP1-FHL1 AND RAP1-GAT3 THEN 000 OR 010
010: 0.020402 (1644,3,140,2)

IF SFF'-HIR3 AND SFF-ARG80 AND m_other_morphogenetic_activities_n7-ARR1 THEN 000 OR 010
010: 0.020402 (1644,3,140,2)

IF SFF-HIR1 AND RAP1-FHL1 AND ALPHA1-GAT3 THEN 000 OR 010
010: 0.020402 (1644,3,140,2)

IF SFF'-HIR1 AND RAP1-FHL1 AND RAP1-YAP5 AND ALPHA1-GAT3 THEN 000 OR 010
010: 0.020402 (1644,3,140,2)

IF m_other_morphogenetic_activities_n7-RCS1 AND RAP1-GAT3 AND RAP1-RAP1 THEN 000 OR 010
010: 0.020402 (1644,3,140,2)

IF SFF'-HIR1 AND RAP1-HAP4 AND RAP1-RAP1 THEN 000 OR 010
010: 0.020402 (1644,3,140,2)

IF RAP1-ARG81 AND RAP1-ARO80 AND RAP1-ZAP1 THEN 000 OR 010
010: 0.020402 (1644,3,140,2)

IF SFF-HIR1 AND RAP1-YAP5 AND ALPHA1-GAT3 THEN 000 OR 010
010: 0.020402 (1644,3,140,2)

IF RAP1-ARG81 AND RAP1-FHL1 AND RAP1-ZAP1 THEN 000 OR 010
010: 0.020402 (1644,3,140,2)

IF m_other_morphogenetic_activities_n7-RCS1 AND RAP1-FHL1 AND RAP1-GAT3 THEN 000 OR 010
010: 0.020402 (1644,3,140,2)

IF SFF-HIR1 AND RAP1-GAT3 THEN 000 OR 010
010: 0.020402 (1644,3,140,2)

IF m_other_morphogenetic_activities_n7-RCS1 AND RAP1-DAT1 AND RAP1-RAP1 THEN 000 OR 010
010: 0.020402 (1644,3,140,2)

IF SFF-HIR1 AND ALPHA1-GAT3 THEN 000 OR 010
010: 0.020402 (1644,3,140,2)

IF SFF'-HIR1 AND SFF'-HIR3 AND RAP1-YAP5 THEN 000 OR 010
010: 0.020402 (1644,3,140,2)

IF SFF'-HIR3 AND RAP1-ARG81 AND RAP1-PDR1 THEN 000 OR 010
010: 0.020402 (1644,3,140,2)

IF RAP1-DAT1 AND RAP1-RGM1 THEN 000 OR 010
010: 0.020402 (1644,3,140,2)

IF m_nutritional_response_pathway_n7-SWI6 AND m_anion_transporters_n10-INO4 THEN 000 OR 010
010: 0.020402 (1644,3,140,2)

IF RAP1-FHL1 AND RAP1-HMS1 AND RAP1-RAP1 THEN 000 OR 010
010: 0.020402 (1644,3,140,2)

IF m_nutritional_response_pathway_n8-INO4 AND SCB-UME6 THEN 001 OR 010
010: 0.020402 (1644,3,140,2)

IF m_nutritional_response_pathway_n8-INO4 AND m_other_energy_generation_activities_n22-INO4 THEN 000 OR 010
010: 0.020402 (1644,3,140,2)

IF SFF'-HIR1 AND SFF'-HIR3 AND RAP1-ZAP1 THEN 000 OR 010

010: 0.020402 (1644,3,140,2)

IF SFF'-HIR1 AND RAP1-ARG81 AND RAP1-YAP5 THEN 000 OR 010
010: 0.020402 (1644,3,140,2)

IF m_other_morphogenetic_activities_n7-RCS1 AND RAP1-RAP1 AND ALPHA1-RGM1 THEN 000 OR 010
010: 0.020402 (1644,3,140,2)

IF RAP1-DAT1 AND ALPHA1-RGM1 THEN 000 OR 010
010: 0.020402 (1644,3,140,2)

IF m_other_morphogenetic_activities_n7-RCS1 AND RAP1-DAT1 THEN 000 OR 010
010: 0.020402 (1644,3,140,2)

IF m_lipid_and_fatty-acid_binding_n15-FHL1 AND RAP1-RAP1 AND RAP1-YAP5 THEN 000 OR 010
010: 0.020402 (1644,3,140,2)

IF SFF-FKH1 AND ALPHA1'-RIM101 AND ALPHA1'-YER051w THEN 000 OR 010
010: 0.020402 (1644,3,140,2)

IF m_other_cell_growth_cell_division_and_dna_synthesis_activities_n10.scn-MGA1 AND ALPHA1-GAT3 THEN 000 OR 010
010: 0.020402 (1644,3,140,2)

IF SFF'-RLM1 AND ABF1-PPR1 THEN 000 OR 010
010: 0.020402 (1644,3,140,2)

IF m_other_pheromone_response_activities_n14-FHL1 AND ALPHA1-GAT3 THEN 000 OR 010
010: 0.020402 (1644,3,140,2)

IF m_other_cell_growth_cell_division_and_dna_synthesis_activities_n10.scn-MGA1 AND ALPHA1-RGM1 THEN 000 OR 010
010: 0.020402 (1644,3,140,2)

IF RAP1-RAP1 AND ALPHA1-DAT1 AND ALPHA1-RGM1 THEN 000 OR 010
010: 0.020402 (1644,3,140,2)

IF m_other_pheromone_response_activities_n14-FHL1 AND ALPHA1-RGM1 THEN 000 OR 010
010: 0.020402 (1644,3,140,2)

IF SFF'-HIR3 AND RAP1-HAP4 AND RAP1-RAP1 THEN 000 OR 010
010: 0.020402 (1644,3,140,2)

IF m_other_morphogenetic_activities_n7-RCS1 AND RAP1-FHL1 AND ALPHA1-RGM1 THEN 000 OR 010
010: 0.020402 (1644,3,140,2)

IF RAP1-GAT3 AND ALPHA1-DAT1 AND ALPHA1-RGM1 THEN 000 OR 010
010: 0.020402 (1644,3,140,2)

IF SFF'-HIR1 AND ALPHA1-GAT3 THEN 000 OR 010
010: 0.020402 (1644,3,140,2)

IF m_other_morphogenetic_activities_n7-RCS1 AND RAP1-RAP1 AND ALPHA1-GAT3 THEN 000 OR 010
010: 0.020402 (1644,3,140,2)

IF SFF'-HIR1 AND RAP1-GAT3 THEN 000 OR 010
010: 0.020402 (1644,3,140,2)

IF m_cytoskeleton-dependenttransport_n4-MBP1 THEN 000 OR 100 OR 110 OR 111
110: 0.020523 (1644,9,115,3)

IF MCM1-NDD1 AND MCM1-SWI4 THEN 000 OR 100 OR 110
110: 0.020523 (1644,9,115,3)

IF m_other_transport_facilitators_n5-SWI6 THEN 000 OR 010 OR 100 OR 110
110: 0.020523 (1644,9,115,3)

IF SFF'-RLM1 AND SWI5-SWI6 THEN 000 OR 100 OR 110 OR 111
110: 0.020523 (1644,9,115,3)

IF m_osmosensing_n6-SWI4 AND SCB-SWI4 THEN 000 OR 010 OR 110 OR 111
110: 0.020523 (1644,9,115,3)

IF m_other_signal-transduction_activities_n8-INO4 AND m_glyoxylate_cycle_n8-INO4 THEN 000 OR 100 OR 110
110: 0.020523 (1644,9,115,3)

IF m_g-proteins_n12-SKN7 AND SWI5-SKN7 AND m_amino-acid_transporters_n11-SKN7 THEN 000 OR 001 OR 010 OR 110
110: 0.020523 (1644,9,115,3)

IF m_g-proteins_n12-INO4 AND m_other_signal-transduction_activities_n8-INO4 THEN 000 OR 001 OR 100 OR 110
110: 0.020523 (1644,9,115,3)

IF SFF'-FKH1 AND MCM1'-SWI4 THEN 000 OR 100 OR 110 OR 111
110: 0.020523 (1644,9,115,3)

IF SFF'-FKH1 AND ECB-FKH2 THEN 000 OR 010 OR 110 OR 111
110: 0.020523 (1644,9,115,3)

IF m_lipid_and_fatty-acid_transport_n11-GAT1 AND SWI5-SWI6 THEN 000 OR 001 OR 100 OR 110
110: 0.020523 (1644,9,115,3)

IF SFF'-FKH1 AND m_other_morphogenetic_activities_n7-MCM1 THEN 000 OR 010 OR 100 OR 110 OR 111
110: 0.020523 (1644,9,115,3)

IF m_osmosensing_n6-INO4 AND m_osmosensing_n6-SWI4 THEN 000 OR 010 OR 100 OR 110
110: 0.020523 (1644,9,115,3)

IF SWI5-SWI6 AND m_other_transport_facilitators_n10-SWI6 THEN 000 OR 010 OR 110
110: 0.020523 (1644,9,115,3)

IF m_other_mrna-transcription_activities_n20-RAP1 AND m_amino-acid_transport_n20-MTH1 THEN 000 OR 001 OR 110
001: 0.020721 (1644,7,55,2)

IF m_anion_transporters_n10-INO4 AND m_amino-acid_transport_n20-INO4 THEN 000 OR 001
001: 0.020721 (1644,7,55,2)

IF m_other_mrna-transcription_activities_n20-RAP1 AND m_amino-acid_transport_n20-MTH1 AND m_amino-acid_transport_n20-RAP1 THEN 000 OR 001 OR 110
001: 0.020721 (1644,7,55,2)

IF m_other_proteolytic_degradation_n2-MTH1 AND m_anion_transporters_n9-MTH1 THEN 000 OR 001 OR 010 OR 110
001: 0.020721 (1644,7,55,2)

IF SFF'-HIR3 AND m_g-proteins_n12-NRG1 THEN 000 OR 001 OR 100
001: 0.020721 (1644,7,55,2)

IF m_nutritional_response_pathway_n8-INO4 AND m_other_mrna-transcription_activities_n20-INO4 THEN 000 OR 001 OR 010 OR 110
001: 0.020721 (1644,7,55,2)

IF SFF'-MCM1 AND SFF-FKH2 THEN 000 OR 010 OR 011 OR 100 OR 110 OR 111
111: 0.021247 (1644,20,19,2)

IF m_nucleotide_transport_n9-MCM1 THEN 000 OR 010 OR 100 OR 101 OR 110
110: 0.021435 (1644,16,115,4)

IF SWI5-RIM101 THEN 000 OR 010 OR 100 OR 110
100: 0.021697 (1644,46,127,8)

IF mPROTEOL18(m_proteolysis_n18)-SWI6 THEN 000 OR 010 OR 100 OR 110 OR 111
110: 0.022268 (1644,24,115,5)

IF SFF'-HIR3 AND SFF-ARG80 THEN 000 OR 001 OR 010 OR 011 OR 110
010: 0.022673 (1644,20,140,5)

IF m_phosphate_transport_n18-YAP6 THEN 000 OR 001 OR 010 OR 011 OR 100 OR 110 OR 111
111: 0.023323 (1644,21,19,2)

IF SWI5-SWI6 THEN 000 OR 001 OR 010 OR 011 OR 100 OR 101 OR 110 OR 111
111: 0.023487 (1644,55,19,3)

IF m_metabolism_of_energy_reserves_n30-EDS1 THEN 000 OR 010
010: 0.024607 (1644,8,140,3)

IF m_trna_processing_n6-STP1 AND m_other_morphogenetic_activities_n7-STP1 THEN 000 OR 010 OR 110
010: 0.024607 (1644,8,140,3)

IF SFF'-HIR3 AND ALPHA1-GAT3 THEN 000 OR 010 OR 100
010: 0.024607 (1644,8,140,3)

IF m_MERE4-MTH1 AND m_glycolysis_and_gluconeogenesis_n14-MTH1 THEN 000 OR 010
010: 0.024607 (1644,8,140,3)

IF SFF'-FKH1 AND SFF'-MCM1 THEN 000 OR 010 OR 100 OR 110 OR 111
111: 0.025479 (1644,22,19,2)

IF m_other_mrna-transcription_activities_n20-INO4 THEN 000 OR 001 OR 010 OR 100 OR 110
110: 0.02632 (1644,25,115,5)

IF SWI5-SKN7 AND SWI5-SWI6 THEN 000 OR 010 OR 100 OR 110 OR 111
110: 0.02632 (1644,25,115,5)

IF m_morphogenesis_n5-SWI6 AND MCB-MBP1 THEN 010 OR 100 OR 110
110: 0.026518 (1644,4,115,2)

IF m_other_mrna-transcription_activities_n20-INO4 AND m_other_mrna-transcription_activities_n20-RAP1 AND m_anion_transporters_n20-RAP1 THEN 000 OR 001 OR 110
110: 0.026518 (1644,4,115,2)

IF m_allantoin_and_allantoate_transporters_n7-ACE2 AND m_allantoin_and_allantoate_transporters_n12-ACE2 THEN 000 OR 110
110: 0.026518 (1644,4,115,2)

IF SFF'-MCM1 AND MCM1-YOX1 AND SFF-FKH2 THEN 000 OR 110 OR 111
110: 0.026518 (1644,4,115,2)

IF SFF'-FKH1 AND m_other_pheromone_response_activities_n8-SWI6 THEN 000 OR 010 OR 110
110: 0.026518 (1644,4,115,2)

IF SFF-FKH1 AND m_other_pheromone_response_activities_n8-SWI6 THEN 000 OR 010 OR 110
110: 0.026518 (1644,4,115,2)

IF m_nucleotide_transport_n9-SWI6 AND MCB-SWI6 THEN 000 OR 010 OR 110
110: 0.026518 (1644,4,115,2)

IF m_other_energy_generation_activities_n12-MTH1 AND m_deoxyribonucleotide_metabolism_n23-MTH1 THEN 000 OR 110
110: 0.026518 (1644,4,115,2)

IF SFF'-FKH1 AND m_ionic_homeostasis_n6-SWI6 THEN 000 OR 110
110: 0.026518 (1644,4,115,2)

IF SFF-FKH1 AND m_ionic_homeostasis_n6-SWI6 THEN 000 OR 110
110: 0.026518 (1644,4,115,2)

IF m_other_energy_generation_activities_n12-MTH1 AND m_deoxyribonucleotide_metabolism_n23-SWI4 THEN 000 OR 110
110: 0.026518 (1644,4,115,2)

IF LYS14-SWI6 AND m_ionic_homeostasis_n6-SWI6 THEN 000 OR 110
110: 0.026518 (1644,4,115,2)

IF m_breakdown_of_lipids_fatty_acids_and_isoprenoids_n8-MBP1 AND m_cytoskeleton-dependenttransport_n4-ACE2 THEN 000 OR 110 OR 111
110: 0.026518 (1644,4,115,2)

IF ECB-DIG1 AND ECB-FKH2 THEN 000 OR 110
110: 0.026518 (1644,4,115,2)

IF SFF-FKH2 AND SCB-SWI4 THEN 000 OR 110 OR 111
110: 0.026518 (1644,4,115,2)

IF MCM1'-NDD1 AND mRRPE-RLM1 THEN 000 OR 110
110: 0.026518 (1644,4,115,2)

IF m_other_energy_generation_activities_n12-SWI6 AND MCM1'-SWI4 THEN 000 OR 101 OR 110

110: 0.026518 (1644,4,115,2)

IF SFF'-MCM1 AND m_osmosensing_n6-ROX1 THEN 000 OR 110
110: 0.026518 (1644,4,115,2)

IF m_other_energy_generation_activities_n12-FKH1 AND SFF-FKH2 THEN 000 OR 010 OR 110
110: 0.026518 (1644,4,115,2)

IF SFF'-RLM1 AND m_osmosensing_n6-SWI4 THEN 000 OR 110
110: 0.026518 (1644,4,115,2)

IF SFF'-RLM1 AND MCM1'-MCM1 AND SFF-FKH2 THEN 000 OR 110 OR 111
110: 0.026518 (1644,4,115,2)

IF SFF'-MCM1 AND SFF'-RLM1 AND SFF-FKH2 THEN 000 OR 110 OR 111
110: 0.026518 (1644,4,115,2)

IF SFF'-FKH2 AND SFF'-RLM1 AND MCM1'-MCM1 THEN 000 OR 110 OR 111
110: 0.026518 (1644,4,115,2)

IF m_other_energy_generation_activities_n12-FKH1 AND m_other_energy_generation_activities_n12-SWI6 THEN 000 OR 110
110: 0.026518 (1644,4,115,2)

IF m_osmosensing_n6-INO4 AND MCM1'-SWI4 THEN 000 OR 100 OR 110
110: 0.026518 (1644,4,115,2)

IF m_other_signal-transduction_activities_n8-INO4 AND m_phosphate_transport_n18-INO4 THEN 000 OR 110
110: 0.026518 (1644,4,115,2)

IF m_glyoxylate_cycle_n8-INO4 AND m_other_mrna-transcription_activities_n20-INO4 AND m_homeostasis_of_other_ions_n30-INO4 THEN 000 OR 010 OR 110
110: 0.026518 (1644,4,115,2)

IF m_drug_transporters_n10-INO4 AND m_drug_transporters_n10-SKN7 THEN 000 OR 110
110: 0.026518 (1644,4,115,2)

IF m_osmosensing_n6-INO4 AND m_drug_transporters_n10-INO4 THEN 000 OR 100 OR 110
110: 0.026518 (1644,4,115,2)

IF m_g-proteins_n11-INO4 AND m_glyoxylate_cycle_n8-INO4 AND m_other_mrna-transcription_activities_n20-INO4 THEN 000 OR 110
110: 0.026518 (1644,4,115,2)

IF SFF-FKH1 AND SFF-FKH2 AND m_amino-acid_degradation_n7-NDD1 THEN 000 OR 110
110: 0.026518 (1644,4,115,2)

IF ECB-YOX1 AND SFF-FKH2 THEN 000 OR 110 OR 111
110: 0.026518 (1644,4,115,2)

IF SFF'-MCM1 AND ECB-FKH2 AND SFF-FKH1 THEN 000 OR 110 OR 111
110: 0.026518 (1644,4,115,2)

IF SFF'-FKH2 AND SFF'-MCM1 AND SFF'-RLM1 THEN 000 OR 110 OR 111
110: 0.026518 (1644,4,115,2)

IF SFF'-FKH1 AND ECB-FKH2 AND SFF-FKH2 AND ALPHA1'-UPC2 THEN 000 OR 110
110: 0.026518 (1644,4,115,2)

IF m_g-proteins_n12-SKN7 AND m_other_protein-destination_activities_n7-MET4 THEN 000 OR 110
110: 0.026518 (1644,4,115,2)

IF ECB-FKH2 AND SFF-FKH1 AND ALPHA1'-UPC2 THEN 000 OR 110
110: 0.026518 (1644,4,115,2)

IF SFF'-FKH1 AND ECB-FKH2 AND ALPHA1'-UPC2 THEN 000 OR 110
110: 0.026518 (1644,4,115,2)

IF m_phosphate_transport_n8-SWI4 AND m_phosphate_transport_n8-SWI5 THEN 000 OR 110
110: 0.026518 (1644,4,115,2)

IF ECB-FKH2 AND SFF-FKH1 AND SFF-FKH2 AND ALPHA1'-UPC2 THEN 000 OR 110

110: 0.026518 (1644,4,115,2)

IF MCM1'-SWI4 AND m_drug_transporters_n9-SWI4 THEN 000 OR 110
110: 0.026518 (1644,4,115,2)

IF SFF'-FKH2 AND ECB-FKH2 AND SFF-FKH1 AND ALPHA1'-UPC2 THEN 000 OR 110
110: 0.026518 (1644,4,115,2)

IF MCM1-FKH2 AND MCM1'-SWI4 AND ECB-FKH2 THEN 000 OR 110
110: 0.026518 (1644,4,115,2)

IF Gcr1-SWI6 AND mPROTEOL18(m_proteolysis_n18)-SWI6 THEN 000 OR 110
110: 0.026518 (1644,4,115,2)

IF Gcr1-SWI6 AND mPROTEOL18(m_proteolysis_n18)-MBP1 THEN 000 OR 110
110: 0.026518 (1644,4,115,2)

IF MCM1-SWI4 AND ECB-NDD1 THEN 000 OR 110
110: 0.026518 (1644,4,115,2)

IF ECB-FKH2 AND SWI5-SWI6 THEN 000 OR 110 OR 111
110: 0.026518 (1644,4,115,2)

IF MCM1'-SWI4 AND m_cell_death_n16-MBP1 THEN 000 OR 110 OR 111
110: 0.026518 (1644,4,115,2)

IF MCM1'-SWI4 AND m_cell_death_n22-TOS8 THEN 000 OR 110
110: 0.026518 (1644,4,115,2)

IF m_deoxyribonucleotide_metabolism_n12-TOS8 AND m_cell_death_n22-TOS8 THEN 000 OR 110
110: 0.026518 (1644,4,115,2)

IF MCM1-SWI4 AND ECB-FKH2 THEN 000 OR 110
110: 0.026518 (1644,4,115,2)

IF ECB-NDD1 AND SWI5-SWI6 THEN 000 OR 110 OR 111
110: 0.026518 (1644,4,115,2)

IF SFF-FKH2 AND mPROTEOL18(m_proteolysis_n18)-SWI6 THEN 000 OR 110
110: 0.026518 (1644,4,115,2)

IF MCM1-NDD1 AND MCM1'-SWI4 AND ECB-FKH2 THEN 000 OR 110
110: 0.026518 (1644,4,115,2)

IF MCM1-SWI4 AND SFF-FKH2 THEN 000 OR 100 OR 110
110: 0.026518 (1644,4,115,2)

IF MCM1-NDD1 AND MCM1-SWI4 AND ECB-NDD1 THEN 000 OR 110
110: 0.026518 (1644,4,115,2)

IF MCM1-SWI4 AND MCM1'-SWI4 AND SFF-FKH2 THEN 000 OR 100 OR 110
110: 0.026518 (1644,4,115,2)

IF MCM1-FKH2 AND MCM1-SWI4 AND ECB-NDD1 THEN 000 OR 110
110: 0.026518 (1644,4,115,2)

IF MCM1-NDD1 AND MCM1-SWI4 AND ECB-FKH2 THEN 000 OR 110
110: 0.026518 (1644,4,115,2)

IF Gcr1-SWI6 AND m_nitrogen_and_sulphur_metabolism_n16-SWI6 THEN 000 OR 101 OR 110
110: 0.026518 (1644,4,115,2)

IF MCM1-SWI4 AND MCM1'-NDD1 AND ECB-NDD1 THEN 000 OR 110
110: 0.026518 (1644,4,115,2)

IF MCM1-FKH2 AND MCM1'-SWI4 AND ECB-NDD1 THEN 000 OR 110
110: 0.026518 (1644,4,115,2)

IF ECB-FKH2 AND SFF-FKH2 AND SWI5-SWI6 THEN 000 OR 110 OR 111
110: 0.026518 (1644,4,115,2)

IF SFF'-FKH2 AND MCM1'-SWI4 AND ECB-NDD1 THEN 000 OR 110
110: 0.026518 (1644,4,115,2)

IF m_glyoxylate_cycle_n8-INO4 AND m_amino-acid_transporters_n11-SKN7 THEN 000 OR 110 OR 111
110: 0.026518 (1644,4,115,2)

IF MCM1-NDD1 AND MCM1'-NDD1 AND MCM1'-SWI4 AND ECB-FKH2 THEN 000 OR 110
110: 0.026518 (1644,4,115,2)

IF MCM1-FKH2 AND MCM1-NDD1 AND MCM1'-SWI4 AND ECB-FKH2 THEN 000 OR 110
110: 0.026518 (1644,4,115,2)

IF SWI5-INO4 AND m_other_transport_facilitators_n5-CIN5 THEN 000 OR 100 OR 110
110: 0.026518 (1644,4,115,2)

IF MCM1-FKH2 AND MCM1-SWI4 AND MCM1'-NDD1 AND ECB-NDD1 THEN 000 OR 110
110: 0.026518 (1644,4,115,2)

IF MCM1-FKH2 AND MCM1-SWI4 AND ECB-FKH2 THEN 000 OR 110
110: 0.026518 (1644,4,115,2)

IF MCM1-SWI4 AND m_drug_transporters_n9-SWI4 THEN 000 OR 110
110: 0.026518 (1644,4,115,2)

IF MCM1-SWI4 AND ECB-FKH2 AND ECB-NDD1 THEN 000 OR 110
110: 0.026518 (1644,4,115,2)

IF m_glyoxylate_cycle_n8-INO4 AND SWI5-SWI6 THEN 000 OR 110
110: 0.026518 (1644,4,115,2)

IF m_g-proteins_n12-INO4 AND m_amino-acid_transporters_n11-SKN7 THEN 001 OR 110 OR 111
110: 0.026518 (1644,4,115,2)

IF m_g-proteins_n11-INO4 AND m_other_transport_facilitators_n5-INO4 THEN 000 OR 010 OR 110
110: 0.026518 (1644,4,115,2)

IF SFF'-FKH1 AND m_phosphate_transport_n18-ASH1 THEN 000 OR 100 OR 110
110: 0.026518 (1644,4,115,2)

IF SFF-FKH2 AND m_phosphate_transport_n18-ASH1 THEN 000 OR 100 OR 110
110: 0.026518 (1644,4,115,2)

IF SFF-FKH1 AND m_phosphate_transport_n18-ASH1 THEN 000 OR 100 OR 110
110: 0.026518 (1644,4,115,2)

IF SFF-FKH1 AND m_nucleotide_transport_n9-SWI6 THEN 000 OR 010 OR 110
110: 0.026518 (1644,4,115,2)

IF mPROTEOL18(m_proteolysis_n18)-MBP1 AND m_nucleotide_transport_n9-SWI6 THEN 000 OR 100 OR 110
110: 0.026518 (1644,4,115,2)

IF m_osmosensing_n6-INO4 AND m_other_signal-transduction_activities_n8-INO4 THEN 000 OR 110
110: 0.026518 (1644,4,115,2)

IF SFF-FKH1 AND m_LFTE17-GAT1 THEN 000 OR 010 OR 110
110: 0.026518 (1644,4,115,2)

IF SFF'-MCM1 AND ALPHA1'-STB4 THEN 000 OR 001 OR 110
110: 0.026518 (1644,4,115,2)

IF MCM1-FKH2 AND MCM1-MCM1 AND MCM1-SWI4 THEN 000 OR 100 OR 110
110: 0.026518 (1644,4,115,2)

IF SFF-FKH1 AND SFF-FKH2 AND m_phosphate_transport_n18-ASH1 THEN 000 OR 100 OR 110
110: 0.026518 (1644,4,115,2)

IF SFF'-FKH2 AND SFF'-MCM1 AND m_osmosensing_n6-SWI4 THEN 100 OR 110 OR 111
110: 0.026518 (1644,4,115,2)

IF m_lipid_and_fatty-acid_transport_n11-MTH1 AND m_anion_transporters_n20-GAT1 THEN 000 OR 110 OR 111
110: 0.026518 (1644,4,115,2)

IF SFF'-FKH1 AND m_nucleotide_transport_n9-MCM1 THEN 000 OR 010 OR 110
110: 0.026518 (1644,4,115,2)

IF m_lipid_and_fatty-acid_transport_n11-GAT1 AND m_anion_transporters_n20-MTH1 THEN 000 OR 110 OR 111
110: 0.026518 (1644,4,115,2)

IF SFF'-MCM1 AND m_nucleotide_transport_n9-SWI6 THEN 000 OR 101 OR 110
110: 0.026518 (1644,4,115,2)

IF SFF'-FKH1 AND m_LFTE17-GAT1 THEN 000 OR 010 OR 110
110: 0.026518 (1644,4,115,2)

IF SFF'-FKH1 AND SFF-FKH1 AND m_phosphate_transport_n18-ASH1 THEN 000 OR 100 OR 110
110: 0.026518 (1644,4,115,2)

IF SFF'-FKH2 AND SFF-FKH1 AND m_phosphate_transport_n18-ASH1 THEN 000 OR 100 OR 110
110: 0.026518 (1644,4,115,2)

IF MCM1-MCM1 AND MCM1-NDD1 AND MCM1-SWI4 THEN 000 OR 100 OR 110
110: 0.026518 (1644,4,115,2)

IF SFF-FKH2 AND m_phosphate_transport_n8-SWI4 THEN 000 OR 110
110: 0.026518 (1644,4,115,2)

IF SWI5-SWI6 AND m_regulation_of_nitrogen_and_sulphur_utilization_n13-MTH1 THEN 000 OR 001 OR 110
110: 0.026518 (1644,4,115,2)

IF m_anion_transporters_n20-GAT1 AND m_anion_transporters_n20-MTH1 THEN 000 OR 110 OR 111
110: 0.026518 (1644,4,115,2)

IF m_lipid_and_fatty-acid_transport_n11-MTH1 AND SWI5-SWI6 AND m_anion_transporters_n15-MTH1 THEN 000 OR 110
110: 0.026518 (1644,4,115,2)

IF m_other_mrna-transcription_activities_n20-INO4 AND m_LFTE17-GAT1 THEN 000 OR 010 OR 110
110: 0.026518 (1644,4,115,2)

IF m_g-proteins_n11-INO4 AND SWI5-SWI6 AND m_other_mrna-transcription_activities_n20-INO4 THEN 010 OR 100 OR 110
110: 0.026518 (1644,4,115,2)

IF m_nitrogen_and_sulphur_metabolism_n16-SWI6 AND mPROTEOL18(m_proteolysis_n18)-SWI6 THEN 000 OR 110
110: 0.026518 (1644,4,115,2)

IF m_osmosensing_n6-INO4 AND m_glyoxylate_cycle_n8-INO4 AND SWI5-SKN7 THEN 000 OR 010 OR 110
110: 0.026518 (1644,4,115,2)

IF m_phosphate_transport_n5-INO4 AND SWI5-SWI6 THEN 000 OR 010 OR 110
110: 0.026518 (1644,4,115,2)

IF m_stress_response_n24-SWI4 AND SCB-SWI4 THEN 000 OR 010 OR 110
110: 0.026518 (1644,4,115,2)

IF m_g-proteins_n12-SKN7 AND m_glyoxylate_cycle_n8-INO4 AND SWI5-INO4 THEN 000 OR 010 OR 110
110: 0.026518 (1644,4,115,2)

IF m_g-proteins_n12-INO4 AND m_glyoxylate_cycle_n8-INO4 AND SWI5-SKN7 THEN 000 OR 010 OR 110
110: 0.026518 (1644,4,115,2)

IF m_g-proteins_n12-SKN7 AND m_glyoxylate_cycle_n8-INO4 AND SWI5-SKN7 THEN 000 OR 010 OR 110
110: 0.026518 (1644,4,115,2)

IF m_g-proteins_n12-INO4 AND m_glyoxylate_cycle_n8-INO4 AND SWI5-INO4 AND SWI5-SKN7 THEN 000 OR 010 OR 110
110: 0.026518 (1644,4,115,2)

IF SWI5-SWI6 AND m_pentose-phosphate_pathway_n14-MTH1 THEN 000 OR 110
110: 0.026518 (1644,4,115,2)

IF MCM1-NDD1 AND m_other_cell_growth_cell_division_and_dna_synthesis_activities_n10.scn-NDD1 THEN 000 OR 010 OR 110

110: 0.026518 (1644,4,115,2)

IF m_other_morphogenetic_activities_n7-MCM1 AND PHO-NDD1 THEN 000 OR 010 OR 110
110: 0.026518 (1644,4,115,2)

IF m_other_morphogenetic_activities_n7-MCM1 AND m_other_cell_growth_cell_division_and_dna_synthesis_activities_n10.scn-NDD1 THEN 000 OR 010 OR 110
110: 0.026518 (1644,4,115,2)

IF SWI5-INO4 AND m_deoxyribonucleotide_metabolism_n23-MTH1 THEN 000 OR 110
110: 0.026518 (1644,4,115,2)

IF SFF'-FKH2 AND PHO-NDD1 THEN 000 OR 010 OR 110
110: 0.026518 (1644,4,115,2)

IF MCM1-FKH2 AND PHO-NDD1 THEN 000 OR 010 OR 110
110: 0.026518 (1644,4,115,2)

IF SFF'-FKH2 AND m_g-proteins_n12-INO4 AND SFF-FKH1 THEN 000 OR 110
110: 0.026518 (1644,4,115,2)

IF m_drug_transporters_n10-INO4 AND m_other_energy_generation_activities_n22-INO4 THEN 000 OR 110
110: 0.026518 (1644,4,115,2)

IF SWI5-SWI6 AND m_anion_transporters_n15-MTH1 THEN 000 OR 110
110: 0.026518 (1644,4,115,2)

IF m_other_transport_facilitators_n5-INO4 AND m_pentose-phosphate_pathway_n14-MTH1 THEN 000 OR 110
110: 0.026518 (1644,4,115,2)

IF m_osmosensing_n6-INO4 AND m_lipid_and_fatty-acid_transport_n11-SKN7 THEN 000 OR 010 OR 110
110: 0.026518 (1644,4,115,2)

IF m_lipid_and_fatty-acid_transport_n11-GAT1 AND m_lipid_and_fatty-acid_transport_n11-MTH1 AND m_anion_transporters_n20-MTH1 THEN 000 OR 110 OR 111
110: 0.026518 (1644,4,115,2)

IF m_lipid_and_fatty-acid_transport_n11-GAT1 AND m_lipid_and_fatty-acid_transport_n11-MTH1 AND m_anion_transporters_n20-GAT1 THEN 000 OR 110 OR 111
110: 0.026518 (1644,4,115,2)

IF m_nitrogen_and_sulphur_metabolism_n17-SKN7 AND m_other_mrna-transcription_activities_n20-SKN7 THEN 000 OR 110
110: 0.026518 (1644,4,115,2)

IF m_other_signal-transduction_activities_n8-INO4 AND m_g-proteins_n11-INO4 THEN 000 OR 001 OR 110
110: 0.026518 (1644,4,115,2)

IF SWI5-SWI6 AND STRE'-MBP1 THEN 000 OR 101 OR 110
110: 0.026518 (1644,4,115,2)

IF ECB-FKH2 AND PHO-NDD1 THEN 000 OR 010 OR 110
110: 0.026518 (1644,4,115,2)

IF MCM1-MCM1 AND m_other_morphogenetic_activities_n7-MCM1 AND m_nucleotide_transport_n9-MCM1 THEN 010 OR 110
110: 0.026518 (1644,4,115,2)

IF m_chromatin_modification_n9-MBP1 THEN 000 OR 100 OR 110
110: 0.026518 (1644,4,115,2)

IF ECB-MCM1 AND m_other_morphogenetic_activities_n7-MCM1 AND m_nucleotide_transport_n9-MCM1 THEN 010 OR 110
110: 0.026518 (1644,4,115,2)

IF m_allantoin_and_allantoate_transporters_n13-SWI6 AND MCB-SWI6 THEN 000 OR 011 OR 110
110: 0.026518 (1644,4,115,2)

IF MCM1-MCM1 AND m_allantoin_and_allantoate_transporters_n18-FKH2 THEN 010 OR 100 OR 110
110: 0.026518 (1644,4,115,2)

IF ECB-MCM1 AND m_pentose-phosphate_pathway_n5-MCM1 THEN 000 OR 110 OR 111
110: 0.026518 (1644,4,115,2)

IF MCM1'-SWI4 AND MCB-SWI6 THEN 000 OR 110 OR 111
110: 0.026518 (1644,4,115,2)

IF m_other_morphogenetic_activities_n7-ARR1 AND m_other_morphogenetic_activities_n7-RTG1 THEN 000 OR 110
110: 0.026518 (1644,4,115,2)

IF m_g-proteins_n12-SKN7 AND m_nitrogen_and_sulphur_metabolism_n17-XBP1 THEN 000 OR 110
110: 0.026518 (1644,4,115,2)

IF m_allantoin_and_allantoate_transporters_n13-SWI6 AND m_nucleotide_transport_n9-SWI6 THEN 000 OR 101 OR 110
110: 0.026518 (1644,4,115,2)

IF m_g-proteins_n12-SKN7 AND m_nucleotide_transport_n9-SWI6 THEN 000 OR 110
110: 0.026518 (1644,4,115,2)

IF SFF-FKH1 AND m_regulation_of_lipid_fatty-acid_and_isoprenoid_biosynthesis_n8.scn-GAT1 THEN 000 OR 100 OR 110
110: 0.026518 (1644,4,115,2)

IF SWI5-SWI6 AND m_regulation_of_lipid_fatty-acid_and_isoprenoid_biosynthesis_n8.scn-FKH1 THEN 000 OR 110
110: 0.026518 (1644,4,115,2)

IF LYS14-SWI6 AND m_regulation_of_lipid_fatty-acid_and_isoprenoid_biosynthesis_n8.scn-FKH1 THEN 000 OR 110
110: 0.026518 (1644,4,115,2)

IF MCM1-FKH2 AND MCM1-MCM1 AND ECB-MCM1 AND SFF-FKH1 THEN 000 OR 110 OR 111
110: 0.026518 (1644,4,115,2)

IF MCM1-FKH2 AND MCM1'-MCM1 AND ECB-MCM1 AND SFF-FKH1 THEN 000 OR 110 OR 111
110: 0.026518 (1644,4,115,2)

IF MCM1-NDD1 AND MCM1'-MCM1 AND ECB-MCM1 AND SFF-FKH1 THEN 000 OR 110 OR 111
110: 0.026518 (1644,4,115,2)

IF MCM1'-NDD1 AND MCM1'-SWI4 AND SFF-FKH1 THEN 000 OR 100 OR 110
110: 0.026518 (1644,4,115,2)

IF MCM1-FKH2 AND ECB-MCM1 AND SFF-FKH1 THEN 000 OR 110 OR 111
110: 0.026518 (1644,4,115,2)

IF MCM1-NDD1 AND ECB-MCM1 AND SFF-FKH1 THEN 000 OR 110 OR 111
110: 0.026518 (1644,4,115,2)

IF MCM1-MCM1 AND MCM1'-MCM1 AND MCM1'-NDD1 AND ECB-MCM1 AND SFF-FKH1 THEN 000 OR 110 OR 111
110: 0.026518 (1644,4,115,2)

IF MCM1-FKH2 AND MCM1'-SWI4 AND SFF-FKH2 THEN 000 OR 100 OR 110
110: 0.026518 (1644,4,115,2)

IF MCM1'-MCM1 AND MCM1'-SWI4 AND SFF-FKH2 THEN 000 OR 100 OR 110
110: 0.026518 (1644,4,115,2)

IF MCM1'-MCM1 AND MCM1'-NDD1 AND MCM1'-SWI4 THEN 000 OR 100 OR 110
110: 0.026518 (1644,4,115,2)

IF SWI5-SWI6 AND m_cytoskeleton-dependenttransport_n4-ACE2 THEN 000 OR 110 OR 111
110: 0.026518 (1644,4,115,2)

IF m_MERE4-MTH1 AND m_phosphate_transport_n13-GAT1 THEN 010 OR 100 OR 110
110: 0.026518 (1644,4,115,2)

IF m_pentose-phosphate_pathway_n21-GAT1 AND m_pentose-phosphate_pathway_n14-FKH1 THEN 000 OR 110
110: 0.026518 (1644,4,115,2)

IF SFF-FKH1 AND RPN4-GAT1 THEN 000 OR 110
110: 0.026518 (1644,4,115,2)

IF m_phosphate_transport_n13-GAT1 AND m_pentose-phosphate_pathway_n14-MTH1 THEN 010 OR 100 OR 110
110: 0.026518 (1644,4,115,2)

IF m_pentose-phosphate_pathway_n21-GAT1 AND m_phosphate_transport_n13-GAT1 THEN 000 OR 010 OR 110
110: 0.026518 (1644,4,115,2)

IF SFF-FKH1 AND m_regulation_of_lipid_fatty-acid_and_isoprenoid_biosynthesis_n8.scn-FKH1 AND m_regulation_of_lipid_fatty-acid_and_isoprenoid_biosynthesis_n8.scn-GAT1 THEN 000 OR 100 OR 110
110: 0.026518 (1644,4,115,2)

IF m_other_mrna-transcription_activities_n20-INO4 AND m_anion_transporters_n20-RAP1 THEN 000 OR 001 OR 110
110: 0.026518 (1644,4,115,2)

IF m_morphogenesis_n5-SWI6 AND MCB-SWI6 THEN 010 OR 100 OR 110
110: 0.026518 (1644,4,115,2)

IF m_g-proteins_n12-SKN7 AND SWI5-SWI6 THEN 000 OR 010 OR 100 OR 110 OR 111
110: 0.02655 (1644,17,115,4)

IF m_g-proteins_n12-SKN7 AND SWI5-SKN7 AND SWI5-SWI6 THEN 000 OR 010 OR 100 OR 110 OR 111
110: 0.02655 (1644,17,115,4)

IF SFF'-MCM1 AND m_other_morphogenetic_activities_n7-MCM1 THEN 000 OR 001 OR 010 OR 011 OR 100 OR 101 OR 110 OR 111
110: 0.026714 (1644,43,115,7)

IF m_other_nucleotide-metabolism_activities_n17-NNF2 THEN 000 OR 001 OR 100
100: 0.026782 (1644,9,127,3)

IF m_RRSE3-SWI4 AND SCB-SWI4 THEN 000 OR 010 OR 100 OR 110
100: 0.026782 (1644,9,127,3)

IF m_phosphate_transport_n18-GAT1 AND m_purine_and_pyrimidine_transporters_n17-GAT1 THEN 000 OR 100 OR 110
100: 0.026782 (1644,9,127,3)

IF m_allantoin_and_allantoate_transporters_n18-HAP2 THEN 000 OR 001 OR 100 OR 110
100: 0.026782 (1644,9,127,3)

IF m_other_pheromone_response_activities_n14-PPR1 THEN 000 OR 010 OR 100 OR 110
100: 0.026782 (1644,9,127,3)

IF m_ion_transporters_n4-INO4 AND m_phosphate_transport_n18-INO4 THEN 000 OR 100
100: 0.026782 (1644,9,127,3)

IF m_lipid_and_fatty-acid_transport_n11-GAT1 AND m_homeostasis_of_other_ions_n30-GAT1 THEN 000 OR 001 OR 010 OR 110 OR 111
001: 0.027041 (1644,8,55,2)

IF m_RPE32-FHL1 THEN 000 OR 001 OR 100
001: 0.027041 (1644,8,55,2)

IF m_anion_transporters_n22-SKN7 AND SWI5-SKN7 THEN 000 OR 001
001: 0.027041 (1644,8,55,2)

IF m_glyoxylate_cycle_n8-MTH1 AND m_other_nutritional-response_activities_n10-MTH1 THEN 000 OR 001
001: 0.027041 (1644,8,55,2)

IF m_regulation_of_nitrogen_and_sulphur_utilization_n13-MTH1 AND m_cell_death_n22-MTH1 THEN 000 OR 001 OR 010 OR 100
001: 0.027041 (1644,8,55,2)

IF PDR-INO4 AND SWI5-INO4 THEN 000 OR 001
001: 0.027041 (1644,8,55,2)

IF m_anion_transporters_n17-UME6 AND m_meiosis_n3-UME6 THEN 000 OR 001
001: 0.027041 (1644,8,55,2)

IF m_g-proteins_n12-INO4 AND SWI5-INO4 AND m_pentose-phosphate_pathway_n23-INO4 THEN 000 OR 001 OR 100
001: 0.027041 (1644,8,55,2)

IF m_lipid_and_fatty-acid_transport_n11-GAT1 AND m_amino-acid_degradation_n7-GAT1 THEN 000 OR 001 OR 100 OR 111
001: 0.027041 (1644,8,55,2)

IF m_amino-acid_transport_n20-MTH1 AND m_amino-acid_transport_n20-RAP1 THEN 000 OR 001 OR 110
001: 0.027041 (1644,8,55,2)

IF m_amino-acid_transporters_n11-SWI4 THEN 000 OR 010 OR 110 OR 111
111: 0.027715 (1644,23,19,2)

IF SFF'-FKH2 AND SFF'-MCM1 THEN 000 OR 010 OR 011 OR 100 OR 110 OR 111
111: 0.027715 (1644,23,19,2)

IF RAP1-PDR1 THEN 000 OR 001 OR 010
010: 0.027773 (1644,21,140,5)

IF m_g-proteins_n12-SKN7 AND m_organization_of_chromosome_structure_n17-SKN7 THEN 000 OR 001 OR 100 OR 110 OR 111
110: 0.027847 (1644,10,115,3)

IF SFF'-RLM1 AND MCM1'-SWI4 THEN 000 OR 100 OR 110 OR 111
110: 0.027847 (1644,10,115,3)

IF SFF'-RLM1 AND SFF-FKH2 THEN 000 OR 100 OR 110 OR 111
110: 0.027847 (1644,10,115,3)

IF m_nitrogen_and_sulphur_metabolism_n16-SWI6 THEN 000 OR 101 OR 110
110: 0.027847 (1644,10,115,3)

IF SWI5-SWI6 AND LYS14-SWI6 THEN 000 OR 110
110: 0.027847 (1644,10,115,3)

IF m_morphogenesis_n5-SWI6 THEN 000 OR 010 OR 100 OR 110
110: 0.027847 (1644,10,115,3)

IF SFF'-FKH1 AND MCM1-FKH2 AND SFF-FKH2 THEN 000 OR 100 OR 110 OR 111
110: 0.027847 (1644,10,115,3)

IF SFF-HIR1 AND m_other_energy_generation_activities_n22-HIR1 THEN 000 OR 010 OR 110
110: 0.027847 (1644,10,115,3)

IF SFF'-FKH2 AND MCM1'-SWI4 THEN 000 OR 010 OR 100 OR 110
110: 0.027847 (1644,10,115,3)

IF m_lipid_and_fatty-acid_transport_n11-MTH1 AND SWI5-SWI6 THEN 000 OR 001 OR 110
110: 0.027847 (1644,10,115,3)

IF SWI5-INO4 AND m_pentose-phosphate_pathway_n23-INO4 THEN 000 OR 001 OR 100 OR 110
100: 0.029806 (1644,16,127,4)

IF m_LFTE17-SKN7 THEN 000 OR 010 OR 100 OR 110 OR 111
111: 0.030028 (1644,24,19,2)

IF mPROTEOL18(m_proteolysis_n18)-SWI6 THEN 000 OR 010 OR 100 OR 110 OR 111
111: 0.030028 (1644,24,19,2)

IF m_RPE17-RAP1 THEN 000 OR 001 OR 010 OR 100
001: 0.030753 (1644,21,55,3)

IF m_g-proteins_n12-INO4 AND SWI5-INO4 AND m_organization_of_chromosome_structure_n12-INO4 THEN 000 OR 001 OR 100
100: 0.03204 (1644,4,127,2)

IF m_biogenesis_of_cytoskeleton_n12-SFP1 THEN 000 OR 100
100: 0.03204 (1644,4,127,2)

IF m_anion_transporters_n22-RAP1 AND STRE-RAP1 THEN 000 OR 100
100: 0.03204 (1644,4,127,2)

IF m_other_morphogenetic_activities_n7-MCM1 AND m_other_morphogenetic_activities_n7-MDS3 THEN 000 OR 100
100: 0.03204 (1644,4,127,2)

IF m_ion_transporters_n4-INO4 AND m_purine_and_pyrimidine_transporters_n17-INO4 THEN 000 OR 100
100: 0.03204 (1644,4,127,2)

IF SFF'-FKH1 AND m_pentose-phosphate_pathway_n23-INO4 THEN 000 OR 100
100: 0.03204 (1644,4,127,2)

IF m_purine_and_pyrimidine_transporters_n17-GAT1 AND PHO4-CBF1 THEN 000 OR 100

100: 0.03204 (1644,4,127,2)

IF m_nitrogen_and_sulphur_utilization_n15-MAL13 THEN 000 OR 100
100: 0.03204 (1644,4,127,2)

IF m_other_transcription_activities_n8-GAT1 AND m_anion_transporters_n15-GAT1 THEN 000 OR 100 OR 110
100: 0.03204 (1644,4,127,2)

IF m_RPE52-SPT2 THEN 000 OR 100
100: 0.03204 (1644,4,127,2)

IF m_ion_transporters_n4-INO4 AND PHO4-CBF1 THEN 000 OR 100
100: 0.03204 (1644,4,127,2)

IF mPROTEOL18(m_proteolysis_n18)-UME6 AND m_pentose-phosphate_pathway_n5-UME6 THEN 000 OR 010 OR 100
100: 0.03204 (1644,4,127,2)

IF SFF'-FKH2 AND m_metal_ion_transporters_n10-SWI6 THEN 000 OR 100 OR 110
100: 0.03204 (1644,4,127,2)

IF mPROTEOL18(m_proteolysis_n18)-SWI6 AND m_other_transcription_activities_n5-SWI6 THEN 000 OR 100 OR 110
100: 0.03204 (1644,4,127,2)

IF m_g-proteins_n12-SKN7 AND m_meiosis_n3-UME6 THEN 000 OR 001 OR 100
100: 0.03204 (1644,4,127,2)

IF m_osmosensing_n6-INO4 AND m_g-proteins_n11-INO4 AND m_anion_transporters_n15-INO4 THEN 000 OR 100 OR 110
100: 0.03204 (1644,4,127,2)

IF m_c-compound_carbohydrate_transport_n11-MIG3 THEN 000 OR 100
100: 0.03204 (1644,4,127,2)

IF SFF-FKH2 AND m_metal_ion_transporters_n10-SWI6 THEN 000 OR 100 OR 110
100: 0.03204 (1644,4,127,2)

IF m_anion_transporters_n17-INO4 AND m_drug_transporters_n10-INO4 THEN 000 OR 100
100: 0.03204 (1644,4,127,2)

IF m_c-compound_carbohydrate_transport_n11-RIM101 THEN 000 OR 100
100: 0.03204 (1644,4,127,2)

IF m_osmosensing_n6-INO4 AND m_anion_transporters_n15-GAT1 THEN 000 OR 010 OR 100
100: 0.03204 (1644,4,127,2)

IF m_regulation_of_lipid_fatty-acid_and_isoprenoid_biosynthesis_n12.scn-GAT1 AND m_tricarboxylic-acid_pathway_n9-GAT1 THEN 000 OR 100
100: 0.03204 (1644,4,127,2)

IF m_anion_transporters_n17-INO4 AND m_pentose-phosphate_pathway_n23-MET4 THEN 000 OR 100
100: 0.03204 (1644,4,127,2)

IF m_phosphate_transport_n13-GAT1 AND m_pentose-phosphate_pathway_n14-GAT1 AND m_phosphate_transport_n18-GAT1 THEN 000 OR 100
100: 0.03204 (1644,4,127,2)

IF m_ion_transporters_n4-INO4 AND PHO4-INO4 THEN 000 OR 100
100: 0.03204 (1644,4,127,2)

IF m_anion_transporters_n32-INO4 AND m_regulation_of_lipid_fatty-acid_and_isoprenoid_biosynthesis_n22.scn-INO4 THEN 000 OR 100
100: 0.03204 (1644,4,127,2)

IF m_purine_and_pyrimidine_transporters_n17-INO4 AND PHO4-CBF1 THEN 000 OR 100
100: 0.03204 (1644,4,127,2)

IF m_pentose-phosphate_pathway_n23-MET4 AND m_deoxyribonucleotide_metabolism_n10-INO4 THEN 000 OR 100
100: 0.03204 (1644,4,127,2)

IF mRRPE-ABF1 AND m_regulation_of_nitrogen_and_sulphur_utilization_n7-INO4 THEN 000 OR 100
100: 0.03204 (1644,4,127,2)

IF m_organization_of_chromosome_structure_n17-SKN7 AND m_cell_death_n16-SKN7 THEN 000 OR 001 OR 100
100: 0.03204 (1644,4,127,2)

IF m_allantoin_and_allantoate_transporters_n18-HAP2 AND m_allantoin_and_allantoate_transporters_n18-YAP1 THEN 000 OR 100 OR 110
100: 0.03204 (1644,4,127,2)

IF m_phosphate_transport_n13-GAT1 AND m_pentose-phosphate_pathway_n14-GAT1 AND m_purine_and_pyrimidine_transporters_n17-GAT1 THEN 000 OR 100
100: 0.03204 (1644,4,127,2)

IF m_anion_transporters_n17-INO4 AND m_pentose-phosphate_pathway_n23-INO4 THEN 000 OR 100
100: 0.03204 (1644,4,127,2)

IF m_phosphate_transport_n18-TOS8 AND m_purine_and_pyrimidine_transporters_n17-INO4 THEN 000 OR 100
100: 0.03204 (1644,4,127,2)

IF PHO-INO4 AND m_deoxyribonucleotide_metabolism_n10-INO4 THEN 000 OR 100
100: 0.03204 (1644,4,127,2)

IF m_anion_transporters_n17-SKN7 AND m_cell_death_n16-SKN7 THEN 000 OR 001 OR 100
100: 0.03204 (1644,4,127,2)

IF m_purine_and_pyrimidine_transporters_n17-GAT1 AND m_purine_and_pyrimidine_transporters_n17-INO4 THEN 000 OR 100
100: 0.03204 (1644,4,127,2)

IF m_ion_transporters_n4-INO4 AND m_phosphate_transport_n18-XBP1 THEN 000 OR 100
100: 0.03204 (1644,4,127,2)

IF m_ion_transporters_n4-INO4 AND m_other_nutritional-response_activities_n10-INO4 AND m_other_pheromone_response_activities_n8-INO4 THEN 000 OR 100
100: 0.03204 (1644,4,127,2)

IF SFF-FKH1 AND m_pentose-phosphate_pathway_n23-INO4 THEN 000 OR 100
100: 0.03204 (1644,4,127,2)

IF STRE-SNT2 THEN 000 OR 100
100: 0.03204 (1644,4,127,2)

IF m_purine_and_pyrimidine_transporters_n17-INO4 AND m_anion_transporters_n10-INO4 THEN 000 OR 100
100: 0.03204 (1644,4,127,2)

IF m_nutritional_response_pathway_n8-INO4 AND m_phosphate_transport_n5-INO4 THEN 010 OR 100 OR 110
100: 0.03204 (1644,4,127,2)

IF m_other_mrna-transcription_activities_n20-XBP1 AND m_pentose-phosphate_pathway_n14-GAT1 THEN 000 OR 100
100: 0.03204 (1644,4,127,2)

IF m_anion_transporters_n17-INO4 AND m_organization_of_chromosome_structure_n17-INO4 THEN 000 OR 001 OR 100
100: 0.03204 (1644,4,127,2)

IF SWI5-INO4 AND m_anion_transporters_n15-INO4 AND m_cell_death_n16-INO4 THEN 000 OR 100 OR 110
100: 0.03204 (1644,4,127,2)

IF RAP1-RAP1 AND PAC-ABF1 THEN 000 OR 100
100: 0.03204 (1644,4,127,2)

IF SFF'-FKH1 AND m_allantoin_and_allantoate_transporters_n18-FKH2 AND m_other_morphogenetic_activities_n7-MCM1 THEN 010 OR 100 OR 110
100: 0.03204 (1644,4,127,2)

IF m_phosphate_transport_n18-XBP1 AND m_purine_and_pyrimidine_transporters_n17-INO4 THEN 000 OR 100
100: 0.03204 (1644,4,127,2)

IF SFF'-FKH1 AND MCM1'-NDD1 AND m_allantoin_and_allantoate_transporters_n18-FKH2 THEN 010 OR 100 OR 110
100: 0.03204 (1644,4,127,2)

IF SFF'-FKH1 AND MCM1'-MCM1 AND m_allantoin_and_allantoate_transporters_n18-FKH2 THEN 010 OR 100 OR 110
100: 0.03204 (1644,4,127,2)

IF m_other_pheromone_response_activities_n5-CBF1 THEN 000 OR 100 OR 110

100: 0.03204 (1644,4,127,2)

IF SFF'-FKH1 AND SFF'-MCM1 AND m_allantoin_and_allantoate_transporters_n18-FKH2 THEN 010 OR 100 OR 110
100: 0.03204 (1644,4,127,2)

IF MCM1'-NDD1 AND m_allantoin_and_allantoate_transporters_n18-FKH2 AND SFF-FKH2 THEN 100 OR 110
100: 0.03204 (1644,4,127,2)

IF m_anion_transporters_n17-INO4 AND m_cell_death_n16-INO4 THEN 000 OR 001 OR 100
100: 0.03204 (1644,4,127,2)

IF m_regulation_of_amino-acid_metabolism_n15-INO4 AND m_cell_death_n16-INO4 THEN 000 OR 100
100: 0.03204 (1644,4,127,2)

IF m_purine_and_pyrimidine_transporters_n17-INO4 AND PHO4-INO4 THEN 000 OR 100
100: 0.03204 (1644,4,127,2)

IF SFF'-FKH2 AND m_ion_transporters_n7-TOS8 THEN 000 OR 100
100: 0.03204 (1644,4,127,2)

IF MCM1-FKH2 AND SWI5-INO4 THEN 010 OR 100 OR 110
100: 0.03204 (1644,4,127,2)

IF SFF'-FKH2 AND m_ion_transporters_n7-XBP1 THEN 000 OR 100
100: 0.03204 (1644,4,127,2)

IF m_regulation_of_amino-acid_metabolism_n15-TBS1 AND m_other_protein-destination_activities_n7-MET4 THEN 000 OR 010 OR 100
100: 0.03204 (1644,4,127,2)

IF SFF'-FKH2 AND m_metabolism_of_cyclic_and_unusual_nucleotides_n5-YAP1 THEN 000 OR 100
100: 0.03204 (1644,4,127,2)

IF SFF'-FKH1 AND m_pentose-phosphate_pathway_n23-MET4 THEN 000 OR 100 OR 110
100: 0.03204 (1644,4,127,2)

IF SFF-FKH1 AND m_pentose-phosphate_pathway_n23-MET4 THEN 000 OR 100 OR 110
100: 0.03204 (1644,4,127,2)

IF m_phosphate_transport_n18-TOS8 AND m_phosphate_transport_n18-XBP1 AND m_purine_and_pyrimidine_transporters_n17-INO4 THEN 000 OR 100
100: 0.03204 (1644,4,127,2)

IF m_purine_and_pyrimidine_transporters_n17-GAT1 AND PHO4-INO4 THEN 000 OR 100
100: 0.03204 (1644,4,127,2)

IF SWI5-INO4 AND m_pentose-phosphate_pathway_n5-SWI4 THEN 000 OR 100
100: 0.03204 (1644,4,127,2)

IF m_ion_transporters_n4-INO4 AND m_other_pheromone_response_activities_n8-INO4 THEN 000 OR 100
100: 0.03204 (1644,4,127,2)

IF m_ion_transporters_n4-INO4 AND m_phosphate_transport_n18-INO4 AND m_other_nutritional-response_activities_n10-INO4 THEN 000 OR 100
100: 0.03204 (1644,4,127,2)

IF SFF-HIR1 AND m_organization_of_chromosome_structure_n12-HIR2 THEN 000 OR 100 OR 101
100: 0.03204 (1644,4,127,2)

IF m_ion_transporters_n4-INO4 AND m_phosphate_transport_n18-INO4 AND m_other_pheromone_response_activities_n8-INO4 THEN 000 OR 100
100: 0.03204 (1644,4,127,2)

IF SFF'-HIR1 AND m_organization_of_chromosome_structure_n12-HIR2 THEN 000 OR 100 OR 101
100: 0.03204 (1644,4,127,2)

IF RAP1-RAP1 AND PAC-REB1 THEN 000 OR 100
100: 0.03204 (1644,4,127,2)

IF m_organization_of_chromosome_structure_n17-SKN7 THEN 000 OR 001 OR 100 OR 110 OR 111
110: 0.032338 (1644,18,115,4)

IF ALPHA1'-RIM101 THEN 000 OR 001 OR 010 OR 100 OR 101 OR 110
100: 0.032368 (1644,68,127,10)

IF m_RPE68-FHL1 THEN 000 OR 001 OR 010 OR 100
001: 0.034029 (1644,9,55,2)

IF m_RPE17-FHL1 AND m_RPE21-FHL1 THEN 000 OR 001 OR 010
001: 0.034029 (1644,9,55,2)

IF m_g-proteins_n12-INO4 AND m_nutritional_response_pathway_n8-INO4 AND SWI5-INO4 THEN 000 OR 001 OR 010 OR 100
001: 0.034029 (1644,9,55,2)

IF Ume6(URS1)-UME6 AND SCB-UME6 THEN 000 OR 001 OR 010
001: 0.034029 (1644,9,55,2)

IF m_RPE17-RAP1 AND m_amino-acid_transport_n20-RAP1 THEN 000 OR 001 OR 010
001: 0.034029 (1644,9,55,2)

IF m_RPE68-PDR1 THEN 000 OR 001 OR 010 OR 100
001: 0.034029 (1644,9,55,2)

IF m_RPE17-RAP1 AND m_other_mrna-transcription_activities_n20-RAP1 THEN 000 OR 001
001: 0.034029 (1644,9,55,2)

IF m_g-proteins_n12-INO4 AND SWI5-INO4 AND m_other_mrna-transcription_activities_n20-INO4 THEN 000 OR 001 OR 010 OR 110
001: 0.034029 (1644,9,55,2)

IF m_fermentation_n4-PDR1 THEN 000 OR 001 OR 010
001: 0.034029 (1644,9,55,2)

IF m_RPE17-RAP1 AND m_RPE21-FHL1 THEN 000 OR 001 OR 010
001: 0.034029 (1644,9,55,2)

IF BAS1-GCN4 THEN 000 OR 001 OR 110
001: 0.034029 (1644,9,55,2)

IF Ume6(URS1)-UME6 AND SCB-UME6 AND m_meiosis_n3-UME6 THEN 000 OR 001 OR 010
001: 0.034029 (1644,9,55,2)

IF SWI5-SWI6 AND SCB-SWI4 THEN 000 OR 010 OR 100 OR 110 OR 111
010: 0.034647 (1644,9,140,3)

IF m_metabolism_of_energy_reserves_n30-BAS1 THEN 000 OR 010 OR 110
010: 0.034647 (1644,9,140,3)

IF m_tricarboxylic-acid_pathway_n9-SKN7 THEN 000 OR 010 OR 111
010: 0.034647 (1644,9,140,3)

IF m_trna_processing_n6-STP1 THEN 000 OR 010 OR 110
010: 0.034647 (1644,9,140,3)

IF SFF'-HIR3 AND m_other_morphogenetic_activities_n7-ARR1 THEN 000 OR 010
010: 0.034647 (1644,9,140,3)

IF SFF'-HIR3 AND SFF'-RTG3 AND SFF-ARG80 THEN 000 OR 001 OR 010 OR 110
010: 0.034647 (1644,9,140,3)

IF PDR-INO4 THEN 000 OR 001 OR 010 OR 100 OR 110
001: 0.034782 (1644,22,55,3)

IF m_other_mrna-transcription_activities_n20-XBP1 THEN 000 OR 001 OR 100 OR 110
001: 0.034782 (1644,22,55,3)

IF m_pentose-phosphate_pathway_n23-INO4 AND m_deoxyribonucleotide_metabolism_n10-INO4 THEN 000 OR 100
100: 0.036139 (1644,10,127,3)

IF SWI5-INO4 AND PHO-INO4 THEN 000 OR 010 OR 100
100: 0.036139 (1644,10,127,3)

IF SFF-FKH2 AND m_other_morphogenetic_activities_n7-MCM1 THEN 000 OR 011 OR 100 OR 110 OR 111
100: 0.036139 (1644,10,127,3)

IF SFF'-FKH2 AND m_allantoin_and_allantoate_transporters_n18-FKH2 THEN 000 OR 010 OR 100 OR 110
100: 0.036139 (1644,10,127,3)

IF SWI5-INO4 AND m_cell_death_n16-INO4 THEN 000 OR 001 OR 100 OR 110
100: 0.036139 (1644,10,127,3)

IF m_tricarboxylic-acid_pathway_n6-HAP4 THEN 000 OR 100 OR 110
100: 0.036139 (1644,10,127,3)

IF m_g-proteins_n12-INO4 AND m_cell_death_n16-INO4 THEN 000 OR 001 OR 100 OR 110
100: 0.036139 (1644,10,127,3)

IF m_OCSE15-MTH1 THEN 000 OR 010 OR 100 OR 110
110: 0.036375 (1644,11,115,3)

IF MCM1'-MCM1 AND m_nucleotide_transport_n9-MCM1 THEN 000 OR 010 OR 100 OR 101 OR 110
110: 0.036375 (1644,11,115,3)

IF SFF'-FKH2 AND SFF'-RLM1 THEN 000 OR 100 OR 110 OR 111
110: 0.036375 (1644,11,115,3)

IF m_cell_death_n16-SKN7 THEN 000 OR 001 OR 010 OR 100 OR 110 OR 111
100: 0.036706 (1644,17,127,4)

IF m_amino-acid_transporters_n11-SKN7 THEN 000 OR 001 OR 010 OR 110 OR 111
111: 0.03741 (1644,27,19,2)

IF m_other_cation_transporters_n14-RAP1 AND PHO4-CBF1 THEN 000 OR 010 OR 100
010: 0.038535 (1644,4,140,2)

IF CCA-MET4 AND m_amino-acid_transport_n18-MET4 THEN 000 OR 010 OR 100
010: 0.038535 (1644,4,140,2)

IF m_lipid_and_fatty-acid_transport_n11-GAT1 AND m_tricarboxylic-acid_pathway_n9-SKN7 THEN 000 OR 010 OR 111
010: 0.038535 (1644,4,140,2)

IF SFF-HIR1 AND RAP1-YAP5 THEN 000 OR 010
010: 0.038535 (1644,4,140,2)

IF SFF'-FKH1 AND m_cytok9-SWI6 THEN 010 OR 110 OR 111
010: 0.038535 (1644,4,140,2)

IF SFF-HIR1 AND RAP1-FHL1 THEN 000 OR 010
010: 0.038535 (1644,4,140,2)

IF m_g-proteins_n11-INO4 AND SCB-UME6 THEN 000 OR 001 OR 010
010: 0.038535 (1644,4,140,2)

IF SFF'-HIR1 AND RAP1-FHL1 THEN 000 OR 010
010: 0.038535 (1644,4,140,2)

IF SFF'-HIR1 AND RAP1-YAP5 THEN 000 OR 010
010: 0.038535 (1644,4,140,2)

IF m_nutritional_response_pathway_n7-SWI6 AND m_morphogenesis_n5-SWI6 THEN 000 OR 010 OR 110
010: 0.038535 (1644,4,140,2)

IF m_cytok9-SWI6 AND SFF-FKH1 THEN 010 OR 110 OR 111
010: 0.038535 (1644,4,140,2)

IF m_regulation_of_amino-acid_metabolism_n15-TBS1 AND m_amino-acid_transport_n18-TBS1 THEN 000 OR 010 OR 100
010: 0.038535 (1644,4,140,2)

IF ATRepeat-UME6 AND m_regulation_of_nitrogen_and_sulphur_utilization_n13-UME6 THEN 000 OR 010 OR 100
010: 0.038535 (1644,4,140,2)

IF m_lipid_and_fatty-acid_transport_n11-SKN7 AND m_glyoxylate_cycle_n11-GAT1 THEN 000 OR 010 OR 111

010: 0.038535 (1644,4,140,2)

IF SFF'-HIR3 AND RAP1-GAT3 AND RAP1-ZAP1 THEN 000 OR 010
010: 0.038535 (1644,4,140,2)

IF m_g-proteins_n12-NRG1 AND m_amino-acid_transporters_n11-CUP9 THEN 000 OR 010
010: 0.038535 (1644,4,140,2)

IF mPROTEOL18(m_proteolysis_n18)-MBP1 AND m_amino-acid_transporters_n11-UME6 THEN 000 OR 010
010: 0.038535 (1644,4,140,2)

IF SFF-HIR1 AND RAP1-FHL1 AND RAP1-RAP1 THEN 000 OR 010
010: 0.038535 (1644,4,140,2)

IF m_g-proteins_n12-NRG1 AND m_MERE11-SUT1 THEN 000 OR 010
010: 0.038535 (1644,4,140,2)

IF SFF-HIR1 AND RAP1-RAP1 AND RAP1-YAP5 THEN 000 OR 010
010: 0.038535 (1644,4,140,2)

IF RAP1-ARO80 AND RAP1-GAT3 AND RAP1-PDR1 THEN 000 OR 010
010: 0.038535 (1644,4,140,2)

IF RAP1-ARO80 AND RAP1-FHL1 AND ALPHA1-GAT3 THEN 000 OR 010
010: 0.038535 (1644,4,140,2)

IF RAP1-ARO80 AND RAP1-YAP5 AND ALPHA1-GAT3 THEN 000 OR 010
010: 0.038535 (1644,4,140,2)

IF SFF-ARG80 AND RAP1-RAP1 THEN 000 OR 010 OR 100
010: 0.038535 (1644,4,140,2)

IF AFT1-RAP1 AND RAP1-YAP5 THEN 000 OR 010
010: 0.038535 (1644,4,140,2)

IF m_other_mrna-transcription_activities_n20-INO4 AND m_glyoxylate_cycle_n11-GAT1 THEN 000 OR 010 OR 100
010: 0.038535 (1644,4,140,2)

IF AFT1-RAP1 AND ALPHA1-GAT3 THEN 000 OR 010
010: 0.038535 (1644,4,140,2)

IF SWI5-SWI6 AND SCB-UME6 THEN 000 OR 010
010: 0.038535 (1644,4,140,2)

IF ABF1-PPR1 AND ALPHA1'-YER051w THEN 000 OR 010
010: 0.038535 (1644,4,140,2)

IF m_amino-acid_degradation_n24-SUT1 AND m_phosphate_transport_n18-YAP6 THEN 000 OR 010
010: 0.038535 (1644,4,140,2)

IF m_osmosensing_n6-SWI4 AND m_amino-acid_transporters_n11-SWI4 THEN 000 OR 010 OR 110
010: 0.038535 (1644,4,140,2)

IF MCM1'-SWI4 AND SWI5-SWI6 AND SCB-SWI4 THEN 000 OR 010 OR 111
010: 0.038535 (1644,4,140,2)

IF SFF-FKH1 AND SFF-YER184C THEN 000 OR 010 OR 110
010: 0.038535 (1644,4,140,2)

IF m_g-proteins_n11-UME6 AND m_amino-acid_transport_n14-UME6 THEN 000 OR 010
010: 0.038535 (1644,4,140,2)

IF m_anion_transporters_n4-MTH1 AND m_regulation_of_nitrogen_and_sulphur_utilization_n10-MTH1 THEN 000 OR 010
010: 0.038535 (1644,4,140,2)

IF m_RRSE3-INO4 AND m_homeostasis_of_other_ions_n30-INO4 THEN 000 OR 010
010: 0.038535 (1644,4,140,2)

IF m_rRSE10-MTH1 AND m_deoxyribonucleotide_metabolism_n23-MTH1 THEN 000 OR 010
010: 0.038535 (1644,4,140,2)

IF SFF-FKH1 AND ALPHA1'-UPC2 AND ALPHA1'-YER051w THEN 000 OR 010 OR 110
010: 0.038535 (1644,4,140,2)

IF SFF'-FKH1 AND SFF-YER184C THEN 000 OR 010 OR 110
010: 0.038535 (1644,4,140,2)

IF SFF'-FKH1 AND ALPHA1'-KSS1 AND ALPHA1'-RIM101 THEN 000 OR 010 OR 100
010: 0.038535 (1644,4,140,2)

IF m_glyoxylate_cycle_n8-INO4 AND m_RPE57-SKN7 THEN 000 OR 010
010: 0.038535 (1644,4,140,2)

IF m_MERE4-SKN7 AND SWI5-SKN7 AND m_RPE57-SKN7 THEN 000 OR 010
010: 0.038535 (1644,4,140,2)

IF m_g-proteins_n12-INO4 AND m_RRSE3-INO4 AND m_homeostasis_of_other_ions_n30-INO4 THEN 000 OR 010
010: 0.038535 (1644,4,140,2)

IF m_osmosensing_n6-INO4 AND m_glyoxylate_cycle_n8-INO4 AND m_homeostasis_of_other_ions_n30-INO4 THEN 000 OR 010
010: 0.038535 (1644,4,140,2)

IF m_lipid_and_fatty-acid_transport_n11-GAT1 AND m_lipid_and_fatty-acid_transport_n11-SKN7 AND m_other_mrna-transcription_activities_n20-INO4 THEN 000 OR 010 OR 110
010: 0.038535 (1644,4,140,2)

IF m_lipid_and_fatty-acid_transport_n11-GAT1 AND m_other_mrna-transcription_activities_n20-INO4 AND m_other_mrna-transcription_activities_n20-SKN7 THEN 000 OR 010 OR 110
010: 0.038535 (1644,4,140,2)

IF m_other_mrna-transcription_activities_n20-INO4 AND SCB-SWI4 THEN 000 OR 010
010: 0.038535 (1644,4,140,2)

IF m_osmosensing_n6-INO4 AND m_ion_transporters_n4-INO4 AND m_homeostasis_of_other_ions_n30-INO4 THEN 000 OR 010
010: 0.038535 (1644,4,140,2)

IF SFF'-FKH1 AND ALPHA1'-RIM101 AND ALPHA1'-UPC2 THEN 000 OR 010 OR 100
010: 0.038535 (1644,4,140,2)

IF SFF-ARG80 AND m_other_morphogenetic_activities_n7-GCR2 AND ALPHA1'-STB4 THEN 000 OR 001 OR 010
010: 0.038535 (1644,4,140,2)

IF m_lipid_and_fatty-acid_transport_n11-SKN7 AND m_tricarboxylic-acid_pathway_n9-GAT1 THEN 000 OR 010 OR 111
010: 0.038535 (1644,4,140,2)

IF SFF-ARG80 AND m_other_morphogenetic_activities_n7-HOG1 THEN 000 OR 010 OR 110
010: 0.038535 (1644,4,140,2)

IF m_other_morphogenetic_activities_n7-GCR2 AND ALPHA1'-KSS1 THEN 000 OR 010
010: 0.038535 (1644,4,140,2)

IF m_tricarboxylic-acid_pathway_n9-SKN7 AND m_glyoxylate_cycle_n11-GAT1 THEN 000 OR 010 OR 111
010: 0.038535 (1644,4,140,2)

IF SFF'-HIR3 AND m_other_morphogenetic_activities_n7-HOG1 THEN 000 OR 010
010: 0.038535 (1644,4,140,2)

IF m_g-proteins_n11-HAL9 AND ECB-YOX1 THEN 000 OR 010 OR 110
010: 0.038535 (1644,4,140,2)

IF m_g-proteins_n11-INO4 AND ABF1-ABF1 THEN 000 OR 010
010: 0.038535 (1644,4,140,2)

IF SFF'-HIR3 AND ECB-YOX1 THEN 000 OR 010 OR 110
010: 0.038535 (1644,4,140,2)

IF m_g-proteins_n12-NRG1 AND SCB-SWI4 THEN 000 OR 010
010: 0.038535 (1644,4,140,2)

IF RAP1-HMS1 AND RAP1-RAP1 THEN 000 OR 010

010: 0.038535 (1644,4,140,2)

IF m_g-proteins_n12-INO4 AND SWI5-SWI6 AND SCB-SWI4 THEN 000 OR 010 OR 110
010: 0.038535 (1644,4,140,2)

IF m_g-proteins_n12-SKN7 AND SWI5-SWI6 AND SCB-SWI4 THEN 000 OR 010 OR 110
010: 0.038535 (1644,4,140,2)

IF m_other_morphogenetic_activities_n7-RCS1 AND RAP1-FHL1 THEN 000 OR 010
010: 0.038535 (1644,4,140,2)

IF ECB-MCM1 AND m_other_morphogenetic_activities_n7-MCM1 AND m_nucleotide_transport_n9-MCM1 THEN 010 OR 110
010: 0.038535 (1644,4,140,2)

IF MCM1-MCM1 AND m_other_morphogenetic_activities_n7-MCM1 AND m_nucleotide_transport_n9-MCM1 THEN 010 OR 110
010: 0.038535 (1644,4,140,2)

IF MCM1'-PDC2 AND SFF-ARG80 THEN 000 OR 001 OR 010
010: 0.038535 (1644,4,140,2)

IF m_deoxyribonucleotide_metabolism_n27-MTH1 AND m_ion_transporters_n3-MTH1 THEN 000 OR 010
010: 0.038535 (1644,4,140,2)

IF RAP1-ARG81 AND RAP1-ZAP1 THEN 000 OR 010
010: 0.038535 (1644,4,140,2)

IF m_regulation_of_nitrogen_and_sulphur_utilization_n10-MTH1 AND m_deoxyribonucleotide_metabolism_n23-MTH1 THEN 000 OR 010
010: 0.038535 (1644,4,140,2)

IF m_regulation_of_lipid_fatty-acid_and_isoprenoid_biosynthesis_n8.scn-MTH1 AND m_cell_death_n22-MTH1 THEN 000 OR 010
010: 0.038535 (1644,4,140,2)

IF m_g-proteins_n12-SKN7 AND SWI5-SKN7 AND SCB-AZF1 THEN 010 OR 111
010: 0.038535 (1644,4,140,2)

IF SFF'-HIR1 AND SFF'-HIR3 AND m_other_morphogenetic_activities_n7-ARR1 THEN 000 OR 010
010: 0.038535 (1644,4,140,2)

IF m_lipid_and_fatty-acid_binding_n15-FHL1 AND ALPHA1-GAT3 THEN 000 OR 010
010: 0.038535 (1644,4,140,2)

IF m_g-proteins_n12-INO4 AND m_nutritional_response_pathway_n7-SWI6 THEN 000 OR 010 OR 110
010: 0.038535 (1644,4,140,2)

IF m_g-proteins_n12-INO4 AND m_regulation_of_lipid_fatty-acid_and_isoprenoid_biosynthesis_n22.scn-INO4 AND m_anion_transporters_n10-INO4 THEN 000 OR 010
010: 0.038535 (1644,4,140,2)

IF m_regulation_of_lipid_fatty-acid_and_isoprenoid_biosynthesis_n8.scn-MTH1 AND m_stress_response_n17-MTH1 THEN 000 OR 010
010: 0.038535 (1644,4,140,2)

IF m_other_morphogenetic_activities_n7-RCS1 AND RAP1-FHL1 AND RAP1-RAP1 THEN 000 OR 010
010: 0.038535 (1644,4,140,2)

IF SFF'-HIR1 AND SFF-HIR1 AND RAP1-FHL1 THEN 000 OR 010
010: 0.038535 (1644,4,140,2)

IF SFF'-HIR1 AND RAP1-FHL1 AND RAP1-RAP1 THEN 000 OR 010
010: 0.038535 (1644,4,140,2)

IF m_anion_transporters_n16-SWI5 THEN 000 OR 010 OR 110
010: 0.038535 (1644,4,140,2)

IF m_anion_transporters_n19-MTH1 AND m_regulation_of_lipid_fatty-acid_and_isoprenoid_biosynthesis_n8.scn-MTH1 THEN 000 OR 010
010: 0.038535 (1644,4,140,2)

IF m_regulation_of_nitrogen_and_sulphur_utilization_n10-MTH1 AND m_regulation_of_lipid_fatty-acid_and_isoprenoid_biosynthesis_n8.scn-MTH1 THEN 000 OR 010
010: 0.038535 (1644,4,140,2)

IF m_other_morphogenetic_activities_n7-ARR1 AND m_other_morphogenetic_activities_n7-STP1 THEN 000 OR 010 OR 110
010: 0.038535 (1644,4,140,2)

IF RAP1-ARO80 AND ALPHA1-GAT3 THEN 000 OR 010
010: 0.038535 (1644,4,140,2)

IF RAP1-ARO80 AND RAP1-GAT3 THEN 000 OR 010
010: 0.038535 (1644,4,140,2)

IF m_lipid_and_fatty-acid_binding_n13-ARG81 THEN 000 OR 010 OR 110
010: 0.038535 (1644,4,140,2)

IF m_MERE4-MTH1 AND m_deoxyribonucleotide_metabolism_n27-MTH1 THEN 000 OR 010
010: 0.038535 (1644,4,140,2)

IF SFF'-HIR3 AND RAP1-HAP4 THEN 000 OR 010
010: 0.038535 (1644,4,140,2)

IF m_other_mrna-transcription_activities_n11-SWI6 AND mPROTEOL18(m_proteolysis_n18)-SWI6 THEN 000 OR 010
010: 0.038535 (1644,4,140,2)

IF AFT1-RAP1 AND m_regulation_of_nitrogen_and_sulphur_utilization_n7-RAP1 THEN 000 OR 010
010: 0.038535 (1644,4,140,2)

IF m_anion_transporters_n22-RAP1 AND m_nitrogen_and_sulphur_transport_n9-FHL1 THEN 000 OR 010 OR 110
010: 0.038535 (1644,4,140,2)

IF RAP1-ARO80 AND RAP1-HAP4 THEN 000 OR 010
010: 0.038535 (1644,4,140,2)

IF m_cell_death_n15-CRZ1 THEN 000 OR 010
010: 0.038535 (1644,4,140,2)

IF m_mitochondrial_biogenesis_n5-MAL13 AND m_cell_death_n22-MAL13 THEN 000 OR 010
010: 0.038535 (1644,4,140,2)

IF RAP1-ARG81 AND RAP1-YAP5 THEN 000 OR 010
010: 0.038535 (1644,4,140,2)

IF RAP1-HIR2 THEN 000 OR 010
010: 0.038535 (1644,4,140,2)

IF m_RPE11-RAP1 AND m_regulation_of_amino-acid_metabolism_n7-RAP1 THEN 000 OR 010
010: 0.038535 (1644,4,140,2)

IF m_allantoin_and_allantoate_transporters_n7-ACE2 THEN 000 OR 010 OR 100 OR 110 OR 111
110: 0.038809 (1644,19,115,4)

IF MCM1'-PDC2 THEN 000 OR 001 OR 010 OR 100 OR 110
010: 0.040871 (1644,16,140,4)

IF SFF-HOG1 THEN 000 OR 001 OR 010 OR 100 OR 110
110: 0.041185 (1644,28,115,5)

IF m_RPE21-SMP1 THEN 000 OR 001 OR 010
001: 0.041636 (1644,10,55,2)

IF m_biogenesis_of_chromosome_structure_n9-YBR239c THEN 000 OR 001 OR 010 OR 100
001: 0.041636 (1644,10,55,2)

IF SFF-GAL3 AND SFF-RPI1 THEN 000 OR 001 OR 110
001: 0.041636 (1644,10,55,2)

IF m_RPE21-FHL1 AND m_RPE21-SMP1 THEN 000 OR 001 OR 010
001: 0.041636 (1644,10,55,2)

IF m_other_nucleotide-metabolism_activities_n17-WAR1 THEN 000 OR 001 OR 100 OR 110
001: 0.041636 (1644,10,55,2)

IF m_g-proteins_n12-INO4 AND SWI5-INO4 AND m_organization_of_chromosome_structure_n17-INO4 THEN 000 OR 001 OR 100 OR 110

001: 0.041636 (1644,10,55,2)

IF m_other_mrna-transcription_activities_n20-INO4 AND m_other_mrna-transcription_activities_n20-RAP1 THEN 000 OR 001 OR 100 OR 110
001: 0.041636 (1644,10,55,2)

IF m_glycolysis_and_gluconeogenesis_n11-YER130C THEN 000 OR 001 OR 100
001: 0.041636 (1644,10,55,2)

IF m_other_energy_generation_activities_n4-UME6 AND Ume6(URS1)-UME6 THEN 000 OR 001 OR 010 OR 100
001: 0.041636 (1644,10,55,2)

IF m_g-proteins_n12-INO4 AND m_osmosensing_n6-SWI4 THEN 000 OR 010 OR 100 OR 110
110: 0.042191 (1644,5,115,2)

IF m_other_mrna-transcription_activities_n20-INO4 AND m_other_transport_facilitators_n5-INO4 THEN 000 OR 010 OR 110
110: 0.042191 (1644,5,115,2)

IF SFF'-MCM1 AND m_other_cell_growth_cell_division_and_dna_synthesis_activities_n10.scn-NDD1 THEN 000 OR 010 OR 110
110: 0.042191 (1644,5,115,2)

IF m_organization_of_intracellular_transport_vesicles_n5-SWI4 AND MCM1-SWI4 THEN 000 OR 010 OR 110
110: 0.042191 (1644,5,115,2)

IF SFF'-FKH2 AND MCM1'-NDD1 AND MCM1'-SWI4 AND SFF-FKH2 THEN 000 OR 100 OR 110
110: 0.042191 (1644,5,115,2)

IF SFF-HIR1 AND m_other_morphogenetic_activities_n7-HOG1 AND m_other_morphogenetic_activities_n7-RCS1 THEN 000 OR 110
110: 0.042191 (1644,5,115,2)

IF m_glyoxylate_cycle_n19-SWI4 THEN 000 OR 110
110: 0.042191 (1644,5,115,2)

IF SFF-FKH2 AND mPROTEOL18(m_proteolysis_n18)-MBP1 THEN 000 OR 110
110: 0.042191 (1644,5,115,2)

IF m_osmosensing_n6-INO4 AND SFF-FKH2 THEN 000 OR 100 OR 110
110: 0.042191 (1644,5,115,2)

IF SWI5-SWI6 AND m_other_mrna-transcription_activities_n20-INO4 THEN 000 OR 010 OR 100 OR 110
110: 0.042191 (1644,5,115,2)

IF m_g-proteins_n12-INO4 AND m_g-proteins_n11-INO4 AND m_glyoxylate_cycle_n8-INO4 THEN 000 OR 110
110: 0.042191 (1644,5,115,2)

IF m_cell_death_n22-SWI4 AND m_cell_death_n22-TOS8 THEN 000 OR 100 OR 110
110: 0.042191 (1644,5,115,2)

IF SFF'-FKH1 AND m_allantoin_and_allantoate_transporters_n13-SWI6 THEN 000 OR 110 OR 111
110: 0.042191 (1644,5,115,2)

IF SFF-FKH1 AND m_allantoin_and_allantoate_transporters_n13-SWI6 THEN 000 OR 110 OR 111
110: 0.042191 (1644,5,115,2)

IF SFF'-FKH1 AND ndt80(MSE)-YDR049W THEN 000 OR 100 OR 110
110: 0.042191 (1644,5,115,2)

IF SFF'-FKH1 AND ndt80(MSE)-YER130C THEN 000 OR 100 OR 110
110: 0.042191 (1644,5,115,2)

IF SWI5-SWI6 AND m_other_energy_generation_activities_n16-SKN7 THEN 000 OR 010 OR 110
110: 0.042191 (1644,5,115,2)

IF MCM1'-MCM1 AND ALPHA1'-UPC2 THEN 000 OR 110
110: 0.042191 (1644,5,115,2)

IF SFF'-FKH2 AND mPROTEOL18(m_proteolysis_n18)-SWI6 THEN 000 OR 010 OR 110
110: 0.042191 (1644,5,115,2)

IF MCM1-FKH2 AND MCM1'-NDD1 AND MCM1'-SWI4 THEN 000 OR 100 OR 110
110: 0.042191 (1644,5,115,2)

IF SFF'-FKH2 AND ECB-YOX1 THEN 000 OR 010 OR 110 OR 111
110: 0.042191 (1644,5,115,2)

IF MCM1-SWI4 AND MCM1'-NDD1 THEN 000 OR 100 OR 110
110: 0.042191 (1644,5,115,2)

IF ECB-FKH2 AND ALPHA1'-UPC2 THEN 000 OR 110
110: 0.042191 (1644,5,115,2)

IF m_g-proteins_n12-SKN7 AND m_amino-acid_degradation_n7-NDD1 THEN 000 OR 110 OR 111
110: 0.042191 (1644,5,115,2)

IF m_other_morphogenetic_activities_n7-HOG1 AND m_other_morphogenetic_activities_n7-RTG1 THEN 000 OR 110
110: 0.042191 (1644,5,115,2)

IF SFF-FKH1 AND m_glyoxylate_cycle_n11-GAT1 THEN 000 OR 010 OR 100 OR 110
110: 0.042191 (1644,5,115,2)

IF MCM1-MCM1 AND ECB-MCM1 AND m_nucleotide_transport_n9-MCM1 THEN 010 OR 100 OR 110
110: 0.042191 (1644,5,115,2)

IF m_other_energy_generation_activities_n12-FKH1 AND ALPHA1'-UPC2 THEN 000 OR 010 OR 100 OR 110
110: 0.042191 (1644,5,115,2)

IF SFF-FKH2 AND m_allantoin_and_allantoate_transporters_n13-SWI6 THEN 000 OR 110 OR 111
110: 0.042191 (1644,5,115,2)

IF SFF'-FKH2 AND m_phosphate_transport_n8-SWI4 THEN 000 OR 110
110: 0.042191 (1644,5,115,2)

IF m_other_transport_facilitators_n5-CIN5 AND m_other_transport_facilitators_n5-INO4 THEN 000 OR 100 OR 110
110: 0.042191 (1644,5,115,2)

IF SFF'-FKH2 AND m_allantoin_and_allantoate_transporters_n13-SWI6 THEN 000 OR 110 OR 111
110: 0.042191 (1644,5,115,2)

IF MCM1-FKH2 AND MCM1-SWI4 AND MCM1'-NDD1 THEN 000 OR 100 OR 110
110: 0.042191 (1644,5,115,2)

IF m_LFTE17-GAT1 AND m_pentose-phosphate_pathway_n7-GAT1 THEN 000 OR 110
110: 0.042191 (1644,5,115,2)

IF SFF'-FKH1 AND RPN4-GAT1 THEN 000 OR 110
110: 0.042191 (1644,5,115,2)

IF SFF'-RLM1 AND MCM1-SWI4 THEN 000 OR 110
110: 0.042191 (1644,5,115,2)

IF SFF'-MCM1 AND ECB-STE12 THEN 000 OR 010 OR 100 OR 110
110: 0.042191 (1644,5,115,2)

IF m_regulation_of_nitrogen_and_sulphur_utilization_n13-MTH1 AND m_LFTE17-GAT1 THEN 000 OR 001 OR 100 OR 110
110: 0.042191 (1644,5,115,2)

IF SFF'-FKH1 AND SFF'-FKH2 AND m_g-proteins_n12-INO4 THEN 000 OR 110
110: 0.042191 (1644,5,115,2)

IF m_glycolysis_and_gluconeogenesis_n11-MBP1 THEN 000 OR 110 OR 111
110: 0.042191 (1644,5,115,2)

IF SFF'-RLM1 AND mPROTEOL18(m_proteolysis_n18)-MBP1 THEN 000 OR 110 OR 111
110: 0.042191 (1644,5,115,2)

IF SFF'-RLM1 AND mPROTEOL18(m_proteolysis_n18)-SWI6 THEN 000 OR 110 OR 111
110: 0.042191 (1644,5,115,2)

IF SFF'-FKH2 AND MCM1-NDD1 AND MCM1-SWI4 THEN 000 OR 100 OR 110
110: 0.042191 (1644,5,115,2)

IF SFF'-FKH1 AND m_osmosensing_n6-SWI4 THEN 000 OR 100 OR 110 OR 111
110: 0.042191 (1644,5,115,2)

IF SFF'-FKH1 AND SCB-UME6 THEN 000 OR 110
110: 0.042191 (1644,5,115,2)

IF SFF'-MCM1 AND m_osmosensing_n6-SWI4 THEN 100 OR 101 OR 110 OR 111
110: 0.042191 (1644,5,115,2)

IF LYS14-SWI6 AND m_pentose-phosphate_pathway_n14-FKH1 THEN 000 OR 110
110: 0.042191 (1644,5,115,2)

IF MCM1-SWI4 AND MCM1'-NDD1 AND MCM1'-SWI4 THEN 000 OR 100 OR 110
110: 0.042191 (1644,5,115,2)

IF SFF-FKH2 AND SCB-UME6 THEN 000 OR 110
110: 0.042191 (1644,5,115,2)

IF MCM1-NDD1 AND MCM1-SWI4 AND MCM1'-NDD1 THEN 000 OR 100 OR 110
110: 0.042191 (1644,5,115,2)

IF MCM1-NDD1 AND MCM1-SWI4 AND MCM1'-SWI4 THEN 000 OR 100 OR 110
110: 0.042191 (1644,5,115,2)

IF MCM1-NDD1 AND MCM1'-NDD1 AND MCM1'-SWI4 THEN 000 OR 100 OR 110
110: 0.042191 (1644,5,115,2)

IF m_organization_of_intracellular_transport_vesicles_n5-SWI4 AND MCM1-SWI4 AND MCM1'-SWI4 THEN 000 OR 010 OR 110
110: 0.042191 (1644,5,115,2)

IF m_breakdown_of_lipids_fatty_acids_and_isoprenoids_n8-MBP1 AND SFF-FKH2 THEN 000 OR 110 OR 111
110: 0.042191 (1644,5,115,2)

IF SFF'-HIR1 AND m_other_morphogenetic_activities_n7-HOG1 AND m_other_morphogenetic_activities_n7-RCS1 THEN 000 OR 110
110: 0.042191 (1644,5,115,2)

IF MCM1'-MCM1 AND PHO-NDD1 THEN 000 OR 010 OR 110
110: 0.042191 (1644,5,115,2)

IF m_breakdown_of_lipids_fatty_acids_and_isoprenoids_n8-MBP1 AND SFF'-FKH2 THEN 000 OR 110 OR 111
110: 0.042191 (1644,5,115,2)

IF SFF'-FKH1 AND m_allantoin_and_allantoate_transporters_n18-FKH2 THEN 010 OR 100 OR 110
110: 0.042191 (1644,5,115,2)

IF SFF'-MCM1 AND PHO-NDD1 THEN 000 OR 010 OR 110
110: 0.042191 (1644,5,115,2)

IF m_cytok9-SWI6 AND m_other_pheromone_response_activities_n8-SWI6 THEN 000 OR 010 OR 110
110: 0.042191 (1644,5,115,2)

IF MCM1'-NDD1 AND PHO-NDD1 THEN 000 OR 010 OR 110
110: 0.042191 (1644,5,115,2)

IF SFF'-FKH2 AND m_phosphate_transport_n18-ASH1 THEN 000 OR 010 OR 100 OR 110
110: 0.042191 (1644,5,115,2)

IF mPROTEOL18(m_proteolysis_n18)-SWI6 AND m_nucleotide_transport_n9-SWI6 THEN 000 OR 100 OR 110
110: 0.042191 (1644,5,115,2)

IF MCM1-MCM1 AND PHO-NDD1 THEN 000 OR 010 OR 110
110: 0.042191 (1644,5,115,2)

IF SFF'-FKH1 AND m_regulation_of_lipid_fatty-acid_and_isoprenoid_biosynthesis_n8.scn-GAT1 THEN 000 OR 010 OR 100 OR 110
110: 0.042191 (1644,5,115,2)

IF m_osmosensing_n6-INO4 AND SWI5-SWI6 THEN 000 OR 100 OR 110
110: 0.042191 (1644,5,115,2)

IF m_regulation_of_lipid_fatty-acid_and_isoprenoid_biosynthesis_n8.scn-FKH1 AND m_regulation_of_lipid_fatty-acid_and_isoprenoid_biosynthesis_n8.scn-GAT1 THEN 000 OR 010 OR 100 OR 110
110: 0.042191 (1644,5,115,2)

IF MCM1-MCM1 AND MCM1'-MCM1 AND m_nucleotide_transport_n9-MCM1 THEN 010 OR 100 OR 110
110: 0.042191 (1644,5,115,2)

IF MCM1-NDD1 AND PHO-NDD1 THEN 000 OR 010 OR 110
110: 0.042191 (1644,5,115,2)

IF m_breakdown_of_lipids_fatty_acids_and_isoprenoids_n8-INO4 AND m_g-proteins_n11-INO4 THEN 000 OR 110
110: 0.042191 (1644,5,115,2)

IF SFF-FKH2 AND m_allantoin_and_allantoate_transporters_n12-ACE2 THEN 000 OR 001 OR 110
110: 0.042191 (1644,5,115,2)

IF m_MERE4-NDD1 AND m_other_protein-destination_activities_n7-XBP1 THEN 000 OR 110
110: 0.042191 (1644,5,115,2)

IF m_trna_transcription_n10-FKH1 THEN 000 OR 010 OR 110
110: 0.042191 (1644,5,115,2)

IF m_nutritional_response_pathway_n7-SWI6 AND m_other_pheromone_response_activities_n8-SWI6 THEN 000 OR 010 OR 110 OR 111
110: 0.042191 (1644,5,115,2)

IF MCM1-NDD1 AND MCM1'-SWI4 THEN 000 OR 100 OR 110
110: 0.042191 (1644,5,115,2)

IF MCM1-MCM1 AND ECB-STE12 THEN 000 OR 010 OR 100 OR 110
110: 0.042191 (1644,5,115,2)

IF m_osmosensing_n6-INO4 AND m_other_mrna-transcription_activities_n20-SKN7 THEN 000 OR 010 OR 100 OR 110
110: 0.042191 (1644,5,115,2)

IF MCM1'-SWI4 AND mRRPE-RLM1 THEN 000 OR 110
110: 0.042191 (1644,5,115,2)

IF m_nucleotide_transport_n9-MCM1 AND m_nucleotide_transport_n9-SWI6 THEN 000 OR 100 OR 101 OR 110
110: 0.042191 (1644,5,115,2)

IF m_g-proteins_n12-SKN7 AND m_amino-acid_degradation_n7-NDD1 AND m_amino-acid_degradation_n7-SKN7 THEN 000 OR 110 OR 111
110: 0.042191 (1644,5,115,2)

IF SFF'-RLM1 AND MCM1'-NDD1 AND MCM1'-SWI4 THEN 000 OR 110
110: 0.042191 (1644,5,115,2)

IF ECB-MCM1 AND PHO-NDD1 THEN 000 OR 010 OR 110
110: 0.042191 (1644,5,115,2)

IF m_peroxisomal_organization_n8-SWI6 AND SWI5-SWI6 THEN 000 OR 110
110: 0.042191 (1644,5,115,2)

IF ECB-NDD1 AND PHO-NDD1 THEN 000 OR 010 OR 110
110: 0.042191 (1644,5,115,2)

IF SWI5-SWI6 AND m_other_transport_facilitators_n5-SWI6 THEN 000 OR 010 OR 110
110: 0.042191 (1644,5,115,2)

IF SFF-FKH1 AND m_metal_ion_transporters_n17-FKH2 THEN 000 OR 010 OR 110
110: 0.042191 (1644,5,115,2)

IF SFF'-FKH1 AND m_metal_ion_transporters_n17-FKH2 THEN 000 OR 010 OR 110
110: 0.042191 (1644,5,115,2)

IF SFF'-FKH2 AND SFF-FKH2 AND mPROTEOL18(m_proteolysis_n18)-MBP1 THEN 000 OR 110
110: 0.042191 (1644,5,115,2)

IF m_glyoxylate_cycle_n8-MTH1 AND m_phosphate_transport_n13-SKN7 THEN 000 OR 010 OR 110
110: 0.042191 (1644,5,115,2)

IF m_pentose-phosphate_pathway_n21-GAT1 AND m_pentose-phosphate_pathway_n14-GAT1 THEN 000 OR 110
110: 0.042191 (1644,5,115,2)

IF m_g-proteins_n12-SKN7 AND m_osmosensing_n6-INO4 THEN 000 OR 010 OR 110
110: 0.042191 (1644,5,115,2)

IF SFF'-FKH2 AND m_allantoin_and_allantoate_transporters_n12-ACE2 THEN 000 OR 001 OR 110
110: 0.042191 (1644,5,115,2)

IF SWI5-INO4 AND m_pentose-phosphate_pathway_n14-MTH1 THEN 000 OR 110
110: 0.042191 (1644,5,115,2)

IF HAP234-HAP2 THEN 000 OR 110
110: 0.042191 (1644,5,115,2)

IF SFF'-FKH1 AND m_rRSE10-GAT1 THEN 000 OR 100 OR 110
110: 0.042191 (1644,5,115,2)

IF SFF-FKH1 AND m_rRSE10-GAT1 THEN 000 OR 100 OR 110
110: 0.042191 (1644,5,115,2)

IF SWI5-SWI6 AND mRRPE-RLM1 THEN 000 OR 110
110: 0.042191 (1644,5,115,2)

IF m_MERE4-MTH1 AND m_pentose-phosphate_pathway_n14-GAT1 THEN 000 OR 010 OR 100 OR 110
110: 0.042191 (1644,5,115,2)

IF PHO4-INO4 THEN 000 OR 001 OR 010 OR 100
100: 0.04445 (1644,18,127,4)

IF m_g-proteins_n12-INO4 AND m_osmosensing_n6-INO4 THEN 000 OR 010 OR 100 OR 101 OR 110
110: 0.045969 (1644,20,115,4)

IF m_other_signal-transduction_activities_n8-INO4 THEN 000 OR 001 OR 100 OR 110
110: 0.045969 (1644,20,115,4)

IF m_osmosensing_n6-INO4 AND SWI5-INO4 THEN 000 OR 010 OR 100 OR 110
110: 0.046083 (1644,12,115,3)

IF m_lipid_and_fatty-acid_transport_n11-GAT1 AND m_lipid_and_fatty-acid_transport_n11-MTH1 THEN 000 OR 001 OR 100 OR 110 OR 111
110: 0.046083 (1644,12,115,3)

IF m_g-proteins_n12-INO4 AND m_g-proteins_n12-SKN7 AND SWI5-INO4 THEN 000 OR 001 OR 010 OR 100 OR 110
110: 0.046083 (1644,12,115,3)

IF m_g-proteins_n12-INO4 AND SWI5-SKN7 THEN 000 OR 001 OR 010 OR 100 OR 110
110: 0.046083 (1644,12,115,3)

IF m_g-proteins_n12-INO4 AND SWI5-INO4 AND SWI5-SKN7 THEN 000 OR 001 OR 010 OR 100 OR 110
110: 0.046083 (1644,12,115,3)

IF m_g-proteins_n12-SKN7 AND SWI5-INO4 THEN 000 OR 001 OR 010 OR 100 OR 110
110: 0.046083 (1644,12,115,3)

IF MET31-32-MET32 THEN 000 OR 010 OR 100
010: 0.046475 (1644,10,140,3)

IF m_g-proteins_n12-INO4 AND SCB-SWI4 THEN 000 OR 010 OR 110 OR 111
010: 0.046475 (1644,10,140,3)

IF m_amino-acid_transporters_n11-SWI4 AND SCB-SWI4 THEN 000 OR 010 OR 110 OR 111
010: 0.046475 (1644,10,140,3)

IF SFF-ARG80 AND m_other_morphogenetic_activities_n7-GCR2 THEN 000 OR 001 OR 010 OR 110
010: 0.046475 (1644,10,140,3)

IF m_other_cell_growth_cell_division_and_dna_synthesis_activities_n10.scn-ASK10 THEN 000 OR 010 OR 110
010: 0.046475 (1644,10,140,3)

IF m_stress_response_n17-MTH1 THEN 000 OR 010

010: 0.046475 (1644,10,140,3)

IF MCM1'-SWI4 AND m_pentose-phosphate_pathway_n5-SWI4 THEN 000 OR 100 OR 101 OR 110 OR 111
100: 0.046948 (1644,11,127,3)

IF m_organization_of_chromosome_structure_n12-INO4 THEN 000 OR 001 OR 100 OR 110
100: 0.046948 (1644,11,127,3)

IF m_anion_transporters_n27-MTH1 THEN 000 OR 100 OR 110
100: 0.046948 (1644,11,127,3)

IF ABF1-ABF1 AND PAC-ABF1 THEN 000 OR 100
100: 0.046948 (1644,11,127,3)

IF ALPHA1-DAT1 THEN 000 OR 010 OR 100 OR 110
010: 0.047201 (1644,24,140,5)

IF m_other_nutritional-response_activities_n10-MTH1 THEN 000 OR 001 OR 010 OR 100 OR 110
001: 0.048419 (1644,25,55,3)

IF m_ion_transporters_n3-INO4 THEN 000 OR 001 OR 100 OR 110
001: 0.049815 (1644,11,55,2)

IF m_g-proteins_n12-INO4 AND m_g-proteins_n11-INO4 AND SWI5-INO4 THEN 000 OR 001 OR 010 OR 100 OR 110
001: 0.049815 (1644,11,55,2)

IF m_RPE32-RAP1 THEN 000 OR 001 OR 100 OR 101
001: 0.049815 (1644,11,55,2)

IF m_other_energy_generation_activities_n4-UME6 AND m_meiosis_n3-UME6 THEN 000 OR 001 OR 010 OR 100
001: 0.049815 (1644,11,55,2)

IF SFF-FKH2 AND m_anion_transporters_n15-INO4 THEN 000 OR 100
100: 0.050724 (1644,5,127,2)

IF SFF'-FKH2 AND m_anion_transporters_n15-INO4 THEN 000 OR 100
100: 0.050724 (1644,5,127,2)

IF m_regulation_of_nitrogen_and_sulphur_utilization_n7-INO4 AND m_cell_death_n16-INO4 THEN 000 OR 100
100: 0.050724 (1644,5,127,2)

IF m_anion_transporters_n17-INO4 AND m_deoxyribonucleotide_metabolism_n10-INO4 THEN 000 OR 001 OR 100
100: 0.050724 (1644,5,127,2)

IF m_phosphate_transport_n18-INO4 AND m_other_nutritional-response_activities_n10-INO4 AND m_other_pheromone_response_activities_n8-INO4 AND m_cell_death_n16-INO4 THEN 000 OR 100 OR 111
100: 0.050724 (1644,5,127,2)

IF PHO-INO4 AND m_pentose-phosphate_pathway_n23-INO4 THEN 000 OR 100
100: 0.050724 (1644,5,127,2)

IF SFF'-HIR3 AND SFF-FKH2 THEN 000 OR 010 OR 100 OR 110
100: 0.050724 (1644,5,127,2)

IF SFF'-FKH2 AND SFF'-HIR3 THEN 000 OR 010 OR 100 OR 110
100: 0.050724 (1644,5,127,2)

IF SFF'-FKH1 AND m_metal_ion_transporters_n10-SWI6 THEN 000 OR 100 OR 110
100: 0.050724 (1644,5,127,2)

IF m_amino-acid_degradation_n27-HIR2 THEN 000 OR 100 OR 110
100: 0.050724 (1644,5,127,2)

IF PDR-SUT1 AND m_MERE11-SUT1 THEN 000 OR 100
100: 0.050724 (1644,5,127,2)

IF m_allantoin_and_allantoate_transporters_n18-FKH2 AND m_other_morphogenetic_activities_n7-MCM1 THEN 010 OR 100 OR 110
100: 0.050724 (1644,5,127,2)

IF SFF-FKH2 AND ABF1-ABF1 THEN 000 OR 100 OR 110

100: 0.050724 (1644,5,127,2)

IF SFF'-MCM1 AND m_allantoin_and_allantoate_transporters_n18-FKH2 THEN 010 OR 100 OR 110
100: 0.050724 (1644,5,127,2)

IF MCM1'-MCM1 AND m_allantoin_and_allantoate_transporters_n18-FKH2 THEN 010 OR 100 OR 110
100: 0.050724 (1644,5,127,2)

IF SFF'-FKH1 AND m_allantoin_and_allantoate_transporters_n18-FKH2 THEN 010 OR 100 OR 110
100: 0.050724 (1644,5,127,2)

IF m_pentose-phosphate_pathway_n14-GAT1 AND m_phosphate_transport_n18-GAT1 AND m_purine_and_pyrimidine_transporters_n17-GAT1 THEN 000 OR 100
100: 0.050724 (1644,5,127,2)

IF CSRE-INO4 AND PHO4-INO4 AND m_cell_death_n16-INO4 THEN 000 OR 100
100: 0.050724 (1644,5,127,2)

IF m_phosphate_transport_n18-INO4 AND m_other_nutritional-response_activities_n10-INO4 AND m_other_pheromone_response_activities_n8-INO4 THEN 000 OR 100 OR 111
100: 0.050724 (1644,5,127,2)

IF m_other_nutritional-response_activities_n10-INO4 AND m_other_pheromone_response_activities_n8-INO4 AND m_cell_death_n16-INO4 THEN 000 OR 100 OR 111
100: 0.050724 (1644,5,127,2)

IF m_ion_transporters_n4-INO4 AND m_phosphate_transport_n18-INO4 AND m_phosphate_transport_n18-TOS8 THEN 000 OR 100
100: 0.050724 (1644,5,127,2)

IF m_biogenesis_of_chromosome_structure_n9-RAP1 AND m_biogenesis_of_chromosome_structure_n9-REB1 THEN 000 OR 100
100: 0.050724 (1644,5,127,2)

IF SFF'-FKH1 AND m_cell_death_n16-SKN7 THEN 000 OR 100
100: 0.050724 (1644,5,127,2)

IF SFF-FKH1 AND m_cell_death_n16-SKN7 THEN 000 OR 100
100: 0.050724 (1644,5,127,2)

IF SFF'-SIP3 AND ALPHA1'-RIM101 THEN 000 OR 100
100: 0.050724 (1644,5,127,2)

IF m_nutritional_response_pathway_n8-INO4 AND PHO-INO4 THEN 000 OR 100
100: 0.050724 (1644,5,127,2)

IF m_regulation_of_lipid_fatty-acid_and_isoprenoid_biosynthesis_n16.scn-NDD1 AND SFF-FKH2 THEN 000 OR 100
100: 0.050724 (1644,5,127,2)

IF m_anion_transporters_n15-MTH1 AND m_nutritional_response_pathway_n12-MTH1 THEN 000 OR 100
100: 0.050724 (1644,5,127,2)

IF m_organization_of_chromosome_structure_n17-INO4 AND m_cell_death_n16-INO4 THEN 000 OR 001 OR 100
100: 0.050724 (1644,5,127,2)

IF m_pheromone_response_generation_n4-SKN7 THEN 000 OR 100 OR 110
100: 0.050724 (1644,5,127,2)

IF m_other_mrna-transcription_activities_n20-INO4 AND m_pentose-phosphate_pathway_n14-GAT1 THEN 000 OR 100 OR 110
100: 0.050724 (1644,5,127,2)

IF m_anion_transporters_n4-INO4 AND m_purine_and_pyrimidine_transporters_n17-INO4 THEN 000 OR 100
100: 0.050724 (1644,5,127,2)

IF SFF-SIP3 AND ALPHA1'-RIM101 THEN 000 OR 100
100: 0.050724 (1644,5,127,2)

IF m_ion_transporters_n4-INO4 AND m_cell_death_n16-INO4 THEN 000 OR 100
100: 0.050724 (1644,5,127,2)

IF m_ion_transporters_n4-INO4 AND m_phosphate_transport_n18-TOS8 THEN 000 OR 100
100: 0.050724 (1644,5,127,2)

IF MCM1'-NDD1 AND SWI5-SWI6 AND m_pentose-phosphate_pathway_n5-SWI4 THEN 000 OR 100
100: 0.050724 (1644,5,127,2)

IF m_anion_transporters_n4-INO4 AND PHO-INO4 THEN 000 OR 100
100: 0.050724 (1644,5,127,2)

IF m_rRSE10-GAT1 AND m_tricarboxylic-acid_pathway_n9-GAT1 THEN 000 OR 100
100: 0.050724 (1644,5,127,2)

IF m_regulation_of_lipid_fatty-acid_and_isoprenoid_biosynthesis_n12.scn-GAT1 AND m_rRSE10-GAT1 THEN 000 OR 100
100: 0.050724 (1644,5,127,2)

IF m_g-proteins_n12-SKN7 AND m_LFTE17-UME6 THEN 000 OR 100
100: 0.050724 (1644,5,127,2)

IF m_LFTE17-SKN7 AND m_LFTE17-UME6 THEN 000 OR 100
100: 0.050724 (1644,5,127,2)

IF m_g-proteins_n12-INO4 AND m_phosphate_transport_n5-INO4 THEN 010 OR 100 OR 110
100: 0.050724 (1644,5,127,2)

IF m_pentose-phosphate_pathway_n14-GAT1 AND m_purine_and_pyrimidine_transporters_n17-GAT1 THEN 000 OR 100
100: 0.050724 (1644,5,127,2)

IF m_other_protein-destination_activities_n7-SKN7 AND m_cell_death_n16-SKN7 THEN 000 OR 100
100: 0.050724 (1644,5,127,2)

IF m_phosphate_transport_n18-INO4 AND m_organization_of_chromosome_structure_n12-INO4 THEN 000 OR 100 OR 110
100: 0.050724 (1644,5,127,2)

IF MCM1'-SWI4 AND SWI5-INO4 THEN 000 OR 010 OR 100
100: 0.050724 (1644,5,127,2)

IF m_other_nutritional-response_activities_n10-INO4 AND m_cell_death_n16-INO4 THEN 000 OR 100 OR 111
100: 0.050724 (1644,5,127,2)

IF MCM1'-SWI4 AND m_g-proteins_n11-INO4 THEN 000 OR 010 OR 100
100: 0.050724 (1644,5,127,2)

IF m_phosphate_transport_n18-GAT1 AND m_phosphate_transport_n18-INO4 THEN 000 OR 100
100: 0.050724 (1644,5,127,2)

IF SFF-FKH1 AND m_metal_ion_transporters_n10-SWI6 THEN 000 OR 100 OR 110
100: 0.050724 (1644,5,127,2)

IF m_phosphate_transport_n18-GAT1 AND m_phosphate_transport_n18-TOS8 THEN 000 OR 001 OR 100
100: 0.050724 (1644,5,127,2)

IF m_ion_transporters_n4-GAT1 AND m_phosphate_transport_n18-GAT1 AND m_purine_and_pyrimidine_transporters_n17-GAT1 THEN 000 OR 100
100: 0.050724 (1644,5,127,2)

IF m_ion_transporters_n4-GAT1 AND m_ion_transporters_n4-INO4 THEN 000 OR 010 OR 100
100: 0.050724 (1644,5,127,2)

IF ATRepeat-UME6 AND m_pentose-phosphate_pathway_n5-UME6 THEN 000 OR 010 OR 100
100: 0.050724 (1644,5,127,2)

IF PDR-UME6 AND m_glyoxylate_cycle_n11-UME6 THEN 000 OR 100
100: 0.050724 (1644,5,127,2)

IF m_amino-acid_transport_n3-SKN7 AND m_regulation_of_lipid_fatty-acid_and_isoprenoid_biosynthesis_n16.scn-SKN7 THEN 000 OR 100
100: 0.050724 (1644,5,127,2)

IF SFF'-FKH1 AND m_regulation_of_lipid_fatty-acid_and_isoprenoid_biosynthesis_n16.scn-SKN7 THEN 000 OR 100
100: 0.050724 (1644,5,127,2)

IF m_other_morphogenetic_activities_n7-MCM1 AND m_other_morphogenetic_activities_n7-RTG1 THEN 000 OR 011 OR 100
100: 0.050724 (1644,5,127,2)

IF m_utilization_of_vitamins_cofactors_and_prosthetic_groups_n7-DAL81 THEN 000 OR 010 OR 100
100: 0.050724 (1644,5,127,2)

IF m_ion_transporters_n4-GAT1 AND m_anion_transporters_n10-INO4 THEN 000 OR 010 OR 100
100: 0.050724 (1644,5,127,2)

IF SFF'-FKH1 AND m_cell_death_n16-INO4 THEN 000 OR 100
100: 0.050724 (1644,5,127,2)

IF SFF-FKH1 AND m_cell_death_n16-INO4 THEN 000 OR 100
100: 0.050724 (1644,5,127,2)

IF m_deoxyribonucleotide_metabolism_n23-MTH1 AND m_pentose-phosphate_pathway_n7-MTH1 THEN 000 OR 010 OR 100
100: 0.050724 (1644,5,127,2)

IF CSRE-INO4 AND PHO4-INO4 THEN 000 OR 100
100: 0.050724 (1644,5,127,2)

IF PAC-ABF1 THEN 000 OR 100 OR 110
100: 0.051668 (1644,27,127,5)

IF m_pentose-phosphate_pathway_n23-MET4 THEN 000 OR 010 OR 100 OR 110
100: 0.053041 (1644,19,127,4)

IF m_other_morphogenetic_activities_n7-MCM1 THEN 000 OR 001 OR 010 OR 011 OR 100 OR 101 OR 110 OR 111
110: 0.055269 (1644,50,115,7)

IF m_nutritional_response_pathway_n8-INO4 AND SWI5-INO4 THEN 000 OR 001 OR 010 OR 100 OR 110
001: 0.058519 (1644,12,55,2)

IF m_homeostasis_of_metal_ions_n20-ACE2 THEN 000 OR 001 OR 100
001: 0.058519 (1644,12,55,2)

IF m_RPE21-RGM1 THEN 000 OR 001 OR 010
001: 0.058519 (1644,12,55,2)

IF m_other_energy_generation_activities_n16-INO4 THEN 000 OR 001 OR 010 OR 110
001: 0.058519 (1644,12,55,2)

IF m_g-proteins_n12-INO4 AND m_pentose-phosphate_pathway_n23-INO4 THEN 000 OR 001 OR 010 OR 100
001: 0.058519 (1644,12,55,2)

IF m_fermentation_n3-GAT1 THEN 000 OR 001 OR 101
001: 0.058519 (1644,12,55,2)

IF m_pentose-phosphate_pathway_n23-RAP1 THEN 000 OR 001 OR 010 OR 100 OR 110
001: 0.058766 (1644,27,55,3)

IF m_anion_transporters_n15-INO4 THEN 000 OR 001 OR 010 OR 100 OR 110 OR 111
100: 0.059145 (1644,28,127,5)

IF SFF'-FKH2 AND m_other_morphogenetic_activities_n7-MCM1 THEN 000 OR 010 OR 011 OR 100 OR 110 OR 111
100: 0.059156 (1644,12,127,3)

IF SFF-FKH1 AND SWI5-INO4 THEN 000 OR 010 OR 100 OR 110
100: 0.059156 (1644,12,127,3)

IF m_phosphate_transport_n5-INO4 THEN 000 OR 001 OR 010 OR 100 OR 110
100: 0.059156 (1644,12,127,3)

IF m_other_nucleotide-metabolism_activities_n17-YPR022C THEN 000 OR 001 OR 010 OR 100 OR 110
100: 0.059156 (1644,12,127,3)

IF SFF'-FKH1 AND SFF-FKH1 AND SWI5-INO4 THEN 000 OR 010 OR 100 OR 110
100: 0.059156 (1644,12,127,3)

IF ABF1-RTS2 THEN 000 OR 010 OR 100
100: 0.059156 (1644,12,127,3)

IF m_other_nucleotide-metabolism_activities_n17-YGR067C THEN 000 OR 001 OR 010 OR 100 OR 110
100: 0.059156 (1644,12,127,3)

IF SFF'-FKH1 AND ALPHA1'-RIM101 THEN 000 OR 010 OR 100 OR 110
010: 0.060019 (1644,11,140,3)

IF m_other_cation_transporters_n14-MET32 THEN 000 OR 010
010: 0.060019 (1644,11,140,3)

IF m_g-proteins_n12-INO4 AND SWI5-INO4 AND SWI5-SWI6 THEN 000 OR 010 OR 100 OR 110
010: 0.060019 (1644,11,140,3)

IF m_g-proteins_n12-INO4 AND SWI5-SWI6 THEN 000 OR 010 OR 100 OR 110
010: 0.060019 (1644,11,140,3)

IF m_mitochondrial_biogenesis_n5-MAL13 THEN 000 OR 010 OR 110
010: 0.060019 (1644,11,140,3)

IF SFF-FKH1 AND ALPHA1'-UPC2 THEN 000 OR 010 OR 110
010: 0.060019 (1644,11,140,3)

IF m_other_transcription_activities_n5-SWI6 THEN 000 OR 010 OR 100 OR 101 OR 110
010: 0.060019 (1644,11,140,3)

IF m_g-proteins_n12-SKN7 AND SWI5-SKN7 THEN 000 OR 001 OR 010 OR 100 OR 110 OR 111
111: 0.06004 (1644,35,19,2)

IF m_g-proteins_n12-INO4 AND m_g-proteins_n11-INO4 AND m_other_mrna-transcription_activities_n20-INO4 THEN 000 OR 001 OR 010 OR 110
110: 0.060433 (1644,6,115,2)

IF m_g-proteins_n12-SKN7 AND m_glyoxylate_cycle_n8-INO4 THEN 000 OR 010 OR 110 OR 111
110: 0.060433 (1644,6,115,2)

IF m_glyoxylate_cycle_n8-INO4 AND m_homeostasis_of_other_ions_n30-INO4 THEN 000 OR 010 OR 110
110: 0.060433 (1644,6,115,2)

IF SFF-HOG1 AND m_other_morphogenetic_activities_n7-MDS3 THEN 000 OR 010 OR 110
110: 0.060433 (1644,6,115,2)

IF m_regulation_of_nitrogen_and_sulphur_utilization_n13-MTH1 AND m_pentose-phosphate_pathway_n7-MTH1 THEN 000 OR 010 OR 110
110: 0.060433 (1644,6,115,2)

IF LYS14-SWI6 AND m_deoxyribonucleotide_metabolism_n10-SWI6 THEN 000 OR 110
110: 0.060433 (1644,6,115,2)

IF SWI5-SWI6 AND m_amino-acid_transporters_n11-SKN7 AND m_amino-acid_transporters_n11-SWI4 THEN 000 OR 010 OR 110
110: 0.060433 (1644,6,115,2)

IF SFF-FKH2 AND m_amino-acid_degradation_n8-NDD1 THEN 000 OR 110
110: 0.060433 (1644,6,115,2)

IF m_g-proteins_n12-INO4 AND MCM1'-NDD1 AND SWI5-INO4 THEN 000 OR 010 OR 100 OR 110
110: 0.060433 (1644,6,115,2)

IF SWI5-SKN7 AND SWI5-SWI6 AND m_amino-acid_transporters_n11-SWI4 THEN 000 OR 010 OR 110
110: 0.060433 (1644,6,115,2)

IF SFF'-FKH2 AND m_regulation_of_lipid_fatty-acid_and_isoprenoid_biosynthesis_n8.scn-FKH1 THEN 000 OR 100 OR 110
110: 0.060433 (1644,6,115,2)

IF SFF'-FKH2 AND m_g-proteins_n12-INO4 AND SWI5-INO4 THEN 000 OR 010 OR 110
110: 0.060433 (1644,6,115,2)

IF SFF-ARG80 AND ALPHA1'-YER051w THEN 000 OR 010 OR 110
110: 0.060433 (1644,6,115,2)

IF m_osmosensing_n6-INO4 AND SWI5-SKN7 THEN 000 OR 010 OR 100 OR 110
110: 0.060433 (1644,6,115,2)

IF SFF-FKH2 AND SWI5-INO4 THEN 000 OR 100 OR 110
110: 0.060433 (1644,6,115,2)

IF SFF-FKH2 AND m_regulation_of_lipid_fatty-acid_and_isoprenoid_biosynthesis_n8.scn-FKH1 THEN 000 OR 100 OR 110
110: 0.060433 (1644,6,115,2)

IF m_other_proteolytic_degradation_n5-PDR1 AND ALPHA1-RGM1 THEN 000 OR 010 OR 110
110: 0.060433 (1644,6,115,2)

IF m_lipid_and_fatty-acid_transport_n11-GAT1 AND m_lipid_and_fatty-acid_transport_n11-MTH1 AND SWI5-SWI6 THEN 000 OR 001 OR 110
110: 0.060433 (1644,6,115,2)

IF m_osmosensing_n6-SWI4 AND SFF-FKH2 THEN 000 OR 100 OR 110 OR 111
110: 0.060433 (1644,6,115,2)

IF m_lipid_and_fatty-acid_transport_n11-MTH1 AND m_anion_transporters_n20-MTH1 THEN 000 OR 110 OR 111
110: 0.060433 (1644,6,115,2)

IF m_other_mrna-transcription_activities_n20-INO4 AND m_other_mrna-transcription_activities_n20-XBP1 THEN 000 OR 001 OR 100 OR 110
110: 0.060433 (1644,6,115,2)

IF SFF'-FKH2 AND MCM1'-NDD1 AND MCM1'-SWI4 THEN 000 OR 100 OR 110
110: 0.060433 (1644,6,115,2)

IF SWI5-SWI6 AND m_nitrogen_and_sulphur_metabolism_n16-SWI6 THEN 000 OR 101 OR 110
110: 0.060433 (1644,6,115,2)

IF m_g-proteins_n11-INO4 AND SWI5-SWI6 THEN 000 OR 010 OR 100 OR 110
110: 0.060433 (1644,6,115,2)

IF SFF-FKH1 AND m_lipid_and_fatty-acid_transport_n11-MTH1 THEN 000 OR 110
110: 0.060433 (1644,6,115,2)

IF m_osmosensing_n6-SWI4 AND MCM1'-SWI4 THEN 000 OR 010 OR 100 OR 101 OR 110
110: 0.060433 (1644,6,115,2)

IF MCM1-FKH2 AND MCM1'-SWI4 THEN 000 OR 010 OR 100 OR 110
110: 0.060433 (1644,6,115,2)

IF m_nutritional_response_pathway_n7-ACE2 AND m_nutritional_response_pathway_n7-SWI6 THEN 000 OR 110
110: 0.060433 (1644,6,115,2)

IF SWI5-SKN7 AND m_amino-acid_transporters_n11-SKN7 AND m_amino-acid_transporters_n11-SWI4 THEN 000 OR 010 OR 110
110: 0.060433 (1644,6,115,2)

IF m_lipid_and_fatty-acid_transport_n11-MTH1 AND m_LFTE17-GAT1 THEN 000 OR 001 OR 100 OR 110 OR 111
110: 0.060433 (1644,6,115,2)

IF m_cell_death_n15-RLM1 THEN 000 OR 101 OR 110
110: 0.060433 (1644,6,115,2)

IF SFF-HOG1 AND m_other_morphogenetic_activities_n7-RCS1 THEN 000 OR 100 OR 110
110: 0.060433 (1644,6,115,2)

IF SWI5-SKN7 AND m_amino-acid_transporters_n11-SWI4 THEN 000 OR 010 OR 110
110: 0.060433 (1644,6,115,2)

IF SFF'-FKH1 AND m_glyoxylate_cycle_n11-GAT1 THEN 000 OR 010 OR 100 OR 110
110: 0.060433 (1644,6,115,2)

IF m_g-proteins_n11-INO4 AND m_lipid_and_fatty-acid_transport_n11-SKN7 THEN 000 OR 010 OR 110
110: 0.060433 (1644,6,115,2)

IF SFF'-RLM1 AND m_metabolism_of_cyclic_and_unusual_nucleotides_n5-NDD1 THEN 000 OR 110 OR 111
110: 0.060433 (1644,6,115,2)

IF m_lipid_and_fatty-acid_transport_n11-GAT1 AND m_lipid_and_fatty-acid_transport_n11-MTH1 AND m_LFTE17-GAT1 THEN 000 OR 001 OR 100 OR 110 OR 111
110: 0.060433 (1644,6,115,2)

IF m_g-proteins_n12-IME4 AND m_g-proteins_n12-NRG1 THEN 000 OR 001 OR 110
110: 0.060433 (1644,6,115,2)

IF m_other_morphogenetic_activities_n7-GCR2 AND m_other_morphogenetic_activities_n7-HOG1 THEN 000 OR 010 OR 110
110: 0.060433 (1644,6,115,2)

IF SFF'-FKH1 AND SFF-FKH1 AND m_lipid_and_fatty-acid_transport_n11-MTH1 THEN 000 OR 110
110: 0.060433 (1644,6,115,2)

IF mPROTEOL18(m_proteolysis_n18)-MBP1 AND STRE-SWI6 THEN 000 OR 010 OR 110
110: 0.060433 (1644,6,115,2)

IF SWI5-INO4 AND m_organization_of_plasma_membrane_n17-INO4 THEN 000 OR 110
110: 0.060433 (1644,6,115,2)

IF m_other_energy_generation_activities_n17-TEC1 THEN 000 OR 001 OR 100 OR 110
110: 0.060433 (1644,6,115,2)

IF SFF'-RTG3 AND ECB-YOX1 THEN 000 OR 010 OR 110
110: 0.060433 (1644,6,115,2)

IF SFF-HOG1 AND m_other_morphogenetic_activities_n7-GCR2 THEN 000 OR 010 OR 110
110: 0.060433 (1644,6,115,2)

IF m_other_signal-transduction_activities_n8-RAP1 AND m_other_mrna-transcription_activities_n20-RAP1 THEN 000 OR 001 OR 110
110: 0.060433 (1644,6,115,2)

IF m_cytok9-SWI6 AND m_nutritional_response_pathway_n7-SWI6 THEN 000 OR 010 OR 110
110: 0.060433 (1644,6,115,2)

IF m_g-proteins_n12-SKN7 AND m_other_energy_generation_activities_n16-SKN7 THEN 000 OR 010 OR 110
110: 0.060433 (1644,6,115,2)

IF ATRepeat-UME6 AND SCB-UME6 THEN 000 OR 100 OR 110
110: 0.060433 (1644,6,115,2)

IF SFF'-FKH2 AND mPROTEOL18(m_proteolysis_n18)-MBP1 THEN 000 OR 010 OR 110
110: 0.060433 (1644,6,115,2)

IF m_osmosensing_n6-INO4 AND m_other_mrna-transcription_activities_n20-INO4 THEN 000 OR 010 OR 100 OR 110
110: 0.060433 (1644,6,115,2)

IF m_other_mrna-transcription_activities_n20-INO4 AND m_homeostasis_of_other_ions_n30-INO4 THEN 000 OR 001 OR 010 OR 110
110: 0.060433 (1644,6,115,2)

IF SFF'-FKH2 AND m_other_energy_generation_activities_n12-FKH1 THEN 000 OR 010 OR 110
110: 0.060433 (1644,6,115,2)

IF SFF'-FKH2 AND MCM1-SWI4 THEN 000 OR 010 OR 100 OR 110
110: 0.060433 (1644,6,115,2)

IF SWI5-SWI6 AND m_other_pheromone_response_activities_n8-SWI6 THEN 000 OR 001 OR 110
110: 0.060433 (1644,6,115,2)

IF m_g-proteins_n12-INO4 AND m_g-proteins_n12-SKN7 AND m_glyoxylate_cycle_n8-INO4 THEN 000 OR 010 OR 110 OR 111
110: 0.060433 (1644,6,115,2)

IF SWI5-SWI6 AND m_deoxyribonucleotide_metabolism_n23-MTH1 THEN 000 OR 110
110: 0.060433 (1644,6,115,2)

IF SFF'-FKH1 AND m_lipid_and_fatty-acid_transport_n11-MTH1 THEN 000 OR 110
110: 0.060433 (1644,6,115,2)

IF SFF'-FKH2 AND m_amino-acid_degradation_n8-NDD1 THEN 000 OR 110
110: 0.060433 (1644,6,115,2)

IF m_rRSE10-MTH1 AND m_regulation_of_lipid_fatty-acid_and_isoprenoid_biosynthesis_n8.scn-MTH1 THEN 000 OR 010 OR 110
010: 0.060677 (1644,5,140,2)

IF RAP1-FHL1 AND RAP1-RAP1 AND m_nitrogen_and_sulphur_transport_n9-FHL1 AND ALPHA1-GAT3 THEN 000 OR 010

010: 0.060677 (1644,5,140,2)

IF m_rRSE10-PDR1 AND RAP1-RAP1 THEN 000 OR 010
010: 0.060677 (1644,5,140,2)

IF m_rRSE10-PDR1 AND RAP1-GAT3 THEN 000 OR 010
010: 0.060677 (1644,5,140,2)

IF m_anion_transporters_n19-MTH1 AND m_deoxyribonucleotide_metabolism_n23-MTH1 THEN 000 OR 010 OR 110
010: 0.060677 (1644,5,140,2)

IF m_other_mrna-transcription_activities_n20-SKN7 AND m_glyoxylate_cycle_n11-GAT1 THEN 000 OR 010 OR 100
010: 0.060677 (1644,5,140,2)

IF m_other_mrna-transcription_activities_n11-SWI6 AND SFF-FKH1 THEN 000 OR 010 OR 100 OR 110
010: 0.060677 (1644,5,140,2)

IF m_nutritional_response_pathway_n7-SWI6 AND m_other_transport_facilitators_n10-SWI6 THEN 000 OR 010 OR 110
010: 0.060677 (1644,5,140,2)

IF SFF'-FKH1 AND m_other_mrna-transcription_activities_n11-SWI6 THEN 000 OR 010 OR 100 OR 110
010: 0.060677 (1644,5,140,2)

IF m_other_transport_facilitators_n10-SWI6 AND m_other_pheromone_response_activities_n8-SWI6 THEN 000 OR 010 OR 110
010: 0.060677 (1644,5,140,2)

IF m_regulation_of_lipid_fatty-acid_and_isoprenoid_biosynthesis_n22.scn-AFT2 THEN 000 OR 010
010: 0.060677 (1644,5,140,2)

IF STE12-THI2 THEN 000 OR 010
010: 0.060677 (1644,5,140,2)

IF m_lipid_and_fatty-acid_transport_n11-GAT1 AND m_other_mrna-transcription_activities_n20-INO4 THEN 000 OR 010 OR 110
010: 0.060677 (1644,5,140,2)

IF SFF-FKH1 AND ALPHA1'-YER051w THEN 000 OR 010 OR 110
010: 0.060677 (1644,5,140,2)

IF RAP1-PDR1 AND RAP1-RAP1 AND m_nitrogen_and_sulphur_transport_n9-FHL1 THEN 000 OR 010
010: 0.060677 (1644,5,140,2)

IF SFF'-HIR3 AND RAP1-FHL1 AND RAP1-RGM1 THEN 000 OR 010
010: 0.060677 (1644,5,140,2)

IF RAP1-RAP1 AND RAP1-ZAP1 AND ALPHA1-GAT3 THEN 000 OR 010
010: 0.060677 (1644,5,140,2)

IF SFF'-HIR3 AND RAP1-RAP1 AND ALPHA1-RGM1 THEN 000 OR 010
010: 0.060677 (1644,5,140,2)

IF m_anion_transporters_n22-RAP1 AND RAP1-GAT3 THEN 000 OR 010
010: 0.060677 (1644,5,140,2)

IF SFF'-HIR3 AND RAP1-PDR1 AND RAP1-ZAP1 THEN 000 OR 010
010: 0.060677 (1644,5,140,2)

IF SFF'-HIR3 AND RAP1-RGM1 AND RAP1-YAP5 THEN 000 OR 010
010: 0.060677 (1644,5,140,2)

IF RAP1-GAT3 AND RAP1-ZAP1 THEN 000 OR 010
010: 0.060677 (1644,5,140,2)

IF SFF'-RTG3 AND m_g-proteins_n11-HAL9 THEN 000 OR 010 OR 110
010: 0.060677 (1644,5,140,2)

IF RAP1-GAT3 AND RAP1-RAP1 AND m_nitrogen_and_sulphur_transport_n9-FHL1 THEN 000 OR 010
010: 0.060677 (1644,5,140,2)

IF SFF'-HIR3 AND RAP1-RGM1 THEN 000 OR 010
010: 0.060677 (1644,5,140,2)

IF SWI5-INO4 AND SCB-SWI4 THEN 000 OR 010 OR 110
010: 0.060677 (1644,5,140,2)

IF m_anion_transporters_n22-RAP1 AND ALPHA1-GAT3 THEN 000 OR 010
010: 0.060677 (1644,5,140,2)

IF m_lipid_and_fatty-acid_transport_n7-SKN7 AND m_RPE57-SKN7 THEN 000 OR 010
010: 0.060677 (1644,5,140,2)

IF SFF'-HIR3 AND RAP1-RGM1 AND ALPHA1-GAT3 THEN 000 OR 010
010: 0.060677 (1644,5,140,2)

IF SFF'-FKH1 AND m_amino-acid_transport_n18-SKN7 THEN 000 OR 010 OR 100
010: 0.060677 (1644,5,140,2)

IF SFF'-HIR3 AND RAP1-GAT3 AND RAP1-RGM1 THEN 000 OR 010
010: 0.060677 (1644,5,140,2)

IF RAP1-ZAP1 AND ALPHA1-GAT3 THEN 000 OR 010
010: 0.060677 (1644,5,140,2)

IF MCM1-MCM1 AND ECB-MCM1 AND m_nucleotide_transport_n9-MCM1 THEN 010 OR 100 OR 110
010: 0.060677 (1644,5,140,2)

IF MCM1-MCM1 AND MCM1'-MCM1 AND m_nucleotide_transport_n9-MCM1 THEN 010 OR 100 OR 110
010: 0.060677 (1644,5,140,2)

IF SFF'-HIR3 AND RAP1-PDR1 AND RAP1-YAP5 AND ALPHA1-RGM1 THEN 000 OR 010
010: 0.060677 (1644,5,140,2)

IF SFF-RTG3 AND m_other_morphogenetic_activities_n7-GCR2 THEN 000 OR 010
010: 0.060677 (1644,5,140,2)

IF m_utilization_of_vitamins_cofactors_and_prosthetic_groups_n7-DAL81 THEN 000 OR 010 OR 100
010: 0.060677 (1644,5,140,2)

IF SWI5-SKN7 AND m_glycolysis_and_gluconeogenesis_n4-INO4 THEN 000 OR 010 OR 110
010: 0.060677 (1644,5,140,2)

IF SFF'-FKH1 AND SCB-AZF1 THEN 000 OR 010 OR 110 OR 111
010: 0.060677 (1644,5,140,2)

IF RAP1-HMS1 THEN 000 OR 010
010: 0.060677 (1644,5,140,2)

IF ALPHA1'-RIM101 AND ALPHA1'-UPC2 AND ALPHA1'-YER051w THEN 000 OR 010 OR 100
010: 0.060677 (1644,5,140,2)

IF m_tricarboxylic-acid_pathway_n9-GAT1 AND m_tricarboxylic-acid_pathway_n9-SKN7 THEN 000 OR 010 OR 111
010: 0.060677 (1644,5,140,2)

IF SFF'-MCM1 AND ECB-MCM1 AND m_nucleotide_transport_n9-MCM1 THEN 010 OR 110
010: 0.060677 (1644,5,140,2)

IF SFF-FKH1 AND ALPHA1'-KSS1 THEN 000 OR 010 OR 100 OR 110
010: 0.060677 (1644,5,140,2)

IF SFF'-FKH1 AND ALPHA1'-KSS1 THEN 000 OR 010 OR 100 OR 110
010: 0.060677 (1644,5,140,2)

IF SFF'-HIR3 AND ALPHA1-RGM1 THEN 000 OR 010
010: 0.060677 (1644,5,140,2)

IF SFF'-HIR3 AND RAP1-PDR1 AND ALPHA1-RGM1 THEN 000 OR 010
010: 0.060677 (1644,5,140,2)

IF RAP1-ARG81 AND RAP1-FHL1 THEN 000 OR 010
010: 0.060677 (1644,5,140,2)

IF m_lipid_and_fatty-acid_binding_n15-FHL1 THEN 000 OR 010
010: 0.060677 (1644,5,140,2)

IF m_other_morphogenetic_activities_n7-RCS1 AND RAP1-RAP1 THEN 000 OR 010
010: 0.060677 (1644,5,140,2)

IF SFF-ARG80 AND SFF-HOG1 THEN 000 OR 001 OR 010 OR 110
010: 0.060677 (1644,5,140,2)

IF MCM1'-SWI4 AND m_g-proteins_n11-INO4 THEN 000 OR 010 OR 100
010: 0.060677 (1644,5,140,2)

IF SFF'-HIR3 AND RAP1-ZAP1 THEN 000 OR 010
010: 0.060677 (1644,5,140,2)

IF m_polynucleotide_degradation_n3-MSN2 THEN 000 OR 010
010: 0.060677 (1644,5,140,2)

IF m_g-proteins_n12-INO4 AND m_glyoxylate_cycle_n8-INO4 AND m_homeostasis_of_other_ions_n30-INO4 THEN 000 OR 010 OR 110
010: 0.060677 (1644,5,140,2)

IF SFF'-HIR3 AND RAP1-FHL1 AND RAP1-PDR1 AND RAP1-ZAP1 THEN 000 OR 010
010: 0.060677 (1644,5,140,2)

IF mRRPE-ABF1 AND ALPHA1'-RIM101 THEN 000 OR 010
010: 0.060677 (1644,5,140,2)

IF m_g-proteins_n12-INO4 AND m_ion_transporters_n4-INO4 AND m_homeostasis_of_other_ions_n30-INO4 THEN 000 OR 010
010: 0.060677 (1644,5,140,2)

IF m_osmosensing_n6-INO4 AND m_glyoxylate_cycle_n8-INO4 AND m_RRSE3-INO4 THEN 000 OR 010 OR 110
010: 0.060677 (1644,5,140,2)

IF RAP1-RAP1 AND m_nitrogen_and_sulphur_transport_n9-FHL1 AND ALPHA1-GAT3 THEN 000 OR 010
010: 0.060677 (1644,5,140,2)

IF RAP1-GAT3 AND m_nitrogen_and_sulphur_transport_n9-FHL1 THEN 000 OR 010
010: 0.060677 (1644,5,140,2)

IF RAP1-PDR1 AND m_nitrogen_and_sulphur_transport_n9-FHL1 THEN 000 OR 010
010: 0.060677 (1644,5,140,2)

IF RAP1-YAP5 AND m_nitrogen_and_sulphur_transport_n9-FHL1 THEN 000 OR 010
010: 0.060677 (1644,5,140,2)

IF m_regulation_of_lipid_fatty-acid_and_isoprenoid_biosynthesis_n16.scn-NDD1 AND PHO-NDD1 THEN 000 OR 010 OR 110
010: 0.060677 (1644,5,140,2)

IF SFF-HIR1 AND RAP1-RAP1 THEN 000 OR 010
010: 0.060677 (1644,5,140,2)

IF RAP1-FHL1 AND RAP1-YAP5 AND m_nitrogen_and_sulphur_transport_n9-FHL1 AND ALPHA1-GAT3 THEN 000 OR 010
010: 0.060677 (1644,5,140,2)

IF ALPHA1-DAT1 AND ALPHA1-RGM1 THEN 000 OR 010 OR 110
010: 0.060677 (1644,5,140,2)

IF m_g-proteins_n12-INO4 AND m_RRSE3-INO4 AND m_ion_transporters_n4-INO4 THEN 000 OR 010
010: 0.060677 (1644,5,140,2)

IF SFF'-HIR1 AND RAP1-RAP1 THEN 000 OR 010
010: 0.060677 (1644,5,140,2)

IF m_other_morphogenetic_activities_n8-SNF1 THEN 000 OR 010
010: 0.060677 (1644,5,140,2)

IF RAP1-FHL1 AND m_nitrogen_and_sulphur_transport_n9-FHL1 AND ALPHA1-GAT3 THEN 000 OR 010
010: 0.060677 (1644,5,140,2)

IF MIG1-SKO1 AND m_MERE11-SUT1 THEN 000 OR 010 OR 100

010: 0.060677 (1644,5,140,2)

IF m_anion_transporters_n22-RAP1 AND RAP1-FHL1 AND ALPHA1-GAT3 THEN 000 OR 010
010: 0.060677 (1644,5,140,2)

IF SFF'-HIR3 AND RAP1-PDR1 AND RAP1-RGM1 THEN 000 OR 010
010: 0.060677 (1644,5,140,2)

IF m_g-proteins_n12-SKN7 AND m_tricarboxylic-acid_pathway_n9-SKN7 THEN 000 OR 010 OR 111
010: 0.060677 (1644,5,140,2)

IF RAP1-GAT3 AND RAP1-PDR1 AND RAP1-ZAP1 THEN 000 OR 010
010: 0.060677 (1644,5,140,2)

IF m_other_morphogenetic_activities_n7-RCS1 AND m_translational_control_n10-MET28 THEN 000 OR 010 OR 100
010: 0.060677 (1644,5,140,2)

IF RAP1-YAP5 AND RAP1-ZAP1 AND ALPHA1-GAT3 THEN 000 OR 010
010: 0.060677 (1644,5,140,2)

IF SFF'-HIR3 AND RAP1-FHL1 AND RAP1-YAP5 AND RAP1-ZAP1 THEN 000 OR 010
010: 0.060677 (1644,5,140,2)

IF RAP1-FHL1 AND RAP1-PDR1 AND m_nitrogen_and_sulphur_transport_n9-FHL1 THEN 000 OR 010
010: 0.060677 (1644,5,140,2)

IF m_rRSE10-PDR1 AND RAP1-YAP5 THEN 000 OR 010
010: 0.060677 (1644,5,140,2)

IF m_RRSE3-SWI4 AND m_amino-acid_transporters_n11-SWI4 AND SCB-SWI4 THEN 000 OR 010 OR 110
010: 0.060677 (1644,5,140,2)

IF SWI5-SKN7 AND SCB-SWI4 THEN 000 OR 010 OR 110
010: 0.060677 (1644,5,140,2)

IF MCM1'-SWI4 AND m_amino-acid_transporters_n11-SWI4 AND SCB-SWI4 THEN 000 OR 010 OR 111
010: 0.060677 (1644,5,140,2)

IF RAP1-GAT3 AND m_nitrogen_and_sulphur_transport_n9-FHL1 AND ALPHA1-GAT3 THEN 000 OR 010
010: 0.060677 (1644,5,140,2)

IF RAP1-PDR1 AND m_nitrogen_and_sulphur_transport_n9-FHL1 AND ALPHA1-GAT3 THEN 000 OR 010
010: 0.060677 (1644,5,140,2)

IF MCM1'-SWI4 AND SWI5-SWI6 AND m_amino-acid_transporters_n11-SWI4 THEN 000 OR 010
010: 0.060677 (1644,5,140,2)

IF m_g-proteins_n11-INO4 AND SWI5-INO4 AND m_regulation_of_lipid_fatty-acid_and_isoprenoid_biosynthesis_n12.scn-INO2 THEN 000 OR 010
010: 0.060677 (1644,5,140,2)

IF RAP1-RAP1 AND RAP1-YAP5 AND m_nitrogen_and_sulphur_transport_n9-FHL1 THEN 000 OR 010
010: 0.060677 (1644,5,140,2)

IF m_g-proteins_n12-INO4 AND m_phosphate_transport_n5-INO4 THEN 010 OR 100 OR 110
010: 0.060677 (1644,5,140,2)

IF m_anion_transporters_n19-MTH1 AND m_cell_death_n22-MTH1 THEN 000 OR 010
010: 0.060677 (1644,5,140,2)

IF mPROTEOL18(m_proteolysis_n18)-SWI6 AND m_other_transport_facilitators_n5-SWI6 THEN 000 OR 010 OR 100
010: 0.060677 (1644,5,140,2)

IF m_anion_transporters_n22-RAP1 AND RAP1-RAP1 AND ALPHA1-GAT3 THEN 000 OR 010
010: 0.060677 (1644,5,140,2)

IF RAP1-FHL1 AND RAP1-GAT3 AND m_nitrogen_and_sulphur_transport_n9-FHL1 THEN 000 OR 010
010: 0.060677 (1644,5,140,2)

IF mPROTEOL18(m_proteolysis_n18)-UME6 AND m_amino-acid_transporters_n11-UME6 THEN 000 OR 010

010: 0.060677 (1644,5,140,2)

IF RAP1-FHL1 AND RAP1-YAP5 AND m_nitrogen_and_sulphur_transport_n9-FHL1 THEN 000 OR 010
010: 0.060677 (1644,5,140,2)

IF m_g-proteins_n11-HAL9 AND SFF-ARG80 THEN 000 OR 010 OR 110
010: 0.060677 (1644,5,140,2)

IF SFF-ARG80 AND ALPHA1-DAT1 THEN 000 OR 010 OR 110
010: 0.060677 (1644,5,140,2)

IF m_amino-acid_transporters_n11-CUP9 AND m_amino-acid_transporters_n11-UME6 THEN 000 OR 010
010: 0.060677 (1644,5,140,2)

IF m_g-proteins_n11-INO4 AND m_g-proteins_n11-UME6 THEN 000 OR 001 OR 010 OR 100
010: 0.060677 (1644,5,140,2)

IF m_glyoxylate_cycle_n8-INO4 AND m_ion_transporters_n4-INO4 THEN 000 OR 010
010: 0.060677 (1644,5,140,2)

IF m_osmosensing_n6-INO4 AND m_ion_transporters_n4-INO4 THEN 000 OR 010
010: 0.060677 (1644,5,140,2)

IF RAP1-YAP5 AND m_nitrogen_and_sulphur_transport_n9-FHL1 AND ALPHA1-GAT3 THEN 000 OR 010
010: 0.060677 (1644,5,140,2)

IF m_g-proteins_n12-INO4 AND m_amino-acid_transporters_n11-SWI4 THEN 000 OR 010 OR 110 OR 111
010: 0.060677 (1644,5,140,2)

IF m_other_proteolytic_degradation_n2-MTH1 AND m_regulation_of_nitrogen_and_sulphur_utilization_n10-MTH1 THEN 000 OR 010
010: 0.060677 (1644,5,140,2)

IF m_anion_transporters_n4-MTH1 AND m_amino-acid_degradation_n8-MTH1 THEN 000 OR 010
010: 0.060677 (1644,5,140,2)

IF m_PNDE6-SFP1 THEN 000 OR 010 OR 100 OR 110
100: 0.062471 (1644,20,127,4)

IF m_phosphate_transport_n18-INO4 THEN 000 OR 001 OR 010 OR 100 OR 110 OR 111
111: 0.066295 (1644,37,19,2)

IF SFF'-RTG3 AND SFF-ARG80 THEN 000 OR 001 OR 010 OR 110
001: 0.067706 (1644,13,55,2)

IF SFF-ARG80 AND SFF-RTG3 THEN 000 OR 001 OR 010 OR 110
001: 0.067706 (1644,13,55,2)

IF m_regulation_of_nitrogen_and_sulphur_utilization_n13-MTH1 THEN 000 OR 001 OR 010 OR 100 OR 110
001: 0.067706 (1644,13,55,2)

IF STRE-RAP1 THEN 000 OR 001 OR 010 OR 100
001: 0.067706 (1644,13,55,2)

IF m_g-proteins_n12-SKN7 AND m_amino-acid_transport_n18-SKN7 THEN 000 OR 001 OR 010 OR 100 OR 110
001: 0.067706 (1644,13,55,2)

IF m_g-proteins_n12-INO4 AND m_organization_of_chromosome_structure_n17-INO4 THEN 000 OR 001 OR 100 OR 110
001: 0.067706 (1644,13,55,2)

IF m_other_pheromone_response_activities_n12-MAL33 THEN 000 OR 001 OR 010 OR 100 OR 110
001: 0.067706 (1644,13,55,2)

IF m_g-proteins_n12-INO4 AND m_other_mrna-transcription_activities_n20-INO4 THEN 000 OR 001 OR 010 OR 110
001: 0.067706 (1644,13,55,2)

IF m_RPE68-RAP1 THEN 000 OR 001 OR 010 OR 100
001: 0.067706 (1644,13,55,2)

IF MCM1'-NDD1 AND MCM1'-SWI4 THEN 000 OR 100 OR 110
110: 0.068875 (1644,14,115,3)

IF m_cytok9-SWI6 THEN 000 OR 010 OR 100 OR 110 OR 111
110: 0.068875 (1644,14,115,3)

IF m_other_energy_generation_activities_n22-HIR1 THEN 000 OR 010 OR 110
110: 0.068875 (1644,14,115,3)

IF m_other_energy_generation_activities_n12-SWI6 THEN 000 OR 100 OR 101 OR 110
110: 0.068875 (1644,14,115,3)

IF SWI5-SKN7 AND m_amino-acid_transporters_n11-SKN7 THEN 000 OR 001 OR 010 OR 110
110: 0.068875 (1644,14,115,3)

IF m_organization_of_intracellular_transport_vesicles_n5-SWI4 THEN 000 OR 010 OR 100 OR 101 OR 110
110: 0.068875 (1644,14,115,3)

IF MCM1'-NDD1 AND SWI5-SWI6 THEN 000 OR 001 OR 010 OR 100 OR 110 OR 111
110: 0.068875 (1644,14,115,3)

IF SFF'-MCM1 AND SFF'-RLM1 THEN 000 OR 010 OR 100 OR 110 OR 111
110: 0.068875 (1644,14,115,3)

IF m_g-proteins_n12-SKN7 AND SWI5-SKN7 THEN 000 OR 001 OR 010 OR 100 OR 110 OR 111
010: 0.07082 (1644,35,140,6)

IF m_MERE11-SUT1 AND CSRE-SUT1 THEN 000 OR 010 OR 100
100: 0.072297 (1644,6,127,2)

IF m_organization_of_chromosome_structure_n12-HIR2 THEN 000 OR 100 OR 101 OR 110
100: 0.072297 (1644,6,127,2)

IF m_anion_transporters_n17-INO4 AND m_anion_transporters_n15-INO4 THEN 000 OR 100
100: 0.072297 (1644,6,127,2)

IF m_g-proteins_n11-INO4 AND m_anion_transporters_n15-INO4 THEN 000 OR 100 OR 110
100: 0.072297 (1644,6,127,2)

IF MCM1'-NDD1 AND m_allantoin_and_allantoate_transporters_n18-FKH2 THEN 010 OR 100 OR 110
100: 0.072297 (1644,6,127,2)

IF CSRE-SKN7 AND m_amino-acid_metabolism_n14-SKN7 THEN 000 OR 100
100: 0.072297 (1644,6,127,2)

IF m_anion_transporters_n17-SKN7 AND m_organization_of_chromosome_structure_n17-SKN7 THEN 000 OR 001 OR 100
100: 0.072297 (1644,6,127,2)

IF m_g-proteins_n11-INO4 AND m_organization_of_chromosome_structure_n12-INO4 THEN 000 OR 001 OR 100 OR 110
100: 0.072297 (1644,6,127,2)

IF m_g-proteins_n12-INO4 AND m_organization_of_chromosome_structure_n12-INO4 THEN 000 OR 001 OR 100
100: 0.072297 (1644,6,127,2)

IF m_phosphate_transport_n13-SKN7 AND m_RRSE3-SKN7 THEN 000 OR 100
100: 0.072297 (1644,6,127,2)

IF m_cell_death_n16-MBP1 AND m_cell_death_n16-SKN7 THEN 000 OR 100 OR 111
100: 0.072297 (1644,6,127,2)

IF m_g-proteins_n12-INO4 AND m_g-proteins_n12-NRG1 THEN 000 OR 010 OR 100
100: 0.072297 (1644,6,127,2)

IF m_phosphate_transport_n18-INO4 AND m_phosphate_transport_n18-PUT3 THEN 000 OR 100
100: 0.072297 (1644,6,127,2)

IF SFF'-FKH1 AND SFF-MIG1 THEN 000 OR 010 OR 100
100: 0.072297 (1644,6,127,2)

IF SFF-FKH1 AND SFF-MIG1 THEN 000 OR 010 OR 100
100: 0.072297 (1644,6,127,2)

IF m_anion_transporters_n10-INO4 AND m_other_energy_generation_activities_n22-INO4 THEN 000 OR 010 OR 100
100: 0.072297 (1644,6,127,2)

IF m_g-proteins_n11-INO4 AND SWI5-INO4 AND m_other_energy_generation_activities_n22-INO4 THEN 000 OR 010 OR 100
100: 0.072297 (1644,6,127,2)

IF m_osmosensing_n6-SWI4 AND SFF-FKH2 THEN 000 OR 100 OR 110 OR 111
100: 0.072297 (1644,6,127,2)

IF m_g-proteins_n11-INO4 AND SWI5-INO4 AND m_pentose-phosphate_pathway_n23-INO4 THEN 000 OR 001 OR 100 OR 110
100: 0.072297 (1644,6,127,2)

IF SFF'-FKH1 AND SFF-FKH2 AND m_other_morphogenetic_activities_n7-MCM1 THEN 000 OR 100 OR 110 OR 111
100: 0.072297 (1644,6,127,2)

IF SFF-FKH1 AND SFF-FKH2 AND m_other_morphogenetic_activities_n7-MCM1 THEN 000 OR 100 OR 110 OR 111
100: 0.072297 (1644,6,127,2)

IF m_other_intracellular-transport_activities_n9-YBL054W THEN 000 OR 100
100: 0.072297 (1644,6,127,2)

IF SFF'-FKH2 AND ABF1-ABF1 THEN 000 OR 010 OR 100 OR 110
100: 0.072297 (1644,6,127,2)

IF m_phosphate_transport_n13-GAT1 AND m_phosphate_transport_n18-GAT1 THEN 000 OR 010 OR 100
100: 0.072297 (1644,6,127,2)

IF SFF'-FKH2 AND SFF-FKH1 AND m_other_morphogenetic_activities_n7-MCM1 THEN 000 OR 100 OR 110 OR 111
100: 0.072297 (1644,6,127,2)

IF m_g-proteins_n11-INO4 AND SWI5-SWI6 THEN 000 OR 010 OR 100 OR 110
100: 0.072297 (1644,6,127,2)

IF m_anion_transporters_n15-MTH1 AND m_other_nutritional-response_activities_n10-MTH1 THEN 000 OR 100
100: 0.072297 (1644,6,127,2)

IF MCM1'-SWI4 AND SWI5-SWI6 AND m_pentose-phosphate_pathway_n5-SWI4 THEN 000 OR 100 OR 101
100: 0.072297 (1644,6,127,2)

IF MCM1'-NDD1 AND MCM1'-SWI4 AND m_pentose-phosphate_pathway_n5-SWI4 THEN 000 OR 100
100: 0.072297 (1644,6,127,2)

IF m_other_transcription_activities_n5-SWI6 AND m_ionic_homeostasis_n6-SWI6 THEN 000 OR 010 OR 100 OR 101 OR 110
100: 0.072297 (1644,6,127,2)

IF m_g-proteins_n11-INO4 AND SWI5-INO4 AND SWI5-SWI6 THEN 000 OR 010 OR 100 OR 110
100: 0.072297 (1644,6,127,2)

IF m_MERE4-MTH1 AND m_MERE17-MTH1 THEN 000 OR 100
100: 0.072297 (1644,6,127,2)

IF m_drug_transporters_n10-INO4 AND m_deoxyribonucleotide_metabolism_n10-INO4 THEN 000 OR 100
100: 0.072297 (1644,6,127,2)

IF m_regulation_of_nitrogen_and_sulphur_utilization_n7-INO4 AND PHO4-INO4 THEN 000 OR 100
100: 0.072297 (1644,6,127,2)

IF m_pheromone_response_generation_n10-DIG1 THEN 000 OR 100
100: 0.072297 (1644,6,127,2)

IF m_drug_transporters_n10-INO4 AND m_drug_transporters_n10-MET4 THEN 000 OR 100
100: 0.072297 (1644,6,127,2)

IF SFF-FKH2 AND SWI5-INO4 THEN 000 OR 100 OR 110
100: 0.072297 (1644,6,127,2)

IF SFF'-FKH2 AND SFF-FKH1 AND SWI5-INO4 THEN 000 OR 100 OR 110
100: 0.072297 (1644,6,127,2)

IF SFF'-FKH1 AND m_metal_ion_transporters_n10-SKN7 THEN 000 OR 100 OR 110

100: 0.072297 (1644,6,127,2)

IF SFF-FKH1 AND m_anion_transporters_n15-INO4 THEN 000 OR 010 OR 100
100: 0.072297 (1644,6,127,2)

IF RPN4-REB1 AND RPN4-RPN4 THEN 000 OR 001 OR 100
100: 0.072297 (1644,6,127,2)

IF MCM1'-NDD1 AND m_pentose-phosphate_pathway_n5-SWI4 THEN 000 OR 100
100: 0.072297 (1644,6,127,2)

IF SFF-FKH1 AND m_metal_ion_transporters_n10-SKN7 THEN 000 OR 100 OR 110
100: 0.072297 (1644,6,127,2)

IF m_phosphate_transport_n18-INO4 AND m_purine_and_pyrimidine_transporters_n17-INO4 THEN 000 OR 100
100: 0.072297 (1644,6,127,2)

IF m_g-proteins_n12-INO4 AND SWI5-INO4 AND m_phosphate_transport_n18-INO4 THEN 000 OR 010 OR 100
100: 0.072297 (1644,6,127,2)

IF m_lipid_and_fatty-acid_transport_n11-SKN7 AND m_deoxyribonucleotide_metabolism_n10-SKN7 THEN 000 OR 100
100: 0.072297 (1644,6,127,2)

IF m_other_pheromone_response_activities_n14-ARG80 THEN 000 OR 100
100: 0.072297 (1644,6,127,2)

IF m_phosphate_transport_n13-GAT1 AND m_purine_and_pyrimidine_transporters_n17-GAT1 THEN 000 OR 001 OR 100
100: 0.072297 (1644,6,127,2)

IF PHO4-INO4 AND m_cell_death_n16-INO4 THEN 000 OR 001 OR 100
100: 0.072297 (1644,6,127,2)

IF m_glyoxylate_cycle_n8-UME6 AND CSRE-UME6 AND m_meiosis_n3-UME6 THEN 000 OR 001 OR 100
100: 0.072297 (1644,6,127,2)

IF m_cell_death_n15-MOT3 THEN 000 OR 100 OR 110
100: 0.072297 (1644,6,127,2)

IF m_anion_transporters_n15-GAT1 AND m_anion_transporters_n15-INO4 THEN 000 OR 010 OR 100
100: 0.072297 (1644,6,127,2)

IF m_nutritional_response_pathway_n3-SWI4 THEN 000 OR 100 OR 110 OR 111
100: 0.072297 (1644,6,127,2)

IF m_other_mrna-transcription_activities_n20-INO4 AND m_other_mrna-transcription_activities_n20-XBP1 THEN 000 OR 001 OR 100 OR 110
100: 0.072297 (1644,6,127,2)

IF m_ion_transporters_n4-GAT1 AND m_phosphate_transport_n18-GAT1 THEN 000 OR 100 OR 111
100: 0.072297 (1644,6,127,2)

IF m_other_nucleotide-metabolism_activities_n18-PUT3 THEN 000 OR 001 OR 010 OR 100 OR 110
100: 0.072693 (1644,13,127,3)

IF m_other_nucleotide-metabolism_activities_n17-YKL222C THEN 000 OR 001 OR 010 OR 100
100: 0.072693 (1644,13,127,3)

IF m_amino-acid_transport_n18-MET4 THEN 000 OR 010 OR 100 OR 111
100: 0.072693 (1644,13,127,3)

IF SFF'-FKH1 AND SWI5-INO4 THEN 000 OR 010 OR 100 OR 110
100: 0.072693 (1644,13,127,3)

IF m_phosphate_transport_n18-XBP1 THEN 000 OR 001 OR 010 OR 100 OR 110 OR 111
001: 0.073419 (1644,48,55,4)

IF m_peroxisomal_transport_n22-RAP1 THEN 000 OR 010 OR 101
010: 0.075184 (1644,12,140,3)

IF m_rRSE10-PDR1 THEN 000 OR 010 OR 100 OR 110
010: 0.075184 (1644,12,140,3)

IF m_g-proteins_n12-SKN7 AND SWI5-INO4 THEN 000 OR 001 OR 010 OR 100 OR 110
010: 0.075184 (1644,12,140,3)

IF m_g-proteins_n12-INO4 AND SWI5-SKN7 THEN 000 OR 001 OR 010 OR 100 OR 110
010: 0.075184 (1644,12,140,3)

IF m_other_nutritional-response_activities_n10-MTH1 AND m_cell_death_n22-MTH1 THEN 000 OR 001 OR 100 OR 110
001: 0.077335 (1644,14,55,2)

IF HAP234-HAP4 THEN 000 OR 001 OR 010 OR 100 OR 110
001: 0.077335 (1644,14,55,2)

IF m_amino-acid_degradation_n7-GAT1 THEN 000 OR 001 OR 010 OR 100 OR 111
001: 0.077335 (1644,14,55,2)

IF m_deoxyribonucleotide_metabolism_n12-TOS8 THEN 000 OR 001 OR 010 OR 110
001: 0.077335 (1644,14,55,2)

IF SWI5-INO4 AND m_other_mrna-transcription_activities_n20-INO4 THEN 000 OR 001 OR 010 OR 100 OR 110
001: 0.077335 (1644,14,55,2)

IF SWI5-INO4 AND m_organization_of_chromosome_structure_n17-INO4 THEN 000 OR 001 OR 100 OR 110
001: 0.077335 (1644,14,55,2)

IF SFF-ARG80 THEN 000 OR 001 OR 010 OR 011 OR 100 OR 110
001: 0.078021 (1644,49,55,4)

IF m_lipid_and_fatty-acid_transport_n11-GAT1 AND m_LFTE17-GAT1 AND m_anion_transporters_n20-GAT1 THEN 000 OR 110 OR 111
110: 0.080812 (1644,7,115,2)

IF SFF'-RLM1 AND SWI5-SKN7 THEN 000 OR 110 OR 111
110: 0.080812 (1644,7,115,2)

IF SFF'-FKH2 AND m_other_cell_growth_cell_division_and_dna_synthesis_activities_n10.scn-NDD1 THEN 000 OR 010 OR 110
110: 0.080812 (1644,7,115,2)

IF STE12-DIG1 THEN 000 OR 001 OR 100 OR 110
110: 0.080812 (1644,7,115,2)

IF m_other_morphogenetic_activities_n7-HOG1 AND m_other_morphogenetic_activities_n7-MDS3 THEN 000 OR 010 OR 110
110: 0.080812 (1644,7,115,2)

IF SFF-FKH1 AND m_other_morphogenetic_activities_n7-MCM1 THEN 000 OR 100 OR 110 OR 111
110: 0.080812 (1644,7,115,2)

IF m_g-proteins_n12-INO4 AND SFF-FKH2 THEN 000 OR 110
110: 0.080812 (1644,7,115,2)

IF m_deoxyribonucleotide_metabolism_n23-MTH1 AND m_deoxyribonucleotide_metabolism_n23-SWI4 THEN 000 OR 100 OR 110
110: 0.080812 (1644,7,115,2)

IF m_g-proteins_n12-INO4 AND SFF-FKH1 THEN 000 OR 010 OR 100 OR 110
110: 0.080812 (1644,7,115,2)

IF m_allantoin_and_allantoate_transporters_n6-PHO4 THEN 000 OR 010 OR 110
110: 0.080812 (1644,7,115,2)

IF SFF'-FKH2 AND m_osmosensing_n6-INO4 THEN 000 OR 100 OR 110
110: 0.080812 (1644,7,115,2)

IF m_cytoskeleton-dependenttransport_n4-ACE2 THEN 000 OR 010 OR 110 OR 111
110: 0.080812 (1644,7,115,2)

IF m_phosphate_transport_n18-INO4 AND m_homeostasis_of_other_ions_n30-INO4 THEN 000 OR 100 OR 110
110: 0.080812 (1644,7,115,2)

IF m_pentose-phosphate_pathway_n14-FKH1 AND m_pentose-phosphate_pathway_n14-MTH1 THEN 000 OR 010 OR 100 OR 110
110: 0.080812 (1644,7,115,2)

IF m_g-proteins_n12-INO4 AND MCM1'-NDD1 THEN 000 OR 010 OR 100 OR 110
110: 0.080812 (1644,7,115,2)

IF m_pentose-phosphate_pathway_n14-FKH1 AND m_pentose-phosphate_pathway_n14-GAT1 THEN 000 OR 010 OR 100 OR 110
110: 0.080812 (1644,7,115,2)

IF m_peroxisomal_organization_n6-RCO1 THEN 000 OR 001 OR 100 OR 110
110: 0.080812 (1644,7,115,2)

IF m_intracellular_communication_n10-INO4 THEN 000 OR 110
110: 0.080812 (1644,7,115,2)

IF SWI5-SWI6 AND m_metal_ion_transporters_n10-SWI6 THEN 000 OR 100 OR 110
110: 0.080812 (1644,7,115,2)

IF mPROTEOL18(m_proteolysis_n18)-SWI6 AND STRE-SWI6 THEN 000 OR 010 OR 100 OR 110
110: 0.080812 (1644,7,115,2)

IF m_sugar_and_carbohydrate_transporters_n6-SKN7 THEN 000 OR 100 OR 110
110: 0.080812 (1644,7,115,2)

IF m_MERE4-SKN7 AND SFF-FKH1 THEN 000 OR 010 OR 110
110: 0.080812 (1644,7,115,2)

IF m_ionic_homeostasis_n6-SWI6 AND m_cell_death_n22-SWI4 THEN 000 OR 101 OR 110
110: 0.080812 (1644,7,115,2)

IF Gcr1-SWI6 AND LYS14-SWI6 THEN 000 OR 100 OR 110
110: 0.080812 (1644,7,115,2)

IF m_organization_of_intracellular_transport_vesicles_n5-SWI4 AND MCM1'-SWI4 AND m_cell_death_n22-SWI4 THEN 000 OR 010 OR 101 OR 110
110: 0.080812 (1644,7,115,2)

IF m_other_morphogenetic_activities_n7-HOG1 AND m_other_morphogenetic_activities_n7-RCS1 THEN 000 OR 100 OR 110
110: 0.080812 (1644,7,115,2)

IF m_osmosensing_n6-INO4 AND m_glyoxylate_cycle_n8-INO4 THEN 000 OR 010 OR 110
110: 0.080812 (1644,7,115,2)

IF SFF'-HIR1 AND m_other_morphogenetic_activities_n7-HOG1 THEN 000 OR 110
110: 0.080812 (1644,7,115,2)

IF m_g-proteins_n12-SKN7 AND m_nitrogen_and_sulphur_metabolism_n17-SKN7 THEN 000 OR 010 OR 110
110: 0.080812 (1644,7,115,2)

IF m_g-proteins_n12-SKN7 AND m_anion_transporters_n15-INO4 THEN 000 OR 010 OR 110 OR 111
110: 0.080812 (1644,7,115,2)

IF SFF-HIR1 AND m_other_morphogenetic_activities_n7-HOG1 THEN 000 OR 110
110: 0.080812 (1644,7,115,2)

IF m_g-proteins_n11-INO4 AND m_other_mrna-transcription_activities_n20-SKN7 THEN 000 OR 001 OR 010 OR 100 OR 110
110: 0.080812 (1644,7,115,2)

IF m_osmosensing_n6-INO4 AND m_g-proteins_n11-INO4 THEN 000 OR 100 OR 101 OR 110
110: 0.080812 (1644,7,115,2)

IF m_g-proteins_n11-INO4 AND SWI5-INO4 AND m_other_mrna-transcription_activities_n20-INO4 THEN 000 OR 001 OR 010 OR 100 OR 110
110: 0.080812 (1644,7,115,2)

IF m_regulation_of_amino-acid_metabolism_n11-CBF1 THEN 000 OR 010 OR 110
110: 0.080812 (1644,7,115,2)

IF m_other_energy_generation_activities_n12-SWI6 AND SWI5-SWI6 THEN 000 OR 100 OR 101 OR 110
110: 0.080812 (1644,7,115,2)

IF SFF-FKH2 AND m_amino-acid_degradation_n7-NDD1 THEN 000 OR 001 OR 100 OR 110
110: 0.080812 (1644,7,115,2)

IF m_anion_transporters_n15-GAT1 AND m_anion_transporters_n15-MTH1 THEN 000 OR 100 OR 110
110: 0.080812 (1644,7,115,2)

IF SWI5-SWI6 AND m_LFTE17-GAT1 THEN 000 OR 001 OR 100 OR 110
110: 0.080812 (1644,7,115,2)

IF ABF1-SNF1 THEN 000 OR 010 OR 100 OR 110
110: 0.080812 (1644,7,115,2)

IF m_g-proteins_n12-SKN7 AND m_amino-acid_transporters_n11-SWI4 THEN 000 OR 010 OR 110 OR 111
110: 0.080812 (1644,7,115,2)

IF m_lipid_and_fatty-acid_transport_n11-SKN7 AND m_other_mrna-transcription_activities_n20-INO4 THEN 000 OR 010 OR 110
110: 0.080812 (1644,7,115,2)

IF SFF'-RLM1 AND SCB-SWI4 THEN 000 OR 110 OR 111
110: 0.080812 (1644,7,115,2)

IF m_g-proteins_n12-INO4 AND SWI5-INO4 AND SWI5-SKN7 AND SWI5-SWI6 THEN 000 OR 010 OR 110
110: 0.080812 (1644,7,115,2)

IF m_g-proteins_n12-SKN7 AND SWI5-INO4 AND SWI5-SWI6 THEN 000 OR 010 OR 110
110: 0.080812 (1644,7,115,2)

IF m_g-proteins_n12-SKN7 AND m_amino-acid_transporters_n11-SKN7 AND m_amino-acid_transporters_n11-SWI4 THEN 000 OR 010 OR 110 OR 111
110: 0.080812 (1644,7,115,2)

IF m_g-proteins_n12-INO4 AND SWI5-SKN7 AND SWI5-SWI6 THEN 000 OR 010 OR 110
110: 0.080812 (1644,7,115,2)

IF m_g-proteins_n12-INO4 AND m_g-proteins_n12-SKN7 AND SWI5-SWI6 THEN 000 OR 010 OR 110
110: 0.080812 (1644,7,115,2)

IF m_g-proteins_n11-INO4 AND SFF-FKH1 THEN 000 OR 100 OR 110
110: 0.080812 (1644,7,115,2)

IF m_deoxyribonucleotide_metabolism_n4-SWI6 THEN 000 OR 010 OR 110 OR 111
110: 0.080812 (1644,7,115,2)

IF m_g-proteins_n11-INO4 AND m_other_mrna-transcription_activities_n20-INO4 AND m_other_mrna-transcription_activities_n20-SKN7 THEN 000 OR 001 OR 010 OR 100 OR 110
110: 0.080812 (1644,7,115,2)

IF ALPHA1-DAT1 THEN 000 OR 010 OR 100 OR 110
110: 0.081402 (1644,24,115,4)

IF m_g-proteins_n12-SKN7 AND m_amino-acid_transporters_n11-SKN7 THEN 000 OR 001 OR 010 OR 110 OR 111
110: 0.08185 (1644,15,115,3)

IF m_metal_ion_transporters_n10-SWI6 THEN 000 OR 100 OR 110
110: 0.08185 (1644,15,115,3)

IF m_g-proteins_n11-HAL9 THEN 000 OR 010 OR 100 OR 110
010: 0.083569 (1644,20,140,4)

IF ALPHA1'-KSS1 AND ALPHA1'-RIM101 THEN 000 OR 010 OR 100 OR 101 OR 110
010: 0.083569 (1644,20,140,4)

IF m_pentose-phosphate_pathway_n14-MTH1 THEN 000 OR 010 OR 100 OR 110
110: 0.083684 (1644,34,115,5)

IF m_RRSE3-SWI4 THEN 000 OR 010 OR 100 OR 101 OR 110
100: 0.083779 (1644,22,127,4)

IF m_pentose-phosphate_pathway_n23-INO4 THEN 000 OR 001 OR 010 OR 100 OR 110
100: 0.083779 (1644,22,127,4)

IF m_anion_transporters_n15-GAT1 THEN 000 OR 001 OR 010 OR 100 OR 101 OR 110
100: 0.083779 (1644,22,127,4)

IF m_other_energy_generation_activities_n4-ABF1 AND ABF1-ABF1 THEN 000 OR 010
010: 0.086024 (1644,6,140,2)

IF m_g-proteins_n12-SKN7 AND m_anion_transporters_n10-INO4 THEN 000 OR 010
010: 0.086024 (1644,6,140,2)

IF RAP1-FHL1 AND RAP1-YAP5 AND RAP1-ZAP1 THEN 000 OR 010
010: 0.086024 (1644,6,140,2)

IF SFF'-RTG3 AND SFF-HOG1 THEN 000 OR 001 OR 010
010: 0.086024 (1644,6,140,2)

IF SFF'-HIR3 AND RAP1-SMP1 THEN 000 OR 010
010: 0.086024 (1644,6,140,2)

IF m_anion_transporters_n22-RAP1 AND RAP1-YAP5 THEN 000 OR 010
010: 0.086024 (1644,6,140,2)

IF m_g-proteins_n11-HAL9 AND ALPHA1-DAT1 THEN 000 OR 010 OR 110
010: 0.086024 (1644,6,140,2)

IF RAP1-PDR1 AND RAP1-YAP5 AND RAP1-ZAP1 THEN 000 OR 010
010: 0.086024 (1644,6,140,2)

IF SFF'-MCM1 AND ALPHA1'-RIM101 THEN 000 OR 010
010: 0.086024 (1644,6,140,2)

IF SFF-HOG1 AND m_other_morphogenetic_activities_n7-GCR2 THEN 000 OR 010 OR 110
010: 0.086024 (1644,6,140,2)

IF m_other_morphogenetic_activities_n7-GCR2 AND m_other_morphogenetic_activities_n7-HOG1 THEN 000 OR 010 OR 110
010: 0.086024 (1644,6,140,2)

IF SFF'-HIR3 AND m_other_morphogenetic_activities_n7-STP1 THEN 000 OR 010
010: 0.086024 (1644,6,140,2)

IF RAP1-FHL1 AND RAP1-RAP1 AND RAP1-ZAP1 THEN 000 OR 010
010: 0.086024 (1644,6,140,2)

IF SWI5-SKN7 AND m_tricarboxylic-acid_pathway_n9-SKN7 THEN 000 OR 010 OR 111
010: 0.086024 (1644,6,140,2)

IF RAP1-PDR1 AND RAP1-RAP1 AND RAP1-ZAP1 THEN 000 OR 010
010: 0.086024 (1644,6,140,2)

IF m_anion_transporters_n22-RAP1 AND RAP1-FHL1 AND RAP1-YAP5 THEN 000 OR 010
010: 0.086024 (1644,6,140,2)

IF SWI5-SKN7 AND m_anion_transporters_n10-INO4 THEN 000 OR 010
010: 0.086024 (1644,6,140,2)

IF m_anion_transporters_n4-MTH1 AND m_rRSE10-MTH1 THEN 000 OR 010
010: 0.086024 (1644,6,140,2)

IF MCM1'-SWI4 AND SCB-SWI4 AND SCB-UME6 THEN 000 OR 010 OR 110
010: 0.086024 (1644,6,140,2)

IF m_anion_transporters_n4-MTH1 AND m_anion_transporters_n19-MTH1 THEN 000 OR 010
010: 0.086024 (1644,6,140,2)

IF m_rRSE10-PDR1 AND RAP1-PDR1 THEN 000 OR 010
010: 0.086024 (1644,6,140,2)

IF ALPHA1'-UPC2 AND m_chromatin_modification_n21-HSF1 THEN 000 OR 001 OR 010 OR 100
010: 0.086024 (1644,6,140,2)

IF ALPHA1'-YER051w AND m_chromatin_modification_n21-HSF1 THEN 000 OR 001 OR 010 OR 100
010: 0.086024 (1644,6,140,2)

IF m_utilization_of_vitamins_cofactors_and_prosthetic_groups_n7-RDR1 THEN 000 OR 010
010: 0.086024 (1644,6,140,2)

IF AFT1-RAP1 AND RAP1-FHL1 THEN 000 OR 010
010: 0.086024 (1644,6,140,2)

IF SFF'-HIR1 AND m_g-proteins_n12-INO4 THEN 000 OR 010 OR 100 OR 110
010: 0.086024 (1644,6,140,2)

IF m_g-proteins_n12-INO4 AND SFF-HIR1 THEN 000 OR 010 OR 100 OR 110
010: 0.086024 (1644,6,140,2)

IF SFF'-HIR3 AND SFF-HOG1 THEN 000 OR 010
010: 0.086024 (1644,6,140,2)

IF SFF-HOG1 AND SFF-RTG3 THEN 000 OR 001 OR 010
010: 0.086024 (1644,6,140,2)

IF m_utilization_of_vitamins_cofactors_and_prosthetic_groups_n6-EDS1 THEN 000 OR 010 OR 101
010: 0.086024 (1644,6,140,2)

IF SFF'-RLM1 AND m_g-proteins_n11-UME6 THEN 000 OR 010
010: 0.086024 (1644,6,140,2)

IF m_osmosensing_n6-INO4 AND m_homeostasis_of_other_ions_n30-INO4 THEN 000 OR 010
010: 0.086024 (1644,6,140,2)

IF m_anion_transporters_n15-INO4 AND m_glycolysis_and_gluconeogenesis_n4-INO4 THEN 000 OR 010 OR 110
010: 0.086024 (1644,6,140,2)

IF m_MERE4-SKN7 AND m_RPE57-SKN7 THEN 000 OR 010
010: 0.086024 (1644,6,140,2)

IF m_glyoxylate_cycle_n8-INO4 AND m_homeostasis_of_other_ions_n30-INO4 THEN 000 OR 010 OR 110
010: 0.086024 (1644,6,140,2)

IF m_RRSE3-INO4 AND m_ion_transporters_n4-INO4 THEN 000 OR 010
010: 0.086024 (1644,6,140,2)

IF MCM1'-SWI4 AND SCB-UME6 THEN 000 OR 010 OR 110
010: 0.086024 (1644,6,140,2)

IF SFF'-FKH1 AND m_glyoxylate_cycle_n11-GAT1 THEN 000 OR 010 OR 100 OR 110
010: 0.086024 (1644,6,140,2)

IF SFF'-FKH1 AND ALPHA1'-RIM101 AND ALPHA1'-YER051w THEN 000 OR 010 OR 100
010: 0.086024 (1644,6,140,2)

IF m_regulation_of_lipid_fatty-acid_and_isoprenoid_biosynthesis_n8.scn-MTH1 AND m_ion_transporters_n3-MTH1 THEN 000 OR 010
010: 0.086024 (1644,6,140,2)

IF m_regulation_of_amino-acid_metabolism_n7-MET32 THEN 000 OR 010
010: 0.086024 (1644,6,140,2)

IF m_regulation_of_lipid_fatty-acid_and_isoprenoid_biosynthesis_n22.scn-INO4 AND m_anion_transporters_n10-INO4 THEN 000 OR 010
010: 0.086024 (1644,6,140,2)

IF m_regulation_of_lipid_fatty-acid_and_isoprenoid_biosynthesis_n20.scn-OAF1 THEN 000 OR 010
010: 0.086024 (1644,6,140,2)

IF AFT1-RAP1 AND RAP1-GAT3 THEN 000 OR 010
010: 0.086024 (1644,6,140,2)

IF m_g-proteins_n12-NRG1 AND SWI5-SKN7 THEN 000 OR 010 OR 100
010: 0.086024 (1644,6,140,2)

IF m_tricarboxylic-acid_pathway_n9-GAT1 AND m_glyoxylate_cycle_n11-GAT1 THEN 000 OR 010 OR 111
010: 0.086024 (1644,6,140,2)

IF m_g-proteins_n12-SKN7 AND SWI5-SKN7 AND m_RPE57-SKN7 THEN 000 OR 010

010: 0.086024 (1644,6,140,2)

IF RAP1-YAP5 AND RAP1-ZAP1 THEN 000 OR 010
010: 0.086024 (1644,6,140,2)

IF m_lipid_and_fatty-acid_transport_n11-GAT1 AND m_tricarboxylic-acid_pathway_n9-GAT1 AND m_glyoxylate_cycle_n11-GAT1 THEN 000 OR 010 OR 111
010: 0.086024 (1644,6,140,2)

IF m_lipid_and_fatty-acid_binding_n15-RPN4 THEN 000 OR 010 OR 100
010: 0.086024 (1644,6,140,2)

IF mPROTEOL18(m_proteolysis_n18)-SWI6 AND SCB-SWI4 THEN 000 OR 010 OR 110 OR 111
010: 0.086024 (1644,6,140,2)

IF m_g-proteins_n12-NRG1 AND SWI5-SWI6 THEN 000 OR 010 OR 100
010: 0.086024 (1644,6,140,2)

IF m_biosynthesis_of_vitamins_cofactors_and_prosthetic_groups_n8-MAC1 THEN 000 OR 010 OR 110
010: 0.086024 (1644,6,140,2)

IF m_g-proteins_n12-NRG1 AND SCB-AZF1 THEN 000 OR 010
010: 0.086024 (1644,6,140,2)

IF MCM1'-MCM1 AND ECB-MCM1 AND m_nucleotide_transport_n9-MCM1 THEN 010 OR 100 OR 110
010: 0.086024 (1644,6,140,2)

IF m_rRSE10-PDR1 AND RAP1-FHL1 THEN 000 OR 010
010: 0.086024 (1644,6,140,2)

IF MCM1-MCM1 AND m_nucleotide_transport_n9-MCM1 THEN 010 OR 100 OR 110
010: 0.086024 (1644,6,140,2)

IF RAP1-HAP4 THEN 000 OR 010
010: 0.086024 (1644,6,140,2)

IF m_organization_of_plasma_membrane_n15-UME6 THEN 000 OR 010
010: 0.086024 (1644,6,140,2)

IF m_regulation_of_amino-acid_metabolism_n7-CBF1 THEN 000 OR 010 OR 100
010: 0.086024 (1644,6,140,2)

IF m_other_cation_transporters_n14-KSS1 THEN 000 OR 010
010: 0.086024 (1644,6,140,2)

IF SWI5-SKN7 AND SCB-AZF1 THEN 000 OR 010 OR 111
010: 0.086024 (1644,6,140,2)

IF SFF'-HIR3 AND m_other_morphogenetic_activities_n7-RCS1 THEN 000 OR 010
010: 0.086024 (1644,6,140,2)

IF m_peroxisomal_transport_n19-YAP5 THEN 000 OR 010 OR 100
010: 0.086024 (1644,6,140,2)

IF m_regulation_of_lipid_fatty-acid_and_isoprenoid_biosynthesis_n12.scn-INO4 AND ABF1-ABF1 THEN 000 OR 010
010: 0.086024 (1644,6,140,2)

IF m_other_mrna-transcription_activities_n11-SWI6 AND m_other_transport_facilitators_n10-SWI6 THEN 000 OR 010 OR 110
010: 0.086024 (1644,6,140,2)

IF m_anion_transporters_n22-SKN7 THEN 000 OR 001 OR 010 OR 110 OR 111
001: 0.087367 (1644,15,55,2)

IF m_RPE17-FHL1 THEN 000 OR 001 OR 010 OR 100
001: 0.087367 (1644,15,55,2)

IF SCB-UME6 AND m_meiosis_n3-UME6 THEN 000 OR 001 OR 010 OR 110
001: 0.087367 (1644,15,55,2)

IF m_anion_transporters_n9-MTH1 THEN 000 OR 001 OR 010 OR 100 OR 110

001: 0.087367 (1644,15,55,2)

IF m_organization_of_plasma_membrane_n17-INO4 THEN 000 OR 100 OR 101 OR 110
100: 0.087474 (1644,14,127,3)

IF m_pentose-phosphate_pathway_n5-SWI4 THEN 000 OR 100 OR 101 OR 110 OR 111
100: 0.087474 (1644,14,127,3)

IF m_allantoin_and_allantoate_transporters_n18-FKH2 THEN 000 OR 010 OR 100 OR 110
100: 0.087474 (1644,14,127,3)

IF SFF'-FKH2 AND SFF-FKH1 THEN 000 OR 010 OR 100 OR 110 OR 111
111: 0.089787 (1644,44,19,2)

IF SFF'-FKH2 AND SFF-FKH1 AND SFF-FKH2 THEN 000 OR 010 OR 100 OR 110 OR 111
111: 0.089787 (1644,44,19,2)

IF SFF'-FKH1 AND SFF-FKH2 THEN 000 OR 010 OR 100 OR 110 OR 111
111: 0.089787 (1644,44,19,2)

IF m_utilization_of_vitamins_cofactors_and_prosthetic_groups_n6-YDR049W THEN 000 OR 010 OR 101
010: 0.091855 (1644,13,140,3)

IF SFF-ARG80 AND SFF-RTG3 THEN 000 OR 001 OR 010 OR 110
010: 0.091855 (1644,13,140,3)

IF m_amino-acid_transport_n18-MET4 THEN 000 OR 010 OR 100 OR 111
010: 0.091855 (1644,13,140,3)

IF SFF'-HIR3 AND SFF-RTG3 THEN 000 OR 001 OR 010 OR 110
010: 0.091855 (1644,13,140,3)

IF m_lipid_and_fatty-acid_transport_n11-GAT1 AND m_glyoxylate_cycle_n11-GAT1 THEN 000 OR 010 OR 100 OR 110 OR 111
010: 0.091855 (1644,13,140,3)

IF SFF'-RTG3 AND SFF-ARG80 THEN 000 OR 001 OR 010 OR 110
010: 0.091855 (1644,13,140,3)

IF ABF1-WAR1 THEN 000 OR 010 OR 011 OR 100 OR 110 OR 111
010: 0.091855 (1644,13,140,3)

IF m_g-proteins_n12-SKN7 AND SWI5-SKN7 THEN 000 OR 001 OR 010 OR 100 OR 110 OR 111
110: 0.092416 (1644,35,115,5)

IF m_g-proteins_n11-INO4 AND SWI5-INO4 THEN 000 OR 001 OR 010 OR 100 OR 110
100: 0.09561 (1644,23,127,4)

IF m_nitrogen_and_sulphur_metabolism_n17-SKN7 THEN 000 OR 001 OR 010 OR 110
110: 0.095792 (1644,16,115,3)

IF m_cell_death_n16-MBP1 THEN 000 OR 100 OR 110 OR 111
110: 0.095792 (1644,16,115,3)

IF m_homeostasis_of_other_ions_n30-INO4 THEN 000 OR 001 OR 010 OR 100 OR 110
110: 0.095792 (1644,16,115,3)

IF SWI5-INO4 AND SWI5-SWI6 THEN 000 OR 010 OR 100 OR 110
110: 0.095792 (1644,16,115,3)

IF SFF-GZF3 AND SFF-MIG1 THEN 000 OR 010 OR 100 OR 110
100: 0.09621 (1644,7,127,2)

IF m_pentose-phosphate_pathway_n23-INO4 AND m_pentose-phosphate_pathway_n23-MET4 THEN 000 OR 100 OR 110
100: 0.09621 (1644,7,127,2)

IF m_allantoin_and_allantoate_transporters_n11-RIM101 THEN 000 OR 010 OR 100
100: 0.09621 (1644,7,127,2)

IF m_other_nutritional-response_activities_n10-INO4 AND m_other_pheromone_response_activities_n8-INO4 THEN 000 OR 100 OR 111
100: 0.09621 (1644,7,127,2)

IF SFF'-FKH1 AND SWI5-RIM101 THEN 000 OR 010 OR 100
100: 0.09621 (1644,7,127,2)

IF PHO-INO4 AND PHO4-INO4 THEN 000 OR 010 OR 100
100: 0.09621 (1644,7,127,2)

IF MET31-32-MET4 THEN 000 OR 010 OR 100
100: 0.09621 (1644,7,127,2)

IF m_phosphate_transport_n18-INO4 AND m_cell_death_n16-INO4 THEN 000 OR 100 OR 111
100: 0.09621 (1644,7,127,2)

IF m_phosphate_transport_n18-INO4 AND m_homeostasis_of_other_ions_n30-INO4 THEN 000 OR 100 OR 110
100: 0.09621 (1644,7,127,2)

IF m_pheromone_response_generation_n10-IFH1 THEN 000 OR 100
100: 0.09621 (1644,7,127,2)

IF SWI5-RIM101 AND ALPHA1'-YBR239c THEN 000 OR 100
100: 0.09621 (1644,7,127,2)

IF m_phosphate_transport_n18-TOS8 AND m_anion_transporters_n10-INO4 THEN 000 OR 100
100: 0.09621 (1644,7,127,2)

IF m_purine_and_pyrimidine_transporters_n10-SKO1 THEN 000 OR 100
100: 0.09621 (1644,7,127,2)

IF SFF'-HIR3 AND SFF-MIG1 THEN 000 OR 010 OR 100
100: 0.09621 (1644,7,127,2)

IF m_other_morphogenetic_activities_n7-RTG1 AND SWI5-RIM101 THEN 000 OR 010 OR 100
100: 0.09621 (1644,7,127,2)

IF SFF'-FKH2 AND m_regulation_of_lipid_fatty-acid_and_isoprenoid_biosynthesis_n16.scn-NDD1 THEN 000 OR 010 OR 100 OR 110
100: 0.09621 (1644,7,127,2)

IF m_metabolism_of_cyclic_and_unusual_nucleotides_n5-NDD1 AND SFF-FKH2 THEN 000 OR 100 OR 110 OR 111
100: 0.09621 (1644,7,127,2)

IF SFF'-FKH2 AND SFF-FKH1 AND SWI5-SWI6 THEN 000 OR 100 OR 110 OR 111
100: 0.09621 (1644,7,127,2)

IF SFF'-FKH1 AND SFF-FKH2 AND SWI5-SWI6 THEN 000 OR 100 OR 110 OR 111
100: 0.09621 (1644,7,127,2)

IF SWI5-INO4 AND m_pentose-phosphate_pathway_n23-MET4 THEN 000 OR 100 OR 110
100: 0.09621 (1644,7,127,2)

IF MCM1'-NDD1 AND MCM1'-SWI4 AND SWI5-SWI6 THEN 000 OR 100 OR 110
100: 0.09621 (1644,7,127,2)

IF m_osmosensing_n6-INO4 AND m_g-proteins_n11-INO4 THEN 000 OR 100 OR 101 OR 110
100: 0.09621 (1644,7,127,2)

IF m_anion_transporters_n22-HAP4 THEN 000 OR 001 OR 010 OR 100
100: 0.09621 (1644,7,127,2)

IF m_other_cell_growth_cell_division_and_dna_synthesis_activities_n14.scn-INO4 THEN 000 OR 010 OR 100 OR 110
100: 0.09621 (1644,7,127,2)

IF SWI5-SWI6 AND m_pentose-phosphate_pathway_n5-SWI4 THEN 000 OR 100 OR 101
100: 0.09621 (1644,7,127,2)

IF m_allantoin_and_allantoate_transporters_n18-FKH2 AND SFF-FKH2 THEN 000 OR 100 OR 110
100: 0.09621 (1644,7,127,2)

IF m_phosphate_transport_n18-GAT1 AND m_phosphate_transport_n18-XBP1 THEN 000 OR 001 OR 010 OR 100
100: 0.09621 (1644,7,127,2)

IF m_deoxyribonucleotide_metabolism_n8-MAL13 THEN 000 OR 010 OR 100 OR 110
100: 0.09621 (1644,7,127,2)

IF m_phosphate_utilization_n7-YAP6 THEN 000 OR 100
100: 0.09621 (1644,7,127,2)

IF SFF-FKH1 AND m_other_morphogenetic_activities_n7-MCM1 THEN 000 OR 100 OR 110 OR 111
100: 0.09621 (1644,7,127,2)

IF MCM1-SWI4 AND MCM1'-MCM1 THEN 000 OR 100 OR 110
100: 0.09621 (1644,7,127,2)

IF MCM1-MCM1 AND MCM1'-SWI4 THEN 000 OR 100 OR 110
100: 0.09621 (1644,7,127,2)

IF m_amino-acid_degradation_n27-GAT1 THEN 000 OR 100
100: 0.09621 (1644,7,127,2)

IF m_amino-acid_transport_n14-SWI6 AND STRE-SWI6 THEN 000 OR 010 OR 100
100: 0.09621 (1644,7,127,2)

IF MCM1-MCM1 AND MCM1'-MCM1 AND MCM1'-SWI4 THEN 000 OR 100 OR 110
100: 0.09621 (1644,7,127,2)

IF m_ion_transporters_n4-GAT1 AND m_purine_and_pyrimidine_transporters_n17-GAT1 THEN 000 OR 001 OR 100
100: 0.09621 (1644,7,127,2)

IF m_phosphate_transport_n18-XBP1 AND m_anion_transporters_n10-INO4 THEN 000 OR 100
100: 0.09621 (1644,7,127,2)

IF m_ion_transporters_n4-INO4 AND m_other_nutritional-response_activities_n10-INO4 THEN 000 OR 010 OR 100
100: 0.09621 (1644,7,127,2)

IF m_phosphate_transport_n18-INO4 AND m_phosphate_transport_n18-TOS8 AND m_anion_transporters_n10-INO4 THEN 000 OR 100
100: 0.09621 (1644,7,127,2)

IF CSRE-SUT1 AND m_glyoxylate_cycle_n11-UME6 THEN 000 OR 010 OR 100
100: 0.09621 (1644,7,127,2)

IF SFF'-FKH1 AND SFF'-FKH2 AND SWI5-INO4 THEN 000 OR 100 OR 110
100: 0.09621 (1644,7,127,2)

IF SFF'-HIR3 AND SFF-FKH1 THEN 000 OR 010 OR 100
100: 0.09621 (1644,7,127,2)

IF m_other_mrna-transcription_activities_n11-SWI6 THEN 000 OR 010 OR 100 OR 101 OR 110 OR 111
010: 0.096697 (1644,21,140,4)

IF m_phosphate_transport_n18-TOS8 THEN 000 OR 001 OR 010 OR 100 OR 110 OR 111
111: 0.096909 (1644,46,19,2)

IF SFF-FKH1 AND SFF-FKH2 THEN 000 OR 010 OR 100 OR 110 OR 111
111: 0.096909 (1644,46,19,2)

IF m_homeostasis_of_other_ions_n30-GAT1 THEN 000 OR 001 OR 010 OR 100 OR 101 OR 110 OR 111
001: 0.097767 (1644,16,55,2)

IF m_glyoxylate_cycle_n8-MTH1 AND m_cell_death_n22-MTH1 THEN 000 OR 001
001: 0.097767 (1644,16,55,2)

IF MCM1'-PDC2 THEN 000 OR 001 OR 010 OR 100 OR 110
001: 0.097767 (1644,16,55,2)

IF SWI5-INO4 AND m_pentose-phosphate_pathway_n23-INO4 THEN 000 OR 001 OR 100 OR 110
001: 0.097767 (1644,16,55,2)

IF m_g-proteins_n11-UME6 THEN 000 OR 001 OR 010 OR 100 OR 110
010: 0.097818 (1644,38,140,6)

IF m_fermentation_n4-FKH2 THEN 000 OR 001 OR 110

110: 0.102947 (1644,8,115,2)

IF SWI5-SKN7 AND m_other_energy_generation_activities_n16-SKN7 THEN 000 OR 001 OR 010 OR 110
110: 0.102947 (1644,8,115,2)

IF m_cell_rescue_defense_cell_death_and_ageing_n20-ACE2 THEN 000 OR 110
110: 0.102947 (1644,8,115,2)

IF MCM1'-SWI4 AND SWI5-SWI6 AND m_cell_death_n22-SWI4 THEN 000 OR 010 OR 101 OR 110
110: 0.102947 (1644,8,115,2)

IF m_glyoxylate_cycle_n8-MTH1 AND m_pentose-phosphate_pathway_n14-MTH1 THEN 000 OR 010 OR 110
110: 0.102947 (1644,8,115,2)

IF m_peroxisomal_organization_n8-SWI6 THEN 000 OR 110
110: 0.102947 (1644,8,115,2)

IF m_organization_of_intracellular_transport_vesicles_n5-SWI4 AND m_cell_death_n22-SWI4 THEN 000 OR 010 OR 101 OR 110
110: 0.102947 (1644,8,115,2)

IF SFF'-HIR1 AND SFF-HOG1 THEN 000 OR 110
110: 0.102947 (1644,8,115,2)

IF m_pentose-phosphate_pathway_n21-MAL33 THEN 000 OR 010 OR 110
110: 0.102947 (1644,8,115,2)

IF Gcr1-SWI6 AND SWI5-SWI6 THEN 000 OR 101 OR 110
110: 0.102947 (1644,8,115,2)

IF m_cell_rescue_defense_cell_death_and_ageing_n20-PHO4 THEN 000 OR 100 OR 110
110: 0.102947 (1644,8,115,2)

IF SFF'-FKH2 AND MCM1'-NDD1 AND SWI5-SWI6 THEN 000 OR 001 OR 100 OR 110 OR 111
110: 0.102947 (1644,8,115,2)

IF MCM1'-NDD1 AND SFF-FKH2 AND SWI5-SWI6 THEN 000 OR 001 OR 100 OR 110 OR 111
110: 0.102947 (1644,8,115,2)

IF SFF-HIR1 AND SFF-HOG1 THEN 000 OR 110
110: 0.102947 (1644,8,115,2)

IF m_anion_transporters_n15-MTH1 AND m_pentose-phosphate_pathway_n14-MTH1 THEN 000 OR 100 OR 110
110: 0.102947 (1644,8,115,2)

IF m_other_mrna-transcription_activities_n20-INO4 AND m_phosphate_transport_n18-INO4 THEN 000 OR 010 OR 100 OR 110
110: 0.102947 (1644,8,115,2)

IF SFF'-FKH1 AND m_g-proteins_n11-INO4 THEN 000 OR 100 OR 110
110: 0.102947 (1644,8,115,2)

IF m_regulation_of_amino-acid_metabolism_n11-SWI6 THEN 000 OR 010 OR 100 OR 110
110: 0.102947 (1644,8,115,2)

IF m_other_cell_growth_cell_division_and_dna_synthesis_activities_n10.scn-SPT23 THEN 000 OR 010 OR 110
110: 0.102947 (1644,8,115,2)

IF m_other_intracellular-transport_activities_n6-UGA3 THEN 000 OR 001 OR 010 OR 110
110: 0.102947 (1644,8,115,2)

IF MCM1'-SWI4 AND mPROTEOL18(m_proteolysis_n18)-MBP1 THEN 000 OR 010 OR 110 OR 111
110: 0.102947 (1644,8,115,2)

IF m_phosphate_transport_n13-GAT1 AND m_pentose-phosphate_pathway_n14-GAT1 THEN 000 OR 010 OR 100 OR 110
110: 0.102947 (1644,8,115,2)

IF m_other_morphogenetic_activities_n8-PDR3 THEN 000 OR 001 OR 110
110: 0.102947 (1644,8,115,2)

IF m_other_morphogenetic_activities_n7-ARR1 AND m_other_morphogenetic_activities_n7-HOG1 THEN 000 OR 010 OR 100 OR 110
110: 0.102947 (1644,8,115,2)

IF m_lipid_and_fatty-acid_transport_n11-MTH1 AND m_deoxyribonucleotide_metabolism_n23-MTH1 THEN 000 OR 010 OR 100 OR 110
110: 0.102947 (1644,8,115,2)

IF MCM1-SWI4 AND SCB-SWI4 THEN 000 OR 010 OR 100 OR 110 OR 111
110: 0.102947 (1644,8,115,2)

IF SWI5-SWI6 AND m_phosphate_transport_n8-SWI4 THEN 000 OR 100 OR 110
110: 0.102947 (1644,8,115,2)

IF STE12-TEC1 THEN 000 OR 010 OR 110
110: 0.102947 (1644,8,115,2)

IF SWI5-INO4 AND m_other_transport_facilitators_n5-INO4 THEN 000 OR 010 OR 100 OR 110
110: 0.102947 (1644,8,115,2)

IF SFF'-MCM1 AND ALPHA1'-UPC2 THEN 000 OR 010 OR 110
110: 0.102947 (1644,8,115,2)

IF MCM1'-NDD1 AND SWI5-INO4 THEN 000 OR 010 OR 100 OR 110
110: 0.102947 (1644,8,115,2)

IF SFF'-FKH2 AND SWI5-INO4 THEN 000 OR 010 OR 100 OR 110
110: 0.102947 (1644,8,115,2)

IF m_metal_ion_transporters_n25-HAP4 THEN 000 OR 100 OR 110
110: 0.102947 (1644,8,115,2)

IF SWI5-SKN7 AND m_anion_transporters_n15-INO4 THEN 000 OR 010 OR 100 OR 110
110: 0.102947 (1644,8,115,2)

IF m_amino-acid_metabolism_n25-SWI6 THEN 000 OR 001 OR 100 OR 110
110: 0.102947 (1644,8,115,2)

IF m_g-proteins_n12-INO4 AND SWI5-INO4 AND m_other_energy_generation_activities_n22-INO4 THEN 000 OR 010 OR 110
110: 0.102947 (1644,8,115,2)

IF m_metal_ion_transporters_n10-SWI6 THEN 000 OR 100 OR 110
100: 0.103408 (1644,15,127,3)

IF SWI5-INO4 AND m_anion_transporters_n15-INO4 THEN 000 OR 001 OR 010 OR 100 OR 110
100: 0.103408 (1644,15,127,3)

IF m_MERE11-SUT1 THEN 000 OR 001 OR 010 OR 100
100: 0.103408 (1644,15,127,3)

IF mRRPE-LEU3 THEN 000 OR 100 OR 110
100: 0.103408 (1644,15,127,3)

IF m_purine_and_pyrimidine_transporters_n17-GAT1 THEN 000 OR 001 OR 100 OR 110
100: 0.103408 (1644,15,127,3)

IF m_g-proteins_n12-INO4 AND m_nutritional_response_pathway_n8-INO4 THEN 000 OR 001 OR 010 OR 100
001: 0.108498 (1644,17,55,2)

IF m_anion_transporters_n17-INO4 THEN 000 OR 001 OR 100 OR 110
001: 0.108498 (1644,17,55,2)

IF m_g-proteins_n12-INO4 AND m_other_energy_generation_activities_n22-INO4 THEN 000 OR 001 OR 010 OR 110
010: 0.109902 (1644,14,140,3)

IF m_other_pheromone_response_activities_n8-SWI6 THEN 000 OR 001 OR 010 OR 110 OR 111
010: 0.109902 (1644,14,140,3)

IF ALPHA1'-KSS1 AND ALPHA1'-STB4 THEN 000 OR 001 OR 010 OR 011
010: 0.109902 (1644,14,140,3)

IF m_utilization_of_vitamins_cofactors_and_prosthetic_groups_n6-YER130C THEN 000 OR 010 OR 101
010: 0.109902 (1644,14,140,3)

IF m_amino-acid_transporters_n11-CUP9 THEN 000 OR 010 OR 100 OR 110 OR 111
010: 0.109902 (1644,14,140,3)

IF mRRPE-IXR1 THEN 000 OR 100 OR 110
110: 0.110629 (1644,17,115,3)

IF m_amino-acid_degradation_n7-NDD1 THEN 000 OR 001 OR 100 OR 110 OR 111
110: 0.110629 (1644,17,115,3)

IF m_lipid_transporters_n8-PHO2 THEN 000 OR 010 OR 110 OR 111
110: 0.110629 (1644,17,115,3)

IF MCM1'-SWI4 AND m_cell_death_n22-SWI4 THEN 000 OR 010 OR 100 OR 101 OR 110 OR 111
110: 0.110629 (1644,17,115,3)

IF SFF'-FKH1 AND SFF'-MCM1 THEN 000 OR 010 OR 100 OR 110 OR 111
010: 0.11073 (1644,22,140,4)

IF m_glyoxylate_cycle_n8-INO4 AND m_RRSE3-INO4 THEN 000 OR 010 OR 100 OR 110
010: 0.113877 (1644,7,140,2)

IF m_lipid_and_fatty-acid_transport_n11-SKN7 AND m_tricarboxylic-acid_pathway_n9-SKN7 THEN 000 OR 010 OR 111
010: 0.113877 (1644,7,140,2)

IF ABF1-ABF1 AND ABF1-WAR1 THEN 000 OR 010 OR 100 OR 110
010: 0.113877 (1644,7,140,2)

IF m_ion_transporters_n14-INO4 THEN 000 OR 001 OR 010
010: 0.113877 (1644,7,140,2)

IF m_g-proteins_n12-INO4 AND SWI5-SKN7 AND SWI5-SWI6 THEN 000 OR 010 OR 110
010: 0.113877 (1644,7,140,2)

IF m_g-proteins_n12-SKN7 AND SWI5-INO4 AND SWI5-SWI6 THEN 000 OR 010 OR 110
010: 0.113877 (1644,7,140,2)

IF m_peroxisomal_transport_n22-RGM1 THEN 000 OR 010
010: 0.113877 (1644,7,140,2)

IF SFF-MIG1 AND m_glyoxylate_cycle_n11-SKO1 THEN 000 OR 010 OR 100
010: 0.113877 (1644,7,140,2)

IF m_nitrogen_and_sulphur_transport_n9-FHL1 AND ALPHA1-GAT3 THEN 000 OR 010
010: 0.113877 (1644,7,140,2)

IF m_phosphate_transport_n13-SKN7 AND m_deoxyribonucleotide_metabolism_n10-SKN7 THEN 000 OR 001 OR 010 OR 100
010: 0.113877 (1644,7,140,2)

IF m_anion_transporters_n4-MTH1 AND m_glycolysis_and_gluconeogenesis_n14-MTH1 THEN 000 OR 010
010: 0.113877 (1644,7,140,2)

IF m_amino-acid_transporters_n11-UME6 AND SCB-UME6 THEN 000 OR 001 OR 010
010: 0.113877 (1644,7,140,2)

IF m_regulation_of_nitrogen_and_sulphur_utilization_n13-UME6 AND CSRE-UME6 THEN 000 OR 001 OR 010 OR 100
010: 0.113877 (1644,7,140,2)

IF SFF'-FKH1 AND ECB-NDD1 THEN 000 OR 010 OR 110 OR 111
010: 0.113877 (1644,7,140,2)

IF RAP1-PDR1 AND RAP1-ZAP1 THEN 000 OR 010
010: 0.113877 (1644,7,140,2)

IF RAP1-FHL1 AND RAP1-ZAP1 THEN 000 OR 010
010: 0.113877 (1644,7,140,2)

IF ALPHA1'-RIM101 AND ALPHA1'-UPC2 THEN 000 OR 010 OR 100
010: 0.113877 (1644,7,140,2)

IF m_other_cation_transporters_n7-SWI6 THEN 000 OR 010 OR 100 OR 110

010: 0.113877 (1644,7,140,2)

IF m_RPE11-RAP1 AND m_other_cation_transporters_n14-RAP1 THEN 000 OR 010
010: 0.113877 (1644,7,140,2)

IF m_regulation_of_amino-acid_metabolism_n7-RAP1 THEN 000 OR 010
010: 0.113877 (1644,7,140,2)

IF SFF-HIR1 AND m_other_morphogenetic_activities_n7-ARR1 THEN 000 OR 010 OR 110
010: 0.113877 (1644,7,140,2)

IF SFF'-HIR1 AND m_other_morphogenetic_activities_n7-ARR1 THEN 000 OR 010 OR 110
010: 0.113877 (1644,7,140,2)

IF SFF-ARG80 AND ALPHA1'-STB4 THEN 000 OR 001 OR 010
010: 0.113877 (1644,7,140,2)

IF m_g-proteins_n11-INO4 AND m_other_nutritional-response_activities_n10-INO2 THEN 000 OR 010
010: 0.113877 (1644,7,140,2)

IF m_g-proteins_n12-INO4 AND m_g-proteins_n12-SKN7 AND SWI5-SWI6 THEN 000 OR 010 OR 110
010: 0.113877 (1644,7,140,2)

IF ALPHA1'-RIM101 AND ALPHA1'-STB4 THEN 000 OR 010
010: 0.113877 (1644,7,140,2)

IF m_anion_transporters_n22-RAP1 AND RAP1-FHL1 THEN 000 OR 010
010: 0.113877 (1644,7,140,2)

IF m_g-proteins_n12-INO4 AND SWI5-INO4 AND m_glycolysis_and_gluconeogenesis_n4-INO4 THEN 000 OR 010 OR 110
010: 0.113877 (1644,7,140,2)

IF ECB-MCM1 AND m_nucleotide_transport_n9-MCM1 THEN 000 OR 010 OR 100 OR 110
010: 0.113877 (1644,7,140,2)

IF m_nitrogen_and_sulphur_utilization_n4-MET32 THEN 000 OR 010
010: 0.113877 (1644,7,140,2)

IF RAP1-FHL1 AND m_nitrogen_and_sulphur_transport_n9-FHL1 THEN 000 OR 010
010: 0.113877 (1644,7,140,2)

IF m_g-proteins_n11-UME6 AND m_LFTE17-UME6 THEN 000 OR 010 OR 100
010: 0.113877 (1644,7,140,2)

IF RAP1-RAP1 AND m_nitrogen_and_sulphur_transport_n9-FHL1 THEN 000 OR 010
010: 0.113877 (1644,7,140,2)

IF m_g-proteins_n12-SKN7 AND m_glycolysis_and_gluconeogenesis_n4-INO4 THEN 000 OR 010 OR 110
010: 0.113877 (1644,7,140,2)

IF m_lysosomal_and_vacuolar_degradation_n3-KSS1 THEN 000 OR 010 OR 100
010: 0.113877 (1644,7,140,2)

IF m_g-proteins_n12-SKN7 AND m_anion_transporters_n15-INO4 THEN 000 OR 010 OR 110 OR 111
010: 0.113877 (1644,7,140,2)

IF m_lipid_and_fatty-acid_transport_n11-SKN7 AND m_other_mrna-transcription_activities_n20-INO4 THEN 000 OR 010 OR 110
010: 0.113877 (1644,7,140,2)

IF m_glyoxylate_cycle_n8-INO4 AND m_lipid_and_fatty-acid_transport_n7-SKN7 THEN 000 OR 010 OR 111
010: 0.113877 (1644,7,140,2)

IF RAP1-RAP1 AND RAP1-ZAP1 THEN 000 OR 010
010: 0.113877 (1644,7,140,2)

IF m_allantoin_and_allantoate_transporters_n6-MAL13 THEN 000 OR 010
010: 0.113877 (1644,7,140,2)

IF m_g-proteins_n11-INO4 AND m_other_nutritional-response_activities_n10-INO4 THEN 000 OR 010
010: 0.113877 (1644,7,140,2)

IF m_g-proteins_n11-INO4 AND m_regulation_of_lipid_fatty-acid_and_isoprenoid_biosynthesis_n12.scn-INO2 THEN 000 OR 010
010: 0.113877 (1644,7,140,2)

IF m_metabolism_of_energy_reserves_n30-MIG3 THEN 000 OR 010
010: 0.113877 (1644,7,140,2)

IF m_other_morphogenetic_activities_n7-GCR2 AND ALPHA1'-STB4 THEN 000 OR 001 OR 010
010: 0.113877 (1644,7,140,2)

IF m_osmosensing_n6-ROX1 THEN 000 OR 001 OR 010 OR 100 OR 110 OR 111
110: 0.114691 (1644,27,115,4)

IF SFF-ARG80 THEN 000 OR 001 OR 010 OR 011 OR 100 OR 110
010: 0.116844 (1644,49,140,7)

IF SFF'-FKH2 AND SFF-FKH1 THEN 000 OR 010 OR 100 OR 110 OR 111
100: 0.11819 (1644,44,127,6)

IF SFF'-FKH1 AND SFF'-FKH2 AND SFF-FKH2 THEN 000 OR 010 OR 100 OR 110 OR 111
100: 0.11819 (1644,44,127,6)

IF SFF'-FKH2 AND SFF-FKH1 AND SFF-FKH2 THEN 000 OR 010 OR 100 OR 110 OR 111
100: 0.11819 (1644,44,127,6)

IF SFF'-FKH1 AND SFF-FKH1 AND SFF-FKH2 THEN 000 OR 010 OR 100 OR 110 OR 111
100: 0.11819 (1644,44,127,6)

IF SFF'-FKH1 AND SFF'-FKH2 AND SFF-FKH1 THEN 000 OR 010 OR 100 OR 110 OR 111
100: 0.11819 (1644,44,127,6)

IF SFF'-FKH1 AND SFF-FKH2 THEN 000 OR 010 OR 100 OR 110 OR 111
100: 0.11819 (1644,44,127,6)

IF SFF'-FKH1 AND SFF'-FKH2 THEN 000 OR 010 OR 100 OR 110 OR 111
111: 0.119212 (1644,52,19,2)

IF m_anion_transporters_n20-RAP1 THEN 000 OR 001 OR 100 OR 110
001: 0.119529 (1644,18,55,2)

IF MCM1'-PDC2 THEN 000 OR 001 OR 010 OR 100 OR 110
100: 0.120393 (1644,16,127,3)

IF m_pentose-phosphate_pathway_n5-MCM1 THEN 000 OR 010 OR 100 OR 101 OR 110 OR 111
100: 0.120393 (1644,16,127,3)

IF m_pentose-phosphate_pathway_n5-UME6 THEN 000 OR 010 OR 100 OR 101 OR 110
100: 0.120393 (1644,16,127,3)

IF SFF'-RLM1 AND m_pentose-phosphate_pathway_n14-AZF1 THEN 000 OR 100 OR 111
100: 0.121978 (1644,8,127,2)

IF m_g-proteins_n12-SKN7 AND m_lipid_and_fatty-acid_transport_n11-SKN7 AND SWI5-SKN7 THEN 000 OR 010 OR 100 OR 110 OR 111
100: 0.121978 (1644,8,127,2)

IF SFF'-MCM1 AND m_other_morphogenetic_activities_n7-MCM1 AND m_pentose-phosphate_pathway_n5-MCM1 THEN 000 OR 010 OR 100 OR 101 OR 110
100: 0.121978 (1644,8,127,2)

IF m_g-proteins_n12-INO4 AND MCM1'-SWI4 THEN 000 OR 010 OR 100 OR 110 OR 111
100: 0.121978 (1644,8,127,2)

IF m_biogenesis_of_chromosome_structure_n18-SIP4 THEN 000 OR 100 OR 101
100: 0.121978 (1644,8,127,2)

IF SFF'-FKH1 AND m_anion_transporters_n15-INO4 THEN 000 OR 010 OR 100 OR 111
100: 0.121978 (1644,8,127,2)

IF SWI5-SWI6 AND m_ion_transporters_n7-SKN7 THEN 000 OR 100 OR 110
100: 0.121978 (1644,8,127,2)

IF m_phosphate_utilization_n9-SKO1 THEN 000 OR 010 OR 100 OR 110
100: 0.121978 (1644,8,127,2)

IF PHO-MET4 THEN 000 OR 010 OR 100 OR 110
100: 0.121978 (1644,8,127,2)

IF RPN4-RPN4 THEN 000 OR 001 OR 010 OR 100
100: 0.121978 (1644,8,127,2)

IF AFT1-MAC1 THEN 000 OR 010 OR 100
100: 0.121978 (1644,8,127,2)

IF m_other_pheromone_response_activities_n8-INO4 AND m_cell_death_n16-INO4 THEN 000 OR 001 OR 010 OR 100 OR 111
100: 0.121978 (1644,8,127,2)

IF mPROTEOL18(m_proteolysis_n18)-SWI6 AND m_amino-acid_transport_n14-SWI6 THEN 000 OR 010 OR 100
100: 0.121978 (1644,8,127,2)

IF SFF'-FKH1 AND SFF'-HIR3 THEN 000 OR 010 OR 100
100: 0.121978 (1644,8,127,2)

IF m_g-proteins_n12-INO4 AND m_organization_of_plasma_membrane_n17-INO4 THEN 000 OR 100 OR 101 OR 110
100: 0.121978 (1644,8,127,2)

IF MCM1'-SWI4 AND SFF-FKH1 THEN 000 OR 100 OR 110
100: 0.121978 (1644,8,127,2)

IF MCM1'-NDD1 AND SWI5-INO4 THEN 000 OR 010 OR 100 OR 110
100: 0.121978 (1644,8,127,2)

IF SFF'-FKH2 AND SWI5-INO4 THEN 000 OR 010 OR 100 OR 110
100: 0.121978 (1644,8,127,2)

IF m_phosphate_transport_n13-GAT1 AND m_pentose-phosphate_pathway_n14-GAT1 THEN 000 OR 010 OR 100 OR 110
100: 0.121978 (1644,8,127,2)

IF m_anion_transporters_n15-GAT1 AND m_pentose-phosphate_pathway_n14-GAT1 THEN 000 OR 100 OR 110
100: 0.121978 (1644,8,127,2)

IF m_other_cell_rescue_activities_n10-MSS11 THEN 000 OR 001 OR 100
100: 0.121978 (1644,8,127,2)

IF m_peroxisomal_organization_n8-YAP1 THEN 000 OR 100
100: 0.121978 (1644,8,127,2)

IF SFF'-FKH1 AND SFF'-FKH2 AND m_other_morphogenetic_activities_n7-MCM1 THEN 000 OR 010 OR 100 OR 110 OR 111
100: 0.121978 (1644,8,127,2)

IF m_nutritional_response_pathway_n3-SKN7 THEN 000 OR 100 OR 111
100: 0.121978 (1644,8,127,2)

IF m_biogenesis_of_cytoskeleton_n5-SKN7 THEN 000 OR 010 OR 100
100: 0.121978 (1644,8,127,2)

IF m_other_transcription_activities_n5-MBP1 THEN 000 OR 001 OR 010 OR 100 OR 101 OR 110
100: 0.121978 (1644,8,127,2)

IF SFF'-FKH2 AND m_osmosensing_n6-SWI4 THEN 000 OR 100 OR 110 OR 111
100: 0.121978 (1644,8,127,2)

IF m_other_transcription_activities_n8-GAT1 THEN 000 OR 100 OR 110
100: 0.121978 (1644,8,127,2)

IF m_anion_transporters_n10-INO4 AND PHO4-CBF1 THEN 000 OR 100
100: 0.121978 (1644,8,127,2)

IF m_anion_transporters_n10-INO4 AND PHO4-INO4 THEN 000 OR 100
100: 0.121978 (1644,8,127,2)

IF SFF'-FKH2 AND MCM1'-SWI4 AND SFF-FKH2 THEN 000 OR 100 OR 110
100: 0.121978 (1644,8,127,2)

IF SWI5-SKN7 AND SWI5-SWI6 AND m_ion_transporters_n7-SKN7 THEN 000 OR 100 OR 110
100: 0.121978 (1644,8,127,2)

IF MCM1-MCM1 AND MCM1-SWI4 THEN 000 OR 100 OR 110
100: 0.121978 (1644,8,127,2)

IF m_cell_death_n22-TOS8 THEN 000 OR 001 OR 010 OR 100 OR 110
001: 0.123856 (1644,37,55,3)

IF m_other_energy_generation_activities_n22-INO4 THEN 000 OR 001 OR 010 OR 100 OR 110
010: 0.125617 (1644,23,140,4)

IF ATRepeat-UME6 THEN 000 OR 010 OR 100 OR 110
110: 0.126288 (1644,18,115,3)

IF m_other_morphogenetic_activities_n7-MCM1 AND m_nucleotide_transport_n9-MCM1 THEN 000 OR 010 OR 100 OR 101 OR 110
110: 0.126496 (1644,9,115,2)

IF SFF'-FKH1 AND m_g-proteins_n12-INO4 THEN 000 OR 010 OR 100 OR 110 OR 111
110: 0.126496 (1644,9,115,2)

IF SWI5-SWI6 AND m_RRSE3-SWI4 THEN 000 OR 010 OR 100 OR 101 OR 110
110: 0.126496 (1644,9,115,2)

IF STE12-STE12 THEN 000 OR 001 OR 010 OR 100 OR 110
110: 0.126496 (1644,9,115,2)

IF m_anion_transporters_n19-OAF1 THEN 000 OR 010 OR 100 OR 110
110: 0.126496 (1644,9,115,2)

IF m_other_mrna-transcription_activities_n20-INO4 AND m_other_mrna-transcription_activities_n20-SKN7 THEN 000 OR 001 OR 010 OR 100 OR 110
110: 0.126496 (1644,9,115,2)

IF m_g-proteins_n11-INO4 AND m_glyoxylate_cycle_n8-INO4 THEN 000 OR 110
110: 0.126496 (1644,9,115,2)

IF m_regulation_of_nitrogen_and_sulphur_utilization_n7-CBF1 THEN 000 OR 010 OR 100 OR 110
110: 0.126496 (1644,9,115,2)

IF m_glycolysis_and_gluconeogenesis_n4-GTS1 THEN 000 OR 010 OR 100 OR 110 OR 111
110: 0.126496 (1644,9,115,2)

IF m_metal_ion_transporters_n10-HAP1 THEN 000 OR 100 OR 110
110: 0.126496 (1644,9,115,2)

IF m_allantoin_and_allantoate_transporters_n18-HAP2 THEN 000 OR 001 OR 100 OR 110
110: 0.126496 (1644,9,115,2)

IF m_g-proteins_n12-SKN7 AND SWI5-SKN7 AND CSRE-SKN7 THEN 000 OR 010 OR 100 OR 110
110: 0.126496 (1644,9,115,2)

IF m_biogenesis_of_chromosome_structure_n18-YDR049W THEN 000 OR 010 OR 100 OR 110
110: 0.126496 (1644,9,115,2)

IF STRE'-MBP1 THEN 000 OR 010 OR 101 OR 110
110: 0.126496 (1644,9,115,2)

IF m_glyoxylate_cycle_n8-INO4 AND m_phosphate_transport_n18-INO4 THEN 000 OR 001 OR 110 OR 111
110: 0.126496 (1644,9,115,2)

IF m_glyoxylate_cycle_n8-INO4 AND SWI5-INO4 AND SWI5-SKN7 THEN 000 OR 010 OR 110
110: 0.126496 (1644,9,115,2)

IF m_other_proteolytic_degradation_n5-PDR1 THEN 000 OR 010 OR 110
110: 0.126496 (1644,9,115,2)

IF m_metabolism_of_cyclic_and_unusual_nucleotides_n5-NDD1 AND SWI5-SWI6 THEN 000 OR 010 OR 110 OR 111
110: 0.126496 (1644,9,115,2)

IF m_g-proteins_n12-INO4 AND m_drug_transporters_n10-INO4 THEN 000 OR 100 OR 110
110: 0.126496 (1644,9,115,2)

IF m_drug_transporters_n9-SWI4 THEN 000 OR 010 OR 110
110: 0.126496 (1644,9,115,2)

IF SWI5-SKN7 AND m_organization_of_chromosome_structure_n17-SKN7 THEN 000 OR 001 OR 100 OR 110 OR 111
110: 0.126496 (1644,9,115,2)

IF SWI5-SWI6 AND m_amino-acid_transporters_n11-SWI4 THEN 000 OR 010 OR 110
110: 0.126496 (1644,9,115,2)

IF MCM1'-SWI4 AND m_nutritional_response_pathway_n7-SWI6 THEN 000 OR 101 OR 110 OR 111
110: 0.126496 (1644,9,115,2)

IF m_deoxyribonucleotide_metabolism_n27-SWI6 THEN 000 OR 110
110: 0.126496 (1644,9,115,2)

IF SFF'-FKH2 AND SFF'-RTG3 THEN 000 OR 010 OR 100 OR 110
110: 0.126496 (1644,9,115,2)

IF m_phosphate_transport_n8-SWI5 THEN 000 OR 001 OR 010 OR 110
110: 0.126496 (1644,9,115,2)

IF m_g-proteins_n12-SKN7 AND m_amino-acid_degradation_n7-SKN7 THEN 000 OR 110 OR 111
110: 0.126496 (1644,9,115,2)

IF m_lipid_and_fatty-acid_transport_n11-GAT1 AND m_anion_transporters_n20-GAT1 THEN 000 OR 110 OR 111
110: 0.126496 (1644,9,115,2)

IF m_glyoxylate_cycle_n8-INO4 AND SWI5-SKN7 THEN 000 OR 010 OR 110
110: 0.126496 (1644,9,115,2)

IF MCM1'-NDD1 AND SWI5-SKN7 THEN 000 OR 010 OR 100 OR 110 OR 111
110: 0.126496 (1644,9,115,2)

IF m_biosynthesis_of_vitamins_cofactors_and_prosthetic_groups_n8-MET4 THEN 000 OR 010 OR 100 OR 110
110: 0.126496 (1644,9,115,2)

IF ECB-YOX1 THEN 000 OR 010 OR 110 OR 111
010: 0.129187 (1644,15,140,3)

IF SFF'-FKH1 AND ALPHA1'-UPC2 THEN 000 OR 010 OR 100 OR 110
010: 0.129187 (1644,15,140,3)

IF SFF'-HIR3 AND m_g-proteins_n11-HAL9 THEN 000 OR 010 OR 100 OR 110
010: 0.129187 (1644,15,140,3)

IF m_MERE11-SUT1 THEN 000 OR 001 OR 010 OR 100
010: 0.129187 (1644,15,140,3)

IF m_rRSE10-MTH1 THEN 000 OR 010 OR 100 OR 110
010: 0.129187 (1644,15,140,3)

IF SFF-GZF3 AND SFF-RPI1 THEN 000 OR 010 OR 110
010: 0.129187 (1644,15,140,3)

IF m_other_morphogenetic_activities_n7-GCR2 THEN 000 OR 001 OR 010 OR 100 OR 110
010: 0.12956 (1644,41,140,6)

IF m_other_energy_generation_activities_n4-UME6 THEN 000 OR 001 OR 010 OR 100 OR 110
001: 0.130828 (1644,19,55,2)

IF m_amino-acid_transport_n18-SKN7 THEN 000 OR 001 OR 010 OR 100 OR 110
001: 0.130828 (1644,19,55,2)

IF SFF-GZF3 THEN 000 OR 001 OR 010 OR 100 OR 110

010: 0.131709 (1644,60,140,8)

IF m_phosphate_transport_n18-PUT3 THEN 000 OR 010 OR 100 OR 111
100: 0.135402 (1644,26,127,4)

IF m_metabolism_of_cyclic_and_unusual_nucleotides_n5-YAP1 THEN 000 OR 001 OR 010 OR 100 OR 110
100: 0.135402 (1644,26,127,4)

IF mRRPE-IXR1 THEN 000 OR 100 OR 110
100: 0.138325 (1644,17,127,3)

IF m_ion_transporters_n4-INO4 THEN 000 OR 010 OR 100
100: 0.138325 (1644,17,127,3)

IF m_ionic_homeostasis_n6-YAP1 THEN 000 OR 100 OR 101 OR 110
100: 0.138325 (1644,17,127,3)

IF m_regulation_of_nitrogen_and_sulphur_utilization_n7-INO4 THEN 000 OR 010 OR 100
100: 0.138325 (1644,17,127,3)

IF SWI5-INO4 AND m_deoxyribonucleotide_metabolism_n10-INO4 THEN 000 OR 001 OR 010 OR 100
100: 0.138325 (1644,17,127,3)

IF SFF-FKH1 AND SFF-FKH2 THEN 000 OR 010 OR 100 OR 110 OR 111
100: 0.138561 (1644,46,127,6)

IF REB1-REB1 THEN 000 OR 001 OR 010 OR 100 OR 110
100: 0.138561 (1644,46,127,6)

IF m_amino-acid_transporters_n11-PHD1 THEN 000 OR 001 OR 010 OR 110 OR 111
001: 0.142366 (1644,20,55,2)

IF m_organization_of_chromosome_structure_n17-INO4 THEN 000 OR 001 OR 100 OR 101 OR 110
001: 0.142366 (1644,20,55,2)

IF m_g-proteins_n12-INO4 AND m_g-proteins_n12-SKN7 THEN 000 OR 001 OR 010 OR 100 OR 110 OR 111
110: 0.14269 (1644,19,115,3)

IF m_g-proteins_n12-INO4 AND m_glyoxylate_cycle_n8-INO4 THEN 000 OR 001 OR 010 OR 100 OR 110 OR 111
110: 0.14269 (1644,19,115,3)

IF m_drug_transporters_n10-INO4 THEN 000 OR 100 OR 110
110: 0.14269 (1644,19,115,3)

IF SFF'-RLM1 AND mRRPE-RLM1 THEN 000 OR 010 OR 110 OR 111
110: 0.14269 (1644,19,115,3)

IF m_nutritional_response_pathway_n7-SWI4 THEN 000 OR 100 OR 101 OR 110 OR 111
110: 0.14269 (1644,19,115,3)

IF m_pentose-phosphate_pathway_n23-MET4 THEN 000 OR 010 OR 100 OR 110
110: 0.14269 (1644,19,115,3)

IF ALPHA1'-KSS1 AND ALPHA1'-STB4 AND ALPHA1'-YER051w THEN 000 OR 001 OR 010
010: 0.143634 (1644,8,140,2)

IF m_pentose-phosphate_pathway_n14-GAT1 AND m_pentose-phosphate_pathway_n14-MTH1 THEN 000 OR 010 OR 100 OR 110
010: 0.143634 (1644,8,140,2)

IF m_MERE4-MTH1 AND m_ion_transporters_n3-MTH1 THEN 000 OR 001 OR 010
010: 0.143634 (1644,8,140,2)

IF ALPHA2-HAP5 THEN 000 OR 010 OR 100
010: 0.143634 (1644,8,140,2)

IF m_organization_of_golgi_n7-MET4 THEN 000 OR 010 OR 100 OR 110
010: 0.143634 (1644,8,140,2)

IF m_g-proteins_n11-INO4 AND SWI5-INO4 AND m_regulation_of_lipid_fatty-acid_and_isoprenoid_biosynthesis_n12.scn-INO4 THEN 000 OR 010 OR 110

010: 0.143634 (1644,8,140,2)

IF RAP1-ARG81 THEN 000 OR 010 OR 100
010: 0.143634 (1644,8,140,2)

IF SFF-FKH1 AND SFF-RPI1 THEN 000 OR 010 OR 100
010: 0.143634 (1644,8,140,2)

IF SFF'-FKH1 AND SFF-RPI1 THEN 000 OR 010 OR 100
010: 0.143634 (1644,8,140,2)

IF m_regulation_of_nitrogen_and_sulphur_utilization_n10-MTH1 THEN 000 OR 010 OR 100
010: 0.143634 (1644,8,140,2)

IF m_anion_transporters_n17-UME6 AND m_glyoxylate_cycle_n11-UME6 THEN 000 OR 010 OR 100
010: 0.143634 (1644,8,140,2)

IF m_other_pheromone_response_activities_n14-FHL1 THEN 000 OR 010 OR 100
010: 0.143634 (1644,8,140,2)

IF m_g-proteins_n12-SKN7 AND SFF-FKH1 THEN 000 OR 010 OR 100 OR 110 OR 111
010: 0.143634 (1644,8,140,2)

IF m_anion_transporters_n22-RAP1 AND RAP1-RAP1 THEN 000 OR 010
010: 0.143634 (1644,8,140,2)

IF m_g-proteins_n12-INO4 AND m_osmosensing_n6-INO4 AND m_RRSE3-INO4 THEN 000 OR 010 OR 100 OR 110
010: 0.143634 (1644,8,140,2)

IF m_other_morphogenetic_activities_n7-GCR2 AND m_other_morphogenetic_activities_n7-MDS3 THEN 000 OR 010 OR 110
010: 0.143634 (1644,8,140,2)

IF SWI5-INO4 AND SWI5-SKN7 AND m_anion_transporters_n15-INO4 THEN 000 OR 010 OR 100 OR 110
010: 0.143634 (1644,8,140,2)

IF mPROTEOL18(m_proteolysis_n18)-SWI6 AND m_other_pheromone_response_activities_n8-SWI6 THEN 000 OR 010 OR 110 OR 111
010: 0.143634 (1644,8,140,2)

IF m_g-proteins_n11-UME6 AND CSRE-UME6 THEN 000 OR 010 OR 100
010: 0.143634 (1644,8,140,2)

IF m_RRSE3-SWI4 AND m_amino-acid_transporters_n11-SWI4 THEN 000 OR 010 OR 110
010: 0.143634 (1644,8,140,2)

IF m_peroxisomal_organization_n6-MAC1 THEN 000 OR 010 OR 100 OR 111
010: 0.143634 (1644,8,140,2)

IF m_other_morphogenetic_activities_n7-RTG1 AND ALPHA1'-KSS1 THEN 000 OR 010 OR 100
010: 0.143634 (1644,8,140,2)

IF m_ion_transporters_n4-INO4 AND m_homeostasis_of_other_ions_n30-INO4 THEN 000 OR 010 OR 100
010: 0.143634 (1644,8,140,2)

IF m_regulation_of_lipid_fatty-acid_and_isoprenoid_biosynthesis_n22.scn-USV1 THEN 000 OR 010
010: 0.143634 (1644,8,140,2)

IF m_lipid_and_fatty-acid_transport_n11-GAT1 AND m_lipid_and_fatty-acid_transport_n11-SKN7 AND m_other_mrna-transcription_activities_n20-SKN7 THEN 000 OR 010 OR 110
010: 0.143634 (1644,8,140,2)

IF m_phosphate_transport_n18-ECM22 AND m_phosphate_transport_n18-GAT1 THEN 000 OR 001 OR 010 OR 110
010: 0.143634 (1644,8,140,2)

IF m_anion_transporters_n17-UME6 AND m_amino-acid_transport_n14-UME6 THEN 000 OR 010
010: 0.143634 (1644,8,140,2)

IF m_utilization_of_vitamins_cofactors_and_prosthetic_groups_n6-GTS1 THEN 000 OR 001 OR 010 OR 101 OR 110
010: 0.143634 (1644,8,140,2)

IF m_lipid_and_fatty-acid_transport_n11-GAT1 AND m_other_mrna-transcription_activities_n20-SKN7 THEN 000 OR 010 OR 110

010: 0.143634 (1644,8,140,2)

IF m_g-proteins_n12-INO4 AND SCB-UME6 THEN 000 OR 001 OR 010
010: 0.143634 (1644,8,140,2)

IF mPROTEOL18(m_proteolysis_n18)-MBP1 AND m_cell_death_n22-SWI4 THEN 000 OR 010 OR 110
010: 0.143634 (1644,8,140,2)

IF SWI5-SKN7 AND m_anion_transporters_n15-INO4 THEN 000 OR 010 OR 100 OR 110
010: 0.143634 (1644,8,140,2)

IF mPROTEOL18(m_proteolysis_n18)-MBP1 AND mPROTEOL18(m_proteolysis_n18)-UME6 THEN 000 OR 010 OR 110
010: 0.143634 (1644,8,140,2)

IF m_g-proteins_n12-INO4 AND SWI5-INO4 AND m_anion_transporters_n10-INO4 THEN 000 OR 001 OR 010
010: 0.143634 (1644,8,140,2)

IF m_g-proteins_n12-SKN7 AND m_other_transport_facilitators_n10-SKN7 THEN 000 OR 010 OR 111
010: 0.143634 (1644,8,140,2)

IF SFF'-RLM1 AND ALPHA1'-YER051w THEN 000 OR 010
010: 0.143634 (1644,8,140,2)

IF SFF'-FKH1 AND MCM1'-SWI4 THEN 000 OR 100 OR 110 OR 111
100: 0.149177 (1644,9,127,2)

IF SFF'-FKH1 AND SFF'-MCM1 AND MCM1'-NDD1 AND SFF-FKH1 THEN 000 OR 100 OR 110 OR 111
100: 0.149177 (1644,9,127,2)

IF m_osmosensing_n6-INO4 AND m_osmosensing_n6-SWI4 THEN 000 OR 010 OR 100 OR 110
100: 0.149177 (1644,9,127,2)

IF SFF'-MCM1 AND MCM1'-NDD1 AND SFF-FKH1 THEN 000 OR 100 OR 110 OR 111
100: 0.149177 (1644,9,127,2)

IF SFF'-FKH2 AND m_metabolism_of_cyclic_and_unusual_nucleotides_n5-NDD1 THEN 000 OR 100 OR 110 OR 111
100: 0.149177 (1644,9,127,2)

IF SFF'-FKH2 AND MCM1'-MCM1 AND MCM1'-NDD1 AND SFF-FKH1 THEN 000 OR 100 OR 110 OR 111
100: 0.149177 (1644,9,127,2)

IF m_phosphate_transport_n18-INO4 AND m_other_nutritional-response_activities_n10-INO4 THEN 000 OR 001 OR 100 OR 111
100: 0.149177 (1644,9,127,2)

IF m_c-compound_and_carbohydrate_metabolism_n8-FKH2 THEN 000 OR 001 OR 100 OR 110
100: 0.149177 (1644,9,127,2)

IF SFF-FKH1 AND SWI5-SKN7 THEN 000 OR 010 OR 100 OR 111
100: 0.149177 (1644,9,127,2)

IF SFF-RTG3 AND SWI5-RIM101 THEN 000 OR 100
100: 0.149177 (1644,9,127,2)

IF SFF'-RTG3 AND SWI5-RIM101 THEN 000 OR 100
100: 0.149177 (1644,9,127,2)

IF m_g-proteins_n12-INO4 AND PHO-INO4 THEN 000 OR 001 OR 010 OR 100
100: 0.149177 (1644,9,127,2)

IF m_g-proteins_n13-ACE2 THEN 000 OR 001 OR 010 OR 100 OR 110
100: 0.149177 (1644,9,127,2)

IF SFF'-FKH1 AND SFF-FKH1 AND SWI5-SKN7 THEN 000 OR 010 OR 100 OR 111
100: 0.149177 (1644,9,127,2)

IF m_osmosensing_n6-INO4 AND m_anion_transporters_n15-INO4 THEN 000 OR 010 OR 100 OR 110
100: 0.149177 (1644,9,127,2)

IF SFF'-MCM1 AND MCM1'-NDD1 AND SFF-FKH1 AND SFF-FKH2 THEN 000 OR 100 OR 110 OR 111
100: 0.149177 (1644,9,127,2)

IF SFF'-RLM1 AND SFF-FKH1 THEN 000 OR 010 OR 100 OR 110 OR 111
100: 0.149177 (1644,9,127,2)

IF MCM1'-SWI4 AND m_RRSE3-SWI4 THEN 000 OR 010 OR 100 OR 101 OR 110
100: 0.149177 (1644,9,127,2)

IF SFF'-FKH1 AND SFF'-RLM1 THEN 000 OR 010 OR 100 OR 110 OR 111
100: 0.149177 (1644,9,127,2)

IF m_RPE68-PDR1 THEN 000 OR 001 OR 010 OR 100
100: 0.149177 (1644,9,127,2)

IF m_g-proteins_n11-INO4 AND m_other_energy_generation_activities_n22-INO4 THEN 000 OR 010 OR 100
100: 0.149177 (1644,9,127,2)

IF CSRE-INO4 AND m_cell_death_n16-INO4 THEN 000 OR 100
100: 0.149177 (1644,9,127,2)

IF SFF'-FKH1 AND m_other_morphogenetic_activities_n7-MCM1 THEN 000 OR 010 OR 100 OR 110 OR 111
100: 0.149177 (1644,9,127,2)

IF m_phosphate_transport_n18-INO4 AND m_other_pheromone_response_activities_n8-INO4 THEN 000 OR 010 OR 100 OR 111
100: 0.149177 (1644,9,127,2)

IF m_RPE17-PDR1 THEN 000 OR 001 OR 010 OR 100
100: 0.149177 (1644,9,127,2)

IF MCM1'-SWI4 AND SFF-FKH2 THEN 000 OR 100 OR 110
100: 0.149177 (1644,9,127,2)

IF m_other_energy_generation_activities_n4-ABF1 THEN 000 OR 010
010: 0.149568 (1644,16,140,3)

IF AFT1-RAP1 THEN 000 OR 010 OR 100 OR 101
010: 0.149568 (1644,16,140,3)

IF m_glycolysis_and_gluconeogenesis_n14-MTH1 THEN 000 OR 010 OR 110
010: 0.149568 (1644,16,140,3)

IF SFF'-HIR3 AND SFF'-RTG3 THEN 000 OR 001 OR 010 OR 110
010: 0.149568 (1644,16,140,3)

IF m_other_signal-transduction_activities_n13-REB1 THEN 000 OR 010 OR 100 OR 110
010: 0.149568 (1644,16,140,3)

IF SWI5-INO4 AND SWI5-SWI6 THEN 000 OR 010 OR 100 OR 110
010: 0.149568 (1644,16,140,3)

IF m_other_proteolytic_degradation_n8-INO2 THEN 000 OR 010 OR 100 OR 110
110: 0.151158 (1644,10,115,2)

IF m_lipid_transporters_n8-RPI1 THEN 000 OR 001 OR 010 OR 110
110: 0.151158 (1644,10,115,2)

IF SFF'-FKH1 AND SFF'-RTG3 THEN 000 OR 010 OR 100 OR 110
110: 0.151158 (1644,10,115,2)

IF m_g-proteins_n12-INO4 AND SWI5-INO4 AND m_anion_transporters_n15-INO4 THEN 000 OR 001 OR 010 OR 100 OR 110
110: 0.151158 (1644,10,115,2)

IF SWI5-SWI6 AND m_nutritional_response_pathway_n7-SWI6 THEN 000 OR 010 OR 101 OR 110 OR 111
110: 0.151158 (1644,10,115,2)

IF SFF'-FKH2 AND m_g-proteins_n12-INO4 THEN 000 OR 010 OR 110
110: 0.151158 (1644,10,115,2)

IF ndt80(MSE)-YDR049W THEN 000 OR 100 OR 110
110: 0.151158 (1644,10,115,2)

IF m_other_pheromone_response_activities_n8-FKH2 THEN 000 OR 001 OR 100 OR 110
110: 0.151158 (1644,10,115,2)

IF m_MERE4-MTH1 AND m_pentose-phosphate_pathway_n14-MTH1 THEN 000 OR 010 OR 100 OR 110
110: 0.151158 (1644,10,115,2)

IF m_other_mrna-transcription_activities_n20-INO4 AND m_other_mrna-transcription_activities_n20-RAP1 THEN 000 OR 001 OR 100 OR 110
110: 0.151158 (1644,10,115,2)

IF m_other_transport_facilitators_n5-INO4 THEN 000 OR 010 OR 100 OR 110
110: 0.151158 (1644,10,115,2)

IF Gcr1-SWI6 THEN 000 OR 100 OR 101 OR 110
110: 0.151158 (1644,10,115,2)

IF m_organization_of_cell_wall_n10-SKN7 THEN 000 OR 110
110: 0.151158 (1644,10,115,2)

IF SFF'-FKH2 AND m_pentose-phosphate_pathway_n14-FKH1 THEN 000 OR 100 OR 110 OR 111
110: 0.151158 (1644,10,115,2)

IF m_drug_transporters_n10-INO4 AND SWI5-INO4 THEN 000 OR 100 OR 110
110: 0.151158 (1644,10,115,2)

IF m_nitrogen_and_sulphur_metabolism_n17-SKN7 AND m_nitrogen_and_sulphur_metabolism_n17-XBP1 THEN 000 OR 001 OR 110
110: 0.151158 (1644,10,115,2)

IF m_anion_transporters_n17-UME6 THEN 000 OR 001 OR 010 OR 100
001: 0.154115 (1644,21,55,2)

IF m_anion_transporters_n22-RAP1 THEN 000 OR 001 OR 010 OR 100 OR 110
001: 0.154115 (1644,21,55,2)

IF m_amino-acid_transport_n20-INO4 THEN 000 OR 001 OR 010 OR 100 OR 110
001: 0.154115 (1644,21,55,2)

IF m_meiosis_n3-UME6 THEN 000 OR 001 OR 010 OR 100 OR 110
001: 0.155061 (1644,41,55,3)

IF m_g-proteins_n12-SKN7 AND m_lipid_and_fatty-acid_transport_n11-SKN7 THEN 000 OR 010 OR 100 OR 110 OR 111
100: 0.1571 (1644,18,127,3)

IF m_allantoin_and_allantoate_transporters_n18-YAP1 THEN 000 OR 100 OR 110
100: 0.1571 (1644,18,127,3)

IF ATRepeat-UME6 THEN 000 OR 010 OR 100 OR 110
100: 0.1571 (1644,18,127,3)

IF m_g-proteins_n12-INO4 AND SWI5-INO4 THEN 000 OR 001 OR 010 OR 100 OR 110
001: 0.163227 (1644,42,55,3)

IF MCM1-MCM1 AND MCM1'-MCM1 THEN 000 OR 010 OR 100 OR 110 OR 111
100: 0.165101 (1644,28,127,4)

IF m_g-proteins_n12-INO4 AND SWI5-INO4 THEN 000 OR 001 OR 010 OR 100 OR 110
110: 0.165458 (1644,42,115,5)

IF m_pentose-phosphate_pathway_n23-INO4 THEN 000 OR 001 OR 010 OR 100 OR 110
001: 0.166048 (1644,22,55,2)

IF m_regulation_of_lipid_fatty-acid_and_isoprenoid_biosynthesis_n12.scn-DOT6 THEN 000 OR 010 OR 111
010: 0.1709 (1644,17,140,3)

IF m_amino-acid_transporters_n11-AZF1 THEN 000 OR 010 OR 111
010: 0.1709 (1644,17,140,3)

IF m_other_transport_facilitators_n10-SWI6 THEN 000 OR 010 OR 110
010: 0.1709 (1644,17,140,3)

IF m_g-proteins_n12-SKN7 AND SWI5-SWI6 THEN 000 OR 010 OR 100 OR 110 OR 111

010: 0.1709 (1644,17,140,3)

IF SFF'-MCM1 AND SFF-FKH1 THEN 000 OR 010 OR 100 OR 110 OR 111
010: 0.1709 (1644,17,140,3)

IF m_RPE57-SKN7 THEN 000 OR 010 OR 111
010: 0.1709 (1644,17,140,3)

IF m_amino-acid_degradation_n8-MTH1 THEN 000 OR 001 OR 010 OR 100 OR 110
010: 0.1709 (1644,17,140,3)

IF SWI5-SKN7 AND m_RPE57-SKN7 THEN 000 OR 010
010: 0.174773 (1644,9,140,2)

IF RAP1-ARO80 THEN 000 OR 001 OR 010
010: 0.174773 (1644,9,140,2)

IF m_MERE4-MTH1 AND m_regulation_of_lipid_fatty-acid_and_isoprenoid_biosynthesis_n8.scn-MTH1 THEN 000 OR 010 OR 110
010: 0.174773 (1644,9,140,2)

IF STE12-IXR1 THEN 000 OR 010 OR 100 OR 110
010: 0.174773 (1644,9,140,2)

IF SFF-FKH1 AND m_lipid_and_fatty-acid_transport_n11-SKN7 THEN 000 OR 010 OR 100 OR 110
010: 0.174773 (1644,9,140,2)

IF m_osmosensing_n6-INO4 AND m_RRSE3-INO4 THEN 000 OR 010 OR 100 OR 110
010: 0.174773 (1644,9,140,2)

IF SFF'-RLM1 AND SFF-FKH1 THEN 000 OR 010 OR 100 OR 110 OR 111
010: 0.174773 (1644,9,140,2)

IF SFF'-FKH1 AND SFF'-RLM1 THEN 000 OR 010 OR 100 OR 110 OR 111
010: 0.174773 (1644,9,140,2)

IF m_cytokinesis_n10-UME1 THEN 000 OR 010 OR 110
010: 0.174773 (1644,9,140,2)

IF m_mitochondrial_biogenesis_n5-GAT1 AND m_phosphate_transport_n13-GAT1 THEN 000 OR 001 OR 010
010: 0.174773 (1644,9,140,2)

IF m_g-proteins_n12-NRG1 AND m_g-proteins_n12-SKN7 THEN 000 OR 010 OR 100
010: 0.174773 (1644,9,140,2)

IF m_other_morphogenetic_activities_n8-SFP1 AND ALPHA1'-SFP1 THEN 000 OR 010
010: 0.174773 (1644,9,140,2)

IF SFF'-FKH1 AND ECB-FKH2 THEN 000 OR 010 OR 110 OR 111
010: 0.174773 (1644,9,140,2)

IF m_other_morphogenetic_activities_n7-MCM1 AND m_nucleotide_transport_n9-MCM1 THEN 000 OR 010 OR 100 OR 101 OR 110
010: 0.174773 (1644,9,140,2)

IF SFF-FKH1 AND SWI5-SKN7 THEN 000 OR 010 OR 100 OR 111
010: 0.174773 (1644,9,140,2)

IF ECB-MCM1 AND m_other_morphogenetic_activities_n7-MCM1 THEN 000 OR 010 OR 110
010: 0.174773 (1644,9,140,2)

IF SFF'-FKH1 AND m_g-proteins_n12-SKN7 THEN 000 OR 010 OR 100 OR 111
010: 0.174773 (1644,9,140,2)

IF SFF'-HIR3 AND m_other_morphogenetic_activities_n7-GCR2 THEN 000 OR 010 OR 100
010: 0.174773 (1644,9,140,2)

IF m_g-proteins_n11-INO4 AND m_amino-acid_transport_n20-INO4 THEN 000 OR 001 OR 010 OR 110
010: 0.174773 (1644,9,140,2)

IF m_g-proteins_n12-SKN7 AND SCB-AZF1 THEN 000 OR 010 OR 100 OR 111
010: 0.174773 (1644,9,140,2)

IF SFF'-FKH2 AND SFF'-RTG3 THEN 000 OR 010 OR 100 OR 110
010: 0.174773 (1644,9,140,2)

IF m_biosynthesis_of_vitamins_cofactors_and_prosthetic_groups_n8-MET4 THEN 000 OR 010 OR 100 OR 110
010: 0.174773 (1644,9,140,2)

IF m_lipid_and_fatty-acid_binding_n14-GAT1 THEN 000 OR 010 OR 100
010: 0.174773 (1644,9,140,2)

IF m_g-proteins_n12-SKN7 AND SCB-SWI4 THEN 000 OR 010 OR 110 OR 111
010: 0.174773 (1644,9,140,2)

IF m_other_morphogenetic_activities_n7-MDS3 AND ALPHA1'-STB4 AND ALPHA1'-YER051w THEN 000 OR 001 OR 010 OR 100
010: 0.174773 (1644,9,140,2)

IF m_other_signal-transduction_activities_n13-MAC1 THEN 000 OR 001 OR 010
010: 0.174773 (1644,9,140,2)

IF m_amino-acid_degradation_n24-CIN5 THEN 000 OR 010
010: 0.174773 (1644,9,140,2)

IF m_other_proteolytic_degradation_n5-PDR1 THEN 000 OR 010 OR 110
010: 0.174773 (1644,9,140,2)

IF m_other_mrna-transcription_activities_n20-INO4 AND m_other_mrna-transcription_activities_n20-SKN7 THEN 000 OR 001 OR 010 OR 100 OR 110
010: 0.174773 (1644,9,140,2)

IF RAP1-ZAP1 THEN 000 OR 010
010: 0.174773 (1644,9,140,2)

IF m_osmosensing_n6-SWI4 AND SCB-SWI4 THEN 000 OR 010 OR 110 OR 111
010: 0.174773 (1644,9,140,2)

IF m_g-proteins_n12-INO4 AND m_ion_transporters_n4-INO4 THEN 000 OR 010 OR 100
010: 0.174773 (1644,9,140,2)

IF m_g-proteins_n11-INO4 AND m_other_energy_generation_activities_n22-INO4 THEN 000 OR 010 OR 100
010: 0.174773 (1644,9,140,2)

IF SWI5-INO4 AND m_regulation_of_lipid_fatty-acid_and_isoprenoid_biosynthesis_n12.scn-INO2 THEN 000 OR 010
010: 0.174773 (1644,9,140,2)

IF SWI5-SWI6 AND m_amino-acid_transporters_n11-SWI4 THEN 000 OR 010 OR 110
010: 0.174773 (1644,9,140,2)

IF m_other_transport_facilitators_n5-SWI6 THEN 000 OR 010 OR 100 OR 110
010: 0.174773 (1644,9,140,2)

IF m_lipid_and_fatty-acid_binding_n15-MSN4 THEN 000 OR 010
010: 0.174773 (1644,9,140,2)

IF m_deoxyribonucleotide_metabolism_n27-MTH1 THEN 000 OR 010 OR 100
010: 0.174773 (1644,9,140,2)

IF SWI5-SKN7 AND m_other_transport_facilitators_n10-SKN7 THEN 000 OR 010 OR 111
010: 0.174773 (1644,9,140,2)

IF m_g-proteins_n12-SKN7 AND SWI5-SKN7 AND m_amino-acid_transporters_n11-SKN7 THEN 000 OR 001 OR 010 OR 110
010: 0.174773 (1644,9,140,2)

IF m_RPE17-RGM1 AND ALPHA1-GAT3 THEN 000 OR 001 OR 010
010: 0.174773 (1644,9,140,2)

IF mPROTEOL18(m_proteolysis_n18)-SWI6 AND m_cell_death_n22-SWI4 THEN 000 OR 010 OR 110
010: 0.174773 (1644,9,140,2)

IF m_RRSE3-SWI4 AND SCB-SWI4 THEN 000 OR 010 OR 100 OR 110
010: 0.174773 (1644,9,140,2)

IF m_g-proteins_n12-INO4 AND SWI5-INO4 AND m_other_mrna-transcription_activities_n20-INO4 THEN 000 OR 001 OR 010 OR 110
010: 0.174773 (1644,9,140,2)

IF m_anion_transporters_n19-OAF1 THEN 000 OR 010 OR 100 OR 110
010: 0.174773 (1644,9,140,2)

IF STE12-STE12 THEN 000 OR 001 OR 010 OR 100 OR 110
010: 0.174773 (1644,9,140,2)

IF m_metabolism_of_cyclic_and_unusual_nucleotides_n5-HAC1 THEN 000 OR 010 OR 100
010: 0.174773 (1644,9,140,2)

IF m_anion_transporters_n4-MTH1 THEN 000 OR 001 OR 010
010: 0.174809 (1644,26,140,4)

IF SFF-GZF3 THEN 000 OR 001 OR 010 OR 100 OR 110
100: 0.175442 (1644,60,127,7)

IF RAP1-RAP1 THEN 000 OR 001 OR 010 OR 100 OR 110
001: 0.17611 (1644,66,55,4)

IF m_drug_transporters_n10-MET4 THEN 000 OR 010 OR 100 OR 110 OR 111
100: 0.176612 (1644,19,127,3)

IF m_metal_ion_transporters_n6-YAP1 THEN 000 OR 100 OR 101 OR 110
100: 0.176612 (1644,19,127,3)

IF m_other_pheromone_response_activities_n12-PHD1 THEN 000 OR 001 OR 010 OR 100 OR 110 OR 111
100: 0.176612 (1644,19,127,3)

IF m_drug_transporters_n10-INO4 THEN 000 OR 100 OR 110
100: 0.176612 (1644,19,127,3)

IF m_allantoin_and_allantoate_transporters_n7-RDS1 THEN 000 OR 100 OR 111
100: 0.176612 (1644,19,127,3)

IF SFF'-HIR1 AND m_other_morphogenetic_activities_n7-RCS1 THEN 000 OR 010 OR 100 OR 110
110: 0.176666 (1644,11,115,2)

IF m_g-proteins_n12-INO4 AND m_glyoxylate_cycle_n8-INO4 AND SWI5-INO4 THEN 000 OR 010 OR 100 OR 110
110: 0.176666 (1644,11,115,2)

IF m_g-proteins_n12-INO4 AND SWI5-INO4 AND SWI5-SWI6 THEN 000 OR 010 OR 100 OR 110
110: 0.176666 (1644,11,115,2)

IF m_g-proteins_n12-INO4 AND SWI5-SWI6 THEN 000 OR 010 OR 100 OR 110
110: 0.176666 (1644,11,115,2)

IF m_anion_transporters_n20-MTH1 THEN 000 OR 010 OR 100 OR 110 OR 111
110: 0.176666 (1644,11,115,2)

IF m_pentose-phosphate_pathway_n7-GAT1 THEN 000 OR 100 OR 110
110: 0.176666 (1644,11,115,2)

IF SWI5-SWI6 AND m_cell_death_n22-SWI4 THEN 000 OR 010 OR 100 OR 101 OR 110
110: 0.176666 (1644,11,115,2)

IF m_g-proteins_n11-INO4 AND m_phosphate_transport_n18-INO4 THEN 000 OR 010 OR 100 OR 110
110: 0.176666 (1644,11,115,2)

IF m_breakdown_of_lipids_fatty_acids_and_isoprenoids_n8-INO4 THEN 000 OR 010 OR 100 OR 110
110: 0.176666 (1644,11,115,2)

IF m_intracellular_communication_n4-SWI5 THEN 000 OR 001 OR 110
110: 0.176666 (1644,11,115,2)

IF m_g-proteins_n12-SKN7 AND m_other_protein-destination_activities_n7-SKN7 THEN 000 OR 001 OR 110 OR 111
110: 0.176666 (1644,11,115,2)

IF m_lipid_and_fatty-acid_transport_n11-MTH1 AND m_lipid_and_fatty-acid_transport_n11-SKN7 THEN 000 OR 110 OR 111
110: 0.176666 (1644,11,115,2)

IF m_lipid_and_fatty-acid_transport_n11-MTH1 AND m_anion_transporters_n15-MTH1 THEN 000 OR 100 OR 110
110: 0.176666 (1644,11,115,2)

IF m_other_transport_facilitators_n5-CIN5 THEN 000 OR 010 OR 100 OR 110
110: 0.176666 (1644,11,115,2)

IF m_other_pheromone_response_activities_n8-MAC1 THEN 000 OR 010 OR 100 OR 110 OR 111
110: 0.176666 (1644,11,115,2)

IF m_lipid_and_fatty-acid_transport_n11-SWI5 THEN 000 OR 001 OR 010 OR 110 OR 111
110: 0.177415 (1644,21,115,3)

IF MCM1'-SWI4 AND SCB-SWI4 THEN 000 OR 010 OR 100 OR 110 OR 111
110: 0.177415 (1644,21,115,3)

IF m_g-proteins_n11-GCN4 THEN 000 OR 010 OR 100 OR 110
110: 0.177415 (1644,21,115,3)

IF SWI5-INO4 AND SWI5-SKN7 THEN 000 OR 001 OR 010 OR 100 OR 110
110: 0.177415 (1644,21,115,3)

IF m_deoxyribonucleotide_metabolism_n23-MTH1 THEN 000 OR 001 OR 010 OR 100 OR 110
110: 0.177415 (1644,21,115,3)

IF m_glyoxylate_cycle_n8-UME6 AND CSRE-UME6 THEN 000 OR 001 OR 100 OR 110
100: 0.177437 (1644,10,127,2)

IF SFF'-FKH2 AND MCM1-FKH2 AND SFF-FKH1 THEN 000 OR 100 OR 110 OR 111
100: 0.177437 (1644,10,127,2)

IF m_trna_processing_n6-ARO80 THEN 000 OR 010 OR 100 OR 110
100: 0.177437 (1644,10,127,2)

IF SWI5-SKN7 AND m_cell_death_n16-SKN7 THEN 000 OR 001 OR 100 OR 110
100: 0.177437 (1644,10,127,2)

IF SFF'-FKH2 AND MCM1'-MCM1 AND SFF-FKH1 THEN 000 OR 100 OR 110 OR 111
100: 0.177437 (1644,10,127,2)

IF SFF'-FKH1 AND MCM1'-MCM1 AND SFF-FKH2 THEN 000 OR 100 OR 110 OR 111
100: 0.177437 (1644,10,127,2)

IF m_phosphate_transport_n18-INO4 AND m_phosphate_transport_n18-XBP1 THEN 000 OR 001 OR 100 OR 110 OR 111
100: 0.177437 (1644,10,127,2)

IF m_stress_response_n24-MTH1 THEN 000 OR 010 OR 100
100: 0.177437 (1644,10,127,2)

IF MIG1-SKO1 THEN 000 OR 001 OR 010 OR 100 OR 110
100: 0.177437 (1644,10,127,2)

IF m_amino-acid_metabolism_n14-SKN7 THEN 000 OR 100 OR 110
100: 0.177437 (1644,10,127,2)

IF SFF-FKH1 AND SWI5-SWI6 THEN 000 OR 100 OR 110 OR 111
100: 0.177437 (1644,10,127,2)

IF m_other_nucleotide-metabolism_activities_n17-WAR1 THEN 000 OR 001 OR 100 OR 110
100: 0.177437 (1644,10,127,2)

IF m_RPE17-SFP1 THEN 000 OR 100
100: 0.177437 (1644,10,127,2)

IF ALPHA2-RCO1 THEN 000 OR 001 OR 010 OR 100 OR 110
100: 0.177437 (1644,10,127,2)

IF SFF'-RLM1 AND SFF-HIR1 THEN 000 OR 100 OR 110

100: 0.177437 (1644,10,127,2)

IF m_ion_transporters_n4-INO4 AND m_anion_transporters_n10-INO4 THEN 000 OR 010 OR 100
100: 0.177437 (1644,10,127,2)

IF SFF-RPI1 AND SFF-YER184C THEN 000 OR 010 OR 100
100: 0.177437 (1644,10,127,2)

IF m_MERE16-MTH1 THEN 000 OR 001 OR 100 OR 110
100: 0.177437 (1644,10,127,2)

IF m_g-proteins_n11-INO4 AND m_pentose-phosphate_pathway_n23-INO4 THEN 000 OR 001 OR 010 OR 100 OR 110
100: 0.177437 (1644,10,127,2)

IF m_lipid_and_fatty-acid_transport_n11-SKN7 AND m_LFTE17-SKN7 THEN 000 OR 010 OR 100 OR 110 OR 111
100: 0.177437 (1644,10,127,2)

IF MIG1-YAP6 THEN 000 OR 010 OR 011 OR 100 OR 110 OR 111
100: 0.177437 (1644,10,127,2)

IF SFF'-FKH1 AND SFF'-FKH2 AND MCM1'-MCM1 AND SFF-FKH2 THEN 000 OR 100 OR 110 OR 111
100: 0.177437 (1644,10,127,2)

IF SFF'-FKH1 AND MCM1'-MCM1 AND SFF-FKH1 AND SFF-FKH2 THEN 000 OR 100 OR 110 OR 111
100: 0.177437 (1644,10,127,2)

IF m_metabolism_of_energy_reserves_n27-PHO4 THEN 000 OR 001 OR 010 OR 100
100: 0.177437 (1644,10,127,2)

IF SFF'-FKH2 AND MCM1-FKH2 AND SFF-FKH1 AND SFF-FKH2 THEN 000 OR 100 OR 110 OR 111
100: 0.177437 (1644,10,127,2)

IF MCM1'-MCM1 AND MCM1'-NDD1 AND SFF-FKH1 THEN 000 OR 100 OR 110 OR 111
100: 0.177437 (1644,10,127,2)

IF SFF'-FKH2 AND MCM1'-SWI4 THEN 000 OR 010 OR 100 OR 110
100: 0.177437 (1644,10,127,2)

IF m_other_morphogenetic_activities_n7-MCM1 AND m_pentose-phosphate_pathway_n5-MCM1 THEN 000 OR 010 OR 100 OR 101 OR 110
100: 0.177437 (1644,10,127,2)

IF m_other_morphogenetic_activities_n7-RTG1 AND ALPHA1'-RIM101 THEN 000 OR 010 OR 100
100: 0.177437 (1644,10,127,2)

IF SFF'-RLM1 AND SFF-FKH2 THEN 000 OR 100 OR 110 OR 111
100: 0.177437 (1644,10,127,2)

IF SFF'-HIR3 AND SFF-HIR1 THEN 000 OR 010 OR 100 OR 101
100: 0.177437 (1644,10,127,2)

IF SFF'-FKH1 AND MCM1-FKH2 AND SFF-FKH2 THEN 000 OR 100 OR 110 OR 111
100: 0.177437 (1644,10,127,2)

IF m_metal_ion_transporters_n10-SKN7 AND m_metal_ion_transporters_n10-SWI6 THEN 000 OR 100 OR 110
100: 0.177437 (1644,10,127,2)

IF SFF'-FKH1 AND SWI5-SWI6 THEN 000 OR 100 OR 110 OR 111
100: 0.177437 (1644,10,127,2)

IF m_morphogenesis_n5-SWI6 THEN 000 OR 010 OR 100 OR 110
100: 0.177437 (1644,10,127,2)

IF m_pentose-phosphate_pathway_n14-GAT1 AND m_phosphate_transport_n18-GAT1 THEN 000 OR 010 OR 100
100: 0.177437 (1644,10,127,2)

IF m_g-proteins_n12-INO4 AND m_g-proteins_n11-INO4 THEN 000 OR 001 OR 010 OR 100 OR 101 OR 110
001: 0.178141 (1644,23,55,2)

IF m_cell_death_n8-XBP1 THEN 000 OR 001 OR 010 OR 110
001: 0.178141 (1644,23,55,2)

IF m_nutritional_response_pathway_n8-SKN7 THEN 000 OR 001 OR 010 OR 110
001: 0.178141 (1644,23,55,2)

IF m_g-proteins_n11-INO4 AND SWI5-INO4 THEN 000 OR 001 OR 010 OR 100 OR 110
001: 0.178141 (1644,23,55,2)

IF m_amino-acid_transport_n20-MTH1 THEN 000 OR 001 OR 010 OR 100 OR 110
001: 0.178141 (1644,23,55,2)

IF m_allantoin_and_allantoate_transporters_n7-STE12 THEN 000 OR 001 OR 010 OR 100 OR 110
001: 0.178141 (1644,23,55,2)

IF MCM1-MCM1 THEN 000 OR 010 OR 100 OR 110 OR 111
100: 0.180763 (1644,29,127,4)

IF m_other_morphogenetic_activities_n7-MCM1 THEN 000 OR 001 OR 010 OR 011 OR 100 OR 101 OR 110 OR 111
100: 0.183785 (1644,50,127,6)

IF m_other_proteolytic_degradation_n2-MTH1 THEN 000 OR 001 OR 010 OR 100 OR 110
001: 0.190371 (1644,24,55,2)

IF m_other_morphogenetic_activities_n7-STP1 THEN 000 OR 001 OR 010 OR 100 OR 110
010: 0.192505 (1644,27,140,4)

IF m_regulation_of_nitrogen_and_sulphur_utilization_n7-RAP1 THEN 000 OR 010 OR 100 OR 110
010: 0.193041 (1644,18,140,3)

IF SWI5-KSS1 AND SWI5-RIM101 THEN 000 OR 010 OR 100 OR 110
010: 0.193041 (1644,18,140,3)

IF m_other_energy_generation_activities_n12-FKH1 THEN 000 OR 010 OR 100 OR 110
110: 0.195584 (1644,22,115,3)

IF SFF'-FKH2 AND SFF'-MCM1 AND SFF-FKH2 THEN 000 OR 010 OR 011 OR 100 OR 110 OR 111
100: 0.196754 (1644,20,127,3)

IF m_osmosensing_n6-SWI4 THEN 000 OR 010 OR 100 OR 101 OR 110 OR 111
100: 0.196754 (1644,20,127,3)

IF m_regulation_of_lipid_fatty-acid_and_isoprenoid_biosynthesis_n22.scn-INO4 THEN 000 OR 010 OR 100 OR 111
100: 0.196754 (1644,20,127,3)

IF SFF'-MCM1 AND SFF-FKH2 THEN 000 OR 010 OR 011 OR 100 OR 110 OR 111
100: 0.196754 (1644,20,127,3)

IF m_amino-acid_transport_n14-SWI6 THEN 000 OR 010 OR 011 OR 100 OR 110
100: 0.196754 (1644,20,127,3)

IF REB1-REB1 THEN 000 OR 001 OR 010 OR 100 OR 110
001: 0.197112 (1644,46,55,3)

IF m_phosphate_transport_n5-INO4 THEN 000 OR 001 OR 010 OR 100 OR 110
110: 0.202782 (1644,12,115,2)

IF SFF-FKH1 AND SWI5-INO4 THEN 000 OR 010 OR 100 OR 110
110: 0.202782 (1644,12,115,2)

IF ndt80(MSE)-YER130C THEN 000 OR 100 OR 110
110: 0.202782 (1644,12,115,2)

IF SFF-HIR1 AND m_other_morphogenetic_activities_n7-RCS1 THEN 000 OR 010 OR 100 OR 110
110: 0.202782 (1644,12,115,2)

IF m_lipid_and_fatty-acid_transport_n11-SKN7 AND m_other_mrna-transcription_activities_n20-SKN7 THEN 000 OR 010 OR 110
110: 0.202782 (1644,12,115,2)

IF m_metal_ion_transporters_n17-FKH2 THEN 000 OR 010 OR 110
110: 0.202782 (1644,12,115,2)

IF MCM1'-SWI4 AND mPROTEOL18(m_proteolysis_n18)-SWI6 THEN 000 OR 010 OR 100 OR 110 OR 111
110: 0.202782 (1644,12,115,2)

IF m_anion_transporters_n32-INO4 THEN 000 OR 100 OR 101 OR 110
110: 0.202782 (1644,12,115,2)

IF m_nutritional_response_pathway_n7-ACE2 THEN 000 OR 010 OR 100 OR 110
110: 0.202782 (1644,12,115,2)

IF m_other_energy_generation_activities_n16-SKN7 THEN 000 OR 001 OR 010 OR 110
110: 0.202782 (1644,12,115,2)

IF CCA-SFP1 THEN 000 OR 110
110: 0.202782 (1644,12,115,2)

IF m_other_morphogenetic_activities_n7-FZF1 AND m_other_morphogenetic_activities_n7-GCR2 THEN 000 OR 010 OR 110
110: 0.202782 (1644,12,115,2)

IF m_metabolism_of_cyclic_and_unusual_nucleotides_n5-NDD1 AND SWI5-SKN7 THEN 000 OR 010 OR 110 OR 111
110: 0.202782 (1644,12,115,2)

IF m_other_cell_growth_cell_division_and_dna_synthesis_activities_n10.scn-NDD1 THEN 000 OR 010 OR 110
110: 0.202782 (1644,12,115,2)

IF SFF'-FKH2 AND SFF'-RLM1 THEN 000 OR 100 OR 110 OR 111
100: 0.206434 (1644,11,127,2)

IF m_anion_transporters_n16-INO4 THEN 000 OR 100 OR 110
100: 0.206434 (1644,11,127,2)

IF MCM1'-MCM1 AND m_nucleotide_transport_n9-MCM1 THEN 000 OR 010 OR 100 OR 101 OR 110
100: 0.206434 (1644,11,127,2)

IF zap1-PUT3 THEN 000 OR 010 OR 100
100: 0.206434 (1644,11,127,2)

IF m_phosphate_transport_n18-INO4 AND m_phosphate_transport_n18-TOS8 THEN 000 OR 001 OR 100 OR 110
100: 0.206434 (1644,11,127,2)

IF CSRE-SUT1 AND CSRE-UME6 THEN 000 OR 010 OR 100 OR 110
100: 0.206434 (1644,11,127,2)

IF SFF'-FKH1 AND SWI5-SKN7 THEN 000 OR 010 OR 100 OR 111
100: 0.206434 (1644,11,127,2)

IF SWI5-SKN7 AND m_deoxyribonucleotide_metabolism_n10-SKN7 THEN 000 OR 001 OR 010 OR 100
100: 0.206434 (1644,11,127,2)

IF MCM1'-MCM1 AND SFF-FKH1 AND SFF-FKH2 THEN 000 OR 100 OR 110 OR 111
100: 0.206434 (1644,11,127,2)

IF RPN4-REB1 THEN 000 OR 001 OR 010 OR 100 OR 110
100: 0.206434 (1644,11,127,2)

IF m_amino-acid_transport_n18-TBS1 THEN 000 OR 010 OR 100 OR 110
100: 0.206434 (1644,11,127,2)

IF SWI5-INO4 AND m_regulation_of_nitrogen_and_sulphur_utilization_n7-INO4 THEN 000 OR 010 OR 100
100: 0.206434 (1644,11,127,2)

IF MCM1-FKH2 AND SFF-FKH1 THEN 000 OR 100 OR 110 OR 111
100: 0.206434 (1644,11,127,2)

IF m_glyoxylate_cycle_n8-UME6 AND m_meiosis_n3-UME6 THEN 000 OR 001 OR 100 OR 110
100: 0.206434 (1644,11,127,2)

IF SWI5-INO4 AND m_phosphate_transport_n18-INO4 THEN 000 OR 010 OR 100
100: 0.206434 (1644,11,127,2)

IF m_chromatin_modification_n21-HSF1 THEN 000 OR 001 OR 010 OR 100

100: 0.206434 (1644,11,127,2)

IF m_translational_control_n10-STB2 THEN 000 OR 100 OR 110
100: 0.206434 (1644,11,127,2)

IF MCM1-FKH2 AND SFF-FKH1 AND SFF-FKH2 THEN 000 OR 100 OR 110 OR 111
100: 0.206434 (1644,11,127,2)

IF m_other_cell_rescue_activities_n10-SMP1 THEN 000 OR 001 OR 100
100: 0.206434 (1644,11,127,2)

IF SCB-SWI4 AND SCB-UME6 THEN 000 OR 010 OR 110
010: 0.206848 (1644,10,140,2)

IF m_metabolism_of_energy_reserves_n27-PHO4 THEN 000 OR 001 OR 010 OR 100
010: 0.206848 (1644,10,140,2)

IF SWI5-INO4 AND m_glycolysis_and_gluconeogenesis_n4-INO4 THEN 000 OR 010 OR 110
010: 0.206848 (1644,10,140,2)

IF REB1-UME6 THEN 000 OR 001 OR 010
010: 0.206848 (1644,10,140,2)

IF m_lipid_transporters_n8-RPI1 THEN 000 OR 001 OR 010 OR 110
010: 0.206848 (1644,10,140,2)

IF m_other_proteolytic_degradation_n8-INO2 THEN 000 OR 010 OR 100 OR 110
010: 0.206848 (1644,10,140,2)

IF m_pentose-phosphate_pathway_n14-GAT1 AND m_phosphate_transport_n18-GAT1 THEN 000 OR 010 OR 100
010: 0.206848 (1644,10,140,2)

IF ALPHA2-RCO1 THEN 000 OR 001 OR 010 OR 100 OR 110
010: 0.206848 (1644,10,140,2)

IF SFF'-HIR3 AND SFF-HIR1 THEN 000 OR 010 OR 100 OR 101
010: 0.206848 (1644,10,140,2)

IF m_anion_transporters_n19-MTH1 THEN 000 OR 010 OR 110
010: 0.206848 (1644,10,140,2)

IF m_other_morphogenetic_activities_n7-RTG1 AND ALPHA1'-RIM101 THEN 000 OR 010 OR 100
010: 0.206848 (1644,10,140,2)

IF STRE-TOS8 THEN 000 OR 001 OR 010
010: 0.206848 (1644,10,140,2)

IF MIG1-SKO1 THEN 000 OR 001 OR 010 OR 100 OR 110
010: 0.206848 (1644,10,140,2)

IF m_g-proteins_n12-INO4 AND SWI5-INO4 AND m_anion_transporters_n15-INO4 THEN 000 OR 001 OR 010 OR 100 OR 110
010: 0.206848 (1644,10,140,2)

IF m_morphogenesis_n5-SWI6 THEN 000 OR 010 OR 100 OR 110
010: 0.206848 (1644,10,140,2)

IF m_g-proteins_n12-SKN7 AND m_RPE57-SKN7 THEN 000 OR 010
010: 0.206848 (1644,10,140,2)

IF SFF'-FKH1 AND ECB-MCM1 THEN 000 OR 010 OR 110 OR 111
010: 0.206848 (1644,10,140,2)

IF SFF'-HIR1 AND SFF'-HIR3 AND SFF-HIR1 THEN 000 OR 010 OR 100 OR 101
010: 0.206848 (1644,10,140,2)

IF SFF'-FKH1 AND ALPHA1'-YER051w THEN 000 OR 010 OR 100 OR 110
010: 0.206848 (1644,10,140,2)

IF m_other_morphogenetic_activities_n8-SFP1 THEN 000 OR 010
010: 0.206848 (1644,10,140,2)

IF m_trna_processing_n6-TBS1 THEN 000 OR 010 OR 100
010: 0.206848 (1644,10,140,2)

IF AFT1-RAP1 AND RAP1-RAP1 THEN 000 OR 010 OR 100
010: 0.206848 (1644,10,140,2)

IF m_lipid_and_fatty-acid_transport_n11-GAT1 AND m_tricarboxylic-acid_pathway_n9-GAT1 THEN 000 OR 010 OR 100 OR 111
010: 0.206848 (1644,10,140,2)

IF MCM1'-SWI4 THEN 000 OR 010 OR 100 OR 101 OR 110 OR 111
100: 0.208366 (1644,52,127,6)

IF SFF'-FKH1 AND SFF'-FKH2 THEN 000 OR 010 OR 100 OR 110 OR 111
100: 0.208366 (1644,52,127,6)

IF m_other_protein-destination_activities_n7-XBP1 THEN 000 OR 001 OR 010 OR 100 OR 110
110: 0.210336 (1644,34,115,4)

IF m_lipid_and_fatty-acid_transport_n11-MTH1 THEN 000 OR 001 OR 010 OR 100 OR 110 OR 111
110: 0.210336 (1644,34,115,4)

IF m_RRSE3-INO4 THEN 000 OR 001 OR 010 OR 100 OR 110
010: 0.210738 (1644,28,140,4)

IF SFF'-HIR3 THEN 000 OR 001 OR 010 OR 011 OR 100 OR 101 OR 110
001: 0.210765 (1644,71,55,4)

IF m_amino-acid_transporters_n11-SWI4 THEN 000 OR 010 OR 110 OR 111
110: 0.214189 (1644,23,115,3)

IF mRRPE-RLM1 THEN 000 OR 010 OR 100 OR 101 OR 110 OR 111
110: 0.214189 (1644,23,115,3)

IF m_pentose-phosphate_pathway_n14-GAT1 THEN 000 OR 010 OR 100 OR 110
110: 0.214189 (1644,23,115,3)

IF Ume6(URS1)-UME6 THEN 000 OR 001 OR 010 OR 100
001: 0.215151 (1644,26,55,2)

IF m_g-proteins_n12-INO4 AND m_anion_transporters_n10-INO4 THEN 000 OR 001 OR 010
010: 0.21585 (1644,19,140,3)

IF m_amino-acid_transporters_n11-UME6 THEN 000 OR 001 OR 010 OR 100
010: 0.21585 (1644,19,140,3)

IF m_amino-acid_transport_n18-SKN7 THEN 000 OR 001 OR 010 OR 100 OR 110
010: 0.21585 (1644,19,140,3)

IF m_g-proteins_n12-INO4 AND m_g-proteins_n12-SKN7 THEN 000 OR 001 OR 010 OR 100 OR 110 OR 111
010: 0.21585 (1644,19,140,3)

IF ALPHA1'-RIM101 THEN 000 OR 001 OR 010 OR 100 OR 101 OR 110
010: 0.216638 (1644,68,140,8)

IF m_pentose-phosphate_pathway_n14-AZF1 THEN 000 OR 010 OR 100 OR 110 OR 111
100: 0.217426 (1644,21,127,3)

IF m_anion_transporters_n22-RAP1 THEN 000 OR 001 OR 010 OR 100 OR 110
100: 0.217426 (1644,21,127,3)

IF m_g-proteins_n12-NRG1 THEN 000 OR 001 OR 010 OR 100 OR 110
100: 0.221083 (1644,53,127,6)

IF SFF-GAL3 THEN 000 OR 001 OR 010 OR 100 OR 110
001: 0.223591 (1644,49,55,3)

IF m_nutritional_response_pathway_n8-INO4 THEN 000 OR 001 OR 010 OR 100 OR 110
001: 0.227662 (1644,27,55,2)

IF SFF'-FKH1 AND SWI5-INO4 THEN 000 OR 010 OR 100 OR 110
110: 0.2293 (1644,13,115,2)

IF STRE-SWI6 THEN 000 OR 001 OR 010 OR 100 OR 110
110: 0.2293 (1644,13,115,2)

IF m_pentose-phosphate_pathway_n21-GAT1 THEN 000 OR 010 OR 110 OR 111
110: 0.2293 (1644,13,115,2)

IF MCM1'-SWI4 AND m_nutritional_response_pathway_n7-SWI4 THEN 000 OR 100 OR 101 OR 110 OR 111
110: 0.2293 (1644,13,115,2)

IF RPN4-GAT1 THEN 000 OR 001 OR 100 OR 110
110: 0.2293 (1644,13,115,2)

IF m_nitrogen_and_sulphur_transport_n9-SFP1 THEN 000 OR 010 OR 110
110: 0.2293 (1644,13,115,2)

IF SFF-ARG80 AND SFF-RTG3 THEN 000 OR 001 OR 010 OR 110
110: 0.2293 (1644,13,115,2)

IF m_regulation_of_nitrogen_and_sulphur_utilization_n13-MTH1 THEN 000 OR 001 OR 010 OR 100 OR 110
110: 0.2293 (1644,13,115,2)

IF SFF'-HIR3 AND SFF-RTG3 THEN 000 OR 001 OR 010 OR 110
110: 0.2293 (1644,13,115,2)

IF m_g-proteins_n12-INO4 AND m_organization_of_chromosome_structure_n17-INO4 THEN 000 OR 001 OR 100 OR 110
110: 0.2293 (1644,13,115,2)

IF SFF'-RTG3 AND SFF-ARG80 THEN 000 OR 001 OR 010 OR 110
110: 0.2293 (1644,13,115,2)

IF m_g-proteins_n12-INO4 AND m_other_mrna-transcription_activities_n20-INO4 THEN 000 OR 001 OR 010 OR 110
110: 0.2293 (1644,13,115,2)

IF m_deoxyribonucleotide_metabolism_n5-MTH1 THEN 000 OR 001 OR 010 OR 100 OR 110 OR 111
110: 0.2293 (1644,13,115,2)

IF MCM1-MCM1 THEN 000 OR 010 OR 100 OR 110 OR 111
010: 0.229441 (1644,29,140,4)

IF SFF-HIR1 THEN 000 OR 001 OR 010 OR 100 OR 101 OR 110
010: 0.2318 (1644,59,140,7)

IF m_g-proteins_n11-INO4 THEN 000 OR 001 OR 010 OR 100 OR 101 OR 110
001: 0.23258 (1644,50,55,3)

IF m_anion_transporters_n15-MTH1 THEN 000 OR 001 OR 100 OR 110
110: 0.233159 (1644,24,115,3)

IF m_other_morphogenetic_activities_n7-RCS1 THEN 000 OR 010 OR 100 OR 101 OR 110
010: 0.23374 (1644,39,140,5)

IF SFF'-MCM1 AND SFF-FKH1 AND SFF-FKH2 THEN 000 OR 010 OR 100 OR 110 OR 111
100: 0.23589 (1644,12,127,2)

IF m_phosphate_transport_n5-GAT1 THEN 000 OR 010 OR 100 OR 110
100: 0.23589 (1644,12,127,2)

IF SFF'-FKH1 AND MCM1'-NDD1 AND SFF-FKH2 THEN 000 OR 010 OR 100 OR 110 OR 111
100: 0.23589 (1644,12,127,2)

IF m_g-proteins_n11-INO4 AND m_anion_transporters_n10-INO4 THEN 000 OR 001 OR 100
100: 0.23589 (1644,12,127,2)

IF SFF'-FKH2 AND MCM1'-NDD1 AND SFF-FKH1 THEN 000 OR 010 OR 100 OR 110 OR 111
100: 0.23589 (1644,12,127,2)

IF m_regulation_of_nucleotide_metabolism_n5-SWI4 THEN 000 OR 010 OR 100 OR 110 OR 111

100: 0.23589 (1644,12,127,2)

IF m_anion_transporters_n22-INO4 THEN 000 OR 001 OR 010 OR 100 OR 110 OR 111
100: 0.23589 (1644,12,127,2)

IF m_c-compound_and_carbohydrate_utilization_n9-MTH1 THEN 000 OR 001 OR 100 OR 110
100: 0.23589 (1644,12,127,2)

IF m_phosphate_transport_n18-INO4 AND m_anion_transporters_n10-INO4 THEN 000 OR 100 OR 110
100: 0.23589 (1644,12,127,2)

IF m_vacuolar_and_lysosomal_organization_n8-SMK1 THEN 000 OR 010 OR 100 OR 110
100: 0.23589 (1644,12,127,2)

IF m_anion_transporters_n32-INO4 THEN 000 OR 100 OR 101 OR 110
100: 0.23589 (1644,12,127,2)

IF MCM1'-MCM1 AND MCM1'-SWI4 THEN 000 OR 100 OR 101 OR 110
100: 0.23589 (1644,12,127,2)

IF m_metabolism_of_energy_reserves_n30-PHD1 THEN 000 OR 001 OR 010 OR 100
100: 0.23589 (1644,12,127,2)

IF SFF'-FKH1 AND SFF'-FKH2 AND MCM1'-MCM1 THEN 000 OR 010 OR 100 OR 110 OR 111
100: 0.23589 (1644,12,127,2)

IF m_other_nucleotide-metabolism_activities_n18-YER184C THEN 000 OR 001 OR 010 OR 100 OR 110
100: 0.23589 (1644,12,127,2)

IF SFF'-FKH1 AND MCM1'-NDD1 AND SFF-FKH1 THEN 000 OR 010 OR 100 OR 110 OR 111
100: 0.23589 (1644,12,127,2)

IF SFF'-FKH2 AND SFF'-MCM1 AND SFF-FKH1 THEN 000 OR 010 OR 100 OR 110 OR 111
100: 0.23589 (1644,12,127,2)

IF SFF'-FKH1 AND SFF'-MCM1 AND SFF-FKH2 THEN 000 OR 010 OR 100 OR 110 OR 111
100: 0.23589 (1644,12,127,2)

IF m_regulation_of_amino-acid_metabolism_n15-TBS1 THEN 000 OR 001 OR 010 OR 100
100: 0.23589 (1644,12,127,2)

IF ALPHA2-INO2 THEN 000 OR 010 OR 100
100: 0.23589 (1644,12,127,2)

IF m_regulation_of_lipid_fatty-acid_and_isoprenoid_biosynthesis_n16.scn-SKN7 THEN 000 OR 001 OR 100
100: 0.238528 (1644,22,127,3)

IF m_amino-acid_transport_n14-UME6 THEN 000 OR 010 OR 100
010: 0.239194 (1644,20,140,3)

IF m_osmosensing_n6-SWI4 THEN 000 OR 010 OR 100 OR 101 OR 110 OR 111
010: 0.239194 (1644,20,140,3)

IF m_amino-acid_transporters_n11-PHD1 THEN 000 OR 001 OR 010 OR 110 OR 111
010: 0.239194 (1644,20,140,3)

IF MCM1'-MCM1 AND m_nucleotide_transport_n9-MCM1 THEN 000 OR 010 OR 100 OR 101 OR 110
010: 0.239476 (1644,11,140,2)

IF m_MERE4-SKN7 AND SWI5-SKN7 THEN 000 OR 010 OR 100
010: 0.239476 (1644,11,140,2)

IF m_other_cell_growth_cell_division_and_dna_synthesis_activities_n10.scn-MGA1 THEN 000 OR 001 OR 010 OR 100
010: 0.239476 (1644,11,140,2)

IF m_nitrogen_and_sulphur_transport_n9-RGM1 THEN 000 OR 010 OR 110
010: 0.239476 (1644,11,140,2)

IF SFF'-FKH1 AND m_lipid_and_fatty-acid_transport_n11-SKN7 THEN 000 OR 010 OR 100 OR 110 OR 111
010: 0.239476 (1644,11,140,2)

IF m_g-proteins_n12-INO4 AND m_regulation_of_lipid_fatty-acid_and_isoprenoid_biosynthesis_n22.scn-INO4 THEN 000 OR 010
010: 0.239476 (1644,11,140,2)

IF m_phosphate_utilization_n9-INO4 THEN 000 OR 001 OR 010 OR 100
010: 0.239476 (1644,11,140,2)

IF SWI5-INO4 AND SWI5-SKN7 AND SWI5-SWI6 THEN 000 OR 010 OR 100 OR 110
010: 0.239476 (1644,11,140,2)

IF m_drug_transporters_n7-MTH1 THEN 000 OR 010
010: 0.239476 (1644,11,140,2)

IF SFF'-HIR1 AND SFF-MIG1 THEN 000 OR 001 OR 010
010: 0.239476 (1644,11,140,2)

IF m_g-proteins_n12-INO4 AND m_glycolysis_and_gluconeogenesis_n4-INO4 THEN 000 OR 010 OR 100 OR 110
010: 0.239476 (1644,11,140,2)

IF SWI5-INO4 AND m_other_nutritional-response_activities_n10-INO4 THEN 000 OR 010 OR 100
010: 0.239476 (1644,11,140,2)

IF m_amino-acid_transport_n18-TBS1 THEN 000 OR 010 OR 100 OR 110
010: 0.239476 (1644,11,140,2)

IF m_amino-acid_transport_n13-MTH1 THEN 000 OR 001 OR 010 OR 100 OR 110
010: 0.239476 (1644,11,140,2)

IF m_deoxyribonucleotide_metabolism_n23-MTH1 AND m_cell_death_n22-MTH1 THEN 000 OR 001 OR 010 OR 110
010: 0.239476 (1644,11,140,2)

IF m_anion_transporters_n4-MTH1 AND m_MERE4-MTH1 THEN 000 OR 010
010: 0.239476 (1644,11,140,2)

IF SFF'-RTG3 AND ALPHA1'-KSS1 THEN 000 OR 010
010: 0.239476 (1644,11,140,2)

IF SFF-HIR1 AND SFF-MIG1 THEN 000 OR 001 OR 010
010: 0.239476 (1644,11,140,2)

IF m_other_pheromone_response_activities_n8-MAC1 THEN 000 OR 010 OR 100 OR 110 OR 111
010: 0.239476 (1644,11,140,2)

IF CSRE-SUT1 AND CSRE-UME6 THEN 000 OR 010 OR 100 OR 110
010: 0.239476 (1644,11,140,2)

IF m_regulation_of_nitrogen_and_sulphur_utilization_n12-INO4 THEN 000 OR 010 OR 100 OR 110
010: 0.239476 (1644,11,140,2)

IF m_other_morphogenetic_activities_n7-MDS3 AND ALPHA1'-STB4 THEN 000 OR 001 OR 010 OR 100
010: 0.239476 (1644,11,140,2)

IF SFF'-FKH1 AND SWI5-SKN7 THEN 000 OR 010 OR 100 OR 111
010: 0.239476 (1644,11,140,2)

IF SFF-HOG1 THEN 000 OR 001 OR 010 OR 100 OR 110
001: 0.240228 (1644,28,55,2)

IF m_LFTE17-GAT1 THEN 000 OR 001 OR 010 OR 100 OR 101 OR 110 OR 111
001: 0.240228 (1644,28,55,2)

IF m_glyoxylate_cycle_n8-INO4 THEN 000 OR 001 OR 010 OR 100 OR 110 OR 111
110: 0.241045 (1644,36,115,4)

IF mRRPE-ABF1 THEN 000 OR 001 OR 010 OR 100 OR 110
100: 0.247266 (1644,55,127,6)

IF m_MERE4-MTH1 THEN 000 OR 001 OR 010 OR 100 OR 110
010: 0.248545 (1644,30,140,4)

IF SCB-SWI4 THEN 000 OR 010 OR 100 OR 110 OR 111
010: 0.250206 (1644,40,140,5)

IF SWI5-SKN7 THEN 000 OR 001 OR 010 OR 100 OR 110 OR 111
110: 0.250705 (1644,61,115,6)

IF SFF'-HIR3 THEN 000 OR 001 OR 010 OR 011 OR 100 OR 101 OR 110
010: 0.252965 (1644,71,140,8)

IF m_other_morphogenetic_activities_n7-RTG1 THEN 000 OR 010 OR 011 OR 100 OR 110
110: 0.255033 (1644,49,115,5)

IF SFF-ARG80 THEN 000 OR 001 OR 010 OR 011 OR 100 OR 110
110: 0.255033 (1644,49,115,5)

IF PDR-UME6 THEN 000 OR 100 OR 110
110: 0.256038 (1644,14,115,2)

IF m_organization_of_plasma_membrane_n17-INO4 THEN 000 OR 100 OR 101 OR 110
110: 0.256038 (1644,14,115,2)

IF SWI5-SKN7 AND CSRE-SKN7 THEN 000 OR 001 OR 010 OR 100 OR 110
110: 0.256038 (1644,14,115,2)

IF SWI5-INO4 AND m_other_mrna-transcription_activities_n20-INO4 THEN 000 OR 001 OR 010 OR 100 OR 110
110: 0.256038 (1644,14,115,2)

IF m_deoxyribonucleotide_metabolism_n12-TOS8 THEN 000 OR 001 OR 010 OR 110
110: 0.256038 (1644,14,115,2)

IF m_amino-acid_degradation_n24-SKN7 THEN 000 OR 010 OR 110
110: 0.256038 (1644,14,115,2)

IF m_g-proteins_n12-SKN7 AND m_lipid_and_fatty-acid_transport_n7-SKN7 THEN 000 OR 010 OR 110 OR 111
110: 0.256038 (1644,14,115,2)

IF m_g-proteins_n12-INO4 AND m_other_energy_generation_activities_n22-INO4 THEN 000 OR 001 OR 010 OR 110
110: 0.256038 (1644,14,115,2)

IF m_other_nutritional-response_activities_n10-ZMS1 THEN 000 OR 100 OR 110
110: 0.256038 (1644,14,115,2)

IF SWI5-SWI6 AND m_deoxyribonucleotide_metabolism_n10-SWI6 THEN 000 OR 001 OR 110 OR 111
110: 0.256038 (1644,14,115,2)

IF MCM1'-SWI4 AND SWI5-SWI6 THEN 000 OR 010 OR 100 OR 101 OR 110 OR 111
110: 0.256038 (1644,14,115,2)

IF mRRPE-A1(MATA1) THEN 000 OR 010 OR 100 OR 110
110: 0.256038 (1644,14,115,2)

IF m_amino-acid_transporters_n11-SKN7 AND m_amino-acid_transporters_n11-SWI4 THEN 000 OR 010 OR 110 OR 111
110: 0.256038 (1644,14,115,2)

IF HAP234-HAP4 THEN 000 OR 001 OR 010 OR 100 OR 110
110: 0.256038 (1644,14,115,2)

IF m_regulation_of_amino-acid_metabolism_n15-CBF1 THEN 000 OR 100 OR 110
110: 0.256038 (1644,14,115,2)

IF SWI5-SKN7 THEN 000 OR 001 OR 010 OR 100 OR 110 OR 111
010: 0.258474 (1644,61,140,7)

IF m_g-proteins_n12-NRG1 THEN 000 OR 001 OR 010 OR 100 OR 110
001: 0.259932 (1644,53,55,3)

IF SFF'-FKH2 AND SFF'-MCM1 THEN 000 OR 010 OR 011 OR 100 OR 110 OR 111
100: 0.259966 (1644,23,127,3)

IF m_allantoin_and_allantoate_transporters_n7-STE12 THEN 000 OR 001 OR 010 OR 100 OR 110

100: 0.259966 (1644,23,127,3)

IF m_pentose-phosphate_pathway_n14-GAT1 THEN 000 OR 010 OR 100 OR 110
100: 0.259966 (1644,23,127,3)

IF SFF'-RTG3 THEN 000 OR 001 OR 010 OR 100 OR 101 OR 110
110: 0.262868 (1644,62,115,6)

IF MCM1'-SWI4 AND SCB-SWI4 THEN 000 OR 010 OR 100 OR 110 OR 111
010: 0.262943 (1644,21,140,3)

IF SWI5-INO4 AND SWI5-SKN7 THEN 000 OR 001 OR 010 OR 100 OR 110
010: 0.262943 (1644,21,140,3)

IF m_anion_transporters_n17-UME6 THEN 000 OR 001 OR 010 OR 100
010: 0.262943 (1644,21,140,3)

IF m_deoxyribonucleotide_metabolism_n23-MTH1 THEN 000 OR 001 OR 010 OR 100 OR 110
010: 0.262943 (1644,21,140,3)

IF m_lipid_and_fatty-acid_transport_n11-SWI5 THEN 000 OR 001 OR 010 OR 110 OR 111
010: 0.262943 (1644,21,140,3)

IF mPROTEOL18(m_proteolysis_n18)-UME6 THEN 000 OR 010 OR 100 OR 110
010: 0.262943 (1644,21,140,3)

IF m_regulation_of_lipid_fatty-acid_and_isoprenoid_biosynthesis_n8.scn-GAT1 THEN 000 OR 001 OR 010 OR 100 OR 110
010: 0.262943 (1644,21,140,3)

IF m_pentose-phosphate_pathway_n14-MTH1 THEN 000 OR 010 OR 100 OR 110
100: 0.265224 (1644,34,127,4)

IF m_deoxyribonucleotide_metabolism_n5-GAT1 THEN 000 OR 001 OR 010 OR 100 OR 110 OR 111
100: 0.265562 (1644,13,127,2)

IF MIG1-SUT1 THEN 000 OR 010 OR 100
100: 0.265562 (1644,13,127,2)

IF m_other_energy_generation_activities_n9-UME6 THEN 000 OR 100 OR 101
100: 0.265562 (1644,13,127,2)

IF SFF'-MCM1 AND MCM1'-NDD1 AND m_other_morphogenetic_activities_n7-MCM1 THEN 000 OR 010 OR 100 OR 110
100: 0.265562 (1644,13,127,2)

IF SFF'-HIR1 AND SFF'-HIR3 THEN 000 OR 010 OR 100 OR 101
100: 0.265562 (1644,13,127,2)

IF SFF'-FKH1 AND SFF'-FKH2 AND MCM1-FKH2 THEN 000 OR 010 OR 100 OR 110 OR 111
100: 0.265562 (1644,13,127,2)

IF m_other_nucleotide-metabolism_activities_n17-SIG1 THEN 000 OR 010 OR 100 OR 110
100: 0.265562 (1644,13,127,2)

IF CCA-MET4 THEN 000 OR 010 OR 100 OR 110
100: 0.265562 (1644,13,127,2)

IF MCM1'-NDD1 AND m_other_morphogenetic_activities_n7-MCM1 THEN 000 OR 010 OR 100 OR 110
100: 0.265562 (1644,13,127,2)

IF m_other_cell_rescue_activities_n10-PHO4 THEN 000 OR 010 OR 100 OR 110
100: 0.265562 (1644,13,127,2)

IF m_g-proteins_n12-INO4 AND m_organization_of_chromosome_structure_n17-INO4 THEN 000 OR 001 OR 100 OR 110
100: 0.265562 (1644,13,127,2)

IF ALPHA1'-UPC2 AND ALPHA1'-YBR239c THEN 000 OR 001 OR 100
100: 0.265562 (1644,13,127,2)

IF ABF1-WAR1 THEN 000 OR 010 OR 011 OR 100 OR 110 OR 111
100: 0.265562 (1644,13,127,2)

IF SFF'-FKH1 AND MCM1'-MCM1 AND SFF-FKH1 THEN 000 OR 010 OR 100 OR 110 OR 111
100: 0.265562 (1644,13,127,2)

IF SFF'-MCM1 AND MCM1'-MCM1 AND SFF-FKH1 THEN 000 OR 010 OR 100 OR 110 OR 111
100: 0.265562 (1644,13,127,2)

IF m_MERE17-MTH1 THEN 000 OR 100
100: 0.265562 (1644,13,127,2)

IF SFF'-MCM1 AND MCM1'-NDD1 AND SFF-FKH2 THEN 000 OR 100 OR 110 OR 111
100: 0.265562 (1644,13,127,2)

IF m_RPE68-RAP1 THEN 000 OR 001 OR 010 OR 100
100: 0.265562 (1644,13,127,2)

IF SFF'-FKH1 AND MCM1-FKH2 THEN 000 OR 010 OR 100 OR 110 OR 111
100: 0.265562 (1644,13,127,2)

IF m_translational_control_n10-MET28 THEN 000 OR 001 OR 010 OR 100
100: 0.265562 (1644,13,127,2)

IF ALPHA1'-RIM101 AND ALPHA1'-YBR239c THEN 000 OR 010 OR 100
100: 0.265562 (1644,13,127,2)

IF MCM1-SWI4 AND MCM1'-SWI4 THEN 000 OR 010 OR 100 OR 110 OR 111
100: 0.265562 (1644,13,127,2)

IF SFF'-FKH2 AND MCM1'-MCM1 AND MCM1'-NDD1 AND SFF-FKH2 THEN 000 OR 100 OR 110 OR 111
100: 0.265562 (1644,13,127,2)

IF mRRPE-SPT2 THEN 000 OR 010 OR 100
100: 0.265562 (1644,13,127,2)

IF STRE-SWI6 THEN 000 OR 001 OR 010 OR 100 OR 110
100: 0.265562 (1644,13,127,2)

IF STRE-RAP1 THEN 000 OR 001 OR 010 OR 100
100: 0.265562 (1644,13,127,2)

IF SFF'-HIR1 AND SFF'-RLM1 THEN 000 OR 100 OR 110
100: 0.265562 (1644,13,127,2)

IF MCM1'-MCM1 AND MCM1'-NDD1 AND m_other_morphogenetic_activities_n7-MCM1 THEN 000 OR 010 OR 100 OR 110
100: 0.265562 (1644,13,127,2)

IF m_g-proteins_n12-INO4 AND m_regulation_of_amino-acid_metabolism_n15-INO4 THEN 000 OR 010 OR 100
100: 0.265562 (1644,13,127,2)

IF m_g-proteins_n12-INO4 AND m_RRSE3-INO4 THEN 000 OR 010 OR 100 OR 110
100: 0.265562 (1644,13,127,2)

IF MCM1'-NDD1 AND SFF-FKH1 THEN 000 OR 010 OR 100 OR 110 OR 111
100: 0.265562 (1644,13,127,2)

IF CSRE-UME6 AND m_meiosis_n3-UME6 THEN 000 OR 001 OR 010 OR 100
100: 0.265562 (1644,13,127,2)

IF m_other_morphogenetic_activities_n7-MDS3 AND m_other_morphogenetic_activities_n7-RTG1 THEN 000 OR 100
100: 0.265562 (1644,13,127,2)

IF ECB-MCM1 THEN 000 OR 010 OR 100 OR 110 OR 111
010: 0.267983 (1644,31,140,4)

IF m_g-proteins_n11-INO4 THEN 000 OR 001 OR 010 OR 100 OR 101 OR 110
110: 0.268758 (1644,50,115,5)

IF m_cell_death_n22-SWI4 THEN 000 OR 010 OR 100 OR 101 OR 110 OR 111
110: 0.271913 (1644,26,115,3)

IF m_cell_death_n22-MAL13 THEN 000 OR 010 OR 100 OR 101
010: 0.272335 (1644,12,140,2)

IF m_phosphate_transport_n18-GAT1 AND m_glyoxylate_cycle_n11-GAT1 THEN 000 OR 010 OR 111
010: 0.272335 (1644,12,140,2)

IF m_regulation_of_nitrogen_and_sulphur_utilization_n13-UME6 THEN 000 OR 001 OR 010 OR 100
010: 0.272335 (1644,12,140,2)

IF RAP1-PDR1 AND RAP1-RAP1 AND RAP1-SMP1 THEN 000 OR 001 OR 010
010: 0.272335 (1644,12,140,2)

IF MCM1'-SWI4 AND m_amino-acid_transporters_n11-SWI4 THEN 000 OR 010 OR 111
010: 0.272335 (1644,12,140,2)

IF m_metal_ion_transporters_n17-FKH2 THEN 000 OR 010 OR 110
010: 0.272335 (1644,12,140,2)

IF m_other_proteolytic_degradation_n2-MTH1 AND m_cell_death_n22-MTH1 THEN 000 OR 010
010: 0.272335 (1644,12,140,2)

IF m_phosphate_transport_n5-INO4 THEN 000 OR 001 OR 010 OR 100 OR 110
010: 0.272335 (1644,12,140,2)

IF m_ion_transporters_n3-MTH1 THEN 000 OR 001 OR 010
010: 0.272335 (1644,12,140,2)

IF m_regulation_of_amino-acid_metabolism_n15-TBS1 THEN 000 OR 001 OR 010 OR 100
010: 0.272335 (1644,12,140,2)

IF PHO-NDD1 THEN 000 OR 010 OR 100 OR 110
010: 0.272335 (1644,12,140,2)

IF RAP1-PDR1 AND RAP1-SMP1 THEN 000 OR 001 OR 010
010: 0.272335 (1644,12,140,2)

IF MCM1'-SWI4 AND mPROTEOL18(m_proteolysis_n18)-SWI6 THEN 000 OR 010 OR 100 OR 110 OR 111
010: 0.272335 (1644,12,140,2)

IF MCM1-MCM1 AND m_other_morphogenetic_activities_n7-MCM1 THEN 000 OR 010 OR 100 OR 110
010: 0.272335 (1644,12,140,2)

IF m_g-proteins_n11-INO4 AND m_other_mrna-transcription_activities_n20-INO4 THEN 000 OR 001 OR 010 OR 100 OR 110
010: 0.272335 (1644,12,140,2)

IF m_lipid_and_fatty-acid_transport_n11-GAT1 AND m_lipid_and_fatty-acid_transport_n11-SKN7 THEN 000 OR 010 OR 100 OR 110 OR 111
010: 0.272335 (1644,12,140,2)

IF m_amino-acid_degradation_n24-SUT1 THEN 000 OR 010 OR 100
010: 0.272335 (1644,12,140,2)

IF SFF'-RLM1 AND m_g-proteins_n12-NRG1 THEN 000 OR 010 OR 100
010: 0.272335 (1644,12,140,2)

IF m_lipid_and_fatty-acid_transport_n11-SKN7 AND m_other_mrna-transcription_activities_n20-SKN7 THEN 000 OR 010 OR 110
010: 0.272335 (1644,12,140,2)

IF m_g-proteins_n12-SKN7 AND SWI5-SKN7 AND m_RRSE3-SKN7 THEN 000 OR 010 OR 110
010: 0.272335 (1644,12,140,2)

IF MCM1'-MCM1 THEN 000 OR 001 OR 010 OR 100 OR 101 OR 110 OR 111
100: 0.274306 (1644,57,127,6)

IF mRRPE-ABF1 THEN 000 OR 001 OR 010 OR 100 OR 110
001: 0.27842 (1644,55,55,3)

IF MCM1-MCM1 AND ECB-MCM1 THEN 000 OR 010 OR 100 OR 110 OR 111
100: 0.281649 (1644,24,127,3)

IF m_deoxyribonucleotide_metabolism_n10-SKN7 THEN 000 OR 001 OR 010 OR 100 OR 111

100: 0.281649 (1644,24,127,3)

IF mPROTEOL18(m_proteolysis_n18)-SWI6 THEN 000 OR 010 OR 100 OR 110 OR 111
100: 0.281649 (1644,24,127,3)

IF PHO4-CBF1 THEN 000 OR 001 OR 010 OR 100
100: 0.281649 (1644,24,127,3)

IF m_LFTE17-UME6 THEN 000 OR 001 OR 010 OR 100 OR 101 OR 110
100: 0.281649 (1644,24,127,3)

IF m_pentose-phosphate_pathway_n7-MTH1 THEN 000 OR 010 OR 100 OR 110
110: 0.282836 (1644,15,115,2)

IF m_deoxyribonucleotide_metabolism_n23-SWI4 THEN 000 OR 100 OR 110 OR 111
110: 0.282836 (1644,15,115,2)

IF m_tricarboxylic-acid_pathway_n6-CBF1 THEN 000 OR 010 OR 100 OR 110
110: 0.282836 (1644,15,115,2)

IF SWI5-INO4 AND m_anion_transporters_n15-INO4 THEN 000 OR 001 OR 010 OR 100 OR 110
110: 0.282836 (1644,15,115,2)

IF SFF-GZF3 AND SFF-RPI1 THEN 000 OR 010 OR 110
110: 0.282836 (1644,15,115,2)

IF m_ionic_homeostasis_n6-SKN7 THEN 000 OR 001 OR 010 OR 110
110: 0.282836 (1644,15,115,2)

IF m_amino-acid_degradation_n7-SKN7 THEN 000 OR 110 OR 111
110: 0.282836 (1644,15,115,2)

IF m_drug_transporters_n10-SKN7 THEN 000 OR 110
110: 0.282836 (1644,15,115,2)

IF m_other_energy_generation_activities_n12-MTH1 THEN 000 OR 110
110: 0.282836 (1644,15,115,2)

IF m_amino-acid_transporters_n11-SKN7 THEN 000 OR 001 OR 010 OR 110 OR 111
110: 0.291565 (1644,27,115,3)

IF SFF'-FKH1 AND SFF'-FKH2 AND MCM1'-NDD1 THEN 000 OR 010 OR 100 OR 110 OR 111
100: 0.295243 (1644,14,127,2)

IF SFF'-FKH2 AND MCM1'-MCM1 AND SFF-FKH2 THEN 000 OR 100 OR 110 OR 111
100: 0.295243 (1644,14,127,2)

IF m_other_morphogenetic_activities_n8-ADR1 THEN 000 OR 001 OR 010 OR 100
100: 0.295243 (1644,14,127,2)

IF PDR-UME6 THEN 000 OR 100 OR 110
100: 0.295243 (1644,14,127,2)

IF MCM1'-SWI4 AND SWI5-SWI6 THEN 000 OR 010 OR 100 OR 101 OR 110 OR 111
100: 0.295243 (1644,14,127,2)

IF MCM1'-MCM1 AND MCM1'-NDD1 AND SFF-FKH2 THEN 000 OR 100 OR 110 OR 111
100: 0.295243 (1644,14,127,2)

IF m_g-proteins_n12-SKN7 AND m_phosphate_transport_n13-SKN7 THEN 000 OR 001 OR 010 OR 100 OR 110
100: 0.295243 (1644,14,127,2)

IF m_metal_ion_transporters_n6-REB1 THEN 000 OR 010 OR 100 OR 110
100: 0.295243 (1644,14,127,2)

IF MCM1'-NDD1 AND SWI5-SWI6 THEN 000 OR 001 OR 010 OR 100 OR 110 OR 111
100: 0.295243 (1644,14,127,2)

IF SFF'-FKH1 AND MCM1'-NDD1 THEN 000 OR 010 OR 100 OR 110 OR 111
100: 0.295243 (1644,14,127,2)

IF MCM1'-NDD1 AND MCM1'-SWI4 THEN 000 OR 100 OR 110
100: 0.295243 (1644,14,127,2)

IF m_trna_processing_n6-MSN2 THEN 000 OR 010 OR 100
100: 0.295243 (1644,14,127,2)

IF SFF-FKH1 AND m_regulation_of_lipid_fatty-acid_and_isoprenoid_biosynthesis_n8.scn-FKH1 THEN 000 OR 100 OR 110
100: 0.295243 (1644,14,127,2)

IF MCM1'-MCM1 AND SFF-FKH1 THEN 000 OR 010 OR 100 OR 110 OR 111
100: 0.295243 (1644,14,127,2)

IF m_glyoxylate_cycle_n8-MTH1 THEN 000 OR 001 OR 010 OR 110
001: 0.303319 (1644,33,55,2)

IF m_RPE68-RAP1 THEN 000 OR 001 OR 010 OR 100
010: 0.305149 (1644,13,140,2)

IF SFF'-HIR1 AND SFF'-HIR3 THEN 000 OR 010 OR 100 OR 101
010: 0.305149 (1644,13,140,2)

IF m_other_proteolytic_degradation_n2-SFP1 THEN 000 OR 001 OR 010 OR 100
010: 0.305149 (1644,13,140,2)

IF m_organization_of_cell_wall_n6-MET32 THEN 000 OR 001 OR 010 OR 100
010: 0.305149 (1644,13,140,2)

IF m_g-proteins_n12-SKN7 AND m_MERE4-SKN7 THEN 000 OR 001 OR 010 OR 100 OR 110
010: 0.305149 (1644,13,140,2)

IF MIG1-SUT1 THEN 000 OR 010 OR 100
010: 0.305149 (1644,13,140,2)

IF m_nitrogen_and_sulphur_transport_n9-FHL1 THEN 000 OR 010 OR 110
010: 0.305149 (1644,13,140,2)

IF m_g-proteins_n12-INO4 AND m_RRSE3-INO4 THEN 000 OR 010 OR 100 OR 110
010: 0.305149 (1644,13,140,2)

IF CCA-MET4 THEN 000 OR 010 OR 100 OR 110
010: 0.305149 (1644,13,140,2)

IF m_g-proteins_n11-INO4 AND m_regulation_of_lipid_fatty-acid_and_isoprenoid_biosynthesis_n12.scn-INO4 THEN 000 OR 010 OR 110
010: 0.305149 (1644,13,140,2)

IF m_other_cell_rescue_activities_n10-PHO4 THEN 000 OR 010 OR 100 OR 110
010: 0.305149 (1644,13,140,2)

IF m_other_transport_facilitators_n10-SKN7 THEN 000 OR 010 OR 111
010: 0.305149 (1644,13,140,2)

IF m_g-proteins_n12-INO4 AND m_other_mrna-transcription_activities_n20-INO4 THEN 000 OR 001 OR 010 OR 110
010: 0.305149 (1644,13,140,2)

IF m_other_mrna-transcription_activities_n11-RGM1 THEN 000 OR 010
010: 0.305149 (1644,13,140,2)

IF m_translational_control_n10-MET28 THEN 000 OR 001 OR 010 OR 100
010: 0.305149 (1644,13,140,2)

IF SFF'-FKH1 AND ABF1-ABF1 THEN 000 OR 010 OR 100 OR 110
010: 0.305149 (1644,13,140,2)

IF SFF'-HIR3 THEN 000 OR 001 OR 010 OR 011 OR 100 OR 101 OR 110
100: 0.306 (1644,71,127,7)

IF SFF'-HIR3 AND SFF'-RTG3 THEN 000 OR 001 OR 010 OR 110
110: 0.309557 (1644,16,115,2)

IF m_pentose-phosphate_pathway_n5-MCM1 THEN 000 OR 010 OR 100 OR 101 OR 110 OR 111
110: 0.309557 (1644,16,115,2)

IF m_amino-acid_degradation_n8-NDD1 THEN 000 OR 110
110: 0.309557 (1644,16,115,2)

IF SFF-FKH1 AND m_pentose-phosphate_pathway_n14-FKH1 THEN 000 OR 100 OR 110 OR 111
110: 0.309557 (1644,16,115,2)

IF m_lipid_and_fatty-acid_transport_n11-GAT1 AND m_LFTE17-GAT1 THEN 000 OR 001 OR 010 OR 100 OR 110 OR 111
110: 0.309557 (1644,16,115,2)

IF m_lipid_transporters_n8-MET4 THEN 000 OR 010 OR 100 OR 110
110: 0.309557 (1644,16,115,2)

IF m_glyoxylate_cycle_n7-ASH1 THEN 000 OR 100 OR 110 OR 111
110: 0.309557 (1644,16,115,2)

IF m_nitrogen_and_sulphur_metabolism_n17-XBP1 THEN 000 OR 001 OR 100 OR 110
110: 0.309557 (1644,16,115,2)

IF mPROTEOL18(m_proteolysis_n18)-MBP1 THEN 000 OR 001 OR 010 OR 100 OR 110 OR 111
010: 0.311181 (1644,23,140,3)

IF m_glyoxylate_cycle_n11-RGM1 THEN 000 OR 001 OR 010 OR 111
010: 0.311181 (1644,23,140,3)

IF m_nutritional_response_pathway_n7-SWI6 THEN 000 OR 010 OR 101 OR 110 OR 111
010: 0.311181 (1644,23,140,3)

IF m_amino-acid_transporters_n11-SWI4 THEN 000 OR 010 OR 110 OR 111
010: 0.311181 (1644,23,140,3)

IF m_pentose-phosphate_pathway_n14-GAT1 THEN 000 OR 010 OR 100 OR 110
010: 0.311181 (1644,23,140,3)

IF SFF-RPI1 THEN 000 OR 001 OR 010 OR 011 OR 100 OR 101 OR 110 OR 111
110: 0.312917 (1644,66,115,6)

IF SFF'-HIR1 THEN 000 OR 001 OR 010 OR 100 OR 101 OR 110
010: 0.314216 (1644,65,140,7)

IF m_phosphate_transport_n18-INO4 THEN 000 OR 001 OR 010 OR 100 OR 110 OR 111
100: 0.319037 (1644,37,127,4)

IF m_other_morphogenetic_activities_n7-GCR2 THEN 000 OR 001 OR 010 OR 100 OR 110
110: 0.321429 (1644,41,115,4)

IF m_amino-acid_degradation_n8-YAP6 THEN 000 OR 001 OR 010 OR 100
100: 0.324756 (1644,15,127,2)

IF SFF'-FKH2 AND MCM1'-MCM1 AND MCM1'-NDD1 THEN 000 OR 010 OR 100 OR 110 OR 111
100: 0.324756 (1644,15,127,2)

IF SFF'-FKH1 AND SFF'-FKH2 AND SFF'-MCM1 THEN 000 OR 010 OR 100 OR 110 OR 111
100: 0.324756 (1644,15,127,2)

IF m_pentose-phosphate_pathway_n7-MTH1 THEN 000 OR 010 OR 100 OR 110
100: 0.324756 (1644,15,127,2)

IF m_allantoin_and_allantoate_transporters_n13-MAL13 THEN 000 OR 100 OR 101 OR 110
100: 0.324756 (1644,15,127,2)

IF ABF1-PPR1 THEN 000 OR 010 OR 100 OR 110
100: 0.324756 (1644,15,127,2)

IF Yap1-YAP1 THEN 000 OR 001 OR 010 OR 100 OR 110
100: 0.324756 (1644,15,127,2)

IF SWI5-SKN7 AND m_ion_transporters_n7-SKN7 THEN 000 OR 100 OR 110

100: 0.324756 (1644,15,127,2)

IF MIG1-ROX1 THEN 000 OR 001 OR 011 OR 100 OR 110 OR 111
100: 0.324756 (1644,15,127,2)

IF m_organization_of_intracellular_transport_vesicles_n5-MTH1 THEN 000 OR 001 OR 100
100: 0.324756 (1644,15,127,2)

IF m_nucleotide_transport_n9-SWI6 THEN 000 OR 010 OR 100 OR 101 OR 110
100: 0.324756 (1644,15,127,2)

IF SFF'-FKH2 AND SFF'-MCM1 AND MCM1'-NDD1 THEN 000 OR 010 OR 100 OR 110 OR 111
100: 0.324756 (1644,15,127,2)

IF MCM1'-MCM1 AND SFF-FKH2 THEN 000 OR 100 OR 110 OR 111
100: 0.324756 (1644,15,127,2)

IF ATRepeat-HAP1 THEN 000 OR 001 OR 010 OR 100
100: 0.324756 (1644,15,127,2)

IF m_g-proteins_n12-SKN7 AND m_deoxyribonucleotide_metabolism_n10-SKN7 THEN 000 OR 001 OR 010 OR 100
100: 0.324756 (1644,15,127,2)

IF m_osmosensing_n6-INO4 THEN 000 OR 010 OR 100 OR 101 OR 110
100: 0.325418 (1644,26,127,3)

IF m_other_protein-destination_activities_n7-MET4 THEN 000 OR 010 OR 100 OR 110
100: 0.325418 (1644,26,127,3)

IF m_other_morphogenetic_activities_n7-RTG1 THEN 000 OR 010 OR 011 OR 100 OR 110
100: 0.326565 (1644,49,127,5)

IF m_phosphate_transport_n18-GAT1 THEN 000 OR 001 OR 010 OR 100 OR 101 OR 110 OR 111
010: 0.327652 (1644,34,140,4)

IF m_other_morphogenetic_activities_n7-ARR1 THEN 000 OR 010 OR 100 OR 110
010: 0.33545 (1644,24,140,3)

IF mPROTEOL18(m_proteolysis_n18)-SWI6 THEN 000 OR 010 OR 100 OR 110 OR 111
010: 0.33545 (1644,24,140,3)

IF m_regulation_of_lipid_fatty-acid_and_isoprenoid_biosynthesis_n12.scn-INO4 THEN 000 OR 001 OR 010 OR 110
010: 0.33545 (1644,24,140,3)

IF m_other_proteolytic_degradation_n2-MTH1 THEN 000 OR 001 OR 010 OR 100 OR 110
010: 0.33545 (1644,24,140,3)

IF ABF1-SIG1 THEN 000 OR 010 OR 011 OR 100 OR 110
110: 0.33608 (1644,17,115,2)

IF m_phosphate_transport_n18-ECM22 THEN 000 OR 001 OR 010 OR 100 OR 110
110: 0.33608 (1644,17,115,2)

IF m_deoxyribonucleotide_metabolism_n10-INO4 THEN 000 OR 001 OR 010 OR 100 OR 101 OR 111
100: 0.337213 (1644,38,127,4)

IF MCM1'-SWI4 AND SWI5-SWI6 THEN 000 OR 010 OR 100 OR 101 OR 110 OR 111
010: 0.33769 (1644,14,140,2)

IF SFF'-FKH1 AND MCM1-MCM1 THEN 000 OR 010 OR 100 OR 110 OR 111
010: 0.33769 (1644,14,140,2)

IF SWI5-SKN7 AND m_RRSE3-SKN7 THEN 000 OR 010 OR 110
010: 0.33769 (1644,14,140,2)

IF m_amino-acid_transport_n14-MIG2 THEN 000 OR 010 OR 100
010: 0.33769 (1644,14,140,2)

IF m_other_energy_generation_activities_n4-SIP4 THEN 000 OR 001 OR 010
010: 0.33769 (1644,14,140,2)

IF SWI5-INO4 AND m_RRSE3-INO4 THEN 000 OR 010 OR 100 OR 110
010: 0.33769 (1644,14,140,2)

IF SFF'-FKH1 AND MCM1'-NDD1 THEN 000 OR 010 OR 100 OR 110 OR 111
010: 0.33769 (1644,14,140,2)

IF SWI5-SKN7 AND m_amino-acid_transport_n18-SKN7 THEN 000 OR 001 OR 010 OR 100 OR 110
010: 0.33769 (1644,14,140,2)

IF SWI5-INO4 AND m_other_mrna-transcription_activities_n20-INO4 THEN 000 OR 001 OR 010 OR 100 OR 110
010: 0.33769 (1644,14,140,2)

IF m_other_nutritional-response_activities_n10-INO2 THEN 000 OR 001 OR 010
010: 0.33769 (1644,14,140,2)

IF m_g-proteins_n12-INO4 AND m_mitochondrial_biogenesis_n5-INO4 THEN 000 OR 010 OR 100 OR 110
010: 0.33769 (1644,14,140,2)

IF m_cytok9-SWI6 THEN 000 OR 010 OR 100 OR 110 OR 111
010: 0.33769 (1644,14,140,2)

IF m_regulation_of_lipid_fatty-acid_and_isoprenoid_biosynthesis_n12.scn-INO2 THEN 000 OR 001 OR 010 OR 111
010: 0.33769 (1644,14,140,2)

IF m_metal_ion_transporters_n25-SFP1 THEN 000 OR 010 OR 100 OR 110
010: 0.33769 (1644,14,140,2)

IF SWI5-SKN7 AND m_amino-acid_transporters_n11-SKN7 THEN 000 OR 001 OR 010 OR 110
010: 0.33769 (1644,14,140,2)

IF m_lipid_and_fatty-acid_transport_n11-GAT1 THEN 000 OR 001 OR 010 OR 100 OR 110 OR 111
110: 0.337847 (1644,42,115,4)

IF m_other_morphogenetic_activities_n7-MDS3 THEN 000 OR 001 OR 010 OR 100 OR 101 OR 110
110: 0.337847 (1644,42,115,4)

IF SFF'-SIP3 THEN 000 OR 001 OR 010 OR 100 OR 110
100: 0.342429 (1644,50,127,5)

IF m_g-proteins_n11-INO4 THEN 000 OR 001 OR 010 OR 100 OR 101 OR 110
100: 0.342429 (1644,50,127,5)

IF SCB-UME6 THEN 000 OR 001 OR 010 OR 100 OR 110
010: 0.353639 (1644,46,140,5)

IF m_lipid_and_fatty-acid_transport_n11-SKN7 AND SWI5-SKN7 THEN 000 OR 010 OR 100 OR 110 OR 111
100: 0.353951 (1644,16,127,2)

IF SWI5-INO4 AND SWI5-SWI6 THEN 000 OR 010 OR 100 OR 110
100: 0.353951 (1644,16,127,2)

IF SFF'-FKH1 AND SFF-FKH1 AND m_pentose-phosphate_pathway_n14-FKH1 THEN 000 OR 100 OR 110 OR 111
100: 0.353951 (1644,16,127,2)

IF m_ion_transporters_n11-SWI4 THEN 000 OR 100 OR 110 OR 111
100: 0.353951 (1644,16,127,2)

IF m_ion_transporters_n4-GAT1 THEN 000 OR 001 OR 010 OR 100 OR 111
100: 0.353951 (1644,16,127,2)

IF m_nucleotide_transport_n9-MCM1 THEN 000 OR 010 OR 100 OR 101 OR 110
100: 0.353951 (1644,16,127,2)

IF m_nitrogen_and_sulphur_metabolism_n17-XBP1 THEN 000 OR 001 OR 100 OR 110
100: 0.353951 (1644,16,127,2)

IF SFF-FKH1 AND m_pentose-phosphate_pathway_n14-FKH1 THEN 000 OR 100 OR 110 OR 111
100: 0.353951 (1644,16,127,2)

IF m_homeostasis_of_other_ions_n30-INO4 THEN 000 OR 001 OR 010 OR 100 OR 110
100: 0.353951 (1644,16,127,2)

IF ALPHA2-MSN2 THEN 000 OR 010 OR 100
100: 0.353951 (1644,16,127,2)

IF m_lipid_and_fatty-acid_transport_n11-GAT1 AND m_LFTE17-GAT1 THEN 000 OR 001 OR 010 OR 100 OR 110 OR 111
100: 0.353951 (1644,16,127,2)

IF SFF'-FKH2 AND SFF'-MCM1 AND MCM1'-MCM1 THEN 000 OR 010 OR 100 OR 110 OR 111
100: 0.353951 (1644,16,127,2)

IF m_cell_death_n16-MBP1 THEN 000 OR 100 OR 110 OR 111
100: 0.353951 (1644,16,127,2)

IF m_metal_ion_transporters_n10-SKN7 THEN 000 OR 100 OR 110
100: 0.353951 (1644,16,127,2)

IF SFF'-FKH2 AND MCM1'-MCM1 THEN 000 OR 010 OR 100 OR 110 OR 111
100: 0.353951 (1644,16,127,2)

IF m_anion_transporters_n4-INO4 AND m_anion_transporters_n10-INO4 THEN 000 OR 001 OR 100 OR 110
100: 0.353951 (1644,16,127,2)

IF m_anion_transporters_n10-INO4 THEN 000 OR 001 OR 010 OR 100 OR 110
100: 0.355436 (1644,39,127,4)

IF m_other_morphogenetic_activities_n7-RCS1 THEN 000 OR 010 OR 100 OR 101 OR 110
100: 0.355436 (1644,39,127,4)

IF SFF'-MCM1 AND MCM1'-MCM1 THEN 000 OR 001 OR 010 OR 100 OR 101 OR 110 OR 111
100: 0.358353 (1644,51,127,5)

IF m_other_mrna-transcription_activities_n20-INO4 THEN 000 OR 001 OR 010 OR 100 OR 110
010: 0.359686 (1644,25,140,3)

IF SWI5-SKN7 AND SWI5-SWI6 THEN 000 OR 010 OR 100 OR 110 OR 111
010: 0.359686 (1644,25,140,3)

IF m_other_morphogenetic_activities_n7-HOG1 THEN 000 OR 001 OR 010 OR 100 OR 110
010: 0.359686 (1644,25,140,3)

IF m_g-proteins_n12-INO4 THEN 000 OR 001 OR 010 OR 100 OR 101 OR 110 OR 111
110: 0.360162 (1644,83,115,7)

IF m_glyoxylate_cycle_n8-INO4 AND SWI5-INO4 THEN 000 OR 010 OR 100 OR 110
110: 0.362304 (1644,18,115,2)

IF m_g-proteins_n12-SKN7 AND CSRE-SKN7 THEN 000 OR 010 OR 100 OR 110 OR 111
110: 0.362304 (1644,18,115,2)

IF SFF'-RLM1 THEN 000 OR 010 OR 011 OR 100 OR 110 OR 111
110: 0.364436 (1644,70,115,6)

IF m_deoxyribonucleotide_metabolism_n10-INO4 THEN 000 OR 001 OR 010 OR 100 OR 101 OR 111
001: 0.365607 (1644,38,55,2)

IF m_other_protein-destination_activities_n7-SKN7 THEN 000 OR 001 OR 100 OR 110 OR 111
100: 0.36922 (1644,28,127,3)

IF m_metabolism_of_cyclic_and_unusual_nucleotides_n5-NDD1 THEN 000 OR 010 OR 100 OR 110 OR 111
100: 0.36922 (1644,28,127,3)

IF SFF-YER184C THEN 000 OR 001 OR 010 OR 100 OR 110
100: 0.36922 (1644,28,127,3)

IF ATRepeat-HAP1 THEN 000 OR 001 OR 010 OR 100
010: 0.369768 (1644,15,140,2)

IF SWI5-INO4 AND m_anion_transporters_n15-INO4 THEN 000 OR 001 OR 010 OR 100 OR 110

010: 0.369768 (1644,15,140,2)

IF SWI5-INO4 AND m_regulation_of_lipid_fatty-acid_and_isoprenoid_biosynthesis_n12.scn-INO4 THEN 000 OR 010 OR 110
010: 0.369768 (1644,15,140,2)

IF m_g-proteins_n12-SKN7 AND m_amino-acid_transporters_n11-SKN7 THEN 000 OR 001 OR 010 OR 110 OR 111
010: 0.369768 (1644,15,140,2)

IF m_anion_transporters_n4-MTH1 AND m_cell_death_n22-MTH1 THEN 000 OR 001 OR 010
010: 0.369768 (1644,15,140,2)

IF ABF1-PPR1 THEN 000 OR 010 OR 100 OR 110
010: 0.369768 (1644,15,140,2)

IF m_regulation_of_amino-acid_metabolism_n10-SKN7 THEN 000 OR 001 OR 010 OR 100
010: 0.369768 (1644,15,140,2)

IF m_RPE11-RAP1 THEN 000 OR 010 OR 111
010: 0.369768 (1644,15,140,2)

IF m_anion_transporters_n10-INO4 THEN 000 OR 001 OR 010 OR 100 OR 110
001: 0.377848 (1644,39,55,2)

IF SFF-FKH2 AND SWI5-SWI6 THEN 000 OR 001 OR 100 OR 110 OR 111
100: 0.382701 (1644,17,127,2)

IF m_allantoin_and_allantoate_transporters_n12-OAF1 THEN 000 OR 010 OR 100 OR 110
100: 0.382701 (1644,17,127,2)

IF m_other_nucleotide-metabolism_activities_n18-PHO2 THEN 000 OR 010 OR 100
100: 0.382701 (1644,17,127,2)

IF m_tricarboxylic-acid_pathway_n9-GAT1 THEN 000 OR 010 OR 100 OR 111
100: 0.382701 (1644,17,127,2)

IF MCM1-SWI4 THEN 000 OR 010 OR 100 OR 110 OR 111
100: 0.382701 (1644,17,127,2)

IF m_phosphate_transport_n18-ECM22 THEN 000 OR 001 OR 010 OR 100 OR 110
100: 0.382701 (1644,17,127,2)

IF SFF'-FKH1 AND SFF'-MCM1 AND SFF-FKH1 THEN 000 OR 010 OR 100 OR 110 OR 111
100: 0.382701 (1644,17,127,2)

IF m_other_cation_transporters_n14-RAP1 THEN 000 OR 010 OR 100 OR 110
100: 0.382701 (1644,17,127,2)

IF SFF'-MCM1 AND SFF-FKH1 THEN 000 OR 010 OR 100 OR 110 OR 111
100: 0.382701 (1644,17,127,2)

IF ABF1-SIG1 THEN 000 OR 010 OR 011 OR 100 OR 110
100: 0.382701 (1644,17,127,2)

IF m_regulation_of_lipid_fatty-acid_and_isoprenoid_biosynthesis_n16.scn-NDD1 THEN 000 OR 010 OR 100 OR 110
100: 0.382701 (1644,17,127,2)

IF SFF'-FKH1 AND MCM1'-MCM1 THEN 000 OR 010 OR 100 OR 110 OR 111
100: 0.382701 (1644,17,127,2)

IF m_other_nutritional-response_activities_n10-INO4 THEN 000 OR 001 OR 010 OR 100 OR 111
010: 0.3838 (1644,26,140,3)

IF m_osmosensing_n6-INO4 THEN 000 OR 010 OR 100 OR 101 OR 110
010: 0.3838 (1644,26,140,3)

IF m_drug_transporters_n10-MET4 THEN 000 OR 010 OR 100 OR 110 OR 111
110: 0.388141 (1644,19,115,2)

IF m_phosphate_transport_n8-INO4 THEN 000 OR 100 OR 110
110: 0.388141 (1644,19,115,2)

IF m_g-proteins_n12-INO4 AND m_anion_transporters_n15-INO4 THEN 000 OR 001 OR 010 OR 100 OR 110 OR 111
110: 0.388141 (1644,19,115,2)

IF SCB-AZF1 THEN 000 OR 010 OR 011 OR 100 OR 110 OR 111
100: 0.39186 (1644,41,127,4)

IF SFF-HIR1 THEN 000 OR 001 OR 010 OR 100 OR 101 OR 110
110: 0.39701 (1644,59,115,5)

IF m_c-compound_and_carbohydrate_utilization_n9-RAP1 THEN 000 OR 001 OR 010 OR 111
010: 0.401225 (1644,16,140,2)

IF m_peroxisomal_organization_n28-SKN7 THEN 000 OR 010 OR 110
010: 0.401225 (1644,16,140,2)

IF m_g-proteins_n11-UME6 AND SCB-UME6 THEN 000 OR 001 OR 010 OR 110
010: 0.401225 (1644,16,140,2)

IF m_drug_transporters_n7-MET4 THEN 000 OR 010 OR 100 OR 110
010: 0.401225 (1644,16,140,2)

IF m_lipid_transporters_n8-MET4 THEN 000 OR 010 OR 100 OR 110
010: 0.401225 (1644,16,140,2)

IF m_homeostasis_of_other_ions_n30-INO4 THEN 000 OR 001 OR 010 OR 100 OR 110
010: 0.401225 (1644,16,140,2)

IF m_g-proteins_n12-INO4 AND m_other_nutritional-response_activities_n10-INO4 THEN 000 OR 001 OR 010 OR 100
010: 0.401225 (1644,16,140,2)

IF m_nucleotide_transport_n9-MCM1 THEN 000 OR 010 OR 100 OR 101 OR 110
010: 0.401225 (1644,16,140,2)

IF RAP1-ROX1 THEN 000 OR 001 OR 010
010: 0.401225 (1644,16,140,2)

IF MCM1-YOX1 THEN 000 OR 010 OR 110 OR 111
010: 0.401225 (1644,16,140,2)

IF m_allantoin_and_allantoate_transporters_n7-SFP1 THEN 000 OR 010 OR 100 OR 110 OR 111
010: 0.407712 (1644,27,140,3)

IF m_pentose-phosphate_pathway_n23-RAP1 THEN 000 OR 001 OR 010 OR 100 OR 110
010: 0.407712 (1644,27,140,3)

IF m_g-proteins_n12-INO4 AND SWI5-INO4 THEN 000 OR 001 OR 010 OR 100 OR 110
100: 0.409986 (1644,42,127,4)

IF m_regulation_of_amino-acid_metabolism_n15-INO4 THEN 000 OR 010 OR 100
100: 0.410901 (1644,18,127,2)

IF m_other_pheromone_response_activities_n8-INO4 THEN 000 OR 001 OR 010 OR 100 OR 111
100: 0.410901 (1644,18,127,2)

IF m_organization_of_chromosome_structure_n17-SKN7 THEN 000 OR 001 OR 100 OR 110 OR 111
100: 0.410901 (1644,18,127,2)

IF m_anion_transporters_n4-INO4 THEN 000 OR 001 OR 100 OR 110
100: 0.412527 (1644,30,127,3)

IF m_phosphate_transport_n13-GAT1 THEN 000 OR 001 OR 010 OR 100 OR 110
110: 0.413517 (1644,20,115,2)

IF m_PNDE6-UPC2 THEN 000 OR 001 OR 100 OR 110
110: 0.413517 (1644,20,115,2)

IF m_organization_of_chromosome_structure_n17-INO4 THEN 000 OR 001 OR 100 OR 101 OR 110
110: 0.413517 (1644,20,115,2)

IF m_g-proteins_n12-SKN7 THEN 000 OR 001 OR 010 OR 100 OR 110 OR 111
110: 0.416449 (1644,74,115,6)

IF SWI5-SWI6 THEN 000 OR 001 OR 010 OR 011 OR 100 OR 101 OR 110 OR 111
100: 0.422105 (1644,55,127,5)

IF m_g-proteins_n11-INO4 THEN 000 OR 001 OR 010 OR 100 OR 101 OR 110
010: 0.424101 (1644,50,140,5)

IF SFF'-MCM1 AND m_other_morphogenetic_activities_n7-MCM1 THEN 000 OR 001 OR 010 OR 011 OR 100 OR 101 OR 110 OR 111
100: 0.428007 (1644,43,127,4)

IF ALPHA1'-YBR239c AND ALPHA1'-YER051w THEN 000 OR 001 OR 010 OR 100 OR 110
010: 0.428071 (1644,39,140,4)

IF m_lipid_and_fatty-acid_transport_n11-ROX1 THEN 000 OR 010 OR 100 OR 110 OR 111
110: 0.429059 (1644,34,115,3)

IF SFF-HOG1 THEN 000 OR 001 OR 010 OR 100 OR 110
010: 0.431349 (1644,28,140,3)

IF SFF-YER184C THEN 000 OR 001 OR 010 OR 100 OR 110
010: 0.431349 (1644,28,140,3)

IF m_tricarboxylic-acid_pathway_n9-GAT1 THEN 000 OR 010 OR 100 OR 111
010: 0.431935 (1644,17,140,2)

IF SFF'-FKH1 AND MCM1'-MCM1 THEN 000 OR 010 OR 100 OR 110 OR 111
010: 0.431935 (1644,17,140,2)

IF m_phosphate_transport_n18-ECM22 THEN 000 OR 001 OR 010 OR 100 OR 110
010: 0.431935 (1644,17,140,2)

IF m_allantoin_and_allantoate_transporters_n12-OAF1 THEN 000 OR 010 OR 100 OR 110
010: 0.431935 (1644,17,140,2)

IF mPROTEOL18(m_proteolysis_n18)-MBP1 AND mPROTEOL18(m_proteolysis_n18)-SWI6 THEN 000 OR 010 OR 100 OR 110 OR 111
010: 0.431935 (1644,17,140,2)

IF m_other_cation_transporters_n14-RAP1 THEN 000 OR 010 OR 100 OR 110
010: 0.431935 (1644,17,140,2)

IF m_MERE4-MTH1 AND m_cell_death_n22-MTH1 THEN 000 OR 001 OR 010 OR 100
010: 0.431935 (1644,17,140,2)

IF RAP1-RAP1 AND RAP1-SMP1 THEN 000 OR 001 OR 010
010: 0.431935 (1644,17,140,2)

IF m_ion_transporters_n4-INO4 THEN 000 OR 010 OR 100
010: 0.431935 (1644,17,140,2)

IF m_RPE17-RGM1 THEN 000 OR 001 OR 010 OR 111
010: 0.431935 (1644,17,140,2)

IF m_regulation_of_lipid_fatty-acid_and_isoprenoid_biosynthesis_n16.scn-NDD1 THEN 000 OR 010 OR 100 OR 110
010: 0.431935 (1644,17,140,2)

IF m_other_energy_generation_activities_n12-FKH1 AND SFF-FKH1 THEN 000 OR 010 OR 110
010: 0.431935 (1644,17,140,2)

IF m_g-proteins_n12-INO4 AND m_nutritional_response_pathway_n8-INO4 THEN 000 OR 001 OR 010 OR 100
010: 0.431935 (1644,17,140,2)

IF RAP1-SMP1 THEN 000 OR 001 OR 010
010: 0.431935 (1644,17,140,2)

IF m_glycolysis_and_gluconeogenesis_n4-INO4 THEN 000 OR 001 OR 010 OR 100 OR 110
010: 0.431935 (1644,17,140,2)

IF ECB-MCM1 THEN 000 OR 010 OR 100 OR 110 OR 111

100: 0.433855 (1644,31,127,3)

IF MCM1'-MCM1 AND m_other_morphogenetic_activities_n7-MCM1 THEN 000 OR 001 OR 010 OR 100 OR 101 OR 110
100: 0.433855 (1644,31,127,3)

IF SFF'-RTG3 THEN 000 OR 001 OR 010 OR 100 OR 101 OR 110
010: 0.43579 (1644,62,140,6)

IF m_rRSE10-GAT1 THEN 000 OR 001 OR 010 OR 100 OR 110
110: 0.43837 (1644,21,115,2)

IF m_other_mrna-transcription_activities_n11-SWI6 THEN 000 OR 010 OR 100 OR 101 OR 110 OR 111
110: 0.43837 (1644,21,115,2)

IF m_other_energy_generation_activities_n4-UME6 THEN 000 OR 001 OR 010 OR 100 OR 110
100: 0.438465 (1644,19,127,2)

IF m_ionic_homeostasis_n6-SWI6 THEN 000 OR 010 OR 100 OR 101 OR 110
100: 0.438465 (1644,19,127,2)

IF m_g-proteins_n12-INO4 AND m_anion_transporters_n15-INO4 THEN 000 OR 001 OR 010 OR 100 OR 110 OR 111
100: 0.438465 (1644,19,127,2)

IF m_anion_transporters_n17-SKN7 THEN 000 OR 001 OR 100
100: 0.438465 (1644,19,127,2)

IF m_g-proteins_n12-INO4 AND m_phosphate_transport_n18-INO4 THEN 000 OR 001 OR 010 OR 100 OR 110 OR 111
100: 0.438465 (1644,19,127,2)

IF m_g-proteins_n12-SKN7 AND m_RRSE3-SKN7 THEN 000 OR 010 OR 100 OR 110
100: 0.438465 (1644,19,127,2)

IF SFF'-FKH2 AND SWI5-SWI6 THEN 000 OR 001 OR 010 OR 100 OR 110 OR 111
100: 0.438465 (1644,19,127,2)

IF RPN4-ABF1 THEN 000 OR 100
100: 0.438465 (1644,19,127,2)

IF PDR-SUT1 THEN 000 OR 010 OR 100 OR 110
100: 0.438465 (1644,19,127,2)

IF m_phosphate_transport_n8-INO4 THEN 000 OR 100 OR 110
100: 0.438465 (1644,19,127,2)

IF SFF'-MCM1 AND MCM1'-NDD1 THEN 000 OR 010 OR 100 OR 110 OR 111
100: 0.438465 (1644,19,127,2)

IF SFF-GAL3 THEN 000 OR 001 OR 010 OR 100 OR 110
110: 0.452242 (1644,49,115,4)

IF mRRPE-ABF1 AND ABF1-ABF1 THEN 000 OR 001 OR 010 OR 100 OR 110
010: 0.454646 (1644,29,140,3)

IF CSRE-UME6 THEN 000 OR 001 OR 010 OR 100 OR 110
100: 0.4549 (1644,32,127,3)

IF m_other_signal-transduction_activities_n8-RAP1 THEN 000 OR 001 OR 010 OR 100 OR 110
100: 0.4549 (1644,32,127,3)

IF SFF'-FKH1 AND SFF'-FKH2 THEN 000 OR 010 OR 100 OR 110 OR 111
010: 0.458928 (1644,52,140,5)

IF m_cell_death_n16-GAT1 THEN 000 OR 010 OR 100
010: 0.461797 (1644,18,140,2)

IF m_other_mrna-transcription_activities_n11-MTH1 THEN 000 OR 010 OR 100 OR 110
010: 0.461797 (1644,18,140,2)

IF ATRepeat-UME6 THEN 000 OR 010 OR 100 OR 110
010: 0.461797 (1644,18,140,2)

IF m_other_mrna-transcription_activities_n20-PIP2 THEN 000 OR 001 OR 010
010: 0.461797 (1644,18,140,2)

IF m_tricarboxylic-acid_pathway_n9-MTH1 THEN 000 OR 001 OR 010 OR 100 OR 111
010: 0.461797 (1644,18,140,2)

IF m_other_pheromone_response_activities_n8-INO4 THEN 000 OR 001 OR 010 OR 100 OR 111
010: 0.461797 (1644,18,140,2)

IF m_anion_transporters_n15-GAT1 THEN 000 OR 001 OR 010 OR 100 OR 101 OR 110
110: 0.462649 (1644,22,115,2)

IF m_MERE4-SKN7 THEN 000 OR 001 OR 010 OR 100 OR 110
110: 0.462649 (1644,22,115,2)

IF m_peroxisomal_transport_n22-TOS8 THEN 000 OR 001 OR 100 OR 110 OR 111
110: 0.462649 (1644,22,115,2)

IF m_other_mrna-transcription_activities_n20-XBP1 THEN 000 OR 001 OR 100 OR 110
110: 0.462649 (1644,22,115,2)

IF m_phosphate_transport_n18-ASH1 THEN 000 OR 010 OR 100 OR 101 OR 110 OR 111
110: 0.462649 (1644,22,115,2)

IF m_allantoin_and_allantoate_transporters_n13-STB2 THEN 000 OR 100 OR 110
110: 0.462649 (1644,22,115,2)

IF SFF-SIP3 THEN 000 OR 001 OR 010 OR 100 OR 110
100: 0.463608 (1644,45,127,4)

IF m_g-proteins_n12-INO4 AND m_osmosensing_n6-INO4 THEN 000 OR 010 OR 100 OR 101 OR 110
100: 0.465321 (1644,20,127,2)

IF PAC-REB1 THEN 000 OR 001 OR 010 OR 100 OR 110
100: 0.465321 (1644,20,127,2)

IF m_other_signal-transduction_activities_n8-INO4 THEN 000 OR 001 OR 100 OR 110
100: 0.465321 (1644,20,127,2)

IF MCM1'-MCM1 AND MCM1'-NDD1 THEN 000 OR 010 OR 100 OR 110 OR 111
100: 0.465321 (1644,20,127,2)

IF m_phosphate_transport_n13-GAT1 THEN 000 OR 001 OR 010 OR 100 OR 110
100: 0.465321 (1644,20,127,2)

IF m_lipid_and_fatty-acid_transport_n11-SKN7 THEN 000 OR 010 OR 100 OR 110 OR 111
100: 0.475623 (1644,33,127,3)

IF m_g-proteins_n12-NRG1 THEN 000 OR 001 OR 010 OR 100 OR 110
010: 0.476133 (1644,53,140,5)

IF SFF-HIR1 THEN 000 OR 001 OR 010 OR 100 OR 101 OR 110
100: 0.484768 (1644,59,127,5)

IF m_cell_death_n22-TOS8 THEN 000 OR 001 OR 010 OR 100 OR 110
110: 0.48551 (1644,37,115,3)

IF m_allantoin_and_allantoate_transporters_n7-STE12 THEN 000 OR 001 OR 010 OR 100 OR 110
110: 0.486312 (1644,23,115,2)

IF m_g-proteins_n12-INO4 AND m_g-proteins_n11-INO4 THEN 000 OR 001 OR 010 OR 100 OR 101 OR 110
110: 0.486312 (1644,23,115,2)

IF m_phosphate_transport_n13-SKN7 THEN 000 OR 001 OR 010 OR 100 OR 110
110: 0.486312 (1644,23,115,2)

IF m_g-proteins_n11-INO4 AND SWI5-INO4 THEN 000 OR 001 OR 010 OR 100 OR 110
110: 0.486312 (1644,23,115,2)

IF m_mitochondrial_biogenesis_n5-INO4 THEN 000 OR 010 OR 100 OR 101 OR 110
110: 0.486312 (1644,23,115,2)

IF m_other_morphogenetic_activities_n7-MDS3 THEN 000 OR 001 OR 010 OR 100 OR 101 OR 110
010: 0.486694 (1644,42,140,4)

IF m_g-proteins_n12-INO4 AND SWI5-INO4 THEN 000 OR 001 OR 010 OR 100 OR 110
010: 0.486694 (1644,42,140,4)

IF m_g-proteins_n12-SKN7 AND m_RRSE3-SKN7 THEN 000 OR 010 OR 100 OR 110
010: 0.490732 (1644,19,140,2)

IF m_g-proteins_n12-INO4 AND m_glyoxylate_cycle_n8-INO4 THEN 000 OR 001 OR 010 OR 100 OR 110 OR 111
010: 0.490732 (1644,19,140,2)

IF SWI5-INO4 AND m_anion_transporters_n10-INO4 THEN 000 OR 001 OR 010 OR 100
010: 0.490732 (1644,19,140,2)

IF m_g-proteins_n12-INO4 AND m_anion_transporters_n15-INO4 THEN 000 OR 001 OR 010 OR 100 OR 110 OR 111
010: 0.490732 (1644,19,140,2)

IF m_g-proteins_n12-INO4 AND m_phosphate_transport_n18-INO4 THEN 000 OR 001 OR 010 OR 100 OR 110 OR 111
010: 0.490732 (1644,19,140,2)

IF m_drug_transporters_n10-MET4 THEN 000 OR 010 OR 100 OR 110 OR 111
010: 0.490732 (1644,19,140,2)

IF m_other_morphogenetic_activities_n7-MDS3 AND ALPHA1'-YER051w THEN 000 OR 001 OR 010 OR 100 OR 101 OR 110
010: 0.490732 (1644,19,140,2)

IF m_regulation_of_lipid_fatty-acid_and_isoprenoid_biosynthesis_n8.scn-GAT1 THEN 000 OR 001 OR 010 OR 100 OR 110
100: 0.491415 (1644,21,127,2)

IF SFF'-SIP3 AND SFF-RPI1 THEN 000 OR 001 OR 100 OR 110
100: 0.491415 (1644,21,127,2)

IF SFF-RPI1 AND SFF-SIP3 THEN 000 OR 001 OR 100 OR 110
100: 0.491415 (1644,21,127,2)

IF MCM1'-SWI4 AND SCB-SWI4 THEN 000 OR 010 OR 100 OR 110 OR 111
100: 0.491415 (1644,21,127,2)

IF m_deoxyribonucleotide_metabolism_n23-MTH1 THEN 000 OR 001 OR 010 OR 100 OR 110
100: 0.491415 (1644,21,127,2)

IF m_other_mrna-transcription_activities_n11-SWI6 THEN 000 OR 010 OR 100 OR 101 OR 110 OR 111
100: 0.491415 (1644,21,127,2)

IF m_amino-acid_transport_n3-SKN7 THEN 000 OR 010 OR 100
100: 0.491415 (1644,21,127,2)

IF m_allantoin_and_allantoate_transporters_n7-GTS1 THEN 000 OR 100 OR 101
100: 0.491415 (1644,21,127,2)

IF MCM1-NDD1 AND MCM1'-NDD1 THEN 000 OR 010 OR 100 OR 110 OR 111
100: 0.491415 (1644,21,127,2)

IF SWI5-INO4 AND SWI5-SKN7 THEN 000 OR 001 OR 010 OR 100 OR 110
100: 0.491415 (1644,21,127,2)

IF mPROTEOL18(m_proteolysis_n18)-UME6 THEN 000 OR 010 OR 100 OR 110
100: 0.491415 (1644,21,127,2)

IF m_rRSE10-GAT1 THEN 000 OR 001 OR 010 OR 100 OR 110
100: 0.491415 (1644,21,127,2)

IF SFF'-FKH2 AND MCM1-FKH2 THEN 000 OR 010 OR 100 OR 110 OR 111
100: 0.491415 (1644,21,127,2)

IF m_biogenesis_of_chromosome_structure_n9-RAP1 THEN 000 OR 001 OR 100 OR 101 OR 110

100: 0.495984 (1644,34,127,3)

IF m_lipid_and_fatty-acid_transport_n11-MTH1 THEN 000 OR 001 OR 010 OR 100 OR 110 OR 111
100: 0.495984 (1644,34,127,3)

IF m_other_protein-destination_activities_n7-XBP1 THEN 000 OR 001 OR 010 OR 100 OR 110
100: 0.495984 (1644,34,127,3)

IF m_phosphate_transport_n18-GAT1 THEN 000 OR 001 OR 010 OR 100 OR 101 OR 110 OR 111
100: 0.495984 (1644,34,127,3)

IF SFF-RTG3 THEN 000 OR 001 OR 010 OR 100 OR 110
110: 0.499599 (1644,52,115,4)

IF SWI5-KSS1 THEN 000 OR 001 OR 010 OR 011 OR 100 OR 110
010: 0.499999 (1644,31,140,3)

IF m_regulation_of_lipid_fatty-acid_and_isoprenoid_biosynthesis_n12.scn-GAT1 THEN 000 OR 100 OR 110
110: 0.509326 (1644,24,115,2)

IF m_glyoxylate_cycle_n8-UME6 THEN 000 OR 001 OR 010 OR 100 OR 110
110: 0.509326 (1644,24,115,2)

IF m_lipid_and_fatty-acid_transport_n7-SKN7 THEN 000 OR 010 OR 110 OR 111
110: 0.509326 (1644,24,115,2)

IF m_other_mrna-transcription_activities_n20-XBP1 THEN 000 OR 001 OR 100 OR 110
100: 0.516704 (1644,22,127,2)

IF SFF'-FKH1 AND SFF'-MCM1 THEN 000 OR 010 OR 100 OR 110 OR 111
100: 0.516704 (1644,22,127,2)

IF SFF'-FKH1 AND m_pentose-phosphate_pathway_n14-FKH1 THEN 000 OR 010 OR 100 OR 110 OR 111
100: 0.516704 (1644,22,127,2)

IF SFF'-FKH2 AND MCM1'-NDD1 AND SFF-FKH2 THEN 000 OR 001 OR 010 OR 100 OR 110 OR 111
100: 0.516704 (1644,22,127,2)

IF m_allantoin_and_allantoate_transporters_n13-STB2 THEN 000 OR 100 OR 110
100: 0.516704 (1644,22,127,2)

IF m_regulation_of_lipid_fatty-acid_and_isoprenoid_biosynthesis_n22.scn-INO4 THEN 000 OR 010 OR 100 OR 111
010: 0.51868 (1644,20,140,2)

IF m_phosphate_transport_n13-GAT1 THEN 000 OR 001 OR 010 OR 100 OR 110
010: 0.51868 (1644,20,140,2)

IF PHO-INO4 THEN 000 OR 001 OR 010 OR 100 OR 101
010: 0.51868 (1644,20,140,2)

IF m_g-proteins_n12-INO4 AND m_osmosensing_n6-INO4 THEN 000 OR 010 OR 100 OR 101 OR 110
010: 0.51868 (1644,20,140,2)

IF m_PNDE6-SFP1 THEN 000 OR 010 OR 100 OR 110
010: 0.51868 (1644,20,140,2)

IF ALPHA1-SFP1 THEN 000 OR 001 OR 010 OR 100 OR 110
001: 0.526349 (1644,52,55,2)

IF m_nutritional_response_pathway_n8-XBP1 THEN 000 OR 001 OR 010 OR 100 OR 110
110: 0.531665 (1644,25,115,2)

IF m_biogenesis_of_chromosome_structure_n9-REB1 THEN 000 OR 001 OR 010 OR 100 OR 110 OR 111
100: 0.541156 (1644,23,127,2)

IF MCM1'-NDD1 AND SFF-FKH2 THEN 000 OR 001 OR 010 OR 100 OR 110 OR 111
100: 0.541156 (1644,23,127,2)

IF m_phosphate_transport_n13-SKN7 THEN 000 OR 001 OR 010 OR 100 OR 110
100: 0.541156 (1644,23,127,2)

IF m_g-proteins_n12-INO4 AND m_g-proteins_n11-INO4 THEN 000 OR 001 OR 010 OR 100 OR 101 OR 110
100: 0.541156 (1644,23,127,2)

IF MCM1-FKH2 THEN 000 OR 010 OR 100 OR 110 OR 111
100: 0.541156 (1644,23,127,2)

IF m_lipid_and_fatty-acid_transport_n11-SKN7 THEN 000 OR 010 OR 100 OR 110 OR 111
010: 0.543399 (1644,33,140,3)

IF m_glyoxylate_cycle_n8-MTH1 THEN 000 OR 001 OR 010 OR 110
010: 0.543399 (1644,33,140,3)

IF LYS14-SWI6 THEN 000 OR 010 OR 100 OR 110
010: 0.5456 (1644,21,140,2)

IF m_mitochondrial_biogenesis_n5-GAT1 THEN 000 OR 001 OR 010 OR 110
010: 0.5456 (1644,21,140,2)

IF m_phosphate_transport_n18-YAP6 THEN 000 OR 001 OR 010 OR 011 OR 100 OR 110 OR 111
010: 0.5456 (1644,21,140,2)

IF m_anion_transporters_n22-RAP1 THEN 000 OR 001 OR 010 OR 100 OR 110
010: 0.5456 (1644,21,140,2)

IF m_anion_transporters_n4-OAF1 THEN 000 OR 001 OR 010
010: 0.5456 (1644,21,140,2)

IF m_amino-acid_transport_n20-INO4 THEN 000 OR 001 OR 010 OR 100 OR 110
010: 0.5456 (1644,21,140,2)

IF m_RPE17-RAP1 THEN 000 OR 001 OR 010 OR 100
010: 0.5456 (1644,21,140,2)

IF GCN4-GCN4 THEN 000 OR 001 OR 010 OR 100 OR 110
010: 0.5456 (1644,21,140,2)

IF m_metabolism_of_cyclic_and_unusual_nucleotides_n5-YAP1 THEN 000 OR 001 OR 010 OR 100 OR 110
110: 0.553311 (1644,26,115,2)

IF m_other_mrna-transcription_activities_n20-SKN7 THEN 000 OR 001 OR 010 OR 100 OR 110
110: 0.553311 (1644,26,115,2)

IF ALPHA1'-YBR239c THEN 000 OR 001 OR 010 OR 100 OR 110
010: 0.559077 (1644,58,140,5)

IF REB1-REB1 THEN 000 OR 001 OR 010 OR 100 OR 110
010: 0.560835 (1644,46,140,4)

IF SWI5-RIM101 THEN 000 OR 010 OR 100 OR 110
010: 0.560835 (1644,46,140,4)

IF m_pentose-phosphate_pathway_n14-MTH1 THEN 000 OR 010 OR 100 OR 110
010: 0.564277 (1644,34,140,3)

IF m_anion_transporters_n15-MTH1 THEN 000 OR 001 OR 100 OR 110
100: 0.564747 (1644,24,127,2)

IF m_LFTE17-SKN7 THEN 000 OR 010 OR 100 OR 110 OR 111
100: 0.564747 (1644,24,127,2)

IF m_glyoxylate_cycle_n8-UME6 THEN 000 OR 001 OR 010 OR 100 OR 110
100: 0.564747 (1644,24,127,2)

IF m_regulation_of_lipid_fatty-acid_and_isoprenoid_biosynthesis_n12.scn-GAT1 THEN 000 OR 100 OR 110
100: 0.564747 (1644,24,127,2)

IF m_MERE4-SKN7 THEN 000 OR 001 OR 010 OR 100 OR 110
010: 0.571462 (1644,22,140,2)

IF m_other_energy_generation_activities_n12-FKH1 THEN 000 OR 010 OR 100 OR 110
010: 0.571462 (1644,22,140,2)

IF m_RRSE3-SWI4 THEN 000 OR 010 OR 100 OR 101 OR 110
010: 0.571462 (1644,22,140,2)

IF SFF'-HIR1 THEN 000 OR 001 OR 010 OR 100 OR 101 OR 110
100: 0.573909 (1644,65,127,5)

IF MCM1'-NDD1 THEN 000 OR 001 OR 010 OR 100 OR 110 OR 111
100: 0.580894 (1644,52,127,4)

IF m_glyoxylate_cycle_n11-UME6 THEN 000 OR 010 OR 100
010: 0.584573 (1644,35,140,3)

IF SFF'-FKH2 AND MCM1'-NDD1 THEN 000 OR 001 OR 010 OR 100 OR 110 OR 111
100: 0.587465 (1644,25,127,2)

IF SWI5-SKN7 AND SWI5-SWI6 THEN 000 OR 010 OR 100 OR 110 OR 111
100: 0.587465 (1644,25,127,2)

IF m_metabolism_of_cyclic_and_unusual_nucleotides_n5-NDD1 THEN 000 OR 010 OR 100 OR 110 OR 111
110: 0.594476 (1644,28,115,2)

IF m_anion_transporters_n15-INO4 THEN 000 OR 001 OR 010 OR 100 OR 110 OR 111
110: 0.594476 (1644,28,115,2)

IF m_allantoin_and_allantoate_transporters_n7-STE12 THEN 000 OR 001 OR 010 OR 100 OR 110
010: 0.596251 (1644,23,140,2)

IF SFF'-FKH2 AND SFF'-MCM1 THEN 000 OR 010 OR 011 OR 100 OR 110 OR 111
010: 0.596251 (1644,23,140,2)

IF m_g-proteins_n12-INO4 AND m_g-proteins_n11-INO4 THEN 000 OR 001 OR 010 OR 100 OR 101 OR 110
010: 0.596251 (1644,23,140,2)

IF m_mitochondrial_biogenesis_n5-INO4 THEN 000 OR 010 OR 100 OR 101 OR 110
010: 0.596251 (1644,23,140,2)

IF m_phosphate_transport_n13-SKN7 THEN 000 OR 001 OR 010 OR 100 OR 110
010: 0.596251 (1644,23,140,2)

IF m_g-proteins_n11-INO4 AND SWI5-INO4 THEN 000 OR 001 OR 010 OR 100 OR 110
010: 0.596251 (1644,23,140,2)

IF MCM1-FKH2 THEN 000 OR 010 OR 100 OR 110 OR 111
010: 0.596251 (1644,23,140,2)

IF m_glyoxylate_cycle_n8-INO4 THEN 000 OR 001 OR 010 OR 100 OR 110 OR 111
010: 0.604266 (1644,36,140,3)

IF SCB-SWI4 THEN 000 OR 010 OR 100 OR 110 OR 111
100: 0.609031 (1644,40,127,3)

IF MCM1-NDD1 THEN 000 OR 010 OR 100 OR 110 OR 111
100: 0.609302 (1644,26,127,2)

IF Ume6(URS1)-UME6 THEN 000 OR 001 OR 010 OR 100
100: 0.609302 (1644,26,127,2)

IF m_other_morphogenetic_activities_n7-RTG1 THEN 000 OR 010 OR 011 OR 100 OR 110
010: 0.612515 (1644,49,140,4)

IF SFF-MIG1 THEN 000 OR 001 OR 010 OR 100 OR 110
001: 0.614741 (1644,61,55,2)

IF m_LFTE17-UME6 THEN 000 OR 001 OR 010 OR 100 OR 101 OR 110
010: 0.61996 (1644,24,140,2)

IF PHO4-CBF1 THEN 000 OR 001 OR 010 OR 100

010: 0.61996 (1644,24,140,2)

IF m_lipid_and_fatty-acid_transport_n7-SKN7 THEN 000 OR 010 OR 110 OR 111
010: 0.61996 (1644,24,140,2)

IF m_deoxyribonucleotide_metabolism_n10-SKN7 THEN 000 OR 001 OR 010 OR 100 OR 111
010: 0.61996 (1644,24,140,2)

IF m_phosphate_transport_n18-INO4 THEN 000 OR 001 OR 010 OR 100 OR 110 OR 111
010: 0.623341 (1644,37,140,3)

IF m_allantoin_and_allantoate_transporters_n7-SFP1 THEN 000 OR 010 OR 100 OR 110 OR 111
100: 0.630258 (1644,27,127,2)

IF m_pentose-phosphate_pathway_n14-FKH1 THEN 000 OR 010 OR 100 OR 110 OR 111
100: 0.630258 (1644,27,127,2)

IF m_nutritional_response_pathway_n8-INO4 THEN 000 OR 001 OR 010 OR 100 OR 110
100: 0.630258 (1644,27,127,2)

IF m_osmosensing_n6-ROX1 THEN 000 OR 001 OR 010 OR 100 OR 110 OR 111
100: 0.630258 (1644,27,127,2)

IF m_anion_transporters_n4-INO4 THEN 000 OR 001 OR 100 OR 110
110: 0.632775 (1644,30,115,2)

IF m_MERE4-MTH1 THEN 000 OR 001 OR 010 OR 100 OR 110
110: 0.632775 (1644,30,115,2)

IF REB1-REB1 THEN 000 OR 001 OR 010 OR 100 OR 110
110: 0.636449 (1644,46,115,3)

IF SFF'-RLM1 THEN 000 OR 010 OR 011 OR 100 OR 110 OR 111
100: 0.64177 (1644,70,127,5)

IF m_nutritional_response_pathway_n8-XBP1 THEN 000 OR 001 OR 010 OR 100 OR 110
010: 0.642593 (1644,25,140,2)

IF m_lipid_and_fatty-acid_transport_n11-GAT1 THEN 000 OR 001 OR 010 OR 100 OR 110 OR 111
100: 0.642806 (1644,42,127,3)

IF m_other_morphogenetic_activities_n7-MDS3 THEN 000 OR 001 OR 010 OR 100 OR 101 OR 110
100: 0.642806 (1644,42,127,3)

IF SFF'-HIR1 THEN 000 OR 001 OR 010 OR 100 OR 101 OR 110
001: 0.649897 (1644,65,55,2)

IF m_RRSE3-INO4 THEN 000 OR 001 OR 010 OR 100 OR 110
100: 0.650338 (1644,28,127,2)

IF m_g-proteins_n12-IME4 THEN 000 OR 001 OR 010 OR 110
110: 0.650853 (1644,31,115,2)

IF SFF-RPI1 THEN 000 OR 001 OR 010 OR 011 OR 100 OR 101 OR 110 OR 111
001: 0.65829 (1644,66,55,2)

IF m_anion_transporters_n10-INO4 THEN 000 OR 001 OR 010 OR 100 OR 110
010: 0.659597 (1644,39,140,3)

IF m_cell_death_n22-MTH1 THEN 000 OR 001 OR 010 OR 100 OR 110
010: 0.659597 (1644,39,140,3)

IF SFF-RTG3 THEN 000 OR 001 OR 010 OR 100 OR 110
010: 0.660334 (1644,52,140,4)

IF Ume6(URS1)-UME6 THEN 000 OR 001 OR 010 OR 100
010: 0.664159 (1644,26,140,2)

IF m_other_mrna-transcription_activities_n20-SKN7 THEN 000 OR 001 OR 010 OR 100 OR 110
010: 0.664159 (1644,26,140,2)

IF m_other_protein-destination_activities_n7-MET4 THEN 000 OR 010 OR 100 OR 110
010: 0.664159 (1644,26,140,2)

IF m_cell_death_n22-SWI4 THEN 000 OR 010 OR 100 OR 101 OR 110 OR 111
010: 0.664159 (1644,26,140,2)

IF m_metabolism_of_cyclic_and_unusual_nucleotides_n5-YAP1 THEN 000 OR 001 OR 010 OR 100 OR 110
010: 0.664159 (1644,26,140,2)

IF CSRE-UME6 THEN 000 OR 001 OR 010 OR 100 OR 110
110: 0.668225 (1644,32,115,2)

IF m_other_signal-transduction_activities_n8-RAP1 THEN 000 OR 001 OR 010 OR 100 OR 110
110: 0.668225 (1644,32,115,2)

IF SFF'-HIR1 THEN 000 OR 001 OR 010 OR 100 OR 101 OR 110
110: 0.679971 (1644,65,115,4)

IF m_amino-acid_transporters_n11-SKN7 THEN 000 OR 001 OR 010 OR 110 OR 111
010: 0.684674 (1644,27,140,2)

IF m_nutritional_response_pathway_n8-INO4 THEN 000 OR 001 OR 010 OR 100 OR 110
010: 0.684674 (1644,27,140,2)

IF m_osmosensing_n6-ROX1 THEN 000 OR 001 OR 010 OR 100 OR 110 OR 111
010: 0.684674 (1644,27,140,2)

IF m_glyoxylate_cycle_n8-MTH1 THEN 000 OR 001 OR 010 OR 110
110: 0.6849 (1644,33,115,2)

IF m_lipid_and_fatty-acid_transport_n11-SKN7 THEN 000 OR 010 OR 100 OR 110 OR 111
110: 0.6849 (1644,33,115,2)

IF m_MERE4-MTH1 THEN 000 OR 001 OR 010 OR 100 OR 110
100: 0.68791 (1644,30,127,2)

IF m_anion_transporters_n15-INO4 THEN 000 OR 001 OR 010 OR 100 OR 110 OR 111
010: 0.704161 (1644,28,140,2)

IF SWI5-SKN7 THEN 000 OR 001 OR 010 OR 100 OR 110 OR 111
100: 0.70802 (1644,61,127,4)

IF m_lipid_and_fatty-acid_transport_n11-GAT1 THEN 000 OR 001 OR 010 OR 100 OR 110 OR 111
010: 0.70919 (1644,42,140,3)

IF m_RRSE3-SKN7 THEN 000 OR 010 OR 100 OR 110
010: 0.722644 (1644,29,140,2)

IF SFF'-RLM1 THEN 000 OR 010 OR 011 OR 100 OR 110 OR 111
010: 0.727237 (1644,70,140,5)

IF CSRE-SKN7 THEN 000 OR 001 OR 010 OR 100 OR 110 OR 111
100: 0.738037 (1644,33,127,2)

IF m_lipid_and_fatty-acid_transport_n11-ROX1 THEN 000 OR 010 OR 100 OR 110 OR 111
100: 0.753165 (1644,34,127,2)

IF m_g-proteins_n12-IME4 THEN 000 OR 001 OR 010 OR 110
010: 0.756713 (1644,31,140,2)

IF SFF-RPI1 THEN 000 OR 001 OR 010 OR 011 OR 100 OR 101 OR 110 OR 111
100: 0.765561 (1644,66,127,4)

IF CSRE-SUT1 THEN 000 OR 001 OR 010 OR 100 OR 110 OR 111
100: 0.767542 (1644,35,127,2)

IF SFF-RTG3 THEN 000 OR 001 OR 010 OR 100 OR 110
100: 0.780679 (1644,52,127,3)

IF m_other_protein-destination_activities_n7-XBP1 THEN 000 OR 001 OR 010 OR 100 OR 110
010: 0.801063 (1644,34,140,2)

IF m_lipid_and_fatty-acid_transport_n11-ROX1 THEN 000 OR 010 OR 100 OR 110 OR 111
010: 0.801063 (1644,34,140,2)

IF SFF-GZF3 THEN 000 OR 001 OR 010 OR 100 OR 110
110: 0.805118 (1644,60,115,3)

IF m_g-proteins_n11-UME6 THEN 000 OR 001 OR 010 OR 100 OR 110
100: 0.806413 (1644,38,127,2)

IF ALPHA1'-STB4 THEN 000 OR 001 OR 010 OR 011 OR 100 OR 110 OR 111
100: 0.822624 (1644,72,127,4)

IF SFF-RPI1 THEN 000 OR 001 OR 010 OR 011 OR 100 OR 101 OR 110 OR 111
010: 0.829525 (1644,66,140,4)

IF MCM1'-SWI4 THEN 000 OR 010 OR 100 OR 101 OR 110 OR 111
010: 0.835067 (1644,52,140,3)

IF m_phosphate_transport_n18-TOS8 THEN 000 OR 001 OR 010 OR 100 OR 110 OR 111
110: 0.845223 (1644,46,115,2)

IF m_phosphate_transport_n18-XBP1 THEN 000 OR 001 OR 010 OR 100 OR 110 OR 111
110: 0.862033 (1644,48,115,2)

IF ALPHA1-RGM1 THEN 000 OR 001 OR 010 OR 110 OR 111
110: 0.862033 (1644,48,115,2)

IF mRRPE-ABF1 THEN 000 OR 001 OR 010 OR 100 OR 110
010: 0.862385 (1644,55,140,3)

IF SFF'-RTG3 THEN 000 OR 001 OR 010 OR 100 OR 101 OR 110
100: 0.871874 (1644,62,127,3)

IF m_other_morphogenetic_activities_n7-FZF1 THEN 000 OR 001 OR 010 OR 100 OR 101 OR 110
100: 0.875589 (1644,45,127,2)

IF m_phosphate_transport_n18-TOS8 THEN 000 OR 001 OR 010 OR 100 OR 110 OR 111
100: 0.883383 (1644,46,127,2)

IF m_g-proteins_n12-NRG1 THEN 000 OR 001 OR 010 OR 100 OR 110
110: 0.897049 (1644,53,115,2)

IF m_phosphate_transport_n18-XBP1 THEN 000 OR 001 OR 010 OR 100 OR 110 OR 111
100: 0.897645 (1644,48,127,2)

IF RAP1-RAP1 THEN 000 OR 001 OR 010 OR 100 OR 110
100: 0.897846 (1644,66,127,3)

IF SFF-GAL3 THEN 000 OR 001 OR 010 OR 100 OR 110
100: 0.904155 (1644,49,127,2)

IF m_other_morphogenetic_activities_n7-FZF1 THEN 000 OR 001 OR 010 OR 100 OR 101 OR 110
010: 0.908533 (1644,45,140,2)

IF SFF-SIP3 THEN 000 OR 001 OR 010 OR 100 OR 110
010: 0.908533 (1644,45,140,2)

IF mRRPE-ABF1 THEN 000 OR 001 OR 010 OR 100 OR 110
110: 0.908607 (1644,55,115,2)

IF m_phosphate_transport_n18-TOS8 THEN 000 OR 001 OR 010 OR 100 OR 110 OR 111
010: 0.914988 (1644,46,140,2)

IF m_phosphate_transport_n18-XBP1 THEN 000 OR 001 OR 010 OR 100 OR 110 OR 111
010: 0.926644 (1644,48,140,2)

IF SFF-MIG1 THEN 000 OR 001 OR 010 OR 100 OR 110

110: 0.936451 (1644,61,115,2)

IF SFF'-SIP3 THEN 000 OR 001 OR 010 OR 100 OR 110
010: 0.936791 (1644,50,140,2)
