# Supplementary material for: Revealing cell cycle control by combining model-based detection of periodic expression with novel cis-regulatory descriptors
Source: BMC Syst Biol. 2007 Oct 16;1:45. doi: 10.1186/1752-0509-1-45 (PMC2200664; doi:10.1186/1752-0509-1-45)
Supplement: Additional file 3 — All hierarchies of cis-regulatory descriptor combinations. All hierarchies of cis-regulatory descriptor combinations. [file 1752-0509-1-45-S3.pdf]

All hierarchies of *cis*-regulatory descriptor combinations.

111: pINO4-phosphate_transport_n18 pSKN7-amino-acid_transporters_n11
 100: pINO4-phosphate_transport_n18
 010: pSKN7-amino-acid_transporters_n11
 010: pINO4-phosphate_transport_n18
111: pSWI4-amino-acid_transporters_n11 pINO4-phosphate_transport_n18
 010: pSWI4-amino-acid_transporters_n11
 100: pINO4-phosphate_transport_n18
 010: pINO4-phosphate_transport_n18
111: pSWI4-SCB pFKH1-SFF'
 010: pSWI4-SCB
 100: pSWI4-SCB
111: pSWI4-MCM1' pINO4-phosphate_transport_n18
 100: pSWI4-MCM1'
 100: pINO4-phosphate_transport_n18
 010: pINO4-phosphate_transport_n18
 010: pSWI4-MCM1'
111: pSWI4-SCB pINO4-phosphate_transport_n18
 010: pSWI4-SCB
 100: pINO4-phosphate_transport_n18
 010: pINO4-phosphate_transport_n18
 100: pSWI4-SCB
111: pSWI4-MCM1' pSKN7-amino-acid_transporters_n11
 100: pSWI4-MCM1'
 010: pSKN7-amino-acid_transporters_n11
 010: pSWI4-MCM1'
111: pSKN7-g-proteins_n12 pFKH1-SFF'
111: pSWI4-SCB pSKN7-amino-acid_transporters_n11
 010: pSWI4-SCB
 010: pSKN7-amino-acid_transporters_n11
 100: pSWI4-SCB
111: pSKN7-g-proteins_n12 pSKN7-lipid_and_fatty-acid_transport_n11
 010: pSKN7-lipid_and_fatty-acid_transport_n11
 100: pSKN7-lipid_and_fatty-acid_transport_n11
111: pSWI4-SCB pXBP1-phosphate_transport_n18
 001: pXBP1-phosphate_transport_n18
 010: pXBP1-phosphate_transport_n18
 010: pSWI4-SCB
 100: pXBP1-phosphate_transport_n18
 100: pSWI4-SCB
111: pSWI4-nutritional_response_pathway_n7 pXBP1-phosphate_transport_n18
 001: pXBP1-phosphate_transport_n18
 010: pXBP1-phosphate_transport_n18
 100: pXBP1-phosphate_transport_n18
111: pSWI6-nutritional_response_pathway_n7 pSWI4-glycolysis_and_gluconeogenesis_n27
 010: pSWI6-nutritional_response_pathway_n7
111: pXBP1-phosphate_transport_n18 pSWI6-nutritional_response_pathway_n7
 001: pXBP1-phosphate_transport_n18
 010: pSWI6-nutritional_response_pathway_n7
 010: pXBP1-phosphate_transport_n18
 100: pXBP1-phosphate_transport_n18
111: pSWI6-glycolysis_and_gluconeogenesis_n27 pSWI4-nutritional_response_pathway_n7
111: pSWI6-glycolysis_and_gluconeogenesis_n27 pSWI6-nutritional_response_pathway_n7
 010: pSWI6-nutritional_response_pathway_n7
111: pXBP1-phosphate_transport_n18 pSWI4-ion_transporters_n11
 001: pXBP1-phosphate_transport_n18
 010: pXBP1-phosphate_transport_n18
 100: pXBP1-phosphate_transport_n18
 100: pSWI4-ion_transporters_n11
111: pSWI6-ion_transporters_n11 pXBP1-phosphate_transport_n18
 001: pXBP1-phosphate_transport_n18
 010: pXBP1-phosphate_transport_n18
 100: pXBP1-phosphate_transport_n18
111: pSWI4-nutritional_response_pathway_n7 pSWI6-mPROTEOL18(proteolysis_n18)
 100: pSWI6-mPROTEOL18(proteolysis_n18)
 010: pSWI6-mPROTEOL18(proteolysis_n18)
111: pSWI6-glycolysis_and_gluconeogenesis_n27 pSWI4-MCM1'

```
  100: pSWI4-MCM1'
  010: pSWI4-MCM1'
111: pSWI6-ion_transporters_n11 pSWI4-MCM1' pSWI4-nutritional_response_pathway_n7
  100: pSWI4-MCM1'
  110: pSWI4-MCM1' pSWI4-nutritional_response_pathway_n7
    100: pSWI4-MCM1'
    010: pSWI4-MCM1'
  010: pSWI4-MCM1'
111: pSWI6-ion_transporters_n11 pSWI4-MCM1' pSWI6-mPROTEOL18(proteolysis_n18)
  100: pSWI4-MCM1'
  100: pSWI6-mPROTEOL18(proteolysis_n18)
  110: pSWI4-MCM1' pSWI6-mPROTEOL18(proteolysis_n18)
    100: pSWI4-MCM1'
    100: pSWI6-mPROTEOL18(proteolysis_n18)
    010: pSWI6-mPROTEOL18(proteolysis_n18)
    010: pSWI4-MCM1'
  010: pSWI4-MCM1' pSWI6-mPROTEOL18(proteolysis_n18)
  010: pSWI6-mPROTEOL18(proteolysis_n18)
  010: pSWI4-MCM1'
111: pSWI6-ion_transporters_n11 pSWI4-MCM1' pSWI4-SCB
  100: pSWI4-MCM1'
  010: pSWI4-MCM1' pSWI4-SCB
  010: pSWI4-SCB
  100: pSWI4-MCM1' pSWI4-SCB
  010: pSWI4-MCM1'
  110: pSWI4-MCM1' pSWI4-SCB
    100: pSWI4-MCM1'
    010: pSWI4-SCB
    010: pSWI4-MCM1'
    100: pSWI4-SCB
  100: pSWI4-SCB
111: pSWI6-ion_transporters_n11 pSWI4-SCB pSWI6-mPROTEOL18(proteolysis_n18)
  010: pSWI4-SCB pSWI6-mPROTEOL18(proteolysis_n18)
  010: pSWI4-SCB
  100: pSWI6-mPROTEOL18(proteolysis_n18)
  010: pSWI6-mPROTEOL18(proteolysis_n18)
  100: pSWI4-SCB
111: pSWI4-MCM1' pSWI4-SCB pSWI4-ion_transporters_n11
  100: pSWI4-MCM1'
  010: pSWI4-MCM1' pSWI4-SCB
  010: pSWI4-SCB
  100: pSWI4-MCM1' pSWI4-SCB
  100: pSWI4-ion_transporters_n11
  010: pSWI4-MCM1'
  110: pSWI4-MCM1' pSWI4-SCB
    100: pSWI4-MCM1'
    010: pSWI4-SCB
    010: pSWI4-MCM1'
    100: pSWI4-SCB
  100: pSWI4-SCB
111: pSWI4-MCM1' pSWI4-nutritional_response_pathway_n7 pSWI4-ion_transporters_n11
  100: pSWI4-MCM1'
  100: pSWI4-ion_transporters_n11
  110: pSWI4-MCM1' pSWI4-nutritional_response_pathway_n7
    100: pSWI4-MCM1'
    010: pSWI4-MCM1'
  010: pSWI4-MCM1'
111: pSWI6-mPROTEOL18(proteolysis_n18) pSWI6-nutritional_response_pathway_n7
  010: pSWI6-nutritional_response_pathway_n7
  100: pSWI6-mPROTEOL18(proteolysis_n18)
  010: pSWI6-mPROTEOL18(proteolysis_n18)
111: pSWI6-mPROTEOL18(proteolysis_n18) pSWI4-ion_transporters_n11
  100: pSWI6-mPROTEOL18(proteolysis_n18)
  100: pSWI4-ion_transporters_n11
  010: pSWI6-mPROTEOL18(proteolysis_n18)
111: pSWI4-MCM1' pSWI6-nutritional_response_pathway_n7
  100: pSWI4-MCM1'
  010: pSWI6-nutritional_response_pathway_n7
  010: pSWI4-MCM1'
111: pSWI4-MCM1' pXBP1-phosphate_transport_n18
```

100: pSWI4-MCM1'
001: pXBP1-phosphate_transport_n18
010: pXBP1-phosphate_transport_n18
100: pXBP1-phosphate_transport_n18
010: pSWI4-MCM1'
111: pSWI6-mPROTEOL18(proteolysis_n18) pXBP1-phosphate_transport_n18
001: pXBP1-phosphate_transport_n18
010: pXBP1-phosphate_transport_n18
100: pSWI6-mPROTEOL18(proteolysis_n18)
100: pXBP1-phosphate_transport_n18
010: pSWI6-mPROTEOL18(proteolysis_n18)
111: pSWI6-ion_transporters_n11 pSWI4-MCM1'
100: pSWI4-MCM1'
010: pSWI4-MCM1'
111: pSWI4-MCM1' pSWI4-ion_transporters_n11
100: pSWI4-MCM1'
100: pSWI4-ion_transporters_n11
010: pSWI4-MCM1'
111: pSWI4-SCB pSWI6-nutritional_response_pathway_n7
010: pSWI6-nutritional_response_pathway_n7
010: pSWI4-SCB
100: pSWI4-SCB
111: pSWI6-ion_transporters_n11 pSWI6-mPROTEOL18(proteolysis_n18)
100: pSWI6-mPROTEOL18(proteolysis_n18)
010: pSWI6-mPROTEOL18(proteolysis_n18)
111: pSWI6-ion_transporters_n11 pSWI6-nutritional_response_pathway_n7
010: pSWI6-nutritional_response_pathway_n7
111: pSWI6-nutritional_response_pathway_n7 pSWI4-ion_transporters_n11
010: pSWI6-nutritional_response_pathway_n7
100: pSWI4-ion_transporters_n11
111: pSWI6-ion_transporters_n11 pSWI4-nutritional_response_pathway_n7
111: pSWI6-ion_transporters_n11 pSWI4-ion_transporters_n11
100: pSWI4-ion_transporters_n11
111: pSWI6-ion_transporters_n11 pSWI4-SCB
010: pSWI4-SCB
100: pSWI4-SCB
111: pSWI4-MCM1' pSWI6-mPROTEOL18(proteolysis_n18)
100: pSWI4-MCM1'
100: pSWI6-mPROTEOL18(proteolysis_n18)
010: pSWI6-mPROTEOL18(proteolysis_n18)
010: pSWI4-MCM1'
111: pSWI4-SCB pSWI6-SWI5
010: pSWI4-SCB
100: pSWI6-SWI5
100: pSWI4-SCB
111: pSWI4-MCM1' pSWI4-glycolysis_and_gluconeogenesis_n27
100: pSWI4-MCM1'
010: pSWI4-MCM1'
111: pSWI4-SCB pSWI6-mPROTEOL18(proteolysis_n18)
010: pSWI4-SCB
100: pSWI6-mPROTEOL18(proteolysis_n18)
010: pSWI6-mPROTEOL18(proteolysis_n18)
100: pSWI4-SCB
111: pSWI4-MCM1' pSWI4-nutritional_response_pathway_n7
100: pSWI4-MCM1'
010: pSWI4-MCM1'
111: pSWI6-glycolysis_and_gluconeogenesis_n27 pSWI4-glycolysis_and_gluconeogenesis_n27
111: pSWI4-MCM1' pSWI4-SCB
100: pSWI4-MCM1'
010: pSWI4-SCB
010: pSWI4-MCM1'
100: pSWI4-SCB
111: pSWI6-ion_transporters_n11 pSWI6-mPROTEOL18(proteolysis_n18) pSWI6-nutritional_response_pathway_n7
010: pSWI6-nutritional_response_pathway_n7
100: pSWI6-mPROTEOL18(proteolysis_n18)
010: pSWI6-mPROTEOL18(proteolysis_n18)
111: pSWI4-MCM1' pSWI6-mPROTEOL18(proteolysis_n18) pSWI6-nutritional_response_pathway_n7
100: pSWI4-MCM1'
010: pSWI6-nutritional_response_pathway_n7
100: pSWI6-mPROTEOL18(proteolysis_n18)

110: pSWI4-MCM1' pSWI6-mPROTEOL18(proteolysis_n18)
100: pSWI4-MCM1'
100: pSWI6-mPROTEOL18(proteolysis_n18)
010: pSWI6-mPROTEOL18(proteolysis_n18)
010: pSWI4-MCM1'
110: pSWI4-MCM1' pSWI6-nutritional_response_pathway_n7
100: pSWI4-MCM1'
010: pSWI6-nutritional_response_pathway_n7
010: pSWI4-MCM1'
010: pSWI4-MCM1' pSWI6-mPROTEOL18(proteolysis_n18)
010: pSWI6-mPROTEOL18(proteolysis_n18)
010: pSWI4-MCM1'
111: pSWI4-SCB pSWI6-mPROTEOL18(proteolysis_n18) pSWI6-nutritional_response_pathway_n7
010: pSWI6-nutritional_response_pathway_n7
010: pSWI4-SCB pSWI6-mPROTEOL18(proteolysis_n18)
010: pSWI4-SCB
100: pSWI6-mPROTEOL18(proteolysis_n18)
010: pSWI6-mPROTEOL18(proteolysis_n18)
100: pSWI4-SCB
111: pFKH2-deoxyribonucleotide_metabolisn4 pACE2-allantoin_and_allantoate_transporters_n7
111: pMCM1-SFF' pACE2-allantoin_and_allantoate_transporters_n7
111: pACE2-allantoin_and_allantoate_transporters_n7 pFKH2-SFF'
111: pNDD1-metabolisof_energy_reserves_n27 pFKH2-SFF'
111: pSWI6-deoxyribonucleotide_metabolisn4 pFKH1-SFF'
111: pSWI6-deoxyribonucleotide_metabolisn4 pACE2-allantoin_and_allantoate_transporters_n7
111: pSKN7-g-proteins_n12 pAZF1-SCB
100: pAZF1-SCB
111: pSKN7-SWI5 pNDD1-metabolisof_energy_reserves_n27
010: pSKN7-SWI5
100: pSKN7-SWI5
111: pACE2-allantoin_and_allantoate_transporters_n7 pFKH1-SFF
111: pACE2-allantoin_and_allantoate_transporters_n7 pFKH1-SFF'
111: pFKH1-SFF' pSWI6-SWI5 pFKH2-SFF'
100: pFKH1-SFF' pFKH2-SFF'
110: pSWI6-SWI5 pFKH2-SFF'
100: pSWI6-SWI5
100: pSWI6-SWI5
100: pFKH1-SFF' pSWI6-SWI5
100: pSWI6-SWI5 pFKH2-SFF'
010: pFKH1-SFF' pFKH2-SFF'
110: pFKH1-SFF' pFKH2-SFF'
111: pSKN7-g-proteins_n12 pFKH2-SFF'
111: pSKN7-SWI5 pFKH2-SFF'
010: pSKN7-SWI5
100: pSKN7-SWI5
111: pFKH1-SFF' pSWI6-SWI5
100: pSWI6-SWI5
111: pSWI6-SWI5 pFKH1-SFF
100: pSWI6-SWI5
111: pMCM1-SFF' pFKH1-SFF
111: pSKN7-g-proteins_n12 pSKN7-SWI5
010: pSKN7-SWI5
100: pSKN7-SWI5
111: pRLM1-SFF' pAZF1-SCB
100: pRLM1-SFF'
100: pAZF1-SCB
010: pRLM1-SFF'
111: pMCM1-SFF' pFKH1-SFF'
111: pFKH1-SFF' pFKH2-SFF'
111: pFKH2-SFF' pFKH1-SFF
111: pFKH1-SFF' pFKH2-SFF
111: pNDD1-metabolisof_energy_reserves_n27 pAZF1-SCB
100: pAZF1-SCB
111: pSWI6-deoxyribonucleotide_metabolisn4 pFKH2-SFF
111: pSWI6-deoxyribonucleotide_metabolisn4 pFKH2-SFF'
111: pSKN7-metabolisof_energy_reserves_n27 pAZF1-SCB
100: pAZF1-SCB
111: pSKN7-metabolisof_energy_reserves_n27 pFKH2-SFF'
111: pFKH2-SFF' pAZF1-SCB
100: pAZF1-SCB

```
111: pACE2-allantoin_and_allantoate_transporters_n7 pFKH2-SFF
111: pSWI6-deoxyribonucleotide_metabolisn4 pSWI6-SWI5
 100: pSWI6-SWI5
111: pACE2-allantoin_and_allantoate_transporters_n7 pFKH1-SFF' pSWI6-SWI5
 110: pACE2-allantoin_and_allantoate_transporters_n7 pSWI6-SWI5
  100: pSWI6-SWI5
 100: pSWI6-SWI5
 100: pFKH1-SFF' pSWI6-SWI5
111: pACE2-allantoin_and_allantoate_transporters_n7 pSWI6-SWI5 pFKH1-SFF
 110: pACE2-allantoin_and_allantoate_transporters_n7 pSWI6-SWI5
  100: pSWI6-SWI5
 100: pSWI6-SWI5
 100: pSWI6-SWI5 pFKH1-SFF
111: pSKN7-SWI5 pAZF1-SCB
 010: pSKN7-SWI5
 100: pAZF1-SCB
 100: pSKN7-SWI5
111: pMCM1-SFF' pFKH1-SFF' pSWI6-SWI5
 110: pMCM1-SFF' pFKH1-SFF'
 010: pMCM1-SFF' pFKH1-SFF'
 100: pSWI6-SWI5
 100: pFKH1-SFF' pSWI6-SWI5
 100: pMCM1-SFF' pFKH1-SFF'
111: pSWI6-SWI5 pFKH2-SFF' pFKH1-SFF
 100: pFKH2-SFF' pFKH1-SFF
 110: pSWI6-SWI5 pFKH2-SFF'
  100: pSWI6-SWI5
 100: pSWI6-SWI5
 100: pSWI6-SWI5 pFKH1-SFF
 100: pSWI6-SWI5 pFKH2-SFF'
111: pMCM1-SFF' pSWI6-SWI5
 100: pSWI6-SWI5
111: pSWI6-SWI5 pFKH2-SFF
 100: pSWI6-SWI5
111: pSWI6-SWI5 pFKH2-SFF'
 100: pSWI6-SWI5
111: pMCM1-SFF' pFKH2-SFF
111: pFKH1-SFF pFKH2-SFF
111: pMCM1-SFF' pFKH2-SFF'
111: pSWI6-deoxyribonucleotide_metabolisn4 pFKH1-SFF
111: pMCM1-SFF' pFKH1-SFF' pFKH2-SFF' pFKH2-SFF
 110: pMCM1-SFF' pFKH1-SFF' pFKH2-SFF
  010: pMCM1-SFF' pFKH1-SFF'
  100: pFKH1-SFF' pFKH2-SFF
  100: pMCM1-SFF' pFKH2-SFF
  100: pMCM1-SFF' pFKH1-SFF'
 110: pMCM1-SFF' pFKH2-SFF
 110: pMCM1-SFF' pFKH1-SFF'
 110: pMCM1-SFF' pFKH2-SFF'
 110: pMCM1-SFF' pFKH1-SFF' pFKH2-SFF'
  010: pMCM1-SFF' pFKH1-SFF'
  100: pFKH1-SFF' pFKH2-SFF'
  100: pMCM1-SFF' pFKH2-SFF'
  010: pMCM1-SFF' pFKH2-SFF'
  100: pMCM1-SFF' pFKH1-SFF'
  010: pFKH1-SFF' pFKH2-SFF'
 010: pMCM1-SFF' pFKH1-SFF'
 100: pFKH1-SFF' pFKH2-SFF
 100: pFKH1-SFF' pFKH2-SFF'
 100: pMCM1-SFF' pFKH1-SFF' pFKH2-SFF
 100: pMCM1-SFF' pFKH1-SFF' pFKH2-SFF'
 100: pMCM1-SFF' pFKH2-SFF
 100: pMCM1-SFF' pFKH2-SFF'
 010: pMCM1-SFF' pFKH2-SFF'
 100: pMCM1-SFF' pFKH2-SFF' pFKH2-SFF
 100: pMCM1-SFF' pFKH1-SFF'
 100: pFKH1-SFF' pFKH2-SFF' pFKH2-SFF
 010: pFKH1-SFF' pFKH2-SFF'
 110: pFKH1-SFF' pFKH2-SFF'
111: pMCM1-SFF' pFKH2-SFF' pFKH1-SFF
```

110: pMCM1-SFF' pFKH1-SFF
110: pMCM1-SFF' pFKH2-SFF'
010: pMCM1-SFF' pFKH1-SFF
100: pFKH2-SFF' pFKH1-SFF
100: pMCM1-SFF' pFKH2-SFF'
010: pMCM1-SFF' pFKH2-SFF'
100: pMCM1-SFF' pFKH1-SFF
111: pSWI6-deoxyribonucleotide_metabolisn4 pMCM1-SFF'
111: pFKH1-SFF' pSWI6-SWI5 pFKH2-SFF
100: pFKH1-SFF' pFKH2-SFF
110: pSWI6-SWI5 pFKH2-SFF
100: pSWI6-SWI5
100: pSWI6-SWI5
100: pFKH1-SFF' pSWI6-SWI5
100: pSWI6-SWI5 pFKH2-SFF
111: pSWI6-SWI5 pFKH1-SFF pFKH2-SFF
100: pFKH1-SFF pFKH2-SFF
110: pFKH1-SFF pFKH2-SFF
110: pSWI6-SWI5 pFKH2-SFF
100: pSWI6-SWI5
100: pSWI6-SWI5
100: pSWI6-SWI5 pFKH1-SFF
100: pSWI6-SWI5 pFKH2-SFF
111: pFKH2-SFF' pFKH1-SFF pFKH2-SFF
100: pFKH2-SFF' pFKH1-SFF
100: pFKH1-SFF pFKH2-SFF
110: pFKH1-SFF pFKH2-SFF
111: pMCM1-SFF' pFKH1-SFF' pFKH2-SFF
110: pMCM1-SFF' pFKH2-SFF
110: pMCM1-SFF' pFKH1-SFF'
010: pMCM1-SFF' pFKH1-SFF'
100: pFKH1-SFF' pFKH2-SFF
100: pMCM1-SFF' pFKH2-SFF
100: pMCM1-SFF' pFKH1-SFF'
111: pFKH2-deoxyribonucleotide_metabolisn4 pSWI6-SWI5
100: pSWI6-SWI5
111: pSKN7-SWI5 pSKN7-metabolisof_energy_reserves_n27 pNDD1-metabolisof_energy_reserves_n27
010: pSKN7-SWI5
100: pSKN7-SWI5
111: pMCM1-SFF' pFKH2-deoxyribonucleotide_metabolisn4
111: pSTB1-MCB pSWI6-deoxyribonucleotide_metabolisn10
111: pFKH2-deoxyribonucleotide_metabolisn4 pFKH1-SFF
111: pFKH2-deoxyribonucleotide_metabolisn4 pFKH2-SFF
111: pFKH2-deoxyribonucleotide_metabolisn4 pFKH2-SFF'
111: pSWI6-MCB pSWI6-deoxyribonucleotide_metabolisn10
111: pFKH2-deoxyribonucleotide_metabolisn4 pFKH1-SFF'
111: pSWI6-deoxyribonucleotide_metabolisn4 pFKH2-deoxyribonucleotide_metabolisn4
111: pACE2-allantoin_and_allantoate_transporters_n7 pSWI6-SWI5
100: pSWI6-SWI5
111: pSWI4-SCB pSWI6-deoxyribonucleotide_metabolisn10
010: pSWI4-SCB
100: pSWI4-SCB
111: pSWI6-deoxyribonucleotide_metabolisn4 pFKH1-SFF' pSWI6-SWI5
100: pSWI6-SWI5
100: pFKH1-SFF' pSWI6-SWI5
111: pSWI6-deoxyribonucleotide_metabolisn4 pACE2-allantoin_and_allantoate_transporters_n7 pFKH1-SFF
111: pYAP6-phosphate_transport_n18 pSKN7-LFTE17
010: pYAP6-phosphate_transport_n18
100: pSKN7-LFTE17
111: pSKN7-ion_transporters_n11 pAZF1-SCB
100: pAZF1-SCB
111: pSKN7-ion_transporters_n11 pSKN7-LFTE17
100: pSKN7-LFTE17
111: pSKN7-LFTE17 pAZF1-SCB
100: pAZF1-SCB
100: pSKN7-LFTE17
111: pYAP6-phosphate_transport_n18 pAZF1-SCB
100: pAZF1-SCB
010: pYAP6-phosphate_transport_n18
111: pSKN7-SWI5 pSKN7-metabolisof_energy_reserves_n27

010: pSKN7-SWI5
100: pSKN7-SWI5
111: pYAP6-phosphate_transport_n18 pSKN7-ion_transporters_n11
010: pYAP6-phosphate_transport_n18
111: pSKN7-g-proteins_n12 pSKN7-SWI5 pAZF1-SCB
010: pSKN7-SWI5
010: pSKN7-g-proteins_n12 pAZF1-SCB
110: pSKN7-g-proteins_n12 pSKN7-SWI5
 010: pSKN7-SWI5
 100: pSKN7-SWI5
100: pAZF1-SCB
100: pSKN7-SWI5
010: pSKN7-SWI5 pAZF1-SCB
010: pSKN7-g-proteins_n12 pSKN7-SWI5
111: pSWI6-deoxyribonucleotide_metabolisn10 pSWI6-nutritional_response_pathway_n7
010: pSWI6-nutritional_response_pathway_n7
111: pSWI4-nutritional_response_pathway_n7 pSWI4-SCB pSWI4-ion_transporters_n11
010: pSWI4-SCB
100: pSWI4-ion_transporters_n11
100: pSWI4-SCB
111: pSWI4-SCB pRLM1-SFF'
100: pRLM1-SFF'
010: pSWI4-SCB
100: pSWI4-SCB
010: pRLM1-SFF'
111: pSWI6-deoxyribonucleotide_metabolisn10 pRLM1-SFF'
100: pRLM1-SFF'
010: pRLM1-SFF'
111: pSWI4-SCB pSWI4-ion_transporters_n11
010: pSWI4-SCB
100: pSWI4-ion_transporters_n11
100: pSWI4-SCB
111: pSWI4-nutritional_response_pathway_n7 pSWI4-ion_transporters_n11
100: pSWI4-ion_transporters_n11
111: pSWI4-amino-acid_transporters_n11 pSWI4-SCB
010: pSWI4-amino-acid_transporters_n11
010: pSWI4-SCB
100: pSWI4-SCB
111: pSWI4-amino-acid_transporters_n11 pSKN7-amino-acid_transporters_n11
010: pSWI4-amino-acid_transporters_n11
010: pSKN7-amino-acid_transporters_n11
111: pSWI4-MCM1' pSWI6-deoxyribonucleotide_metabolisn10
100: pSWI4-MCM1'
010: pSWI4-MCM1'
111: pSWI4-MCM1' pSWI4-SCB pSWI6-mPROTEOL18(proteolysis_n18)
100: pSWI4-MCM1'
010: pSWI4-MCM1' pSWI4-SCB
010: pSWI4-SCB pSWI6-mPROTEOL18(proteolysis_n18)
010: pSWI4-SCB
100: pSWI6-mPROTEOL18(proteolysis_n18)
100: pSWI4-MCM1' pSWI4-SCB
110: pSWI4-MCM1' pSWI6-mPROTEOL18(proteolysis_n18)
 100: pSWI4-MCM1'
 100: pSWI6-mPROTEOL18(proteolysis_n18)
 010: pSWI6-mPROTEOL18(proteolysis_n18)
 010: pSWI4-MCM1'
010: pSWI4-MCM1' pSWI6-mPROTEOL18(proteolysis_n18)
010: pSWI6-mPROTEOL18(proteolysis_n18)
010: pSWI4-MCM1'
110: pSWI4-MCM1' pSWI4-SCB
 100: pSWI4-MCM1'
 010: pSWI4-SCB
 010: pSWI4-MCM1'
 100: pSWI4-SCB
100: pSWI4-SCB
111: pSWI4-nutritional_response_pathway_n7 pSWI4-SCB
010: pSWI4-SCB
100: pSWI4-SCB
111: pSWI4-MCM1' pSWI4-amino-acid_transporters_n11
100: pSWI4-MCM1'

010: pSWI4-amino-acid_transporters_n11
010: pSWI4-MCM1'
111: pSWI4-nutritional_response_pathway_n7 pSWI6-deoxyribonucleotide_metabolisn10
111: pSWI4-MCM1' pSWI4-amino-acid_transporters_n11 pSWI4-SCB
100: pSWI4-MCM1'
010: pSWI4-amino-acid_transporters_n11 pSWI4-SCB
010: pSWI4-amino-acid_transporters_n11
010: pSWI4-MCM1' pSWI4-SCB
010: pSWI4-SCB
100: pSWI4-MCM1' pSWI4-SCB
010: pSWI4-MCM1'
010: pSWI4-MCM1' pSWI4-amino-acid_transporters_n11
110: pSWI4-MCM1' pSWI4-SCB
100: pSWI4-MCM1'
010: pSWI4-SCB
010: pSWI4-MCM1'
100: pSWI4-SCB
100: pSWI4-SCB
111: pSWI6-ion_transporters_n11 pSWI4-SCB pSWI6-nutritional_response_pathway_n7
010: pSWI6-nutritional_response_pathway_n7
010: pSWI4-SCB
100: pSWI4-SCB
111: pSWI4-SCB pSWI6-nutritional_response_pathway_n7 pSWI4-ion_transporters_n11
010: pSWI6-nutritional_response_pathway_n7
010: pSWI4-SCB
100: pSWI4-ion_transporters_n11
100: pSWI4-SCB
111: pSWI4-nutritional_response_pathway_n7 pSWI4-glycolysis_and_gluconeogenesis_n27
111: pSWI4-MCM1' pSWI6-deoxyribonucleotide_metabolisn10 pSWI6-nutritional_response_pathway_n7
100: pSWI4-MCM1'
010: pSWI6-nutritional_response_pathway_n7
110: pSWI4-MCM1' pSWI6-nutritional_response_pathway_n7
100: pSWI4-MCM1'
010: pSWI6-nutritional_response_pathway_n7
010: pSWI4-MCM1'
010: pSWI4-MCM1'
111: pSWI4-nutritional_response_pathway_n7 pSWI6-nutritional_response_pathway_n7
010: pSWI6-nutritional_response_pathway_n7
110: pUPC2-ALPHA1' pMCM1-ECB
100: pMCM1-ECB
010: pMCM1-ECB
110: pNDD1-MCM1 pDIG1-ECB
100: pNDD1-MCM1
110: pNDD1-ECB pINO4-osmosensing_n6
010: pINO4-osmosensing_n6
100: pINO4-osmosensing_n6
110: pYOX1-MCM1 pDIG1-ECB
010: pYOX1-MCM1
110: pFKH2-ECB pINO4-osmosensing_n6
010: pINO4-osmosensing_n6
100: pINO4-osmosensing_n6
110: pYDR049W-ndt80(MSE) pFKH2-SFF'
110: pYER130C-ndt80(MSE) pFKH1-SFF
110: pYDR049W-ndt80(MSE) pFKH1-SFF
110: pYER130C-ndt80(MSE) pFKH2-SFF'
110: pINO4-g-proteins_n12 pFKH2-SFF
110: pMCM1-MCM1' pINO4-osmosensing_n6
100: pMCM1-MCM1'
010: pINO4-osmosensing_n6
100: pINO4-osmosensing_n6
110: pDIG1-ECB pNDD1-ECB
110: pMCM1-SFF' pINO4-osmosensing_n6
010: pINO4-osmosensing_n6
100: pINO4-osmosensing_n6
110: pYDR049W-ndt80(MSE) pFKH2-SFF
110: pFKH2-SFF' pROX1-osmosensing_n6
100: pROX1-osmosensing_n6
010: pROX1-osmosensing_n6
110: pNDD1-MCM1' pINO4-osmosensing_n6
100: pNDD1-MCM1'

010: pINO4-osmosensing_n6
100: pINO4-osmosensing_n6
110: pFKH1-SFF' pINO4-g-proteins_n12
110: pFKH1-SFF pINO4-g-proteins_n12
110: pFKH1-SFF pINO4-osmosensing_n6
010: pINO4-osmosensing_n6
100: pINO4-osmosensing_n6
110: pFKH2-ECB pDIG1-ECB
110: pINO4-osmosensing_n6 pFKH2-SFF
010: pINO4-osmosensing_n6
100: pINO4-osmosensing_n6
110: pDIG1-ECB pFKH2-MCM1
100: pFKH2-MCM1
010: pFKH2-MCM1
110: pROX1-osmosensing_n6 pINO4-osmosensing_n6
010: pINO4-osmosensing_n6
100: pROX1-osmosensing_n6
010: pROX1-osmosensing_n6
100: pINO4-osmosensing_n6
110: pFKH1-SFF' pINO4-osmosensing_n6
010: pINO4-osmosensing_n6
100: pINO4-osmosensing_n6
110: pYER130C-ndt80(MSE) pFKH2-SFF
110: pDIG1-ECB pFKH2-SFF
110: pMCM1-MCM1' pROX1-osmosensing_n6
100: pMCM1-MCM1'
100: pROX1-osmosensing_n6
010: pROX1-osmosensing_n6
110: pFKH1-SFF pINO4-SWI5
110: pFKH2-SFF' pINO4-osmosensing_n6
010: pINO4-osmosensing_n6
100: pINO4-osmosensing_n6
110: pMCM1-SFF' pROX1-osmosensing_n6
100: pROX1-osmosensing_n6
010: pROX1-osmosensing_n6
110: pFKH2-SFF' pINO4-SWI5
110: pFKH1-SFF' pINO4-SWI5
110: pINO4-SWI5 pFKH2-SFF
110: pFKH2-SFF' pINO4-g-proteins_n12
110: pUPC2-ALPHA1' pFKH1-SFF pFKH2-SFF
010: pUPC2-ALPHA1' pFKH1-SFF
100: pFKH1-SFF pFKH2-SFF
110: pYDR049W-ndt80(MSE) pFKH1-SFF'
110: pYER130C-ndt80(MSE) pFKH1-SFF'
110: pINO4-osmosensing_n6 pINO4-SWI5
010: pINO4-osmosensing_n6
100: pINO4-osmosensing_n6
110: pUPC2-ALPHA1' pFKH2-SFF
110: pMCM1-MCM1' pUPC2-ALPHA1'
100: pMCM1-MCM1'
110: pINO4-osmosensing_n6 pINO4-g-proteins_n12
010: pINO4-osmosensing_n6
100: pINO4-osmosensing_n6
110: pUPC2-ALPHA1' pFKH2-SFF'
110: pNDD1-MCM1' pINO4-g-proteins_n12
100: pNDD1-MCM1'
110: pNDD1-MCM1' pINO4-SWI5
100: pNDD1-MCM1'
110: pMCM1-MCM1' pNDD1-ECB
100: pMCM1-MCM1'
110: pYOX1-ECB pFKH2-SFF'
010: pYOX1-ECB
110: pFKH2-ECB pYOX1-MCM1
010: pYOX1-MCM1
110: pMCM1-MCM1' pFKH2-ECB
100: pMCM1-MCM1'
110: pMCM1-ECB pFKH2-SFF'
100: pMCM1-ECB
010: pMCM1-ECB
110: pNDD1-MCM1' pFKH2-ECB

100: pNDD1-MCM1'
110: pYOX1-MCM1 pMCM1-ECB
100: pMCM1-ECB
010: pMCM1-ECB
010: pYOX1-MCM1
110: pFKH1-SFF' pNDD1-ECB
110: pMCM1-MCM1 pNDD1-ECB
100: pMCM1-MCM1
010: pMCM1-MCM1
110: pNDD1-ECB pFKH2-SFF'
110: pNDD1-MCM1 pMCM1-ECB
100: pMCM1-ECB
010: pMCM1-ECB
100: pNDD1-MCM1
110: pYOX1-ECB pMCM1-SFF'
010: pYOX1-ECB
110: pMCM1-SFF' pNDD1-ECB
110: pYOX1-ECB pMCM1-ECB
100: pMCM1-ECB
010: pMCM1-ECB
010: pYOX1-ECB
110: pFKH2-ECB pMCM1-ECB
100: pMCM1-ECB
010: pMCM1-ECB
110: pYOX1-ECB pMCM1-MCM1
100: pMCM1-MCM1
010: pMCM1-MCM1
010: pYOX1-ECB
110: pYOX1-ECB pMCM1-MCM1'
100: pMCM1-MCM1'
010: pYOX1-ECB
110: pMCM1-MCM1' pYOX1-MCM1
100: pMCM1-MCM1'
010: pYOX1-MCM1
110: pYOX1-MCM1 pFKH2-SFF
010: pYOX1-MCM1
110: pNDD1-MCM1 pFKH2-ECB
100: pNDD1-MCM1
110: pMCM1-ECB pFKH2-SFF
100: pMCM1-ECB
010: pMCM1-ECB
110: pMCM1-ECB pFKH1-SFF'
100: pMCM1-ECB
010: pMCM1-ECB
110: pMCM1-SFF' pYOX1-MCM1
010: pYOX1-MCM1
110: pMCM1-ECB pNDD1-ECB
100: pMCM1-ECB
010: pMCM1-ECB
110: pYOX1-ECB pFKH1-SFF'
010: pYOX1-ECB
110: pYOX1-ECB pNDD1-MCM1'
100: pNDD1-MCM1'
010: pYOX1-ECB
110: pNDD1-MCM1' pMCM1-ECB
100: pNDD1-MCM1'
100: pMCM1-ECB
010: pMCM1-ECB
110: pNDD1-ECB pFKH2-SFF
110: pMCM1-SFF' pFKH2-ECB
110: pYOX1-ECB pFKH1-SFF
010: pYOX1-ECB
110: pMCM1-SFF' pUPC2-ALPHA1'
110: pYOX1-ECB pFKH2-MCM1
100: pFKH2-MCM1
010: pFKH2-MCM1
010: pYOX1-ECB
110: pYOX1-MCM1 pFKH2-SFF'
010: pYOX1-MCM1
110: pNDD1-ECB pFKH1-SFF

110: pYOX1-MCM1 pFKH1-SFF
010: pYOX1-MCM1
110: pMCM1-ECB pFKH1-SFF
100: pMCM1-ECB
010: pMCM1-ECB
110: pYOX1-ECB pNDD1-ECB
010: pYOX1-ECB
110: pMCM1-ECB pFKH2-MCM1
100: pFKH2-MCM1
100: pMCM1-ECB
010: pMCM1-ECB
010: pFKH2-MCM1
110: pNDD1-MCM1 pNDD1-ECB
100: pNDD1-MCM1
110: pMCM1-MCM1 pFKH2-ECB
100: pMCM1-MCM1
010: pMCM1-MCM1
110: pYOX1-ECB pNDD1-MCM1
100: pNDD1-MCM1
010: pYOX1-ECB
110: pMCM1-MCM1 pYOX1-MCM1
100: pMCM1-MCM1
010: pMCM1-MCM1
010: pYOX1-MCM1
110: pNDD1-ECB pFKH2-MCM1
100: pFKH2-MCM1
010: pFKH2-MCM1
110: pYOX1-ECB pFKH2-ECB
010: pYOX1-ECB
110: pNDD1-MCM1 pYOX1-MCM1
100: pNDD1-MCM1
010: pYOX1-MCM1
110: pYOX1-ECB pFKH2-SFF
010: pYOX1-ECB
110: pYOX1-MCM1 pFKH1-SFF'
010: pYOX1-MCM1
110: pNDD1-MCM1' pYOX1-MCM1
100: pNDD1-MCM1'
010: pYOX1-MCM1
110: pYOX1-MCM1 pFKH2-MCM1
100: pFKH2-MCM1
010: pFKH2-MCM1
010: pYOX1-MCM1
110: pFKH2-ECB pNDD1-ECB
110: pYOX1-MCM1 pNDD1-ECB
010: pYOX1-MCM1
110: pMCM1-SFF' pFKH2-SFF' pFKH1-SFF
010: pMCM1-SFF' pFKH1-SFF
100: pFKH2-SFF' pFKH1-SFF
100: pMCM1-SFF' pFKH2-SFF'
010: pMCM1-SFF' pFKH2-SFF'
100: pMCM1-SFF' pFKH1-SFF
110: pMCM1-SFF' pFKH1-SFF pFKH2-SFF
010: pMCM1-SFF' pFKH1-SFF
100: pFKH1-SFF pFKH2-SFF
100: pMCM1-SFF' pFKH2-SFF
100: pMCM1-SFF' pFKH1-SFF
110: pMCM1-MCM1 pFKH1-SFF'
100: pMCM1-MCM1
010: pMCM1-MCM1
110: pMCM1-MCM1' pFKH1-SFF'
100: pMCM1-MCM1'
110: pNDD1-MCM1' pFKH2-SFF'
100: pNDD1-MCM1'
110: pMCM1-MCM1' pFKH2-SFF
100: pMCM1-MCM1'
110: pNDD1-MCM1' pFKH2-SFF
100: pNDD1-MCM1'
110: pNDD1-MCM1' pFKH2-MCM1
100: pFKH2-MCM1

100: pNDD1-MCM1'
010: pFKH2-MCM1
110: pNDD1-MCM1' pMCM1-SFF'
100: pNDD1-MCM1'
110: pMCM1-MCM1' pNDD1-MCM1'
100: pNDD1-MCM1'
100: pMCM1-MCM1'
110: pMCM1-MCM1' pFKH2-SFF'
100: pMCM1-MCM1'
110: pNDD1-MCM1' pFKH1-SFF'
100: pNDD1-MCM1'
110: pMCM1-MCM1 pFKH2-SFF'
100: pMCM1-MCM1
010: pMCM1-MCM1
110: pNDD1-MCM1' pMCM1-MCM1
100: pNDD1-MCM1'
100: pMCM1-MCM1
010: pMCM1-MCM1
110: pMCM1-MCM1 pFKH2-MCM1
100: pFKH2-MCM1
100: pMCM1-MCM1
010: pMCM1-MCM1
010: pFKH2-MCM1
110: pMCM1-MCM1 pFKH2-SFF
100: pMCM1-MCM1
010: pMCM1-MCM1
110: pMCM1-MCM1 pFKH1-SFF
100: pMCM1-MCM1
010: pMCM1-MCM1
110: pMCM1-MCM1' pFKH1-SFF
100: pMCM1-MCM1'
110: pNDD1-MCM1' pFKH1-SFF
100: pNDD1-MCM1'
110: pMCM1-MCM1' pFKH2-MCM1
100: pFKH2-MCM1
100: pMCM1-MCM1'
010: pFKH2-MCM1
110: pMCM1-SFF' pFKH2-MCM1
100: pFKH2-MCM1
010: pFKH2-MCM1
110: pNDD1-MCM1' pUPC2-ALPHA1'
100: pNDD1-MCM1'
110: pNDD1-MCM1' pDIG1-ECB
100: pNDD1-MCM1'
110: pDIG1-ECB pINO4-g-proteins_n12
110: pNDD1-MCM1' pROX1-osmosensing_n6
100: pNDD1-MCM1'
100: pROX1-osmosensing_n6
010: pROX1-osmosensing_n6
110: pYOX1-ECB pDIG1-ECB
010: pYOX1-ECB
110: pMCM1-ECB pROX1-osmosensing_n6
100: pMCM1-ECB
100: pROX1-osmosensing_n6
010: pMCM1-ECB
010: pROX1-osmosensing_n6
110: pNDD1-ECB pROX1-osmosensing_n6
100: pROX1-osmosensing_n6
010: pROX1-osmosensing_n6
110: pUPC2-ALPHA1' pNDD1-ECB
110: pFKH2-ECB pROX1-osmosensing_n6
100: pROX1-osmosensing_n6
010: pROX1-osmosensing_n6
110: pNDD1-MCM1' pFKH2-SFF' pINO4-SWI5
100: pFKH2-SFF' pINO4-SWI5
100: pNDD1-MCM1' pFKH2-SFF'
100: pNDD1-MCM1' pINO4-SWI5
100: pNDD1-MCM1'
110: pFKH2-ECB pUPC2-ALPHA1' pFKH1-SFF' pFKH2-SFF
010: pUPC2-ALPHA1' pFKH1-SFF'

100: pFKH1-SFF' pFKH2-SFF
010: pFKH2-ECB pFKH1-SFF'
110: pFKH2-ECB pUPC2-ALPHA1'
110: pUPC2-ALPHA1' pINO4-osmosensing_n6
010: pINO4-osmosensing_n6
100: pINO4-osmosensing_n6
110: pDIG1-ECB pINO4-osmosensing_n6
010: pINO4-osmosensing_n6
100: pINO4-osmosensing_n6
110: pFKH2-ECB pDIG1-ECB pMCM1-ECB
100: pMCM1-ECB
010: pMCM1-ECB
110: pYER130C-ndt80(MSE) pFKH1-SFF' pFKH2-SFF'
100: pFKH1-SFF' pFKH2-SFF'
010: pFKH1-SFF' pFKH2-SFF'
110: pFKH2-ECB pDIG1-ECB pFKH2-MCM1
100: pFKH2-MCM1
010: pFKH2-MCM1
110: pUPC2-ALPHA1' pFKH1-SFF' pFKH2-SFF'
010: pUPC2-ALPHA1' pFKH1-SFF'
100: pFKH1-SFF' pFKH2-SFF'
010: pFKH1-SFF' pFKH2-SFF'
110: pMCM1-SFF' pYOX1-MCM1 pFKH1-SFF'
010: pMCM1-SFF' pFKH1-SFF'
100: pMCM1-SFF' pFKH1-SFF'
010: pYOX1-MCM1
110: pDIG1-ECB pFKH1-SFF'
110: pNDD1-MCM1' pFKH1-SFF' pINO4-SWI5
100: pFKH1-SFF' pINO4-SWI5
100: pNDD1-MCM1' pFKH1-SFF'
100: pNDD1-MCM1' pINO4-SWI5
100: pNDD1-MCM1'
010: pNDD1-MCM1' pFKH1-SFF'
110: pFKH2-ECB pUPC2-ALPHA1' pFKH1-SFF
010: pUPC2-ALPHA1' pFKH1-SFF
110: pFKH2-ECB pUPC2-ALPHA1' pFKH1-SFF'
010: pUPC2-ALPHA1' pFKH1-SFF'
010: pFKH2-ECB pFKH1-SFF'
110: pMCM1-ECB pINO4-osmosensing_n6
100: pMCM1-ECB
010: pINO4-osmosensing_n6
010: pMCM1-ECB
100: pINO4-osmosensing_n6
110: pROX1-osmosensing_n6 pFKH2-SFF
100: pROX1-osmosensing_n6
010: pROX1-osmosensing_n6
110: pUPC2-ALPHA1' pFKH2-SFF' pFKH1-SFF
010: pUPC2-ALPHA1' pFKH1-SFF
100: pFKH2-SFF' pFKH1-SFF
110: pMCM1-SFF' pUPC2-ALPHA1' pFKH1-SFF'
010: pMCM1-SFF' pFKH1-SFF'
010: pUPC2-ALPHA1' pFKH1-SFF'
100: pMCM1-SFF' pFKH1-SFF'
110: pFKH2-SFF' pINO4-g-proteins_n12 pINO4-SWI5
100: pFKH2-SFF' pINO4-SWI5
100: pINO4-g-proteins_n12 pINO4-SWI5
010: pINO4-g-proteins_n12 pINO4-SWI5
001: pINO4-g-proteins_n12 pINO4-SWI5
110: pUPC2-ALPHA1' pFKH1-SFF' pFKH2-SFF
010: pUPC2-ALPHA1' pFKH1-SFF'
100: pFKH1-SFF' pFKH2-SFF
110: pFKH2-ECB pUPC2-ALPHA1' pFKH1-SFF pFKH2-SFF
010: pUPC2-ALPHA1' pFKH1-SFF
100: pFKH1-SFF pFKH2-SFF
110: pDIG1-ECB pFKH1-SFF
110: pNDD1-MCM1' pFKH1-SFF pINO4-SWI5
100: pFKH1-SFF pINO4-SWI5
100: pNDD1-MCM1' pFKH1-SFF
100: pNDD1-MCM1' pINO4-SWI5
100: pNDD1-MCM1'

110: pMCM1-MCM1' pUPC2-ALPHA1' pROX1-osmosensing_n6
100: pMCM1-MCM1'
100: pROX1-osmosensing_n6
010: pROX1-osmosensing_n6
110: pNDD1-MCM1' pINO4-g-proteins_n12 pINO4-SWI5
100: pNDD1-MCM1' pINO4-SWI5
100: pINO4-g-proteins_n12 pINO4-SWI5
100: pNDD1-MCM1'
010: pINO4-g-proteins_n12 pINO4-SWI5
001: pINO4-g-proteins_n12 pINO4-SWI5
110: pNDD1-MCM1' pMCM1-SFF' pFKH1-SFF
010: pMCM1-SFF' pFKH1-SFF
100: pNDD1-MCM1' pFKH1-SFF
100: pNDD1-MCM1' pMCM1-SFF'
100: pNDD1-MCM1'
100: pMCM1-SFF' pFKH1-SFF
110: pFKH2-ECB pUPC2-ALPHA1' pFKH2-SFF' pFKH1-SFF
010: pUPC2-ALPHA1' pFKH1-SFF
100: pFKH2-SFF' pFKH1-SFF
110: pNDD1-MCM1' pFKH2-SFF' pINO4-g-proteins_n12
100: pNDD1-MCM1' pFKH2-SFF'
100: pNDD1-MCM1'
110: pUPC2-ALPHA1' pFKH1-SFF' pFKH2-SFF' pFKH1-SFF
010: pUPC2-ALPHA1' pFKH1-SFF
010: pUPC2-ALPHA1' pFKH1-SFF'
100: pFKH2-SFF' pFKH1-SFF
100: pFKH1-SFF' pFKH2-SFF'
100: pFKH1-SFF' pFKH2-SFF' pFKH1-SFF
010: pFKH1-SFF' pFKH2-SFF'
110: pRPI1-SFF pGZF3-SFF
010: pGZF3-SFF
010: pRPI1-SFF
100: pRPI1-SFF
100: pGZF3-SFF
001: pRPI1-SFF
110: pSWI4-MCM1' pSWI4-drug_transporters_n9 pSWI4-MCM1
100: pSWI4-MCM1'
100: pSWI4-MCM1' pSWI4-MCM1
100: pSWI4-MCM1
010: pSWI4-MCM1'
110: pSWI4-drug_transporters_n9 pSWI4-MCM1
100: pSWI4-MCM1
110: pSWI4-MCM1' pYOX1-MCM1
100: pSWI4-MCM1'
010: pSWI4-MCM1'
010: pYOX1-MCM1
110: pMCM1-MCM1' pSWI4-MCM1'
100: pSWI4-MCM1'
100: pMCM1-MCM1'
010: pSWI4-MCM1'
110: pSWI4-MCM1' pMCM1-MCM1
100: pSWI4-MCM1'
100: pMCM1-MCM1
010: pMCM1-MCM1
010: pSWI4-MCM1'
110: pSWI4-MCM1' pMCM1-SFF'
100: pSWI4-MCM1'
010: pSWI4-MCM1'
110: pYOX1-ECB pSWI4-MCM1'
100: pSWI4-MCM1'
010: pSWI4-MCM1'
010: pYOX1-ECB
110: pSWI4-MCM1' pMCM1-ECB
100: pSWI4-MCM1'
100: pMCM1-ECB
010: pMCM1-ECB
010: pSWI4-MCM1'
110: pMCM1-other_morphogenetic_activities_n7 pYOX1-MCM1
100: pMCM1-other_morphogenetic_activities_n7
010: pYOX1-MCM1

110: pMCM1-other_morphogenetic_activities_n7 pMCM1-ECB
100: pMCM1-other_morphogenetic_activities_n7
100: pMCM1-ECB
010: pMCM1-ECB
110: pMCM1-other_morphogenetic_activities_n7 pMCM1-MCM1
100: pMCM1-other_morphogenetic_activities_n7
100: pMCM1-MCM1
010: pMCM1-MCM1
110: pYOX1-ECB pMCM1-other_morphogenetic_activities_n7
100: pMCM1-other_morphogenetic_activities_n7
010: pYOX1-ECB
110: pMBP1-mPROTEOL18(proteolysis_n18) pSWI6-allantoin_and_allantoate_transporters_n13
010: pMBP1-mPROTEOL18(proteolysis_n18)
110: pMCM1-MCM1 pSWI6-LYS14
100: pMCM1-MCM1
010: pMCM1-MCM1
010: pSWI6-LYS14
110: pMCM1-MCM1 pUME6-SCB
100: pMCM1-MCM1
010: pMCM1-MCM1
010: pUME6-SCB
110: pMCM1-SFF' pSWI6-LYS14
010: pSWI6-LYS14
110: pMBP1-glycolysis_and_gluconeogenesis_n11 pSWI6-allantoin_and_allantoate_transporters_n13
110: pSWI6-LYS14 pSWI4-SCB
010: pSWI4-SCB
010: pSWI6-LYS14
100: pSWI4-SCB
110: pMCM1-SFF' pUME6-SCB
010: pUME6-SCB
110: pSWI6-mPROTEOL18(proteolysis_n18) pSWI6-allantoin_and_allantoate_transporters_n13
100: pSWI6-mPROTEOL18(proteolysis_n18)
010: pSWI6-mPROTEOL18(proteolysis_n18)
110: pSWI4-MCM1 pSWI6-LYS14
100: pSWI4-MCM1
010: pSWI6-LYS14
110: pMBP1-mPROTEOL18(proteolysis_n18) pSWI6-deoxyribonucleotide_metabolisn27
010: pMBP1-mPROTEOL18(proteolysis_n18)
110: pSWI6-STRE pSWI6-deoxyribonucleotide_metabolisn27
100: pSWI6-STRE
110: pUME6-SCB pSWI6-LYS14
010: pSWI6-LYS14
010: pUME6-SCB
110: pSWI4-MCM1 pSWI6-mPROTEOL18(proteolysis_n18)
100: pSWI6-mPROTEOL18(proteolysis_n18)
100: pSWI4-MCM1
010: pSWI6-mPROTEOL18(proteolysis_n18)
110: pSWI4-MCM1 pRLM1-SFF'
100: pRLM1-SFF'
100: pSWI4-MCM1
010: pRLM1-SFF'
110: pSWI6-LYS14 pRLM1-SFF'
100: pRLM1-SFF'
010: pSWI6-LYS14
010: pRLM1-SFF'
110: pMCM1-ECB pRLM1-SFF'
100: pRLM1-SFF'
100: pMCM1-ECB
010: pMCM1-ECB
010: pRLM1-SFF'
110: pMCM1-MCM1 pSWI4-SCB
010: pSWI4-SCB
100: pMCM1-MCM1
010: pMCM1-MCM1
100: pSWI4-SCB
110: pUME6-SCB pSWI4-MCM1
100: pSWI4-MCM1
010: pUME6-SCB
110: pSWI6-deoxyribonucleotide_metabolisn10 pSWI6-allantoin_and_allantoate_transporters_n13
110: pSWI4-SCB pSWI6-allantoin_and_allantoate_transporters_n13

010: pSWI4-SCB
100: pSWI4-SCB
110: pSWI4-MCM1 pMBP1-mPROTEOL18(proteolysis_n18)
100: pSWI4-MCM1
010: pMBP1-mPROTEOL18(proteolysis_n18)
110: pMCM1-SFF' pRLM1-SFF'
100: pRLM1-SFF'
010: pRLM1-SFF'
110: pMCM1-MCM1' pRLM1-SFF'
100: pRLM1-SFF'
100: pMCM1-MCM1'
010: pRLM1-SFF'
110: pSWI4-MCM1 pSWI4-SCB
010: pSWI4-SCB
100: pSWI4-MCM1
100: pSWI4-SCB
110: pSWI4-MCM1' pSWI6-LYS14
100: pSWI4-MCM1'
010: pSWI6-LYS14
010: pSWI4-MCM1'
110: pSWI6-LYS14 pSWI6-mPROTEOL18(proteolysis_n18)
100: pSWI6-mPROTEOL18(proteolysis_n18)
010: pSWI6-LYS14
010: pSWI6-mPROTEOL18(proteolysis_n18)
110: pSWI6-LYS14 pMBP1-mPROTEOL18(proteolysis_n18)
010: pMBP1-mPROTEOL18(proteolysis_n18)
010: pSWI6-LYS14
110: pSWI6-LYS14 pSWI6-deoxyribonucleotide_metabolisn10
010: pSWI6-LYS14
110: pMCM1-SFF' pSWI4-SCB
010: pSWI4-SCB
100: pSWI4-SCB
110: pMCM1-MCM1 pSWI4-MCM1
100: pSWI4-MCM1
100: pMCM1-MCM1
010: pMCM1-MCM1
110: pMCM1-SFF' pMCM1-ECB
100: pMCM1-ECB
010: pMCM1-ECB
110: pMCM1-SFF' pMCM1-MCM1
100: pMCM1-MCM1
010: pMCM1-MCM1
110: pMBP1-mPROTEOL18(proteolysis_n18) pRLM1-SFF'
100: pRLM1-SFF'
010: pMBP1-mPROTEOL18(proteolysis_n18)
010: pRLM1-SFF'
110: pMCM1-MCM1' pSWI4-MCM1
100: pSWI4-MCM1
100: pMCM1-MCM1'
110: pMCM1-SFF' pSWI4-MCM1
100: pSWI4-MCM1
110: pSWI4-MCM1 pMCM1-ECB
100: pMCM1-ECB
100: pSWI4-MCM1
010: pMCM1-ECB
110: pSWI6-mPROTEOL18(proteolysis_n18) pRLM1-SFF'
100: pRLM1-SFF'
100: pSWI6-mPROTEOL18(proteolysis_n18)
010: pSWI6-mPROTEOL18(proteolysis_n18)
010: pRLM1-SFF'
110: pSWI4-SCB pRLM1-SFF'
100: pRLM1-SFF'
010: pSWI4-SCB
100: pSWI4-SCB
010: pRLM1-SFF'
110: pMCM1-MCM1' pMCM1-SFF'
100: pMCM1-MCM1'
110: pSWI4-MCM1' pSWI4-MCM1
100: pSWI4-MCM1'
100: pSWI4-MCM1

010: pSWI4-MCM1'
110: pSWI4-MCM1' pRLM1-SFF'
100: pRLM1-SFF'
100: pSWI4-MCM1'
010: pSWI4-MCM1'
010: pRLM1-SFF'
110: pSWI4-MCM1' pMBP1-mPROTEOL18(proteolysis_n18)
100: pSWI4-MCM1'
010: pMBP1-mPROTEOL18(proteolysis_n18)
010: pSWI4-MCM1'
110: pMCM1-MCM1' pUME6-SCB
100: pMCM1-MCM1'
010: pUME6-SCB
110: pUME6-SCB pMCM1-ECB
100: pMCM1-ECB
010: pMCM1-ECB
010: pUME6-SCB
110: pMCM1-MCM1' pSWI4-MCM1' pSWI4-MCM1
100: pSWI4-MCM1'
100: pMCM1-MCM1' pSWI4-MCM1
100: pSWI4-MCM1' pSWI4-MCM1
100: pMCM1-MCM1' pSWI4-MCM1'
100: pSWI4-MCM1
100: pMCM1-MCM1'
010: pSWI4-MCM1'
110: pNDD1-MCM1 pSWI6-LYS14
010: pSWI6-LYS14
100: pNDD1-MCM1
110: pSWI6-other_proteolytic_degradation_n7 pFKH2-SFF'
110: pSWI6-LYS14 pFKH2-MCM1
100: pFKH2-MCM1
010: pSWI6-LYS14
010: pFKH2-MCM1
110: pSWI6-other_proteolytic_degradation_n7 pSWI4-SCB
010: pSWI4-SCB
100: pSWI4-SCB
110: pMCM1-nucleotide_transport_n9 pSWI6-other_proteolytic_degradation_n7
100: pMCM1-nucleotide_transport_n9
010: pMCM1-nucleotide_transport_n9
110: pUME6-SCB pNDD1-MCM1
100: pNDD1-MCM1
010: pUME6-SCB
110: pSWI6-nucleotide_transport_n9 pFKH2-SFF'
100: pSWI6-nucleotide_transport_n9
110: pMCM1-SFF' pSWI6-other_proteolytic_degradation_n7
110: pSWI4-osmosensing_n6 pSWI4-SCB pFKH2-SFF'
010: pSWI4-osmosensing_n6 pSWI4-SCB
010: pSWI4-osmosensing_n6
010: pSWI4-SCB
100: pSWI4-osmosensing_n6 pFKH2-SFF'
100: pSWI4-osmosensing_n6
100: pSWI4-SCB
110: pSWI6-LYS14 pFKH2-SFF'
010: pSWI6-LYS14
110: pMCM1-nucleotide_transport_n9 pMCM1-SFF' pMCM1-MCM1
100: pMCM1-MCM1
010: pMCM1-MCM1
100: pMCM1-nucleotide_transport_n9
010: pMCM1-nucleotide_transport_n9 pMCM1-MCM1
010: pMCM1-nucleotide_transport_n9
110: pMCM1-MCM1 pSWI4-MCM1 pFKH2-MCM1
100: pFKH2-MCM1
100: pMCM1-MCM1 pSWI4-MCM1
100: pSWI4-MCM1
100: pMCM1-MCM1
010: pMCM1-MCM1
010: pFKH2-MCM1
110: pSWI4-MCM1 pNDD1-MCM1 pFKH2-SFF'
100: pSWI4-MCM1
100: pNDD1-MCM1

110: pMCM1-SFF' pSWI4-osmosensing_n6 pFKH2-SFF'
100: pMCM1-SFF' pFKH2-SFF'
010: pMCM1-SFF' pFKH2-SFF'
010: pSWI4-osmosensing_n6
100: pSWI4-osmosensing_n6 pFKH2-SFF'
100: pSWI4-osmosensing_n6
110: pSWI4-MCM1 pNDD1-MCM1 pFKH2-MCM1
100: pFKH2-MCM1
100: pSWI4-MCM1
100: pNDD1-MCM1
010: pFKH2-MCM1
110: pMCM1-nucleotide_transport_n9 pFKH2-SFF'
100: pMCM1-nucleotide_transport_n9
010: pMCM1-nucleotide_transport_n9
110: pMCM1-nucleotide_transport_n9 pFKH2-MCM1
100: pFKH2-MCM1
010: pFKH2-MCM1
100: pMCM1-nucleotide_transport_n9
010: pMCM1-nucleotide_transport_n9
110: pSWI4-MCM1 pSWI4-osmosensing_n6
010: pSWI4-osmosensing_n6
100: pSWI4-MCM1
100: pSWI4-osmosensing_n6
110: pSWI4-osmosensing_n6 pSWI4-SCB
010: pSWI4-osmosensing_n6
010: pSWI4-SCB
100: pSWI4-osmosensing_n6
100: pSWI4-SCB
110: pSWI4-osmosensing_n6 pFKH1-SFF'
010: pSWI4-osmosensing_n6
100: pSWI4-osmosensing_n6
110: pMCM1-nucleotide_transport_n9 pNDD1-MCM1
100: pNDD1-MCM1
100: pMCM1-nucleotide_transport_n9
010: pMCM1-nucleotide_transport_n9
110: pMCM1-nucleotide_transport_n9 pFKH1-SFF'
100: pMCM1-nucleotide_transport_n9
010: pMCM1-nucleotide_transport_n9
110: pSWI4-osmosensing_n6 pFKH2-SFF'
010: pSWI4-osmosensing_n6
100: pSWI4-osmosensing_n6
110: pSWI4-MCM1 pNDD1-MCM1
100: pSWI4-MCM1
100: pNDD1-MCM1
110: pUME6-SCB pFKH1-pentose-phosphate_pathway_n14
010: pUME6-SCB
100: pFKH1-pentose-phosphate_pathway_n14
110: pUME6-SCB pFKH1-SFF'
010: pUME6-SCB
110: pMCM1-SFF' pFKH1-pentose-phosphate_pathway_n14
100: pFKH1-pentose-phosphate_pathway_n14
110: pMCM1-MCM1 pFKH1-pentose-phosphate_pathway_n14
100: pMCM1-MCM1
010: pMCM1-MCM1
100: pFKH1-pentose-phosphate_pathway_n14
110: pSWI4-SCB pFKH1-SFF'
010: pSWI4-SCB
100: pSWI4-SCB
110: pMCM1-SFF' pSWI6-nucleotide_transport_n9
100: pSWI6-nucleotide_transport_n9
110: pNDD1-MCM1 pFKH2-MCM1
100: pFKH2-MCM1
100: pNDD1-MCM1
010: pFKH2-MCM1
110: pNDD1-MCM1 pFKH2-SFF'
100: pNDD1-MCM1
110: pMCM1-MCM1 pNDD1-MCM1
100: pMCM1-MCM1
010: pMCM1-MCM1
100: pNDD1-MCM1

110: pMCM1-SFF' pNDD1-MCM1
 100: pNDD1-MCM1
110: pNDD1-MCM1 pFKH1-SFF'
 100: pNDD1-MCM1
110: pFKH1-SFF' pFKH2-MCM1
 100: pFKH2-MCM1
 010: pFKH2-MCM1
110: pMCM1-SFF' pSWI4-osmosensing_n6
 010: pSWI4-osmosensing_n6
 100: pSWI4-osmosensing_n6
110: pMCM1-nucleotide_transport_n9 pMCM1-SFF'
 100: pMCM1-nucleotide_transport_n9
 010: pMCM1-nucleotide_transport_n9
110: pFKH1-pentose-phosphate_pathway_n14 pFKH2-SFF'
 100: pFKH1-pentose-phosphate_pathway_n14
110: pSWI6-LYS14 pFKH1-SFF'
 010: pSWI6-LYS14
110: pSWI6-LYS14 pFKH1-pentose-phosphate_pathway_n14
 010: pSWI6-LYS14
 100: pFKH1-pentose-phosphate_pathway_n14
110: pSWI6-LYS14 pSWI6-nucleotide_transport_n9
 100: pSWI6-nucleotide_transport_n9
 010: pSWI6-LYS14
110: pSWI6-other_proteolytic_degradation_n7 pSWI6-nucleotide_transport_n9
 100: pSWI6-nucleotide_transport_n9
110: pSWI6-nucleotide_transport_n9 pFKH1-SFF'
 100: pSWI6-nucleotide_transport_n9
110: pMCM1-MCM1 pSWI4-MCM1 pNDD1-MCM1
 100: pMCM1-MCM1 pSWI4-MCM1
 100: pSWI4-MCM1
 100: pMCM1-MCM1
 010: pMCM1-MCM1
 100: pNDD1-MCM1
110: pSWI6-MCB pMBP1-STRE'
110: pMBP1-chromatin_modification_n9 pMBP1-MCB
110: pMBP1-STRE' pMBP1-MCB
110: pSWI6-cytok9 pMBP1-MCB
 010: pSWI6-cytok9
110: pMBP1-STRE' pSWI6-SWI5
 100: pSWI6-SWI5
110: pSWI6-MCB pSWI6-cytok9
 010: pSWI6-cytok9
110: pSWI6-MCB pSWI6-other_pheromone_response_activities_n8
 010: pSWI6-other_pheromone_response_activities_n8
110: pSWI6-metal_ion_transporters_n10 pSWI6-SWI5
 100: pSWI6-SWI5
 100: pSWI6-metal_ion_transporters_n10
110: pSWI6-other_pheromone_response_activities_n8 pMBP1-MCB
 010: pSWI6-other_pheromone_response_activities_n8
110: pSWI6-cytok9 pSWI6-other_pheromone_response_activities_n8
 010: pSWI6-cytok9
 010: pSWI6-other_pheromone_response_activities_n8
110: pSWI6-metal_ion_transporters_n10 pMBP1-STRE'
 100: pSWI6-metal_ion_transporters_n10
110: pSWI6-other_pheromone_response_activities_n8 pSWI6-SWI5
 010: pSWI6-other_pheromone_response_activities_n8
 100: pSWI6-SWI5
110: pMBP1-mPROTEOL18(proteolysis_n18) pSWI6-SWI5
 100: pSWI6-SWI5
 010: pMBP1-mPROTEOL18(proteolysis_n18)
110: pSWI6-mPROTEOL18(proteolysis_n18) pSWI6-SWI5
 100: pSWI6-mPROTEOL18(proteolysis_n18)
 100: pSWI6-SWI5
 010: pSWI6-mPROTEOL18(proteolysis_n18)
110: pSWI6-STRE pMBP1-mPROTEOL18(proteolysis_n18)
 100: pSWI6-STRE
 010: pMBP1-mPROTEOL18(proteolysis_n18)
110: pSWI6-STRE pSWI6-mPROTEOL18(proteolysis_n18)
 100: pSWI6-mPROTEOL18(proteolysis_n18)
 100: pSWI6-STRE

010: pSWI6-mPROTEOL18(proteolysis_n18)
110: pMBP1-mPROTEOL18(proteolysis_n18) pSWI6-mPROTEOL18(proteolysis_n18)
100: pSWI6-mPROTEOL18(proteolysis_n18)
010: pMBP1-mPROTEOL18(proteolysis_n18)
010: pSWI6-mPROTEOL18(proteolysis_n18)
110: pNDD1-MCM1' pNDD1-MCM1
100: pNDD1-MCM1'
100: pNDD1-MCM1
110: pXBP1-other_protein-destination_activities_n7 pNDD1-MERE4
100: pXBP1-other_protein-destination_activities_n7
010: pXBP1-other_protein-destination_activities_n7
110: pFKH1-SFF' pSWI6-nutritional_response_pathway_n7
010: pSWI6-nutritional_response_pathway_n7
110: pSWI6-MCB pSWI6-morphogenesis_n5
010: pSWI6-morphogenesis_n5
100: pSWI6-morphogenesis_n5
110: pSWI6-nutritional_response_pathway_n7 pFKH1-SFF
010: pSWI6-nutritional_response_pathway_n7
110: pSWI6-morphogenesis_n5 pMBP1-MCB
010: pSWI6-morphogenesis_n5
100: pSWI6-morphogenesis_n5
110: pSWI6-nucleotide_transport_n9 pSWI6-other_cation_transporters_n7
100: pSWI6-nucleotide_transport_n9
010: pSWI6-other_cation_transporters_n7
110: pSWI6-other_pheromone_response_activities_n8 pSWI6-nutritional_response_pathway_n7
010: pSWI6-nutritional_response_pathway_n7
010: pSWI6-other_pheromone_response_activities_n8
110: pSWI6-nutritional_response_pathway_n7 pMBP1-MCB
010: pSWI6-nutritional_response_pathway_n7
110: pSWI6-MCB pSWI6-nutritional_response_pathway_n7
010: pSWI6-nutritional_response_pathway_n7
110: pSWI6-cytok9 pSWI6-nutritional_response_pathway_n7
010: pSWI6-cytok9
010: pSWI6-nutritional_response_pathway_n7
110: pSWI6-other_pheromone_response_activities_n8 pFKH1-SFF'
010: pSWI6-other_pheromone_response_activities_n8
110: pSWI6-other_pheromone_response_activities_n8 pFKH1-SFF
010: pSWI6-other_pheromone_response_activities_n8
110: pSWI6-MCB pSWI6-nucleotide_transport_n9
100: pSWI6-nucleotide_transport_n9
110: pSWI4-MCM1' pSWI6-LYS14 pFKH2-SFF'
100: pSWI4-MCM1'
010: pSWI6-LYS14
010: pSWI4-MCM1'
100: pSWI4-MCM1' pFKH2-SFF'
110: pSWI6-ionic_homeostasis_n6 pSWI4-MCM1' pFKH2-SFF'
100: pSWI4-MCM1'
100: pSWI6-ionic_homeostasis_n6
010: pSWI4-MCM1'
100: pSWI4-MCM1' pFKH2-SFF'
110: pSWI4-MCM1' pUPC2-ALPHA1' pFKH2-SFF'
100: pSWI4-MCM1'
010: pSWI4-MCM1'
100: pSWI4-MCM1' pFKH2-SFF'
110: pSWI4-MCM1' pUPC2-ALPHA1' pFKH1-SFF'
100: pSWI4-MCM1'
010: pUPC2-ALPHA1' pFKH1-SFF'
010: pSWI4-MCM1'
100: pSWI4-MCM1' pFKH1-SFF'
110: pSWI6-ionic_homeostasis_n6 pSWI6-LYS14 pFKH2-SFF
100: pSWI6-ionic_homeostasis_n6
010: pSWI6-LYS14
110: pTEC1-other_energy_generation_activities_n17 pFKH2-SFF
110: pTEC1-other_energy_generation_activities_n17 pFKH1-SFF
110: pSWI6-ionic_homeostasis_n6 pFKH1-SFF'
100: pSWI6-ionic_homeostasis_n6
110: pSWI6-ionic_homeostasis_n6 pFKH2-SFF
100: pSWI6-ionic_homeostasis_n6
110: pSWI6-LYS14 pFKH1-SFF
010: pSWI6-LYS14

110: pSWI6-ionic_homeostasis_n6 pFKH2-SFF'
100: pSWI6-ionic_homeostasis_n6
110: pSWI6-LYS14 pFKH2-SFF
010: pSWI6-LYS14
110: pSWI6-ionic_homeostasis_n6 pFKH1-SFF
100: pSWI6-ionic_homeostasis_n6
110: pSWI6-ionic_homeostasis_n6 pSWI6-LYS14
100: pSWI6-ionic_homeostasis_n6
010: pSWI6-LYS14
110: pSWI6-ionic_homeostasis_n6 pSWI4-MCM1'
100: pSWI4-MCM1'
100: pSWI6-ionic_homeostasis_n6
010: pSWI4-MCM1'
110: pSWI4-MCM1' pFKH1-SFF
100: pSWI4-MCM1'
010: pSWI4-MCM1'
110: pSWI4-MCM1' pFKH2-SFF
100: pSWI4-MCM1'
010: pSWI4-MCM1'
110: pSWI4-MCM1' pFKH2-SFF'
100: pSWI4-MCM1'
010: pSWI4-MCM1'
110: pSWI4-SCB pFKH2-SFF
010: pSWI4-SCB
100: pSWI4-SCB
110: pSWI4-SCB pFKH2-SFF'
010: pSWI4-SCB
100: pSWI4-SCB
110: pSWI4-MCM1' pFKH1-SFF'
100: pSWI4-MCM1'
010: pSWI4-MCM1'
110: pSWI6-ionic_homeostasis_n6 pSWI4-MCM1' pFKH2-SFF
100: pSWI4-MCM1'
100: pSWI6-ionic_homeostasis_n6
010: pSWI4-MCM1'
100: pSWI4-MCM1' pFKH2-SFF
110: pSWI4-MCM1' pUPC2-ALPHA1' pFKH1-SFF
100: pSWI4-MCM1'
010: pUPC2-ALPHA1' pFKH1-SFF
010: pSWI4-MCM1'
100: pSWI4-MCM1' pFKH1-SFF
110: pSWI4-MCM1' pSWI6-LYS14 pFKH2-SFF
100: pSWI4-MCM1'
010: pSWI6-LYS14
010: pSWI4-MCM1'
100: pSWI4-MCM1' pFKH2-SFF
110: pSWI4-MCM1' pUPC2-ALPHA1' pFKH2-SFF
100: pSWI4-MCM1'
010: pSWI4-MCM1'
100: pSWI4-MCM1' pFKH2-SFF
110: pSWI6-ionic_homeostasis_n6 pSWI6-LYS14 pFKH2-SFF'
100: pSWI6-ionic_homeostasis_n6
010: pSWI6-LYS14
110: pSTB4-ALPHA1' pFKH1-pentose-phosphate_pathway_n14
100: pSTB4-ALPHA1'
100: pFKH1-pentose-phosphate_pathway_n14
110: pMCM1-MCM1 pFKH1-pheromone_response_generation_n12
100: pMCM1-MCM1
010: pMCM1-MCM1
110: pSTB4-ALPHA1' pFKH1-pheromone_response_generation_n12
100: pSTB4-ALPHA1'
110: pYFL052w-MCB pSPT23-other_cell_growth_cell_division_and_dna_synthesis_activities_n10.scn
110: pUGA3-other_intracellular-transport_activities_n6 pMIG2-MCB
110: pYFL052w-MCB pMCM1-ECB
100: pMCM1-ECB
010: pMCM1-ECB
110: pUGA3-other_intracellular-transport_activities_n6 pMCM1-MCM1
100: pMCM1-MCM1
010: pMCM1-MCM1
110: pMCM1-ECB pMIG2-MCB

100: pMCM1-ECB
010: pMCM1-ECB
110: pMCM1-SFF' pMIG2-MCB
110: pUGA3-other_intracellular-transport_activities_n6 pFKH1-pheromone_response_generation_n12
110: pYNR063W-MCB pMCM1-MCM1'
100: pMCM1-MCM1'
110: pSPT23-other_cell_growth_cell_division_and_dna_synthesis_activities_n10.scn pSTB4-MCB
110: pMCM1-MCM1' pFKH1-pheromone_response_generation_n12
100: pMCM1-MCM1'
110: pSPT23-other_cell_growth_cell_division_and_dna_synthesis_activities_n10.scn pFKH1-pheromone_response_generation_n12
110: pYOX1-ECB pUGA3-other_intracellular-transport_activities_n6
010: pYOX1-ECB
110: pMCM1-MCM1 pUGA3-amino-acid_degradation_n32
100: pMCM1-MCM1
010: pMCM1-MCM1
110: pYNR063W-MCB pYOX1-MCM1
010: pYOX1-MCM1
110: pYFL052w-MCB pFKH1-pentose-phosphate_pathway_n14
100: pFKH1-pentose-phosphate_pathway_n14
110: pYFL052w-MCB pFKH1-pheromone_response_generation_n12
110: pSPT23-other_cell_growth_cell_division_and_dna_synthesis_activities_n10.scn pMIG2-MCB
110: pSPT23-other_cell_growth_cell_division_and_dna_synthesis_activities_n10.scn pUGA3-amino-acid_degradation_n32
110: pYOX1-ECB pMIG2-MCB
010: pYOX1-ECB
110: pYOX1-MCM1 pSTB4-MCB
010: pYOX1-MCM1
110: pMCM1-MCM1' pUGA3-amino-acid_degradation_n32
100: pMCM1-MCM1'
110: pSPT23-other_cell_growth_cell_division_and_dna_synthesis_activities_n10.scn pMCM1-MCM1'
100: pMCM1-MCM1'
110: pSTB4-MCB pMCM1-ECB
100: pMCM1-ECB
010: pMCM1-ECB
110: pMCM1-MCM1' pSTB4-MCB
100: pMCM1-MCM1'
110: pYFL052w-MCB pMCM1-MCM1'
100: pMCM1-MCM1'
110: pYFL052w-MCB pFKH1-SFF'
110: pMCM1-SFF' pSTB4-MCB
110: pMCM1-MCM1' pMIG2-MCB
100: pMCM1-MCM1'
110: pYFL052w-MCB pMCM1-SFF'
110: pSTB4-ALPHA1' pMCM1-MCM1'
100: pMCM1-MCM1'
100: pSTB4-ALPHA1'
110: pSPT23-other_cell_growth_cell_division_and_dna_synthesis_activities_n10.scn pFKH1-pentose-phosphate_pathway_n14
100: pFKH1-pentose-phosphate_pathway_n14
110: pFKH1-pentose-phosphate_pathway_n14 pMIG2-MCB
100: pFKH1-pentose-phosphate_pathway_n14
110: pYFL052w-MCB pYOX1-MCM1
010: pYOX1-MCM1
110: pYNR063W-MCB pFKH1-pentose-phosphate_pathway_n14
100: pFKH1-pentose-phosphate_pathway_n14
110: pYNR063W-MCB pMCM1-SFF'
110: pYFL052w-MCB pMIG2-MCB
110: pSPT23-other_cell_growth_cell_division_and_dna_synthesis_activities_n10.scn pMCM1-SFF'
110: pSTB4-MCB pFKH1-pheromone_response_generation_n12
110: pFKH1-pheromone_response_generation_n12 pFKH1-pentose-phosphate_pathway_n14
100: pFKH1-pentose-phosphate_pathway_n14
110: pYOX1-ECB pYNR063W-MCB
010: pYOX1-ECB
110: pUGA3-amino-acid_degradation_n32 pFKH1-pentose-phosphate_pathway_n14
100: pFKH1-pentose-phosphate_pathway_n14
110: pUGA3-other_intracellular-transport_activities_n6 pFKH1-pentose-phosphate_pathway_n14
100: pFKH1-pentose-phosphate_pathway_n14
110: pUGA3-amino-acid_degradation_n32 pYOX1-MCM1
010: pYOX1-MCM1
110: pFKH1-SFF' pMIG2-MCB
110: pUGA3-other_intracellular-transport_activities_n6 pYOX1-MCM1
010: pYOX1-MCM1

110: pYNR063W-MCB pMCM1-ECB
100: pMCM1-ECB
010: pMCM1-ECB
110: pYOX1-ECB pSPT23-other_cell_growth_cell_division_and_dna_synthesis_activities_n10.scn
010: pYOX1-ECB
110: pSTB4-ALPHA1' pUGA3-other_intracellular-transport_activities_n6
100: pSTB4-ALPHA1'
110: pUGA3-amino-acid_degradation_n32 pMCM1-ECB
100: pMCM1-ECB
010: pMCM1-ECB
110: pSPT23-other_cell_growth_cell_division_and_dna_synthesis_activities_n10.scn pUGA3-other_intracellular-transport_activities_n6
110: pYNR063W-MCB pUGA3-other_intracellular-transport_activities_n6
110: pUGA3-other_intracellular-transport_activities_n6 pFKH1-SFF'
110: pUGA3-other_intracellular-transport_activities_n6 pSTB4-MCB
110: pSTB4-ALPHA1' pMCM1-ECB
100: pMCM1-ECB
010: pMCM1-ECB
100: pSTB4-ALPHA1'
110: pSTB4-ALPHA1' pUGA3-amino-acid_degradation_n32
100: pSTB4-ALPHA1'
110: pYNR063W-MCB pMIG2-MCB
110: pYOX1-ECB pUGA3-amino-acid_degradation_n32
010: pYOX1-ECB
110: pYFL052w-MCB pUGA3-other_intracellular-transport_activities_n6
110: pYOX1-ECB pYFL052w-MCB
010: pYOX1-ECB
110: pMCM1-ECB pFKH1-pheromone_response_generation_n12
100: pMCM1-ECB
010: pMCM1-ECB
110: pUGA3-amino-acid_degradation_n32 pMIG2-MCB
110: pYOX1-ECB pSTB4-MCB
010: pYOX1-ECB
110: pYFL052w-MCB pUGA3-amino-acid_degradation_n32
110: pYFL052w-MCB pMCM1-MCM1
100: pMCM1-MCM1
010: pMCM1-MCM1
110: pSTB4-ALPHA1' pMIG2-MCB
100: pSTB4-ALPHA1'
110: pMCM1-SFF' pUGA3-amino-acid_degradation_n32
110: pMCM1-SFF' pFKH1-pheromone_response_generation_n12
110: pSPT23-other_cell_growth_cell_division_and_dna_synthesis_activities_n10.scn pMCM1-MCM1
100: pMCM1-MCM1
010: pMCM1-MCM1
110: pSPT23-other_cell_growth_cell_division_and_dna_synthesis_activities_n10.scn pMCM1-ECB
100: pMCM1-ECB
010: pMCM1-ECB
110: pYNR063W-MCB pMCM1-MCM1
100: pMCM1-MCM1
010: pMCM1-MCM1
110: pYNR063W-MCB pSPT23-other_cell_growth_cell_division_and_dna_synthesis_activities_n10.scn
110: pYOX1-MCM1 pFKH1-pheromone_response_generation_n12
010: pYOX1-MCM1
110: pUGA3-amino-acid_degradation_n32 pFKH1-pheromone_response_generation_n12
110: pYNR063W-MCB pUGA3-amino-acid_degradation_n32
110: pSTB4-MCB pFKH1-SFF'
110: pYOX1-MCM1 pMIG2-MCB
010: pYOX1-MCM1
110: pSTB4-MCB pFKH1-pentose-phosphate_pathway_n14
100: pFKH1-pentose-phosphate_pathway_n14
110: pSPT23-other_cell_growth_cell_division_and_dna_synthesis_activities_n10.scn pYOX1-MCM1
010: pYOX1-MCM1
110: pMCM1-MCM1 pMIG2-MCB
100: pMCM1-MCM1
010: pMCM1-MCM1
110: pUGA3-other_intracellular-transport_activities_n6 pUGA3-amino-acid_degradation_n32
110: pMCM1-MCM1 pSTB4-MCB
100: pMCM1-MCM1
010: pMCM1-MCM1
110: pYNR063W-MCB pFKH1-SFF'
110: pYOX1-ECB pFKH1-pheromone_response_generation_n12

010: pYOX1-ECB
110: pUGA3-amino-acid_degradation_n32 pSTB4-MCB
110: pYNR063W-MCB pFKH1-pheromone_response_generation_n12
110: pSTB4-ALPHA1' pMCM1-MCM1
100: pMCM1-MCM1
010: pMCM1-MCM1
100: pSTB4-ALPHA1'
110: pFKH1-pheromone_response_generation_n12 pMIG2-MCB
110: pYOX1-ECB pSTB4-ALPHA1'
100: pSTB4-ALPHA1'
010: pYOX1-ECB
110: pSTB4-ALPHA1' pYOX1-MCM1
100: pSTB4-ALPHA1'
010: pYOX1-MCM1
110: pSTB4-ALPHA1' pMCM1-SFF'
100: pSTB4-ALPHA1'
110: pSWI6-other_pheromone_response_activities_n8 pFKH1-pheromone_response_generation_n12
010: pSWI6-other_pheromone_response_activities_n8
110: pFKH2-metal_ion_transporters_n17 pFKH1-SFF
010: pFKH2-metal_ion_transporters_n17
110: pFKH1-pheromone_response_generation_n12 pSWI6-nutritional_response_pathway_n7
010: pSWI6-nutritional_response_pathway_n7
110: pFKH2-metal_ion_transporters_n17 pFKH1-SFF'
010: pFKH2-metal_ion_transporters_n17
110: pFKH2-SFF' pFKH1-SFF pROX1-osmosensing_n6
100: pFKH2-SFF' pFKH1-SFF
100: pROX1-osmosensing_n6
010: pROX1-osmosensing_n6
110: pFKH1-SFF' pFKH2-SFF' pROX1-osmosensing_n6
100: pFKH1-SFF' pFKH2-SFF'
100: pROX1-osmosensing_n6
010: pROX1-osmosensing_n6
010: pFKH1-SFF' pFKH2-SFF'
110: pFKH1-organization_of_golgi_n7 pROX1-osmosensing_n6
100: pROX1-osmosensing_n6
010: pROX1-osmosensing_n6
110: pFKH1-SFF' pROX1-osmosensing_n6
100: pROX1-osmosensing_n6
010: pROX1-osmosensing_n6
110: pFKH1-SFF pGAT1-rRSE10
100: pGAT1-rRSE10
110: pFKH1-SFF pROX1-osmosensing_n6
100: pROX1-osmosensing_n6
010: pROX1-osmosensing_n6
110: pFKH1-SFF' pGAT1-rRSE10
100: pGAT1-rRSE10
110: pSWI6-allantoin_and_allantoate_transporters_n13 pMBP1-abc_transporters_n10
110: pSWI6-glyoxylate_cycle_n19 pMBP1-abc_transporters_n10
110: pSWI6-breakdown_of_lipids_fatty_acids_and_isoprenoids_n8 pSWI6-SWI5
100: pSWI6-SWI5
110: pSWI6-metal_ion_transporters_n10 pMBP1-MCB
100: pSWI6-metal_ion_transporters_n10
110: pMBP1-breakdown_of_lipids_fatty_acids_and_isoprenoids_n8 pACE2-allantoin_and_allantoate_transporters_n7
110: pSWI6-MCB pSWI6-metal_ion_transporters_n10
100: pSWI6-metal_ion_transporters_n10
110: pSWI6-deoxyribonucleotide_metabolisn10 pSWI6-SWI5
100: pSWI6-SWI5
110: pSWI6-nutritional_response_pathway_n7 pSWI6-SWI5
010: pSWI6-nutritional_response_pathway_n7
100: pSWI6-SWI5
110: pSWI6-breakdown_of_lipids_fatty_acids_and_isoprenoids_n8 pACE2-allantoin_and_allantoate_transporters_n7
110: pMBP1-breakdown_of_lipids_fatty_acids_and_isoprenoids_n8 pSWI6-SWI5
100: pSWI6-SWI5
110: pACE2-nutritional_response_pathway_n7 pSWI6-nutritional_response_pathway_n7
010: pSWI6-nutritional_response_pathway_n7
110: pSWI6-abc_transporters_n10 pSWI6-glyoxylate_cycle_n19
110: pYNR063W-MCB pRPI1-SFF
010: pRPI1-SFF
100: pRPI1-SFF
001: pRPI1-SFF

110: pMBP1-breakdown_of_lipids_fatty_acids_and_isoprenoids_n8 pMBP1-MCB
110: pACE2-nutritional_response_pathway_n7 pMBP1-MCB
110: pSWI6-abc_transporters_n10 pSWI6-allantoin_and_allantoate_transporters_n13
110: pACE2-allantoin_and_allantoate_transporters_n7 pYDR049W-biogenesis_of_chromosome_structure_n18
110: pYFL052w-MCB pRPI1-SFF
010: pRPI1-SFF
100: pRPI1-SFF
001: pRPI1-SFF
110: pSWI6-MCB pACE2-nutritional_response_pathway_n7
110: pMBP1-breakdown_of_lipids_fatty_acids_and_isoprenoids_n8 pSWI6-breakdown_of_lipids_fatty_acids_and_isoprenoids_n8 pSWI6-SWI5
100: pSWI6-SWI5
110: pACE2-allantoin_and_allantoate_transporters_n7 pFKH2-SFF
110: pACE2-allantoin_and_allantoate_transporters_n7 pFKH2-SFF'
110: pFKH1-organization_of_golgi_n7 pFKH2-SFF'
110: pFKH1-organization_of_golgi_n7 pFKH2-SFF
110: pUME6-SCB pSWI6-LYS14 pFKH1-pentose-phosphate_pathway_n14
010: pSWI6-LYS14
010: pUME6-SCB
100: pFKH1-pentose-phosphate_pathway_n14
110: pSWI6-other_transport_facilitators_n5 pFKH1-pentose-phosphate_pathway_n14
010: pSWI6-other_transport_facilitators_n5
100: pFKH1-pentose-phosphate_pathway_n14
110: pUME6-SCB pSWI6-LYS14 pFKH1-SFF'
010: pSWI6-LYS14
010: pUME6-SCB
110: pUME6-SCB pSWI6-LYS14 pFKH2-SFF'
010: pSWI6-LYS14
010: pUME6-SCB
110: pSWI4-MCM1' pDIG1-ECB
100: pSWI4-MCM1'
010: pSWI4-MCM1'
110: pSWI6-MCB pDIG1-ECB
110: pSWI4-MCM1 pDIG1-ECB
100: pSWI4-MCM1
110: pDIG1-ECB pMBP1-MCB
110: pMCM1-pentose-phosphate_pathway_n5 pMCM1-ECB
100: pMCM1-pentose-phosphate_pathway_n5
100: pMCM1-ECB
010: pMCM1-ECB
110: pSWI6-MCB pSWI4-MCM1'
100: pSWI4-MCM1'
010: pSWI4-MCM1'
110: pSWI4-MCM1 pMBP1-MCB
100: pSWI4-MCM1
110: pSWI6-MCB pSWI4-MCM1
100: pSWI4-MCM1
110: pSWI4-MCM1' pMBP1-MCB
100: pSWI4-MCM1'
010: pSWI4-MCM1'
110: pSKN7-g-proteins_n12 pXBP1-nitrogen_and_sulphur_metabolisn17
100: pXBP1-nitrogen_and_sulphur_metabolisn17
110: pSKN7-g-proteins_n12 pSKN7-other_energy_generation_activities_n16
110: pSWI6-nucleotide_transport_n9 pSWI6-allantoin_and_allantoate_transporters_n13
100: pSWI6-nucleotide_transport_n9
110: pSWI6-other_transport_facilitators_n5 pINO4-other_transport_facilitators_n5
010: pSWI6-other_transport_facilitators_n5
110: pSKN7-SWI5 pINO4-anion_transporters_n15
010: pSKN7-SWI5
100: pSKN7-SWI5
100: pINO4-anion_transporters_n15
010: pINO4-anion_transporters_n15
110: pSKN7-g-proteins_n12 pSWI6-nucleotide_transport_n9
100: pSWI6-nucleotide_transport_n9
110: pSWI6-allantoin_and_allantoate_transporters_n13 pSWI6-SWI5
100: pSWI6-SWI5
110: pSWI6-MCB pSWI6-allantoin_and_allantoate_transporters_n13
110: pSKN7-g-proteins_n12 pSKN7-amino-acid_transporters_n11
010: pSKN7-amino-acid_transporters_n11
110: pINO4-other_signal-transduction_activities_n8 pINO4-g-proteins_n11
100: pINO4-other_signal-transduction_activities_n8

010: pINO4-g-proteins_n11
001: pINO4-g-proteins_n11
100: pINO4-g-proteins_n11
110: pSKN7-other_mrna-transcription_activities_n20 pSKN7-nitrogen_and_sulphur_metabolisn17
010: pSKN7-other_mrna-transcription_activities_n20
110: pSWI6-SWI5 pSKN7-other_energy_generation_activities_n16
100: pSWI6-SWI5
110: pMTH1-pentose-phosphate_pathway_n14 pINO4-g-proteins_n11 pINO4-SWI5
010: pINO4-g-proteins_n11
100: pINO4-g-proteins_n11 pINO4-SWI5
001: pINO4-g-proteins_n11
010: pINO4-g-proteins_n11 pINO4-SWI5
010: pMTH1-pentose-phosphate_pathway_n14
100: pMTH1-pentose-phosphate_pathway_n14
001: pINO4-g-proteins_n11 pINO4-SWI5
100: pINO4-g-proteins_n11
110: pINO4-other_transport_facilitators_n5 pMTH1-pentose-phosphate_pathway_n14
010: pMTH1-pentose-phosphate_pathway_n14
100: pMTH1-pentose-phosphate_pathway_n14
110: pMTH1-anion_transporters_n15 pSWI6-SWI5
100: pSWI6-SWI5
100: pMTH1-anion_transporters_n15
110: pSKN7-SWI5 pINO4-glyoxylate_cycle_n8
010: pSKN7-SWI5
010: pINO4-glyoxylate_cycle_n8
100: pSKN7-SWI5
110: pSKN7-SWI5 pSKN7-amino-acid_transporters_n11
010: pSKN7-SWI5
100: pSKN7-SWI5
010: pSKN7-amino-acid_transporters_n11
110: pINO4-g-proteins_n11 pINO4-g-proteins_n12
010: pINO4-g-proteins_n11
001: pINO4-g-proteins_n11
100: pINO4-g-proteins_n11
110: pINO4-g-proteins_n11 pSKN7-nitrogen_and_sulphur_metabolisn17
010: pINO4-g-proteins_n11
001: pINO4-g-proteins_n11
100: pINO4-g-proteins_n11
110: pSKN7-SWI5 pSWI6-nucleotide_transport_n9 pSWI6-SWI5
010: pSKN7-SWI5
100: pSWI6-nucleotide_transport_n9
100: pSWI6-SWI5
100: pSKN7-SWI5
100: pSKN7-SWI5 pSWI6-SWI5
010: pSKN7-SWI5 pSWI6-SWI5
110: pSKN7-amino-acid_transporters_n11 pINO4-SWI5
010: pSKN7-amino-acid_transporters_n11
110: pMTH1-deoxyribonucleotide_metabolisn23 pINO4-SWI5
010: pMTH1-deoxyribonucleotide_metabolisn23
100: pMTH1-deoxyribonucleotide_metabolisn23
110: pSWI6-other_transport_facilitators_n5 pINO4-g-proteins_n11
010: pINO4-g-proteins_n11
001: pINO4-g-proteins_n11
010: pSWI6-other_transport_facilitators_n5
100: pINO4-g-proteins_n11
110: pINO4-other_transport_facilitators_n5 pINO4-other_mrna-transcription_activities_n20
010: pINO4-other_mrna-transcription_activities_n20
110: pMTH1-pentose-phosphate_pathway_n14 pSWI6-SWI5
100: pSWI6-SWI5
010: pMTH1-pentose-phosphate_pathway_n14
100: pMTH1-pentose-phosphate_pathway_n14
110: pINO4-other_mrna-transcription_activities_n20 pSKN7-nitrogen_and_sulphur_metabolisn17
010: pINO4-other_mrna-transcription_activities_n20
110: pMTH1-pentose-phosphate_pathway_n14 pINO4-other_mrna-transcription_activities_n20
010: pINO4-other_mrna-transcription_activities_n20
010: pMTH1-pentose-phosphate_pathway_n14
100: pMTH1-pentose-phosphate_pathway_n14
110: pINO4-g-proteins_n11 pXBP1-other_mrna-transcription_activities_n20
010: pINO4-g-proteins_n11
001: pINO4-g-proteins_n11

100: pXBP1-other_mrna-transcription_activities_n20
001: pXBP1-other_mrna-transcription_activities_n20
100: pINO4-g-proteins_n11
110: pSWI6-other_transport_facilitators_n5 pINO4-other_mrna-transcription_activities_n20
010: pINO4-other_mrna-transcription_activities_n20
010: pSWI6-other_transport_facilitators_n5
110: pSWI6-allantoin_and_allantoate_transporters_n13 pMTH1-anion_transporters_n15
100: pMTH1-anion_transporters_n15
110: pMTH1-lipid_and_fatty-acid_transport_n11 pSKN7-other_mrna-transcription_activities_n20
100: pMTH1-lipid_and_fatty-acid_transport_n11
010: pSKN7-other_mrna-transcription_activities_n20
110: pSKN7-lipid_and_fatty-acid_transport_n11 pINO4-g-proteins_n11
010: pSKN7-lipid_and_fatty-acid_transport_n11
010: pINO4-g-proteins_n11
100: pSKN7-lipid_and_fatty-acid_transport_n11
001: pINO4-g-proteins_n11
100: pINO4-g-proteins_n11
110: pSKN7-lipid_and_fatty-acid_transport_n11 pINO4-other_mrna-transcription_activities_n20
010: pSKN7-lipid_and_fatty-acid_transport_n11
100: pSKN7-lipid_and_fatty-acid_transport_n11
010: pINO4-other_mrna-transcription_activities_n20
110: pINO4-glyoxylate_cycle_n8 pINO4-other_mrna-transcription_activities_n20
010: pINO4-glyoxylate_cycle_n8
010: pINO4-other_mrna-transcription_activities_n20
110: pINO4-other_mrna-transcription_activities_n20 pINO4-g-proteins_n11
010: pINO4-g-proteins_n11
001: pINO4-g-proteins_n11
010: pINO4-other_mrna-transcription_activities_n20
100: pINO4-g-proteins_n11
110: pSKN7-SWI5 pINO4-SWI5
010: pSKN7-SWI5
100: pSKN7-SWI5
110: pCIN5-other_transport_facilitators_n5 pSWI6-other_transport_facilitators_n5
010: pSWI6-other_transport_facilitators_n5
110: pINO4-other_signal-transduction_activities_n8 pINO4-osmosensing_n6
100: pINO4-other_signal-transduction_activities_n8
010: pINO4-osmosensing_n6
100: pINO4-osmosensing_n6
110: pSWI6-SWI5 pINO4-g-proteins_n11
010: pINO4-g-proteins_n11
100: pSWI6-SWI5
001: pINO4-g-proteins_n11
100: pINO4-g-proteins_n11
110: pINO4-other_signal-transduction_activities_n8 pINO4-other_mrna-transcription_activities_n20
100: pINO4-other_signal-transduction_activities_n8
010: pINO4-other_mrna-transcription_activities_n20
110: pINO4-other_transport_facilitators_n5 pSWI6-SWI5
100: pSWI6-SWI5
110: pINO4-other_mrna-transcription_activities_n20 pINO4-g-proteins_n11 pINO4-SWI5
010: pINO4-other_mrna-transcription_activities_n20 pINO4-g-proteins_n11
010: pINO4-g-proteins_n11
100: pINO4-g-proteins_n11 pINO4-SWI5
001: pINO4-g-proteins_n11
010: pINO4-other_mrna-transcription_activities_n20 pINO4-SWI5
010: pINO4-other_mrna-transcription_activities_n20
010: pINO4-g-proteins_n11 pINO4-SWI5
001: pINO4-g-proteins_n11 pINO4-SWI5
001: pINO4-other_mrna-transcription_activities_n20 pINO4-SWI5
100: pINO4-g-proteins_n11
110: pINO4-other_mrna-transcription_activities_n20 pXBP1-other_mrna-transcription_activities_n20
100: pXBP1-other_mrna-transcription_activities_n20
010: pINO4-other_mrna-transcription_activities_n20
001: pXBP1-other_mrna-transcription_activities_n20
110: pSKN7-SWI5 pINO4-osmosensing_n6
010: pSKN7-SWI5
010: pINO4-osmosensing_n6
100: pSKN7-SWI5
100: pINO4-osmosensing_n6
110: pINO4-g-proteins_n11 pINO4-osmosensing_n6
010: pINO4-g-proteins_n11

010: pINO4-osmosensing_n6
001: pINO4-g-proteins_n11
100: pINO4-osmosensing_n6
100: pINO4-g-proteins_n11
110: pMTH1-deoxyribonucleotide_metabolisn23 pMTH1-lipid_and_fatty-acid_transport_n11
010: pMTH1-deoxyribonucleotide_metabolisn23
100: pMTH1-lipid_and_fatty-acid_transport_n11
100: pMTH1-deoxyribonucleotide_metabolisn23
110: pSKN7-SWI5 pSWI6-SWI5
010: pSKN7-SWI5
100: pSWI6-SWI5
100: pSKN7-SWI5
110: pSWI6-SWI5 pSKN7-amino-acid_transporters_n11
100: pSWI6-SWI5
010: pSKN7-amino-acid_transporters_n11
110: pSWI6-nucleotide_transport_n9 pSKN7-amino-acid_transporters_n11
100: pSWI6-nucleotide_transport_n9
010: pSKN7-amino-acid_transporters_n11
110: pMTH1-pentose-phosphate_pathway_n14 pINO4-g-proteins_n11
010: pINO4-g-proteins_n11
001: pINO4-g-proteins_n11
010: pMTH1-pentose-phosphate_pathway_n14
100: pMTH1-pentose-phosphate_pathway_n14
100: pINO4-g-proteins_n11
110: pMTH1-lipid_and_fatty-acid_transport_n11 pSWI6-SWI5
100: pSWI6-SWI5
100: pMTH1-lipid_and_fatty-acid_transport_n11
110: pINO4-glyoxylate_cycle_n8 pINO4-g-proteins_n11
010: pINO4-g-proteins_n11
010: pINO4-glyoxylate_cycle_n8
001: pINO4-g-proteins_n11
100: pINO4-g-proteins_n11
110: pMTH1-pentose-phosphate_pathway_n14 pMTH1-anion_transporters_n15
100: pMTH1-anion_transporters_n15
010: pMTH1-pentose-phosphate_pathway_n14
100: pMTH1-pentose-phosphate_pathway_n14
110: pMTH1-lipid_and_fatty-acid_transport_n11 pMTH1-anion_transporters_n15
100: pMTH1-anion_transporters_n15
100: pMTH1-lipid_and_fatty-acid_transport_n11
110: pINO4-glyoxylate_cycle_n8 pINO4-SWI5
010: pINO4-glyoxylate_cycle_n8
110: pMTH1-lipid_and_fatty-acid_transport_n11 pSWI6-nucleotide_transport_n9
100: pSWI6-nucleotide_transport_n9
100: pMTH1-lipid_and_fatty-acid_transport_n11
110: pCIN5-other_transport_facilitators_n5 pINO4-other_transport_facilitators_n5
110: pINO4-g-proteins_n11 pSKN7-other_mrna-transcription_activities_n20
010: pINO4-g-proteins_n11
001: pINO4-g-proteins_n11
010: pSKN7-other_mrna-transcription_activities_n20
100: pINO4-g-proteins_n11
110: pMTH1-pentose-phosphate_pathway_n14 pMTH1-glyoxylate_cycle_n8
010: pMTH1-glyoxylate_cycle_n8
001: pMTH1-glyoxylate_cycle_n8
010: pMTH1-pentose-phosphate_pathway_n14
100: pMTH1-pentose-phosphate_pathway_n14
110: pSKN7-lipid_and_fatty-acid_transport_n11 pSKN7-other_mrna-transcription_activities_n20
010: pSKN7-lipid_and_fatty-acid_transport_n11
100: pSKN7-lipid_and_fatty-acid_transport_n11
010: pSKN7-other_mrna-transcription_activities_n20
110: pINO4-glyoxylate_cycle_n8 pINO4-g-proteins_n12
010: pINO4-glyoxylate_cycle_n8
110: pINO4-glyoxylate_cycle_n8 pXBP1-other_mrna-transcription_activities_n20
010: pINO4-glyoxylate_cycle_n8
100: pXBP1-other_mrna-transcription_activities_n20
001: pXBP1-other_mrna-transcription_activities_n20
110: pINO4-other_signal-transduction_activities_n8 pXBP1-other_mrna-transcription_activities_n20
100: pINO4-other_signal-transduction_activities_n8
100: pXBP1-other_mrna-transcription_activities_n20
001: pXBP1-other_mrna-transcription_activities_n20
110: pINO4-other_transport_facilitators_n5 pINO4-g-proteins_n11

010: pINO4-g-proteins_n11
001: pINO4-g-proteins_n11
100: pINO4-g-proteins_n11
110: pINO4-other_signal-transduction_activities_n8 pINO4-glyoxylate_cycle_n8
100: pINO4-other_signal-transduction_activities_n8
010: pINO4-glyoxylate_cycle_n8
110: pMTH1-deoxyribonucleotide_metabolisn23 pSWI6-SWI5
010: pMTH1-deoxyribonucleotide_metabolisn23
100: pSWI6-SWI5
100: pMTH1-deoxyribonucleotide_metabolisn23
110: pINO4-glyoxylate_cycle_n8 pINO4-g-proteins_n12 pINO4-SWI5
100: pINO4-g-proteins_n12 pINO4-SWI5
010: pINO4-glyoxylate_cycle_n8
010: pINO4-g-proteins_n12 pINO4-SWI5
010: pINO4-glyoxylate_cycle_n8 pINO4-g-proteins_n12
001: pINO4-g-proteins_n12 pINO4-SWI5
110: pSKN7-g-proteins_n12 pINO4-SWI5
110: pINO4-other_transport_facilitators_n5 pINO4-SWI5
110: pSKN7-SWI5 pINO4-g-proteins_n12
010: pSKN7-SWI5
100: pSKN7-SWI5
110: pCIN5-other_transport_facilitators_n5 pINO4-g-proteins_n11
010: pINO4-g-proteins_n11
001: pINO4-g-proteins_n11
100: pINO4-g-proteins_n11
110: pCIN5-other_transport_facilitators_n5 pSWI6-SWI5
100: pSWI6-SWI5
110: pSKN7-other_mrna-transcription_activities_n20 pINO4-osmosensing_n6
010: pINO4-osmosensing_n6
010: pSKN7-other_mrna-transcription_activities_n20
100: pINO4-osmosensing_n6
110: pSKN7-amino-acid_transporters_n11 pINO4-g-proteins_n12
010: pSKN7-amino-acid_transporters_n11
110: pSWI6-other_transport_facilitators_n5 pSWI6-SWI5
100: pSWI6-SWI5
010: pSWI6-other_transport_facilitators_n5
110: pXBP1-other_mrna-transcription_activities_n20 pINO4-g-proteins_n12
100: pXBP1-other_mrna-transcription_activities_n20
001: pXBP1-other_mrna-transcription_activities_n20
110: pSKN7-g-proteins_n12 pINO4-anion_transporters_n15
100: pINO4-anion_transporters_n15
010: pINO4-anion_transporters_n15
110: pSWI6-MCB pINO4-other_signal-transduction_activities_n8
100: pINO4-other_signal-transduction_activities_n8
110: pSKN7-g-proteins_n12 pSKN7-nitrogen_and_sulphur_metabolisn17
110: pSKN7-g-proteins_n12 pINO4-osmosensing_n6
010: pINO4-osmosensing_n6
100: pINO4-osmosensing_n6
110: pSKN7-g-proteins_n12 pSKN7-SWI5 pSWI6-SWI5
010: pSKN7-SWI5
100: pSWI6-SWI5
100: pSKN7-SWI5
010: pSKN7-g-proteins_n12 pSKN7-SWI5
100: pSKN7-SWI5 pSWI6-SWI5
010: pSKN7-g-proteins_n12 pSWI6-SWI5
010: pSKN7-SWI5 pSWI6-SWI5
110: pMTH1-lipid_and_fatty-acid_transport_n11 pSKN7-lipid_and_fatty-acid_transport_n11
010: pSKN7-lipid_and_fatty-acid_transport_n11
100: pMTH1-lipid_and_fatty-acid_transport_n11
100: pSKN7-lipid_and_fatty-acid_transport_n11
110: pSKN7-nitrogen_and_sulphur_metabolisn17 pINO4-osmosensing_n6
010: pINO4-osmosensing_n6
100: pINO4-osmosensing_n6
110: pSKN7-SWI5 pSKN7-other_energy_generation_activities_n16
010: pSKN7-SWI5
100: pSKN7-SWI5
110: pINO4-organization_of_plasma_membrane_n17 pMTH1-deoxyribonucleotide_metabolisn23
100: pINO4-organization_of_plasma_membrane_n17
010: pMTH1-deoxyribonucleotide_metabolisn23
100: pMTH1-deoxyribonucleotide_metabolisn23

110: pSKN7-lipid_and_fatty-acid_transport_n11 pSKN7-nitrogen_and_sulphur_metabolisn17
010: pSKN7-lipid_and_fatty-acid_transport_n11
100: pSKN7-lipid_and_fatty-acid_transport_n11
110: pSWI6-other_transport_facilitators_n5 pINO4-SWI5
010: pSWI6-other_transport_facilitators_n5
110: pINO4-glyoxylate_cycle_n8 pINO4-osmosensing_n6
010: pINO4-osmosensing_n6
010: pINO4-glyoxylate_cycle_n8
100: pINO4-osmosensing_n6
110: pMTH1-pentose-phosphate_pathway_n14 pINO4-SWI5
010: pMTH1-pentose-phosphate_pathway_n14
100: pMTH1-pentose-phosphate_pathway_n14
110: pCIN5-other_transport_facilitators_n5 pMTH1-pentose-phosphate_pathway_n14
010: pMTH1-pentose-phosphate_pathway_n14
100: pMTH1-pentose-phosphate_pathway_n14
110: pINO4-other_mrna-transcription_activities_n20 pSKN7-other_mrna-transcription_activities_n20
010: pINO4-other_mrna-transcription_activities_n20
010: pSKN7-other_mrna-transcription_activities_n20
110: pINO4-glyoxylate_cycle_n8 pSWI6-SWI5
010: pINO4-glyoxylate_cycle_n8
100: pSWI6-SWI5
110: pSKN7-phosphate_transport_n13 pMTH1-pentose-phosphate_pathway_n14
010: pSKN7-phosphate_transport_n13
010: pMTH1-pentose-phosphate_pathway_n14
100: pMTH1-pentose-phosphate_pathway_n14
100: pSKN7-phosphate_transport_n13
110: pSKN7-SWI5 pINO4-glyoxylate_cycle_n8 pINO4-SWI5
010: pSKN7-SWI5
010: pINO4-glyoxylate_cycle_n8
100: pSKN7-SWI5
010: pSKN7-SWI5 pINO4-SWI5
100: pSKN7-SWI5 pINO4-SWI5
110: pSWI6-amino-acid_metabolisn25 pXBP1-nitrogen_and_sulphur_metabolisn17
100: pXBP1-nitrogen_and_sulphur_metabolisn17
110: pSKN7-g-proteins_n12 pINO4-g-proteins_n12
110: pINO4-g-proteins_n11 pINO4-SWI5
010: pINO4-g-proteins_n11
001: pINO4-g-proteins_n11
100: pINO4-g-proteins_n11
110: pMTH1-lipid_and_fatty-acid_transport_n11 pSWI6-allantoin_and_allantoate_transporters_n13
100: pMTH1-lipid_and_fatty-acid_transport_n11
110: pCIN5-other_transport_facilitators_n5 pINO4-other_mrna-transcription_activities_n20
010: pINO4-other_mrna-transcription_activities_n20
110: pMTH1-lipid_and_fatty-acid_transport_n11 pSKN7-nitrogen_and_sulphur_metabolisn17
100: pMTH1-lipid_and_fatty-acid_transport_n11
110: pINO4-other_signal-transduction_activities_n8 pINO4-g-proteins_n12
100: pINO4-other_signal-transduction_activities_n8
110: pSWI6-other_transport_facilitators_n5 pMTH1-pentose-phosphate_pathway_n14
010: pMTH1-pentose-phosphate_pathway_n14
100: pMTH1-pentose-phosphate_pathway_n14
010: pSWI6-other_transport_facilitators_n5
110: pSKN7-g-proteins_n12 pSWI6-SWI5
100: pSWI6-SWI5
110: pSKN7-g-proteins_n12 pINO4-glyoxylate_cycle_n8
010: pINO4-glyoxylate_cycle_n8
110: pXBP1-nitrogen_and_sulphur_metabolisn17 pSKN7-nitrogen_and_sulphur_metabolisn17
100: pXBP1-nitrogen_and_sulphur_metabolisn17
110: pSKN7-SWI5 pSWI6-nucleotide_transport_n9
010: pSKN7-SWI5
100: pSWI6-nucleotide_transport_n9
100: pSKN7-SWI5
110: pCIN5-other_transport_facilitators_n5 pINO4-SWI5
110: pINO4-glyoxylate_cycle_n8 pSKN7-amino-acid_transporters_n11
010: pINO4-glyoxylate_cycle_n8
010: pSKN7-amino-acid_transporters_n11
110: pMTH1-lipid_and_fatty-acid_transport_n11 pINO4-other_mrna-transcription_activities_n20
100: pMTH1-lipid_and_fatty-acid_transport_n11
010: pINO4-other_mrna-transcription_activities_n20
110: pSKN7-g-proteins_n12 pSWI6-amino-acid_metabolisn25
110: pMTH1-lipid_and_fatty-acid_transport_n11 pINO4-g-proteins_n11

010: pINO4-g-proteins_n11
100: pMTH1-lipid_and_fatty-acid_transport_n11
001: pINO4-g-proteins_n11
100: pINO4-g-proteins_n11
110: pINO4-other_signal-transduction_activities_n8 pINO4-glyoxylate_cycle_n8 pINO4-other_mrna-transcription_activities_n20
100: pINO4-other_signal-transduction_activities_n8
010: pINO4-glyoxylate_cycle_n8
010: pINO4-other_mrna-transcription_activities_n20
110: pINO4-glyoxylate_cycle_n8 pINO4-other_mrna-transcription_activities_n20 pINO4-g-proteins_n11
010: pINO4-other_mrna-transcription_activities_n20 pINO4-g-proteins_n11
010: pINO4-g-proteins_n11
010: pINO4-glyoxylate_cycle_n8
001: pINO4-g-proteins_n11
010: pINO4-other_mrna-transcription_activities_n20
100: pINO4-g-proteins_n11
110: pINO4-homeostasis_of_other_ions_n30 pINO4-glyoxylate_cycle_n8 pINO4-other_mrna-transcription_activities_n20
010: pINO4-homeostasis_of_other_ions_n30 pINO4-glyoxylate_cycle_n8
100: pINO4-homeostasis_of_other_ions_n30
010: pINO4-glyoxylate_cycle_n8
010: pINO4-other_mrna-transcription_activities_n20
010: pINO4-homeostasis_of_other_ions_n30
110: pINO4-other_signal-transduction_activities_n8 pINO4-phosphate_transport_n18
100: pINO4-other_signal-transduction_activities_n8
100: pINO4-phosphate_transport_n18
010: pINO4-phosphate_transport_n18
110: pINO4-phosphate_transport_n18 pINO4-glyoxylate_cycle_n8
100: pINO4-phosphate_transport_n18
010: pINO4-glyoxylate_cycle_n8
010: pINO4-phosphate_transport_n18
110: pINO4-other_signal-transduction_activities_n8 pINO4-homeostasis_of_other_ions_n30
100: pINO4-other_signal-transduction_activities_n8
100: pINO4-homeostasis_of_other_ions_n30
010: pINO4-homeostasis_of_other_ions_n30
110: pINO4-homeostasis_of_other_ions_n30 pINO4-glyoxylate_cycle_n8
100: pINO4-homeostasis_of_other_ions_n30
010: pINO4-glyoxylate_cycle_n8
010: pINO4-homeostasis_of_other_ions_n30
110: pINO4-other_mrna-transcription_activities_n20 pINO4-g-proteins_n11 pINO4-g-proteins_n12
010: pINO4-other_mrna-transcription_activities_n20 pINO4-g-proteins_n11
010: pINO4-g-proteins_n11
100: pINO4-g-proteins_n11 pINO4-g-proteins_n12
001: pINO4-g-proteins_n11
010: pINO4-other_mrna-transcription_activities_n20 pINO4-g-proteins_n12
010: pINO4-other_mrna-transcription_activities_n20
001: pINO4-g-proteins_n11 pINO4-g-proteins_n12
001: pINO4-other_mrna-transcription_activities_n20 pINO4-g-proteins_n12
010: pINO4-g-proteins_n11 pINO4-g-proteins_n12
100: pINO4-g-proteins_n11
110: pINO4-homeostasis_of_other_ions_n30 pINO4-phosphate_transport_n18 pINO4-other_mrna-transcription_activities_n20
100: pINO4-homeostasis_of_other_ions_n30 pINO4-phosphate_transport_n18
100: pINO4-homeostasis_of_other_ions_n30
100: pINO4-phosphate_transport_n18
010: pINO4-other_mrna-transcription_activities_n20
010: pINO4-homeostasis_of_other_ions_n30
010: pINO4-phosphate_transport_n18
110: pINO4-phosphate_transport_n18 pINO4-g-proteins_n11
010: pINO4-g-proteins_n11
100: pINO4-phosphate_transport_n18
001: pINO4-g-proteins_n11
010: pINO4-phosphate_transport_n18
100: pINO4-g-proteins_n11
110: pINO4-homeostasis_of_other_ions_n30 pINO4-other_mrna-transcription_activities_n20
100: pINO4-homeostasis_of_other_ions_n30
010: pINO4-other_mrna-transcription_activities_n20
010: pINO4-homeostasis_of_other_ions_n30
110: pINO4-other_mrna-transcription_activities_n20 pINO4-g-proteins_n12
010: pINO4-other_mrna-transcription_activities_n20
110: pINO4-homeostasis_of_other_ions_n30 pINO4-phosphate_transport_n18
100: pINO4-homeostasis_of_other_ions_n30
100: pINO4-phosphate_transport_n18

010: pINO4-homeostasis_of_other_ions_n30
010: pINO4-phosphate_transport_n18
110: pINO4-glyoxylate_cycle_n8 pINO4-g-proteins_n11 pINO4-g-proteins_n12
010: pINO4-g-proteins_n11
100: pINO4-g-proteins_n11 pINO4-g-proteins_n12
010: pINO4-glyoxylate_cycle_n8
001: pINO4-g-proteins_n11
010: pINO4-glyoxylate_cycle_n8 pINO4-g-proteins_n12
001: pINO4-g-proteins_n11 pINO4-g-proteins_n12
010: pINO4-g-proteins_n11 pINO4-g-proteins_n12
100: pINO4-g-proteins_n11
110: pINO4-other_signal-transduction_activities_n8 pINO4-homeostasis_of_other_ions_n30 pINO4-glyoxylate_cycle_n8
100: pINO4-other_signal-transduction_activities_n8
010: pINO4-homeostasis_of_other_ions_n30 pINO4-glyoxylate_cycle_n8
100: pINO4-homeostasis_of_other_ions_n30
010: pINO4-glyoxylate_cycle_n8
010: pINO4-homeostasis_of_other_ions_n30
110: pINO4-other_signal-transduction_activities_n8 pINO4-glyoxylate_cycle_n8 pINO4-g-proteins_n11
100: pINO4-other_signal-transduction_activities_n8
010: pINO4-g-proteins_n11
010: pINO4-glyoxylate_cycle_n8
001: pINO4-g-proteins_n11
100: pINO4-g-proteins_n11
110: pINO4-other_signal-transduction_activities_n8 pINO4-homeostasis_of_other_ions_n30 pINO4-phosphate_transport_n18
100: pINO4-other_signal-transduction_activities_n8
100: pINO4-homeostasis_of_other_ions_n30 pINO4-phosphate_transport_n18
100: pINO4-homeostasis_of_other_ions_n30
100: pINO4-phosphate_transport_n18
010: pINO4-homeostasis_of_other_ions_n30
010: pINO4-phosphate_transport_n18
110: pINO4-other_signal-transduction_activities_n8 pINO4-phosphate_transport_n18 pINO4-glyoxylate_cycle_n8
100: pINO4-other_signal-transduction_activities_n8
100: pINO4-phosphate_transport_n18
010: pINO4-glyoxylate_cycle_n8
010: pINO4-phosphate_transport_n18
110: pINO4-g-proteins_n11 pXBP1-other_mrna-transcription_activities_n20 pINO4-g-proteins_n12
010: pINO4-g-proteins_n11
100: pINO4-g-proteins_n11 pINO4-g-proteins_n12
001: pINO4-g-proteins_n11
100: pXBP1-other_mrna-transcription_activities_n20
001: pINO4-g-proteins_n11 pINO4-g-proteins_n12
001: pXBP1-other_mrna-transcription_activities_n20
010: pINO4-g-proteins_n11 pINO4-g-proteins_n12
100: pINO4-g-proteins_n11
110: pINO4-phosphate_transport_n18 pINO4-glyoxylate_cycle_n8 pINO4-other_mrna-transcription_activities_n20
100: pINO4-phosphate_transport_n18
010: pINO4-glyoxylate_cycle_n8
010: pINO4-other_mrna-transcription_activities_n20
010: pINO4-phosphate_transport_n18
110: pINO4-organization_of_plasma_membrane_n17 pINO4-SWI5
100: pINO4-organization_of_plasma_membrane_n17
110: pMTH1-other_energy_generation_activities_n12 pSWI4-deoxyribonucleotide_metabolisn23
110: pMTH1-deoxyribonucleotide_metabolisn23 pSWI4-deoxyribonucleotide_metabolisn23
010: pMTH1-deoxyribonucleotide_metabolisn23
100: pMTH1-deoxyribonucleotide_metabolisn23
110: pMTH1-other_energy_generation_activities_n12 pMTH1-deoxyribonucleotide_metabolisn23
010: pMTH1-deoxyribonucleotide_metabolisn23
100: pMTH1-deoxyribonucleotide_metabolisn23
110: pFKH2-other_energy_generation_activities_n9 pFKH2-fermentation_n4
110: pNDD1-MCM1' pYOX1-MCM1 pFKH2-SFF
100: pNDD1-MCM1' pFKH2-SFF
100: pNDD1-MCM1'
010: pYOX1-MCM1
110: pYOX1-MCM1 pFKH1-SFF' pFKH2-SFF
100: pFKH1-SFF' pFKH2-SFF
010: pYOX1-MCM1
110: pNDD1-MCM1 pYOX1-MCM1 pFKH2-SFF
100: pNDD1-MCM1
010: pYOX1-MCM1
110: pMCM1-MCM1 pYOX1-MCM1 pFKH2-SFF

100: pMCM1-MCM1
010: pMCM1-MCM1
010: pYOX1-MCM1
110: pMCM1-SFF' pYOX1-MCM1 pFKH2-SFF
100: pMCM1-SFF' pFKH2-SFF
010: pYOX1-MCM1
110: pMCM1-MCM1' pYOX1-MCM1 pFKH2-SFF
100: pMCM1-MCM1' pFKH2-SFF
100: pMCM1-MCM1'
010: pYOX1-MCM1
110: pMCM1-other_morphogenetic_activities_n7 pFKH2-SFF
100: pMCM1-other_morphogenetic_activities_n7
110: pMCM1-other_morphogenetic_activities_n7 pFKH1-SFF
100: pMCM1-other_morphogenetic_activities_n7
110: pNDD1-MCM1' pFKH1-SFF' pFKH2-SFF
100: pFKH1-SFF' pFKH2-SFF
100: pNDD1-MCM1' pFKH1-SFF'
100: pNDD1-MCM1' pFKH2-SFF
100: pNDD1-MCM1'
010: pNDD1-MCM1' pFKH1-SFF'
110: pFKH1-SFF' pFKH2-MCM1 pFKH2-SFF
100: pFKH2-MCM1
100: pFKH1-SFF' pFKH2-SFF
010: pFKH2-MCM1
100: pFKH1-SFF' pFKH2-MCM1
110: pNDD1-MCM1' pFKH2-MCM1 pFKH2-SFF
100: pFKH2-MCM1
100: pNDD1-MCM1' pFKH2-SFF
100: pNDD1-MCM1'
010: pFKH2-MCM1
110: pNDD1-MCM1 pFKH2-SFF
100: pNDD1-MCM1
110: pNDD1-MCM1 pFKH1-SFF
100: pNDD1-MCM1
110: pFKH2-MCM1 pFKH1-SFF
100: pFKH2-MCM1
010: pFKH2-MCM1
110: pNDD1-MCM1 pTEC1-ECB
100: pNDD1-MCM1
110: pNDD1-MCM1 pSTE12-ECB
100: pNDD1-MCM1
110: pRLM1-cell_death_n15 pNDD1-MCM1'
100: pNDD1-MCM1'
110: pSWI4-MCM1 pSTE12-ECB
100: pSWI4-MCM1
110: pTEC1-ECB pFKH1-SFF
110: pNDD1-MCM1' pTEC1-ECB
100: pNDD1-MCM1'
110: pSTE12-ECB pFKH2-MCM1
100: pFKH2-MCM1
010: pFKH2-MCM1
110: pSTE12-ECB pFKH2-SFF
110: pRLM1-cell_death_n15 pFKH2-ECB
110: pNDD1-MCM1' pSTE12-ECB
100: pNDD1-MCM1'
110: pSWI4-MCM1 pTEC1-ECB
100: pSWI4-MCM1
110: pRLM1-cell_death_n15 pMCM1-ECB
100: pMCM1-ECB
010: pMCM1-ECB
110: pFKH2-ECB pTEC1-ECB
110: pTEC1-ECB pFKH2-MCM1
100: pFKH2-MCM1
010: pFKH2-MCM1
110: pSWI4-MCM1' pTEC1-ECB
100: pSWI4-MCM1'
010: pSWI4-MCM1'
110: pRLM1-cell_death_n15 pNDD1-ECB
110: pTEC1-ECB pFKH2-SFF
110: pTEC1-ECB pNDD1-ECB

110: pSWI4-MCM1' pSTE12-ECB
100: pSWI4-MCM1'
010: pSWI4-MCM1'
110: pMCM1-MCM1' pRLM1-cell_death_n15
100: pMCM1-MCM1'
110: pMCM1-MCM1' pSWI4-MCM1' pNDD1-ECB
100: pSWI4-MCM1'
100: pMCM1-MCM1' pSWI4-MCM1'
100: pMCM1-MCM1'
010: pSWI4-MCM1'
110: pSWI4-MCM1 pYOX1-MCM1
100: pSWI4-MCM1
010: pYOX1-MCM1
110: pMCM1-ECB pSTE12-ECB
100: pMCM1-ECB
010: pMCM1-ECB
110: pSTE12-ECB pNDD1-ECB
110: pFKH2-ECB pSTE12-ECB
110: pMCM1-MCM1' pSTE12-ECB
100: pMCM1-MCM1'
110: pYOX1-ECB pSWI4-MCM1
100: pSWI4-MCM1
010: pYOX1-ECB
110: pMCM1-MCM1 pSTE12-ECB
100: pMCM1-MCM1
010: pMCM1-MCM1
110: pSTE12-ECB pFKH1-SFF
110: pMCM1-MCM1' pSWI4-MCM1' pNDD1-MCM1'
100: pSWI4-MCM1'
100: pMCM1-MCM1' pNDD1-MCM1'
100: pSWI4-MCM1' pNDD1-MCM1'
100: pNDD1-MCM1'
100: pMCM1-MCM1' pSWI4-MCM1'
100: pMCM1-MCM1'
010: pSWI4-MCM1'
110: pSWI4-MCM1' pNDD1-MCM1' pFKH2-SFF
100: pSWI4-MCM1'
100: pNDD1-MCM1' pFKH2-SFF
100: pSWI4-MCM1' pNDD1-MCM1'
100: pNDD1-MCM1'
010: pSWI4-MCM1'
100: pSWI4-MCM1' pFKH2-SFF
110: pMCM1-MCM1' pSWI4-MCM1' pFKH2-SFF
100: pSWI4-MCM1'
100: pMCM1-MCM1' pFKH2-SFF
100: pMCM1-MCM1' pSWI4-MCM1'
100: pMCM1-MCM1'
010: pSWI4-MCM1'
100: pSWI4-MCM1' pFKH2-SFF
110: pSWI4-MCM1' pNDD1-MCM1' pNDD1-ECB
100: pSWI4-MCM1'
100: pSWI4-MCM1' pNDD1-MCM1'
100: pNDD1-MCM1'
010: pSWI4-MCM1'
110: pSWI4-MCM1' pFKH2-MCM1 pFKH2-SFF
100: pSWI4-MCM1'
100: pFKH2-MCM1
010: pFKH2-MCM1
010: pSWI4-MCM1'
100: pSWI4-MCM1' pFKH2-SFF
110: pSWI4-MCM1' pNDD1-MCM1
100: pSWI4-MCM1'
100: pNDD1-MCM1
010: pSWI4-MCM1'
110: pSWI4-MCM1' pFKH2-ECB
100: pSWI4-MCM1'
010: pSWI4-MCM1'
110: pSWI4-MCM1' pNDD1-MCM1'
100: pSWI4-MCM1'
100: pNDD1-MCM1'

010: pSWI4-MCM1'
110: pMCM1-MCM1' pNDD1-MCM1' pMCM1-MCM1 pMCM1-ECB pFKH1-SFF
100: pNDD1-MCM1' pFKH1-SFF
100: pMCM1-MCM1' pNDD1-MCM1'
100: pNDD1-MCM1'
100: pMCM1-MCM1 pMCM1-ECB
100: pMCM1-MCM1' pMCM1-MCM1
100: pMCM1-ECB
100: pMCM1-MCM1
100: pMCM1-MCM1'
010: pMCM1-MCM1
010: pMCM1-ECB
100: pMCM1-MCM1' pFKH1-SFF
100: pMCM1-MCM1' pNDD1-MCM1' pFKH1-SFF
110: pNDD1-MCM1 pMCM1-ECB pFKH1-SFF
100: pMCM1-ECB
010: pMCM1-ECB
100: pNDD1-MCM1
110: pMCM1-ECB pFKH2-MCM1 pFKH1-SFF
100: pFKH2-MCM1
100: pMCM1-ECB
010: pMCM1-ECB
010: pFKH2-MCM1
100: pFKH2-MCM1 pFKH1-SFF
110: pFKH2-ECB pFKH1-SFF
110: pNDD1-MCM1' pMCM1-MCM1 pFKH2-ECB
100: pNDD1-MCM1'
100: pMCM1-MCM1
010: pMCM1-MCM1
110: pFKH2-ECB pFKH2-SFF
110: pHIR1-SFF pHIR1-other_energy_generation_activities_n22
010: pHIR1-SFF
100: pHIR1-SFF
110: pSWI4-MCM1' pNDD1-MCM1' pFKH1-SFF
100: pSWI4-MCM1'
100: pNDD1-MCM1' pFKH1-SFF
100: pSWI4-MCM1' pNDD1-MCM1'
100: pNDD1-MCM1'
010: pSWI4-MCM1'
100: pSWI4-MCM1' pFKH1-SFF
110: pSWI4-MCM1' pNDD1-ECB pFKH2-SFF
100: pSWI4-MCM1'
010: pSWI4-MCM1'
100: pSWI4-MCM1' pFKH2-SFF
110: pMCM1-MCM1' pSWI4-MCM1' pFKH1-SFF
100: pSWI4-MCM1'
100: pMCM1-MCM1' pSWI4-MCM1'
100: pMCM1-MCM1'
010: pSWI4-MCM1'
100: pSWI4-MCM1' pFKH1-SFF
100: pMCM1-MCM1' pFKH1-SFF
110: pMCM1-MCM1' pSWI4-MCM1' pFKH2-ECB
100: pSWI4-MCM1'
100: pMCM1-MCM1' pSWI4-MCM1'
100: pMCM1-MCM1'
010: pSWI4-MCM1'
110: pSWI4-MCM1' pNDD1-ECB
100: pSWI4-MCM1'
010: pSWI4-MCM1'
110: pSWI4-MCM1' pFKH2-MCM1
100: pSWI4-MCM1'
100: pFKH2-MCM1
010: pFKH2-MCM1
010: pSWI4-MCM1'
110: pMCM1-MCM1' pNDD1-MCM1 pMCM1-ECB pFKH1-SFF
100: pMCM1-ECB
100: pMCM1-MCM1'
010: pMCM1-ECB
100: pNDD1-MCM1
100: pMCM1-MCM1' pFKH1-SFF

110: pNDD1-MCM1' pMCM1-ECB pFKH1-SFF
100: pNDD1-MCM1' pFKH1-SFF
100: pNDD1-MCM1'
100: pMCM1-ECB
010: pMCM1-ECB
110: pMCM1-ECB pFKH1-SFF pFKH2-SFF
100: pFKH1-SFF pFKH2-SFF
100: pMCM1-ECB
010: pMCM1-ECB
110: pSWI4-MCM1' pFKH2-ECB pFKH2-SFF
100: pSWI4-MCM1'
010: pSWI4-MCM1'
100: pSWI4-MCM1' pFKH2-SFF
110: pSWI4-MCM1 pNDD1-ECB pFKH2-SFF
100: pSWI4-MCM1
110: pMCM1-MCM1' pMCM1-ECB pFKH2-MCM1 pFKH1-SFF
100: pFKH2-MCM1
100: pMCM1-ECB
100: pMCM1-MCM1'
010: pMCM1-ECB
010: pFKH2-MCM1
100: pMCM1-MCM1' pFKH1-SFF
100: pFKH2-MCM1 pFKH1-SFF
110: pSWI4-MCM1 pFKH2-ECB pFKH2-SFF
100: pSWI4-MCM1
110: pMCM1-MCM1' pNDD1-MCM1' pMCM1-ECB pFKH1-SFF
100: pNDD1-MCM1' pFKH1-SFF
100: pMCM1-MCM1' pNDD1-MCM1'
100: pNDD1-MCM1'
100: pMCM1-ECB
100: pMCM1-MCM1'
010: pMCM1-ECB
100: pMCM1-MCM1' pFKH1-SFF
100: pMCM1-MCM1' pNDD1-MCM1' pFKH1-SFF
110: pMCM1-MCM1 pMCM1-ECB pFKH2-MCM1 pFKH1-SFF
100: pFKH2-MCM1
100: pMCM1-MCM1 pMCM1-ECB
100: pMCM1-ECB
100: pMCM1-MCM1
010: pMCM1-MCM1
010: pMCM1-ECB
010: pFKH2-MCM1
100: pFKH2-MCM1 pFKH1-SFF
110: pSWI6-SWI5 pGAT1-lipid_and_fatty-acid_transport_n11 pFKH1-SFF
010: pGAT1-lipid_and_fatty-acid_transport_n11
100: pSWI6-SWI5
100: pGAT1-lipid_and_fatty-acid_transport_n11
100: pSWI6-SWI5 pFKH1-SFF
110: pFKH1-SFF' pSWI6-SWI5 pGAT1-lipid_and_fatty-acid_transport_n11
010: pGAT1-lipid_and_fatty-acid_transport_n11
100: pSWI6-SWI5
100: pGAT1-lipid_and_fatty-acid_transport_n11
100: pFKH1-SFF' pSWI6-SWI5
110: pGAT1-glyoxylate_cycle_n11 pFKH1-regulation_of_lipid_fatty-acid_and_isoprenoid_biosynthesis_n8.scn pFKH1-SFF
100: pFKH1-regulation_of_lipid_fatty-acid_and_isoprenoid_biosynthesis_n8.scn pFKH1-SFF
110: pSWI6-LYS14 pMET4-pentose-phosphate_pathway_n23
010: pSWI6-LYS14
100: pMET4-pentose-phosphate_pathway_n23
110: pFKH1-regulation_of_lipid_fatty-acid_and_isoprenoid_biosynthesis_n8.scn pSWI6-LYS14
010: pSWI6-LYS14
110: pGAT1-glyoxylate_cycle_n11 pFKH1-SFF
110: pGAT1-lipid_and_fatty-acid_transport_n11 pFKH1-SFF
010: pGAT1-lipid_and_fatty-acid_transport_n11
100: pGAT1-lipid_and_fatty-acid_transport_n11
110: pFKH1-regulation_of_lipid_fatty-acid_and_isoprenoid_biosynthesis_n8.scn pSWI6-SWI5
100: pSWI6-SWI5
110: pFKH1-SFF' pGAT1-lipid_and_fatty-acid_transport_n11
010: pGAT1-lipid_and_fatty-acid_transport_n11
100: pGAT1-lipid_and_fatty-acid_transport_n11
110: pGAT1-regulation_of_lipid_fatty-acid_and_isoprenoid_biosynthesis_n8.scn pFKH1-SFF

010: pGAT1-regulation_of_lipid_fatty-acid_and_isoprenoid_biosynthesis_n8.scn
100: pGAT1-regulation_of_lipid_fatty-acid_and_isoprenoid_biosynthesis_n8.scn
110: pSWI6-SWI5 pGAT1-lipid_and_fatty-acid_transport_n11
010: pGAT1-lipid_and_fatty-acid_transport_n11
100: pSWI6-SWI5
100: pGAT1-lipid_and_fatty-acid_transport_n11
110: pFKH1-regulation_of_lipid_fatty-acid_and_isoprenoid_biosynthesis_n8.scn pFKH1-SFF
110: pGAT1-glyoxylate_cycle_n11 pGAT1-regulation_of_lipid_fatty-acid_and_isoprenoid_biosynthesis_n8.scn pFKH1-SFF'
010: pGAT1-regulation_of_lipid_fatty-acid_and_isoprenoid_biosynthesis_n8.scn
100: pGAT1-regulation_of_lipid_fatty-acid_and_isoprenoid_biosynthesis_n8.scn
010: pGAT1-glyoxylate_cycle_n11 pFKH1-SFF'
110: pGAT1-glyoxylate_cycle_n11 pFKH1-regulation_of_lipid_fatty-acid_and_isoprenoid_biosynthesis_n8.scn
110: pFKH1-regulation_of_lipid_fatty-acid_and_isoprenoid_biosynthesis_n8.scn pGAT1-regulation_of_lipid_fatty-acid_and_isoprenoid_biosynthesis_n8.scn
010: pGAT1-regulation_of_lipid_fatty-acid_and_isoprenoid_biosynthesis_n8.scn
100: pGAT1-regulation_of_lipid_fatty-acid_and_isoprenoid_biosynthesis_n8.scn
110: pGAT1-glyoxylate_cycle_n11 pFKH1-SFF'
110: pGAT1-regulation_of_lipid_fatty-acid_and_isoprenoid_biosynthesis_n8.scn pFKH1-SFF'
010: pGAT1-regulation_of_lipid_fatty-acid_and_isoprenoid_biosynthesis_n8.scn
100: pGAT1-regulation_of_lipid_fatty-acid_and_isoprenoid_biosynthesis_n8.scn
110: pSWI6-LYS14 pSWI6-SWI5
100: pSWI6-SWI5
010: pSWI6-LYS14
110: pSKN7-organization_of_chromosome_structure_n17 pSKN7-sugar_and_carbohydrate_transporters_n6
100: pSKN7-organization_of_chromosome_structure_n17
110: pSWI6-amino-acid_metabolisn25 pSKN7-nitrogen_and_sulphur_metabolisn17
110: pSKN7-organization_of_chromosome_structure_n17 pSKN7-MERE4
100: pSKN7-organization_of_chromosome_structure_n17
010: pSKN7-MERE4
110: pSKN7-organization_of_chromosome_structure_n17 pFKH1-SFF
100: pSKN7-organization_of_chromosome_structure_n17
110: pSKN7-g-proteins_n12 pSKN7-sugar_and_carbohydrate_transporters_n6
110: pFKH1-SFF pSKN7-nitrogen_and_sulphur_metabolisn17
110: pSKN7-g-proteins_n12 pSKN7-organization_of_chromosome_structure_n17
100: pSKN7-organization_of_chromosome_structure_n17
110: pNDD1-amino-acid_degradation_n7 pFKH1-SFF pFKH2-SFF
100: pFKH1-SFF pFKH2-SFF
110: pMET4-other_protein-destination_activities_n7 pSKN7-sugar_and_carbohydrate_transporters_n6
100: pMET4-other_protein-destination_activities_n7
010: pMET4-other_protein-destination_activities_n7
110: pSKN7-other_protein-destination_activities_n7 pSKN7-sugar_and_carbohydrate_transporters_n6
100: pSKN7-other_protein-destination_activities_n7
110: pSKN7-g-proteins_n12 pSKN7-other_protein-destination_activities_n7
100: pSKN7-other_protein-destination_activities_n7
110: pSKN7-g-proteins_n12 pSKN7-amino-acid_degradation_n7
110: pNDD1-amino-acid_degradation_n7 pFKH2-SFF
110: pSKN7-g-proteins_n12 pNDD1-amino-acid_degradation_n7
110: pSKN7-g-proteins_n12 pSKN7-lipid_and_fatty-acid_transport_n7
010: pSKN7-lipid_and_fatty-acid_transport_n7
110: pSKN7-g-proteins_n12 pMET4-other_protein-destination_activities_n7
100: pMET4-other_protein-destination_activities_n7
010: pMET4-other_protein-destination_activities_n7
110: pSKN7-MERE4 pFKH1-SFF
010: pSKN7-MERE4
101: pSWI6-nutritional_response_pathway_n7 pSWI6-SWI5
010: pSWI6-nutritional_response_pathway_n7
100: pSWI6-SWI5
110: pPHO4-cell_rescue_defense_cell_death_and_ageing_n20 pACE2-cytoskeleton-dependenttransport_n4
110: pSWI6-trna_transcription_n10 pSWI4-phosphate_transport_n8
110: pPIP2-other_transport_facilitators_n10 pSWI6-breakdown_of_lipids_fatty_acids_and_isoprenoids_n8
110: pPIP2-other_transport_facilitators_n10 pMBP1-cytoskeleton-dependenttransport_n4
110: pPHO4-allantoin_and_allantoate_transporters_n6 pFKH2-SFF
110: pPIP2-metabolisof_energy_reserves_n8 pFKH2-SFF
110: pSWI4-phosphate_transport_n8 pMBP1-cytoskeleton-dependenttransport_n4
110: pPIP2-metabolisof_energy_reserves_n8 pMBP1-cytoskeleton-dependenttransport_n4
110: pSWI6-trna_transcription_n10 pFKH2-SFF
110: pPIP2-metabolisof_energy_reserves_n8 pSWI6-breakdown_of_lipids_fatty_acids_and_isoprenoids_n8
110: pSWI6-trna_transcription_n10 pMBP1-breakdown_of_lipids_fatty_acids_and_isoprenoids_n8
110: pSWI6-breakdown_of_lipids_fatty_acids_and_isoprenoids_n8 pPHO4-cell_rescue_defense_cell_death_and_ageing_n20
110: pPIP2-other_transport_facilitators_n10 pSWI4-phosphate_transport_n8

110: pPIP2-metabolisof_energy_reserves_n8 pPHO4-allantoin_and_allantoate_transporters_n6
110: pSWI6-trna_transcription_n10 pPIP2-other_transport_facilitators_n10
110: pOAF1-other_transport_facilitators_n10 pPHO4-cell_rescue_defense_cell_death_and_ageing_n20
110: pSWI4-phosphate_transport_n8 pSTB1-cytoskeleton-dependenttransport_n4
110: pSWI4-phosphate_transport_n8 pPIP2-metabolisof_energy_reserves_n8
110: pSWI6-trna_transcription_n10 pACE2-cytoskeleton-dependenttransport_n4
110: pPIP2-other_transport_facilitators_n10 pPHO4-allantoin_and_allantoate_transporters_n6
110: pOAF1-other_transport_facilitators_n10 pSTB1-cytoskeleton-dependenttransport_n4
110: pACE2-cytoskeleton-dependenttransport_n4 pACE2-cell_rescue_defense_cell_death_and_ageing_n20
110: pPIP2-other_transport_facilitators_n10 pMBP1-breakdown_of_lipids_fatty_acids_and_isoprenoids_n8
110: pSWI6-trna_transcription_n10 pSTB1-cytoskeleton-dependenttransport_n4
110: pSWI6-trna_transcription_n10 pPHO4-allantoin_and_allantoate_transporters_n6
110: pOAF1-allantoin_and_allantoate_transporters_n12 pFKH2-SFF
100: pOAF1-allantoin_and_allantoate_transporters_n12
010: pOAF1-allantoin_and_allantoate_transporters_n12
110: pPIP2-other_transport_facilitators_n10 pACE2-cytoskeleton-dependenttransport_n4
110: pMBP1-cytoskeleton-dependenttransport_n4 pACE2-cell_rescue_defense_cell_death_and_ageing_n20
110: pSWI6-SWI5 pACE2-cell_rescue_defense_cell_death_and_ageing_n20
100: pSWI6-SWI5
110: pOAF1-allantoin_and_allantoate_transporters_n12 pACE2-allantoin_and_allantoate_transporters_n7
100: pOAF1-allantoin_and_allantoate_transporters_n12
010: pOAF1-allantoin_and_allantoate_transporters_n12
110: pPHO4-allantoin_and_allantoate_transporters_n6 pSWI6-other_transport_facilitators_n10
010: pSWI6-other_transport_facilitators_n10
110: pSTB1-cytoskeleton-dependenttransport_n4 pACE2-cytoskeleton-dependenttransport_n4
110: pSWI6-trna_transcription_n10 pACE2-cell_rescue_defense_cell_death_and_ageing_n20
110: pSWI6-trna_transcription_n10 pOAF1-allantoin_and_allantoate_transporters_n12
100: pOAF1-allantoin_and_allantoate_transporters_n12
010: pOAF1-allantoin_and_allantoate_transporters_n12
110: pPIP2-other_transport_facilitators_n10 pACE2-allantoin_and_allantoate_transporters_n7
110: pPHO4-allantoin_and_allantoate_transporters_n6 pACE2-cell_rescue_defense_cell_death_and_ageing_n20
110: pOAF1-allantoin_and_allantoate_transporters_n12 pFKH2-SFF'
100: pOAF1-allantoin_and_allantoate_transporters_n12
010: pOAF1-allantoin_and_allantoate_transporters_n12
110: pPHO4-allantoin_and_allantoate_transporters_n6 pSTB1-cytoskeleton-dependenttransport_n4
110: pSWI6-breakdown_of_lipids_fatty_acids_and_isoprenoids_n8 pOAF1-other_transport_facilitators_n10
110: pPHO4-allantoin_and_allantoate_transporters_n6 pFKH2-SFF'
110: pSWI6-trna_transcription_n10 pACE2-allantoin_and_allantoate_transporters_n7
110: pOAF1-allantoin_and_allantoate_transporters_n12 pACE2-allantoin_and_allantoate_transporters_n12
100: pOAF1-allantoin_and_allantoate_transporters_n12
010: pOAF1-allantoin_and_allantoate_transporters_n12
110: pSWI4-phosphate_transport_n8 pACE2-allantoin_and_allantoate_transporters_n12
110: pSWI4-phosphate_transport_n8 pPHO4-allantoin_and_allantoate_transporters_n6
110: pSTB1-cytoskeleton-dependenttransport_n4 pFKH2-SFF'
110: pMBP1-breakdown_of_lipids_fatty_acids_and_isoprenoids_n8 pPHO4-cell_rescue_defense_cell_death_and_ageing_n20
110: pPIP2-metabolisof_energy_reserves_n8 pACE2-cell_rescue_defense_cell_death_and_ageing_n20
110: pSWI4-phosphate_transport_n8 pOAF1-allantoin_and_allantoate_transporters_n12
100: pOAF1-allantoin_and_allantoate_transporters_n12
010: pOAF1-allantoin_and_allantoate_transporters_n12
110: pMBP1-cytoskeleton-dependenttransport_n4 pSWI6-other_transport_facilitators_n10
010: pSWI6-other_transport_facilitators_n10
110: pACE2-cell_rescue_defense_cell_death_and_ageing_n20 pACE2-allantoin_and_allantoate_transporters_n12
110: pOAF1-allantoin_and_allantoate_transporters_n12 pSWI6-SWI5
100: pSWI6-SWI5
100: pOAF1-allantoin_and_allantoate_transporters_n12
010: pOAF1-allantoin_and_allantoate_transporters_n12
110: pMBP1-breakdown_of_lipids_fatty_acids_and_isoprenoids_n8 pACE2-cell_rescue_defense_cell_death_and_ageing_n20
110: pSTB1-cytoskeleton-dependenttransport_n4 pFKH2-SFF
110: pSWI6-breakdown_of_lipids_fatty_acids_and_isoprenoids_n8 pACE2-cell_rescue_defense_cell_death_and_ageing_n20
110: pSTB1-cytoskeleton-dependenttransport_n4 pSWI6-other_transport_facilitators_n10
010: pSWI6-other_transport_facilitators_n10
110: pMBP1-breakdown_of_lipids_fatty_acids_and_isoprenoids_n8 pSTB1-cytoskeleton-dependenttransport_n4
110: pPIP2-metabolisof_energy_reserves_n8 pSTB1-cytoskeleton-dependenttransport_n4
110: pSWI6-trna_transcription_n10 pSWI6-breakdown_of_lipids_fatty_acids_and_isoprenoids_n8
110: pSTB1-cytoskeleton-dependenttransport_n4 pSWI6-SWI5
100: pSWI6-SWI5
110: pOAF1-allantoin_and_allantoate_transporters_n12 pACE2-cell_rescue_defense_cell_death_and_ageing_n20
100: pOAF1-allantoin_and_allantoate_transporters_n12
010: pOAF1-allantoin_and_allantoate_transporters_n12
110: pFKH2-SFF' pACE2-cell_rescue_defense_cell_death_and_ageing_n20

110: pACE2-allantoin_and_allantoate_transporters_n7 pACE2-cell_rescue_defense_cell_death_and_ageing_n20
110: pSWI4-phosphate_transport_n8 pPHO4-cell_rescue_defense_cell_death_and_ageing_n20
110: pSWI4-phosphate_transport_n8 pACE2-cell_rescue_defense_cell_death_and_ageing_n20
110: pSWI6-other_transport_facilitators_n10 pPHO4-cell_rescue_defense_cell_death_and_ageing_n20
010: pSWI6-other_transport_facilitators_n10
110: pSWI6-other_transport_facilitators_n10 pACE2-cell_rescue_defense_cell_death_and_ageing_n20
010: pSWI6-other_transport_facilitators_n10
110: pOAF1-allantoin_and_allantoate_transporters_n12 pSTB1-cytoskeleton-dependenttransport_n4
100: pOAF1-allantoin_and_allantoate_transporters_n12
010: pOAF1-allantoin_and_allantoate_transporters_n12
110: pSTB1-cytoskeleton-dependenttransport_n4 pACE2-cell_rescue_defense_cell_death_and_ageing_n20
110: pPIP2-metabolisof_energy_reserves_n8 pACE2-allantoin_and_allantoate_transporters_n12
110: pPIP2-other_transport_facilitators_n10 pPHO4-cell_rescue_defense_cell_death_and_ageing_n20
110: pPHO4-cell_rescue_defense_cell_death_and_ageing_n20 pACE2-allantoin_and_allantoate_transporters_n12
110: pPHO4-allantoin_and_allantoate_transporters_n6 pPHO4-cell_rescue_defense_cell_death_and_ageing_n20
110: pACE2-cell_rescue_defense_cell_death_and_ageing_n20 pFKH2-SFF
110: pMBP1-breakdown_of_lipids_fatty_acids_and_isoprenoids_n8 pOAF1-allantoin_and_allantoate_transporters_n12
100: pOAF1-allantoin_and_allantoate_transporters_n12
010: pOAF1-allantoin_and_allantoate_transporters_n12
110: pSWI6-breakdown_of_lipids_fatty_acids_and_isoprenoids_n8 pOAF1-allantoin_and_allantoate_transporters_n12
100: pOAF1-allantoin_and_allantoate_transporters_n12
010: pOAF1-allantoin_and_allantoate_transporters_n12
110: pSWI4-phosphate_transport_n8 pOAF1-other_transport_facilitators_n10
110: pPHO4-allantoin_and_allantoate_transporters_n6 pOAF1-allantoin_and_allantoate_transporters_n12
100: pOAF1-allantoin_and_allantoate_transporters_n12
010: pOAF1-allantoin_and_allantoate_transporters_n12
110: pSWI6-trna_transcription_n10 pACE2-allantoin_and_allantoate_transporters_n12
110: pOAF1-allantoin_and_allantoate_transporters_n12 pMBP1-cytoskeleton-dependenttransport_n4
100: pOAF1-allantoin_and_allantoate_transporters_n12
010: pOAF1-allantoin_and_allantoate_transporters_n12
110: pMBP1-cytoskeleton-dependenttransport_n4 pOAF1-other_transport_facilitators_n10
110: pSWI6-breakdown_of_lipids_fatty_acids_and_isoprenoids_n8 pACE2-allantoin_and_allantoate_transporters_n12
110: pACE2-allantoin_and_allantoate_transporters_n7 pSTB1-cytoskeleton-dependenttransport_n4
110: pMBP1-cytoskeleton-dependenttransport_n4 pSTB1-cytoskeleton-dependenttransport_n4
110: pOAF1-allantoin_and_allantoate_transporters_n12 pPHO4-cell_rescue_defense_cell_death_and_ageing_n20
100: pOAF1-allantoin_and_allantoate_transporters_n12
010: pOAF1-allantoin_and_allantoate_transporters_n12
110: pOAF1-other_transport_facilitators_n10 pFKH2-SFF
110: pOAF1-other_transport_facilitators_n10 pACE2-cell_rescue_defense_cell_death_and_ageing_n20
110: pSWI6-trna_transcription_n10 pFKH2-SFF'
110: pSWI6-SWI5 pPHO4-cell_rescue_defense_cell_death_and_ageing_n20
100: pSWI6-SWI5
110: pACE2-allantoin_and_allantoate_transporters_n7 pPHO4-cell_rescue_defense_cell_death_and_ageing_n20
110: pSWI6-breakdown_of_lipids_fatty_acids_and_isoprenoids_n8 pSTB1-cytoskeleton-dependenttransport_n4
110: pACE2-allantoin_and_allantoate_transporters_n7 pSWI6-SWI5 pACE2-allantoin_and_allantoate_transporters_n12
100: pSWI6-SWI5
110: pSWI4-phosphate_transport_n8 pSWI6-other_transport_facilitators_n10
010: pSWI6-other_transport_facilitators_n10
110: pMBP1-cytoskeleton-dependenttransport_n4 pFKH2-SFF
110: pSWI6-breakdown_of_lipids_fatty_acids_and_isoprenoids_n8 pMBP1-cytoskeleton-dependenttransport_n4
110: pSWI6-breakdown_of_lipids_fatty_acids_and_isoprenoids_n8 pACE2-cytoskeleton-dependenttransport_n4
110: pMBP1-cytoskeleton-dependenttransport_n4 pFKH2-SFF'
110: pPHO4-allantoin_and_allantoate_transporters_n6 pACE2-allantoin_and_allantoate_transporters_n7
110: pACE2-allantoin_and_allantoate_transporters_n7 pSWI6-other_transport_facilitators_n10
010: pSWI6-other_transport_facilitators_n10
110: pPHO4-cell_rescue_defense_cell_death_and_ageing_n20 pFKH2-SFF
110: pMBP1-cytoskeleton-dependenttransport_n4 pSWI6-SWI5
100: pSWI6-SWI5
110: pPHO4-cell_rescue_defense_cell_death_and_ageing_n20 pFKH2-SFF'
110: pACE2-cytoskeleton-dependenttransport_n4 pFKH2-SFF
110: pSWI4-phosphate_transport_n8 pMBP1-breakdown_of_lipids_fatty_acids_and_isoprenoids_n8
110: pSWI4-phosphate_transport_n8 pFKH2-SFF'
110: pFKH2-SFF' pACE2-cytoskeleton-dependenttransport_n4
110: pSWI4-phosphate_transport_n8 pSWI6-breakdown_of_lipids_fatty_acids_and_isoprenoids_n8
110: pPHO4-allantoin_and_allantoate_transporters_n6 pMBP1-breakdown_of_lipids_fatty_acids_and_isoprenoids_n8
110: pSWI4-phosphate_transport_n8 pFKH2-SFF
110: pPHO4-allantoin_and_allantoate_transporters_n6 pOAF1-other_transport_facilitators_n10
110: pPHO4-allantoin_and_allantoate_transporters_n6 pMBP1-cytoskeleton-dependenttransport_n4
110: pPHO4-allantoin_and_allantoate_transporters_n6 pACE2-cytoskeleton-dependenttransport_n4
110: pSWI6-other_transport_facilitators_n10 pACE2-allantoin_and_allantoate_transporters_n12

010: pSWI6-other_transport_facilitators_n10
110: pPIP2-other_transport_facilitators_n10 pFKH2-SFF'
110: pSWI4-phosphate_transport_n8 pSWI6-SWI5
100: pSWI6-SWI5
110: pSWI4-phosphate_transport_n8 pACE2-cytoskeleton-dependenttransport_n4
110: pSWI4-phosphate_transport_n8 pACE2-allantoin_and_allantoate_transporters_n7
110: pSWI6-SWI5 pACE2-cytoskeleton-dependenttransport_n4
100: pSWI6-SWI5
110: pACE2-allantoin_and_allantoate_transporters_n12 pFKH2-SFF
110: pACE2-allantoin_and_allantoate_transporters_n7 pACE2-allantoin_and_allantoate_transporters_n12
110: pFKH2-SFF' pACE2-allantoin_and_allantoate_transporters_n12
110: pMBP1-breakdown_of_lipids_fatty_acids_and_isoprenoids_n8 pACE2-cytoskeleton-dependenttransport_n4
110: pPIP2-other_transport_facilitators_n10 pSWI6-SWI5
100: pSWI6-SWI5
110: pSWI6-other_transport_facilitators_n10 pSWI6-SWI5
100: pSWI6-SWI5
010: pSWI6-other_transport_facilitators_n10
110: pSWI6-other_transport_facilitators_n10 pFKH2-SFF'
010: pSWI6-other_transport_facilitators_n10
110: pSWI6-other_transport_facilitators_n10 pFKH2-SFF
010: pSWI6-other_transport_facilitators_n10
110: pSWI6-breakdown_of_lipids_fatty_acids_and_isoprenoids_n8 pSWI6-other_transport_facilitators_n10
010: pSWI6-other_transport_facilitators_n10
110: pMBP1-breakdown_of_lipids_fatty_acids_and_isoprenoids_n8 pSWI6-other_transport_facilitators_n10
010: pSWI6-other_transport_facilitators_n10
110: pMBP1-breakdown_of_lipids_fatty_acids_and_isoprenoids_n8 pFKH2-SFF'
110: pMBP1-breakdown_of_lipids_fatty_acids_and_isoprenoids_n8 pFKH2-SFF
110: pACE2-cytoskeleton-dependenttransport_n4 pACE2-allantoin_and_allantoate_transporters_n12
110: pSWI6-trna_transcription_n10 pPHO4-cell_rescue_defense_cell_death_and_ageing_n20
110: pMBP1-breakdown_of_lipids_fatty_acids_and_isoprenoids_n8 pACE2-allantoin_and_allantoate_transporters_n12
110: pSTB1-cytoskeleton-dependenttransport_n4 pACE2-allantoin_and_allantoate_transporters_n12
110: pOAF1-allantoin_and_allantoate_transporters_n12 pACE2-cytoskeleton-dependenttransport_n4
100: pOAF1-allantoin_and_allantoate_transporters_n12
010: pOAF1-allantoin_and_allantoate_transporters_n12
110: pOAF1-other_transport_facilitators_n10 pACE2-allantoin_and_allantoate_transporters_n12
110: pSTB1-cytoskeleton-dependenttransport_n4 pPHO4-cell_rescue_defense_cell_death_and_ageing_n20
110: pPHO4-allantoin_and_allantoate_transporters_n6 pSWI6-breakdown_of_lipids_fatty_acids_and_isoprenoids_n8
110: pPHO4-allantoin_and_allantoate_transporters_n6 pSWI6-SWI5
100: pSWI6-SWI5
110: pPHO4-allantoin_and_allantoate_transporters_n6 pACE2-allantoin_and_allantoate_transporters_n12
110: pMBP1-cytoskeleton-dependenttransport_n4 pACE2-allantoin_and_allantoate_transporters_n12
110: pPIP2-metabolisof_energy_reserves_n8 pACE2-allantoin_and_allantoate_transporters_n7
110: pSWI6-trna_transcription_n10 pPIP2-metabolisof_energy_reserves_n8
110: pOAF1-other_transport_facilitators_n10 pACE2-cytoskeleton-dependenttransport_n4
110: pSWI6-trna_transcription_n10 pOAF1-other_transport_facilitators_n10
110: pPIP2-other_transport_facilitators_n10 pFKH2-SFF
110: pACE2-allantoin_and_allantoate_transporters_n7 pOAF1-other_transport_facilitators_n10
110: pMBP1-cytoskeleton-dependenttransport_n4 pPHO4-cell_rescue_defense_cell_death_and_ageing_n20
110: pSWI6-other_transport_facilitators_n10 pACE2-cytoskeleton-dependenttransport_n4
010: pSWI6-other_transport_facilitators_n10
110: pPIP2-other_transport_facilitators_n10 pACE2-cell_rescue_defense_cell_death_and_ageing_n20
110: pPIP2-metabolisof_energy_reserves_n8 pACE2-cytoskeleton-dependenttransport_n4
110: pSWI6-trna_transcription_n10 pMBP1-cytoskeleton-dependenttransport_n4
110: pOAF1-other_transport_facilitators_n10 pFKH2-SFF'
110: pPIP2-metabolisof_energy_reserves_n8 pPHO4-cell_rescue_defense_cell_death_and_ageing_n20
110: pPIP2-metabolisof_energy_reserves_n8 pMBP1-breakdown_of_lipids_fatty_acids_and_isoprenoids_n8
110: pPIP2-metabolisof_energy_reserves_n8 pFKH2-SFF'
110: pMBP1-breakdown_of_lipids_fatty_acids_and_isoprenoids_n8 pOAF1-other_transport_facilitators_n10
110: pPIP2-other_transport_facilitators_n10 pSTB1-cytoskeleton-dependenttransport_n4
110: pOAF1-other_transport_facilitators_n10 pSWI6-SWI5
100: pSWI6-SWI5
110: pPIP2-metabolisof_energy_reserves_n8 pSWI6-SWI5
100: pSWI6-SWI5
110: pPIP2-other_transport_facilitators_n10 pACE2-allantoin_and_allantoate_transporters_n12
110: pACE2-allantoin_and_allantoate_transporters_n7 pSWI6-other_transport_facilitators_n10 pSWI6-SWI5
100: pSWI6-SWI5
010: pSWI6-other_transport_facilitators_n10
110: pHIR1-SFF pHOG1-other_morphogenetic_activities_n7 pRCS1-other_morphogenetic_activities_n7
010: pHIR1-SFF
010: pRCS1-other_morphogenetic_activities_n7

100: pHIR1-SFF
100: pRCS1-other_morphogenetic_activities_n7
010: pHOG1-other_morphogenetic_activities_n7
110: pHOG1-other_morphogenetic_activities_n7 pRCS1-other_morphogenetic_activities_n7 pHIR1-SFF'
010: pHIR1-SFF'
010: pRCS1-other_morphogenetic_activities_n7
100: pHIR1-SFF'
100: pRCS1-other_morphogenetic_activities_n7
001: pHIR1-SFF'
010: pHOG1-other_morphogenetic_activities_n7
110: pARR1-other_morphogenetic_activities_n7 pHOG1-other_morphogenetic_activities_n7
010: pARR1-other_morphogenetic_activities_n7
010: pHOG1-other_morphogenetic_activities_n7
110: pIME4-g-proteins_n12 pNRG1-g-proteins_n12
010: pNRG1-g-proteins_n12
100: pNRG1-g-proteins_n12
001: pNRG1-g-proteins_n12
010: pIME4-g-proteins_n12
110: pRCS1-other_morphogenetic_activities_n7 pHOG1-SFF
010: pRCS1-other_morphogenetic_activities_n7
100: pRCS1-other_morphogenetic_activities_n7
010: pHOG1-SFF
001: pHOG1-SFF
110: pHOG1-other_morphogenetic_activities_n7 pRCS1-other_morphogenetic_activities_n7
010: pRCS1-other_morphogenetic_activities_n7
100: pRCS1-other_morphogenetic_activities_n7
010: pHOG1-other_morphogenetic_activities_n7
110: pHOG1-other_morphogenetic_activities_n7 pHIR1-SFF'
010: pHIR1-SFF'
100: pHIR1-SFF'
001: pHIR1-SFF'
010: pHOG1-other_morphogenetic_activities_n7
110: pHOG1-SFF pHIR1-SFF'
010: pHIR1-SFF'
100: pHIR1-SFF'
001: pHIR1-SFF'
010: pHOG1-SFF
001: pHOG1-SFF
110: pRTG1-other_morphogenetic_activities_n7 pHOG1-other_morphogenetic_activities_n7
100: pRTG1-other_morphogenetic_activities_n7
010: pHOG1-other_morphogenetic_activities_n7
010: pRTG1-other_morphogenetic_activities_n7
110: pHIR1-SFF pHOG1-other_morphogenetic_activities_n7
010: pHIR1-SFF
100: pHIR1-SFF
010: pHOG1-other_morphogenetic_activities_n7
110: pHIR1-SFF pHOG1-SFF
010: pHIR1-SFF
100: pHIR1-SFF
010: pHOG1-SFF
001: pHOG1-SFF
110: pRTG1-other_morphogenetic_activities_n7 pARR1-other_morphogenetic_activities_n7
100: pRTG1-other_morphogenetic_activities_n7
010: pARR1-other_morphogenetic_activities_n7
010: pRTG1-other_morphogenetic_activities_n7
110: pGCR2-other_morphogenetic_activities_n7 pHOG1-SFF
010: pGCR2-other_morphogenetic_activities_n7
010: pHOG1-SFF
001: pHOG1-SFF
110: pGCR2-other_morphogenetic_activities_n7 pHOG1-other_morphogenetic_activities_n7
010: pGCR2-other_morphogenetic_activities_n7
010: pHOG1-other_morphogenetic_activities_n7
110: pGCR2-other_morphogenetic_activities_n7 pFZF1-other_morphogenetic_activities_n7
010: pGCR2-other_morphogenetic_activities_n7
100: pFZF1-other_morphogenetic_activities_n7
010: pFZF1-other_morphogenetic_activities_n7
110: pRCS1-other_morphogenetic_activities_n7 pHIR1-SFF'
010: pHIR1-SFF'
010: pRCS1-other_morphogenetic_activities_n7
100: pHIR1-SFF'

100: pRCS1-other_morphogenetic_activities_n7
001: pHIR1-SFF'
110: pHIR1-SFF pRCS1-other_morphogenetic_activities_n7
010: pHIR1-SFF
010: pRCS1-other_morphogenetic_activities_n7
100: pHIR1-SFF
100: pRCS1-other_morphogenetic_activities_n7
110: pTEC1-STE12 pDIG1-ECB
110: pMCM1-MCM1' pIXR1-mRRPE
100: pMCM1-MCM1'
100: pIXR1-mRRPE
110: pFKH1-SFF pIXR1-mRRPE
100: pIXR1-mRRPE
110: pSTE12-ECB pIXR1-mRRPE
100: pIXR1-mRRPE
110: pSTE12-STE12 pTEC1-ECB
010: pSTE12-STE12
110: pTEC1-STE12 pSTE12-ECB
110: pTEC1-ECB pFKH1-SFF'
110: pDIG1-STE12 pTEC1-ECB
110: pMCM1-SFF' pIXR1-mRRPE
100: pIXR1-mRRPE
110: pTEC1-STE12 pTEC1-ECB
110: pMCM1-ECB pIXR1-mRRPE
100: pMCM1-ECB
010: pMCM1-ECB
100: pIXR1-mRRPE
110: pFKH1-SFF' pIXR1-mRRPE
100: pIXR1-mRRPE
110: pTEC1-ECB pIXR1-mRRPE
100: pIXR1-mRRPE
110: pTEC1-STE12 pDIG1-STE12
110: pDIG1-STE12 pSTE12-ECB
110: pSTE12-STE12 pSTE12-ECB
010: pSTE12-STE12
110: pTEC1-STE12 pSTE12-STE12
010: pSTE12-STE12
110: pDIG1-STE12 pDIG1-ECB
110: pSTE12-STE12 pDIG1-ECB
010: pSTE12-STE12
110: pFKH1-SFF' pSTE12-ECB
110: pMDS3-other_morphogenetic_activities_n7 pHOG1-SFF
100: pMDS3-other_morphogenetic_activities_n7
010: pMDS3-other_morphogenetic_activities_n7
010: pHOG1-SFF
001: pHOG1-SFF
110: pMDS3-other_morphogenetic_activities_n7 pHOG1-other_morphogenetic_activities_n7
100: pMDS3-other_morphogenetic_activities_n7
010: pMDS3-other_morphogenetic_activities_n7
010: pHOG1-other_morphogenetic_activities_n7
110: pYER051w-ALPHA1' pARG80-SFF
010: pARG80-SFF
001: pARG80-SFF
110: pMAL33-pentose-phosphate_pathway_n21 pMTH1-pentose-phosphate_pathway_n14
010: pMTH1-pentose-phosphate_pathway_n14
100: pMTH1-pentose-phosphate_pathway_n14
110: pGAT1-phosphate_transport_n13 pFKH1-pentose-phosphate_pathway_n14
100: pGAT1-phosphate_transport_n13
010: pGAT1-phosphate_transport_n13
100: pFKH1-pentose-phosphate_pathway_n14
110: pMTH1-MERE4 pGAT1-pentose-phosphate_pathway_n21
010: pMTH1-MERE4
100: pMTH1-MERE4
110: pMAL33-pentose-phosphate_pathway_n21 pMTH1-MERE4
010: pMTH1-MERE4
100: pMTH1-MERE4
110: pMTH1-pentose-phosphate_pathway_n14 pGAT1-pentose-phosphate_pathway_n21
010: pMTH1-pentose-phosphate_pathway_n14
100: pMTH1-pentose-phosphate_pathway_n14
110: pGAT1-phosphate_transport_n13 pMTH1-MERE4

010: pMTH1-MERE4
100: pMTH1-MERE4
100: pGAT1-phosphate_transport_n13
010: pGAT1-phosphate_transport_n13
110: pFKH1-pentose-phosphate_pathway_n14 pGAT1-pentose-phosphate_pathway_n21
100: pFKH1-pentose-phosphate_pathway_n14
110: pGAT1-RPN4 pFKH1-SFF
110: pMTH1-MERE4 pFKH1-pentose-phosphate_pathway_n14
010: pMTH1-MERE4
100: pMTH1-MERE4
100: pFKH1-pentose-phosphate_pathway_n14
110: pGAT1-phosphate_transport_n13 pMTH1-pentose-phosphate_pathway_n14
100: pGAT1-phosphate_transport_n13
010: pGAT1-phosphate_transport_n13
010: pMTH1-pentose-phosphate_pathway_n14
100: pMTH1-pentose-phosphate_pathway_n14
110: pGAT1-phosphate_transport_n13 pGAT1-pentose-phosphate_pathway_n21
100: pGAT1-phosphate_transport_n13
010: pGAT1-phosphate_transport_n13
110: pFKH1-SFF' pGAT1-RPN4
110: pGAT1-pentose-phosphate_pathway_n14 pMTH1-MERE4
010: pMTH1-MERE4
100: pMTH1-MERE4
100: pGAT1-pentose-phosphate_pathway_n14
010: pGAT1-pentose-phosphate_pathway_n14
110: pMTH1-MERE4 pMTH1-pentose-phosphate_pathway_n14
010: pMTH1-MERE4
100: pMTH1-MERE4
010: pMTH1-pentose-phosphate_pathway_n14
100: pMTH1-pentose-phosphate_pathway_n14
110: pFKH1-pentose-phosphate_pathway_n14 pFKH1-SFF
100: pFKH1-pentose-phosphate_pathway_n14
110: pGAT1-pentose-phosphate_pathway_n14 pGAT1-phosphate_transport_n13
100: pGAT1-phosphate_transport_n13
100: pGAT1-pentose-phosphate_pathway_n14
010: pGAT1-phosphate_transport_n13
010: pGAT1-pentose-phosphate_pathway_n14
110: pMTH1-pentose-phosphate_pathway_n14 pFKH1-pentose-phosphate_pathway_n14
010: pMTH1-pentose-phosphate_pathway_n14
100: pMTH1-pentose-phosphate_pathway_n14
100: pFKH1-pentose-phosphate_pathway_n14
110: pGAT1-pentose-phosphate_pathway_n14 pFKH1-pentose-phosphate_pathway_n14
100: pGAT1-pentose-phosphate_pathway_n14
010: pGAT1-pentose-phosphate_pathway_n14
100: pFKH1-pentose-phosphate_pathway_n14
110: pSKN7-phosphate_transport_n13 pMTH1-glyoxylate_cycle_n8
010: pMTH1-glyoxylate_cycle_n8
001: pMTH1-glyoxylate_cycle_n8
010: pSKN7-phosphate_transport_n13
100: pSKN7-phosphate_transport_n13
110: pFKH1-pentose-phosphate_pathway_n14 pFKH1-SFF'
100: pFKH1-pentose-phosphate_pathway_n14
110: pGAT1-pentose-phosphate_pathway_n14 pMTH1-pentose-phosphate_pathway_n14
100: pGAT1-pentose-phosphate_pathway_n14
010: pGAT1-pentose-phosphate_pathway_n14
010: pMTH1-pentose-phosphate_pathway_n14
100: pMTH1-pentose-phosphate_pathway_n14
110: pFKH1-regulation_of_lipid_fatty-acid_and_isoprenoid_biosynthesis_n8.scn pGAT1-regulation_of_lipid_fatty-acid_and_isoprenoid_biosynthesis_n8.scn pFKH1-SFF
010: pGAT1-regulation_of_lipid_fatty-acid_and_isoprenoid_biosynthesis_n8.scn
100: pGAT1-regulation_of_lipid_fatty-acid_and_isoprenoid_biosynthesis_n8.scn
100: pFKH1-regulation_of_lipid_fatty-acid_and_isoprenoid_biosynthesis_n8.scn pFKH1-SFF
110: pRAP1-other_mrna-transcription_activities_n20 pINO4-anion_transporters_n32
100: pINO4-anion_transporters_n32
001: pRAP1-other_mrna-transcription_activities_n20
110: pINO4-anion_transporters_n32 pINO4-other_mrna-transcription_activities_n20
010: pINO4-other_mrna-transcription_activities_n20
100: pINO4-anion_transporters_n32
110: pRAP1-anion_transporters_n20 pINO4-anion_transporters_n32
001: pRAP1-anion_transporters_n20

100: pINO4-anion_transporters_n32
110: pRAP1-other_mrna-transcription_activities_n20 pRAP1-other_signal-transduction_activities_n8
100: pRAP1-other_signal-transduction_activities_n8
001: pRAP1-other_mrna-transcription_activities_n20
110: pRAP1-anion_transporters_n20 pINO4-other_mrna-transcription_activities_n20
001: pRAP1-anion_transporters_n20
010: pINO4-other_mrna-transcription_activities_n20
110: pINO4-phosphate_transport_n18 pINO4-other_mrna-transcription_activities_n20
100: pINO4-phosphate_transport_n18
010: pINO4-other_mrna-transcription_activities_n20
010: pINO4-phosphate_transport_n18
110: pRAP1-other_mrna-transcription_activities_n20 pINO4-other_mrna-transcription_activities_n20
010: pINO4-other_mrna-transcription_activities_n20
001: pRAP1-other_mrna-transcription_activities_n20
110: pINO4-other_signal-transduction_activities_n8 pINO4-phosphate_transport_n8
100: pINO4-other_signal-transduction_activities_n8
100: pINO4-phosphate_transport_n8
110: pRAP1-other_mrna-transcription_activities_n20 pRAP1-anion_transporters_n20 pINO4-other_mrna-transcription_activities_n20
001: pRAP1-anion_transporters_n20
010: pINO4-other_mrna-transcription_activities_n20
001: pRAP1-other_mrna-transcription_activities_n20
001: pRAP1-other_mrna-transcription_activities_n20 pINO4-other_mrna-transcription_activities_n20
110: pSWI6-ionic_homeostasis_n6 pMET4-biosynthesis_of_vitamins_cofactors_and_prosthetic_groups_n8
100: pSWI6-ionic_homeostasis_n6
010: pMET4-biosynthesis_of_vitamins_cofactors_and_prosthetic_groups_n8
110: pSKN7-g-proteins_n12 pMET4-lipid_transporters_n8
010: pMET4-lipid_transporters_n8
110: pNDD1-MCM1' pMET4-biosynthesis_of_vitamins_cofactors_and_prosthetic_groups_n8
100: pNDD1-MCM1'
010: pMET4-biosynthesis_of_vitamins_cofactors_and_prosthetic_groups_n8
110: pSKN7-organization_of_chromosome_structure_n17 pMET4-lipid_transporters_n8
100: pSKN7-organization_of_chromosome_structure_n17
010: pMET4-lipid_transporters_n8
110: pSKN7-SWI5 pMET4-lipid_transporters_n8
010: pSKN7-SWI5
100: pSKN7-SWI5
010: pMET4-lipid_transporters_n8
110: pSWI6-ionic_homeostasis_n6 pNDD1-MCM1'
100: pNDD1-MCM1'
100: pSWI6-ionic_homeostasis_n6
110: pSWI6-SWI5 pMET4-biosynthesis_of_vitamins_cofactors_and_prosthetic_groups_n8
100: pSWI6-SWI5
010: pMET4-biosynthesis_of_vitamins_cofactors_and_prosthetic_groups_n8
110: pSKN7-SWI5 pNDD1-MCM1'
010: pSKN7-SWI5
100: pNDD1-MCM1'
100: pSKN7-SWI5
110: pNDD1-MCM1' pSWI6-SWI5
100: pNDD1-MCM1'
100: pSWI6-SWI5
110: pINO4-drug_transporters_n10 pRLM1-mRRPE
100: pINO4-drug_transporters_n10
110: pNDD1-MCM1' pINO4-drug_transporters_n10
100: pINO4-drug_transporters_n10
100: pNDD1-MCM1'
110: pINO4-drug_transporters_n10 pFKH1-SFF
100: pINO4-drug_transporters_n10
110: pINO4-drug_transporters_n10 pFKH2-SFF'
100: pINO4-drug_transporters_n10
110: pSTE12-ECB pFKH2-SFF'
110: pTEC1-ECB pRLM1-mRRPE
110: pTEC1-ECB pFKH2-SFF'
110: pSWI4-osmosensing_n6 pRLM1-mRRPE
010: pSWI4-osmosensing_n6
100: pSWI4-osmosensing_n6
110: pSKN7-drug_transporters_n10 pRLM1-SFF'
100: pRLM1-SFF'
010: pRLM1-SFF'
110: pINO4-drug_transporters_n10 pFKH2-SFF
100: pINO4-drug_transporters_n10

110: pFKH2-ECB pRLM1-mRRPE
110: pMCM1-MCM1' pSWI4-MCM1' pRLM1-SFF'
100: pRLM1-SFF'
100: pSWI4-MCM1'
100: pMCM1-MCM1' pSWI4-MCM1'
100: pMCM1-MCM1'
010: pSWI4-MCM1'
010: pRLM1-SFF'
110: pSWI4-MCM1' pNDD1-MCM1' pRLM1-SFF'
100: pRLM1-SFF'
100: pSWI4-MCM1'
100: pSWI4-MCM1' pNDD1-MCM1'
100: pNDD1-MCM1'
010: pSWI4-MCM1'
010: pRLM1-SFF'
110: pMCM1-SFF' pUPC2-ALPHA1' pFKH1-SFF
010: pMCM1-SFF' pFKH1-SFF
010: pUPC2-ALPHA1' pFKH1-SFF
100: pMCM1-SFF' pFKH1-SFF
110: pSWI4-MCM1' pMCM1-SFF' pRLM1-SFF'
100: pRLM1-SFF'
100: pSWI4-MCM1'
010: pSWI4-MCM1'
010: pRLM1-SFF'
110: pFKH1-other_energy_generation_activities_n12 pUPC2-ALPHA1'
010: pFKH1-other_energy_generation_activities_n12
110: pNDD1-MCM1' pRLM1-mRRPE
100: pNDD1-MCM1'
110: pSWI4-MCM1' pUPC2-ALPHA1'
100: pSWI4-MCM1'
010: pSWI4-MCM1'
110: pTEC1-ECB pSTE12-ECB
110: pSWI6-other_energy_generation_activities_n12 pSWI4-MCM1'
100: pSWI4-MCM1'
010: pSWI4-MCM1'
110: pFKH1-other_energy_generation_activities_n12 pFKH2-SFF
010: pFKH1-other_energy_generation_activities_n12
110: pTEC1-ECB pRLM1-SFF'
100: pRLM1-SFF'
010: pRLM1-SFF'
110: pSWI4-osmosensing_n6 pRLM1-SFF'
100: pRLM1-SFF'
010: pSWI4-osmosensing_n6
100: pSWI4-osmosensing_n6
010: pRLM1-SFF'
110: pRLM1-SFF' pINO4-osmosensing_n6
100: pRLM1-SFF'
010: pINO4-osmosensing_n6
100: pINO4-osmosensing_n6
010: pRLM1-SFF'
110: pTEC1-ECB pMCM1-ECB
100: pMCM1-ECB
010: pMCM1-ECB
110: pSWI4-osmosensing_n6 pINO4-drug_transporters_n10
100: pINO4-drug_transporters_n10
010: pSWI4-osmosensing_n6
100: pSWI4-osmosensing_n6
110: pMCM1-SFF' pTEC1-ECB
110: pFKH2-SFF' pRLM1-mRRPE
110: pRLM1-mRRPE pFKH2-SFF
110: pSWI4-MCM1' pRLM1-mRRPE
100: pSWI4-MCM1'
010: pSWI4-MCM1'
110: pMCM1-MCM1' pTEC1-ECB
100: pMCM1-MCM1'
110: pFKH1-other_energy_generation_activities_n12 pFKH2-SFF'
010: pFKH1-other_energy_generation_activities_n12
110: pNDD1-MCM1' pFKH2-SFF' pRLM1-SFF'
100: pRLM1-SFF'
100: pNDD1-MCM1' pFKH2-SFF'

100: pNDD1-MCM1'
100: pFKH2-SFF' pRLM1-SFF'
010: pRLM1-SFF'
110: pMCM1-MCM1' pNDD1-MCM1' pRLM1-SFF'
100: pRLM1-SFF'
100: pMCM1-MCM1' pNDD1-MCM1'
100: pNDD1-MCM1'
100: pMCM1-MCM1'
010: pRLM1-SFF'
110: pMCM1-MCM1' pRLM1-SFF' pFKH2-SFF
100: pRLM1-SFF'
100: pMCM1-MCM1' pFKH2-SFF
100: pMCM1-MCM1'
100: pRLM1-SFF' pFKH2-SFF
010: pRLM1-SFF'
110: pMCM1-SFF' pRLM1-SFF' pFKH2-SFF
100: pRLM1-SFF'
100: pMCM1-SFF' pFKH2-SFF
100: pRLM1-SFF' pFKH2-SFF
010: pRLM1-SFF'
110: pNDD1-MCM1' pFKH2-ECB pFKH1-SFF
100: pNDD1-MCM1' pFKH1-SFF
100: pNDD1-MCM1'
110: pNDD1-MCM1' pRLM1-SFF' pFKH2-SFF
100: pRLM1-SFF'
100: pNDD1-MCM1' pFKH2-SFF
100: pNDD1-MCM1'
100: pRLM1-SFF' pFKH2-SFF
010: pRLM1-SFF'
110: pMCM1-MCM1' pFKH2-SFF' pRLM1-SFF'
100: pRLM1-SFF'
100: pMCM1-MCM1' pFKH2-SFF'
100: pMCM1-MCM1'
100: pFKH2-SFF' pRLM1-SFF'
010: pRLM1-SFF'
110: pNDD1-MCM1' pFKH2-ECB pFKH1-SFF'
100: pNDD1-MCM1' pFKH1-SFF'
100: pNDD1-MCM1'
010: pFKH2-ECB pFKH1-SFF'
010: pNDD1-MCM1' pFKH1-SFF'
110: pMCM1-MCM1' pFKH2-ECB pFKH1-SFF
100: pMCM1-MCM1'
100: pMCM1-MCM1' pFKH1-SFF
110: pFKH2-ECB pRLM1-SFF'
100: pRLM1-SFF'
010: pRLM1-SFF'
110: pSWI6-other_energy_generation_activities_n12 pFKH1-SFF'
110: pSTE12-ECB pRLM1-mRRPE
110: pNDD1-ECB pRLM1-SFF'
100: pRLM1-SFF'
010: pRLM1-SFF'
110: pMCM1-SFF' pSTE12-ECB
110: pSWI6-other_energy_generation_activities_n12 pFKH1-other_energy_generation_activities_n12
010: pFKH1-other_energy_generation_activities_n12
110: pSTE12-ECB pRLM1-SFF'
100: pRLM1-SFF'
010: pRLM1-SFF'
110: pSWI6-other_energy_generation_activities_n12 pFKH1-SFF
110: pINO4-drug_transporters_n10 pFKH1-SFF'
100: pINO4-drug_transporters_n10
110: pSWI4-osmosensing_n6 pINO4-osmosensing_n6
010: pSWI4-osmosensing_n6
010: pINO4-osmosensing_n6
100: pSWI4-osmosensing_n6
100: pINO4-osmosensing_n6
110: pSWI4-MCM1' pINO4-osmosensing_n6
100: pSWI4-MCM1'
010: pINO4-osmosensing_n6
010: pSWI4-MCM1'
100: pINO4-osmosensing_n6

110: pRLM1-mRRPE pRLM1-SFF'
100: pRLM1-SFF'
010: pRLM1-SFF'
110: pNDD1-MCM1' pRLM1-SFF'
100: pRLM1-SFF'
100: pNDD1-MCM1'
010: pRLM1-SFF'
110: pRLM1-SFF' pFKH2-SFF
100: pRLM1-SFF'
010: pRLM1-SFF'
110: pFKH2-SFF' pRLM1-SFF'
100: pRLM1-SFF'
010: pRLM1-SFF'
110: pSWI4-osmosensing_n6 pFKH2-SFF
010: pSWI4-osmosensing_n6
100: pSWI4-osmosensing_n6
110: pMCM1-MCM1' pMCM1-SFF' pFKH1-SFF' pFKH2-SFF'
010: pMCM1-SFF' pFKH1-SFF'
100: pFKH1-SFF' pFKH2-SFF'
100: pMCM1-SFF' pFKH1-SFF' pFKH2-SFF'
100: pMCM1-MCM1' pFKH1-SFF' pFKH2-SFF'
100: pMCM1-SFF' pFKH2-SFF'
100: pMCM1-MCM1' pFKH2-SFF'
010: pMCM1-SFF' pFKH2-SFF'
100: pMCM1-MCM1'
010: pMCM1-MCM1' pFKH1-SFF'
100: pMCM1-MCM1' pMCM1-SFF'
100: pMCM1-MCM1' pFKH1-SFF'
100: pMCM1-SFF' pFKH1-SFF'
100: pMCM1-MCM1' pMCM1-SFF' pFKH2-SFF'
010: pFKH1-SFF' pFKH2-SFF'
110: pMCM1-MCM1' pFKH1-SFF pFKH2-SFF
100: pFKH1-SFF pFKH2-SFF
100: pMCM1-MCM1' pFKH2-SFF
100: pMCM1-MCM1'
100: pMCM1-MCM1' pFKH1-SFF
110: pMCM1-MCM1' pFKH1-SFF' pFKH2-SFF
100: pFKH1-SFF' pFKH2-SFF
100: pMCM1-MCM1' pFKH2-SFF
100: pMCM1-MCM1'
010: pMCM1-MCM1' pFKH1-SFF'
100: pMCM1-MCM1' pFKH1-SFF'
110: pINO4-drug_transporters_n10 pSKN7-drug_transporters_n10
100: pINO4-drug_transporters_n10
110: pSKN7-drug_transporters_n10 pINO4-osmosensing_n6
010: pINO4-osmosensing_n6
100: pINO4-osmosensing_n6
110: pINO4-drug_transporters_n10 pINO4-osmosensing_n6
100: pINO4-drug_transporters_n10
010: pINO4-osmosensing_n6
100: pINO4-osmosensing_n6
110: pSWI4-MCM1' pSWI4-osmosensing_n6
100: pSWI4-MCM1'
010: pSWI4-osmosensing_n6
100: pSWI4-osmosensing_n6
010: pSWI4-MCM1'
110: pNDD1-MCM1' pNDD1-ECB
100: pNDD1-MCM1'
110: pFKH2-ECB pFKH1-SFF'
110: pINO4-drug_transporters_n10 pRLM1-SFF'
100: pRLM1-SFF'
100: pINO4-drug_transporters_n10
010: pRLM1-SFF'
110: pSKN7-drug_transporters_n10 pRLM1-mRRPE
110: pTEC1-ECB pSWI4-osmosensing_n6
010: pSWI4-osmosensing_n6
100: pSWI4-osmosensing_n6
110: pNDD1-ECB pRLM1-mRRPE
110: pSWI4-osmosensing_n6 pSTE12-ECB
010: pSWI4-osmosensing_n6

100: pSWI4-osmosensing_n6
110: pSTE12-ECB pINO4-osmosensing_n6
010: pINO4-osmosensing_n6
100: pINO4-osmosensing_n6
110: pMCM1-SFF' pFKH2-ECB pFKH1-SFF'
010: pMCM1-SFF' pFKH1-SFF'
010: pFKH2-ECB pFKH1-SFF'
100: pMCM1-SFF' pFKH1-SFF'
110: pNDD1-MCM1' pMCM1-SFF' pRLM1-SFF'
100: pRLM1-SFF'
100: pNDD1-MCM1' pMCM1-SFF'
100: pNDD1-MCM1'
010: pRLM1-SFF'
110: pMCM1-SFF' pFKH2-ECB pFKH1-SFF
010: pMCM1-SFF' pFKH1-SFF
100: pMCM1-SFF' pFKH1-SFF
110: pMCM1-MCM1' pFKH2-SFF' pFKH1-SFF
100: pFKH2-SFF' pFKH1-SFF
100: pMCM1-MCM1' pFKH2-SFF'
100: pMCM1-MCM1'
100: pMCM1-MCM1' pFKH1-SFF
110: pTEC1-ECB pINO4-osmosensing_n6
010: pINO4-osmosensing_n6
100: pINO4-osmosensing_n6
110: pRLM1-mRRPE pINO4-osmosensing_n6
010: pINO4-osmosensing_n6
100: pINO4-osmosensing_n6
110: pMCM1-MCM1' pFKH2-ECB pFKH1-SFF'
100: pMCM1-MCM1'
010: pMCM1-MCM1' pFKH1-SFF'
010: pFKH2-ECB pFKH1-SFF'
100: pMCM1-MCM1' pFKH1-SFF'
110: pMCM1-MCM1' pFKH1-SFF' pFKH2-SFF'
100: pFKH1-SFF' pFKH2-SFF'
100: pMCM1-MCM1' pFKH2-SFF'
100: pMCM1-MCM1'
010: pMCM1-MCM1' pFKH1-SFF'
100: pMCM1-MCM1' pFKH1-SFF'
010: pFKH1-SFF' pFKH2-SFF'
110: pSWI4-osmosensing_n6 pSKN7-drug_transporters_n10
010: pSWI4-osmosensing_n6
100: pSWI4-osmosensing_n6
110: pUPC2-ALPHA1' pFKH1-SFF pROX1-osmosensing_n6
010: pUPC2-ALPHA1' pFKH1-SFF
100: pROX1-osmosensing_n6
010: pROX1-osmosensing_n6
110: pMCM1-SFF' pFKH2-SFF' pRLM1-SFF'
100: pRLM1-SFF'
100: pMCM1-SFF' pFKH2-SFF'
010: pMCM1-SFF' pFKH2-SFF'
100: pFKH2-SFF' pRLM1-SFF'
010: pRLM1-SFF'
110: pSKN7-g-proteins_n12 pMET4-other_protein-destination_activities_n7 pSKN7-sugar_and_carbohydrate_transporters_n6
100: pMET4-other_protein-destination_activities_n7
010: pMET4-other_protein-destination_activities_n7
110: pINO4-anion_transporters_n15 pINO4-g-proteins_n12 pINO4-SWI5
100: pINO4-g-proteins_n12 pINO4-SWI5
100: pINO4-anion_transporters_n15
010: pINO4-anion_transporters_n15 pINO4-g-proteins_n12
010: pINO4-g-proteins_n12 pINO4-SWI5
010: pINO4-anion_transporters_n15 pINO4-SWI5
010: pINO4-anion_transporters_n15
100: pINO4-anion_transporters_n15 pINO4-g-proteins_n12
001: pINO4-g-proteins_n12 pINO4-SWI5
100: pINO4-anion_transporters_n15 pINO4-SWI5
110: pSKN7-g-proteins_n12 pSKN7-SWI5 pSKN7-CSRE
010: pSKN7-SWI5
100: pSKN7-CSRE
100: pSKN7-SWI5
010: pSKN7-g-proteins_n12 pSKN7-SWI5

110: pSKN7-SWI5 pSKN7-organization_of_chromosome_structure_n17
010: pSKN7-SWI5
100: pSKN7-organization_of_chromosome_structure_n17
100: pSKN7-SWI5
110: pINO4-organization_of_chromosome_structure_n17 pINO4-g-proteins_n12
001: pINO4-organization_of_chromosome_structure_n17
110: pINO4-anion_transporters_n15 pINO4-SWI5
100: pINO4-anion_transporters_n15
010: pINO4-anion_transporters_n15
110: pINO4-g-proteins_n12 pINO4-SWI5
110: pSKN7-g-proteins_n12 pSKN7-CSRE
100: pSKN7-CSRE
110: pINO4-anion_transporters_n15 pINO4-g-proteins_n12
100: pINO4-anion_transporters_n15
010: pINO4-anion_transporters_n15
110: pSKN7-SWI5 pSKN7-CSRE
010: pSKN7-SWI5
100: pSKN7-CSRE
100: pSKN7-SWI5
110: pSWI4-organization_of_intracellular_transport_vesicles_n5 pTEC1-ECB
110: pSWI4-phosphate_transport_n8 pSWI5-phosphate_transport_n8
110: pSWI4-phosphate_transport_n8 pSWI4-organization_of_intracellular_transport_vesicles_n5
110: pSWI4-organization_of_intracellular_transport_vesicles_n5 pSWI4-MCM1
100: pSWI4-MCM1
110: pSWI6-MCB pINO4-osmosensing_n6
010: pINO4-osmosensing_n6
100: pINO4-osmosensing_n6
110: pSWI4-osmosensing_n6 pINO4-g-proteins_n12
010: pSWI4-osmosensing_n6
100: pSWI4-osmosensing_n6
110: pSWI4-phosphate_transport_n8 pSWI4-MCM1'
100: pSWI4-MCM1'
010: pSWI4-MCM1'
110: pSWI4-MCM1' pSWI4-drug_transporters_n9
100: pSWI4-MCM1'
010: pSWI4-MCM1'
110: pSWI4-MCM1' pSWI5-phosphate_transport_n8
100: pSWI4-MCM1'
010: pSWI4-MCM1'
110: pSWI4-stress_response_n24 pINO4-osmosensing_n6
010: pINO4-osmosensing_n6
100: pINO4-osmosensing_n6
110: pSWI6-MCB pINO4-g-proteins_n12
110: pSWI4-organization_of_intracellular_transport_vesicles_n5 pSTE12-ECB
110: pSWI6-MCB pSWI6-cytok9 pSWI6-nutritional_response_pathway_n7
010: pSWI6-cytok9
010: pSWI6-nutritional_response_pathway_n7
110: pSWI4-stress_response_n24 pINO4-g-proteins_n12
110: pMET4-pentose-phosphate_pathway_n23 pSWI6-SWI5
100: pSWI6-SWI5
100: pMET4-pentose-phosphate_pathway_n23
110: pSWI6-ionic_homeostasis_n6 pSWI4-cell_death_n22
100: pSWI6-ionic_homeostasis_n6
010: pSWI4-cell_death_n22
110: pFKH2-allantoin_and_allantoate_transporters_n18 pFKH2-SFF
100: pFKH2-allantoin_and_allantoate_transporters_n18
110: pSWI6-SWI5 pRLM1-mRRPE
100: pSWI6-SWI5
110: pSWI4-MCM1' pSWI4-cell_death_n22 pSWI6-SWI5
100: pSWI4-MCM1'
100: pSWI6-SWI5
010: pSWI4-MCM1' pSWI6-SWI5
010: pSWI4-cell_death_n22
010: pSWI4-MCM1'
100: pSWI4-MCM1' pSWI6-SWI5
110: pTOS8-cell_death_n22 pSWI4-organization_of_intracellular_transport_vesicles_n5
001: pTOS8-cell_death_n22
110: pMBP1-mPROTEOL18(proteolysis_n18) pFKH2-SFF
010: pMBP1-mPROTEOL18(proteolysis_n18)
110: pTOS8-cell_death_n22 pSWI4-cell_death_n22

001: pTOS8-cell_death_n22
010: pSWI4-cell_death_n22
110: pSWI6-ionic_homeostasis_n6 pSWI6-SWI5
100: pSWI6-ionic_homeostasis_n6
100: pSWI6-SWI5
110: pSWI4-MCM1' pFKH2-ECB pFKH2-MCM1
100: pSWI4-MCM1'
100: pFKH2-MCM1
010: pFKH2-MCM1
010: pSWI4-MCM1'
110: pSWI4-organization_of_intracellular_transport_vesicles_n5 pSWI4-cell_death_n22
010: pSWI4-cell_death_n22
110: pSWI6-LYS14 pSWI6-Gcr1
010: pSWI6-LYS14
110: pSWI6-Gcr1 pSWI6-mPROTEOL18(proteolysis_n18)
100: pSWI6-mPROTEOL18(proteolysis_n18)
010: pSWI6-mPROTEOL18(proteolysis_n18)
110: pSWI6-Gcr1 pMBP1-mPROTEOL18(proteolysis_n18)
010: pMBP1-mPROTEOL18(proteolysis_n18)
110: pSWI4-MCM1 pNDD1-ECB
100: pSWI4-MCM1
110: pFKH2-ECB pSWI6-SWI5
100: pSWI6-SWI5
110: pSWI4-cell_death_n22 pSWI6-SWI5
100: pSWI6-SWI5
010: pSWI4-cell_death_n22
110: pFKH2-ECB pFKH2-SFF'
110: pSWI4-MCM1' pSWI4-cell_death_n22
100: pSWI4-MCM1'
010: pSWI4-cell_death_n22
010: pSWI4-MCM1'
110: pSWI6-mPROTEOL18(proteolysis_n18) pFKH2-SFF'
100: pSWI6-mPROTEOL18(proteolysis_n18)
010: pSWI6-mPROTEOL18(proteolysis_n18)
110: pSWI4-MCM1' pMBP1-cell_death_n16
100: pSWI4-MCM1'
100: pMBP1-cell_death_n16
010: pSWI4-MCM1'
110: pSWI6-LYS14 pMBP1-mPROTEOL18(proteolysis_n18) pSWI6-SWI5
100: pSWI6-SWI5
010: pMBP1-mPROTEOL18(proteolysis_n18)
010: pSWI6-LYS14
110: pTOS8-cell_death_n22 pSWI4-MCM1'
100: pSWI4-MCM1'
001: pTOS8-cell_death_n22
010: pSWI4-MCM1'
110: pMBP1-mPROTEOL18(proteolysis_n18) pFKH2-SFF'
010: pMBP1-mPROTEOL18(proteolysis_n18)
110: pTOS8-cell_death_n22 pMBP1-cell_death_n16
100: pMBP1-cell_death_n16
001: pTOS8-cell_death_n22
110: pTOS8-cell_death_n22 pTOS8-deoxyribonucleotide_metabolisn12
001: pTOS8-deoxyribonucleotide_metabolisn12
001: pTOS8-cell_death_n22
110: pTOS8-deoxyribonucleotide_metabolisn12 pMBP1-cell_death_n16
001: pTOS8-deoxyribonucleotide_metabolisn12
100: pMBP1-cell_death_n16
110: pSWI6-SWI5 pRLM1-SFF'
100: pRLM1-SFF'
100: pSWI6-SWI5
010: pRLM1-SFF'
110: pSWI4-MCM1 pFKH2-ECB
100: pSWI4-MCM1
110: pNDD1-MCM1 pNDD1-ECB pFKH2-SFF
100: pNDD1-MCM1
110: pSWI4-MCM1 pFKH2-SFF'
100: pSWI4-MCM1
110: pNDD1-MCM1' pSWI4-MCM1
100: pNDD1-MCM1'
100: pSWI4-MCM1

110: pSWI6-SWI5 pNDD1-ECB
 100: pSWI6-SWI5
110: pFKH2-allantoin_and_allantoate_transporters_n18 pFKH2-MCM1
 100: pFKH2-MCM1
 100: pFKH2-allantoin_and_allantoate_transporters_n18
 010: pFKH2-MCM1
110: pNDD1-MCM1' pFKH2-allantoin_and_allantoate_transporters_n18
 100: pFKH2-allantoin_and_allantoate_transporters_n18
 100: pNDD1-MCM1'
110: pNDD1-MCM1 pFKH2-allantoin_and_allantoate_transporters_n18
 100: pFKH2-allantoin_and_allantoate_transporters_n18
 100: pNDD1-MCM1
110: pSWI4-MCM1 pFKH2-MCM1
 100: pFKH2-MCM1
 100: pSWI4-MCM1
 010: pFKH2-MCM1
110: pSWI6-mPROTEOL18(proteolysis_n18) pFKH2-SFF
 100: pSWI6-mPROTEOL18(proteolysis_n18)
 010: pSWI6-mPROTEOL18(proteolysis_n18)
110: pSWI4-MCM1' pNDD1-MCM1 pFKH2-ECB
 100: pSWI4-MCM1'
 100: pNDD1-MCM1
 010: pSWI4-MCM1'
110: pSWI4-MCM1' pNDD1-MCM1' pFKH2-SFF' pFKH2-SFF
 100: pSWI4-MCM1'
 100: pNDD1-MCM1' pFKH2-SFF
 100: pNDD1-MCM1' pFKH2-SFF'
 100: pSWI4-MCM1' pNDD1-MCM1'
 100: pNDD1-MCM1'
 010: pSWI4-MCM1'
 100: pSWI4-MCM1' pFKH2-SFF'
 100: pSWI4-MCM1' pFKH2-SFF
 100: pNDD1-MCM1' pFKH2-SFF' pFKH2-SFF
 100: pSWI4-MCM1' pFKH2-SFF' pFKH2-SFF
110: pFKH2-ECB pNDD1-ECB pFKH2-SFF'
110: pSWI4-MCM1 pFKH2-SFF
 100: pSWI4-MCM1
110: pFKH2-MCM1 pFKH2-SFF
 100: pFKH2-MCM1
 010: pFKH2-MCM1
110: pSWI4-MCM1' pSWI6-SWI5
 100: pSWI4-MCM1'
 100: pSWI6-SWI5
 010: pSWI4-MCM1'
110: pFKH2-SFF' pMBP1-abc_transporters_n10
110: pSWI4-MCM1' pNDD1-MCM1' pFKH2-SFF'
 100: pSWI4-MCM1'
 100: pNDD1-MCM1' pFKH2-SFF'
 100: pSWI4-MCM1' pNDD1-MCM1'
 100: pNDD1-MCM1'
 010: pSWI4-MCM1'
 100: pSWI4-MCM1' pFKH2-SFF'
110: pFKH2-ECB pFKH2-MCM1
 100: pFKH2-MCM1
 010: pFKH2-MCM1
110: pFKH2-allantoin_and_allantoate_transporters_n18 pNDD1-ECB
 100: pFKH2-allantoin_and_allantoate_transporters_n18
110: pSWI6-Gcr1 pMBP1-mPROTEOL18(proteolysis_n18) pSWI6-SWI5
 100: pSWI6-SWI5
 010: pMBP1-mPROTEOL18(proteolysis_n18)
110: pFKH2-allantoin_and_allantoate_transporters_n18 pFKH2-SFF'
 100: pFKH2-allantoin_and_allantoate_transporters_n18
110: pFKH2-ECB pFKH2-allantoin_and_allantoate_transporters_n18
 100: pFKH2-allantoin_and_allantoate_transporters_n18
110: pSWI4-MCM1 pNDD1-MCM1 pNDD1-ECB
 100: pSWI4-MCM1
 100: pNDD1-MCM1
110: pSWI4-MCM1' pFKH2-SFF' pFKH2-SFF
 100: pSWI4-MCM1'
 010: pSWI4-MCM1'

100: pSWI4-MCM1' pFKH2-SFF'
100: pSWI4-MCM1' pFKH2-SFF
110: pNDD1-MCM1' pNDD1-ECB pFKH2-MCM1
100: pFKH2-MCM1
100: pNDD1-MCM1'
010: pFKH2-MCM1
110: pSWI4-MCM1' pNDD1-MCM1' pFKH2-MCM1
100: pSWI4-MCM1'
100: pFKH2-MCM1
100: pSWI4-MCM1' pNDD1-MCM1'
100: pNDD1-MCM1'
010: pFKH2-MCM1
010: pSWI4-MCM1'
110: pSWI4-MCM1' pSWI4-MCM1 pFKH2-SFF
100: pSWI4-MCM1'
100: pSWI4-MCM1' pSWI4-MCM1
100: pSWI4-MCM1
010: pSWI4-MCM1'
100: pSWI4-MCM1' pFKH2-SFF
110: pSWI6-LYS14 pSWI6-mPROTEOL18(proteolysis_n18) pSWI6-SWI5
100: pSWI6-mPROTEOL18(proteolysis_n18)
100: pSWI6-SWI5
010: pSWI6-LYS14
010: pSWI6-mPROTEOL18(proteolysis_n18)
110: pSWI4-MCM1 pNDD1-ECB pFKH2-MCM1
100: pFKH2-MCM1
100: pSWI4-MCM1
010: pFKH2-MCM1
110: pNDD1-MCM1' pFKH2-ECB pNDD1-ECB
100: pNDD1-MCM1'
110: pMBP1-abc_transporters_n10 pFKH2-SFF
110: pSWI4-MCM1' pNDD1-MCM1' pFKH2-ECB
100: pSWI4-MCM1'
100: pSWI4-MCM1' pNDD1-MCM1'
100: pNDD1-MCM1'
010: pSWI4-MCM1'
110: pSWI6-Gcr1 pSWI6-mPROTEOL18(proteolysis_n18) pSWI6-SWI5
100: pSWI6-mPROTEOL18(proteolysis_n18)
100: pSWI6-SWI5
010: pSWI6-mPROTEOL18(proteolysis_n18)
110: pSWI6-LYS14 pMBP1-mPROTEOL18(proteolysis_n18) pSWI6-mPROTEOL18(proteolysis_n18)
100: pSWI6-mPROTEOL18(proteolysis_n18)
010: pMBP1-mPROTEOL18(proteolysis_n18)
010: pSWI6-LYS14
010: pMBP1-mPROTEOL18(proteolysis_n18) pSWI6-mPROTEOL18(proteolysis_n18)
010: pSWI6-mPROTEOL18(proteolysis_n18)
110: pSWI4-MCM1 pNDD1-MCM1 pFKH2-ECB
100: pSWI4-MCM1
100: pNDD1-MCM1
110: pNDD1-MCM1' pSWI6-SWI5 pFKH2-SFF
100: pNDD1-MCM1' pFKH2-SFF
100: pNDD1-MCM1' pSWI6-SWI5
100: pNDD1-MCM1'
100: pSWI6-SWI5
100: pSWI6-SWI5 pFKH2-SFF
110: pNDD1-MCM1' pSWI6-SWI5 pFKH2-SFF'
100: pNDD1-MCM1' pFKH2-SFF'
100: pNDD1-MCM1' pSWI6-SWI5
100: pNDD1-MCM1'
100: pSWI6-SWI5
100: pSWI6-SWI5 pFKH2-SFF'
110: pSWI6-LYS14 pSWI6-Gcr1 pMBP1-mPROTEOL18(proteolysis_n18) pSWI6-SWI5
100: pSWI6-SWI5
010: pMBP1-mPROTEOL18(proteolysis_n18)
010: pSWI6-LYS14
110: pNDD1-MCM1 pFKH2-ECB pNDD1-ECB
100: pNDD1-MCM1
110: pNDD1-MCM1' pSWI4-MCM1 pFKH2-MCM1
100: pFKH2-MCM1
100: pNDD1-MCM1'

100: pSWI4-MCM1
010: pFKH2-MCM1
110: pSWI6-LYS14 pFKH2-allantoin_and_allantoate_transporters_n18
100: pFKH2-allantoin_and_allantoate_transporters_n18
010: pSWI6-LYS14
110: pSWI6-Gcr1 pSWI6-nitrogen_and_sulphur_metabolisn16
110: pSWI6-Gcr1 pMBP1-mPROTEOL18(proteolysis_n18) pSWI6-mPROTEOL18(proteolysis_n18) pSWI6-SWI5
100: pSWI6-mPROTEOL18(proteolysis_n18)
100: pSWI6-SWI5
010: pMBP1-mPROTEOL18(proteolysis_n18)
010: pMBP1-mPROTEOL18(proteolysis_n18) pSWI6-mPROTEOL18(proteolysis_n18)
010: pSWI6-mPROTEOL18(proteolysis_n18)
110: pSWI4-MCM1' pSWI4-MCM1 pFKH2-ECB pFKH2-SFF
100: pSWI4-MCM1'
100: pSWI4-MCM1' pSWI4-MCM1
100: pSWI4-MCM1
010: pSWI4-MCM1'
100: pSWI4-MCM1' pFKH2-SFF
110: pSWI4-MCM1' pFKH2-ECB pFKH2-MCM1 pFKH2-SFF
100: pSWI4-MCM1'
100: pFKH2-MCM1
010: pFKH2-MCM1
010: pSWI4-MCM1'
100: pSWI4-MCM1' pFKH2-SFF
110: pNDD1-MCM1' pSWI4-MCM1 pNDD1-ECB
100: pNDD1-MCM1'
100: pSWI4-MCM1
110: pTOS8-cell_death_n22 pSWI4-organization_of_intracellular_transport_vesicles_n5 pSWI4-MCM1'
100: pSWI4-MCM1'
001: pTOS8-cell_death_n22
010: pSWI4-MCM1'
110: pSWI4-MCM1' pNDD1-ECB pFKH2-MCM1
100: pSWI4-MCM1'
100: pFKH2-MCM1
010: pFKH2-MCM1
010: pSWI4-MCM1'
110: pNDD1-MCM1' pNDD1-ECB pFKH2-SFF'
100: pNDD1-MCM1' pFKH2-SFF'
100: pNDD1-MCM1'
110: pFKH2-ECB pSWI6-SWI5 pFKH2-SFF
100: pSWI6-SWI5
100: pSWI6-SWI5 pFKH2-SFF
110: pSWI6-Gcr1 pSWI6-SWI5
100: pSWI6-SWI5
110: pSWI4-MCM1' pNDD1-ECB pFKH2-SFF'
100: pSWI4-MCM1'
010: pSWI4-MCM1'
100: pSWI4-MCM1' pFKH2-SFF'
110: pNDD1-MCM1 pFKH2-SFF' pFKH2-SFF
100: pNDD1-MCM1
110: pMBP1-mPROTEOL18(proteolysis_n18) pSWI6-mPROTEOL18(proteolysis_n18) pSWI6-SWI5
100: pSWI6-mPROTEOL18(proteolysis_n18)
100: pSWI6-SWI5
010: pMBP1-mPROTEOL18(proteolysis_n18)
010: pMBP1-mPROTEOL18(proteolysis_n18) pSWI6-mPROTEOL18(proteolysis_n18)
010: pSWI6-mPROTEOL18(proteolysis_n18)
110: pSWI4-organization_of_intracellular_transport_vesicles_n5 pSWI4-MCM1' pSWI4-cell_death_n22
100: pSWI4-MCM1'
010: pSWI4-cell_death_n22
010: pSWI4-MCM1'
110: pSWI4-MCM1' pNDD1-MCM1' pSWI4-MCM1
100: pSWI4-MCM1'
100: pSWI4-MCM1' pNDD1-MCM1'
100: pNDD1-MCM1'
100: pSWI4-MCM1' pSWI4-MCM1
100: pSWI4-MCM1
010: pSWI4-MCM1'
110: pSWI4-MCM1' pNDD1-MCM1' pNDD1-MCM1 pFKH2-ECB
100: pSWI4-MCM1'
100: pSWI4-MCM1' pNDD1-MCM1'

100: pNDD1-MCM1'
100: pNDD1-MCM1' pNDD1-MCM1
100: pNDD1-MCM1
010: pSWI4-MCM1'
110: pSWI4-MCM1' pNDD1-MCM1 pFKH2-ECB pFKH2-MCM1
100: pSWI4-MCM1'
100: pFKH2-MCM1
100: pNDD1-MCM1
010: pFKH2-MCM1
010: pSWI4-MCM1'
110: pNDD1-MCM1' pFKH2-ECB pFKH2-SFF
100: pNDD1-MCM1' pFKH2-SFF
100: pNDD1-MCM1'
110: pNDD1-MCM1' pFKH2-SFF' pFKH2-SFF
100: pNDD1-MCM1' pFKH2-SFF
100: pNDD1-MCM1' pFKH2-SFF'
100: pNDD1-MCM1'
110: pSWI6-LYS14 pMBP1-mPROTEOL18(proteolysis_n18) pSWI6-mPROTEOL18(proteolysis_n18) pSWI6-SWI5
100: pSWI6-mPROTEOL18(proteolysis_n18)
100: pSWI6-SWI5
010: pMBP1-mPROTEOL18(proteolysis_n18)
010: pSWI6-LYS14
010: pMBP1-mPROTEOL18(proteolysis_n18) pSWI6-mPROTEOL18(proteolysis_n18)
010: pSWI6-mPROTEOL18(proteolysis_n18)
110: pNDD1-MCM1' pSWI4-MCM1 pNDD1-MCM1
100: pNDD1-MCM1'
100: pSWI4-MCM1
100: pNDD1-MCM1' pNDD1-MCM1
100: pNDD1-MCM1
110: pSWI4-MCM1' pSWI4-MCM1 pNDD1-MCM1
100: pSWI4-MCM1'
100: pSWI4-MCM1' pSWI4-MCM1
100: pSWI4-MCM1
100: pNDD1-MCM1
010: pSWI4-MCM1'
110: pSWI4-MCM1' pNDD1-MCM1' pNDD1-MCM1
100: pSWI4-MCM1'
100: pSWI4-MCM1' pNDD1-MCM1'
100: pNDD1-MCM1'
100: pNDD1-MCM1' pNDD1-MCM1
100: pNDD1-MCM1
010: pSWI4-MCM1'
110: pNDD1-MCM1' pSWI4-MCM1 pNDD1-ECB pFKH2-MCM1
100: pFKH2-MCM1
100: pNDD1-MCM1'
100: pSWI4-MCM1
010: pFKH2-MCM1
110: pSWI4-organization_of_intracellular_transport_vesicles_n5 pSWI4-MCM1' pSWI4-MCM1
100: pSWI4-MCM1'
100: pSWI4-MCM1' pSWI4-MCM1
100: pSWI4-MCM1
010: pSWI4-MCM1'
110: pNDD1-MCM1 pFKH2-ECB pFKH2-MCM1
100: pFKH2-MCM1
100: pNDD1-MCM1
010: pFKH2-MCM1
110: pNDD1-MCM1 pNDD1-ECB pFKH2-SFF'
100: pNDD1-MCM1
110: pNDD1-MCM1' pNDD1-MCM1 pFKH2-SFF
100: pNDD1-MCM1' pFKH2-SFF
100: pNDD1-MCM1'
100: pNDD1-MCM1' pNDD1-MCM1
100: pNDD1-MCM1
110: pNDD1-MCM1' pNDD1-ECB pFKH2-SFF' pFKH2-SFF
100: pNDD1-MCM1' pFKH2-SFF
100: pNDD1-MCM1' pFKH2-SFF'
100: pNDD1-MCM1'
100: pNDD1-MCM1' pFKH2-SFF' pFKH2-SFF
110: pSWI4-MCM1 pFKH2-ECB pFKH2-MCM1
100: pFKH2-MCM1

100: pSWI4-MCM1
010: pFKH2-MCM1
110: pSWI4-MCM1 pFKH2-ECB pNDD1-ECB
100: pSWI4-MCM1
110: pMTH1-lipid_and_fatty-acid_transport_n11 pSWI6-SWI5 pGAT1-lipid_and_fatty-acid_transport_n11
010: pGAT1-lipid_and_fatty-acid_transport_n11
100: pSWI6-SWI5
100: pMTH1-lipid_and_fatty-acid_transport_n11
100: pGAT1-lipid_and_fatty-acid_transport_n11
110: pSWI6-SWI5 pMTH1-OCSE15
100: pSWI6-SWI5
110: pGAT1-lipid_and_fatty-acid_transport_n11 pMTH1-OCSE15
010: pGAT1-lipid_and_fatty-acid_transport_n11
100: pGAT1-lipid_and_fatty-acid_transport_n11
110: pMTH1-lipid_and_fatty-acid_transport_n11 pMTH1-OCSE15
100: pMTH1-lipid_and_fatty-acid_transport_n11
110: pMTH1-lipid_and_fatty-acid_transport_n11 pGAT1-lipid_and_fatty-acid_transport_n11
010: pGAT1-lipid_and_fatty-acid_transport_n11
100: pMTH1-lipid_and_fatty-acid_transport_n11
100: pGAT1-lipid_and_fatty-acid_transport_n11
110: pFKH2-allantoin_and_allantoate_transporters_n18 pFKH1-SFF'
100: pFKH2-allantoin_and_allantoate_transporters_n18
110: pMCM1-other_morphogenetic_activities_n7 pNDD1-MCM1' pMCM1-ECB
100: pMCM1-other_morphogenetic_activities_n7 pNDD1-MCM1'
100: pMCM1-other_morphogenetic_activities_n7
100: pNDD1-MCM1'
100: pMCM1-ECB
010: pMCM1-ECB
010: pMCM1-other_morphogenetic_activities_n7 pMCM1-ECB
110: pMCM1-other_morphogenetic_activities_n7 pMCM1-MCM1 pMCM1-ECB
100: pMCM1-other_morphogenetic_activities_n7
100: pMCM1-MCM1 pMCM1-ECB
100: pMCM1-ECB
100: pMCM1-MCM1
010: pMCM1-MCM1
010: pMCM1-ECB
010: pMCM1-other_morphogenetic_activities_n7 pMCM1-MCM1
010: pMCM1-other_morphogenetic_activities_n7 pMCM1-ECB
110: pMCM1-other_morphogenetic_activities_n7 pMCM1-SFF' pMCM1-ECB
100: pMCM1-other_morphogenetic_activities_n7
100: pMCM1-ECB
010: pMCM1-ECB
100: pMCM1-other_morphogenetic_activities_n7 pMCM1-SFF'
010: pMCM1-other_morphogenetic_activities_n7 pMCM1-ECB
110: pMCM1-other_morphogenetic_activities_n7 pNDD1-MCM1 pMCM1-ECB
100: pMCM1-other_morphogenetic_activities_n7
100: pMCM1-ECB
010: pMCM1-ECB
100: pNDD1-MCM1
010: pMCM1-other_morphogenetic_activities_n7 pMCM1-ECB
110: pMCM1-other_morphogenetic_activities_n7 pNDD1-ECB
100: pMCM1-other_morphogenetic_activities_n7
110: pMCM1-pentose-phosphate_pathway_n5 pMCM1-MCM1 pMCM1-ECB
100: pMCM1-pentose-phosphate_pathway_n5
100: pMCM1-MCM1 pMCM1-ECB
100: pMCM1-ECB
100: pMCM1-MCM1
010: pMCM1-MCM1
010: pMCM1-ECB
110: pMCM1-pentose-phosphate_pathway_n5 pMCM1-SFF' pMCM1-ECB
100: pMCM1-pentose-phosphate_pathway_n5
100: pMCM1-ECB
010: pMCM1-ECB
110: pMCM1-MCM1 pMCM1-ECB
100: pMCM1-ECB
100: pMCM1-MCM1
010: pMCM1-MCM1
010: pMCM1-ECB
110: pMCM1-other_morphogenetic_activities_n7 pNDD1-MCM1
100: pMCM1-other_morphogenetic_activities_n7

100: pNDD1-MCM1
110: pMCM1-other_morphogenetic_activities_n7 pNDD1-MCM1'
100: pMCM1-other_morphogenetic_activities_n7
100: pNDD1-MCM1'
110: pMCM1-other_morphogenetic_activities_n7 pMCM1-SFF'
100: pMCM1-other_morphogenetic_activities_n7
110: pHAP4-HAP234 pHAP2-allantoin_and_allantoate_transporters_n18
100: pHAP2-allantoin_and_allantoate_transporters_n18
001: pHAP4-HAP234
110: pHAP4-metal_ion_transporters_n25 pHAP2-allantoin_and_allantoate_transporters_n18
100: pHAP2-allantoin_and_allantoate_transporters_n18
110: pRGM1-ALPHA1 pPDR1-other_proteolytic_degradation_n5
010: pPDR1-other_proteolytic_degradation_n5
110: pSWI6-allantoin_and_allantoate_transporters_n13 pFKH1-SFF' pSWI6-SWI5
100: pSWI6-SWI5
100: pFKH1-SFF' pSWI6-SWI5
110: pSWI6-allantoin_and_allantoate_transporters_n13 pSWI6-SWI5 pFKH1-SFF
100: pSWI6-SWI5
100: pSWI6-SWI5 pFKH1-SFF
110: pSWI6-allantoin_and_allantoate_transporters_n13 pFKH1-SFF'
110: pSWI6-allantoin_and_allantoate_transporters_n13 pFKH1-SFF
110: pSWI6-other_energy_generation_activities_n12 pSWI6-SWI5
100: pSWI6-SWI5
110: pMCM1-other_morphogenetic_activities_n7 pNDD1-ECB pFKH2-SFF
100: pMCM1-other_morphogenetic_activities_n7 pFKH2-SFF
100: pMCM1-other_morphogenetic_activities_n7
110: pFKH2-allantoin_and_allantoate_transporters_n18 pNDD1-ECB pFKH2-SFF
100: pFKH2-allantoin_and_allantoate_transporters_n18 pFKH2-SFF
100: pFKH2-allantoin_and_allantoate_transporters_n18
110: pFKH2-ECB pFKH2-allantoin_and_allantoate_transporters_n18 pFKH2-SFF
100: pFKH2-allantoin_and_allantoate_transporters_n18 pFKH2-SFF
100: pFKH2-allantoin_and_allantoate_transporters_n18
110: pMCM1-other_morphogenetic_activities_n7 pFKH2-ECB pFKH2-SFF
100: pMCM1-other_morphogenetic_activities_n7 pFKH2-SFF
100: pMCM1-other_morphogenetic_activities_n7
110: pMCM1-nucleotide_transport_n9 pFKH2-SFF
100: pMCM1-nucleotide_transport_n9
010: pMCM1-nucleotide_transport_n9
110: pMCM1-other_morphogenetic_activities_n7 pMCM1-ECB pFKH2-SFF
100: pMCM1-other_morphogenetic_activities_n7 pFKH2-SFF
100: pMCM1-other_morphogenetic_activities_n7
100: pMCM1-ECB
010: pMCM1-ECB
010: pMCM1-other_morphogenetic_activities_n7 pMCM1-ECB
110: pGAT1-pentose-phosphate_pathway_n7 pMTH1-pentose-phosphate_pathway_n7
100: pMTH1-pentose-phosphate_pathway_n7
110: pSWI6-allantoin_and_allantoate_transporters_n13 pFKH2-SFF
110: pSWI6-allantoin_and_allantoate_transporters_n13 pFKH2-SFF'
110: pGAT1-anion_transporters_n15 pMTH1-anion_transporters_n15
100: pMTH1-anion_transporters_n15
100: pGAT1-anion_transporters_n15
110: pGAT1-pentose-phosphate_pathway_n7 pGAT1-LFTE17
001: pGAT1-LFTE17
110: pGAT1-LFTE17 pSWI6-SWI5
001: pGAT1-LFTE17
100: pSWI6-SWI5
110: pMTH1-regulation_of_nitrogen_and_sulphur_utilization_n13 pGAT1-LFTE17
001: pGAT1-LFTE17
001: pMTH1-regulation_of_nitrogen_and_sulphur_utilization_n13
110: pMTH1-regulation_of_nitrogen_and_sulphur_utilization_n13 pMTH1-pentose-phosphate_pathway_n7
001: pMTH1-regulation_of_nitrogen_and_sulphur_utilization_n13
100: pMTH1-pentose-phosphate_pathway_n7
110: pASH1-phosphate_transport_n18 pFKH1-SFF'
110: pASH1-phosphate_transport_n18 pFKH2-SFF
110: pASH1-phosphate_transport_n18 pFKH1-SFF
110: pMTH1-lipid_and_fatty-acid_transport_n11 pFKH1-SFF'
100: pMTH1-lipid_and_fatty-acid_transport_n11
110: pASH1-phosphate_transport_n18 pFKH2-SFF'
110: pSWI6-nucleotide_transport_n9 pFKH1-SFF
100: pSWI6-nucleotide_transport_n9

110: pSWI6-nucleotide_transport_n9 pSWI6-mPROTEOL18(proteolysis_n18)
100: pSWI6-mPROTEOL18(proteolysis_n18)
100: pSWI6-nucleotide_transport_n9
010: pSWI6-mPROTEOL18(proteolysis_n18)
110: pSWI6-nucleotide_transport_n9 pMBP1-mPROTEOL18(proteolysis_n18)
100: pSWI6-nucleotide_transport_n9
010: pMBP1-mPROTEOL18(proteolysis_n18)
110: pGAT1-anion_transporters_n20 pFKH1-SFF
110: pSWI6-regulation_of_amino-acid_metabolisn11 pFKH2-SFF'
110: pSWI6-nucleotide_transport_n9 pMTH1-anion_transporters_n15
100: pSWI6-nucleotide_transport_n9
100: pMTH1-anion_transporters_n15
110: pGAT1-LFTE17 pFKH1-SFF
001: pGAT1-LFTE17
110: pMTH1-lipid_and_fatty-acid_transport_n11 pGAT1-LFTE17
001: pGAT1-LFTE17
100: pMTH1-lipid_and_fatty-acid_transport_n11
110: pASH1-phosphate_transport_n18 pFKH1-SFF pFKH2-SFF
100: pFKH1-SFF pFKH2-SFF
110: pMBP1-mPROTEOL18(proteolysis_n18) pSWI6-SWI5 pFKH2-SFF
100: pSWI6-SWI5
010: pMBP1-mPROTEOL18(proteolysis_n18)
100: pSWI6-SWI5 pFKH2-SFF
110: pMTH1-lipid_and_fatty-acid_transport_n11 pFKH1-SFF
100: pMTH1-lipid_and_fatty-acid_transport_n11
110: pFKH1-SFF' pMTH1-OCSE15
110: pMTH1-lipid_and_fatty-acid_transport_n11 pGAT1-anion_transporters_n20
100: pMTH1-lipid_and_fatty-acid_transport_n11
110: pMTH1-anion_transporters_n20 pMTH1-lipid_and_fatty-acid_transport_n11
100: pMTH1-lipid_and_fatty-acid_transport_n11
110: pMTH1-anion_transporters_n20 pGAT1-lipid_and_fatty-acid_transport_n11
010: pGAT1-lipid_and_fatty-acid_transport_n11
100: pGAT1-lipid_and_fatty-acid_transport_n11
110: pGAT1-LFTE17 pFKH1-SFF'
001: pGAT1-LFTE17
110: pSWI6-mPROTEOL18(proteolysis_n18) pSWI6-SWI5 pFKH2-SFF' pFKH2-SFF
100: pSWI6-mPROTEOL18(proteolysis_n18)
100: pSWI6-SWI5
010: pSWI6-mPROTEOL18(proteolysis_n18)
100: pSWI6-SWI5 pFKH2-SFF
100: pSWI6-SWI5 pFKH2-SFF'
110: pASH1-phosphate_transport_n18 pFKH1-SFF' pFKH1-SFF
110: pASH1-phosphate_transport_n18 pFKH2-SFF' pFKH1-SFF
100: pFKH2-SFF' pFKH1-SFF
110: pGAT1-pentose-phosphate_pathway_n7 pSWI6-SWI5
100: pSWI6-SWI5
110: pGAT1-anion_transporters_n20 pFKH1-SFF'
110: pSWI6-regulation_of_amino-acid_metabolisn11 pFKH2-SFF
110: pGAT1-metal_ion_transporters_n14 pMTH1-pentose-phosphate_pathway_n7
100: pMTH1-pentose-phosphate_pathway_n7
110: pMTH1-anion_transporters_n20 pFKH1-SFF'
110: pGAT1-LFTE17 pMTH1-pentose-phosphate_pathway_n7
001: pGAT1-LFTE17
100: pMTH1-pentose-phosphate_pathway_n7
110: pMBP1-mPROTEOL18(proteolysis_n18) pSWI6-SWI5 pFKH2-SFF' pFKH2-SFF
100: pSWI6-SWI5
010: pMBP1-mPROTEOL18(proteolysis_n18)
100: pSWI6-SWI5 pFKH2-SFF
100: pSWI6-SWI5 pFKH2-SFF'
110: pSWI6-nucleotide_transport_n9 pFKH2-SFF
100: pSWI6-nucleotide_transport_n9
110: pMTH1-regulation_of_nitrogen_and_sulphur_utilization_n13 pSWI6-SWI5
100: pSWI6-SWI5
001: pMTH1-regulation_of_nitrogen_and_sulphur_utilization_n13
110: pMTH1-anion_transporters_n20 pFKH1-SFF
110: pGAT1-pentose-phosphate_pathway_n7 pGAT1-metal_ion_transporters_n14
110: pMTH1-regulation_of_nitrogen_and_sulphur_utilization_n13 pGAT1-pentose-phosphate_pathway_n7
001: pMTH1-regulation_of_nitrogen_and_sulphur_utilization_n13
110: pFKH1-SFF pMTH1-OCSE15
110: pMTH1-anion_transporters_n20 pGAT1-anion_transporters_n20

110: pMTH1-anion_transporters_n20 pGAT1-LFTE17
001: pGAT1-LFTE17
110: pSWI6-nucleotide_transport_n9 pMBP1-mPROTEOL18(proteolysis_n18) pSWI6-SWI5
100: pSWI6-nucleotide_transport_n9
100: pSWI6-SWI5
010: pMBP1-mPROTEOL18(proteolysis_n18)
110: pMTH1-regulation_of_nitrogen_and_sulphur_utilization_n13 pGAT1-metal_ion_transporters_n14
001: pMTH1-regulation_of_nitrogen_and_sulphur_utilization_n13
110: pSWI6-SWI5 pMTH1-pentose-phosphate_pathway_n7
100: pSWI6-SWI5
100: pMTH1-pentose-phosphate_pathway_n7
110: pSWI6-mPROTEOL18(proteolysis_n18) pSWI6-SWI5 pFKH2-SFF
100: pSWI6-mPROTEOL18(proteolysis_n18)
100: pSWI6-SWI5
010: pSWI6-mPROTEOL18(proteolysis_n18)
100: pSWI6-SWI5 pFKH2-SFF
110: pGAT1-anion_transporters_n20 pMTH1-OCSE15
110: pMBP1-mPROTEOL18(proteolysis_n18) pSWI6-mPROTEOL18(proteolysis_n18) pSWI6-SWI5 pFKH2-SFF' pFKH2-SFF
100: pSWI6-mPROTEOL18(proteolysis_n18)
100: pSWI6-SWI5
010: pMBP1-mPROTEOL18(proteolysis_n18)
010: pMBP1-mPROTEOL18(proteolysis_n18) pSWI6-mPROTEOL18(proteolysis_n18)
010: pSWI6-mPROTEOL18(proteolysis_n18)
100: pSWI6-SWI5 pFKH2-SFF
100: pSWI6-SWI5 pFKH2-SFF'
110: pGAT1-LFTE17 pMTH1-OCSE15
001: pGAT1-LFTE17
110: pMTH1-anion_transporters_n20 pMTH1-OCSE15
110: pMTH1-lipid_and_fatty-acid_transport_n11 pFKH1-SFF' pFKH1-SFF
100: pMTH1-lipid_and_fatty-acid_transport_n11
110: pSWI6-nucleotide_transport_n9 pSWI6-allantoin_and_allantoate_transporters_n13 pSWI6-SWI5
100: pSWI6-nucleotide_transport_n9
100: pSWI6-SWI5
110: pSWI6-SWI5 pFKH2-SFF' pFKH2-SFF
100: pSWI6-SWI5
100: pSWI6-SWI5 pFKH2-SFF
100: pSWI6-SWI5 pFKH2-SFF'
110: pMTH1-lipid_and_fatty-acid_transport_n11 pMTH1-anion_transporters_n15 pSWI6-SWI5
100: pSWI6-SWI5
100: pMTH1-anion_transporters_n15
100: pMTH1-lipid_and_fatty-acid_transport_n11
110: pMBP1-mPROTEOL18(proteolysis_n18) pFKH2-SFF' pFKH2-SFF
010: pMBP1-mPROTEOL18(proteolysis_n18)
110: pHIR1-SFF pSWI4-SCB
010: pHIR1-SFF
010: pSWI4-SCB
100: pHIR1-SFF
100: pSWI4-SCB
110: pSWI4-SCB pHIR1-SFF'
010: pHIR1-SFF'
010: pSWI4-SCB
100: pHIR1-SFF'
100: pSWI4-SCB
001: pHIR1-SFF'
110: pSWI6-abc_transporters_n10 pFKH2-SFF'
110: pNDD1-amino-acid_degradation_n8 pFKH2-SFF
110: pSWI6-regulation_of_amino-acid_metabolisn11 pSWI6-allantoin_and_allantoate_transporters_n13
110: pNDD1-amino-acid_degradation_n8 pFKH2-SFF'
110: pSWI6-allantoin_and_allantoate_transporters_n13 pSWI6-glyoxylate_cycle_n19
110: pSWI6-abc_transporters_n10 pFKH2-SFF
110: pINO4-breakdown_of_lipids_fatty_acids_and_isoprenoids_n8 pINO4-other_mrna-transcription_activities_n20
010: pINO4-other_mrna-transcription_activities_n20
110: pMTH1-deoxyribonucleotide_metabolisn5 pINO4-SWI5
110: pGAT1-metal_ion_transporters_n14 pSWI6-SWI5
100: pSWI6-SWI5
110: pSWI6-other_transport_facilitators_n5 pSWI6-SWI5 pINO4-g-proteins_n11
010: pINO4-g-proteins_n11
100: pSWI6-SWI5 pINO4-g-proteins_n11
100: pSWI6-SWI5
001: pINO4-g-proteins_n11

010: pSWI6-other_transport_facilitators_n5
100: pINO4-g-proteins_n11
110: pSWI6-SWI5 pINO4-other_mrna-transcription_activities_n20
100: pSWI6-SWI5
010: pINO4-other_mrna-transcription_activities_n20
110: pINO4-other_mrna-transcription_activities_n20 pINO4-SWI5
010: pINO4-other_mrna-transcription_activities_n20
110: pSWI6-SWI5 pINO4-SWI5
100: pSWI6-SWI5
110: pINO4-breakdown_of_lipids_fatty_acids_and_isoprenoids_n8 pGAT1-LFTE17
001: pGAT1-LFTE17
110: pGAT1-metal_ion_transporters_n14 pGAT1-LFTE17
001: pGAT1-LFTE17
110: pINO4-breakdown_of_lipids_fatty_acids_and_isoprenoids_n8 pINO4-g-proteins_n11
010: pINO4-g-proteins_n11
001: pINO4-g-proteins_n11
100: pINO4-g-proteins_n11
110: pGAT1-pentose-phosphate_pathway_n14 pGAT1-pentose-phosphate_pathway_n21
100: pGAT1-pentose-phosphate_pathway_n14
010: pGAT1-pentose-phosphate_pathway_n14
110: pGAT1-LFTE17 pINO4-g-proteins_n11
001: pGAT1-LFTE17
010: pINO4-g-proteins_n11
001: pINO4-g-proteins_n11
100: pINO4-g-proteins_n11
110: pSWI6-other_transport_facilitators_n5 pSWI6-SWI5 pINO4-SWI5
100: pSWI6-SWI5 pINO4-SWI5
100: pSWI6-SWI5
010: pSWI6-SWI5 pINO4-SWI5
010: pSWI6-other_transport_facilitators_n5
110: pGAT1-LFTE17 pINO4-other_mrna-transcription_activities_n20
001: pGAT1-LFTE17
010: pINO4-other_mrna-transcription_activities_n20
110: pSWI6-SWI5 pINO4-other_mrna-transcription_activities_n20 pINO4-g-proteins_n11
010: pINO4-other_mrna-transcription_activities_n20 pINO4-g-proteins_n11
010: pINO4-g-proteins_n11
100: pSWI6-SWI5 pINO4-g-proteins_n11
100: pSWI6-SWI5
001: pINO4-g-proteins_n11
010: pINO4-other_mrna-transcription_activities_n20
100: pINO4-g-proteins_n11
110: pSWI6-other_transport_facilitators_n5 pSWI6-SWI5 pINO4-other_mrna-transcription_activities_n20
100: pSWI6-SWI5
010: pINO4-other_mrna-transcription_activities_n20
010: pSWI6-other_transport_facilitators_n5
110: pMTH1-MERE4 pMTH1-pentose-phosphate_pathway_n14 pFKH1-pentose-phosphate_pathway_n14
010: pMTH1-MERE4
100: pMTH1-MERE4
010: pMTH1-pentose-phosphate_pathway_n14
100: pMTH1-pentose-phosphate_pathway_n14
100: pFKH1-pentose-phosphate_pathway_n14
110: pRTG3-SFF pARG80-SFF
010: pARG80-SFF
001: pARG80-SFF
100: pRTG3-SFF
010: pRTG3-SFF
110: pHIR3-SFF' pRTG3-SFF'
010: pHIR3-SFF'
010: pRTG3-SFF'
001: pHIR3-SFF'
100: pRTG3-SFF'
100: pHIR3-SFF'
110: pRTG3-SFF' pARG80-SFF
010: pARG80-SFF
010: pRTG3-SFF'
001: pARG80-SFF
100: pRTG3-SFF'
110: pHIR3-SFF' pRTG3-SFF
010: pHIR3-SFF'
001: pHIR3-SFF'

100: pRTG3-SFF
100: pHIR3-SFF'
010: pRTG3-SFF
110: pRTG1-other_morphogenetic_activities_n7 pARR1-other_morphogenetic_activities_n7 pHOG1-other_morphogenetic_activities_n7
100: pRTG1-other_morphogenetic_activities_n7
010: pARR1-other_morphogenetic_activities_n7
010: pHOG1-other_morphogenetic_activities_n7
010: pRTG1-other_morphogenetic_activities_n7
110: pSWI5-lipid_and_fatty-acid_transport_n11 pRIM101-ALPHA1'
100: pRIM101-ALPHA1'
010: pRIM101-ALPHA1'
010: pSWI5-lipid_and_fatty-acid_transport_n11
110: pSKN7-g-proteins_n12 pSKN7-SWI5 pSWI4-amino-acid_transporters_n11
010: pSKN7-SWI5
010: pSWI4-amino-acid_transporters_n11
100: pSKN7-SWI5
010: pSKN7-g-proteins_n12 pSKN7-SWI5
110: pSWI6-nitrogen_and_sulphur_metabolisn16 pSWI6-SWI5
100: pSWI6-SWI5
110: pSWI4-amino-acid_transporters_n11 pSWI6-SWI5 pSKN7-amino-acid_transporters_n11
010: pSWI4-amino-acid_transporters_n11
100: pSWI6-SWI5
010: pSKN7-amino-acid_transporters_n11
010: pSWI4-amino-acid_transporters_n11 pSWI6-SWI5
110: pSWI6-LYS14 pSWI6-nitrogen_and_sulphur_metabolisn16
010: pSWI6-LYS14
110: pSWI6-nitrogen_and_sulphur_metabolisn16 pSWI6-mPROTEOL18(proteolysis_n18)
100: pSWI6-mPROTEOL18(proteolysis_n18)
010: pSWI6-mPROTEOL18(proteolysis_n18)
110: pSKN7-g-proteins_n12 pSWI4-amino-acid_transporters_n11
010: pSWI4-amino-acid_transporters_n11
110: pSKN7-SWI5 pSWI4-amino-acid_transporters_n11
010: pSKN7-SWI5
010: pSWI4-amino-acid_transporters_n11
100: pSKN7-SWI5
110: pSWI6-nitrogen_and_sulphur_metabolisn16 pMBP1-mPROTEOL18(proteolysis_n18)
010: pMBP1-mPROTEOL18(proteolysis_n18)
110: pSWI4-amino-acid_transporters_n11 pSWI6-SWI5
010: pSWI4-amino-acid_transporters_n11
100: pSWI6-SWI5
110: pSKN7-g-proteins_n12 pSWI6-SWI5 pSKN7-amino-acid_transporters_n11
100: pSWI6-SWI5
010: pSKN7-g-proteins_n12 pSKN7-amino-acid_transporters_n11
010: pSKN7-amino-acid_transporters_n11
010: pSKN7-g-proteins_n12 pSWI6-SWI5
110: pSWI6-peroxisomal_organization_n8 pSWI6-SWI5
100: pSWI6-SWI5
110: pSWI4-amino-acid_transporters_n11 pSKN7-amino-acid_transporters_n11
010: pSWI4-amino-acid_transporters_n11
010: pSKN7-amino-acid_transporters_n11
110: pSWI6-nucleotide_transport_n9 pSWI6-SWI5
100: pSWI6-nucleotide_transport_n9
100: pSWI6-SWI5
110: pSWI6-nucleotide_transport_n9 pSWI6-SWI5 pSKN7-amino-acid_transporters_n11
100: pSWI6-nucleotide_transport_n9
100: pSWI6-SWI5
010: pSKN7-amino-acid_transporters_n11
110: pSKN7-g-proteins_n12 pSWI6-nucleotide_transport_n9 pSWI6-SWI5
100: pSWI6-nucleotide_transport_n9
100: pSWI6-SWI5
010: pSKN7-g-proteins_n12 pSWI6-SWI5
110: pSWI6-LYS14 pSWI6-nitrogen_and_sulphur_metabolisn16 pSWI6-SWI5
100: pSWI6-SWI5
010: pSWI6-LYS14
110: pSKN7-g-proteins_n12 pSWI4-amino-acid_transporters_n11 pSWI6-SWI5
010: pSWI4-amino-acid_transporters_n11
100: pSWI6-SWI5
010: pSWI4-amino-acid_transporters_n11 pSWI6-SWI5
010: pSKN7-g-proteins_n12 pSWI6-SWI5
110: pSKN7-g-proteins_n12 pSKN7-SWI5 pSKN7-amino-acid_transporters_n11

010: pSKN7-SWI5
100: pSKN7-SWI5
010: pSKN7-g-proteins_n12 pSKN7-SWI5
010: pSKN7-g-proteins_n12 pSKN7-amino-acid_transporters_n11
010: pSKN7-amino-acid_transporters_n11
010: pSKN7-SWI5 pSKN7-amino-acid_transporters_n11
110: pSWI4-stress_response_n24 pSWI4-osmosensing_n6
010: pSWI4-osmosensing_n6
100: pSWI4-osmosensing_n6
110: pSKN7-SWI5 pINO4-glyoxylate_cycle_n8 pINO4-osmosensing_n6
010: pSKN7-SWI5
010: pINO4-glyoxylate_cycle_n8 pINO4-osmosensing_n6
010: pINO4-osmosensing_n6
010: pINO4-glyoxylate_cycle_n8
100: pSKN7-SWI5
100: pINO4-osmosensing_n6
110: pSWI6-SWI5 pINO4-osmosensing_n6
010: pINO4-osmosensing_n6
100: pSWI6-SWI5
100: pINO4-osmosensing_n6
110: pINO4-phosphate_transport_n5 pSWI6-SWI5
100: pINO4-phosphate_transport_n5
100: pSWI6-SWI5
010: pINO4-phosphate_transport_n5
110: pNDD1-metabolisof_cyclic_and_unusual_nucleotides_n5 pSWI6-SWI5
100: pNDD1-metabolisof_cyclic_and_unusual_nucleotides_n5
100: pSWI6-SWI5
110: pSKN7-SWI5 pNDD1-metabolisof_cyclic_and_unusual_nucleotides_n5
010: pSKN7-SWI5
100: pNDD1-metabolisof_cyclic_and_unusual_nucleotides_n5
100: pSKN7-SWI5
110: pSKN7-SWI5 pSWI6-SWI5 pINO4-g-proteins_n12 pINO4-SWI5
010: pSKN7-SWI5
100: pSWI6-SWI5 pINO4-SWI5
100: pINO4-g-proteins_n12 pINO4-SWI5
100: pSWI6-SWI5
100: pSKN7-SWI5
010: pSKN7-SWI5 pINO4-g-proteins_n12
010: pSKN7-SWI5 pSWI6-SWI5 pINO4-SWI5
010: pSKN7-SWI5 pINO4-SWI5
010: pSWI6-SWI5 pINO4-g-proteins_n12
010: pSWI6-SWI5 pINO4-SWI5
010: pINO4-g-proteins_n12 pINO4-SWI5
001: pINO4-g-proteins_n12 pINO4-SWI5
010: pSWI6-SWI5 pINO4-g-proteins_n12 pINO4-SWI5
100: pSKN7-SWI5 pSWI6-SWI5
100: pSKN7-SWI5 pINO4-SWI5
010: pSKN7-SWI5 pSWI6-SWI5 pINO4-g-proteins_n12
010: pSKN7-SWI5 pSWI6-SWI5
110: pSWI6-SWI5 pINO4-g-proteins_n12 pINO4-SWI5
100: pSWI6-SWI5 pINO4-SWI5
100: pINO4-g-proteins_n12 pINO4-SWI5
100: pSWI6-SWI5
010: pSWI6-SWI5 pINO4-g-proteins_n12
010: pSWI6-SWI5 pINO4-SWI5
010: pINO4-g-proteins_n12 pINO4-SWI5
001: pINO4-g-proteins_n12 pINO4-SWI5
110: pSWI4-RRSE3 pSWI6-SWI5
010: pSWI4-RRSE3
100: pSWI4-RRSE3
100: pSWI6-SWI5
110: pINO4-other_energy_generation_activities_n22 pINO4-g-proteins_n12 pINO4-SWI5
100: pINO4-g-proteins_n12 pINO4-SWI5
010: pINO4-other_energy_generation_activities_n22 pINO4-g-proteins_n12
010: pINO4-g-proteins_n12 pINO4-SWI5
001: pINO4-g-proteins_n12 pINO4-SWI5
010: pINO4-other_energy_generation_activities_n22
110: pINO4-other_energy_generation_activities_n22 pINO4-g-proteins_n12
010: pINO4-other_energy_generation_activities_n22
110: pSKN7-amino-acid_transporters_n11 pINO4-osmosensing_n6

010: pINO4-osmosensing_n6
010: pSKN7-amino-acid_transporters_n11
100: pINO4-osmosensing_n6
110: pSWI4-stress_response_n24 pSWI4-SCB
010: pSWI4-SCB
100: pSWI4-SCB
110: pNDD1-metabolisof_cyclic_and_unusual_nucleotides_n5 pRLM1-SFF'
100: pRLM1-SFF'
100: pNDD1-metabolisof_cyclic_and_unusual_nucleotides_n5
010: pRLM1-SFF'
110: pSKN7-g-proteins_n12 pSWI6-SWI5 pINO4-SWI5
100: pSWI6-SWI5 pINO4-SWI5
100: pSWI6-SWI5
010: pSKN7-g-proteins_n12 pINO4-SWI5
010: pSWI6-SWI5 pINO4-SWI5
010: pSKN7-g-proteins_n12 pSWI6-SWI5
110: pSWI6-SWI5 pINO4-g-proteins_n12
100: pSWI6-SWI5
110: pSKN7-g-proteins_n12 pSWI4-amino-acid_transporters_n11 pSKN7-amino-acid_transporters_n11
010: pSWI4-amino-acid_transporters_n11
010: pSKN7-g-proteins_n12 pSKN7-amino-acid_transporters_n11
010: pSKN7-amino-acid_transporters_n11
110: pSKN7-g-proteins_n12 pINO4-glyoxylate_cycle_n8 pINO4-SWI5
010: pINO4-glyoxylate_cycle_n8
010: pSKN7-g-proteins_n12 pINO4-SWI5
110: pSKN7-g-proteins_n12 pINO4-glyoxylate_cycle_n8 pINO4-g-proteins_n12
010: pINO4-glyoxylate_cycle_n8
010: pSKN7-g-proteins_n12 pINO4-g-proteins_n12
010: pINO4-glyoxylate_cycle_n8 pINO4-g-proteins_n12
110: pSKN7-SWI5 pSWI6-SWI5 pINO4-g-proteins_n12
010: pSKN7-SWI5
100: pSWI6-SWI5
100: pSKN7-SWI5
010: pSKN7-SWI5 pINO4-g-proteins_n12
010: pSWI6-SWI5 pINO4-g-proteins_n12
100: pSKN7-SWI5 pSWI6-SWI5
010: pSKN7-SWI5 pSWI6-SWI5
110: pSKN7-SWI5 pINO4-glyoxylate_cycle_n8 pINO4-g-proteins_n12
010: pSKN7-SWI5
010: pINO4-glyoxylate_cycle_n8
100: pSKN7-SWI5
010: pSKN7-SWI5 pINO4-g-proteins_n12
010: pINO4-glyoxylate_cycle_n8 pINO4-g-proteins_n12
110: pSKN7-SWI5 pSWI4-amino-acid_transporters_n11 pSWI6-SWI5
010: pSKN7-SWI5
010: pSWI4-amino-acid_transporters_n11
100: pSWI6-SWI5
100: pSKN7-SWI5
010: pSWI4-amino-acid_transporters_n11 pSWI6-SWI5
100: pSKN7-SWI5 pSWI6-SWI5
010: pSKN7-SWI5 pSWI6-SWI5
110: pSKN7-SWI5 pSWI4-amino-acid_transporters_n11 pSKN7-amino-acid_transporters_n11
010: pSKN7-SWI5
010: pSWI4-amino-acid_transporters_n11
100: pSKN7-SWI5
010: pSKN7-amino-acid_transporters_n11
010: pSKN7-SWI5 pSKN7-amino-acid_transporters_n11
110: pSKN7-g-proteins_n12 pSWI6-SWI5 pINO4-g-proteins_n12
100: pSWI6-SWI5
010: pSKN7-g-proteins_n12 pINO4-g-proteins_n12
010: pSWI6-SWI5 pINO4-g-proteins_n12
010: pSKN7-g-proteins_n12 pSWI6-SWI5
110: pSKN7-g-proteins_n12 pNDD1-amino-acid_degradation_n7 pSKN7-amino-acid_degradation_n7
110: pSKN7-g-proteins_n12 pSKN7-SWI5 pSWI6-SWI5 pSKN7-amino-acid_transporters_n11
010: pSKN7-SWI5
100: pSWI6-SWI5
100: pSKN7-SWI5
010: pSKN7-g-proteins_n12 pSKN7-SWI5
010: pSKN7-g-proteins_n12 pSKN7-amino-acid_transporters_n11
010: pSKN7-amino-acid_transporters_n11

010: pSKN7-SWI5 pSKN7-amino-acid_transporters_n11
010: pSKN7-g-proteins_n12 pSKN7-SWI5 pSKN7-amino-acid_transporters_n11
100: pSKN7-SWI5 pSWI6-SWI5
010: pSKN7-g-proteins_n12 pSWI6-SWI5
010: pSKN7-SWI5 pSWI6-SWI5
110: pSKN7-g-proteins_n12 pSKN7-SWI5 pINO4-glyoxylate_cycle_n8
010: pSKN7-SWI5
010: pINO4-glyoxylate_cycle_n8
100: pSKN7-SWI5
010: pSKN7-g-proteins_n12 pSKN7-SWI5
110: pSKN7-SWI5 pINO4-g-proteins_n12 pINO4-SWI5
010: pSKN7-SWI5
100: pINO4-g-proteins_n12 pINO4-SWI5
100: pSKN7-SWI5
010: pSKN7-SWI5 pINO4-g-proteins_n12
010: pSKN7-SWI5 pINO4-SWI5
010: pINO4-g-proteins_n12 pINO4-SWI5
001: pINO4-g-proteins_n12 pINO4-SWI5
100: pSKN7-SWI5 pINO4-SWI5
110: pSKN7-SWI5 pINO4-glyoxylate_cycle_n8 pINO4-g-proteins_n12 pINO4-SWI5
010: pSKN7-SWI5
100: pINO4-g-proteins_n12 pINO4-SWI5
010: pINO4-glyoxylate_cycle_n8
100: pSKN7-SWI5
010: pSKN7-SWI5 pINO4-g-proteins_n12
010: pSKN7-SWI5 pINO4-SWI5
010: pINO4-g-proteins_n12 pINO4-SWI5
010: pINO4-glyoxylate_cycle_n8 pINO4-g-proteins_n12
001: pINO4-g-proteins_n12 pINO4-SWI5
100: pSKN7-SWI5 pINO4-SWI5
110: pSKN7-g-proteins_n12 pINO4-g-proteins_n12 pINO4-SWI5
100: pINO4-g-proteins_n12 pINO4-SWI5
010: pSKN7-g-proteins_n12 pINO4-SWI5
010: pSKN7-g-proteins_n12 pINO4-g-proteins_n12
010: pINO4-g-proteins_n12 pINO4-SWI5
001: pINO4-g-proteins_n12 pINO4-SWI5
110: pINO4-g-proteins_n11 pFKH1-SFF
010: pINO4-g-proteins_n11
001: pINO4-g-proteins_n11
100: pINO4-g-proteins_n11
110: pFKH1-SFF' pINO4-g-proteins_n11
010: pINO4-g-proteins_n11
001: pINO4-g-proteins_n11
100: pINO4-g-proteins_n11
110: pUME6-SCB pFKH2-MCM1
100: pFKH2-MCM1
010: pFKH2-MCM1
010: pUME6-SCB
110: pUME6-ATRepeat pUME6-SCB
100: pUME6-ATRepeat
010: pUME6-ATRepeat
010: pUME6-SCB
110: pMCM1-other_morphogenetic_activities_n7 pFKH2-ECB
100: pMCM1-other_morphogenetic_activities_n7
110: pNDD1-MCM1' pNDD1-other_cell_growth_cell_division_and_dna_synthesis_activities_n10.scn
100: pNDD1-MCM1'
110: pNDD1-other_cell_growth_cell_division_and_dna_synthesis_activities_n10.scn pNDD1-PHO
010: pNDD1-PHO
110: pNDD1-other_cell_growth_cell_division_and_dna_synthesis_activities_n10.scn pFKH2-MCM1
100: pFKH2-MCM1
010: pFKH2-MCM1
110: pNDD1-MCM1 pNDD1-other_cell_growth_cell_division_and_dna_synthesis_activities_n10.scn
100: pNDD1-MCM1
110: pMCM1-SFF' pNDD1-other_cell_growth_cell_division_and_dna_synthesis_activities_n10.scn
110: pNDD1-other_cell_growth_cell_division_and_dna_synthesis_activities_n10.scn pNDD1-ECB
110: pMCM1-other_morphogenetic_activities_n7 pNDD1-PHO
100: pMCM1-other_morphogenetic_activities_n7
010: pNDD1-PHO
110: pMCM1-other_morphogenetic_activities_n7 pNDD1-other_cell_growth_cell_division_and_dna_synthesis_activities_n10.scn
100: pMCM1-other_morphogenetic_activities_n7

110: pMCM1-MCM1' pNDD1-other_cell_growth_cell_division_and_dna_synthesis_activities_n10.scn
100: pMCM1-MCM1'
110: pMCM1-MCM1 pNDD1-other_cell_growth_cell_division_and_dna_synthesis_activities_n10.scn
100: pMCM1-MCM1
010: pMCM1-MCM1
110: pFKH2-ECB pNDD1-other_cell_growth_cell_division_and_dna_synthesis_activities_n10.scn
110: pNDD1-other_cell_growth_cell_division_and_dna_synthesis_activities_n10.scn pMCM1-ECB
100: pMCM1-ECB
010: pMCM1-ECB
110: pMCM1-other_morphogenetic_activities_n7 pFKH2-MCM1
100: pFKH2-MCM1
100: pMCM1-other_morphogenetic_activities_n7
010: pFKH2-MCM1
110: pNDD1-PHO pFKH2-SFF'
010: pNDD1-PHO
110: pMCM1-other_morphogenetic_activities_n7 pFKH2-SFF'
100: pMCM1-other_morphogenetic_activities_n7
110: pNDD1-MCM1 pNDD1-PHO
100: pNDD1-MCM1
010: pNDD1-PHO
110: pMCM1-MCM1 pNDD1-PHO
100: pMCM1-MCM1
010: pMCM1-MCM1
010: pNDD1-PHO
110: pNDD1-MCM1' pNDD1-PHO
100: pNDD1-MCM1'
010: pNDD1-PHO
110: pMCM1-SFF' pNDD1-PHO
010: pNDD1-PHO
110: pMCM1-MCM1' pNDD1-PHO
100: pMCM1-MCM1'
010: pNDD1-PHO
110: pNDD1-PHO pFKH2-MCM1
100: pFKH2-MCM1
010: pFKH2-MCM1
010: pNDD1-PHO
110: pUME6-SCB pFKH2-SFF
010: pUME6-SCB
110: pUME6-SCB pFKH2-SFF'
010: pUME6-SCB
110: pNDD1-MCM1' pFKH1-SFF' pINO4-g-proteins_n12
100: pNDD1-MCM1' pFKH1-SFF'
100: pNDD1-MCM1'
010: pNDD1-MCM1' pFKH1-SFF'
110: pNDD1-MCM1' pINO4-g-proteins_n12 pFKH2-SFF
100: pNDD1-MCM1' pFKH2-SFF
100: pNDD1-MCM1'
110: pNDD1-MCM1' pFKH1-SFF pINO4-g-proteins_n12
100: pNDD1-MCM1' pFKH1-SFF
100: pNDD1-MCM1'
110: pFKH2-SFF' pFKH1-SFF pINO4-g-proteins_n12
100: pFKH2-SFF' pFKH1-SFF
110: pFKH1-SFF' pFKH2-SFF' pINO4-g-proteins_n12
100: pFKH1-SFF' pFKH2-SFF'
010: pFKH1-SFF' pFKH2-SFF'
110: pFKH1-regulation_of_lipid_fatty-acid_and_isoprenoid_biosynthesis_n8.scn pFKH2-SFF'
110: pFKH1-regulation_of_lipid_fatty-acid_and_isoprenoid_biosynthesis_n8.scn pFKH2-SFF
110: pINO4-other_energy_generation_activities_n22 pINO4-drug_transporters_n10
100: pINO4-drug_transporters_n10
010: pINO4-other_energy_generation_activities_n22
110: pINO4-drug_transporters_n10 pINO4-g-proteins_n12
100: pINO4-drug_transporters_n10
110: pINO4-drug_transporters_n10 pINO4-SWI5
100: pINO4-drug_transporters_n10
110: pFKH1-organization_of_golgi_n7 pINO4-g-proteins_n11
010: pINO4-g-proteins_n11
001: pINO4-g-proteins_n11
100: pINO4-g-proteins_n11
110: pINO4-other_mrna-transcription_activities_n20 pINO4-g-proteins_n11 pSKN7-other_mrna-transcription_activities_n20
010: pINO4-other_mrna-transcription_activities_n20 pINO4-g-proteins_n11

010: pINO4-g-proteins_n11
010: pINO4-other_mrna-transcription_activities_n20 pSKN7-other_mrna-transcription_activities_n20
001: pINO4-g-proteins_n11
010: pINO4-other_mrna-transcription_activities_n20
010: pSKN7-other_mrna-transcription_activities_n20
100: pINO4-g-proteins_n11
110: pGAT1-anion_transporters_n20 pGAT1-lipid_and_fatty-acid_transport_n11
010: pGAT1-lipid_and_fatty-acid_transport_n11
100: pGAT1-lipid_and_fatty-acid_transport_n11
110: pGAT1-LFTE17 pGAT1-lipid_and_fatty-acid_transport_n11
001: pGAT1-LFTE17
010: pGAT1-lipid_and_fatty-acid_transport_n11
100: pGAT1-lipid_and_fatty-acid_transport_n11
110: pINO4-other_mrna-transcription_activities_n20 pINO4-osmosensing_n6
010: pINO4-osmosensing_n6
010: pINO4-other_mrna-transcription_activities_n20
100: pINO4-osmosensing_n6
110: pSKN7-lipid_and_fatty-acid_transport_n11 pINO4-osmosensing_n6
010: pSKN7-lipid_and_fatty-acid_transport_n11
010: pINO4-osmosensing_n6
100: pSKN7-lipid_and_fatty-acid_transport_n11
100: pINO4-osmosensing_n6
110: pMTH1-lipid_and_fatty-acid_transport_n11 pINO4-osmosensing_n6
010: pINO4-osmosensing_n6
100: pMTH1-lipid_and_fatty-acid_transport_n11
100: pINO4-osmosensing_n6
110: pFKH1-SFF' pGAT1-lipid_and_fatty-acid_transport_n11 pFKH1-SFF
010: pGAT1-lipid_and_fatty-acid_transport_n11
100: pGAT1-lipid_and_fatty-acid_transport_n11
110: pGAT1-anion_transporters_n20 pGAT1-LFTE17 pGAT1-lipid_and_fatty-acid_transport_n11
001: pGAT1-LFTE17
010: pGAT1-lipid_and_fatty-acid_transport_n11
100: pGAT1-LFTE17 pGAT1-lipid_and_fatty-acid_transport_n11
100: pGAT1-lipid_and_fatty-acid_transport_n11
110: pMTH1-anion_transporters_n20 pGAT1-anion_transporters_n20 pGAT1-LFTE17
001: pGAT1-LFTE17
110: pMTH1-lipid_and_fatty-acid_transport_n11 pGAT1-LFTE17 pGAT1-lipid_and_fatty-acid_transport_n11
001: pGAT1-LFTE17
010: pGAT1-lipid_and_fatty-acid_transport_n11
100: pGAT1-LFTE17 pGAT1-lipid_and_fatty-acid_transport_n11
100: pMTH1-lipid_and_fatty-acid_transport_n11
100: pGAT1-lipid_and_fatty-acid_transport_n11
110: pMTH1-anion_transporters_n20 pMTH1-lipid_and_fatty-acid_transport_n11 pGAT1-lipid_and_fatty-acid_transport_n11
010: pGAT1-lipid_and_fatty-acid_transport_n11
100: pMTH1-lipid_and_fatty-acid_transport_n11
100: pGAT1-lipid_and_fatty-acid_transport_n11
110: pMTH1-lipid_and_fatty-acid_transport_n11 pGAT1-anion_transporters_n20 pGAT1-lipid_and_fatty-acid_transport_n11
010: pGAT1-lipid_and_fatty-acid_transport_n11
100: pMTH1-lipid_and_fatty-acid_transport_n11
100: pGAT1-lipid_and_fatty-acid_transport_n11
110: pMTH1-lipid_and_fatty-acid_transport_n11 pGAT1-anion_transporters_n20 pGAT1-LFTE17
001: pGAT1-LFTE17
100: pMTH1-lipid_and_fatty-acid_transport_n11
110: pMCM1-nucleotide_transport_n9 pMCM1-SFF' pMCM1-ECB
100: pMCM1-ECB
010: pMCM1-ECB
100: pMCM1-nucleotide_transport_n9
010: pMCM1-nucleotide_transport_n9 pMCM1-ECB
010: pMCM1-nucleotide_transport_n9
110: pMCM1-nucleotide_transport_n9 pMCM1-SFF' pFKH2-ECB
100: pMCM1-nucleotide_transport_n9
010: pMCM1-nucleotide_transport_n9
110: pMCM1-MCM1' pMCM1-nucleotide_transport_n9 pFKH2-ECB
100: pMCM1-MCM1' pMCM1-nucleotide_transport_n9
100: pMCM1-MCM1'
100: pMCM1-nucleotide_transport_n9
010: pMCM1-MCM1' pMCM1-nucleotide_transport_n9
010: pMCM1-nucleotide_transport_n9
110: pMCM1-nucleotide_transport_n9 pMCM1-SFF' pNDD1-ECB
100: pMCM1-nucleotide_transport_n9

010: pMCM1-nucleotide_transport_n9
110: pMCM1-MCM1' pMCM1-nucleotide_transport_n9 pNDD1-ECB
100: pMCM1-MCM1' pMCM1-nucleotide_transport_n9
100: pMCM1-MCM1'
100: pMCM1-nucleotide_transport_n9
010: pMCM1-MCM1' pMCM1-nucleotide_transport_n9
010: pMCM1-nucleotide_transport_n9
110: pNDD1-MCM1' pMCM1-nucleotide_transport_n9
100: pNDD1-MCM1'
100: pMCM1-nucleotide_transport_n9
010: pMCM1-nucleotide_transport_n9
110: pMCM1-nucleotide_transport_n9 pMCM1-ECB
100: pMCM1-ECB
010: pMCM1-ECB
100: pMCM1-nucleotide_transport_n9
010: pMCM1-nucleotide_transport_n9
110: pMCM1-nucleotide_transport_n9 pFKH2-ECB
100: pMCM1-nucleotide_transport_n9
010: pMCM1-nucleotide_transport_n9
110: pMCM1-nucleotide_transport_n9 pNDD1-ECB
100: pMCM1-nucleotide_transport_n9
010: pMCM1-nucleotide_transport_n9
110: pMCM1-nucleotide_transport_n9 pSWI6-nucleotide_transport_n9
100: pSWI6-nucleotide_transport_n9
100: pMCM1-nucleotide_transport_n9
010: pMCM1-nucleotide_transport_n9
110: pSKN7-SWI5 pRLM1-SFF'
100: pRLM1-SFF'
010: pSKN7-SWI5
100: pSKN7-SWI5
010: pRLM1-SFF'
110: pMCM1-MCM1' pMCM1-nucleotide_transport_n9
100: pMCM1-MCM1'
100: pMCM1-nucleotide_transport_n9
010: pMCM1-nucleotide_transport_n9
110: pMCM1-nucleotide_transport_n9 pFKH1-SFF' pFKH2-SFF'
100: pFKH1-SFF' pFKH2-SFF'
100: pMCM1-nucleotide_transport_n9
010: pFKH1-SFF' pFKH2-SFF'
010: pMCM1-nucleotide_transport_n9
110: pMCM1-other_morphogenetic_activities_n7 pFKH1-SFF' pFKH2-MCM1
100: pFKH2-MCM1
100: pMCM1-other_morphogenetic_activities_n7
010: pFKH2-MCM1
100: pFKH1-SFF' pFKH2-MCM1
100: pMCM1-other_morphogenetic_activities_n7 pFKH1-SFF'
110: pMCM1-SFF' pNDD1-other_cell_growth_cell_division_and_dna_synthesis_activities_n10.scn pFKH2-SFF'
100: pMCM1-SFF' pFKH2-SFF'
010: pMCM1-SFF' pFKH2-SFF'
110: pMCM1-other_morphogenetic_activities_n7 pNDD1-PHO pFKH2-MCM1
100: pFKH2-MCM1
100: pMCM1-other_morphogenetic_activities_n7
010: pFKH2-MCM1
010: pNDD1-PHO
110: pRTG3-SFF' pFKH1-SFF'
010: pRTG3-SFF'
100: pRTG3-SFF'
110: pYOX1-ECB pRTG3-SFF'
010: pRTG3-SFF'
100: pRTG3-SFF'
010: pYOX1-ECB
110: pRTG3-SFF' pFKH2-SFF'
010: pRTG3-SFF'
100: pRTG3-SFF'
110: pFKH2-ECB pNDD1-PHO
010: pNDD1-PHO
110: pNDD1-PHO pMCM1-ECB
100: pMCM1-ECB
010: pMCM1-ECB
010: pNDD1-PHO

110: pNDD1-PHO pNDD1-ECB
010: pNDD1-PHO
110: pMCM1-nucleotide_transport_n9 pFKH2-ECB pFKH2-MCM1
100: pFKH2-MCM1
010: pFKH2-MCM1
100: pMCM1-nucleotide_transport_n9
010: pMCM1-nucleotide_transport_n9
110: pMCM1-other_morphogenetic_activities_n7 pMCM1-nucleotide_transport_n9 pNDD1-MCM1
100: pMCM1-other_morphogenetic_activities_n7
100: pNDD1-MCM1
100: pMCM1-nucleotide_transport_n9
010: pMCM1-other_morphogenetic_activities_n7 pMCM1-nucleotide_transport_n9
010: pMCM1-nucleotide_transport_n9
110: pMCM1-nucleotide_transport_n9 pNDD1-ECB pFKH2-SFF'
100: pMCM1-nucleotide_transport_n9
010: pMCM1-nucleotide_transport_n9
110: pMCM1-other_morphogenetic_activities_n7 pMCM1-nucleotide_transport_n9 pFKH2-SFF'
100: pMCM1-other_morphogenetic_activities_n7 pFKH2-SFF'
100: pMCM1-other_morphogenetic_activities_n7
100: pMCM1-nucleotide_transport_n9
010: pMCM1-other_morphogenetic_activities_n7 pMCM1-nucleotide_transport_n9
010: pMCM1-nucleotide_transport_n9
110: pMCM1-nucleotide_transport_n9 pFKH2-ECB pFKH2-SFF'
100: pMCM1-nucleotide_transport_n9
010: pMCM1-nucleotide_transport_n9
110: pMCM1-nucleotide_transport_n9 pNDD1-MCM1 pNDD1-ECB
100: pNDD1-MCM1
100: pMCM1-nucleotide_transport_n9
010: pMCM1-nucleotide_transport_n9
110: pMCM1-MCM1' pMCM1-nucleotide_transport_n9 pFKH2-MCM1
100: pFKH2-MCM1
100: pMCM1-MCM1' pMCM1-nucleotide_transport_n9
100: pMCM1-MCM1'
010: pFKH2-MCM1
100: pMCM1-nucleotide_transport_n9
010: pMCM1-MCM1' pMCM1-nucleotide_transport_n9
010: pMCM1-nucleotide_transport_n9
110: pMCM1-nucleotide_transport_n9 pNDD1-ECB pFKH2-MCM1
100: pFKH2-MCM1
010: pFKH2-MCM1
100: pMCM1-nucleotide_transport_n9
010: pMCM1-nucleotide_transport_n9
110: pMCM1-other_morphogenetic_activities_n7 pMCM1-nucleotide_transport_n9 pNDD1-ECB
100: pMCM1-other_morphogenetic_activities_n7
100: pMCM1-nucleotide_transport_n9
010: pMCM1-other_morphogenetic_activities_n7 pMCM1-nucleotide_transport_n9
010: pMCM1-nucleotide_transport_n9
110: pMCM1-MCM1' pMCM1-nucleotide_transport_n9 pFKH2-SFF'
100: pMCM1-MCM1' pFKH2-SFF'
100: pMCM1-MCM1' pMCM1-nucleotide_transport_n9
100: pMCM1-MCM1'
100: pMCM1-nucleotide_transport_n9
010: pMCM1-MCM1' pMCM1-nucleotide_transport_n9
010: pMCM1-nucleotide_transport_n9
110: pMCM1-MCM1' pMCM1-nucleotide_transport_n9 pMCM1-ECB
100: pMCM1-MCM1' pMCM1-nucleotide_transport_n9
100: pMCM1-ECB
100: pMCM1-MCM1'
010: pMCM1-ECB
100: pMCM1-nucleotide_transport_n9
010: pMCM1-nucleotide_transport_n9 pMCM1-ECB
010: pMCM1-MCM1' pMCM1-nucleotide_transport_n9
010: pMCM1-nucleotide_transport_n9
110: pMCM1-MCM1' pMCM1-nucleotide_transport_n9 pNDD1-MCM1
100: pMCM1-MCM1' pMCM1-nucleotide_transport_n9
100: pMCM1-MCM1'
100: pNDD1-MCM1
100: pMCM1-nucleotide_transport_n9
010: pMCM1-MCM1' pMCM1-nucleotide_transport_n9
010: pMCM1-nucleotide_transport_n9

110: pMCM1-nucleotide_transport_n9 pMCM1-MCM1 pNDD1-ECB
100: pMCM1-MCM1
010: pMCM1-MCM1
100: pMCM1-nucleotide_transport_n9
010: pMCM1-nucleotide_transport_n9 pMCM1-MCM1
010: pMCM1-nucleotide_transport_n9
110: pMCM1-other_morphogenetic_activities_n7 pMCM1-nucleotide_transport_n9 pMCM1-MCM1
100: pMCM1-other_morphogenetic_activities_n7
100: pMCM1-MCM1
010: pMCM1-MCM1
100: pMCM1-nucleotide_transport_n9
010: pMCM1-nucleotide_transport_n9 pMCM1-MCM1
010: pMCM1-other_morphogenetic_activities_n7 pMCM1-MCM1
010: pMCM1-other_morphogenetic_activities_n7 pMCM1-nucleotide_transport_n9
010: pMCM1-nucleotide_transport_n9
110: pMCM1-MCM1' pMCM1-nucleotide_transport_n9 pMCM1-MCM1
100: pMCM1-MCM1' pMCM1-MCM1
100: pMCM1-MCM1' pMCM1-nucleotide_transport_n9
100: pMCM1-MCM1
100: pMCM1-MCM1'
010: pMCM1-MCM1
100: pMCM1-nucleotide_transport_n9
010: pMCM1-nucleotide_transport_n9 pMCM1-MCM1
010: pMCM1-MCM1' pMCM1-nucleotide_transport_n9
010: pMCM1-nucleotide_transport_n9
110: pMCM1-other_morphogenetic_activities_n7 pMCM1-nucleotide_transport_n9 pFKH2-ECB
100: pMCM1-other_morphogenetic_activities_n7
100: pMCM1-nucleotide_transport_n9
010: pMCM1-other_morphogenetic_activities_n7 pMCM1-nucleotide_transport_n9
010: pMCM1-nucleotide_transport_n9
110: pMCM1-nucleotide_transport_n9 pNDD1-MCM1 pFKH2-ECB
100: pNDD1-MCM1
100: pMCM1-nucleotide_transport_n9
010: pMCM1-nucleotide_transport_n9
110: pMCM1-nucleotide_transport_n9 pMCM1-MCM1 pFKH2-ECB
100: pMCM1-MCM1
010: pMCM1-MCM1
100: pMCM1-nucleotide_transport_n9
010: pMCM1-nucleotide_transport_n9 pMCM1-MCM1
010: pMCM1-nucleotide_transport_n9
110: pMCM1-nucleotide_transport_n9 pNDD1-MCM1 pMCM1-ECB
100: pMCM1-ECB
010: pMCM1-ECB
100: pNDD1-MCM1
100: pMCM1-nucleotide_transport_n9
010: pMCM1-nucleotide_transport_n9 pMCM1-ECB
010: pMCM1-nucleotide_transport_n9
110: pMCM1-other_morphogenetic_activities_n7 pMCM1-nucleotide_transport_n9 pMCM1-ECB
100: pMCM1-other_morphogenetic_activities_n7
100: pMCM1-ECB
010: pMCM1-ECB
100: pMCM1-nucleotide_transport_n9
010: pMCM1-nucleotide_transport_n9 pMCM1-ECB
010: pMCM1-other_morphogenetic_activities_n7 pMCM1-ECB
010: pMCM1-other_morphogenetic_activities_n7 pMCM1-nucleotide_transport_n9
010: pMCM1-nucleotide_transport_n9
110: pMCM1-other_morphogenetic_activities_n7 pMCM1-nucleotide_transport_n9 pFKH2-MCM1
100: pFKH2-MCM1
100: pMCM1-other_morphogenetic_activities_n7
010: pFKH2-MCM1
100: pMCM1-nucleotide_transport_n9
010: pMCM1-other_morphogenetic_activities_n7 pMCM1-nucleotide_transport_n9
010: pMCM1-nucleotide_transport_n9
110: pMCM1-nucleotide_transport_n9 pMCM1-ECB pFKH2-MCM1
100: pFKH2-MCM1
100: pMCM1-ECB
010: pMCM1-ECB
010: pFKH2-MCM1
100: pMCM1-nucleotide_transport_n9
010: pMCM1-nucleotide_transport_n9 pMCM1-ECB

010: pMCM1-nucleotide_transport_n9
110: pNDD1-other_cell_growth_cell_division_and_dna_synthesis_activities_n10.scn pFKH2-SFF'
110: pFKH2-allantoin_and_allantoate_transporters_n18 pMCM1-ECB
100: pFKH2-allantoin_and_allantoate_transporters_n18
100: pMCM1-ECB
010: pMCM1-ECB
110: pMCM1-nucleotide_transport_n9 pFKH2-allantoin_and_allantoate_transporters_n18
100: pFKH2-allantoin_and_allantoate_transporters_n18
100: pMCM1-nucleotide_transport_n9
010: pMCM1-nucleotide_transport_n9
110: pMCM1-other_morphogenetic_activities_n7 pMCM1-ECB pFKH2-SFF'
100: pMCM1-other_morphogenetic_activities_n7 pFKH2-SFF'
100: pMCM1-other_morphogenetic_activities_n7
100: pMCM1-ECB
010: pMCM1-ECB
010: pMCM1-other_morphogenetic_activities_n7 pMCM1-ECB
110: pMCM1-other_morphogenetic_activities_n7 pNDD1-ECB pFKH2-SFF'
100: pMCM1-other_morphogenetic_activities_n7 pFKH2-SFF'
100: pMCM1-other_morphogenetic_activities_n7
110: pMCM1-other_morphogenetic_activities_n7 pMCM1-ECB pFKH2-MCM1
100: pFKH2-MCM1
100: pMCM1-other_morphogenetic_activities_n7
100: pMCM1-ECB
010: pMCM1-ECB
010: pFKH2-MCM1
010: pMCM1-other_morphogenetic_activities_n7 pMCM1-ECB
110: pMCM1-other_morphogenetic_activities_n7 pNDD1-ECB pFKH2-MCM1
100: pFKH2-MCM1
100: pMCM1-other_morphogenetic_activities_n7
010: pFKH2-MCM1
110: pMCM1-nucleotide_transport_n9 pMCM1-MCM1
100: pMCM1-MCM1
010: pMCM1-MCM1
100: pMCM1-nucleotide_transport_n9
010: pMCM1-nucleotide_transport_n9
110: pMCM1-MCM1 pFKH2-allantoin_and_allantoate_transporters_n18
100: pFKH2-allantoin_and_allantoate_transporters_n18
100: pMCM1-MCM1
010: pMCM1-MCM1
110: pMCM1-other_morphogenetic_activities_n7 pFKH1-SFF'
100: pMCM1-other_morphogenetic_activities_n7
110: pMCM1-other_morphogenetic_activities_n7 pMCM1-nucleotide_transport_n9
100: pMCM1-other_morphogenetic_activities_n7
100: pMCM1-nucleotide_transport_n9
010: pMCM1-nucleotide_transport_n9
110: pMCM1-other_morphogenetic_activities_n7 pNDD1-PHO pFKH2-SFF'
100: pMCM1-other_morphogenetic_activities_n7 pFKH2-SFF'
100: pMCM1-other_morphogenetic_activities_n7
010: pNDD1-PHO
110: pMCM1-nucleotide_transport_n9 pFKH1-SFF' pFKH2-MCM1
100: pFKH2-MCM1
010: pFKH2-MCM1
100: pMCM1-nucleotide_transport_n9
100: pFKH1-SFF' pFKH2-MCM1
010: pMCM1-nucleotide_transport_n9
110: pMCM1-other_morphogenetic_activities_n7 pNDD1-MCM1 pFKH1-SFF'
100: pMCM1-other_morphogenetic_activities_n7
100: pNDD1-MCM1
100: pMCM1-other_morphogenetic_activities_n7 pFKH1-SFF'
110: pMCM1-other_morphogenetic_activities_n7 pNDD1-MCM1' pFKH1-SFF'
100: pNDD1-MCM1' pFKH1-SFF'
100: pMCM1-other_morphogenetic_activities_n7 pNDD1-MCM1'
100: pMCM1-other_morphogenetic_activities_n7
100: pNDD1-MCM1'
100: pMCM1-other_morphogenetic_activities_n7 pFKH1-SFF'
010: pNDD1-MCM1' pFKH1-SFF'
110: pMCM1-other_morphogenetic_activities_n7 pFKH1-SFF' pFKH2-SFF'
100: pFKH1-SFF' pFKH2-SFF'
100: pMCM1-other_morphogenetic_activities_n7 pFKH2-SFF'
100: pMCM1-other_morphogenetic_activities_n7

100: pMCM1-other_morphogenetic_activities_n7 pFKH1-SFF'
010: pFKH1-SFF' pFKH2-SFF'
110: pMCM1-nucleotide_transport_n9 pMCM1-MCM1 pMCM1-ECB
100: pMCM1-MCM1 pMCM1-ECB
100: pMCM1-ECB
100: pMCM1-MCM1
010: pMCM1-MCM1
010: pMCM1-ECB
100: pMCM1-nucleotide_transport_n9
010: pMCM1-nucleotide_transport_n9 pMCM1-ECB
010: pMCM1-nucleotide_transport_n9 pMCM1-MCM1
010: pMCM1-nucleotide_transport_n9
110: pMCM1-nucleotide_transport_n9 pMCM1-ECB pFKH2-SFF'
100: pMCM1-ECB
010: pMCM1-ECB
100: pMCM1-nucleotide_transport_n9
010: pMCM1-nucleotide_transport_n9 pMCM1-ECB
010: pMCM1-nucleotide_transport_n9
110: pMCM1-nucleotide_transport_n9 pMCM1-MCM1 pFKH1-SFF'
100: pMCM1-MCM1
010: pMCM1-MCM1 pFKH1-SFF'
010: pMCM1-MCM1
100: pMCM1-nucleotide_transport_n9
010: pMCM1-nucleotide_transport_n9 pMCM1-MCM1
010: pMCM1-nucleotide_transport_n9
110: pMCM1-nucleotide_transport_n9 pNDD1-MCM1 pFKH1-SFF'
100: pNDD1-MCM1
100: pMCM1-nucleotide_transport_n9
010: pMCM1-nucleotide_transport_n9
110: pMCM1-other_morphogenetic_activities_n7 pMCM1-MCM1 pFKH1-SFF'
100: pMCM1-other_morphogenetic_activities_n7
100: pMCM1-MCM1
010: pMCM1-MCM1 pFKH1-SFF'
010: pMCM1-MCM1
100: pMCM1-other_morphogenetic_activities_n7 pFKH1-SFF'
010: pMCM1-other_morphogenetic_activities_n7 pMCM1-MCM1
110: pMCM1-other_morphogenetic_activities_n7 pFKH2-ECB pNDD1-PHO
100: pMCM1-other_morphogenetic_activities_n7
010: pNDD1-PHO
