# Supplementary material for: Revealing cell cycle control by combining model-based detection of periodic expression with novel cis-regulatory descriptors
Source: BMC Syst Biol. 2007 Oct 16;1:45. doi: 10.1186/1752-0509-1-45 (PMC2200664; doi:10.1186/1752-0509-1-45)
Supplement: Additional file 4 — Names of the putative sequence motifs. See P# in Figure 5. [file 1752-0509-1-45-S4.pdf]

Names of the putative sequence motifs (P# in Figure 5).

P#	Putative sequence motif (full name)
1	g-proteins_n11
2	g-proteins_n12
3	phosphate_transport_n5
4	pentose-phosphate_pathway_n14
5	amino-acid_degradation_n8
6	organization_of_plasma_membrane_n17
7	other_signal-transduction_activities_n8
8	osmosensing_n6
9	other_pheromone_response_activities_n14
10	allantoin_and_allantoate_transporters_n7
11	drug_transporters_n7
12	phosphate_transport_n18
13	other_proteolytic_degradation_n2
14	regulation_of_nitrogen_and_sulphur_utilization_n10
15	anion_transporters_n4
16	regulation_of_lipid_fatty-acid_and_isoprenoid_biosynthesis_n8.scn
17	deoxyribonucleotide_metabolism_n23
18	mPROTEOL18(m_proteolysis_n18)
19	other_proteolytic_degradation_n8
20	utilization_of_vitamins_cofactors_and_prosthetic_groups_n7
21	utilization_of_vitamins_cofactors_and_prosthetic_groups_n6
22	amino-acid_transport_n14
23	mitochondrial_biogenesis_n5
24	allantoin_and_allantoate_transporters_n18
25	drug_transporters_n9
26	other_morphogenetic_activities_n7
27	allantoin_and_allantoate_transporters_n13
28	glycolysis_and_gluconeogenesis_n11
29	deoxyribonucleotide_metabolism_n27
30	deoxyribonucleotide_metabolism_n10
31	other_proteolytic_degradation_n7
32	nucleotide_transport_n9
33	ion_transporters_n3
34	anion_transporters_n19
35	cell_death_n22
36	regulation_of_amino-acid_metabolism_n7
37	other_energy_generation_activities_n4
38	regulation_of_lipid_fatty-acid_and_isoprenoid_biosynthesis_n20.scn
39	nitrogen_and_sulphur_utilization_n4
40	amino-acid_transport_n18
41	regulation_of_amino-acid_metabolism_n15
42	glycolysis_and_gluconeogenesis_n14
43	chromatin_modification_n9
44	other_pheromone_response_activities_n8
45	metal_ion_transporters_n10
46	regulation_of_nitrogen_and_sulphur_utilization_n7
47	biogenesis_of_chromosome_structure_n9

48	metabolism_of_cyclic_and_unusual_nucleotides_n5
49	other_cation_transporters_n14
50	metal_ion_transporters_n14
51	organization_of_cell_wall_n6
52	cell_death_n15
53	lipid_and_fatty-acid_transport_n11
54	pheromone_response_generation_n10
55	amino-acid_transporters_n11
56	other_mrna-transcription_activities_n11
57	nutritional_response_pathway_n7
58	other_transport_facilitators_n10
59	morphogenesis_n5
60	other_protein-destination_activities_n7
61	other_cation_transporters_n7
62	pheromone_response_generation_n12
63	trna_transcription_n10
64	drug_transporters_n10
65	ionic_homeostasis_n6
66	other_energy_generation_activities_n17
67	metabolism_of_energy_reserves_n30
68	chromatin_modification_n21
69	cytokinesis_n10
70	anion_transporters_n17
71	other_morphogenetic_activities_n8
72	anion_transporters_n15
73	nitrogen_and_sulphur_metabolism_n17
74	cell_death_n16
75	amino-acid_degradation_n7
76	homeostasis_of_other_ions_n30
77	biogenesis_of_cytoskeleton_n12
78	lipid_transporters_n8
79	amino-acid_transport_n13
80	stress_response_n17
81	glyoxylate_cycle_n11
82	other_cell_growth_cell_division_and_dna_synthesis_activities_n10.scn
83	other_intracellular-transport_activities_n6
84	amino-acid_degradation_n32
85	metal_ion_transporters_n17
86	anion_transporters_n16
87	pentose-phosphate_pathway_n23
88	other_signal-transduction_activities_n13
89	organization_of_golgi_n7
90	amino-acid_degradation_n24
91	other_intracellular-transport_activities_n9
92	other_pheromone_response_activities_n5
93	pheromone_response_generation_n4
94	amino-acid_degradation_n27
95	trna_processing_n6
96	glyoxylate_cycle_n8
97	lipid_and_fatty-acid_binding_n15

98	peroxisomal_organization_n28
99	other_nucleotide-metabolism_activities_n18
100	phosphate_utilization_n9
101	abc_transporters_n10
102	glyoxylate_cycle_n19
103	breakdown_of_lipids_fatty_acids_and_isoprenoids_n8
104	deoxyribonucleotide_metabolism_n4
105	biogenesis_of_chromosome_structure_n18
106	other_nucleotide-metabolism_activities_n17
107	allantoin_and_allantoate_transporters_n11
108	g-proteins_n13
109	other_transport_facilitators_n5
110	tricarboxylic-acid_pathway_n6
111	anion_transporters_n22
112	pentose-phosphate_pathway_n5
113	regulation_of_lipid_fatty-acid_and_isoprenoid_biosynthesis_n16.scn
114	other_energy_generation_activities_n9
115	metabolism_of_energy_reserves_n27
116	vacuolar_and_lysosomal_organization_n8
117	fermentation_n18
118	lipid_and_fatty-acid_binding_n13
119	nitrogen_and_sulphur_transport_n9
120	peroxisomal_transport_n22
121	other_proteolytic_degradation_n5
122	translational_control_n10
123	other_cell_rescue_activities_n10
124	ion_transporters_n4
125	other_mrna-transcription_activities_n20
126	tricarboxylic-acid_pathway_n9
127	lipid_and_fatty-acid_binding_n14
128	anion_transporters_n20
129	deoxyribonucleotide_metabolism_n12
130	amino-acid_transport_n20
131	other_cell_growth_cell_division_and_dna_synthesis_activities_n14.scn
132	organization_of_chromosome_structure_n12
133	other_transcription_activities_n5
134	other_energy_generation_activities_n16
135	amino-acid_metabolism_n25
136	phosphate_transport_n13
137	organization_of_chromosome_structure_n17
138	nutritional_response_pathway_n3
139	amino-acid_metabolism_n14
140	other_energy_generation_activities_n12
141	deoxyribonucleotide_metabolism_n5
142	fermentation_n4
143	glycolysis_and_gluconeogenesis_n4
144	other_energy_generation_activities_n22
145	sugar_and_carbohydrate_transporters_n6
146	lipid_and_fatty-acid_transport_n7
147	cell_rescue_defense_cell_death_and_ageing_n20

148 cytoskeleton-dependenttransport_n4
149 phosphate_transport_n8
150 allantoin_and_allantoate_transporters_n6
151 metabolism_of_energy_reserves_n8
152 allantoin_and_allantoate_transporters_n12
153 regulation_of_amino-acid_metabolism_n10
154 regulation_of_lipid_fatty-acid_and_isoprenoid_biosynthesis_n12.scn
155 regulation_of_nitrogen_and_sulphur_utilization_n12
156 nutritional_response_pathway_n12
157 other_nutritional-response_activities_n10
158 purine_and_pyrimidine_transporters_n17
159 other_transcription_activities_n8
160 glycolysis_and_gluconeogenesis_n27
161 ion_transporters_n11
162 phosphate_utilization_n7
163 regulation_of_nitrogen_and_sulphur_utilization_n13
164 biosynthesis_of_vitamins_cofactors_and_prosthetic_groups_n8
165 cell_death_n8
166 anion_transporters_n10
167 regulation_of_lipid_fatty-acid_and_isoprenoid_biosynthesis_n22.scn
168 nutritional_response_pathway_n8
169 organization_of_intracellular_transport_vesicles_n5
170 nitrogen_and_sulphur_utilization_n15
171 biogenesis_of_cytoskeleton_n5
172 c-compound_and_carbohydrate_utilization_n9
173 metal_ion_transporters_n25
174 pentose-phosphate_pathway_n21
175 anion_transporters_n32
176 ion_transporters_n14
177 peroxisomal_organization_n6
178 c-compound_and_carbohydrate_metabolism_n8
179 stress_response_n24
180 intracellular_communication_n10
181 nitrogen_and_sulphur_metabolism_n16
182 organization_of_plasma_membrane_n15
183 lysosomal_and_vacuolar_degradation_n3
184 amino-acid_transport_n3
185 pentose-phosphate_pathway_n7
186 other_pheromone_response_activities_n12
187 anion_transporters_n9
188 peroxisomal_organization_n8
189 purine_and_pyrimidine_transporters_n10
190 anion_transporters_n27
191 pheromone_response_generation_n7
192 regulation_of_amino-acid_metabolism_n11
193 lysosomal_and_vacuolar_degradation_n8
194 polynucleotide_degradation_n3
195 deoxyribonucleotide_metabolism_n8
196 ion_transporters_n7
197 c-compound_carbohydrate_transport_n11

198 other_nutritional-response_activities_n6
199 metal_ion_transporters_n6
200 regulation_of_nucleotide_metabolism_n5
201 organization_of_cell_wall_n10
202 intracellular_communication_n4
203 peroxisomal_transport_n19
204 fermentation_n3
205 glyoxylate_cycle_n7
206 homeostasis_of_metal_ions_n20
