# Supplementary material for: Revealing cell cycle control by combining model-based detection of periodic expression with novel cis-regulatory descriptors
Source: BMC Syst Biol. 2007 Oct 16;1:45. doi: 10.1186/1752-0509-1-45 (PMC2200664; doi:10.1186/1752-0509-1-45)
Supplement: Additional file 5 — The functional signatures of each periodic class. Overrepresentation of function was calculated for a background consisting only of genes detected as periodically expressed in at least one of the experiments. Terms with a two-sided p-value below 0.05 in at least one class are shown. [file 1752-0509-1-45-S5.pdf]

The functional signatures of each periodic class. Over representation was calculated for a background consisting only of genes detected as periodically expressed in at least one of the experiments. Terms with a two-sided p-value below 0.05 in at least one class are shown.

	Over represented (two sided p-values)						
**GO id,term**	**001**	**010**	**011**	**100**	**101**	**110**	**111**
GO:0000921 septin ring assembly	1	1	1	1	1	1	0.00111
GO:0031106 septin ring organization	1	1	1	1	1	1	0.00111
GO:0007126 meiosis	0.62	0.365	1	0.198	1	0.00285	0.335
GO:0051321 meiotic cell cycle	0.62	0.365	1	0.198	1	0.00285	0.335
GO:0051327 M phase of meiotic cell cycle	0.62	0.365	1	0.198	1	0.00285	0.335
GO:0000910 cytokinesis	0.249	0.0122	1	0.799	0.375	0.287	0.00317
GO:0051301 cell division	0.256	0.0336	1	1	0.405	0.205	0.00465
GO:0000271 polysaccharide biosynthesis	1	0.187	1	1	1	0.00473	1
GO:0043284 biopolymer biosynthesis	1	0.187	1	1	1	0.00473	1
GO:0007049 cell cycle	0.0402	0.317	1	0.00266	0.405	0.00522	0.0409
GO:0000278 mitotic cell cycle	0.176	1	1	0.00796	0.627	0.00576	0.0101
GO:0042144 vacuole fusion, non-autophagic	1	0.319	1	0.00617	1	0.578	1
GO:0000244 assembly of spliceosomal tri-snRNP	1	1	0.00672	1	1	1	1
GO:0000387 spliceosomal snRNP biogenesis	1	1	0.00672	1	1	1	1
GO:0008643	1	0.674	1	0.00795	1	0.347	1

carbohydrate transport							
GO:0006333 chromatin assembly or disassembly	1	0.187	1	0.359	0.00835	1	1
GO:0000074 regulation of cell cycle	0.507	0.665	1	0.264	1	0.817	0.00884
GO:0006119 oxidative phosphorylation	0.00921	1	1	1	1	1	1
GO:0006891 intra-Golgi transport	0.00921	1	1	1	1	1	1
GO:0007008 outer mitochondrial membrane organization and biogenesis	0.00921	1	1	1	1	1	1
GO:0045040 mitochondrial outer membrane protein import	0.00921	1	1	1	1	1	1
GO:0006486 protein amino acid glycosylation	1	0.0316	1	0.0113	1	1	0.439
GO:0009100 glycoprotein metabolism	1	0.0316	1	0.0113	1	1	0.439
GO:0009101 glycoprotein biosynthesis	1	0.0316	1	0.0113	1	1	0.439
GO:0000279 M phase	0.253	0.474	1	0.00799	1	0.0113	0.118
GO:0006732 coenzyme metabolism	1	0.0123	1	0.735	1	0.0755	1
GO:0046907 intracellular transport	0.0133	0.853	1	0.703	1	0.428	1
GO:0051325 interphase	0.62	0.121	1	0.529	1	0.0146	0.0588
GO:0051329	0.62	0.121	1	0.529	1	0.0146	0.0588

interphase of mitotic cell cycle							
GO:0006944 membrane fusion	0.286	0.19	1	0.0147	0.238	0.0764	1
GO:0000086 G2/M transition of mitotic cell cycle	1	0.187	1	0.195	1	0.0382	0.0152
GO:0007088 regulation of mitosis	1	1	1	1	1	1	0.0152
GO:0009250 glucan biosynthesis	1	0.557	1	0.564	1	0.0158	1
GO:0007120 axial bud site selection	1	0.332	1	0.331	1	0.0159	0.156
GO:0008380 RNA splicing	0.0161	1	0.0716	0.738	1	0.311	1
GO:0006269 DNA replication, synthesis of RNA primer	1	0.557	0.02	1	1	1	1
GO:0006301 postreplication repair	1	0.557	0.02	0.564	0.0715	1	1
GO:0000075 cell cycle checkpoint	1	0.447	1	0.202	1	1	0.0209
GO:0006364 rRNA processing	1	0.684	1	0.0214	1	0.688	1
GO:0016072 rRNA metabolism	1	0.684	1	0.0214	1	0.688	1
GO:0043119 positive regulation of physiological process	1	1	1	0.0214	1	0.203	1
GO:0048518 positive regulation of biological process	1	1	1	0.0214	1	0.203	1
GO:0006519 amino acid and derivative metabolism	0.0223	1	1	0.749	1	0.0456	1
GO:0006520	0.0223	1	1	0.749	1	0.0456	1

amino acid metabolism							
GO:0006082 organic acid metabolism	0.024	1	1	0.302	1	0.0583	1
GO:0019752 carboxylic acid metabolism	0.024	1	1	0.302	1	0.0583	1
GO:0000722 telomerase-independent telomere maintenance	1	0.332	1	0.024	1	1	1
GO:0006887 exocytosis	1	1	1	0.024	1	0.34	1
GO:0006280 mutagenesis	1	1	1	1	0.0244	1	1

**Under represented (two sided p-values)**							
**GO id,term**	**001**	**010**	**011**	**100**	**101**	**110**	**111**
GO:0007049 cell cycle	0.0402	0.317	1	0.00266	0.405	0.00522	0.0409
GO:0007059 chromosome segregation	1	0.121	1	0.00338	1	0.18	0.458
GO:0000278 mitotic cell cycle	0.176	1	1	0.00796	0.627	0.00576	0.0101
GO:0000279 M phase	0.253	0.474	1	0.00799	1	0.0113	0.118
GO:0000087 M phase of mitotic cell cycle	0.514	0.314	1	0.0106	1	0.123	0.0653
GO:0007067 mitosis	0.514	0.314	1	0.0106	1	0.123	0.0653
GO:0000910 cytokinesis	0.249	0.0122	1	0.799	0.375	0.287	0.00317
GO:0044248 cellular catabolism	0.272	0.256	1	0.817	1	0.016	0.543
GO:0009056 catabolism	0.282	0.122	1	1	1	0.0161	0.558
GO:0050789 regulation of biological process	0.02	0.423	1	0.87	1	0.236	1
